# Site‐Selective C─H Bond Functionalization of Sugars

**DOI:** 10.1002/anie.202424455

**Published:** 2025-03-31

**Authors:** Elena V. Stepanova, Andrey Shatskiy, Ivan Doroshenko, Peter Dinér, Markus D. Kärkäs

**Affiliations:** ^1^ Department of Chemistry, KTH Royal Institute of Technology Teknikringen 30 Stockholm SE‐100 44 Sweden; ^2^ Tomsk Polytechnic University Tomsk 634050 Russia

**Keywords:** C─H bond activation, Carbohydrates, Photoredox catalysis, Radicals, Visible light

## Abstract

Non‐typical C‐functionalized sugars represent a prominent yet hardly accessible class of biologically‐active compounds. The available synthetic methodologies toward such sugar derivatives suffer either from an extensive use of protecting groups, requiring long and laborious synthetic manipulations, or from limited predictability and noncontrollable site‐selectivity of the employed C‐functionalization reactions. In this work, we disclose an alternative synthetic methodology toward nontypical sugars that allows facile, site‐selective, and stereocontrolled C‐functionalization of sugars through a traceless tethering approach. The described silyl‐based redox‐active tethering group appends directly to the unprotected sugar substrate and mediates the C‐functionalization reaction through a photochemically‐promoted 1,6‐hydrogen atom transfer (HAT) mechanism, while transforming into a readily‐removable silyl protecting group. The protocol is compatible with a variety of unprotected carbohydrate substrates featuring sensitive aglycons and a diverse set of coupling partners, providing a straightforward and scalable route to pharmaceutically relevant C‐functionalized carbohydrate conjugates.

## Graphical abstract

Nontypical sugars represent a prominent, yet hardly, accessible class of biologically‐active compounds. Here, we disclose a technology toward non‐typical sugars that allows facile, and site‐selective functionalization of native sugars. The protocol is compatible with a variety of unprotected carbohydrates featuring sensitive aglycons and a diverse set of coupling partners, providing a straightforward and scalable route to pharmaceutically relevant carbohydrates.

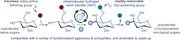

## Introduction

**Figure 1:**
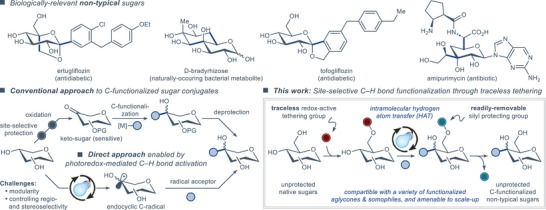
Examples of biologically‐relevant nontypical sugars (the bonds that can be formed through C─H or C─C bond activation are highlighted in blue), synthetic approaches to nontypical sugars through protecting group chemistry and keto‐sugars, and the development of a traceless tethering approach to nontypical sugars as disclosed in this work.

Sugars represent one of the most abundant classes of natural products, exhibiting a multitude of biological functions. Unsurprisingly, functionalized carbohydrates and their conjugates based on both mono‐ and oligosaccharides have found numerous applications in biochemistry, molecular biology, and medicine, in particular, for the development of new pharmaceutical and diagnostic agents.^[^ ^1^, ^2^, ^3^, ^4^, ^5^, ^6^, ^7^ ^]^ The synthetic efforts toward modified sugars have traditionally focused on the regioselective functionalization of carbohydrate hydroxy groups^[^ ^8^, ^9^ ^]^ and the stereoselective synthesis of functionalized *O*‐glycosides.^[^ ^10^, ^11^, ^12^ ^]^ Subsequently, the lability of *O*‐glycosidic bond under enzymatic conditions has stimulated the development of synthetic methods toward *C*‐glycosides, which typically exhibit enhanced metabolic stability.^[^ ^13^, ^14^, ^15^, ^16^, ^17^, ^18^, ^19^, ^20^, ^21^, ^22^, ^23^, ^24^, ^25^, ^26^, ^27^, ^28^, ^29^ ^]^ Considerable efforts were also put fourth toward the synthesis of nontypical C‐functionalized sugars (Figure 1, top), in which various structural motifs are appended to the nonanomeric C‐centers.^[^ ^30^, ^31^, ^32^, ^33^ ^]^ Importantly, such nontypical sugars are frequently found as metabolites in bacteria but not in mammals, making them an attractive synthetic target for the development of pharmaceutical agents with intrinsically low toxicity.^[^ ^34^ ^]^

Modification of the sugar skeleton to access nontypical C‐functionalized sugars has traditionally been achieved through tedious synthetic sequences featuring extensive use of protecting groups and unstable synthetic intermediates, such as keto‐sugars (Figure 1, bottom left).^[^ ^30^, ^31^, ^32^ ^]^ As an alternative, various metal‐ and free radical–mediated sequences proceeding through C─H bond activation have been devised,^[^ ^35^, ^36^, ^37^ ^]^ but these methods have not gained widespread applicability due to being highly substrate‐specific. A far more direct approach to nontypical C‐functionalized sugars was contrived through intermolecular radical‐mediated C─H bond activation propelled by photoredox catalysis.^[^ ^38^, ^39^, ^40^ ^]^ In these systems, C‐functionalization of the carbohydrate skeleton of sugars does not require the use of protecting groups and even employs free hydroxy groups to promote the key C─H bond activation step with an aid of phosphorous, boron, or tin‐based cocatalysts.^[^ ^41^, ^42^, ^43^, ^44^, ^45^, ^46^ ^]^ However, the intermolecular nature of the key hydrogen atom transfer (HAT) step in these manifolds severely limits the control over site‐selectivity of the C‐functionalization reaction. This challenge was most recently addressed in photoredox‐mediated systems proceeding through intramolecular HAT, displaying predictable site‐selectivity.^[^ ^47^, ^48^ ^]^ However, the employed photoredox‐active tethering groups both necessitated the protection of sugar hydroxy groups and required considerably harsh conditions for the removal of the tethering group after the C‐functionalization reaction, decreasing the atom‐economy and limiting the applicability of these systems.

In this work, we sought to develop a photocatalytic system that allows for site‐selective C‐functionalization of sugars with a traceless redox‐active tethering group, providing a straightforward and general route to completely unprotected nontypical sugars (Figure 1, bottom right).

## Results and Discussion

### Reaction Design and Development

**Figure 2:**
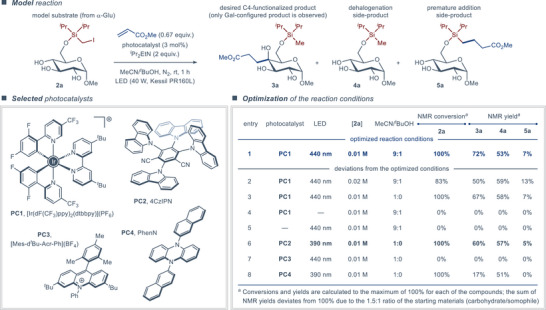
Optimization of reaction conditions featuring the C6‐activated methyl α‐D‐glucoside substrate **2a**.

The envisioned synthetic technology was achieved by devising a redox‐active tethering group that can be regioselectively appended to the unprotected sugar and then transformed into a readily‐removable protecting group upon the photoredox‐mediated C‐functionalization reaction (Figure 1, bottom right). Leveraging the known ability of sterically encumbered chlorosilanes to selectively append to the primary alcohol functionality in unprotected sugars,^[^ ^49^ ^]^ we identified (iodomethyl)di‐isopropylsilyl as the prime candidate for the desired redox‐active tethering group (Figure 2, top).^[^ ^50^, ^51^ ^]^ While this group has typically been employed in manifolds involving intramolecular HAT promoted by light‐mediated palladium and copper catalysis,^[^ ^52^, ^53^, ^54^, ^55^ ^]^ we sought to realize the envisioned reactivity under more benign photoredox conditions in the absence of metal‐based cocatalysts. In the proposed mechanistic sequence, the C─I bond in the tethering group is activated through either one‐electron reduction or halogen atom transfer (XAT) promoted by photoredox catalysis,^[^ ^56^ ^]^ leading to the key α‐silyl C‐radical intermediate. This radical was envisioned to undergo intramolecular HAT with one of the endocyclic C─H bonds in the sugar, followed by addition of the resulting α‐oxyl C‐radical to an appropriate radical acceptor (somophile^[^ ^57^ ^]^) to furnish the desired C‐functionalized product. Notably, as the result of the outlined sequence, the tethering group is transformed into a trivial di‐isopropylmethylsilyl protecting group, which can be easily removed from the C‐functionalized product, while facilitating its purification.

We commenced preliminary optimization of the reaction conditions for the envisioned C‐functionalization with several silylated model substrates derived from protected and unprotected, α‐ and β‐, gluco‐, and galacto‐configured pyranosides (e.g., substrate **2a**, Figure 2, top, see the Supporting Information for details). Under the typical conditions used in Giese‐type photoredox‐mediated C‐couplings with alkyl halides,^[^ ^58^ ^]^ all substrates displayed formation of the C4‐functionalized products with methyl acrylate somophile (e.g., product **3a**), while no detectable side‐products from functionalization of the other positions in the carbohydrate skeleton were observed. Nevertheless, considerable amounts of side‐products were formed due to the reductive dehalogenation of the substrate (e.g., side‐product **4a**) and premature addition of the somophile to α‐silyl C‐radical intermediate (e.g., side‐product **5a**). Decreasing the concentration of the reaction suppressed formation of the premature addition side‐product, while preventing the reductive dehalogenation side‐reaction proved considerably more challenging. Several alternative mechanistic strategies were assessed, including the use of supersilanol,^[^ ^59^, ^60^ ^]^ bis(catecholato)borate,^[^ ^61^ ^]^ formate,^[^ ^62^, ^63^ ^]^ dimanganese decacarbonyl,^[^ ^64^, ^65^ ^]^ as well as carboxylate,^[^ ^66^ ^]^ phosphorous,^[^ ^42^ ^]^ or boron‐based^[^ ^43^ ^]^ cocatalysts, with no positive influence on the reaction outcome (see Tables S1 and S2). Alternative redox‐active silyl tethering groups, including (iodomethyl)diphenylsilyl and (2‐iodophenyl)dimethylsilyl groups, also proved ineffective. Gratifyingly, adjusting the ratio of the coupling partners and further screening of the photocatalyst, sacrificial reductant/XAT‐agent, and the solvent allowed transformation of the methyl α‐D‐glucoside substrate **2a** into the desired C4‐functionalized product **3a** in a reasonably high yield of 72% (Figure 2, bottom, entry 1). The optimal conditions for further investigation of the reaction included the use of [Ir(dF(CF_3_)ppy)_2_(dtbbpy)](PF_6_) (**PC1**) as the photocatalyst under 440 nm LED irradiation, *N*,*N*‐diisopropylethylamine (*^i^* Pr_2_NEt) as the XAT‐agent, and MeCN/*^t^* BuOH 9:1 as the solvent mixture. Increasing the concentration of the substrates or excluding *^t^* BuOH as a cosolvent had a marginally detrimental effect (entries 2 and 3), while conducting the reaction in dark or in the absence of the photocatalyst completely impeded conversion of the substrate (entries 4 and 5). Alternative oxidizing photocatalysts with comparable redox properties to **PC1** proved less effective (entries 6, 7 and 8). Notably, the organic 4CzIPN photocatalyst (**PC2**) delivered product **3a** in a reasonably good yield of 60% (entry 6), exemplifying an attractive variation of the disclosed protocol that does not require any metal‐containing reaction components.

### Reaction Scope and Applicability

**Figure 3:**
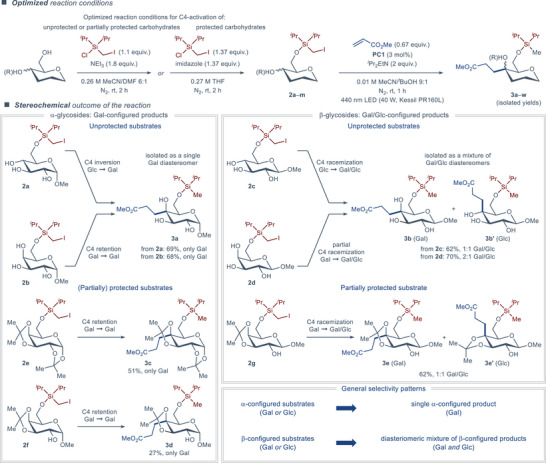
Optimized reaction conditions for functionalization of unprotected and protected carbohydrates as well as stereochemical outcome of the reaction.

Prior to the investigation of the reaction scope, we optimized the procedure for synthesis of the precursor to (iodomethyl)di‐isopropylsilyl tethering group, allowing preparation of the corresponding silyl chloride from commercial reagents in near quantitative yield (97% over three steps) on large scale (30 mmol) in only one day (see the Supporting Information for details). Similarly, the silylation reaction was optimized for both protected and unprotected carbohydrates, allowing fast access to the activated carbohydrate substrates in up to quantitative yields (Figure 3, top).

Gratifyingly, applying the optimized reaction conditions to the unprotected α‐ and β‐configured methyl *D*‐glucoside (**2a** and **2c**, respectively) and *D*‐galactoside substrates (**2b** and **2d**, respectively) revealed that neither C1‐ nor C4‐configuration of the sugar is detrimental to the reaction yield (Figure 3, bottom). The expected products **3a**–**b** were all isolated in high yields (62%–70%) yet displayed an unexpected trend in C4‐stereoselectivity. Both gluco‐ (**2a**) and galacto‐configured (**2b**) α‐glycoside substrates produced a galacto‐configured product **3a** with perfect C4‐stereoselectivity (Gal/Glc 1:0). Conversely, gluco‐ (**2c**) and galacto‐configured (**2d**) β‐glycoside substrates delivered the C4‐functionalized products **3b**/**3b′** as mixtures of C4‐stereoisomers (Gal/Glc 1:1 and 2:1 for the products from **2c** and **2d**, respectively). The same trend was conserved even for C3/C4 protected α‐ (**2e** and **2f**) and β‐glycosides (**2g**), which delivered the C4‐functionalized products as exclusively galacto‐ (**3c** and **3d**, 51% and 27% yield, respectively, Gal/Glc 1:0) or a mixture of gluco/galacto‐configured adducts (**3e**/**3e′**, 62% yield, and Gal/Glc 1:1).

**Figure 4:**
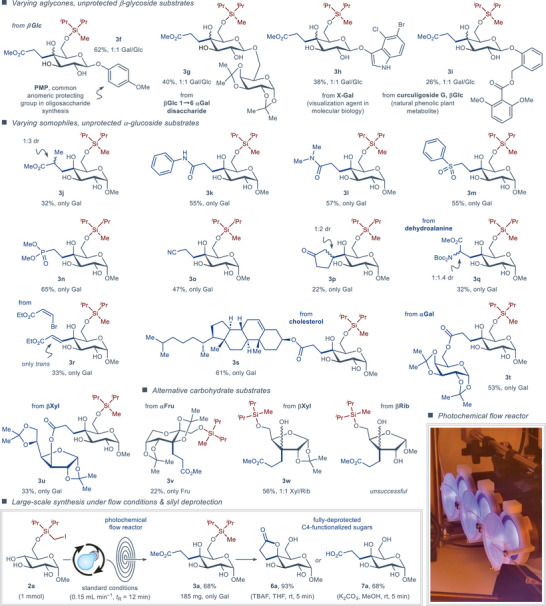
Scope of site‐selective C─H bond functionalization using β‐glycoside substrates with various aglycones and various somophiles, and large‐scale reaction under continuous flow conditions and deprotection of the silyl group.

The broader generality of the disclosed protocol was investigated with a series of glycosides and somophile coupling partners (Figure 4). Several β‐glycosides bearing sensitive aglycones successfully engaged in the reaction with methyl acrylate somophile (Figure 4, top). A common para‐methoxyphenyl anomeric protecting group^[^ ^67^ ^]^ did not impede formation of the expected product (**3f**, 62% yield), despite being sensitive to both oxidative and acidic conditions. Furthermore, employing βGlc 1→6 αGal partially‐protected disaccharide as the substrate delivered the desired C4‐functionalized product **3g** in 40% yield, which could present a significant regioselectivity challenge for the alternative C─H functionalization strategies proceeding through intermolecular HAT. A substrate derived from the common visualization agent X‐Gal,^[^ ^68^, ^69^ ^]^ bearing a polyfunctionalized 5‐bromo‐4‐chloro‐3‐indolyl aglycone, produced the expected product **3h** in a reasonable yield of 39%, despite being designed to undergo facile glycosidic bond cleavage to release the corresponding indigo dye derivative. Finally, employing a naturally‐occurring phenolic glycoside curculigoside G^[^ ^70^ ^]^ as the substrate produced the C4‐functionalized product **3i**, albeit in lower yield (26%), despite bearing an oxidatively‐sensitive aglycone with multiple sites for potential deleterious HAT reactions. The β‐glycoside products **3f**–**i** firmly followed the previously observed stereoselectivity trends, displaying complete racemization of the C4‐stereocenter to deliver the respective adducts as Gal/Glc 1:1 mixtures.

The scope of compatible somophiles was investigated with methyl α‐D‐glucoside **2a** as the model carbohydrate substrate (Figure 4, middle). A wide range of alkene somophiles, including methyl methacrylate, *N*‐phenyl and *N*,*N*‐dimethylacrylamides, phenyl vinyl sulfone, dimethyl vinylphosphonate, and acrylonitrile produced the expected C4‐functionalized products **3j**–**o** in moderate to good yields (32%–65%), while the less electron‐deficient and more sterically‐encumbered 2‐cyclopentenone somophile delivered the coupling product **3p** in lower yield (22%). In line with our continuing efforts in photoredox‐mediated synthesis of unnatural α‐amino acids,^[^ ^71^, ^72^ ^]^ we attempted to use glyoxylate imine‐ and dehydroalanine‐derived somophiles for the synthesis of α‐amino acid–carbohydrate conjugates. Here, while the glyoxylate‐derived radical acceptor proved ineffective, the *N*,*N*‐Boc‐protected dehydroalanine somophile successfully delivered the unconventional α‐amino acid–carbohydrate conjugate **3q** in reasonable yield (32%), complete stereoselectivity at C4 (Gal/Glc 1:0), and partial diastereoselectivity at the α‐center of the resulting α‐amino acid (1:1.4 dr). Employing the ethyl ester of (*Z*)‐3‐bromoacrylic acid as the somophile allowed appending olefinic functionality to the carbohydrate skeleton, delivering exclusively trans‐configured alkene **3r** in 33% yield through an addition‐elimination mechanism. Finally, utilizing complex acrylate esters as somophiles allowed access to a steroid‐decorated sugar (**3s**, 61% yield) and two pseudodisaccharides based on galactose (**3t**, 53% yield) and xylose (**3u**, 33% yield). As expected, all the above α‐glycoside substrates delivered exclusively the galacto‐configured products (Gal/Glc 1:0).

Alternative carbohydrate substrates displayed varying compatibility with the disclosed protocol. A fully‐protected α‐D‐fructopyranose‐derived substrate delivered C3‐functionalized product **3v** in 22% yield, manifesting the same 1,6‐HAT process as for all previously investigated substrates. Similarly, 1,6‐HAT was observed for the partially‐protected β‐D‐xylofuranoside substrate, delivering the C3‐functionalized product **3w** in good yield (56%) as a 1:1 mixture of xylo/ribo‐configured adducts. Unfortunately, *D*‐ribofuranose‐ and *D*‐mannopyranose‐derived substrates proved incompatible with the disclosed reaction.

To demonstrate the practicality of the disclosed protocol, the synthesis of C4‐functionalized α‐D‐galactoside **3a** was carried out on a 1 mmol scale under continuous flow conditions (Figure 4, bottom), displaying the same yield (68%) and stereoselectivity (Gal/Glc 1:0) as for the small‐scale batch reaction (69% yield, Gal/Glc 1:0). Furthermore, deprotection of the silyl ether group from **3a** could be carried out under benign conditions to produce either spirocyclic lactone (**6a**, 93% yield) or linear fully‐unprotected C4‐functionalized product (**7a**, 68% yield) in high efficiency. Notably, the majority of contemporary protocols for C─H functionalization of unprotected carbohydrates via intermolecular HAT employ acidic cocatalysts, which promote the lactonization reaction in situ and prohibit isolation of the linear coupling products, such as **7a**.^[^ ^41^, ^43^, ^44^ ^]^

### Mechanistic Considerations

**Figure 5:**
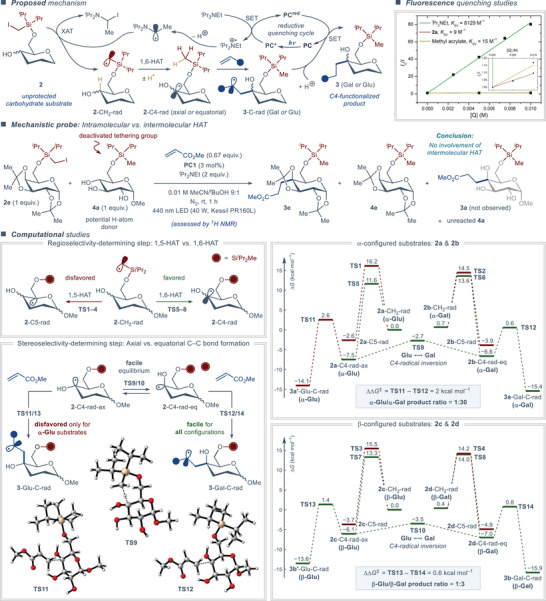
Mechanistic considerations.

According to the proposed mechanism (Figure 5, top left), the reaction commences with excitation of the photocatalyst by the visible light irradiation, followed by reductive quenching of the excited‐state photocatalyst by *^i^* Pr_2_NEt. This mechanistic step was confirmed by the steady‐state fluorescence quenching studies (Figure 5, top right), which identified *^i^* Pr_2_NEt as the only reaction component that effectively quenches the excited photocatalyst. Accordingly, the Stern–Volmer quenching constant for *^i^* Pr_2_NEt (*K*_SV_ = 8129 M^−1^) was found to be almost three orders of magnitude higher than for carbohydrate substrate **2a** and the methyl acrylate somophile (*K*_SV_ = 9 and 15 M^−1^, respectively). Upon quenching through a single‐electron transfer, *^i^* Pr_2_NEt is oxidized to an N‐centered cation‐radical species, which undergoes facile deprotonation to furnish α‐amino C‐centered radical as an active XAT agent.^[^ ^73^ ^]^ The latter abstracts an iodine atom from the carbohydrate substrate **2** to furnish an α‐silyl C‐radical (**2**‐CH_2_‐rad). This radical intermediate then engages in the regiodetermining 1,6‐HAT reaction to produce the C4‐centered α‐hydroxy C‐radical (**2**‐C4‐rad). Finally, the C4‐radical adds to the somophile during the stereodetermining C─C bond‐forming step of the reaction, followed by one‐electron reduction of the formed α‐carbonyl C‐radical (**3**‐C‐rad) by the reduced photocatalyst and subsequent protonation. These steps furnish the desired C4‐functionalized product **3** and complete the photocatalytic cycle. To rule out the contribution of a competitive intermolecular HAT pathway toward **2**‐C4‐rad, a mixture of activated (**2e**) and deactivated (**4a**) carbohydrate substrates was subjected to the standard reaction conditions, producing the expected C4‐functionalized product **3e** only from the activated substrate, along with dehalogenated side‐product **4e** (Figure 5, middle). The deactivated substrate did not engage in the reaction and no traces of the otherwise expected product **3a** were observed, confirming the intramolecular nature of the regiodetermining HAT step.

To gain better insight into the key regio‐ and stereodetermining steps of the reaction, the relevant mechanistic steps were investigated through density functional theory (DFT) calculations at the B3LYP/6–311 + G(d,p) level of theory using the Grimme correction for dispersion (D3) and the conductor‐like polarizable continuum model (CPCM, UFF, and MeCN), as implemented in the Gaussian 16 software (see the Supporting Information for details). The calculations were performed with α‐ and β‐configured model glycoside substrates derived from D‐glucose (**2a** and **2c**) and D‐galactose (**2b** and **2d**) (Figure 5, bottom). The experimentally observed 1,6‐HAT pathway was found to be consistently favorable for all of the substrates, while the alternative 1,5‐HAT pathway was less favorable both kinetically and thermodynamically. The formation of the C4‐centered endocyclic radicals (**2**‐C4‐rad) upon the regioselectivity‐determining 1,6‐HAT step proceeds with reasonably low barriers (Δ*G* ^‡^ = 11.6–13.6 kcal mol^−1^) and a substantial driving force (Δ*G* = −6.1 to −7.5 kcal mol^−1^). Depending on the C4‐configuration of the initial sugar, the latter step produces either axial (**2a/c**‐C4‐rad‐ax) or equatorial (**2b/d**‐C4‐rad‐eq) C4‐radical intermediates displaying pyramidal geometry. Subsequently, these intermediates engage in a rapid axial/equatorial interconversion (Δ*G* ^‡^ = 2.6–4.8 kcal mol^−1^) with only minimal energy differences between the stable conformers (Δ*G* = 0.9 kcal mol^−1^) for both α‐ and β‐configured substrates. The following C─C bond forming reaction between the C4‐radical and the acrylate somophile exhibits significantly higher reaction barriers (Δ*G* ^‡^ = 7.2–10.1 kcal mol^−1^) and considerable driving forces (Δ*G* = −6.6 to −8.9 kcal mol^−1^), identifying this as the stereoselectivity determining step for the overall transformation. The latter implies that the stereochemical outcome of the reaction can be estimated using the Curtin–Hammet principle,^[^ ^74^ ^]^ according to which the ratio of products is defined only by the difference in energy of the barriers leading to the alternative products (ΔΔ*G* ^‡^ = 2.0 and 0.6 kcal mol^−1^ for α‐ and β‐glycosides, respectively) and not by the difference in energy between the unstable intermediates. Thereby, the predicted ratios of gluco/galacto products amount to 1:30 for α‐configured substrates and to 1:3 for β‐configured substrates. Considering the complexity of the evaluated kinetic system, the predicted ratios are in excellent agreement with the experimental results, which displayed a gluco/galacto ratio of 0:1 for α‐glycoside substrates **2a** and **2b**, and either 1:1 or 1:2 gluco/galacto ratio for β‐glycoside substrates **2c** and **2d**, respectively (Figure 3).

## Conclusions

The disclosed traceless tethering approach offers a practical synthetic route for drastic modification of the carbocyclic skeleton in sugars, allowing rapid access to non‐typical C‐functionalized sugar conjugates without the use of protecting groups and with full control over site‐selectivity. The stereochemical outcome of the C‐functionalization reaction is firmly controlled by the anomeric configuration of the sugar substrate, furnishing equatorial addition products for α‐glycosides and a mixture of equatorial and axial adducts for β‐glycosides. The disclosed protocol operates under mild photoredox‐mediated conditions, tolerates a wide range of sensitive aglycones and coupling partners, and is easily amenable to scale‐up, allowing access to C‐alkylated and C‐alkenylated sugar derivatives, including α‐amino acid–decorated and pseudodisaccharide assemblies. Although the described approach illustrates C‐functionalization of sugars proceeding through 1,6‐HAT from the primary alcohol functionality, it also opens up numerous possibilities for further development of related traceless tethering systems targeting activation of other carbohydrate skeleton sites.

## Author Contributions

M.D.K. conceptualized and directed the project. A.S. conceived the project and drafted the manuscript. E.V.S, A.S., and I.D. performed the experimental part of the project. P.D. designed and conducted the computational studies. All authors contributed to the discussion of the results, as well as reviewing and editing of the manuscript.

## Conflict of Interests

The authors declare no conflict of interest.

## Supporting information



Supporting Information

## Data Availability

The data that support the findings of this study are available in the supplementary material of this article.
