# Supplementary material for: Site‐Selective C─H Bond Functionalization of Sugars
Source: Angew Chem Int Ed Engl. 2025 Mar 31;64(19):e202424455. doi: 10.1002/anie.202424455 (PMC12051779; doi:10.1002/anie.202424455)
Supplement: Supplementary file 1 — Supporting Information [file ANIE-64-e202424455-s001.pdf]

Supporting Information

Site-Selective C–H Bond Functionalization of Sugars

Elena V. Stepanova,[‡,‖,†] Andrey Shatskiy,[‡,†] Ivan Doroshenko,[‡,‖] Peter Dinér,[‡]

Markus D. Kärkäs[‡]*

[‡] Department of Chemistry, KTH Royal Institute of Technology, SE-100 44 Stockholm, Sweden

[‖] Tomsk Polytechnic University, 634050 Tomsk, Russia

[†] These authors contributed equally

* Corresponding author e-mail: karkas@kth.se

Table of Contents

1. General methods S1

2. Synthesis of starting materials S2

2.1 Synthesis of chloro(iodomethyl)diisopropylsilane (SI-1) S2

2.1 General procedures for synthesis of starting materials S3

2.1.1 General procedure A: Silylation of non-protected carbohydrate substrates with SI-1 .... S3

2.1.2 General procedure B: Silylation of protected carbohydrate substrates with SI-1 .... S3

2.1.3 General procedure C: Synthesis of acrylate esters S3

2.2 Analytical data for starting materials S4

Compound **2a** (methyl 6-*O*-(iodomethyl)diisopropylsilyl-α-D-glucopyranoside) S4

Compound **2b** (methyl 6-*O*-(iodomethyl)diisopropylsilyl-α-D-galactopyranoside) S4

Compound **2c** (methyl 6-*O*-(iodomethyl)diisopropylsilyl-β-D-glucopyranoside) S4

Compound **2d** (methyl 6-*O*-(iodomethyl)diisopropylsilyl-β-D-galactopyranoside) S5

Compound **2e** (1,2:3,4-di-*O*-isopropylidene-6-*O*-(iodomethyl)diisopropylsilyl-α-D-galactopyranose) S5

Compound **2f** (methyl 3,4-*O*-isopropylidene-6-*O*-(iodomethyl)diisopropylsilyl-α-D-galactopyranoside) S5

Compound **2g** (methyl 3,4-*O*-isopropylidene-6-*O*-(iodomethyl)diisopropylsilyl-β-D-galactopyranoside) S6

Compound **2h** (4-methoxyphenyl 6-*O*-(iodomethyl)diisopropylsilyl-β-D-glucopyranoside) S6

Compound **2i** (6-*O*-(6-*O*-(iodomethyl)diisopropylsilyl-β-D-glucopyranosyl)-1,2:3,4-di-*O*-isopropylidene α-D-galactopyranose) S7

Compound **2j** ((5-bromo-4-chloro-1*H*-indol-3-yl)-6-*O*-(iodomethyl)diisopropylsilyl-β-D-glucopyranoside) S7

Compound **2k** ((((2,6-dimethoxybenzoyl)oxy)methyl)phenyl 6-*O*-(iodomethyl)diisopropylsilyl-β-D-glucopyranoside) S8

Compound **2l** (2,3:4,5-di-*O*-isopropylidene-1-*O*-(iodomethyl)diisopropylsilyl-α-D-fructopyranose) S8

Compound **2m** (1,2-*O*-isopropylidene-5-*O*-(iodomethyl)diisopropylsilyl-α-D-xylofuranose) S8

Compound **SI-2** (cholesteryl acrylate) S9

Compound **SI-3** (6-*O*-acryloyl-1,2:3,4-di-*O*-isopropylidene α-D-galactopyranose) S9

Compound **SI-4** (3-*O*-acryloyl-1,2:5,6-di-*O*-isopropylidene α-D-glucofuranose) S10

3. Optimization of the reaction conditions S11

4. Investigation of the substrate scope S21

4.1 General procedure D: Photoredox-mediated C–H functionalization of the silylated carbohydrate substrates S21

4.2 Large-scale photochemical flow reaction S22

4.3 Removal of the silyl tethering group after the photoreaction S23

4.3.1 Removal of the silyl tethering group towards a lactone product S23

4.3.2 Removal of the silyl tethering group towards a linear C-functionalized product S23

4.4 Analytical data for C-functionalized carbohydrate products..S23

Compound **3a** (methyl 6-*O*-methyldiisopropylsilyl-4-(3-methoxy-3-oxopropyl)-α-D-galactopyranoside).. S23

Compound **4a** (methyl 6-*O*-methyldiisopropylsilyl-α-D-glucopyranoside)................................. S24

Compounds **3b**/**3b'** (methyl 6-*O*-methyldiisopropylsilyl-4-(3-methoxy-3-oxopropyl)-β-D-galacto/glucopyranoside) S24

Compound **3c** (1,2:3,4-di-*O*-isopropylidene-6-*O*-methyldiisopropylsilyl-4-(3-methoxy-3-oxopropyl)-α-D-galactopyranose)... S25

Compound **3d** (methyl 6-*O*-methyldiisopropylsilyl-3,4-O-isopropylidene-4-(3-methoxy-3-oxopropyl)-α-D-galactopyranoside).. S25

Compounds **3e**/**3e'** (methyl 6-*O*-methyldiisopropylsilyl-3,4-*O*-isopropylidene-4-(3-methoxy-3-oxopropyl)-β-D-galacto/glucopyranoside) S26

Compound **3f** (4-methoxyphenyl 6-*O*-methyldiisopropylsilyl-4-(3-methoxy-3-oxopropyl)-β-D-galacto/glucopyranoside) S27

Compound **3g** (6-*O*-(6-*O*-methyldiisopropylsilyl-4-(3-methoxy-3-oxopropyl)-β-D-glucopyranosyl)-1,2:3,4-di-*O*-isopropylidene-α-D-galacto/glucopyranoside)....................................... S27

Compound **3h** ((5-bromo-4-chloro-1*H*-indol-3-yl)-6-*O*-methyldiisopropylsilyl-4-(3-methoxy-3-oxopropyl)-β-D-galacto/glucopyranoside) S28

Compound **3i** ((((2,6-dimethoxybenzoyl)oxy)methyl)phenyl 6-*O*-methyldiisopropylsilyl-4-(3-methoxy-3-oxopropyl)-β-D-galacto/glucopyranoside) S28

Compound **3j** (methyl 6-*O*-methyldiisopropylsilyl-4-(3-methoxy-2-methyl-3-oxopropyl)-α-D-galactopyranoside).. S29

Compound **3k** (methyl 6-*O*-methyldiisopropylsilyl-4-(3-oxo-3-(phenylamino)propyl)-α-D-galactopyranoside).. S30

Compound **3l** (methyl 6-*O*-methyldiisopropylsilyl-4-(3-(dimethylamino)-3-oxopropyl)-α-D-galactopyranoside).. S30

Compound **3m** (methyl 6-*O*-methyldiisopropylsilyl-4-(2-(phenylsulfonyl)ethyl)-α-D-galactopyranoside).. S31

Compound **3n** (methyl 6-*O*-methyldiisopropylsilyl-4-(2-(dimethoxyphosphoryl)ethyl)-α-D-galactopyranoside).. S31

Compound **3o** (methyl 6-*O*-methyldiisopropylsilyl-4-(2-cyanoethyl)-α-D-galactopyranoside) ... S31

Compound **3p** (methyl 6-*O*-methyldiisopropylsilyl-4-(3-oxocyclopentyl)-α-D-galactopyranoside) S32

Compound **3q** (methyl 6-*O*-methyldiisopropylsilyl-4-(2-(bis(*tert*-butoxycarbonyl)amino)-3-methoxy-3-oxopropyl)-α-D-galactopyranoside)... S32

Compound **3r** (methyl *E*-6-*O*-methyldiisopropylsilyl-4-(3-ethoxy-3-oxoprop-1-en-1-yl)-α-D-galactopyranoside).. S33

Compound **3s** (methyl 6-*O*-methyldiisopropylsilyl-4-(3-cholesteryloxy-3-oxopropyl)-α-D-galactopyranoside) S34

Compound **3t** (methyl 6-*O*-methyldiisopropylsilyl-4-(3-(6-*O*-(1,2:3,4-di-*O*-isopropylidene-α-D-galactopyranos)yl)-3-oxopropyl)-α-D-galactopyranoside) S34

Compound **3u** (methyl 6-*O*-methyldiisopropylsilyl-4-(3-(3-*O*-(1,2:5,6-di-*O*-isopropylidene-α-D-glucofuranos)yl)-3-oxopropyl)-α-D-galactopyranoside) S35

Compound **3v** (2,3:4,5-di-*O*-isopropylidene-3-(3-methoxy-3-oxopropyl)-1-*O*-methyldiisopropylsilyl-β-D-fructopyranose) S35

Compounds **3w/3w'** (1,2-*O*-isopropylidene-3-(3-methoxy-3-oxopropyl)-5-*O*-methyldiisopropylsilyl-α-D-xylo/ribofuranose) S36

Compound **6a** ((5*R*,6*R*,8*S*,9*R*,10*R*)-6-(((diisopropyl(methyl)silyl)oxy)methyl)-9,10-dihydroxy-8-methoxy-1,7-dioxaspiro[4.5]decan-2-one) S37

Compound **7a** (methyl 6-*O*-methyldiisopropylsilyl-4-(2-carboxyethyl)-α-D-galactopyranoside). S37

5. Mechanistic studies S38

5.1 Fluorescence quenching studies S38

5.2 Intramolecular vs. intermolecular hydrogen atom transfer control experiment S38

5.3 Computational studies S40

5.3.1 Computational methods and energies of the calculated species and transition states S40

5.3.2 Cartesian coordinates S42

2a-CH_2-rad S42

TS1 .... S43

2a-C5-rad S44

2b-CH_2-rad S45

2c-CH_2-rad S46

TS3 .... S47

2c-C5-rad S48

2d-CH_2-rad S49

TS4 .... S50

2d-C5-rad S51

TS5 .... S52

2a-C4-rad-ax S53

TS6 .... S54

2b-C4-rad-eq S55

TS7 .... S56

2c-C4-rad-ax S57

TS8 .... S58

2d-C4-rad-eq S59

TS9 .... S60

TS10 .... S61

TS11 .... S62

3a'-Glu-C-rad S63

TS12 .... S64

3a-Gal-C-rad S65

TS13 .... S66

3b'-Glu-C-rad S67

TS14 .... S68

3b-Gal-C-rad S69

4. NMR spectra S70

^{1}H NMR (500 MHz, $CDCl_3$) of compound **SI-1** S70

^{13}C{^{1}H} NMR (126 MHz, $CDCl_3$) of compound **SI-1** S71

HSQC of compound **SI-1** S72

^{1}H NMR (500 MHz, $CDCl_3$) of compound **2a** S73

^{13}C{^{1}H} NMR (126 MHz, $CDCl_3$) of compound **2a** S74

COSY of compound **2a** S75

HSQC of compound **2a** S76

^{1}H NMR (500 MHz, $CDCl_3$) of compound **2b** S77

^{13}C{^{1}H} NMR (126 MHz, $CDCl_3$) of compound **2b** S78

COSY of compound **2b** S79

HSQC of compound **2b** S80

^{1}H NMR (500 MHz, $CDCl_3$) of compound **2c** S81

^{13}C{^{1}H} NMR (126 MHz, $CDCl_3$) of compound **2c** S82

COSY of compound **2c** S83

HSQC of compound **2c** S84

^{1}H NMR (500 MHz, $CDCl_3$) of compound **2d** S85

^{13}C{^{1}H} NMR (126 MHz, $CDCl_3$) of compound **2d** S86

COSY of compound **2d** S87

HSQC of compound **2d** S88

^{1}H NMR (500 MHz, $CDCl_3$) of compound **2e** S89

^{13}C{^{1}H} NMR (126 MHz, $CDCl_3$) of compound **2e** S90

COSY of compound **2e** S91

HSQC of compound **2e** S92

^{1}H NMR (500 MHz, $CDCl_3$) of compound **2f** S93

^{13}C{^{1}H} NMR (126 MHz, $CDCl_3$) of compound **2f** S94

COSY of compound **2f** S95

HSQC of compound **2f** S96

^{1}H NMR (500 MHz, $CDCl_3$) of compound **2g** S97

$^{13}C\{^{1}H\}$ NMR (126 MHz, $CDCl_3$) of compound **2g** S98
COSY of compound **2g** S99
HSQC of compound **2g** S100
^{1}H NMR (500 MHz, $CDCl_3$) of compound **2h** S101
$^{13}C\{^{1}H\}$ NMR (126 MHz, $CDCl_3$) of compound **2h** S102
COSY of compound **2h** S103
HSQC of compound **2h** S104
^{1}H NMR (500 MHz, $CDCl_3$) of compound **2i** S105
$^{13}C\{^{1}H\}$ NMR (126 MHz, $CDCl_3$) of compound **2i** S106
COSY of compound **2i** S107
HSQC of compound **2i** S108
^{1}H NMR (500 MHz, MeOD) of compound **2j** S109
$^{13}C\{^{1}H\}$ NMR (126 MHz, MeOD) of compound **2j** S110
COSY of compound **2j** S111
HSQC of compound **2j** S112
^{1}H NMR (500 MHz, MeOD) of compound **2k** S113
^{1}H NMR (500 MHz, $CDCl_3$) of compound **2l** S114
$^{13}C\{^{1}H\}$ NMR (126 MHz, $CDCl_3$) of compound **2l** S115
COSY of compound **2l** S116
HSQC of compound **2l** S117
^{1}H NMR (500 MHz, $CDCl_3$) of compound **2m** S118
$^{13}C\{^{1}H\}$ NMR (126 MHz, $CDCl_3$) of compound **2m** S119
COSY of compound **2m** S120
HSQC of compound **2m** S121
^{1}H NMR (500 MHz, $CDCl_3$) of compound **SI-2** S122
$^{13}C\{^{1}H\}$ NMR (126 MHz, $CDCl_3$) of compound **SI-2** S123
COSY of compound **SI-2** S124
HSQC of compound **SI-2** S125
^{1}H NMR (500 MHz, $CDCl_3$) of compound **SI-3** S126
$^{13}C\{^{1}H\}$ NMR (126 MHz, $CDCl_3$) of compound **SI-3** S127
COSY of compound **SI-3** S128
HSQC of compound **SI-3** S129
^{1}H NMR (500 MHz, $CDCl_3$) of compound **SI-4** S130
$^{13}C\{^{1}H\}$ NMR (126 MHz, $CDCl_3$) of compound **SI-4** S131
COSY of compound **SI-4** S132
HSQC of compound **SI-4** S133
^{1}H NMR (500 MHz, $CDCl_3$) of compound **3a** S134
$^{13}C\{^{1}H\}$ NMR (126 MHz, $CDCl_3$) of compound **3a** S135

COSY of compound **3a** S136
HSQC of compound **3a** S137
HMBC of compound **3a** S138
^{1}H NMR (500 MHz, $CDCl_3$) of compound **4a** S139
^{13}C{^{1}H} NMR (126 MHz, $CDCl_3$) of compound **4a** S140
COSY of compound **4a** S141
HSQC of compound **4a** S142
^{1}H NMR (500 MHz, $CDCl_3$) of compound **3b/3b’** (from **2c**) S143
^{13}C{^{1}H} NMR (126 MHz, $CDCl_3$) of compound **3b/3b’** (from **2c**) S144
COSY of compound **3b/3b’** (from **2c**) S145
HSQC of compound **3b/3b’** (from **2c**) S146
^{1}H NMR (500 MHz, $CDCl_3$) of compound **3b/3b’** (from **2d**) S147
^{1}H NMR (500 MHz, $CDCl_3$) of compound **3c** S148
^{13}C{^{1}H} NMR (126 MHz, $CDCl_3$) of compound **3c** S149
COSY of compound **3c** S150
HSQC of compound **3c** S151
^{1}H NMR (500 MHz, $CDCl_3$) of compound **3d** S152
^{13}C{^{1}H} NMR (126 MHz, $CDCl_3$) of compound **3d** S153
COSY of compound **3d** S154
HSQC of compound **3d** S155
HMBC of compound **3d** S156
^{1}H NMR (500 MHz, $CDCl_3$) of compound **3e** S157
^{13}C{^{1}H} NMR (126 MHz, $CDCl_3$) of compound **3e** S158
COSY of compound **3e** S159
HSQC of compound **3e** S160
NOESY of compound **3e** S161
^{1}H NMR (500 MHz, $CDCl_3$) of compound **3e’** S162
^{13}C{^{1}H} NMR (126 MHz, $CDCl_3$) of compound **3e’** S163
COSY of compound **3e’** S164
HSQC of compound **3e’** S165
HMBC of compound **3e’** S166
^{1}H NMR (500 MHz, $CDCl_3$) of compound **3f** S167
^{13}C{^{1}H} NMR (126 MHz, $CDCl_3$) of compound **3f** S168
COSY of compound **3f** S169
HSQC of compound **3f** S170
^{1}H NMR (500 MHz, $CDCl_3$) of compound **3g** S171
^{13}C{^{1}H} NMR (126 MHz, $CDCl_3$) of compound **3g** S172
COSY of compound **3g** S173

HSQC of compound **3g** S174
^{1}H NMR (500 MHz, acetone-d_6) of compound **3h** S175
^{13}C{^{1}H} NMR (126 MHz, acetone-d_6) of compound **3h** S176
COSY of compound **3h** S177
HSQC of compound **3h** S178
^{1}H NMR (500 MHz, $CDCl_3$) of compound **3i** S179
^{13}C{^{1}H} NMR (126 MHz, $CDCl_3$) of compound **3i** S180
COSY of compound **3i** S181
^{1}H NMR (500 MHz, $CDCl_3$) of compound **3j** S182
^{13}C{^{1}H} NMR (126 MHz, $CDCl_3$) of compound **3j** S183
COSY of compound **3j** S184
HSQC of compound **3j** S185
^{1}H NMR (500 MHz, $CDCl_3$) of compound **3k** S186
^{13}C{^{1}H} NMR (126 MHz, $CDCl_3$) of compound **3k** S187
COSY of compound **3k** S188
HSQC of compound **3k** S189
^{1}H NMR (500 MHz, $CDCl_3$) of compound **3l** S190
^{13}C{^{1}H} NMR (126 MHz, $CDCl_3$) of compound **3l** S191
COSY of compound **3l** S192
HSQC of compound **3l** S193
^{1}H NMR (500 MHz, $CDCl_3$) of compound **3m** S194
^{13}C{^{1}H} NMR (126 MHz, $CDCl_3$) of compound **3m** S195
COSY of compound **3m** S196
HSQC of compound **3m** S197
^{1}H NMR (500 MHz, $CDCl_3$) of compound **3n** S198
^{13}C{^{1}H} NMR (126 MHz, $CDCl_3$) of compound **3n** S199
COSY of compound **3n** S200
HSQC of compound **3n** S201
^{1}H NMR (500 MHz, $CDCl_3$) of compound **3o** S202
^{13}C{^{1}H} NMR (126 MHz, $CDCl_3$) of compound **3o** S203
COSY of compound **3o** S204
HSQC of compound **3o** S205
^{1}H NMR (500 MHz, $CDCl_3$) of compound **3p** S206
^{13}C{^{1}H} NMR (126 MHz, $CDCl_3$) of compound **3p** S207
COSY of compound **3p** S208
HSQC of compound **3p** S209
^{1}H NMR (500 MHz, $CDCl_3$) of compound **3q** S210
^{13}C{^{1}H} NMR (126 MHz, $CDCl_3$) of compound **3q** S211

COSY of compound **3q** S212
HSQC of compound **3q** S213
^{1}**H NMR** (500 MHz, $CDCl_3$) of compound **3r** S214
^{13}**C{**^{1}**H} NMR** (126 MHz, $CDCl_3$) of compound **3r** S215
COSY of compound **3r** S216
^{1}**H NMR** (500 MHz, $CDCl_3$) of compound **3s** S217
^{13}**C{**^{1}**H} NMR** (126 MHz, $CDCl_3$) of compound **3s** S218
COSY of compound **3s** S219
HSQC of compound **3s** S220
^{1}**H NMR** (500 MHz, $CDCl_3$) of compound **3t** S221
^{13}**C{**^{1}**H} NMR** (126 MHz, $CDCl_3$) of compound **3t** S222
COSY of compound **3t** S223
HSQC of compound **3t** S224
^{1}**H NMR** (500 MHz, $CDCl_3$) of compound **3u** S225
^{13}**C{**^{1}**H} NMR** (126 MHz, $CDCl_3$) of compound **3u** S226
COSY of compound **3u** S227
HSQC of compound **3u** S228
^{1}**H NMR** (500 MHz, $CDCl_3$) of compound **3v** S229
^{13}**C{**^{1}**H} NMR** (126 MHz, $CDCl_3$) of compound **3v** S230
COSY of compound **3v** S231
HSQC of compound **3v** S232
^{1}**H NMR** (500 MHz, $CDCl_3$) of compound **3w** S233
^{13}**C{**^{1}**H} NMR** (126 MHz, $CDCl_3$) of compound **3w** S234
COSY of compound **3w** S235
HSQC of compound **3w** S236
NOESY of compound **3w** S237
^{1}**H NMR** (500 MHz, $CDCl_3$) of compound **3w’** S238
^{13}**C{**^{1}**H} NMR** (126 MHz, $CDCl_3$) of compound **3w’** S239
COSY of compound **3w’** S240
HSQC of compound **3w’** S241
^{1}**H NMR** (500 MHz, $CDCl_3$) of compound **6a** S242
^{13}**C{**^{1}**H} NMR** (126 MHz, $CDCl_3$) of compound **6a** S243
COSY of compound **6a** S244
HSQC of compound **6a** S245
HMBC of compound **6a** S246
^{1}**H NMR** (500 MHz, $CDCl_3$) of compound **7a** S247
^{13}**C{**^{1}**H} NMR** (126 MHz, $CDCl_3$) of compound **7a** S248
COSY of compound **7a** S249

HSQC of compound **7a** S250
HMBC of compound **7a** S251
References **S252**

1. General methods

All reactions were performed using reagents from commercial suppliers (Merc, VWR, Fisher) without additional purification, unless otherwise stated. HPLC grade solvents were used for the reactions and purification of products. Acetonitrile was dried by refluxing over CaH_2 and subsequent distillation under nitrogen. CH_2Cl_2, DMF, DMSO and toluene were dried using a Solvent Purification System (Pure Process Technology). Other anhydrous solvents used for optimization of the reaction conditions were dried over activated 3 Å or 4 Å molecular sieves. Thin-layer chromatography (TLC) was performed on aluminum-supported silica gel 60 F_{254} TLC plates (Merck, Art. No. 1.05554). The TLC plates were visualized either under UV light (254 nm) or by immersing in a methanolic solution of H_3PO_4 (ca. 5%) followed by heating with a heat-gun (ca. 300 °C). Column chromatography was performed with high-purity grade silica gel (Merk, Art. No. 60738 for the C-functionalized products **3–7** and Art. No. 288608 for all other products). Product **3w'** was purified by reversed-phase column chromatography with Isolute C18(EC) as the stationary phase. NMR spectra were recorded on Bruker Avance DMX 500 MHz or Bruker Ascend 400 MHz NMR spectrometers. The ^{1}H NMR chemical shifts were calibrated against the signals of residual protic solvents: $CHCl_3$ (7.26 ppm), CHD_2OD (3.31 ppm), or acetone-d_5 (2.05 ppm). The ^{13}C NMR chemical shifts were calibrated against the solvent signals ($CDCl_3$, 77.00 ppm; CD_3OD, 49.00 ppm; acetone-d_6, 29.84 ppm). The chemical shifts for NMR are reported in ppm and peak multiplicities are denoted as s (singlet), d (doublet), t (triplet), q (quartet), quint (quintet), sept (septet), dd (doublet of doublets), ddd (doublet of doublet of doublets), dddd (doublet of doublet of doublet of doublets), dt (doublet of triplets), dq (doublet of quartets), dtd (doublet of triplet of doublets), td (triplet of doublets), ABq (AB-quartet), and m (multiplet); prefix br. is used to denote broadened signals. Assignment of the signals in the NMR spectra was performed with the aid of 2D-NMR experiments (COSY, HSQC, HMBC, NOESY). Note, for compounds **2a–2m** an aliased signal from $SiCH_2I$ carbon is observed at 140–160 ppm in HSQC due to the ^{13}C NMR signal of this group being outside of the spectral window (ca. −20 ppm). High resolution mass-spectra (electro-spray ionization, ESI) were recorded in a positive ion mode on 2×10^{-5} M solutions of the analytes in MeCN, using Bruker micrOTOF II or Agilent 6530 LC/Q-TOF mass spectrometers.

2. Synthesis of starting materials

2.1 Synthesis of chloro(iodomethyl)diisopropylsilane (SI-1)

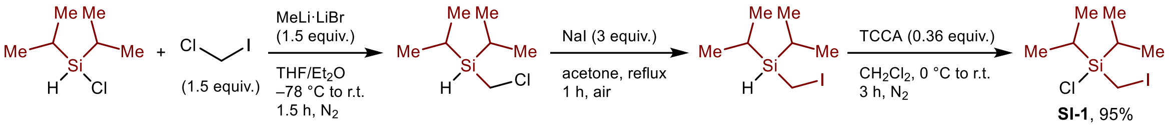

The synthetic procedure was adapted from literature.[1] Chlorodiisopropylsilane (5.12 mL, 30 mmol, 1 equiv.) and chloroiodomethane (3.275 mL, 45 mmol, 1.5 equiv.) were dissolved in THF (35 mL) under nitrogen, and the stirred solution was cooled on an acetone/CO_2 bath to –78 °C. Solution of MeLi·LiBr (1.5 M in Et_2O, 30 mL, 45 mmol, 1.5 equiv.) was added to the reaction mixture dropwise (ca. 1 mL/min). The reaction mixture was allowed to warm to room temperature while stirring over 1.5 h, saturated aqueous solution of NH_4Cl (100 mL) was added, and the mixture was extracted with hexanes (3 × 100 mL), dried over Na_2SO_4, filtered, and concentrated on rotary evaporator (200 mbar, 40 °C bath temperature) to give (chloromethyl)dissopropylsilane as a pale-yellow oil. The latter product was dissolved in acetone (35 mL) and NaI (13.49 g, 90 mmol, 3 equiv.) was added under air. The reaction mixture was refluxed for 1 h (75 °C oil bath temperature) and allowed to cool to room temperature. Aqueous solution of $Na_2S_2O_3$ (10 wt.%, 100 mL) was added to the reaction mixture and the resulting colorless solution was extracted with hexanes (3 × 100 mL). The combined organic phases were washed with water (2 × 150 mL), dried over Na_2SO_4, filtered, and concentrated on a rotary evaporator (200 mbar, 40 °C bath temperature) to give (iodomethyl)dissopropylsilane as colorless oil. The latter product was dissolved in anhydrous CH_2Cl_2 (10 mL) and added dropwise (over 40 min) to a cooled (0 °C, ice/water bath) stirred solution of trichloroisocyanuric acid (TCCA, 2.51 g, 10.8 mmol, 0.36 equiv.) in anhydrous CH_2Cl_2 (15 mL) under nitrogen. The reaction mixture was allowed to warm to room temperature over 3 h, filtered through celite, washing with CH_2Cl_2 (4 × 10 mL). The filtrate was concentrated on a rotary evaporator (200 mbar, 40 °C bath temperature), and hexanes (70 mL) was added to obtain a pale suspension. The mixture was filtered through celite, washing with hexanes (3 × 20 mL). The solution was concentrated on rotary evaporator (200 mbar, 40 °C bath temperature) to give the desired chloro(iodomethyl)diisopropylsilane product **SI-1** as purple oil (8.42 g, 97% yield, including ca. 2% hexane).

Important! Product **SI-1** and all of the intermediate products for its synthesis are volatile liquids; therefore, high vacuum should be avoided when removing the solvents from these compounds. A small amount of hexane might remain in the final product after concentration; however, further exposure of the product to vacuum leads to significantly lower yields, while the trace amounts of hexane do not affect the following reactions employing **SI-1**.

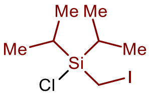

^{1}H NMR (500 MHz, $CDCl_3$) δ 2.22 (s, 2H), 1.43 (sept, J = 7.4 Hz, 2H), 1.14 (d, J = 7.5 Hz, 6H), 1.12 (d, J = 7.2 Hz, 6H).

^{13}C{^{1}H} NMR (126 MHz, $CDCl_3$) δ 17.42, 17.09, 13.6, −20.7.

The spectroscopic data are in agreement with the literature.[1]

2.1 General procedures for synthesis of starting materials

2.1.1 General procedure A: Silylation of non-protected carbohydrate substrates with SI-1

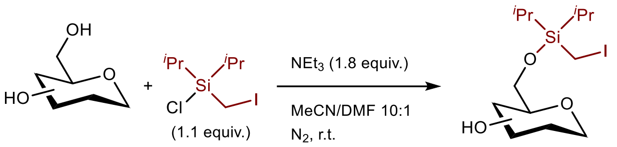

An unprotected sugar substrate (3 mmol) was dissolved in anhydrous MeCN (10 mL), followed by addition of Et_3N (0.72 mL, 5.4 mmol, 1.8 equiv.) and anhydrous DMF (1.5 mL) under nitrogen. Subsequently, solution of silyl chloride **SI-1** (960 mg, 3.3 mmol, 1.1 equiv.) in anhydrous MeCN (5 mL) was added dropwise to the reaction mixture under stirring. Upon reaching full conversion of the starting carbohydrate (monitored by TLC), the reaction mixture was concentrated on rotary evaporator and DMF was removed by co-evaporation with toluene (5 × 5 mL). The resulting crude product was purified by column chromatography.

2.1.2 General procedure B: Silylation of protected carbohydrate substrates with SI-1

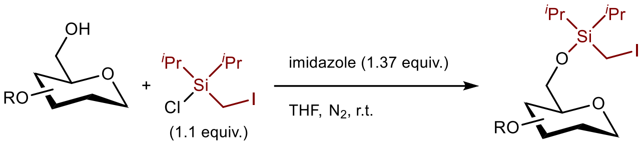

A protected or partially-protected sugar substrate (5.76 mmol) and imidazole (784 g, 11.52 mmol, 1.37 equivalents) were dried in vacuo and dissolved in anhydrous THF (15 mL) under nitrogen. Solution of silyl chloride **SI-1** (2.3 g, 7.9 mmol, 1.37 equiv.) in anhydrous THF (6 mL) was added dropwise to the reaction mixture under stirring. Upon reacting full conversion of the starting carbohydrate (monitored by TLC), water (20 mL) was added to the reaction mixture. The organic phase was separated and the aqueous phase was extracted with CH_2Cl_2 (3 × 20 mL). The combined organic phases were dried over Na_2SO_4, filtered, and concentrated on a rotary evaporator. The resulting crude product was purified by column chromatography.

2.1.3 General procedure C: Synthesis of acrylate esters

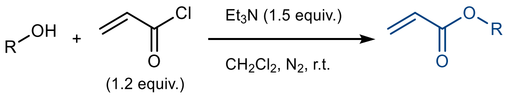

An alcohol (1 mmol) was dissolved in anhydrous CH_2Cl_2 (10 mL), Et_3N (200 μL, 1.5 mmol, 1.5 equiv.), and acryloyl chloride (98 μL, 1.2 mmol, 1.2 equiv.) was added to the reaction mixture under nitrogen. Upon reacting full conversion of the starting alcohol (monitored by TLC), the reaction mixture was concentrated on a rotary evaporator and the crude product was purified by column chromatography.

2.2 Analytical data for starting materials

Compound **2a** (methyl 6-*O*-(iodomethyl)diisopropylsilyl-α-D-glucopyranoside)

Synthesized according to **General procedure A** from methyl β-D-glucopyranoside on 3.0 mmol scale. The crude product was purified by column chromatography with gradient CH_2Cl_2/MeOH 25:1→10:1 as the eluent, resulting in compound **2a** as a white amorphous solid (810 mg, 90% yield).

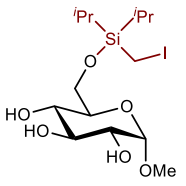

^{1}H NMR (500 MHz, $CDCl_3$) δ 4.75 (d, *J* = 3.8 Hz, 1H, H-1), 4.02 (dd, *J* = 10.7, 4.6 Hz, 1H, H-6a), 3.97 (dd, *J* = 10.7, 4.3 Hz, 1H, H-6b), 3.74 (dd~br.t, *J* = 9.1 Hz, 1H, H-3), 3.65 (ddd~dt, *J* = 9.1, 4.5 Hz, 1H, H-5), 3.59 (dd, *J* = 9.7, 8.6 Hz, 1H, H-4), 3.52 (dd, *J* = 9.5, 3.8 Hz, 1H, H-2), 3.43 (s, 3H, OCH_3), 2.12 (s, 2H, CH_2I), 1.35 – 1.19 (m, 2H, (C<u>H</u>(CH_3)$_2$), 1.08, 1.09, 1.11 (all s, 12H, CH(C<u>H</u>$_3$)$_2$).

^{13}C{^{1}H} NMR (126 MHz, $CDCl_3$) δ 99.1 (C-1), 74.7 (C-3), 72.2 (C-2), 71.6 (C-4), 70.7 (C-5), 64.4 (C-6), 55.4 (OCH_3), 17.6 (CH(<u>C</u>H$_3$)$_2$), 17.4 (CH(<u>C</u>H$_3$)$_2$), 12.2 (<u>C</u>H(CH_3)$_2$).

***R*$_f$** = 0.34 (CH_2Cl_2/MeOH 10:1, brown color upon treatment with 5% methanolic H_3PO_4 and heating)

HRMS (ESI, m/z): calcd. for $[C_{14}H_{29}IO_6SiNa]^+$, 471.0670; found, 471.0669

Compound **2b** (methyl 6-*O*-(iodomethyl)diisopropylsilyl-α-D-galactopyranoside)

Synthesized according to **General procedure A** from methyl α-D-galactopyranoside on 2.0 mmol scale. The crude product was purified by column chromatography with gradient CH_2Cl_2/MeOH 25:1→10:1 as the eluent, resulting in compound **2b** as a white amorphous solid (888 mg, 99%).

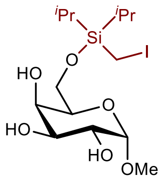

^{1}H NMR (500 MHz, $CDCl_3$) δ 4.80 (d, *J* = 3.8 Hz, 1H, H-1), 4.11 (d, *J* = 3.2 Hz, 1H, H-4), 4.01 (dd, *J* = 10.3, 6.1 Hz, 1H, H-6a), 3.96 (dd, *J* = 10.3, 5.3 Hz, 1H, H-6b), 3.87 – 3.79 (m, 2H, H-2, H-5), 3.76 (dd, *J* = 9.7, 3.2 Hz, 1H, H-3), 3.43 (s, 3H, OCH_3), 2.11 (s, 2H, CH_2I), 1.24 (quint, *J* = 7.6 Hz, 2H, C<u>H</u>(CH_3)$_2$), 1.10, 1.08, 1.07 (all s, 12H, CH(C<u>H</u>$_3$)$_2$).

^{13}C{^{1}H} NMR (126 MHz, $CDCl_3$) δ 99.5 (C-1), 71.3 (C-3), 69.9 (C-2 or C-5), 69.8 (C-2 or C-5), 69.2 (C-4), 63.3 (C-6), 55.5 (OCH_3), 17.6, 17.5, 17.4, 17.3 (CH(<u>C</u>H$_3$)$_2$), 12.2, 12.1 (<u>C</u>H(CH_3)$_2$).

***R*$_f$** = 0.48 (CH_2Cl_2/MeOH 10:1, brown color upon treatment with 5% methanolic H_3PO_4 and heating).

HRMS (ESI, m/z): calcd. for $[C_{14}H_{29}IO_6SiNa]^+$, 471.0670; found, 471.0669

Compound **2c** (methyl 6-*O*-(iodomethyl)diisopropylsilyl-β-D-glucopyranoside)

Synthesized according to **General procedure A** from methyl β-D-glucopyranoside on 2.0 mmol scale. The crude product was purified by column chromatography with gradient CH_2Cl_2/MeOH 25:1→10:1 as the eluent, resulting in compound **2c** as a white amorphous solid (605 mg, 74%).

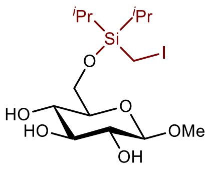

^{1}H NMR (500 MHz, $CDCl_3$) δ 4.22 (d, *J* = 7.7 Hz, 1H, H-1), 4.07 (dd, *J* = 10.5, 4.8 Hz, 1H, H-6a), 4.00 (dd, *J* = 10.5, 5.4 Hz, 1H, H-6b), 3.66 (dd~t, *J* = 9.0 Hz, 1H, H-4), 3.60 (dd~t, *J* = 9.0 Hz, H-3H), 3.54 (s, 3H, OCH_3), 3.43 (ddd~dt, *J* = 9.0, 5.1 Hz, 1H, H-5), 3.38 (dd, *J* = 9.0, 7.7 Hz, 1H, H-2), 2.12 (s, 2H, CH_2I),1.40 – 1.20 (m, 2H, C<u>H</u>(CH_3)$_2$), 1.11, 1.09, 1.08 (all s, 12H, CH(C<u>H</u>$_3$)$_2$).

^{13}C{^{1}H} NMR (126 MHz, $CDCl_3$) δ 103.4 (C-1), 76.3 (C-3), 74.9 (C-5), 73.4 (C-2), 71.6 (C-4), 64.5 (C-6), 57.1 (OCH_3), 17.63, 17.59, 17.40, 17.39 (CH(<u>C</u>H$_3$)$_2$), 12.1 (<u>C</u>H(CH_3)$_2$).

***R*$_f$** = 0.38 (CH_2Cl_2/MeOH 10:1, brown color upon treatment with 5% methanolic H_3PO_4 and heating).

HRMS (ESI, m/z): calcd. for $[C_{14}H_{29}IO_6SiNa]^+$, 471.0670; found, 471.0670

Compound **2d** (methyl 6-*O*-(iodomethyl)diisopropylsilyl-β-D-galactopyranoside)

Synthesized according to **General procedure A** from methyl β-D-galactopyranoside on 3.0 mmol scale. The crude product was purified by column chromatography with gradient CH_2Cl_2/MeOH 25:1→10:1 as the eluent, resulting in compound **2d** as a white amorphous solid (810 mg, 90%).

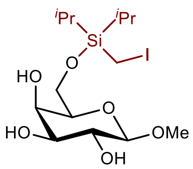

^{1}H NMR (500 MHz, $CDCl_3$) δ 4.16 (d, *J* = 7.7 Hz, 1H, H-1), 4.03 (br.s, 1H, H-4), 4.01 (dd, *J* = 10.1, 6.1 Hz, 1H, H-6a), 3.95 (dd, *J* = 10.1, 5.4 Hz, 1H, H-6b), 3.66 (dd, *J* = 9.7, 7.7 Hz, 1H, H-2), 3.59 (dd, *J* = 9.7, 3.0 Hz, 1H, H-3), 3.56 (d, *J* = 6.1, 5.4, 3.6 Hz, 1H, H-5), 3.54 (s, 3H, OCH_3), 2.11 (s, 2H, CH_2I), 1.24 (quint, *J* = 7.3 Hz, 2H, CH(CH_3)$_2$), 1.10, 1.08, 1.07 (all s, 12H, CH(CH_3)$_2$).

^{13}C{^{1}H} NMR (126 MHz, $CDCl_3$) δ 104.0 (C-1), 74.6 (C-5), 73.6 (C-3), 71.9(C-2), 68.5 (C-4), 62.6 (C-6), 57.1 (OCH_3), 17.6, 17.5, 17.4, 17.4 (CH(CH_3)$_2$), 12.1 (CH(CH_3)$_2$).

***R*$_f$** = 0.56 (CH_2Cl_2/MeOH 10:1, brown color upon treatment with 5% methanolic H_3PO_4 and heating).

HRMS (ESI, m/z): calcd. for $[C_{14}H_{29}IO_6SiNa]^+$, 471.0670; found, 471.0669

Compound **2e** (1,2:3,4-di-*O*-isopropylidene-6-*O*-(iodomethyl)diisopropylsilyl-α-D-galactopyranose)

Synthesized according to **General procedure B** from 1,2:3,4-diisopropylidene galactose[2] on 2.0 mmol scale. The crude product was purified by column chromatography with gradient hexane/EtOAc 25:1→10:1 as the eluent, resulting in compound **2e** as a colorless oil (941 mg, 92%).

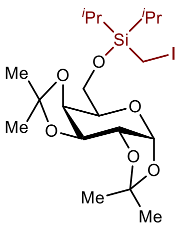

^{1}H NMR (500 MHz, $CDCl_3$) δ 5.51 (d, *J* = 5.0 Hz, 1H, H-1), 4.59 (dd, *J* = 8.0, 2.3 Hz, 1H, H-3), 4.31 (dd, *J* = 8.0, 1.4 Hz, 1H, H-4), 4.29 (dd, *J* = 5.0, 2.3 Hz, 1H, H-2), 3.95 – 3.80 (m, 3H, H-5, H-6), 2.11 (s, 2H, CH_2I), 1.54 (s, 3H), 1.43 (s, 3H), 1.33 (s, 6H, C(CH_3)$_2$), 1.30 – 1.19 (m, 2H, (CH(CH_3)$_2$), 1.10, 1.08, 1.07 (all s, 12H, CH(CH_3)$_2$).

^{13}C{^{1}H} NMR (126 MHz, $CDCl_3$) δ 109.0 (C(CH_3)$_2$), 108.5(C(CH_3)$_2$), 96.3 (C-1), 70.8 (C-3), 70.6 (C-2, C-4), 68.4 (C-5), 62.6 (C-6), 26.1, 26.0, 25.0, 24.3 (C(CH_3)$_2$), 17.62, 17.60, 17.36, 17.34 (CH(CH_3)$_2$), 12.22, 12.17 (CH(CH_3)$_2$).

***R*$_f$** = 0.28 (hexane/EtOAc 10:1, brown color upon treatment with 5% methanolic H_3PO_4 and heating).

HRMS (ESI, m/z): calcd. for $[C_{19}H_{35}IO_6SiNa]^+$, 537.1140; found, 537.1141

Compound **2f** (methyl 3,4-*O*-isopropylidene-6-*O*-(iodomethyl)diisopropylsilyl-α-D-galactopyranoside)

Synthesized according to **General procedure B** from methyl 3,4-diisopropylidene-α-D-galacto-pyranoside[3] on 0.12 mmol scale. The crude product was purified by column chromatography with gradient hexane/EtOAc 2:1→1:1 as the eluent, resulting in compound **2f** as a colorless oil (50 mg, 86%).

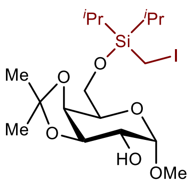

^{1}H NMR (500 MHz, $CDCl_3$) δ 4.73 (d, *J* = 3.8 Hz, 1H, H-1), 4.24 (dd, *J* = 6.0, 2.3 Hz, 1H, H-4), 4.18 (dd~t, *J* = 6.3 Hz, 1H, H-3), 4.05 (ddd~td, *J* = 6.5, 2.3 Hz, 1H, H-5), 3.97 (dd, *J* = 9.9, 6.3 Hz, 1H, H-6a), 3.92 (dd, *J* = 10.0, 6.7 Hz, 1H, H-6b), 3.77 (br.s, 1H, H-2), 3.44 (s, 3H, OCH_3), 2.36 (br.d, *J* = 5.6 Hz, 1H, OH-2), 2.10 (s, 2H, CH_2I), 1.49 (s, 3H, $C(CH_3)_2$), 1.33 (s, 3H, $C(CH_3)_2$), 1.30 – 1.18 (m, 2H, 2 × $C\underline{H}(CH_3)_2$), 1.10, 1.08, 1.07 (all s, 12H, 2 × $CH(C\underline{H}_3)_2$).

$^{13}C\{^1H\}$ NMR (126 MHz, $CDCl_3$) δ 109.4 ($\underline{C}(CH_3)_2$), 98.5 (C-1), 76.2 (C-3), 72.7 (C-4), 69.8 (C-2), 68.6 (C-5), 62.9 (C-6), 55.4 (OCH_3), 27.8 ($C(\underline{C}H_3)_2$), 26.0 ($\underline{C}(CH_3)_2$), 17.60 ($CH(\underline{C}H_3)_2$), 17.57 ($CH(\underline{C}H_3)_2$), 17.33 (2 × $CH(\underline{C}H_3)_2$), 12.19 ($\underline{C}H(CH_3)_2$), 12.12 ($\underline{C}H(CH_3)_2$).

***R*$_f$** = 0.28 (hexane/EtOAc 3:1, brown color upon treatment with 5% methanolic H_3PO_4 and heating).

HRMS (ESI, m/z): calcd. for $[C_{17}H_{33}IO_6SiNa]^+$, 511.0983; found, 511.0984

Compound **2g** (methyl 3,4-*O*-isopropylidene-6-*O*-(iodomethyl)diisopropylsilyl-β-D-galactopyranoside)

Synthesized according to **General procedure B** from methyl 3,4-diisopropylidene-β-D-galactopyranoside[4] on 1.0 mmol scale. The crude product was purified by column chromatography with gradient hexane/EtOAc 2:1→1:1 as the eluent, resulting in compound **2g** as a colorless oil (342 mg, 70%).

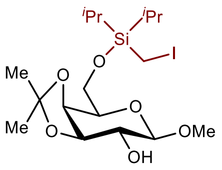

^{1}H NMR (500 MHz, $CDCl_3$) δ 4.22 (dd, *J* = 5.5, 2.2 Hz, 1H, H-4), 4.08 (d, *J* = 8.3 Hz, 1H, H-1), 4.07 – 4.05 (m, 1H, H-3), 4.04 (dd, *J* = 9.8, 7.1 Hz, 1H, H-6a), 4.00 (dd, *J* = 9.8, 6.1 Hz, 1H, H-6b), 3.86 (ddd, *J* = 7.1, 6.1, 2.2 Hz, 1H, H-5), 3.54 (s, 3H, OCH_3), 3.52 (dd, *J* = 8.3, 7.9 Hz, 1H, H-2), 2.10 (d, *J* = 1.9 Hz, 2H), 1.51 (s, 3H, $C(CH_3)_2$), 1.34 (s, 3H, $C(CH_3)_2$), 1.31 – 1.19 (m, 2H, 2 × $C\underline{H}(CH_3)_2$), 1.10, 1.09, 1.07 (all s, 12H, 2 × $CH(C\underline{H}_3)_2$).

$^{13}C\{^1H\}$ NMR (126 MHz, $CDCl_3$) δ 110.0 ($\underline{C}(CH_3)_2$), 103.3 (C-1), 78.6 (C-3), 73.9 (C-2), 73.7 (C-5), 73.1 (C-4), 62.6 (C-6), 57.0 (OCH_3), 28.2 ($C(\underline{C}H_3)_2$), 26.3 ($C(\underline{C}H_3)_2$), 17.60 ($CH(\underline{C}H_3)_2$), 17.57 ($CH(\underline{C}H_3)_2$), 17.33 (2 × $CH(\underline{C}H_3)_2$), 12.18 ($\underline{C}H(CH_3)_2$), 12.11 ($\underline{C}H(CH_3)_2$).

***R*$_f$** = 0.34 (hexane/EtOAc 2:1, brown color upon treatment with 5% methanolic H_3PO_4 and heating).

HRMS (ESI, m/z): calcd. for $[C_{17}H_{33}IO_6SiNa]^+$, 511,0983; found, 511.0981

Compound **2h** (4-methoxyphenyl 6-*O*-(iodomethyl)diisopropylsilyl-β-D-glucopyranoside)

Synthesized according to **General procedure A** from 4-methoxyphenyl β-D-glucopyranoside[5] on 1.05 mmol scale. The crude product was purified by column chromatography with gradient CH_2Cl_2/MeOH 20:1→12:1 as the eluent, resulting in compound **2h** as a white amorphous solid (488 mg, 86%).

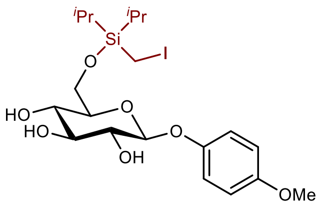

^{1}H NMR (500 MHz, $CDCl_3$) δ 7.01 (d, *J* = 9.0 Hz, 1H, C_6H_4), 6.81 (d, *J* = 9.0 Hz, 1H C_6H_4), 4.79 (d, *J* = 6.9 Hz, 1H, H-1), 4.10 – 3.98 (m, 2H, H-6), 3.77 (s, 3H, OCH_3), 3.73 – 3.61 (m, 3H, H-2, H-3, H-4), 3.51 (ddd~dt, *J* = 9.4, 5.1 Hz, 1H, H-5), 2.07 (ABq, *J* = 15.0 Hz, 2H, CH_2I), 1.23 (sept, *J* = 7.6 Hz, 2H, $C\underline{H}(CH_3)_2$), 1.09, 1.072, 1.070, 1.06, 1.05 (all s, 12H, $CH(C\underline{H}_3)_2$).

$^{13}C\{^1H\}$ NMR (126 MHz, $CDCl_3$) δ 155.2 (C, C_6H_4), 151.0 (C, C_6H_4), 118.5 (2 × CH, C_6H_4), 114.4 (2 × CH, C_6H_4), 101.5 (C-1), 76.4 (C-3), 76.0 (C-5), 73.2 (C-2 or C-4), 70.8 (C-2 or C-4), 63.9 (C-6), 55.5 (OCH_3), 17.6, 17.39, 17.37 ($CH(\underline{C}H_3)_2$), 12.05, 12.03 ($\underline{C}H(CH_3)_2$).

R_f = 0.50 (CH_2Cl_2/MeOH 8:1, brown color upon treatment with 5% methanolic H_3PO_4 and heating).

HRMS (ESI, m/z): calcd. for $[C_{20}H_{33}IO_7SiNa]^+$, 563.0932; found, 563.0935

Compound **2i** (6-*O*-(6-*O*-(iodomethyl)diisopropylsilyl-β-D-glucopyranosyl)-1,2:3,4-di-*O*-isopropylidene α-D-galactopyranose)

Synthesized according to **General procedure B** from 6-(β-D-glucopyranosyloxy) 1,2:3,4-diisopropylidene α-D-galactopyranose[6] on 0.14 mmol scale. The crude product was purified by column chromatography with hexane/EtOAc 25:1 as the eluent, resulting in compound **2i** as a colorless oil (59 mg, 62%).

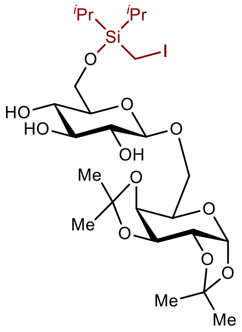

^{1}H NMR (500 MHz, $CDCl_3$) δ 5.55 (d, J = 5.0 Hz, 1H, H-1Gal), 4.61 (dd, J = 7.9, 2.4 Hz, 1H, H-3 Gal), 4.41 (d, J = 7.8 Hz, 1H, H-1Glu), 4.33 (dd, J = 5.0, 2.4 Hz, 1H, H-2 Gal), 4.22 (dd, J = 7.9, 1.8 Hz, 1H, H-4 Gal), 4.12 – 3.90 (m, 4H, H-5 Gal, 2H-6 Glu, H-6a Gal), 3.73 (dd, J = 10.9, 7.6 Hz, 1H, H-6b Gal), 3.70 – 3.56 (m, 2H, H-3 Glu, H-4Glu), 3.49 – 3.36 (m, 2H, H-2 Glu, H-5 Glu), 2.11 (s, 2H, CH_2I), 1.54 (s, 3H, $C(CH_3)_2$), 1.45 (s, 3H, $C(CH_3)_2$), 1.33 (s, 6H, 2 × $C(CH_3)_2$), 1.30 – 1.20 (m, 2H, C<u>H</u>$(CH_3)_2$), 1.07, 1.09, 1.10, (all s, 12H, CH(C<u>H</u>$_3)_2$).

^{13}C{^{1}H} NMR (126 MHz, $CDCl_3$) δ 109.6 (<u>C</u>$(CH_3)_2$), 108.9 (<u>C</u>$(CH_3)_2$), 103.6 (C-1 Glu), 96.3 (C-1 Gal), 76.3, 74.6, 73.2, 71.9, 71.1 (C-4 Gal), 70.7 (C-3 Gal), 70.4 (C-2 Gal), 69.0 (C-6 Gal), 68.0 (C-5 Gal), 64.8 (C-6 Glu), 25.99, 25.95, 24.9, 24.4 (C(<u>C</u>H$_3)_2$), 17.60, 17.57, 17.39, 17.34, 17.31 (CH(<u>C</u>H$_3)_2$), 12.13, 12.11 (<u>C</u>H$(CH_3)_2$).

R_f = 0.70 (CH_2Cl_2/MeOH 10:1, brown color upon treatment with 5% methanolic H_3PO_4 and heating).

HRMS (ESI, m/z): calcd. for $[C_{25}H_{45}IO_{11}SiNa]^+$, 699.1668; found, 699.1664

Compound **2j** ((5-bromo-4-chloro-1*H*-indol-3-yl)-6-*O*-(iodomethyl)diisopropylsilyl-β-D-glucopyranoside)

Synthesized according to **General procedure A** from X-Gal on 1.0 mmol scale. The crude product was purified by column chromatography with CH_2Cl_2/MeOH 25:1 → 10:1 as the eluent, resulting in compound **2j** as a brown amorphous solid (245 mg, 40%).

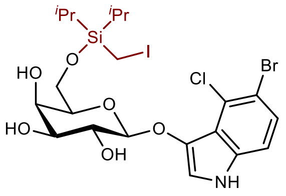

^{1}H NMR (500 MHz, MeOD) δ 7.94 (s, 1H, NH), 7.25 (d, J = 8.7 Hz, 1H, CH), 7.17 (s, 1H, CH), 7.13 (d, J = 8.7 Hz, 1H, CH), 4.74 (d, J = 7.8 Hz, 1H, H-1), 4.04 – 3.95 (m, 2H, H-6), 3.92 (d, J = 3.3 Hz, 1H, H-4), 3.88 (dd, J = 9.7, 7.8 Hz, 1H, H-2), 3.67 (td, J = 5.3, 2.6 Hz, 1H, H-5), 3.60 (dd, J = 9.7, 3.3 Hz, 1H, H-3), 2.13 – 2.02 (m, 2H, CH_2I), 1.28 – 1.09 (m, 2H, C<u>H</u>$(CH_3)_2$), 1.11 – 0.99 (m, 14H, CH(C<u>H</u>$_3)_2$).

^{13}C{^{1}H} NMR (126 MHz, MeOD) δ 138.2, 134.9, 129.8, 129.1, 126.9, 125.2, 119.5, 114.3, 113.4, 112.6, 105.8 (C-1), 77.2 (C-5), 74.9 (C-2), 72.5 (C-3), 70.2 (C-4), 64.5 (C-6), 18.10, 18.08, 17.8 (CH(<u>C</u>H$_3)_2$), 13.3 (<u>C</u>H$(CH_3)_2$).

R_f = 0.42 (CH_2Cl_2/MeOH 10:1, green color upon treatment with 5% methanolic H_3PO_4 and heating).

HRMS (ESI, m/z): calcd. for $[C_{21}H_{30}BrClINO_6SiNa]^+$, 683.9651; found, 683.9648

Note! Product **2j** is highly sensitive and should be stored at –20 °C under Ar in dark.

Compound **2k** ((((2,6-dimethoxybenzoyl)oxy)methyl)phenyl 6-*O*-(iodomethyl)diisopropylsilyl-β-D-glucopyranoside)

Synthesized according to **General procedure A** from curculigoside G[7] on 0.206 mmol scale. The crude product was purified by column chromatography with CH_2Cl_2/MeOH 50:1 → 10:1 as the eluent, resulting in compound **2k** as a colorless oil (110 mg, 76%).

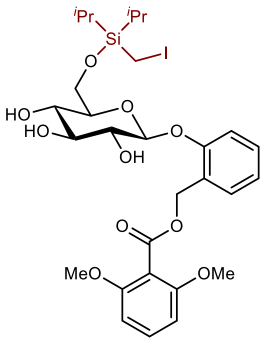

^{1}H NMR (500 MHz, $CDCl_3$) δ 7.41 (dd, *J* = 7.6, 1.7 Hz, 1H), 7.35 – 7.22 (m, 1H), 7.22 – 7.12 (m, 2H), 7.04 (t, *J* = 7.5 Hz, 1H), 6.53 (d, *J* = 8.4 Hz, 2H), 5.50 (d, *J* = 11.8 Hz, 1H), 5.44 (d, *J* = 11.8 Hz, 1H), 4.86 (d, *J* = 7.4 Hz, 1H, H-1), 4.12 (dd, *J* = 10.7, 4.1 Hz, 1H, H-6a), 4.04 (dd, *J* = 10.7, 5.6 Hz, 1H, H-6b), 2.96 (s, 3H, OCH_3), 2.89 (s, 3H, OCH_3), 3.72 – 3.65 (m, 3H), 3.62 (ddd, *J* = 9.5, 8.5, 3.9 Hz, 1H), 1.26 (quint, *J* = 7.4 Hz, 2H), 1.15 – 0.93 (m, 14H).

***R*$_f$** = 0.60 (CH_2Cl_2/MeOH 10:1, red color upon treatment with 5% methanolic H_3PO_4 and heating).

HRMS (ESI, m/z): calcd. for [C29H41O10SiNa]$^+$, 727.1406; found, 727.1406

Compound **2l** (2,3:4,5-di-*O*-isopropylidene-1-*O*-(iodomethyl)diisopropylsilyl-α-D-fructopyranose)

Synthesized according to **General procedure B** from 2,3:4,5-diisopropylidene-β-D-fructopyranose[8] on 1.0 mmol scale. The crude product was purified by column chromatography with hexane/EtOAc 10:1 → 8:1 as the eluent, resulting in compound **2l** as a colorless oil (488 mg, 95%).

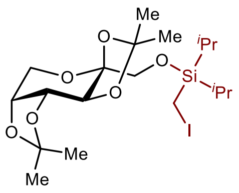

^{1}H NMR (500 MHz, $CDCl_3$) δ 4.61 (dd, *J* = 7.9, 2.6 Hz, 1H, H-4), 4.48 (d, *J* = 2.6 Hz, 1H, H-3), 4.24 (ddd, *J* = 7.9, 1.8, 0.8 Hz, 1H, H-5), 3.93 (dd, *J* = 12.9, 1.8 Hz, 1H, H-5a), 3.87 (d, *J* = 10.4 Hz, 1H, H-1a), 3.79 (d, *J* = 10.4 Hz, 1H, H-1b), 3.73 (dd, *J* = 12.9, 0.8 Hz, 1H, H-6b), 2.09 (s, 2H, CH_2I), 1.54 (s, 3H), 1.46 (s, 3H), 1.43 (s, 3H), 1.34 (s, 3H, all $C(CH_3)_2$), 1.32 – 1.21 (m, 2H, C<u>H</u>$(CH_3)_2$), 1.16 – 1.02 (m, 12H, CH(C<u>H</u>$_3$)$_2$).

^{13}C{^{1}H} NMR (126 MHz, $CDCl_3$) δ 108.8 (<u>C</u>$(CH_3)_2$), 108.4(<u>C</u>$(CH_3)_2$), 103.0 (C-2), 71.1 (C-5), 70.2 (C-4), 69.1 (C-3), 64.7 (C-1), 61.0 (C-6), 26.7, 25.87, 25.86, 23.9 (C(<u>C</u>H$_3$)$_2$), 17.65, 17.60, 17.34, 17.30 (CH(<u>C</u>H$_3$)$_2$), 12.4, 12.1 (<u>C</u>H$(CH_3)_2$).

***R*$_f$** = 0.53 (hexane/EtOAc 5:1, brown color upon treatment with 5% methanolic H_3PO_4 and heating).

HRMS (ESI, m/z): calcd. for $[C_{19}H_{35}IO_6SiNa]^+$, 537.1140; found, 537.1138

Compound **2m** (1,2-*O*-isopropylidene-5-*O*-(iodomethyl)diisopropylsilyl-α-D-xylofuranose)

Synthesized according to **General procedure B** from 1,2-isopropylidene-β-D-xylofuranose[9] on 0.81 mmol scale. The crude product was purified by column chromatography with hexane/EtOAc 7:1 → 1:1 as the eluent, resulting in compound **2m** as a white amorphous solid (297 mg, 83%).

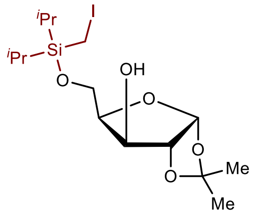

^{1}H NMR (500 MHz, $CDCl_3$) δ 5.96 (d, *J* = 3.6 Hz, 1H, H-1), 4.52 (d, *J* = 3.6 Hz, 1H, H-2), 4.35 (d, *J* = 2.6 Hz, 1H, H-3), 4.26 – 4.20 (m, 2H, H-5), 4.19 – 4.14 (m, 1H, H-4), 3.79 (br.s, 1H, OH), 2.11 (s, 2H, CH_2I), 1.49 (s, 3H), 1.32 (s, 3H), 1.25 (quint, *J* = 7.3 Hz, 2H, C<u>H</u>$(CH_3)_2$), 1.11 – 1.06 (m, 12H, CH(C<u>H</u>$_3$)$_2$).

^{13}C NMR (126 MHz, $CDCl_3$) δ 111.6 (C(CH_3)$_2$), 105.0 (C-1), 85.4 (C-2), 78.4 (C-4), 76.7 (C-3), 62.6 (C-5), 26.8 (C(CH_3)$_2$), 26.2 (C(CH_3)$_2$), 17.58, 17.40, 17.34, 17.24 (CH(CH_3)$_2$), 12.05, 11.96 (CH(CH_3)$_2$).

***R*$_f$** = 0.54 (hexane/EtOAc 2:1, brown color upon treatment with 5% methanolic H_3PO_4 and heating).

HRMS (ESI, m/z): calcd. for $[C_{15}H_{29}IO_5SiNa]^+$, 467.0721; found, 467.0721

Compound **SI-2** (cholesteryl acrylate)

Synthesized according to **General procedure C** from cholesterol on 0.18 mmol scale. The crude product was purified by column chromatography with hexane/EtOAc 10:1 → 5:1 as the eluent, resulting in compound **SI-2** as a white solid (47 mg, 60%).

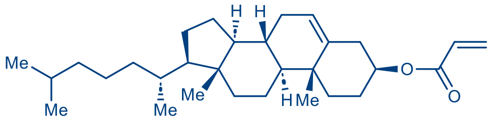

^{1}H NMR (500 MHz, $CDCl_3$) δ 6.39 (dd, *J* = 17.3, 1.5 Hz, 1H, CH_2=CH), 6.10 (dd, *J* = 17.3, 10.4 Hz, 1H, CH_2=CH), 5.80 (dd, *J* = 10.4, 1.5 Hz, 1H, CH_2=CH), 5.39 (d, *J* = 4.7 Hz, 1H), 4.69 (dddd, *J* = 11.4, 9.0, 7.1, 4.3 Hz, 1H), 2.07 – 1.93 (m, 2H), 1.93 – 1.78 (m, 3H), 1.68 – 1.40 (m, 7H), 1.40 – 1.21 (m, 5H), 1.21 – 1.04 (m, 6H), 1.03 (s, 3H, CH_3), 1.02 – 0.93 (m, 3H), 0.92 (d, *J* = 6.6 Hz, 3H, CH_3), 0.87 (d, *J* = 6.6 Hz, 3H, CH_3), 0.86 (d, *J* = 6.6 Hz, 3H, CH_3), 0.68 (s, 3H, CH_3).

^{13}C{^{1}H} NMR (126 MHz, $CDCl_3$) δ 165.6 (C=O), 139.6 (CH_2=CH), 130.2 (CH_2=CH), 129.0 (CH_2=CH), 122.7 (CH=C), 74.1, 56.7, 56.1, 50.0, 42.3, 39.7, 39.5, 38.1, 37.0, 36.6, 36.2, 35.8, 31.91, 31.87, 28.23, 28.01, 27.77, 24.3, 23.8, 22.8, 22.6, 21.0, 19.3, 18.7, 11.9.

***R*$_f$** = 0.62 (hexane/EtOAc 2:1, red color upon treatment with 5% methanolic H_3PO_4 and heating).

The spectroscopic data are in agreement with the literature.[10]

Compound **SI-3** (6-*O*-acryloyl-1,2:3,4-di-*O*-isopropylidene α-D-galactopyranose)

Synthesized according to **General procedure C** from 1,2:3,4-diisopropylidene α-D-galactopyranose[2] on 1.0 mmol scale. The crude product was purified by column chromatography with hexane/EtOAc 5:1 → 3:1 as the eluent, resulting in compound **SI-3** as a colorless oil (205 mg, 65%).

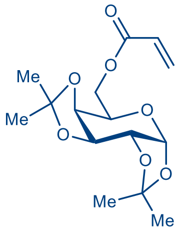

^{1}H NMR (500 MHz, $CDCl_3$) δ 6.41 (dd, *J* = 17.3, 1.4 Hz, 1H, CH_2CH), 6.15 (dd, *J* = 17.4, 10.4 Hz, 1H, CH_2CH), 5.82 (dd, *J* = 10.4, 1.4 Hz, 1H, CH_2CH), 5.53 (d, *J* = 5.0 Hz, 1H, H-1), 4.61 (dd, *J* = 7.9, 2.5 Hz, 1H, H-3), 4.36 (dd, *J* = 11.6, 4.7 Hz, 1H, H-6a), 4.31 (dd, *J* = 5.0, 2.5 Hz, 1H, H-2), 4.28 – 4.21 (m, 2H, H-4, H-6b), 4.05 (ddd, *J* = 7.2, 4.7, 1.9 Hz, 1H, H-5), 1.49 (s, 3H), 1.44 (s, 3H), 1.33 (s, 3H), 1.31 (s, 3H, C(CH_3)$_2$).

^{13}C{^{1}H} NMR (126 MHz, $CDCl_3$) δ 166.0 (C=O), 131.0 (CH_2CH), 128.2 (CH_2CH), 109.6 (C(CH_3)$_2$), 108.7 (C(CH_3)$_2$), 96.3 (C-1), 71.0 (C-4), 70.7 (C-3), 70.4 (C-2), 66.0 (C-5), 63.5 (C-6), 25.95, 25.92, 24.9, 24.4 (C(CH_3)$_2$).

***R*$_f$** = 0.78 (hexane/EtOAc 2:1, brown color upon treatment with 5% methanolic H_3PO_4 and heating).

HRMS (ESI, m/z): calcd. for $[C_{15}H_{22}O_7Na]^+$, 337.1258; found, 337.1259

Compound **SI-4** (3-*O*-acryloyl-1,2:5,6-di-*O*-isopropylidene α-D-glucofuranose)

Synthesized according to **General procedure C** from 1,2:4,6-diisopropylidene α-D-glucofuranose on 1.0 mmol scale. The crude product was purified by column chromatography with hexane/EtOAc 5:1 → 3:1 as the eluent, resulting in compound **SI-4** as a colorless oil (314 mg, 99%).

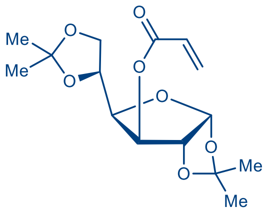

^{1}H NMR (500 MHz, $CDCl_3$) δ 6.43 (dd, *J* = 17.3, 1.2 Hz, 1H, C<u>H</u>$_2$=CH), 6.11 (dd, *J* = 17.3, 10.5 Hz, 1H, CH_2=C<u>H</u>), 5.88 (dd, *J* = 10.5, 1.2 Hz, 1H, CH_2=CH), 5.88 (d, *J* = 3.7 Hz, 1H, H-1), 5.31 (d, *J* = 2.3 Hz, 1H), 4.51 (d, *J* = 3.7 Hz, 1H, H-2), 4.26 – 4.19 (m, 2H), 4.05 (dd, *J* = 8.5, 5.5 Hz, 1H, H-6a), 4.01 (dd, *J* = 8.5, 4.7 Hz, 1H, H-6b), 1.51 (s, 3H, CH_3), 1.39 (s, 3H, CH_3), 1.29 (s, 3H, CH_3), 1.28 (s, 3H, CH_3).

^{13}C{^{1}H} NMR (126 MHz, $CDCl_3$) δ 164.6 (C=O), 131.9 (<u>C</u>H$_2$=CH), 127.7 (CH_2=<u>C</u>H), 112.2 (<u>C</u>(CH_3)$_2$), 109.2 (<u>C</u>(CH_3)$_2$), 105.0 (C-1), 83.2 (C-2), 79.7, 76.1, 72.4, 67.1 (C-6), 26.8 (C(<u>C</u>H$_3$)$_2$), 26.7 (C(<u>C</u>H$_3$)$_2$), 26.2 (C(<u>C</u>H$_3$)$_2$), 25.2 (C(<u>C</u>H$_3$)$_2$).

***R*$_f$** = 0.76 (hexane/EtOAc 2:1, brown color upon treatment with 5% methanolic H_3PO_4 and heating).

HRMS (ESI, m/z): calcd. for $[C_{15}H_{22}O_7Na]^+$, 337.1258; found, 337.1263

3. Optimization of the reaction conditions

Optimization of the reaction conditions was performed with several carbohydrate substrates according to the following procedure. The carbohydrate substrate **2** (0.05 mmol), photocatalyst, and other solid reagents were placed in a dry 8 mL screw neck vial (VWR, Art. No. MANA702096) equipped with a magnetic stirring bar (VWR, Art. No. 442-0401) and a septum (VWR, Art. No. 217-0183). The mixture was evacuated and backfilled with nitrogen three times, followed by addition of deaerated solvent, methyl acrylate somophile (1.2 equiv., unless otherwise noted), and other liquid reagents. For the reactions featuring insoluble or partially soluble reagents, the reaction mixture was additionally sonicated for ca. 1 min prior irradiation. The closed reaction vial was disconnected from the nitrogen line and placed into a 3D-printed polypropylene holder ca. 2 cm from the LED light source (440 nm, 40 W Kessil PR160L). The reaction mixture was stirred at 1200 rpm under irradiation at room temperature with fan cooling (Figure S1). The reaction progress was monitored by ^{1}H NMR with an internal standard: an aliquot was taken from the reaction mixture after indicated time, filtered through a short silica plug eluting with EtOAc, concentrated on rotary evaporator, and dissolved in a stock solution of the internal standard (1,3,5-trimethoxybenzene) in $CDCl_3$, followed by ^{1}H NMR analysis.

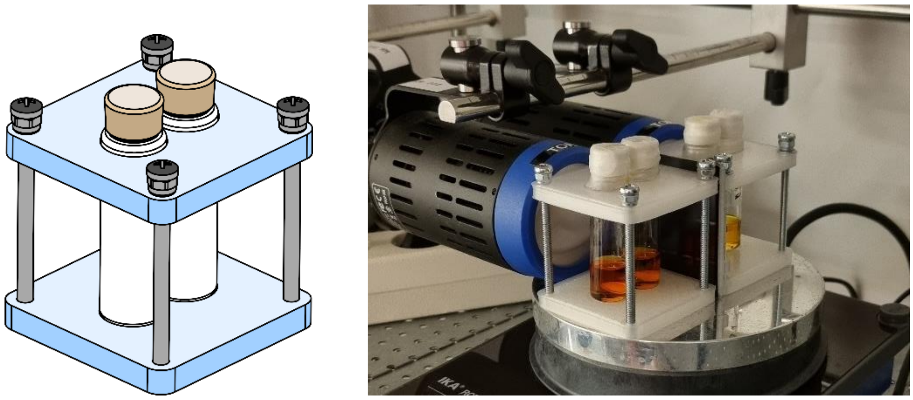

Figure S1. Scheme of the 3D-printed vial holder used for the photochemical reactions and the representative reaction setup. The cooling fan (not shown) is placed in front of the LED lamps and the reaction vials.

Tables S1 and S2 exemplify selected entries from the optimization studies for acetonide-protected substrate **2e** and fully unprotected substrate **2d**. For substrate **2e**, the conditions from entry 1 (Table S1) were initially selected as optimal, while only marginal improvements could be observed when screening the reaction solvent, XAT agent, photocatalyst (Figure S2), base, and other parameters. Notably, the yield of the desired product **3c** and the rate of the reaction decreased significantly without the addition of a base (entry 45, Table S1), and this reaction component was initially deemed necessary. Alternative mechanistic strategies based on the use of bis(catecholato)borate[11] and formate salts[12] as sacrificial reductants were also assessed, demonstrating significantly lower yields of the desired product for the former (entry 76, Table S1) and marginally lower yields for the latter approach (entry 84, Table S1).

Applying the optimal conditions identified for the protected substrate **2e** (entry 1, Table S1) to the unprotected substrate **2d** (entry 1, Table S2) resulted in significantly lower yields of the desired C-functionalized diastereomeric products **3b** and **3b’** (entry 1, Table S2). Unfortunately, the ^{1}H NMR yields of the individual gluco/galacto diastereomers, as well as the yield of the dehalogenated side-product **4d**, could not always be reliably determined by ^{1}H NMR of the reaction mixture due to overlapping NMR signals. Both the low UV absorbance and the low volatility of the starting materials and products

precluded the use of other standard analytical techniques, such as HPLC and GC. For substrate **2d**, a significant increase in yield of the desired products was observed when changing the solvent to EtOAc (entry 2, Table S2). Some of the other variations in the reaction conditions also led to increased yields (e.g., entries 15, 22, and 25, Table S2), while using carboxylate[13] and borinic acid[14] additives proved ineffective (entries 37–41, Table S2), and using supersilanol as the XAT agent[15] resulted in formation of a considerable amount of side-products (entries 33–36, Table S2). Notably, decreasing the amount of heterogeneous base proved detrimental to the reaction (*cf.* entries 22, 25, and 27, Table S2). Any attempts at conducting the reaction at larger scale (0.2 mmol of substrate **2d**) failed to deliver the desired products in reasonable yields. A strong dependence of the yield on the particle size of the

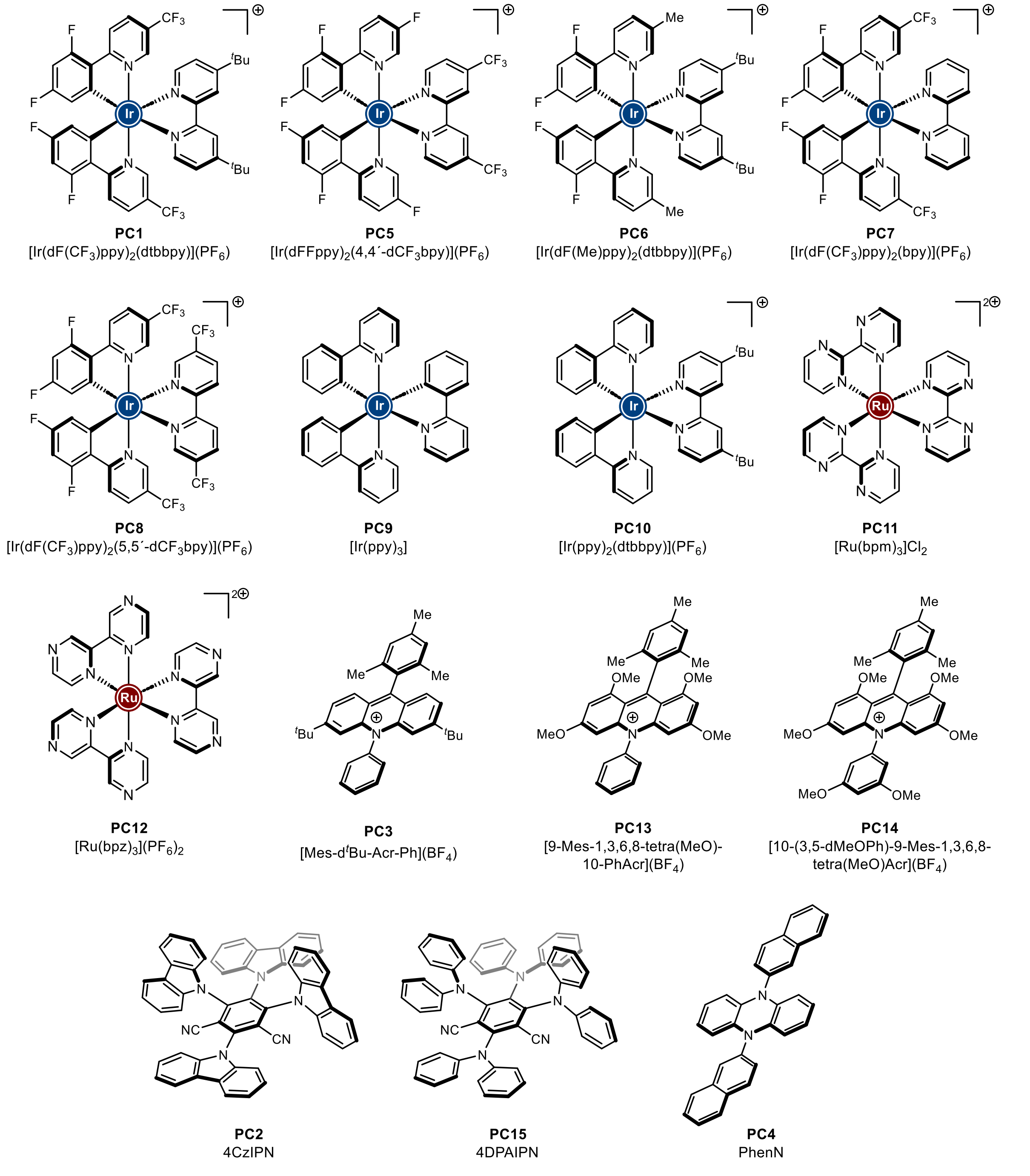

Figure S2. Photoredox catalysts **PC1–15** used for optimization of the reaction conditions.

employed heterogeneous base was also observed; thereby, the related reaction conditions were deemed unsuitable for further optimization. Gratifyingly, employing 1,2-dimethylimidazole or 1,4-diazabicyclo[2.2.2]octane (DABCO) as the base provided homogeneous reaction mixtures delivering the desired products in good yields (entries 43 and 50, Table S2). Further adjustment of the solvent composition, the amount of XAT agent, and the ratio of substrate **2d** and methyl acrylate somophile allowed formation of the desired products **3b** and **3b'** in preparative yields without addition of a base (entry 68, Table S2). These reaction conditions were selected as the final and were used for investigation of the generality of the devised transformation. The key variations from the standard reaction conditions are highlighted for substrate **2a** (Figure 2), which provided the most conclusive ^{1}H NMR spectra of the crude reaction mixtures.

Table S1. Selected entries from the optimization of the reaction conditions for the photoredox-mediated C-functionalization reaction with substrate **2e**.

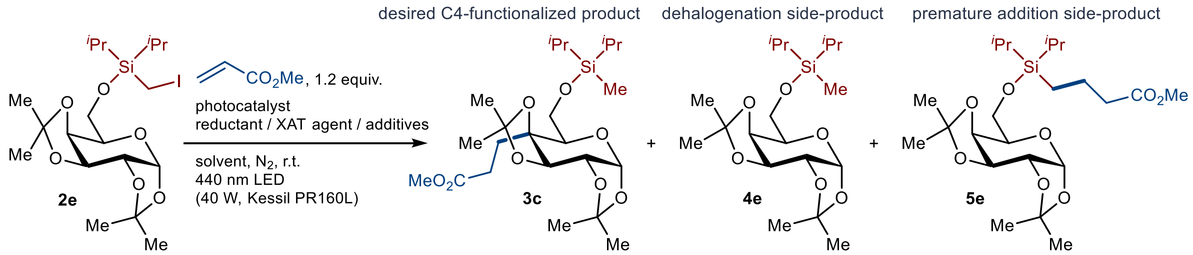

№	photocatalyst	2e concentration & solvent	reductant / XAT agent / additives / other conditions	time	NMR yields (%)[b]			
					2e	3c	4e	5e
			solvent screening					
1	**PC1**, 3 mol%	0.01 M $PhCF_3$[a]	$^iPr_2NEt$, 1.2 equiv., Cs_2CO_3, 2 equiv.	2 h	10	**37**	23	20
				5 h	0	**39**	24	25
2	**PC1**, 3 mol%	0.01 M PhMe[a]	$^iPr_2NEt$, 1.2 equiv., Cs_2CO_3, 2 equiv.	2 h	50	**7**	29	10
				5 h	22	**15**	45	13
3	**PC1**, 3 mol%	0.01 M DMF[a]	$^iPr_2NEt$, 1.2 equiv., Cs_2CO_3, 2 equiv.	2 h	0	**13**	47	14
4	**PC1**, 3 mol%	0.01 M DMSO[a]	$^iPr_2NEt$, 1.2 equiv., Cs_2CO_3, 2 equiv.	2 h	0	**17**	20	8
5	**PC1**, 3 mol%	0.01 M MeCN[a]	$^iPr_2NEt$, 1.2 equiv., Cs_2CO_3, 2 equiv.	2 h	13	**23**	24	23
				5 h	0	**29**	31	25
6	**PC1**, 3 mol%	0.01 M ^{t}BuCN	$^iPr_2NEt$, 1.2 equiv., Cs_2CO_3, 2 equiv.	2 h	8	**29**	19	14
				5 h	0	**39**	3	36
7	**PC1**, 3 mol%	0.01 M CH_2Cl_2[a]	$^iPr_2NEt$, 1.2 equiv., Cs_2CO_3, 2 equiv.	2 h	52	**0**	44	0
8	**PC1**, 3 mol%	0.01 M PhF	$^iPr_2NEt$, 1.2 equiv., Cs_2CO_3, 2 equiv.	2 h	31	**30**	9	18
				5 h	0	**42**	12	28
9	**PC1**, 3 mol%	0.01 M PhF[a]	$^iPr_2NEt$, 1.2 equiv., Cs_2CO_3, 2 equiv.	2 h	29	**30**	10	18
				5 h	0	**42**	24	24
10	**PC1**, 3 mol%	0.01 M DME[a]	$^iPr_2NEt$, 1.2 equiv., Cs_2CO_3, 2 equiv.	2 h	44	**11**	30	8
11	**PC1**, 3 mol%	0.01 M EtOAc[a]	$^iPr_2NEt$, 1.2 equiv., Cs_2CO_3, 2 equiv.	2 h	12	**32**	0	23
				5 h	0	**38**	28	23
12	**PC1**, 3 mol%	0.01 M EtOAc	$^iPr_2NEt$, 1.2 equiv., Cs_2CO_3, 2 equiv.	2 h	33	**22**	17	22

№	photocatalyst	2e concentration & solvent	reductant / XAT agent / additives / other conditions	time	NMR yields (%)[b]			
					2e	3c	4e	5e
13	**PC1**, 3 mol%	0.01 M EtOAc/CF_3CH_2OH 9:1	$^iPr_2NEt$, 1.2 equiv., Cs_2CO_3, 1 equiv.	2 h	0	**38**	n.d.	34
14	**PC1**, 3 mol%	0.01 M EtOAc/$(CF_3)_2CHOH$ 9:1	$^iPr_2NEt$, 1.2 equiv., Cs_2CO_3, 1 equiv.	2 h	0	**30**	n.d.	18
15	**PC1**, 3 mol%	0.01 M MeOH	$^iPr_2NEt$, 1.2 equiv., Cs_2CO_3, 2 equiv.	2 h	0	**28**	29	37
16	**PC1**, 3 mol%	0.01 M Me_2CO	$^iPr_2NEt$, 1.2 equiv., Cs_2CO_3, 2 equiv.	2 h	66	**7**	7	12
				5 h	56	**9**	4	17
17	**PC1**, 3 mol%	0.01 M $PhCF_3$/ CF_3CH_2OH 10:1	$^iPr_2NEt$, 1.2 equiv., Cs_2CO_3, 2 equiv.	2 h	11	**34**	13	38
18	**PC1**, 3 mol%	0.01 M $PhCF_3/(CF_3)_2CHOH$ 10:1	$^iPr_2NEt$, 1.2 equiv., Cs_2CO_3, 2 equiv.	2 h	0	**14**	55	16
19	**PC1**, 3 mol%	0.01 M Ph^tBu	$^iPr_2NEt$, 1.2 equiv., Cs_2CO_3, 2 equiv.	2 h	29	**9**	56	9
20	**PC1**, 3 mol%	0.01 M PhCN	$^iPr_2NEt$, 1.2 equiv., Cs_2CO_3, 2 equiv.	2 h	0	**27**	7	39
21	**PC1**, 3 mol%	0.01 M ^iPrOAc	$^iPr_2NEt$, 1.2 equiv., Cs_2CO_3, 1 equiv.	2 h	n.d.	**31**	30	30
			XAT agent screening					
22	**PC1**, 3 mol%	0.01 M $PhCF_3$[a]	$^iPr_2NEt$, 0.6 equiv., Cs_2CO_3, 2 equiv.	2 h	44	**23**	6	17
				5 h	33	**26**	5	22
23	**PC1**, 3 mol%	0.01 M $PhCF_3$[a]	$^iPr_2NEt$, 2 equiv., Cs_2CO_3, 2 equiv.	2 h	9	**43**	18	21
				5 h	0	**40**	7	35
24	**PC1**, 3 mol%	0.01 M $PhCF_3$[a]	$^iBu_3N$, 1.2 equiv., Cs_2CO_3, 2 equiv.	2 h	11	**33**	21	18
				5 h	0	**40**	25	20
25	**PC1**, 3 mol%	0.01 M $PhCF_3$[a]	Bu_3N, 1.2 equiv., Cs_2CO_3, 2 equiv.	2 h	0	**40**	19	28
				5 h	0	**42**	16	32
26	**PC1**, 3 mol%	0.01 M $PhCF_3$[a]	Et_3N, 1.2 equiv., Cs_2CO_3, 2 equiv.	2 h	19	**31**	11	26
				5 h	0	**39**	19	31
27	**PC1**, 3 mol%	0.01 M $PhCF_3$[a]	Cy_2NMe, 1.2 equiv., Cs_2CO_3, 2 equiv.	2 h	28	**26**	24	12
28	**PC1**, 3 mol%	0.01 M $PhCF_3$[a]	1,2,2,6,6-pentamethylpiperidine, 1.2 equiv., Cs_2CO_3, 2 equiv.	2 h	0	**39**	25	20
29	**PC1**, 3 mol%	0.01 M $PhCF_3$[a]	2,2,6,6-tetramethylpiperidine, 1.2 equiv., Cs_2CO_3, 2 equiv.	2 h	96	**0**	0	0
30	**PC1**, 3 mol%	0.01 M $PhCF_3$[a]	$BnNMe_2$, 1.2 equiv., Cs_2CO_3, 2 equiv.	2 h	23	**25**	21	15
			photocatalyst screening					
31	**PC1**, 1 mol%	0.01 M $PhCF_3$[a]	$^iPr_2NEt$, 1.2 equiv., Cs_2CO_3, 2 equiv.	2 h	33	**24**	18	16
32	**PC5**, 3 mol%	0.01 M $PhCF_3$[a]	$^iPr_2NEt$, 1.2 equiv., Cs_2CO_3, 2 equiv.	2 h	48	**21**	7	15
				5 h	21	**32**	7	21
33	**PC5**, 3 mol%	0.01 M $PhCF_3$[a]	NBu_3, 1.2 equiv., Cs_2CO_3, 2 equiv.	2 h	44	**22**	5	17
				5 h	10	**36**	10	24

№	photocatalyst	2e concentration & solvent	reductant / XAT agent / additives / other conditions	time	NMR yields (%)[b]			
					2e	3c	4e	5e
34	**PC5**, 3 mol%	0.01 M EtOAc	NBu_3, 1.2 equiv., Cs_2CO_3, 2 equiv.	2 h	88	**0**	3	0
35	**PC6**, 3 mol%	0.01 M $PhCF_3$[a]	$^iPr_2NEt$, 1.2 equiv., Cs_2CO_3, 2 equiv.	2 h	13	**35**	12	25
				5 h	0	**42**	19	26
36	**PC7**, 3 mol%	0.01 M $PhCF_3$[a]	$^iPr_2NEt$, 1.2 equiv., Cs_2CO_3, 2 equiv.	2 h	25	**28**	22	11
				5 h	4	**29**	38	16
37	**PC8**, 3 mol%	0.01 M $PhCF_3$[a]	$^iPr_2NEt$, 1.2 equiv., Cs_2CO_3, 2 equiv.	2 h	85	**0**	12	0
38	**PC2**, 3 mol%	0.01 M $PhCF_3$[a]	$^iPr_2NEt$, 1.2 equiv., Cs_2CO_3, 2 equiv.	2 h	0	**37**	34	19
39	**PC2**, 3 mol%	0.01 M EtOAc	$^iPr_2NEt$, 1.2 equiv., Cs_2CO_3, 2 equiv.	2 h	0	**23**	59	0
40	**PC3**, 3 mol%	0.01 M $PhCF_3$[a]	$^iPr_2NEt$, 1.2 equiv., Cs_2CO_3, 2 equiv.	2 h	40	**9**	17	0
41	**PC14**, 3 mol%	0.01 M $PhCF_3$[a]	$^iPr_2NEt$, 1.2 equiv., Cs_2CO_3, 2 equiv.	2 h	81	**0**	19	0
42	**PC13**, 3 mol%	0.01 M $PhCF_3$[a]	$^iPr_2NEt$, 1.2 equiv., Cs_2CO_3, 2 equiv.	2 h	85	**0**	13	0
43	**PC11**, 3 mol%	0.01 M $PhCF_3$[a]	$^iPr_2NEt$, 1.2 equiv., Cs_2CO_3, 2 equiv.	2 h	100	**0**	0	0
44	**PC12**, 3 mol%	0.01 M $PhCF_3$[a]	$^iPr_2NEt$, 1.2 equiv., Cs_2CO_3, 2 equiv.	2 h	100	**0**	0	0
			base screening					
45	**PC1**, 3 mol%	0.01 M $PhCF_3$[a]	$^iPr_2NEt$, 1.2 equiv.	2 h	45	**19**	11	19
				5 h	30	**26**	13	23
46	**PC1**, 3 mol%	0.01 M $PhCF_3$[a]	$^iPr_2NEt$, 1.2 equiv., 1,5,7-triazabicyclo [4.4.0]dec-5-ene (TBD), 0.2 equiv.	2 h	44	**24**	0	22
				5 h	28	**31**	8	23
47	**PC1**, 3 mol%	0.01 M $PhCF_3$[a]	$^iPr_2NEt$, 1.2 equiv., 2,4,6-collidine, 2 equiv.	2 h	40	**23**	8	21
				5 h	35	**27**	14	22
48	**PC1**, 3 mol%	0.01 M $PhCF_3$[a]	$^iPr_2NEt$, 1.2 equiv., imidazole, 2 equiv.	2 h	10	**32**	19	24
49	**PC1**, 3 mol%	0.01 M $PhCF_3$[a]	$^iPr_2NEt$, 1.2 equiv., $Bu_4NH_2PO_4$, 2 equiv.	2 h	23	**33**	1	28
				5 h	0	**37**	16	27
50	**PC1**, 3 mol%	0.01 M EtOAc	$^iPr_2NEt$, 1.2 equiv., $Bu_4NH_2PO_4$, 2 equiv.	2 h	29	**28**	18	17
51	**PC1**, 3 mol%	0.01 M $PhCF_3$[a]	$^iPr_2NEt$, 1.2 equiv., $(Bu_4N)_2HPO_4$, 2 equiv.	2 h	0	**0**	82	0
52	**PC1**, 3 mol%	0.01 M EtOAc	$^iPr_2NEt$, 1.2 equiv., $(Bu_4N)_2HPO_4$, 2 equiv.	2 h	0	**0**	65	0
53	**PC1**, 3 mol%	0.01 M $PhCF_3$[a]	$^iPr_2NEt$, 1.2 equiv., K_3PO_4, 2 equiv.	2 h	25	**32**	16	20
				5 h	29	**32**	18	18
54	**PC1**, 3 mol%	0.01 M $PhCF_3$[a]	$^iPr_2NEt$, 1.2 equiv., Na_2HPO_4, 2 equiv.	2 h	42	**26**	9	18
				5 h	6	**40**	23	24
55	**PC1**, 3 mol%	0.01 M $PhCF_3$[a]	$^iPr_2NEt$, 1.2 equiv., Li_2CO_3, 2 equiv.	2 h	38	**23**	16	17
				5 h	30	**26**	11	19
56	**PC1**, 3 mol%	0.01 M $PhCF_3$[a]	$^iPr_2NEt$, 1.2 equiv., K_2CO_3, 1 equiv.	2 h	69	**11**	1	14
				5 h	57	**18**	0	18
57	**PC1**, 3 mol%	0.01 M $PhCF_3$[a]	$^iPr_2NEt$, 1.2 equiv., $(Bu_4N)(BuO)_2HPO_2$, 2 equiv.	2 h	0	**39**	21	20

№	photocatalyst	2e concentration & solvent	reductant / XAT agent / additives / other conditions	time	NMR yields (%)[b]			
					2e	3c	4e	5e
			other parameters					
58	**PC1**, 3 mol% (2 x LEDs)	0.01 M $PhCF_3$[a]	$^{i}Pr_2NEt$, 1.2 equiv., Cs_2CO_3, 2 equiv.	2 h	0	**41**	27	19
59	**PC1**, 3 mol%	0.005 M $PhCF_3$[a]	$^{i}Pr_2NEt$, 1.2 equiv., Cs_2CO_3, 2 equiv.	2 h	10	**23**	34	24
				5 h	2	**23**	40	25
60	**PC1**, 3 mol%	0.02 M $PhCF_3$[a]	$^{i}Pr_2NEt$, 1.2 equiv., Cs_2CO_3, 2 equiv.	2 h	30	**21**	6	32
				5 h	5	**32**	0	51
61	**no PC**, 390 nm LED	0.01 M $PhCF_3$[a]	$^{i}Pr_2NEt$, 1.2 equiv., Cs_2CO_3, 2 equiv.	2 h	100	**0**	0	0
62	**PC1**, 3 mol%	0.01 M $PhCF_3$[a]	$^{i}Pr_2NEt$, 1.2 equiv., Cs_2CO_3, 2 equiv., 16 °C	2 h	57	**15**	0	25
				5 h	36	**26**	0	35
63	**PC1**, 3 mol%	0.01 M $PhCF_3$[a]	$^{i}Pr_2NEt$, 1.2 equiv., Cs_2CO_3, 2 equiv., 65 °C	2 h	39	**31**	6	17
				5 h	27	**37**	5	20
64	**PC1**, 3 mol%	0.01 M $PhCF_3$[a]	$^{i}Pr_2NEt$, 1.2 equiv., Cs_2CO_3, 2 equiv., 1.5 equiv. of methyl acrylate	2 h	28	**29**	9	21
65	**PC1**, 3 mol%	0.01 M $PhCF_3$[a]	$^{i}Pr_2NEt$, 2 equiv., Cs_2CO_3, 2 equiv., 1.5 equiv. of methyl acrylate	2 h	45	**23**	3	21
66	**PC1**, 3 mol%	0.01 M $PhCF_3$[a]	$^{i}Pr_2NEt$, 1.2 equiv., Cs_2CO_3, 2 equiv., 2 equiv. of methyl acrylate	2 h	30	**26**	11	24
			bis-catechol borate as the sacrificial reductant					
67	**PC9**, 3 mol%	0.01 M $PhCF_3$[a]	$Bu_4NB(cat)_2$, 2 equiv., Cs_2CO_3, 2 equiv.	2 h	37	**0**	11	0
68	**PC10**, 3 mol%	0.01 M $PhCF_3$[a]	$Bu_4NB(cat)_2$, 2 equiv., Cs_2CO_3, 2 equiv.	2 h	48	**0**	2	0
69	**PC6**, 3 mol%	0.01 M $PhCF_3$[a]	$Bu_4NB(cat)_2$, 2 equiv., Cs_2CO_3, 2 equiv.	2 h	48	**0**	4	0
70	**PC9**, 3 mol%	0.01 M DMF[a]	$Bu_4NB(cat)_2$, 2 equiv., Cs_2CO_3, 2 equiv.	2 h	0	**0**	67	0
71	**PC10**, 3 mol%	0.01 M $PhCF_3$[a]	$Bu_4NB(cat)_2$, 2 equiv., Cs_2CO_3, 2 equiv.	2 h	15	**9**	74	0
72	**PC10**, 3 mol%	0.01 M EtOAc	$Bu_4NB(cat)_2$, 2 equiv., Cs_2CO_3, 2 equiv.	2 h	91	**0**	9	0
73	**PC1**, 3 mol%	0.01 M EtOAc	$LiB(cat)_2$, 2 equiv., Cs_2CO_3, 2 equiv.	2 h	86	**0**	5	0
74	**PC14**, 5 mol%	0.01 M EtOAc	$LiB(cat)_2$, 2 equiv., Cs_2CO_3, 2 equiv.	2 h	99	**0**	1	0
75	**PC3**, 5 mol%	0.01 M EtOAc	$LiB(cat)_2$, 2 equiv., Cs_2CO_3, 2 equiv.	2 h	99	**0**	1	0
76	**PC10**, 3 mol%	0.01 M EtOAc	$LiB(cat)_2$, 2 equiv., Cs_2CO_3, 2 equiv.	2 h	84	**0**	9	0
				5 h	26	**18**	22	0
			formate as the sacrificial reductant					
77	**PC15**, 5 mol%	0.01 M DMSO[a]	HCO_2Na, 3 equiv., Cs_2CO_3, 2 equiv.	2 h	4	**15**	32	13
78	**PC15**, 5 mol%	0.01 M EtOAc	HCO_2Na, 3 equiv., Cs_2CO_3, 2 equiv.	2 h	88	**0**	0	0
79	**PC2**, 5 mol%	0.01 M DMSO[a]	HCO_2Na, 3 equiv., Cs_2CO_3, 2 equiv.	2 h	0	**10**	46	2
80	**PC2**, 5 mol%	0.01 M EtOAc	HCO_2Na, 3 equiv., Cs_2CO_3, 2 equiv.	2 h	70	**12**	8	7
				5 h	60	**17**	11	8

№	photocatalyst	2e concentration & solvent	reductant / XAT agent / additives / other conditions	time	NMR yields (%)[b]			
					2e	3c	4e	5e
81	**PC2**, 5 mol%	0.01 M EtOAc[a]	HCO_2Na, 3 equiv., Cs_2CO_3, 2 equiv.	2 h	78	**10**	4	10
				5 h	45	**22**	10	15
82	**PC2**, 5 mol%	0.01 M EtOAc[a], water, 5 equiv.	HCO_2Na, 3 equiv., Cs_2CO_3, 2 equiv.	2 h	53	**19**	7	17
				5 h	25	**33**	11	25
83	**PC2**, 5 mol% 427 nm LED	0.01 M EtOAc	HCO_2Na, 3 equiv., Cs_2CO_3, 2 equiv.	2 h	70	**14**	5	12
				5 h	30	**30**	12	21
84	**PC2**, 5 mol% 390 nm LED	0.01 M EtOAc	HCO_2Na, 3 equiv., Cs_2CO_3, 2 equiv.	2 h	60	**19**	4	16
				5 h	20	**37**	9	28
85	**PC2**, 5 mol% 390 nm LED	0.01 M EtOAc/water 9:1	HCO_2Na, 3 equiv., Cs_2CO_3, 2 equiv.	2 h	91	**0**	4	0
86	**PC2**, 5 mol% 390 nm LED	0.01 M EtOAc	HCO_2Na, 3 equiv., 2,4,6-collidine, 2 equiv.	2 h	90	**0**	2	0
87	**PC1**, 3 mol%	0.01 M EtOAc	HCO_2Na, 3 equiv., Cs_2CO_3, 2 equiv.	2 h	75	**9**	5	0
				5 h	68	**11**	18	0
88	**PC8**, 3 mol%	0.01 M EtOAc	HCO_2Na, 3 equiv., Cs_2CO_3, 2 equiv.	2 h	97	**0**	3	0
89	**PC14**, 5 mol%	0.01 M EtOAc	HCO_2Na, 3 equiv., Cs_2CO_3, 2 equiv.	2 h	93	**0**	6	0
90	**PC3**, 5 mol%	0.01 M EtOAc	HCO_2Na, 3 equiv., Cs_2CO_3, 2 equiv.	2 h	95	**0**	5	0

[a] anhydrous solvent; [b] NMR conversion for substrate **2e** and NMR yields for products **3c**, **4e**, and **5e**; n.d. = the yield could not be determined due to overlapping NMR signals

Table S2. Selected entries from the optimization of the reaction conditions for the photoredox-mediated C-functionalization reaction with substrate **2d**.

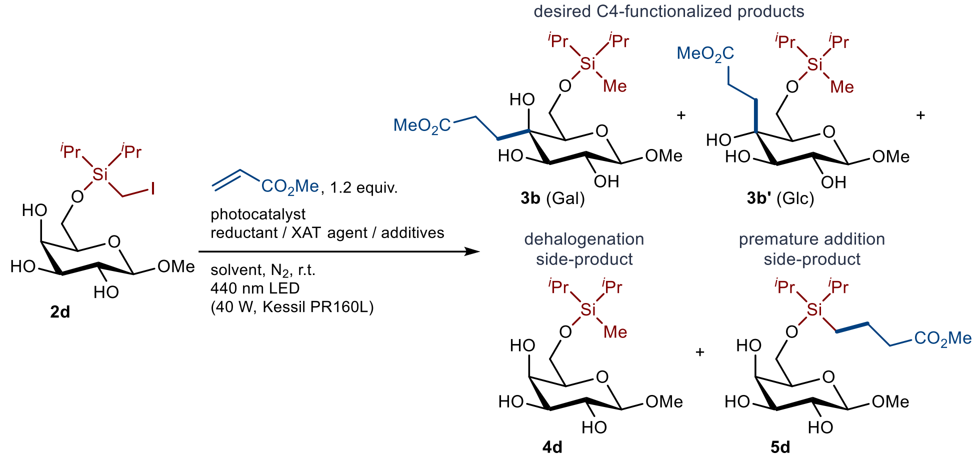

№	photocatalyst	2e concentration & solvent	reductant / XAT agent / additives / other conditions	time	NMR yields (%)[b]		
					2d	3b + 3b'	5d
			solvent and base screening				
1	**PC1**, 3 mol%	0.01 M $PhCF_3$[a]	$^iPr_2NEt$, 1.2 equiv., Cs_2CO_3, 2 equiv.	5 h	0	**11**	22
2	**PC1**, 3 mol%	0.01 M EtOAc	$^iPr_2NEt$, 1.2 equiv., Cs_2CO_3, 2 equiv.	2 h	2	**37**	27

№	photocatalyst	2e concentration & solvent	reductant / XAT agent / additives / other conditions	time	NMR yields (%)[b]		
					**2d**	**3b + 3b'**	**5d**
3	**PC1**, 3 mol%	0.01 M EtOAc/water 9:1	$^{i}Pr_2NEt$, 1.2 equiv., Cs_2CO_3, 2 equiv.	1 h	2	**18**	4
4	**PC1**, 3 mol%	0.01 M MeCN[a]	$^{i}Pr_2NEt$, 1.2 equiv., Cs_2CO_3, 2 equiv.	2 h	0	**29**	0
5	**PC1**, 3 mol%	0.01 M ^{t}BuCN[a]	$^{i}Pr_2NEt$, 1.2 equiv., Cs_2CO_3, 2 equiv.	1 h	20	**19**	33
				2 h	0	**22**	13
6	**PC1**, 3 mol%	0.01 M MeOAc	$^{i}Pr_2NEt$, 1.2 equiv., Cs_2CO_3, 2 equiv.	2 h	5	**23**	10
7	**PC1**, 3 mol%	0.01 M methyl propionate	$^{i}Pr_2NEt$, 1.2 equiv., Cs_2CO_3, 2 equiv.	2 h	5	**20**	10
8	**PC1**, 3 mol%	0.01 M methyl isobutyrate	$^{i}Pr_2NEt$, 1.2 equiv., Cs_2CO_3, 2 equiv.	2 h	0	**0**	5
9	**PC1**, 3 mol%	0.01 M ^{n}PrOAc	$^{i}Pr_2NEt$, 1.2 equiv., Cs_2CO_3, 2 equiv.	2 h	11	**11**	5
10	**PC1**, 3 mol%	0.01 M EtOAc	$^{i}Pr_2NEt$, 1.2 equiv., NaOAc, 2 equiv.	1 h	75	**3**	11
11	**PC1**, 3 mol%	0.01 M EtOAc	$^{i}Pr_2NEt$, 1.2 equiv., CsOAc, 2 equiv.	1 h	32	**23**	0
12	**PC1**, 3 mol%	0.01 M EtOAc	$^{i}Pr_2NEt$, 1.2 equiv., 2,4,6-collidine, 2 equiv.	1 h	81	**9**	6
13	**PC1**, 3 mol%	0.01 M EtOAc	$^{i}Pr_2NEt$, 1.2 equiv., $Bu_4NH_2PO_4$, 2 equiv.	1.5 h	19	**17**	11
14	**PC1**, 3 mol%	0.01 M EtOAc	$^{i}Pr_2NEt$, 1.2 equiv., $(Bu_4N)(BuO)_2PO_2$, 2 equiv.	1 h	34	**33**	56
15	**PC1**, 3 mol%	0.01 M EtOAc/CF_3CH_2OH 9:1	$^{i}Pr_2NEt$, 1.2 equiv., $Bu_4NH_2PO_4$, 2 equiv.	1 h	n.d.	**42**	12
			photocatalyst and other components screening				
16	**PC5**, 3 mol%	0.01 M EtOAc	$^{i}Pr_2NEt$, 1.2 equiv., Cs_2CO_3, 2 equiv.	2 h	55	**13**	26
17	**PC5**, 3 mol%	0.01 M $PhCF_3$[a]	$^{i}Pr_2NEt$, 1.2 equiv., Cs_2CO_3, 2 equiv.	2 h	39	**0**	16
18	**PC2**, 3 mol%	0.01 M EtOAc	$^{i}Pr_2NEt$, 1.2 equiv., Cs_2CO_3, 2 equiv.	2 h	0	**19**	n.d.
19	**PC6**, 3 mol%	0.01 M EtOAc	$^{i}Pr_2NEt$, 1.2 equiv., Cs_2CO_3, 2 equiv.	2 h	0	**34**	28
20	**PC6**, 3 mol%	0.01 M EtOAc	$^{i}Pr_2NEt$, 1.2 equiv., $CsHCO_3$, 2 equiv.	2 h	64	**0**	5
21	**PC6**, 3 mol%	0.01 M $PhCF_3$[a]	$^{i}Pr_2NEt$, 1.2 equiv., Cs_2CO_3, 2 equiv.	2 h	11	**25**	18
22	**PC6**, 3 mol%	0.01 M ^{i}PrOAc	$^{i}Pr_2NEt$, 1.2 equiv., Cs_2CO_3, 2 equiv.	2 h	0	**56**	28
23	**PC6**, 3 mol%	0.01 M ^{t}BuOAc	$^{i}Pr_2NEt$, 1.2 equiv., Cs_2CO_3, 2 equiv.	2 h	0	**39**	19
24	**PC6**, 3 mol%	0.01 M $(MeO)_2CO$	$^{i}Pr_2NEt$, 1.2 equiv., Cs_2CO_3, 2 equiv.	2 h	0	**27**	15
25	**PC6**, 3 mol%	0.01 M ^{i}PrOAc	$^{i}Pr_2NEt$, 1.2 equiv., Cs_2CO_3, 1 equiv.	2 h	0	**50**	23
26	**PC1**, 3 mol%	0.01 M ^{i}PrOAc	$^{i}Pr_2NEt$, 1.2 equiv., Cs_2CO_3, 1 equiv.	2 h	0	**35**	17
27	**PC6**, 3 mol%	0.01 M ^{i}PrOAc	$^{i}Pr_2NEt$, 1.2 equiv., Cs_2CO_3, 0.5 equiv.	2 h	60	**0**	7
28	**PC6**, 1 mol%	0.01 M ^{i}PrOAc	$^{i}Pr_2NEt$, 1.2 equiv., Cs_2CO_3, 1 equiv.	2 h	0	**32**	31
29	**PC6**, 5 mol%	0.01 M ^{i}PrOAc	$^{i}Pr_2NEt$, 1.2 equiv., Cs_2CO_3, 1 equiv.	2 h	0	**21**	9
30	**PC1**, 3 mol%	0.01 M EtOAc	$^{i}Pr_2NEt$, 1.2 equiv., Cs_2CO_3, 2 equiv.,	1 h	25	**30**	11
			1.5 equiv. of methyl acrylate	2 h	6	**37**	18

№	photocatalyst	2e concentration & solvent	reductant / XAT agent / additives / other conditions	time	NMR yields (%)[b]		
					2d	3b + 3b’	5d
31	**PC1**, 3 mol%	0.01 M EtOAc	$^{i}Pr_2NEt$, 1.2 equiv., Cs_2CO_3, 2 equiv., 2 equiv. of methyl acrylate	1 h<br>2 h	27<br>6	**17**<br>**34**	7<br>18
32	**PC1**, 3 mol%	0.02 M EtOAc	$^{i}Pr_2NEt$, 1.2 equiv., Cs_2CO_3, 2 equiv.	1 h	50	**7**	21
			supersilanol as the XAT-agent				
33	**PC1**, 3 mol%	0.01 M EtOAc	supersilanol, 1.5 eq., Cs_2CO_3, 2 eq.	2 h	0	**37**	n.d.
34	**PC1**, 3 mol%	0.01 M MeCN[a]	supersilanol, 1.5 eq., Cs_2CO_3, 2 eq.	2 h	0	**n.d.**	n.d.
35	**PC5**, 3 mol%	0.01 M EtOAc	supersilanol, 1.5 eq., Cs_2CO_3, 2 eq.	2 h	0	**n.d.**	n.d.
36	**PC5**, 3 mol%	0.01 M MeCN[a]	supersilanol, 1.5 eq., Cs_2CO_3, 2 eq.	2 h	0	**30**	n.d.
			additives				
37	**PC1**, 3 mol%	0.01 M $PhCF_3$[a]	$^{i}Pr_2NEt$, 1.2 equiv., Cs_2CO_3, 2 equiv., *p*-$ClC_6H_4CO_2(NBu_4)$, 0. 25 equiv.	1 h	25	**19**	9
38	**PC1**, 3 mol%	0.01 M $PhCF_3$[a]	$^{i}Pr_2NEt$, 1.2 equiv., *p*-$ClC_6H_4CO_2(NBu_4)$, 0. 25 equiv.	1 h	19	**4**	17
39	**PC1**, 3 mol%	0.01 M EtOAc	$^{i}Pr_2NEt$, 1.2 equiv., Cs_2CO_3, 2 equiv., *p*-$ClC_6H_4CO_2(NBu_4)$, 0. 25 equiv.	1 h	0	**34**	11
40	**PC1**, 3 mol%	0.01 M $PhCF_3$[a]	$^{i}Pr_2NEt$, 1.2 equiv., Cs_2CO_3, 2 equiv., *p*-$ClC_6H_4CO_2(NBu_4)$, 0. 25 equiv.	1 h	0	**10**	25
41	**PC1**, 3 mol%	0.01 M $PhCF_3$[a]	$^{i}Pr_2NEt$, 1.2 equiv., Cs_2CO_3, 2 equiv., $(Ph_2B)_2O$, 0.05 equiv.	1.5 h	0	**17**	49
			optimization towards the final conditions without base				
42	**PC1**, 3 mol%	0.01 M EtOAc	$^{i}Pr_2NEt$, 1.2 equiv., 1,2-dimethylimidazole, 2 equiv.	1 h	0	**9**	5
43	**PC1**, 3 mol%	0.01 M MeCN[a]	$^{i}Pr_2NEt$, 1.2 equiv., 1,2-dimethylimidazole, 2 equiv.	1 h	0	**32**	12
44	**PC1**, 3 mol%	0.01 M EtOAc	$^{i}Pr_2NEt$, 1.2 equiv., DABCO, 2 equiv.	1 h	0	**44**	17
45	**PC1**, 3 mol%	0.01 M $PhCF_3$[a]	$^{i}Pr_2NEt$, 1.2 equiv., DABCO, 2 equiv.	2 h	0	**21**	14
46	**PC1**, 3 mol%	0.01 M $^{i}PrOAc$	$^{i}Pr_2NEt$, 1.2 equiv., DABCO, 2 equiv.	2 h	100	**0**	0
47	**PC1**, 3 mol%	0.01 M $^{t}BuCN$	$^{i}Pr_2NEt$, 1.2 equiv., DABCO, 2 equiv.	2 h	0	**40**	6
48	**PC1**, 3 mol%	0.01 M MeCN	$^{i}Pr_2NEt$, 1.2 equiv., DABCO, 2 equiv.	1 h	0	**34**	14
49	**PC1**, 3 mol%	0.01 M $^{t}BuOH$	$^{i}Pr_2NEt$, 1.2 equiv., DABCO, 2 equiv.	1 h	0	**38**	31
50	**PC1**, 3 mol%	0.01 M EtOAc/$^{t}BuOH$ 9:1	$^{i}Pr_2NEt$, 1.2 equiv., DABCO, 2 equiv.	2 h	0	**52**	18
51	**PC1**, 3 mol%	0.01 M MeCN/$^{t}BuOH$ 9:1	$^{i}Pr_2NEt$, 1.2 equiv., DABCO, 2 equiv.	2 h	0	**38**	14
52	**PC1**, 3 mol%	0.01 M MeCN/$^{t}BuOH$ 9:1	$^{i}Pr_2NEt$, 1.2 equiv.	2 h	20	**33**	11
53	**PC1**, 3 mol%	0.01 M MeCN/$^{t}BuOH$ 9:1	$^{i}Pr_2NEt$, 2 equiv.	2 h	0	**37**	18

№	photocatalyst	2e concentration & solvent	reductant / XAT agent / additives / other conditions	time	NMR yields (%)[b]		
					**2d**	**3b + 3b'**	**5d**
54	**PC1**, 3 mol%	0.01 M MeCN/^{t}BuOH 9:1	^{i}Pr$_2$NEt, 3 equiv.	2 h	10	**48**	19
55	**PC1**, 3 mol%	0.01 M EtOAc/^{t}BuOH 9:1	^{i}Pr$_2$NEt, 2 equiv.	2 h	66	**9**	6
56	**PC1**, 3 mol%	0.01 M MeCN/^{t}BuOH 9:1	^{i}Bu$_3$N, 2 equiv.	2 h	37	**3**	5
57	**PC1**, 3 mol%	0.01 M MeCN/^{t}BuOH 9:1	Et$_3$N, 2 equiv.	2 h	30	**30**	25
58	**PC1**, 3 mol%	0.01 M MeCN/^{t}BuOH 9:1	^{n}Bu$_3$N, 2 equiv.	2 h	19	**41**	19
59	**PC4**, 3 mol%	0.01 M MeCN	^{i}Pr$_2$NEt, 2 equiv.	2 h	0	**0**	0
60	**PC6**, 3 mol%	0.01 M MeCN	^{i}Pr$_2$NEt, 2 equiv.	2 h	3	**33**	16
61	**PC2**, 3 mol%	0.01 M MeCN	^{i}Pr$_2$NEt, 2 equiv.	2 h	0	**13**	16
62	**PC13**, 3 mol%	0.01 M MeCN	^{i}Pr$_2$NEt, 2 equiv.	2 h	47	**15**	8
63	**PC1**, 3 mol%	0.02 M MeCN/^{t}BuOH 9:1	^{i}Pr$_2$NEt, 2 equiv.	2 h	0	**38**	22
64	**PC1**, 3 mol%	0.005 M MeCN/^{t}BuOH 9:1	^{i}Pr$_2$NEt, 2 equiv.	2 h	10	**32**	7
65	**PC1**, 3 mol%	0.01 M MeCN/^{t}BuOH 9:1	^{i}Pr$_2$NEt, 2 equiv., 1.6 equiv. of methyl acrylate	2 h	0	**37**	18
66[c]	**PC1**, 3 mol%	0.01 M MeCN/^{t}BuOH 9:1	^{i}Pr$_2$NEt, 2 equiv., 0.8 equiv. of methyl acrylate	2 h	0	**52**	8
67[c]	**PC1**, 3 mol%	0.02 M MeCN/^{t}BuOH 9:1	^{i}Pr$_2$NEt, 2 equiv., 0.8 equiv. of methyl acrylate	1.5 h	0	**47**	17
68[c]	**PC1**, 3 mol%	0.01 M MeCN/^{t}BuOH 9:1	^{i}Pr$_2$NEt, 2 equiv., 0.67 equiv. of methyl acrylate	2 h	0	**61**	6
69[c]	**PC1**, 3 mol%	0.01 M MeCN/^{t}BuOH 9:1	^{i}Pr$_2$NEt, 2 equiv., 0.5 equiv. of methyl acrylate	2 h	0	**57**	3

[a] anhydrous solvent; [b] NMR conversion for substrate **2b** and NMR yields for products **3b** and **3b'** (combined), and **5d**; n.d. = the yield could not be determined due to overlapping NMR signals; [c] NMR yields are calculated relative to the limiting reagent (methyl acrylate)

4. Investigation of the substrate scope

4.1 General procedure D: Photoredox-mediated C–H functionalization of the silylated carbohydrate substrates

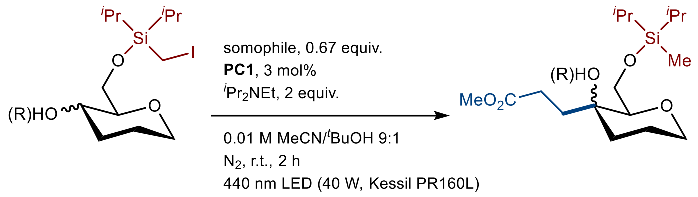

The carbohydrate substrate **2** (0.1 mmol, unless otherwise stated), photocatalyst **PC1** (3.36 mg, 0.003 mmol, 3 mol%), and solid somophile (0.667 equiv., when applicable) were placed into a dry 12 mL screw-neck vial (VWR, Art. No. MANA70285) equipped with a magnetic stirring bar (VWR, Art. No. 442-0401) and a septum (VWR, Art. No. 217-0183). The mixture was evacuated and backfilled with nitrogen three times, followed by addition of deaerated MeCN and ^{t}BuOH (9 mL and 1 mL, respectively, for the reaction performed on 0.1 mmol scale). The mixture was agitated or sonicated until all solids are dissolved. Subsequently, ^{i}Pr$_2$NEt (35 µL, 0.2 mmol, 2 equiv.) and liquid somophile (0.667 equiv., when applicable) were added to the reaction mixture, the reaction vial was disconnected from the nitrogen line and placed into a 3D-printed polypropylene holder ca. 2 cm from the LED light source (440 nm, 40 W Kessil PR160L). The reaction mixture was stirred at 1200 rpm under irradiation at room temperature with fan cooling (Figure S1). After 2 h, the reaction mixture was concentrated on a rotary evaporator and purified by column chromatography (*vide infra*), delivering the desired product **3**.

Note! For the majority of the reactions, the R_f values for substrate **2**, product **3**, and dehalogenated side-product **4** are very similar. Therefore, chromatographic purification of the crude product should be performed with considerably slow gradient of the specified eluents (*vide infra*), and close attention should be paid not only to the R_f values of the eluted products, but also to the color of the TLC spots upon TLC visualization. Figure S3 provides a representative example of a developed TLC plate featuring several combined chromatographic fractions from purification of product **3a**.

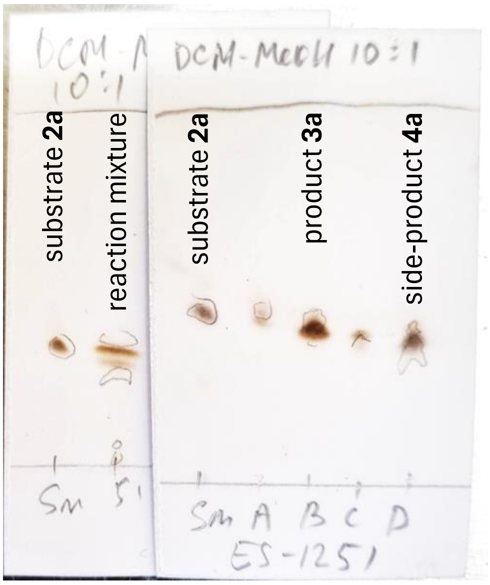

Figure S3. Representative TLC from the chromatographic purification of crude product **3a**. The TLC is visualized by dipping into 5% methanolic H_3PO_4 and heating with a heat gun.

4.2 Large-scale photochemical flow reaction

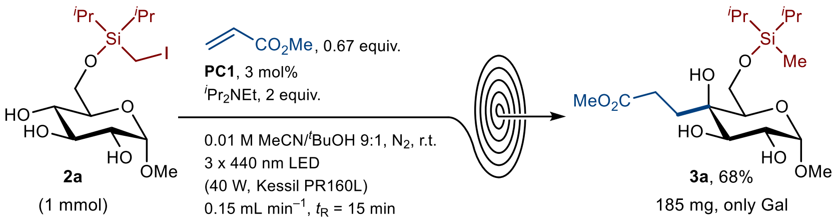

The carbohydrate substrate **2a** (448.4 mg, 1.0 mmol, 1 equiv.) and photocatalyst **PC1** (33.6 mg, 0.03 mmol, 3 mol%) were placed into a 250 mL pear-shaped flask, and the mixture was evacuated and backfilled with nitrogen three times. Subsequently, deaerated MeCN (90 mL) and ^{t}BuOH (10 mL) were added to the flask and the mixture was agitated until all solids are dissolved, followed by addition of $^i Pr_2NEt$ (3.0 mL, 2.0 mmol, 2 equiv.) and methyl acrylate (60 µL, 0.667 mmol, 0.667 equiv.). The photochemical flow reaction was conducted using three 3D-printed photochemical flow reactors connected in series and attached to three 440 nm LED light sources (440 nm, 40 W Kessil PR160L) (Figure S4). In the flow reactors, the reaction mixture is passed through a coiled plastic tubing from poly(tetrafluoroethylene-co-perfluoro(propylvinyl ether)) (CAS 26655-00-5, I.D. 0.8 mm, O.D. 1.6 mm, BOLA, Bohlender GmbH, Art. No. S2032-02) with a total length of ca. 3200 mm, total volume 1.6 mL (ca. 1070 mm per coil). Prior to the photoreaction, the end of the plastic tubing was inserted into 250 mL product collection flask through a septa, and the tubing was purged with nitrogen using a nitrogen balloon. The reaction mixture was taken with 50 mL syringe (refilled in the middle of the reaction) and connected to the flow reactor tubing with backflow of nitrogen to maintain the inert conditions. Both the syringe with the reaction mixture and the product collection flask were wrapped with aluminum foil. The reaction mixture was passed through the reactor tubing under irradiation with a syringe pump at 0.15 mL min^{-1} flow rate, 12 min residence time, 12 h total time. The reaction mixture was concentrated on a rotary evaporator and purified by column chromatography with gradient CH_2Cl_2/MeOH 50:1 → 20:1 as eluent, resulting in the desired product **3a** as yellowish amorphous solid (185 mg, 68%) and dehalogenated side-product **4a** as yellow foam (167 mg, 52%). The analytical data for product **3a** from the large-scale (1 mmol) flow reaction was identical to the analytical data from the small-scale (0.1 mmol) batch reaction (Section 4.1).

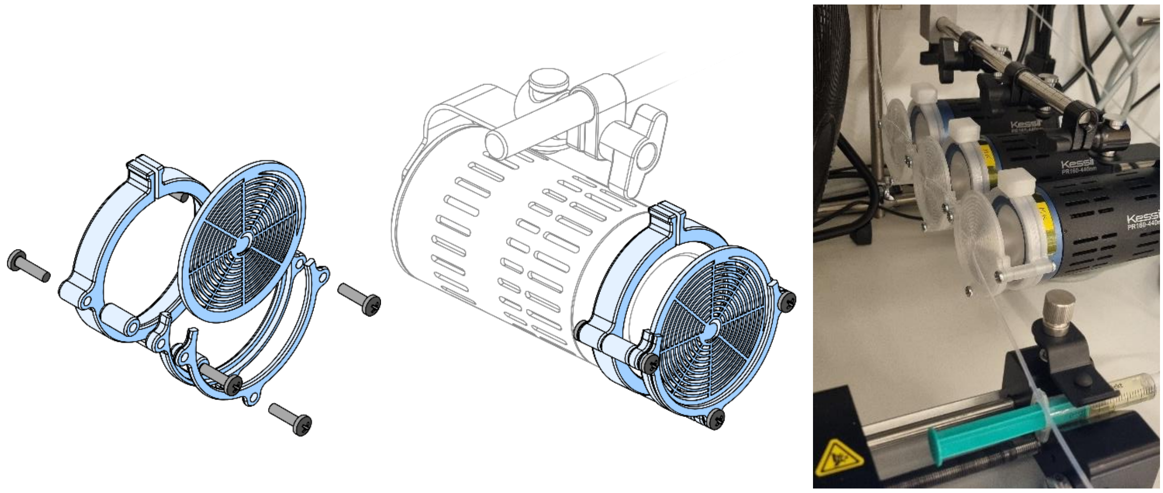

Figure S4. Scheme of the 3D-printed photochemical flow reactor and fully assembled setup for the synthesis of product **3a** using three reactors connected in series.

4.3 Removal of the silyl tethering group after the photoreaction

4.3.1 Removal of the silyl tethering group towards a lactone product

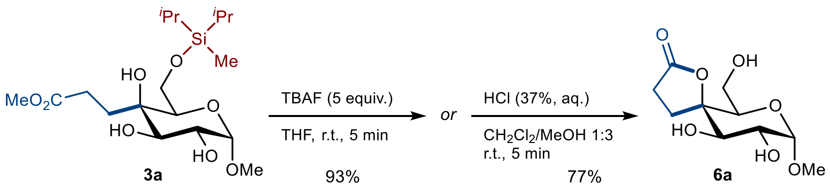

Silylated product **3a** (40.8 mg, 0.1 mmol, 1 equiv.) was dissolved in THF (3 mL), tetrabutylammonium fluoride (0.5 mL, 5 equiv., 1 M solution in THF) was added, and the reaction mixture was stirred for 5 min. The solvent was removed on a rotary evaporator and the crude product was purified by column chromatography with gradient CH_2Cl_2/MeOH 25:1 → 10:1 as eluent, resulting in the desired lactone product **6a** as a colorless oil (23 mg, 93%).

Alternatively, silylated product **3a** (40.8 mg, 0.1 mmol, 1 equiv.) was dissolved in CH_2Cl_2/MeOH 1:3 (4 mL), 37% aqueous HCl (0.5 mL) was added, and the reaction mixture was stirred for 5 min. The reaction mixture was neutralized by addition of Amberlite 21 ion-exchange resin until pH 7 (pH indicator paper), the resin was filtered off and washed with MeOH (40 mL). The solvents were removed on a rotary evaporator and the crude product was purified by column chromatography with gradient CH_2Cl_2/MeOH 25:1 → 10:1 as eluent, resulting in the desired lactone product **6a** as a colorless oil (19 mg, 77%). The analytical data for product **3a** from the two deprotection methods was identical.

4.3.2 Removal of the silyl tethering group towards a linear C-functionalized product

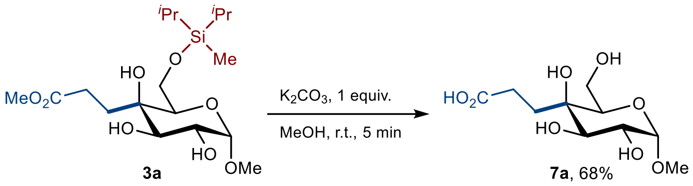

Silylated product **3a** (20.4 mg, 0.05 mmol, 1 equiv.) was dissolved in MeOH (3 mL), K_2CO_3 (6.9 mg, 1 equiv.) was added, and the reaction mixture was stirred for 5 min. The reaction mixture was neutralized by addition of Amberlite IR120 ion-exchange resin until pH 7 (pH indicator paper), the resin was filtered off and washed with MeOH (10 mL). The solvent was removed on a rotary evaporator and the crude product was purified by column chromatography with gradient CH_2Cl_2/MeOH 10:1 → 5:1 as eluent, resulting in the desired deprotected product **7a** as a yellowish solid (9 mg, 68%).

4.4 Analytical data for C-functionalized carbohydrate products

Compound **3a** (methyl 6-*O*-methyldiisopropylsilyl-4-(3-methoxy-3-oxopropyl)-α-D-galactopyranoside)

Synthesized according to **General procedure D** from silylated α-D-glucopyranoside **2a** and methyl acrylate. The crude product was purified by column chromatography with gradient CH_2Cl_2/MeOH 25:1→15:1 as the eluent, resulting in desired product **3a** as a yellow oil (18.5 mg, 69% yield). Employing silylated α-D-galactopyranoside **2b** as the starting material provided the desired product **3a** as a yellow oil (18.2 mg, 68% yield), displaying identical analytical data.

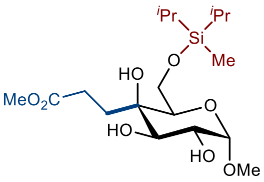

^{1}H NMR (500 MHz, $CDCl_3$) δ 4.80 (d, *J* = 4.0 Hz, 1H, H-1), 4.04 (dd, *J* = 11.4, 3.3 Hz, 1H, H-6a), 4.05 (s, 1H, OH-4), 3.98 (dd, *J* = 11.4, 3.0 Hz, 1H, H-6b), 3.84 (ddd, *J* = 8.8, 8.3, 4.0 Hz, 1H, H-2), 3.68 (s, 3H, OCH_3), 3.57 – 3.49 (m, 2H, H-3, H-5), 3.41 (s, 3H, CO_2CH_3), 2.57 – 2.39 (m, 3H, CH_2, OH-3), 2.14 (dq, *J* = 15.1, 7.7 Hz, 1H, CH_2), 2.09 (d, *J* = 8.3 Hz, 1H, OH-2), 1.99 (dq, *J* = 15.1, 7.7 Hz, 1H, CH_2), 1.08 – 0.90 (m, 14H, $SiCH(CH_3)_2$), 0.07 (s, 3H, $SiCH_3$).

^{13}C{^{1}H} NMR (126 MHz, $CDCl_3$) δ 174.4 ($\underline{C}O_2CH_3$), 99.3 (C-1), 74.8 (C-4), 72.9 (C-5), 70.7 (C-2 or C-3), 70.4 (C-2 or C-3), 63.7 (C-6), 55.4 ($CO_2\underline{C}H_3$), 51.8 (OCH_3), 30.2 (CH_2), 29.1 (CH_2), 17.31, 17.26, 17.23 ($CH(\underline{C}H_3)_2$), 12.8, 12.7 ($\underline{C}H(CH_3)_2$), −8.7 ($SiCH_3$).

***R*$_f$** = 0.35 (CH_2Cl_2/MeOH 10:1, red-orange color upon treatment with 5% methanolic H_3PO_4 and heating).

HRMS (ESI, m/z): calcd. for $[C_{18}H_{36}O_8SiNa]^+$, 431.2072; found, 431.2072

Compound **4a** (methyl 6-*O*-methyldiisopropylsilyl-α-D-glucopyranoside)

Synthesized according to the procedure described in **Section 4.2**.

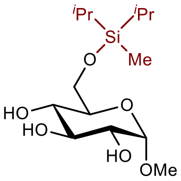

^{1}H NMR (500 MHz, $CDCl_3$) δ 4.75 (d, *J* = 3.8 Hz, 1H, H-1), 3.92 (dd, *J* = 10.4, 4.8 Hz, 1H, H-6a), 3.83 (dd, *J* = 10.4, 5.5 Hz, 1H, H-6b), 3.79 – 3.68 (m, 1H, H-3), 3.61 (ddd~dd, *J* = 10.1, 5.1 Hz, 1H, H-5), 3.59 – 3.51 (m, 1H, H-4), 3.50 (dd, *J* = 9.5, 3.8 Hz, 1H, H-2), 3.43 (s, 3H), 2.12 (d, *J* = 9.4 Hz, 1H, OH-2), 1.48 (d, *J* = 6.6 Hz, 1H, OH-4), 1.05 – 0.92 (m, 14H), 0.06 (s, 3H).

^{13}C{^{1}H} NMR (126 MHz, $CDCl_3$) δ 99.1 (C-1), 74.7 (C-3), 72.7 (C-4), 72.2 (C-2), 70.2 (C-5), 64.6 (C-6), 55.3 (OCH_3), 17.34, 17.32, 17.28 ($CH(\underline{C}H_3)_2$), 12.84, 12.80 ($\underline{C}H(CH_3)$, −8.7 ($SiCH_3$).

***R*$_f$** = 0.55 (CH_2Cl_2/MeOH 2:1, brown-gray color upon treatment with 5% methanolic H_3PO_4 and heating).

HRMS (ESI, m/z): calcd. for $[C_{14}H_{30}O_6SiNa]^+$, 345.1704; found, 345.1706

Compounds **3b/3b'** (methyl 6-*O*-methyldiisopropylsilyl-4-(3-methoxy-3-oxopropyl)-β-D-galacto/glucopyranoside)

Synthesized according to **General procedure D** from silylated β-D-glucopyranoside **2c** and methyl acrylate. The crude product was purified by column chromatography with gradient CH_2Cl_2/MeOH 25:1→15:1 as the eluent, resulting in desired product **3b/3b'** (Gal/Glc 1:1) as a brown oil (16.9 mg, 62% yield). Employing silylated β-D-galactopyranoside **2d** as the starting material provided the desired product **3b/3b'** (Gal/Glc 2:1) as a brown oil (18.6 mg, 70% yield). NMR description for product **3b/3b'** from **2c** is provided.

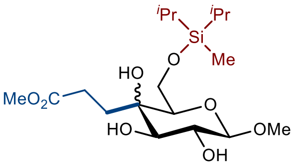

^{1}H NMR (500 MHz, $CDCl_3$) δ 4.38 (d, *J* = 7.9 Hz, 1H, H-1 Gal), 4.16 (d, *J* = 7.8 Hz, 1H, H-1 Glu), 4.04 (dd~t, *J* = 3.2 Hz, 2H, H-6 Gal), 3.76 (d, *J* = 9.0 Hz, 1H, H-3 Gal), 3.75 – 3.70 (m, 2H, H-5 Glu, H-6a Glu), 3.69 (s, 3H, CO_2CH_3), 3.67 (s, 3H, CO_2CH_3), 3.62 (dd, *J* = 9.1, 7.8 Hz, 1H, H-2 Glu), 3.55 (dd, *J* = 9.9, 1.2 Hz, 1H, H-6b Glu), 3.54 (s, 3H, OCH_3 Glu), 3.50 (s, 3H, OCH_3 Gal), 3.37 (dd~t, *J* = 8.4 Hz, 1H, H-2 Gal), 3.32 (dd, *J* = 9.1, 7.2 Hz, 1H, H-3 Glu), 3.25 (dd~t, *J* = 3.2 Hz, 1H, H-5 Gal), 3.08 (s, 1H), 2.87 (d, *J* = 7.2 Hz, 1H, OH-3 Glu), 2.81 (br.s, 1H, OH), 2.79 (br.s, 1H, OH), 2.57 – 2.44 (m, 2H, CH_2, Gal), 2.37 (dd, *J* = 9.2, 7.1 Hz, 2H, CH_2, Gal), 2.32 – 2.21 (m, 1H, CH_2, Glu), 2.12 (ddd, *J* = 14.4, 9.1,

6.4 Hz, 1H, CH_2, Glu), 2.08 – 1.91 (m, 2H, CH_2, Glu), 1.05 – 0.91 (m, 28H, $SiCH(CH_3)_2$, Glu, Gal), 0.06 (s, 6H, $SiCH_3$, Glu, Gal).

$^{13}C\{^1H\}$ NMR (126 MHz, $CDCl_3$) δ 174.3, 173.6 ($\underline{C}O_2CH_3$), 103.5 (C-1 Glu), 99.1 (C-1 Gal), 76.8 (C-5 Glu), 75.9 (C-5 Gal), 75.7 (C-4 Glu or Gal), 75.6 (C-3 Glu), 74.2 (C-4 Glu or Gal), 73.9 (C-2 Gal), 72.73 (C-2 Glu or C-3 Gal), 72.71 (C-2 Glu or C-3 Gal), 68.7 (C-6 Glu), 63.3 (C-6 Gal), 57.0 (OCH_3), 56.6 (OCH_3), 51.85 ($CO_2\underline{C}H_3$), 51.80 ($CO_2\underline{C}H_3$), 29.8 (CH_2, Gal), 28.9 (CH_2, Gal), 26.9 (CH_2, Glu), 21.4 (CH_2, Glu), 17.32, 17.28, 17.24, 17.21, 17.18 ($CH(\underline{C}H_3)_2$), 12.82, 12.80, 12.7, 12.6 ($\underline{C}H(CH_3)_2$), −8.72 ($SiCH_3$), −8.79 ($SiCH_3$).

R_f = 0.35 (CH_2Cl_2/MeOH 10:1, red-orange color upon treatment with 5% methanolic H_3PO_4 and heating).

HRMS (ESI, m/z): calcd. for $[C_{18}H_{36}O_8SiNa]^+$, 431.2072; found, 431.2071

Compound **3c** (1,2:3,4-di-*O*-isopropylidene-6-*O*-methyldiisopropylsilyl-4-(3-methoxy-3-oxopropyl)-α-D-galactopyranose)

Synthesized according to **General procedure D** from silylated β-D-galactopyranose **2c**. The crude product was purified by column chromatography with gradient hexane/EtOAc 20:1→5:1 as the eluent, resulting in the desired product **3b** as a yellow oil (16.1 mg, 51% yield).

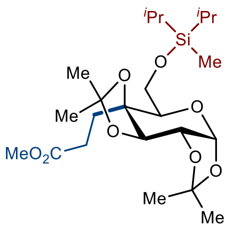

^1H NMR (500 MHz, $CDCl_3$) δ 5.55 (d, *J* = 5.2 Hz, 1H, H-1), 4.38 (dd, *J* = 5.2, 3.2 Hz, 1H, H-2), 4.27 (d, *J* = 3.2 Hz, 1H, H-3), 3.97 (dd, *J* = 11.1, 4.2 Hz, 1H, H-6a), 3.72 (dd, *J* = 11.1, 6.4 Hz, 1H, H-6b), 3.67 (s, 3H, OCH_3), 3.66 (dd, *J* = 4.2, 6.4 Hz, 1H, H-5), 2.53 (ddd, *J* = 16.2, 11.0, 5.5 Hz, 1H, CH_2), 2.37 (ddd, *J* = 16.2, 11.0, 5.4 Hz, 1H, CH_2), 2.21 – 2.12 (m, 1H, CH_2), 1.98 (ddd, *J* = 14.4, 11.0, 5.5 Hz, 1H, CH_2), 1.54 (s, 3H), 1.42 (s, 3H), 1.37 (s, 3H), 1.32 (s, 3H), 1.10 – 0.92 (m, 14H, $SiCH(CH_3)_2$), 0.04 (s, 3H, $SiCH_3$).

$^{13}C\{^1H\}$ NMR (126 MHz, $CDCl_3$) δ 173.6 ($\underline{C}O_2CH_3$), 110.1 ($\underline{C}(CH_3)_2$), 109.1 ($\underline{C}(CH_3)_2$), 96.4 (C-1), 80.5 (C-4), 74.9 (C-3), 72.6 (C-5), 70.8 (C-2), 62.6 (C-6), 51.6 (OCH_3), 31.1 (CH_2), 28.5 (CH_2), 27.3, 27.1, 25.7, 25.0 ($C(\underline{C}H_3)_2$), 17.39, 17.35, 17.33 ($CH(\underline{C}H_3)_2$), 13.0, 12.9 ($\underline{C}H(CH_3)_2$), −8.6 ($SiCH_3$).

R_f = 0.32 (hexane/EtOAc 10:1, red-orange color upon treatment with 5% methanolic H_3PO_4 and heating).

HRMS (ESI, m/z): calcd. for $[C_{23}H_{42}O_8SiNa]^+$, 497.2541; found, 497.2552

Compound **3d** (methyl 6-*O*-methyldiisopropylsilyl-3,4-O-isopropylidene-4-(3-methoxy-3-oxopropyl)-α-D-galactopyranoside)

Synthesized according to **General procedure D** from silylated α-D-galactopyranoside **2e** and methyl acrylate. The crude product was purified by column chromatography with gradient hexane/EtOAc 5:1→2:1 as the eluent, resulting in the desired product **3d** as a brown oil (8 mg, 27% yield).

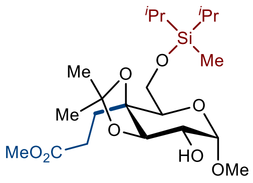

^1H NMR (500 MHz, $CDCl_3$) δ 4.79 (d, *J* = 4.8 Hz, 1H, H-1), 4.22 (dd, *J* = 4.8, 3.8 Hz, 1H, H-2), 4.20 (d, *J* = 3.8 Hz, 1H, H-3), 3.92 (dd, *J* = 11.0, 2.8 Hz, 1H, H-6a), 3.77 (dd, *J* = 7.4, 2.8 Hz, 1H, H-5), 3.72 (dd, *J* = 11.0, 7.4 Hz, 1H, H-6b), 3.66 (s, 3H, OCH_3), 3.52 (s, 3H, OCH_3), 2.55 (ddd, *J* = 16.3, 10.8, 5.6 Hz, 1H), 2.39 (ddd, *J* = 16.3, 10.8, 5.6 Hz, 1H, CH_2), 2.11 (ddd, *J* = 14.5, 10.8, 5.7 Hz, 1H, CH_2), 1.98 (ddd, *J* = 14.5, 10.8, 5.6 Hz, 1H, CH_2), 1.43 (s, 3H, $C(CH_3)_2$), 1.36 (s, 3H, $C(CH_3)_2$), 1.15 – 0.88 (m, 14H, $SiCH(CH_3)_2$), 0.04 (s, 3H, $SiCH_3$).

^{13}C{^{1}H} NMR (126 MHz, $CDCl_3$) δ 173.7 ($\underline{C}O_2CH_3$), 110.3 ($\underline{C}(CH_3)_2$), 95.1 (C-1), 81.4 (C-4), 77.0 C-3), 74.4 C-5), 65.3 (C-2), 62.6 (C-6), 54.7 (OCH_3), 51.6 (OCH_3), 30.7 (CH_2), 28.4 (CH_2), 27.4 ($C(\underline{C}H_3)_2$), 27.1 ($C(\underline{C}H_3)_2$), 17.4, 17.36, 17.33 ($CH(\underline{C}H_3)_2$), 13.0, 12.9 ($\underline{C}H(CH_3)_2$), −8.4 ($SiCH_3$).

***R*$_f$** = 0.34 (hexane/EtOAc 3:1, brown color upon treatment with 5% methanolic H_3PO_4 and heating).

HRMS (ESI, m/z): calcd. for $[C_{21}H_{40}O_8SiNa]^+$, 471,2385; found, 471.2386

Compounds **3e**/**3e'** (methyl 6-*O*-methyldiisopropylsilyl-3,4-*O*-isopropylidene-4-(3-methoxy-3-oxopropyl)-β-D-galacto/glucopyranoside)

Synthesized according to **General procedure D** from silylated β-D-galactopyranoside **2g** and methyl acrylate. The crude product was purified by column chromatography with gradient hexane/EtOAc 5:1→2:1 as the eluent, resulting in the desired products **3e** (Gal) (7.1 mg, 24% yield) and **3e'** (Glc) (11.3 mg, 38% yield) as brown oils.

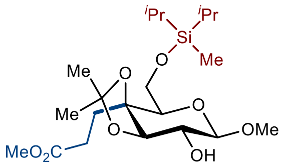

Analytical data for compound **3e**:

^{1}H NMR (500 MHz, $CDCl_3$) δ 4.29 (d, *J* = 5.2 Hz, 1H, H-1), 4.02 (d, *J* = 4.7 Hz, 1H, H-3), 3.95 (dd, *J* = 11.4, 3.4 Hz, 1H, H-6a), 3.87 (dd~br.t, *J* = 5.1 Hz, 1H, H-2), 3.79 (dd, *J* = 11.4, 6.9 Hz, 1H, H-6b), 3.68 (s, 3H, CO_2CH_3), 3.59 (dd, *J* = 7.0, 3.4 Hz, 1H, H-5), 3.53 (s, 3H, OCH_3), 2.46 (ddd, *J* = 9.1, 6.5, 3.7 Hz, 2H, CH_2), 2.26 (br.s, 1H, OH-2), 2.11 (ddd, *J* = 14.4, 9.2, 6.8 Hz, 1H, CH_2), 1.96 (ddd, *J* = 14.4, 9.5, 6.5 Hz, 1H, CH_2), 1.47 (s, 3H, $C(CH_3)_2$), 1.40 (s, 3H, $C(CH_3)_2$), 1.07 – 0.94 (m, 14H, $SiCH(CH_3)_2$), 0.05 (s, 3H, $SiCH_3$).

^{13}C{^{1}H} NMR (126 MHz, $CDCl_3$) δ 173.7 ($\underline{C}O_2CH_3$), 110.7 ($\underline{C}(CH_3)_2$), 104.6 (C-1), 82.3 (C-3), 81.9 (C-4), 79.0 C-5), 72.3 (C-2), 63.0 (C-6), 56.8 (OCH_3), 51.8 (OCH_3), 31.9 (CH_2), 28.9 (CH_2), 28.1 ($C(\underline{C}H_3)_2$), 27.9 ($C(\underline{C}H_3)_2$), 17.40, 17.36, 17.32, 17.27 ($CH(\underline{C}H_3)_2$), 13.0, 12.9 ($\underline{C}H(CH_3)_2$), −8.6 ($SiCH_3$).

***R*$_f$** = 0.43 (hexane/EtOAc 2:1, brown color upon treatment with 5% methanolic H_3PO_4 and heating).

HRMS (ESI, m/z): calcd. for $[C_{21}H_{40}O_8SiNa]^+$, 471.2385; found, 471.2386

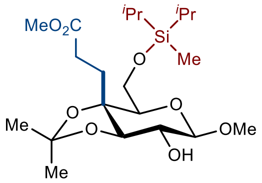

Analytical data for compound **3e'**:

^{1}H NMR (500 MHz, $CDCl_3$) δ 4.32 (d, *J* = 8.2 Hz, 1H, H-1), 4.13 (dd, *J* = 7.9, 4.9 Hz, 1H, H-3), 3.99 (d, *J* = 5.1 Hz, 1H, H-5), 3.90 (d, *J* = 9.4 Hz, 1H, H-6a), 3.70 (s, 3H, CO_2CH_3), 3.58 – 3.52 (m, 1H, H-2), 3.50 (s, 3H, OCH_3), 3.46 (d, *J* = 9.4 Hz, 1H, H-6b), 2.52 – 2.37 (m, 2H, CH_2), 2.37 (d, *J* = 2.7 Hz, 1H, OH-2), 2.21 (ddd, *J* = 14.5, 11.1, 6.2 Hz, 1H, CH_2), 1.91 (ddd, *J* = 15.1, 11.0, 4.4 Hz, 1H, CH_2), 1.50 (s, 3H, $C(CH_3)_2$), 1.32 (s, 3H, $C(CH_3)_2$), 1.14 – 0.80 (m, 14H, $SiCH(CH_3)_2$), 0.02 (s, 3H, $SiCH_3$).

^{13}C{^{1}H} NMR (126 MHz, $CDCl_3$) δ 173.6 ($\underline{C}O_2CH_3$), 109.4 ($\underline{C}(CH_3)_2$), 98.5 (C-1), 77.2 (C-3), 76.5 (C-4), 76.0 (C-5), 73.5 (C-2), 62.3 (C-6), 56.9 (OCH_3), 51.8 (OCH_3), 28.3 ($C(\underline{C}H_3)_2$), 26.8 (CH_2), 26.3 ($C(\underline{C}H_3)_2$), 25.5 (CH_2), 17.40, 17.28, 17.24, 17.22 ($CH(\underline{C}H_3)_2$), 13.0, 12.8 ($\underline{C}H(CH_3)_2$), −8.9 ($SiCH_3$).

***R*$_f$** = 0.23 (hexane/EtOAc 2:1, red-orange color upon treatment with 5% methanolic H_3PO_4 and heating).

HRMS (ESI, m/z): calcd. for $[C_{21}H_{40}O_8SiNa]^+$, 471.2385; found, 471.2383

Compound **3f** (4-methoxyphenyl 6-*O*-methyldiisopropylsilyl-4-(3-methoxy-3-oxopropyl)-β-D-galacto/glucopyranoside)

Synthesized according to **General procedure D** from silylated β-D-glucopyranoside **2h** and methyl acrylate. The crude product was purified by column chromatography with hexane/EtOH 10:1 as the eluent, resulting in the desired product **3f** (Gal/Glc 1:1) as a brown oil (20.6 mg, 62% yield).

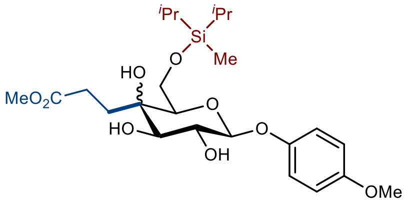

^{1}H NMR (500 MHz, $CDCl_3$) δ 7.06 – 6.98 (m, 4H, $C_6H_4OCH_3$), 6.85 – 6.76 (m, 4H, $C_6H_4OCH_3$), 4.95 (d, *J* = 7.8 Hz, 1H, H-1 Gal), 4.72 (d, *J* = 7.8 Hz, 1H, H-1 Glu), 4.05 – 4.02 (m, 2H, H-6 Gal), 3.92 – 3.87 (m, 1H, H-2 Glu), 3.85 – 3.80 (m, 2H, H-3 Gal, H-5 Glu), 3.77 (s, 6H, OCH_3 Gal, Glu), 3.69 (s, 3H, OCH_3 Gal or Glu), 3.67 – 3.63 (m, 2H, H-2 Gal, H-6a Glu), 3.62 (s, 3H, OCH_3 Gal or Glu), 3.59 (d, *J* = 9.8 Hz, 1H, H-6b Glu), 3.44 – 3.40 (m, 1H, H-3 Glu), 3.34 (t, *J* = 3.2 Hz, 1H, H-5 Gal), 2.64 – 2.43 (m, 2H, CH_2), 2.29 – 2.18 (m, 2H, CH_2), 2.19 – 2.08 (m, 2H, CH_2), 2.08 – 1.95 (m, 2H, CH_2), 1.00 – 0.97 (m, 28H, $SiCH(CH_3)_2$), 0.05, 0.03 (s, 6H, $SiCH_3$).

^{13}C{^{1}H} NMR (126 MHz, $CDCl_3$) δ 174.3, 173.6 (CO_2CH_3), 155.9, 155.4, 151.2, 150.7, 120.0, 118.9, 118.6, 117.8, 114.5, 114.4 ($C_6H_4OCH_3$), 102.2 (C-1 Glu), 98.7 (C-1 Gal), 76.5 (C-4 Gal), 76.4 (C-5 Glu), 76.3 (C-5 Gal), 75.6 (C-3 Glu), 74.1 (C-4 Glu), 73.9 (C-2 Gal), 72.7 (C-3 Gal), 72.6 (C-2 Glu), 68.7 (C-6 Gal), 63.2 (C-6 Glu), 55.62 (OCH_3), 55.59 (OCH_3), 51.9 (OCH_3), 51.8 (OCH_3), 29.9 (CH_2), 28.9 (CH_2), 26.9 (CH_2), 21.5 (CH_2), 17.42, 17.33, 17.29, 17.26, 17.23, 17.22 ($CH(\underline{C}H_3)_2$), 12.80, 12.78, 12.73, 12.59 ($\underline{C}H(CH_3)_2$), −8.7, −8.8 ($SiCH_3$).

***R*$_f$** = 0.35 (CH_2Cl_2/MeOH 10:1, brown color upon treatment with 5% methanolic H_3PO_4 and heating).

HRMS (ESI, m/z): calcd. for $[C_{24}H_{40}O_9SiNa]^+$, 523.2334; found, 523.2332

Compound **3g** (6-*O*-(6-*O*-methyldiisopropylsilyl-4-(3-methoxy-3-oxopropyl)-β-D-glucopyranosyl)-1,2:3,4-di-*O*-isopropylidene-α-D-galacto/glucopyranoside)

Synthesized according to **General procedure D** from silylated disaccharide **2i** and methyl acrylate on 0.074 mmol scale. The crude product was purified by column chromatography with gradient CH_2Cl_2/MeOH 25:1→10:1 as the eluent, resulting in the desired product **3g** (Gal/Glc 1:1) as a brownish foam (12.5 mg, 40% yield).

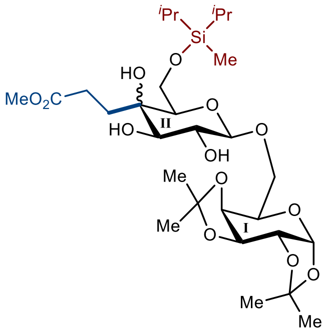

^{1}H NMR (500 MHz, $CDCl_3$) δ 5.54 (2 dd, *J* = 5.0, Hz, 2H, H-1 Gal I), 4.64 – 4.57 (m, 3H), 4.35 (d, *J* = 8.0 Hz, 1H, H-1 Glu II), 4.32 (dd, *J* = 5.0, 2.5 Hz, 1H, H-2 Gal I), 4.24 (dd, *J* = 7.9, 1.8 Hz, 1H), 4.21 (dd, *J* = 7.9, 1.7 Hz, 1H), 4.08 – 3.92 (m, 6H), 3.82 – 3.76 (m, 2H), 3.78 – 3.67 (m, 4H), 3.68 (s, 3H, OCH_3), 3.67 (s, 3H, OCH_3), 3.55 (d, *J* = 9.6 Hz, 1H, H-6), 3.45 – 3.38 (m, 1H), 3.37 (br.s, 1H), 3.26 (dd~t, *J* = 3.4 Hz, 1H), 2.51 (ddd, *J* = 15.1, 9.4, 6.6 Hz, 2H), 2.42 (td, *J* = 11.3, 5.8 Hz, 1H), 2.34 (td, *J* = 10.6, 5.3 Hz, 1H), 2.27 (ddd, *J* = 20.7, 11.1, 5.5 Hz, 1H), 2.14 (ddd, *J* = 14.4, 9.3, 6.5 Hz, 1H), 2.04 (td, *J* = 12.2, 11.5, 6.4 Hz, 1H), 1.98 (ddd, *J* = 14.4, 7.7, 5.3 Hz, 1H), 1.54 (s, 3H), 1.53 (s, 3H), 1.45 (s, 3H), 1.44 (s, 3H), 1.33 (s, 12H), 1.03 – 0.95 (m, 28H, $SiCH(CH_3)_2$), 0.06 (s, 6H, $SiCH_3$).

^{13}C{^{1}H} NMR (126 MHz, $CDCl_3$) δ 174.2, 173.6 ($\underline{C}O_2CH_3$), 109.6, 109.5, 108.9, 108.8 ($C(CH_3)_2$), 103.5 (C-1 Glu II), 99.5 (C-1 Gal II), 96.29, 96.27 (C-1 Gal I), 76.5, 76.1, 75.8, 75.4, 74.1, 73.7, 72.7, 71.1, 70.74,

70.73, 70.4, 68.80, 68.78, 68.2, 67.8, 63.4, 51.8, 29.9, 29.0, 26.9, 25.99, 25.97, 25.94, 25.88, 24.9, 24.5, 24.4, 21.3, 17.33, 17.31, 17.27, 17.24, 17.23, 12.83, 12.81, 12.7, 12.6, −8.7, −8.8.

***R*~f~** = 0.31 (CH_2Cl_2/MeOH 10:1, brown color upon treatment with 5% methanolic H_3PO_4 and heating).

HRMS (ESI, m/z): calcd. for $[C_{29}H_{52}O_{13}SiNa]^+$, 659.3069; found, 659.3071

Compound **3h** ((5-bromo-4-chloro-1*H*-indol-3-yl)-6-*O*-methyldiisopropylsilyl-4-(3-methoxy-3-oxopropyl)-β-D-galacto/glucopyranoside)

Synthesized according to **General procedure D** from silylated X-Gal-derived substrate **2j** and methyl acrylate. The crude product was purified by column chromatography with gradient CH_2Cl_2/MeOH 50:1→10:1 as the eluent, resulting in the desired product **3h** (Gal/Glc 1:1) as a green foam (20.0 mg, 38% yield).

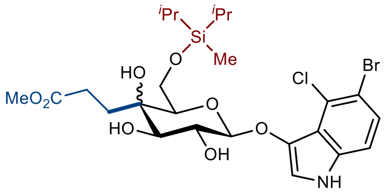

^{1}H NMR (500 MHz, acetone-*d*$_6$) δ 10.24 (s, 2H, NH), 7.35 – 7.28 (m, 4H), 7.28 – 7.21 (m, 2H), 4.99 (d, *J* = 7.4 Hz, 1H, H-1 Gal), 4.77 (d, *J* = 7.9 Hz, 1H, H-1 Glu), 4.16 (s, 1H, OH), 4.13 (dd, *J* = 11.0, 3.7 Hz, 1H, H-6a Glu), 4.09 (d, *J* = 9.6 Hz, 1H, H-6a Gal), 3.96 – 3.95 (m, 1H, H-2 Gal), 3.93 (dd, *J* = 11.0, 5.8 Hz, 1H, H-6b Glu), 3.89 – 3.84 (m, 2H, H-1 Glu, H-5 Gal), 3.83 – 3.79 (m, 1H, H-3 Gal), 3.75 – 3.71 (m, 2H, 2 × OH), 3.67 (dd, *J* = 5.8, 3.6 Hz, 1H, H-5 Glu), 3.62 (s, 3H, OCH_3), 3.54 (d, *J* = 9.5 Hz, 1H, H-6b Gal), 3.54 (m, overlapped, 1H, H-3 Glu), 3.53 (s, 3H, OCH_3), 2.51 (ddd, *J* = 16.4, 10.8, 5.7 Hz, 2H, CH_2), 2.41 – 2.30 (m, 2H, CH_2), 2.19 (ddd, *J* = 14.1, 11.0, 5.6 Hz, 1H, CH_2), 2.12 – 2.06 (m, 2H, CH_2), 1.98 – 1.88 (m, 1H, CH_2), 1.04 – 0.94 (m, 28H, $SiCH(CH_3)_2$), 0.06 (s, 6H, $SiCH_3$).

^{13}C{^{1}H} NMR (126 MHz, acetone-*d*$_6$) δ 174.2, 174.0 ($\underline{C}O_2CH_3$), 138.4, 137.5, 134.6, 126.8, 124.95, 124.89, 119.7, 119.3, 115.2, 114.0, 113.2, 112.84, 112.77 (aglycone), 105.1 (C-1 Glu), 102.3 (C-1 Gal), 79.9 (C-4), 78.6 (C-5 Glu), 75.6 (C-3 Glu), 74.4 (C-4), 73.0 (C-5 Gal), 72.4 (C-2 Gal), 72.0 (C-2 Glu), 71.1 (C-3 Gal), 63.5 (C-6), 63.4 (C-6), 51.69, 51.66 ($CO_2\underline{C}H_3$), 30.7 (CH_2), 29.1 (CH_2), 28.3 (CH_2), 25.4 (CH_2), 17.93, 17.86, 17.83, 17.80, 17.77, 17.74, 17.72 ($CH(\underline{C}H_3)_2$), 13.82, 13.80, 13.68, 13.60 ($\underline{C}H(CH_3)_2$), −8.4, −8.5 ($SiCH_3$).

***R*~f~** = 0.35 (CH_2Cl_2/MeOH 10:1, brown color upon treatment with 5% methanolic H_3PO_4 and heating).

HRMS (ESI, m/z): calcd. for $[C_{25}H_{37}BrClNO_8SiNa]^+$, 644.1053; found, 644.1050

Compound **3i** ((((2,6-dimethoxybenzoyl)oxy)methyl)phenyl 6-*O*-methyldiisopropylsilyl-4-(3-methoxy-3-oxopropyl)-β-D-galacto/glucopyranoside)

Synthesized according to **General procedure D** from silylated curculigoside G substrate **2j**. The crude product was initially purified by column chromatography with gradient CH_2Cl_2/MeOH 50:1→10:1 as the eluent, followed by purification of the fractions containing the desired product by another chromatographic column with gradient hexane/EtOAc 1:1→1:3 as the eluent, resulting in the desired product **3i** (Gal/Glc 1:1) as a yellowish oil (11.5 mg, 26% yield).

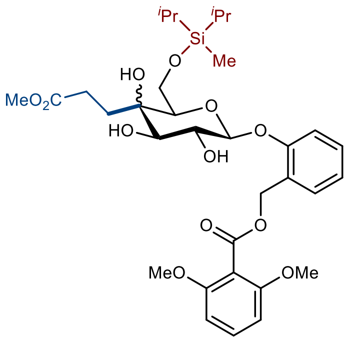

^{1}H NMR (500 MHz, $CDCl_3$) δ 7.47 – 7.35 (m, 2H), 7.33 – 7.22 (m, 4H), 7.22 – 7.18 (m, 2H), 7.03 (t, *J* = 7.4 Hz, 2H), 6.58 – 6.49 (m, 4H), 5.77 (d, *J* = 11.8 Hz, 1H), 5.57 (d, *J* = 12.0 Hz, 1H), 5.39 (d, *J* = 12.0 Hz, 1H), 5.18 (d, *J* = 11.8 Hz, 1H), 4.83 (d, *J* = 7.7 Hz, 1H), 4.79 (d, *J* = 7.9 Hz, 1H), 4.16 – 4.13 (m, 1H), 4.12 (d, *J* = 7.2 Hz, 1H), 4.11 – 4.02 (m, 2H), 4.01 – 3.87 (m, 3H), 3.85 (dd, *J* = 10.9, 5.4 Hz, 1H), 3.80 (s, 6H), 3.77 (s, 9H), 3.69 (s, 3H), 3.66 – 3.60 (m, 2H), 3.50 – 3.44 (m, 2H), 2.81 (ddd, *J* = 17.3, 11.3, 6.9 Hz, 1H), 2.72 (br.s, 1H), 2.67 (br.s, 1H), 2.59 – 2.52 (m, 2H), 2.51 – 2.44 (m, 1H), 2.34 (td, *J* = 12.5, 11.7, 4.3 Hz, 1H), 2.24 – 2.13 (m, 2H), 2.00 (ddd, *J* = 15.1, 9.1, 6.8 Hz, 1H), 1.10 – 0.86 (m, 28H), 0.07 (s, 3H), 0.04 (s, 3H).

^{13}C NMR (126 MHz, $CDCl_3$) δ 176.9, 169.6, 167.1, 166.5, 157.42, 157.35, 156.4, 156.0, 131.3, 131.2, 130.9, 130.3, 129.9, 125.5, 125.0, 122.8, 122.5, 115.6, 115.4, 104.2, 104.0, 102.6, 101.9, 82.6, 77.8, 76.5, 71.3, 69.8, 69.3, 62.84, 62.70, 61.8, 56.1, 56.0, 29.7, 28.7, 28.4, 26.3, 25.0, 17.38, 17.35, 17.34, 17.32, 17.27, 17.22, 17.20, 14.19, 14.11, 12.83, 12.81, 12.79, 12.76, -8.6, -8.7.

***R*$_f$** = 0.31 (CH_2Cl_2/MeOH 10:1, brown color upon treatment with 5% methanolic H_3PO_4 and heating).

HRMS (ESI, m/z): calcd. for $[C_{33}H_{48}O_{12}Na]^+$, 687.2807; found, 687.2820

Compound **3j** (methyl 6-*O*-methyldiisopropylsilyl-4-(3-methoxy-2-methyl-3-oxopropyl)-α-D-galactopyranoside)

Synthesized according to **General procedure D** from silylated α-D-glucopyranoside **2a** and methyl methacrylate. The crude product was purified by column chromatography with gradient CH_2Cl_2/MeOH 25:1→15:1 as the eluent, resulting in the desired product **3j** (d.r. 1:3) as a brownish foam (13.3 mg, 32% yield). NMR assignment of the major diastereomer is reported.

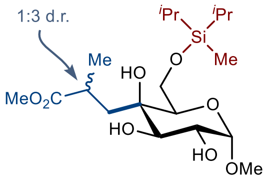

^{1}H NMR (500 MHz, $CDCl_3$) δ 4.81 (d, *J* = 3.9 Hz, 1H, H-1), 4.06 – 3.95 (m, 2H, H-6), 3.89 (s, 1H, OH-4), 3.83 (ddd, *J* = 9.2, 7.1, 3.9 Hz, 1H, H-2), 3.69 (s, 3H, CO_2CH_3), 3.55 (dd~t, *J* = 2.7 Hz, 1H, H-5), 3.42 – 3.40 (m, 2H, H-3), 3.41 (s, 3H, OCH_3), 2.86 (d, *J* = 6.8 Hz, 1H, CH), 2.85 – 2.80 (m, 1H, OH-3), 2.28 (dd, *J* = 14.7, 10.2 Hz, 1H, CH_2), 2.21 (d, *J* = 7.2 Hz, 1H, OH-2), 1.61 (dd, *J* = 14.7, 3.0 Hz, 1H, CH_2), 1.20 (d, *J* = 7.1 Hz, 3H, CH_3), 1.09 – 0.83 (m, 14H, $SiCH(CH_3)_2$), 0.07 (s, 3H, $SiCH_3$).

^{13}C{^{1}H} NMR (126 MHz, $CDCl_3$) δ 178.5 (CO), 99.1 (C-1), 74.7 (C-4), 73.4 (C-3), 71.4 (C-5), 70.2 (C-2), 63.4 (C-6), 55.3 (OCH_3), 52.1 (OCH_3), 39.7 (CH_2), 35.6 (CH), 19.9 (CH_3), 17.34, 17.31, 17.28, 17.20, 17.17 ($CH(\underline{C}H_3)_2$), 12.79, 12.77 ($\underline{C}H(CH_3)_2$), −8.7 ($SiCH_3$).

***R*$_f$** = 0.55 (CH_2Cl_2/MeOH 10:1, brown-orange color upon treatment with 5% methanolic H_3PO_4 and heating).

HRMS (ESI, m/z): calcd. for $[C_{19}H_{38}O_8SiNa]^+$, 445.2228; found, 445.2230

Compound **3k** (methyl 6-*O*-methyldiisopropylsilyl-4-(3-oxo-3-(phenylamino)propyl)-α-D-galactopyranoside)

Synthesized according to **General procedure D** from silylated α-D-glucopyranoside **2a** and N-phenylacrylamide. The crude product was purified by column chromatography with gradient CH_2Cl_2/MeOH 25:1→15:1 as the eluent, resulting in the desired product **3k** as a brownish foam (18 mg, 55% yield).

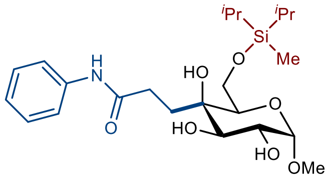

^{1}H NMR (500 MHz, $CDCl_3$) δ 8.11 (s, 1H, NH), 7.52 (d, *J* = 7.8 Hz, 2H), 7.28 (dd, *J* = 7.8, 7.4 Hz, 2H), 7.07 (t, *J* = 7.4 Hz, 1H), 4.79 (d, *J* = 3.9 Hz, 1H, H-1), 4.10 (dd, *J* = 11.5, 3.6 Hz, 1H, H-6a), 3.97 (dd, *J* = 11.5, 2.8 Hz, 1H, H-6b), 3.94 – 3.88 (m, 1H, H-2), 3.80 – 3.73 (m, 1H, OH-3), 3.66 (dd, *J* = 9.4, 6.8 Hz, 1H, H-3), 3.54 (dd~br.t, *J* = 3.2 Hz, 1H, H-5), 3.35 (s, 3H, OCH_3), 2.59 (ddd, *J* = 15.2, 8.6, 6.6 Hz, 1H, CH_2), 2.49 (ddd, *J* = 15.1, 8.7, 6.4 Hz, 1H, CH_2), 2.21 (ddd, *J* = 15.0, 8.8, 6.6 Hz, 1H, CH_2), 2.03 (ddd, *J* = 14.7, 8.6, 6.5 Hz, 1H, CH_2), 1.05 – 0.89 (m, 14H, $SiCH(CH_3)_2$), 0.06 (s, 3H, $SiCH_3$).

^{13}C{^{1}H} NMR (126 MHz, $CDCl_3$) δ 171.7 (<u>C</u>=O), 138.1, 128.9, 124.1, 120.0, 99.4 (C-1), 75.2 (C-4), 72.6 (C-3), 70.6 (C-2 or C-5), 70.4(C-2 or C-5), 63.7 (C-6), 55.3 (OCH_3), 32.4 (CH_2), 30.9 (CH_2), 17.32, 17.27 (2 × C), 17.25 (CH(<u>C</u>$H_3)_2$), 12.8, 12.7 (<u>C</u>$H(CH_3)_2$), −8.6 ($SiCH_3$).

***R*$_f$** = 0.38 (CH_2Cl_2/MeOH 10:1, brown-red color upon treatment with 5% methanolic H_3PO_4 and heating).

HRMS (ESI, m/z): calcd. for $[C_{23}H_{39}NO_7SiNa]^+$, 492.2388; found, 492.2389

Compound **3l** (methyl 6-*O*-methyldiisopropylsilyl-4-(3-(dimethylamino)-3-oxopropyl)-α-D-galactopyranoside)

Synthesized according to **General procedure D** from silylated α-D-glucopyranoside **2a** and *N*,*N*-dimethylacrylamide. The crude product was purified by column chromatography with gradient CH_2Cl_2/MeOH 25:1→15:1 as the eluent, resulting in the desired product **3l** as a brownish foam (16 mg, 57% yield).

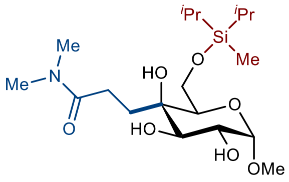

^{1}H NMR (500 MHz, $CDCl_3$) δ 4.81 (d, *J* = 3.9 Hz, 1H, H-1), 4.02 (dd, *J* = 11.2, 3.8 Hz, 1H, H-6a), 3.92 (dd, *J* = 11.2, 4.4 Hz, 1H, H-6b), 3.85 (dd, *J* = 9.2, 3.9 Hz, 1H, H-2), 3.59 (dd~br.t, *J* = 4.0 Hz, 1H, H-5), 3.51 (d, *J* = 9.2 Hz, 1H, H-3), 3.42 (s, 3H, OCH_3), 3.00 (s, 3H, NCH_3), 2.95 (s, 3H, NCH_3), 2.54 – 2.44 (m, 2H, CH_2CO), 2.18 (ddd, *J* = 14.2, 8.3, 5.6 Hz, 1H, CH_2), 1.92 (ddd, *J* = 14.7, 7.0, 5.4 Hz, 1H, CH_2), 1.04 – 0.93 (m, 14H, $SiCH(CH_3)_2$), 0.05 (s, 3H, $SiCH_3$).

^{13}C{^{1}H} NMR (126 MHz, $CDCl_3$) δ 173.6 (<u>C</u>=O), 99.1 (C-1), 74.5 (C-4), 72.3 (C-3), 71.9 (C-5), 70.0 (C-2), 63.1 (C-6), 55.3 (OCH_3), 37.3 (C(O)N(<u>C</u>$H_3)_2$), 35.8 (C(O)N(<u>C</u>$H_3)_2$), 29.7 (CH_2), 28.3 (CH_2), 17.34, 17.30, 17.27 (CH(<u>C</u>$H_3)_2$), 12.83, 12.81 (<u>C</u>$H(CH_3)_2$), −8.6 ($SiCH_3$).

***R*$_f$** = 0.42 (CH_2Cl_2/MeOH 10:1, brown-orange color upon treatment with 5% methanolic H_3PO_4 and heating).

HRMS (ESI, m/z): calcd. for $[C_{19}H_{39}NO_7SiNa]^+$, 444.2388; found, 444.2393

Compound **3m** (methyl 6-*O*-methyldiisopropylsilyl-4-(2-(phenylsulfonyl)ethyl)-α-D-galactopyranoside)

Synthesized according to **General procedure D** from silylated α-D-glucopyranoside **2a** and phenyl vinyl sulfone. The crude product was purified by column chromatography with gradient CH_2Cl_2/MeOH 25:1→15:1 as the eluent, resulting in the desired product **3m** as a brownish foam (18 mg, 55% yield).

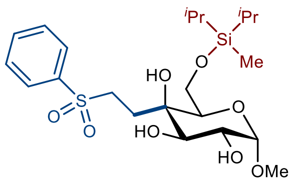

^{1}H NMR (500 MHz, $CDCl_3$) δ 7.94 – 7.87 (m, 2H), 7.69 – 7.62 (m, 1H), 7.59 – 7.53 (m, 2H), 4.79 (d, *J* = 4.0 Hz, 1H, H-1), 4.10 (dd, *J* = 11.7, 2.9 Hz, 1H, H-6a), 3.93 (dd, *J* = 11.7, 2.4 Hz, 1H, H-6b), 3.77 (td, *J* = 9.0, 4.0 Hz, 1H, H-2), 3.55 – 3.45 (m, 1H, CH_2SO_2), 3.47 – 3.43 (m, 2H, H-3, H-5), 3.39 (s, 3H, OCH_3), 3.38 – 3.31 (m, 1H, CH_2SO_2), 2.33 (d, *J* = 7.3 Hz, 1H, OH-3), 2.16 (ddd, *J* = 13.9, 12.2, 4.3 Hz, 1H, CH_2), 2.09 (d, *J* = 9.0 Hz, 1H, OH-2), 2.01 (ddd, *J* = 13.9, 12.2, 4.9 Hz, 1H, CH_2), 1.02 – 0.92 (m, 14H, $SiCH(CH_3)_2$), 0.06 (s, 3H, $SiCH_3$).

^{13}C{^{1}H} NMR (126 MHz, $CDCl_3$) δ 139.1, 133.7, 129.3 (2 × CH), 128.0 (2 × CH), 99.3 (C-1), 74.5 (C-4), 74.1 (C-3 or C-5), 70.7 (C-2), 70.2 (C-3 or C-5), 64.0 (C-6), 55.5 (OCH_3), 52.0 (CH_2SO_2), 28.7 (CH_2), 17.27, 17.20, 17.17 ($CH(\underline{C}H_3)_2$), 12.74, 12.62 ($\underline{C}H(CH_3)_2$), −8.8 ($SiCH_3$).

***R*$_f$** = 0.31 (CH_2Cl_2/MeOH 10:1, brown-orange color upon treatment with 5% methanolic H_3PO_4 and heating).

HRMS (ESI, m/z): calcd. for $[C_{22}H_{38}O_8SSiNa]^+$, 513,1949; found, 513.1952

Compound **3n** (methyl 6-*O*-methyldiisopropylsilyl-4-(2-(dimethoxyphosphoryl)ethyl)-α-D-galactopyranoside)

Synthesized according to **General procedure D** from silylated α-D-glucopyranoside **2a** and dimethyl vinylphosphonate. The crude product was purified by column chromatography with gradient CH_2Cl_2/MeOH 25:1→15:1 as the eluent, resulting in the desired product **3n** as a brownish foam (20 mg, 65% yield).

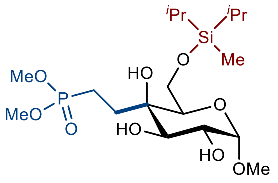

^{1}H NMR (500 MHz, $CDCl_3$) δ 4.81 (d, *J* = 3.9 Hz, 1H, H-1), 4.32 – 4.25 (m, 1H, OH), 4.02 – 3.93 (m, 2H, H-6), 3.93 – 3.86 (m, 1H, H-2), 3.75 (d, *J* = 7.1 Hz, 3H, $P(OCH_3)_2$), 3.73 (d, *J* = 7.1 Hz, 3H, $P(OCH_3)_2$), 3.67 (d, *J* = 8.1 Hz, 1H, H-3), 3.62 (br.s., 1H, OH), 3.56 (t, *J* = 3.4 Hz, 1H, H-5), 3.40 (s, 3H, OCH_3), 2.13 – 2.01 (m, 1H, CH_2), 2.00 – 1.85 (m, 3H, CH_2CH_2P), 1.57 (d, *J* = 6.6 Hz, 6H), 1.03 – 0.92 (m, 14H, $CH(CH_3)_2$), 0.06 (s, 3H, $SiCH_3$).

^{13}C{^{1}H} NMR (126 MHz, $CDCl_3$) δ 99.4 (C-1), 75.2 (d, *J* = 15.4 Hz, C-4), 71.6 (C-3), 70.2 (C-2 or C-5), 70.1 (C-2 or C-5), 63.6 (C-6), 55.3 (OCH_3), 52.8 (d, *J* = 6.6, Hz $P(OCH_3)_2$), 52.7 (d, *J* = 6.7 Hz, $P(OCH_3)_2$), 48.3, 27.2 (d, *J* = 4.3 Hz, $\underline{C}H_2CH_2P$), 19.4 (d, *J* = 141.0 Hz, $CH_2\underline{C}H_2P$), 17.31, 17.28, 17.24, 17.23, 17.21 ($CH(CH_3)_2$), 12.73, 12.67 ($\underline{C}H(CH_3)_2$), −8.7 ($SiCH_3$).

***R*$_f$** = 0.29 (CH_2Cl_2/MeOH 10:1, brown-red color upon treatment with 5% methanolic H_3PO_4 and heating).

HRMS (ESI, m/z): calcd. for $[C_{18}H_{39}O_9PSiNa]^+$, 481.1993; found, 481,1993

Compound **3o** (methyl 6-*O*-methyldiisopropylsilyl-4-(2-cyanoethyl)-α-D-galactopyranoside)

Synthesized according to **General procedure D** from silylated α-D-glucopyranoside **2a** and acrylonitrile. The crude product was purified by column chromatography with gradient CH_2Cl_2/MeOH 25:1→15:1 as the eluent, resulting in the desired product **3o** as a brownish foam (12.8 mg, 47% yield).

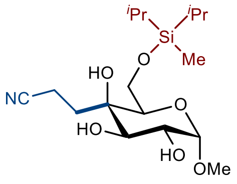

^{1}H NMR (500 MHz, $CDCl_3$) δ 4.81 (d, *J* = 3.9 Hz, 1H, H-1), 4.37 (s, 1H, OH-4), 4.11 (dd, *J* = 11.6, 2.8 Hz, 1H, H-6a), 3.99 (dd, *J* = 11.6, 2.8 Hz, 1H, H-6b), 3.86 – 3.77 (m, 1H, H-2), 3.59 – 3.52 (m, 1H, H-3), 3.51 (t, *J* = 2.8 Hz, 1H, H-5), 3.41 (s, 3H, OCH_3), 2.71 (ddd, *J* = 16.9, 10.2, 6.5 Hz, 1H, CH_2), 2.62 (ddd, *J* = 17.1, 10.1, 5.5 Hz, 1H, CH_2), 2.50 (d, *J* = 7.0 Hz, 1H, OH-3), 2.20 (d, *J* = 9.6 Hz, 1H, OH-2), 2.12 (ddd, *J* = 14.1, 10.2, 5.5 Hz, 1H, CH_2), 2.01 (ddd, *J* = 14.1, 10.1, 6.5 Hz, 1H, CH_2), 1.09 – 0.89 (m, 14H, $SiCH(CH_3)_2$), 0.09 (s, 3H, $SiCH_3$).

^{13}C{^{1}H} NMR (126 MHz, $CDCl_3$) δ 120.2 (CN), 99.3 (C-1), 74.3 (C-4), 73.9 (C-3), 70.7 (C-2), 70.1 (C-5), 64.0 (C-6), 55.5 (OCH_3), 32.1 (CH_2), 17.28, 17.20, 17.17 (CH(<u>C</u>H$_3$)$_2$), 12.74, (2 × <u>C</u>H(CH$_3$)$_2$), 12.68 (CH_2), −8.7 ($SiCH_3$).

***R*$_f$** = 0.55 (CH_2Cl_2/MeOH 10:1, brown-red color upon treatment with 5% methanolic H_3PO_4 and heating).

HRMS (ESI, m/z): calcd. for $[C_{17}H_{33}NO_6SiNa]^+$, 398.1969; found, 398.1970

Compound **3p** (methyl 6-*O*-methyldiisopropylsilyl-4-(3-oxocyclopentyl)-α-D-galactopyranoside)

Synthesized according to **General procedure D** from silylated α-D-glucopyranoside **2a** and 2-cyclopenten-1-one. The crude product was purified by column chromatography with gradient CH_2Cl_2/MeOH 25:1→15:1 as the eluent, resulting in the desired product **3p** (mixture of two diastereomers, d.r. 2:1) as a brownish foam (6 mg, 22% yield).

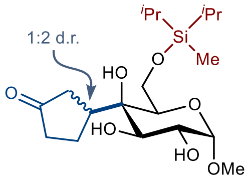

^{1}H NMR (500 MHz, $CDCl_3$) for *major diastereomer*: δ 4.81 (d, *J* = 3.8 Hz, 1H, H-1), 4.31 (s, 1H, OH-4), 4.08 (dd, *J* = 11.6, 2.9 Hz, 1H, H-6a), 4.00 (dd, *J* = 11.6, 2.6 Hz, 1H, H-6b), 3.89 – 3.82 (m, 1H, H-2), 3.68 (dd, *J* = 9.1, 6.3 Hz, 1H, H-3), 3.53 (dd~t, *J* = 2.8 Hz, 1H, H-5), 3.41 (s, 3H, OCH_3), 2.73 – 2.60 (m, 1H, CH_2), 2.46 – 2.03 (m, 8H, CH_2, OH-3, OH-2), 1.09 – 0.89 (m, 14H, $CH(CH_3)_2$), 0.07 (s, 3H, $SiCH_3$). **^{1}H NMR** (500 MHz, $CDCl_3$) for *minor diastereomer*: δ 4.81 (d, *J* = 3.5 Hz, 1H, H-1), 4.24 (s, 1H, OH-4), 4.12 (dd, *J* = 11.5, 3.1 Hz, 1H, H-6a), 4.02 (dd, *J* = 11.5, 2.7 Hz, 1H, H-6b), 3.89 – 3.82 (m, 1H, H-2), 3.77 (dd, *J* = 9.2, 6.0 Hz, 1H, H-3), 3.65 (dd~t, *J* = 2.8 Hz, 1H, H-5), 3.42 (s, 3H, OCH_3), 2.89 (ddd, *J* = 18.5, 10.8, 1.5 Hz, 1H, CH_2), 2.73 – 2.60 (m, 1H, CH_2), 2.46 – 2.03 (m, 6H, CH_2, OH-3, OH-2), 1.89 (dtd, *J* = 12.7, 10.2, 8.5 Hz, 1H, CH_2), 1.09 – 0.89 (m, 14H, $CH(CH_3)_2$), 0.08 (s, 3H, $SiCH_3$).

^{13}C{^{1}H} NMR (126 MHz, $CDCl_3$) for *major diastereomer*: δ 218.1 (CO), 99.1 (C-1), 76.2 (C-4), 71.2 (C-3), 71.1 (C-2), 70.0 (C-5), 64.4 (C-6), 55.5 (OCH_3), 43.5, 40.8, 38.8, 25.0 (all CH_2), 17.30, 17.22, 17.18 (CH(<u>C</u>H$_3$)$_2$), 12.8, 12.7 (<u>C</u>H(CH$_3$)$_2$), −8.7 ($SiCH_3$). **^{13}C NMR** (126 MHz, $CDCl_3$) for *minor diastereomer*: δ 218.4 (CO), 99.1 (C-1), 76.0 (C-4), 72.4 (C-3), 71.1 (C-2), 69.7 (C-5), 64.3 (C-6), 55.5 (OCH_3), 43.5, 41.5, 38.4, 24.8 (all CH_2), 17.30, 17.22, 17.18 (CH(<u>C</u>H$_3$)$_2$), 12.8, 12.7 (<u>C</u>H(CH$_3$)$_2$), −8.7 ($SiCH_3$).

***R*$_f$** = 0.58 (CH_2Cl_2/MeOH 10:1, brown-red color upon treatment with 5% methanolic H_3PO_4 and heating).

HRMS (ESI, m/z): calcd. for $[C_{19}H_{36}O_7SiNa]^+$, 427.2123; found, 427.2123

Compound **3q** (methyl 6-*O*-methyldiisopropylsilyl-4-(2-(bis(*tert*-butoxycarbonyl)amino)-3-methoxy-3-oxopropyl)-α-D-galactopyranoside)

Synthesized according to **General procedure D** from silylated α-D-glucopyranoside **2a** and methyl 2-(bis(*tert*-butoxycarbonyl)amino)acrylate.[16] The crude product was purified by column chromatography with gradient CH_2Cl_2/MeOH 25:1→15:1 as the eluent, resulting in the desired product **3q** (mixture of two diastereomers, d.r. 1.4:1) as a brownish foam (13.3 mg, 32% yield).

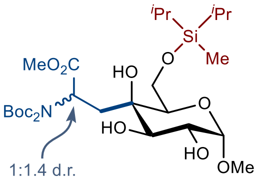

^{1}H NMR (500 MHz, $CDCl_3$) δ 5.40 (dd, *J* = 6.7, 4.3 Hz, 1H, CHN, *major diastereomer*), 5.16 (dd, *J* = 7.0, 3.7 Hz, 1H, CHN, *minor diastereomer*), 4.84 (d, *J* = 3.9 Hz, 1H, H-1 *minor diastereomer*), 4.81 (d, *J* = 4.0 Hz, 1H, H-1 *major diastereomer*), 4.18 – 4.12 (m, 2H, H-6, OH-4), 3.99 – 3.95 (m, 3H, H-6), 3.93 (ddd, *J* = 9.3, 5.1, 3.9 Hz, 1H, H-2 *minor diastereomer*), 3.84 (ddd~td, *J* = 9.0, 4.0 Hz, 1H, H-2 *major diastereomer*), 3.74 (s, 3H, OCH_3, *minor diastereomer*), 3.71 (s, 3H, OCH_3, *major diastereomer*), 3.69 – 3.60 (m, 4H, H-3, H-5), 3.42 (s, 3H, OCH_3, *minor diastereomer*), 3.40 (s, 3H, OCH_3, *major diastereomer*), 3.37 (d, *J* = 6.0 Hz, 1H, OH-3 *minor diastereomer*), 2.89 (dd, *J* = 15.5, 7.1 Hz, 1H, CH_2, *minor diastereomer*), 2.64 (d, *J* = 6.7 Hz, 1H, OH-3, *major diastereomer*), 2.60 (dd, *J* = 15.5, 4.3 Hz, 1H, CH_2, *major diastereomer*), 2.27 (dd, *J* = 15.4, 6.7 Hz, 1H, CH_2, *major diastereomer*), 2.22 (d, *J* = 7.0 Hz, 1H, OH-2, *minor diastereomer*), 2.07 (d, *J* = 8.5 Hz, 1H, OH-2, *major diastereomer*), 1.85 (dd, *J* = 15.6, 3.6 Hz, 1H, CH_2, *minor diastereomer*), 1.49 (s, 36H, 4 × $C(CH_3)_3$), 1.04 – 0.90 (m, 28H, $SiCH(CH_3)_2$), 0.06 (s, 3H, $SiCH_3$, *major diastereomer*), 0.04 (s, 2H, $SiCH_3$, *minor diastereomer*).

^{13}C{^{1}H} NMR (126 MHz, $CDCl_3$) δ 173.5 (C=O, *minor diastereomer*), 171.8 (C=O, *major diastereomer*), 152.2 (C=O, *major diastereomer*), 151.7 (C=O, *minor diastereomer*), 99.2 (C-1, *major diastereomer*), 99.1 (C-1, *minor diastereomer*), 83.6 ($\underline{C}(CH_3)_3$, *minor diastereomer*), 83.3 ($\underline{C}(CH_3)_3$, *minor diastereomer*), 74.7 (C-4, *major diastereomer*), 74.5 (C-4, *minor diastereomer*), 73.9 (*major diastereomer*), 72.4 (*minor diastereomer*), 71.3 (*minor diastereomer*), 71.0 (*major diastereomer*), 70.7(C-2, *major diastereomer*), 69.9 (C-2, *minor diastereomer*), 63.71 (C-6, *major diastereomer*), 63.66 (C-6, *minor diastereomer*), 55.29 (OCH_3, *minor diastereomer*), 55.26 (OCH_3, *major diastereomer*), 54.6 (CHN, *minor diastereomer*), 54.5 (CHN, *major diastereomer*), 52.9 (OCH_3, *minor diastereomer*), 52.5 (OCH_3, *major diastereomer*), 37.9 (CH_2, *minor diastereomer*), 36.9 (CH_2, *major diastereomer*), 27.98 ($C(\underline{C}H_3)_3$, *major diastereomer*), 27.96 ($C(\underline{C}H_3)_3$, *minor diastereomer*), 17.33, 17.31, 17.29, 17.27 ($CH(\underline{C}H_3)_2$), 12.83, 12.80, 12.72 ($\underline{C}H(CH_3)_2$), −8.70, −8.67 ($SiCH_3$).

***R*$_f$** = 0.55 (CH_2Cl_2/MeOH 10:1, brown-orange color upon treatment with 5% methanolic H_3PO_4 and heating).

HRMS (ESI, m/z): calcd. for $[C_{28}H_{53}NO_{12}SiNa]^+$, 646.3229; found, 646.3242

Compound **3r** (methyl *E*-6-*O*-methyldiisopropylsilyl-4-(3-ethoxy-3-oxoprop-1-en-1-yl)-α-D-galactopyranoside)

Synthesized according to **General procedure D** from silylated α-D-glucopyranoside **2a** (1 equiv.) and ethyl (2Z)-3-bromo-2-propenoate (2 equiv.). The crude product was purified by column chromatography with gradient CH_2Cl_2/MeOH 25:1→15:1 as the eluent, resulting in the desired product **3r** (only *trans* isomer) as a brownish foam (14 mg, 33% yield).

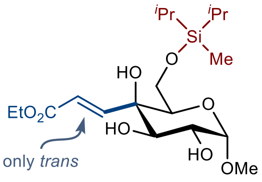

^{1}H NMR (500 MHz, $CDCl_3$) δ 6.85 (d, *J* = 15.4 Hz, 1H), 6.31 (d, *J* = 15.4 Hz, 1H), 4.88 (d, *J* = 3.9 Hz, 1H, H-1), 4.66 (s, 1H, OH-4), 4.21 (q, *J* = 7.1 Hz, 2H, $C\underline{H_2}CH_3$), 3.93 (dd, *J* = 11.4, 3.2 Hz, 1H, H-6a), 3.91 – 3.89 (m, 1H, H-2), 3.87 (dd, *J* = 11.4, 2.4 Hz, 1H, H-6b), 3.68 (dd~br.t, *J* = 2.7 Hz, 1H, H-5), 3.64 (dd, *J* = 9.4, 7.6 Hz, 1H, H-3), 3.44 (s, 3H, OCH_3), 2.21 (d, *J* = 7.6 Hz, 1H, OH-3), 2.12 (d, *J* = 8.5 Hz, 1H, OH-2), 1.29 (t, *J* = 7.1 Hz, 3H, $CH_2C\underline{H_3}$), 1.03 – 0.87 (m, 14H, $SiCH(CH_3)_2$), 0.04 (s, 3H, $SiCH_3$).

^{13}C{^{1}H} NMR (126 MHz, $CDCl_3$) δ 166.0 (C=O), 147.3 (CH=CH), 123.7 (CH=CH), 99.5 (C-1), 77.5, 73.0, 70.2, 69.9, 64.3 (C-6), 60.5 ($\underline{C}H_2CH_3$), 55.5 (OCH_3), 17.32, 17.24, 17.20, 17.16 ($CH(\underline{C}H_3)_2$), 14.2 ($CH_2\underline{C}H_3$), 12.77, 12.68 ($\underline{C}H(CH_3)_2$), −8.8 ($SiCH_3$).

***R*f** = 0.43 (CH_2Cl_2/MeOH 10:1, brown-orange color upon treatment with 5% methanolic H_3PO_4 and heating).

HRMS (ESI, m/z): calcd. for $[C_{19}H_{36}O_8SiNa]^+$, 443.2072; found, 443.2074

Compound **3s** (methyl 6-*O*-methyldiisopropylsilyl-4-(3-cholesteryloxy-3-oxopropyl)-α-D-galactopyranoside)

Synthesized according to **General procedure D** from silylated α-D-glucopyranoside **2a** and acrylate ester **SI-2**. The crude product was purified by column chromatography with gradient CH_2Cl_2/MeOH 25:1→15:1 as the eluent, resulting in the desired product **3s** as a brownish foam (31.2 mg, 61% yield).

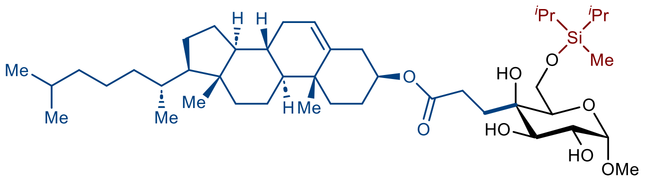

^{1}H NMR (500 MHz, $CDCl_3$) δ 5.37 (dd, *J* = 4.9, 1.9 Hz, 1H), 4.81 (d, *J* = 3.9 Hz, 1H, H-1), 4.71 – 4.55 (m, 1H), 4.05 (dd, *J* = 11.4, 3.5 Hz, 1H, H-6a), 4.01 (s, 1H, OH-4), 3.97 (dd, *J* = 11.4, 3.0 Hz, 1H, H-6b), 3.84 (td, *J* = 8.3, 7.6, 3.9 Hz, 1H, H-2), 3.61 – 3.47 (m, 2H, H-3, H-5), 3.40 (s, 3H, OCH_3), 2.64 (d, *J* = 7.2 Hz, 1H, OH-3), 2.45 (t, *J* = 7.4 Hz, 2H, CH_2), 2.34 – 2.24 (m, 3H), 2.17 (d, *J* = 8.3 Hz, 1H, OH-2), 2.12 (q, *J* = 7.8 Hz, 1H, CH_2), 2.04 – 1.91 (m, 3H), 1.89 – 1.76 (m, 3H), 1.66 – 1.40 (m, 12H), 1.39 – 1.28 (m, 4H), 1.25 (td, *J* = 9.8, 5.7 Hz, 2H), 1.19 – 1.06 (m, 10H), 1.07 – 0.96 (m, 27H), 0.91 (d, *J* = 6.5 Hz, 3H), 0.86 (d, *J* = 6.6 Hz, 6H), 0.67 (s, 3H), 0.07 (s, 3H, $SiCH_3$).

^{13}C{^{1}H} NMR (126 MHz, $CDCl_3$) δ 173.3 (C=O), 139.5, 122.7, 99.3 (C-1), 74.8 (C-4), 74.3, 72.7 (C-3 or C-5), 70.7 (C-2), 70.5 (C-3 or C-5), 63.6 (C-6), 56.7, 56.1, 55.4 (OCH_3), 50.0, 42.3, 39.7, 39.5, 38.1, 37.0, 36.6, 36.2, 35.8, 31.90, 31.85, 30.1 (CH_2), 29.6 (CH_2), 28.2, 28.0, 27.7, 24.3, 23.8, 22.8, 22.6, 21.0, 19.3, 18.7, 17.34, 17.31, 17.28, 17.25 (CH($\underline{C}H_3$)), 12.81, 12.76 ($\underline{C}H(CH_3)$), 11.9, −8.7 ($SiCH_3$).

***R*f** = 0.30 (CH_2Cl_2/MeOH 10:1, brown-red color upon treatment with 5% methanolic H_3PO_4 and heating).

HRMS (ESI, m/z): calcd. for $[C_{45}H_{82}O_8SiNa]^+$, 801.5671; found, 801.5671

Compound **3t** (methyl 6-*O*-methyldiisopropylsilyl-4-(3-(6-*O*-(1,2:3,4-di-*O*-isopropylidene-α-D-galactopyranos)yl)-3-oxopropyl)-α-D-galactopyranoside)

Synthesized according to **General procedure D** from silylated α-D-glucopyranoside **2a** and acrylate ester **SI-3**. The crude product was purified by column chromatography with gradient CH_2Cl_2/MeOH 25:1→15:1 as the eluent, resulting in the desired product **3t** as a brownish foam (22.4 mg, 53% yield).

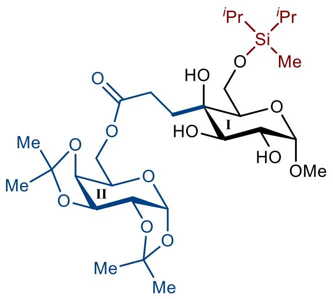

^{1}H NMR (500 MHz, $CDCl_3$) δ 5.54 (d, *J* = 5.0 Hz, 1H, H-1^{II}), 4.80 (d, *J* = 3.9 Hz, 1H, H-1^{I}), 4.62 (dd, *J* = 7.9, 2.5 Hz, 1H, H-3^{II}), 4.33 (dd, *J* = 5.0, 2.5 Hz, 1H, H-2^{II}), 4.27 (dd, *J* = 11.5, 4.9 Hz, 1H, H-6a^{II}), 4.25 – 4.18 (m, 2H, H-4^{II}, H-6b^{II}), 4.06 (ddd, *J* = 7.3, 4.9, 1.9 Hz, 1H, H-5^{II}), 4.03 – 3.93 (m, 2H, H-6^{I}), 3.83 – 3.77 (m, 1H, H-2^{I}), 3.58 – 3.52 (m, 2H, H-3^{I}, H-5^{I}), 3.40 (s, 3H, OCH_3), 2.90 (d, *J* = 6.8 Hz, 1H, OH-3^{I}), 2.58 (dt, *J* = 16.9, 7.3 Hz, 1H, CH_2), 2.47 (dt, *J* = 16.9, 7.3 Hz, 1H, CH_2), 2.35 (d, *J* = 8.6 Hz, 1H, OH-2^{I}), 2.13 (dt, *J* = 14.6, 7.3 Hz, 1H, CH_2), 1.96 (dt, *J* = 14.6, 7.3 Hz, 1H, CH_2), 1.52, 1.44, 1.33, 1.32 (all s, 12H, $C(CH_3)_2$), 1.03 – 0.96 (m, 14H, $SiCH(CH_3)_2$), 0.06 (s, 3H, $SiCH_3$).

^{13}C{^{1}H} NMR (126 MHz, $CDCl_3$) δ 173.7 (C=O), 109.7 (C(CH$_3$)$_2$), 108.9 (C(CH$_3$)$_2$), 99.3 (C-1^{I}), 96.2 (C-1^{II}), 74.7 (C-4^{I}), 72.4 (C-3^{I}), 70.92 (C-5^{I} or C-4^{II}), 70.91 (C-5^{I} or C-4^{II}), 70.63 (C-2^{I} or C-3^{II}), 70.60 (C-2^{I} or C-3^{II}), 70.4 (C-2^{II}), 65.8 (C-5^{II}), 63.5 (C-6^{II}), 63.4 (C-6^{I}), 55.3 (OCH$_3$), 30.1 (CH$_2$), 29.2 (CH$_2$), 25.9 (2 × C(CH$_3$)$_2$), 24.9 (C(CH$_3$)$_2$), 24.5 (C(CH$_3$)$_2$), 17.32, 17.28, 17.26, 17.24 (CH(CH$_3$)$_2$), 12.80, 12.76 (CH(CH$_3$)$_2$), −8.7 (SiCH$_3$).

***R*$_f$** = 0.45 (CH_2Cl_2/MeOH 10:1, brown-orange color upon treatment with 5% methanolic H_3PO_4 and heating).

HRMS (ESI, m/z): calcd. for [$C_{29}H_{52}O_{13}SiNa$]$^+$, 659.3069; found, 659.3072

Compound **3u** (methyl 6-*O*-methyldiisopropylsilyl-4-(3-(3-*O*-(1,2:5,6-di-*O*-isopropylidene-α-D-glucofuranos)yl)-3-oxopropyl)-α-D-galactopyranoside)

Synthesized according to **General procedure D** from silylated α-D-glucopyranoside **2a** and acrylate ester **SI-4**. The crude product was purified by column chromatography with gradient CH_2Cl_2/MeOH 25:1→15:1 as the eluent, resulting in the desired product **3u** as a brownish foam (14 mg, 33% yield).

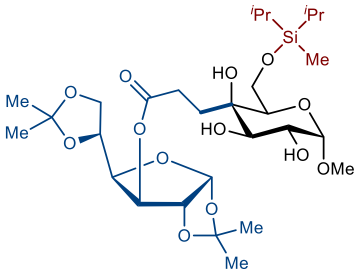

^{1}H NMR (500 MHz, $CDCl_3$) δ 5.87 (d, *J* = 3.6 Hz, 1H, H-1 Glu), 5.32 (d, *J* = 3.0 Hz, 1H, H-3 Glu), 4.80 (d, *J* = 4.0 Hz, 1H, H-1 Gal), 4.47 (d, *J* = 3.6 Hz, 1H, H-2 Glu), 4.26 (ddd, *J* = 8.5, 5.6, 4.5 Hz, 1H, H-5 Glu), 4.17 (dd, *J* = 8.5, 3.1 Hz, 1H, H-4 Glu), 4.11 – 4.05 (m, 3H, H-3 Gal, H-6a Glu), 3.97 (dd, *J* = 11.4, 2.8 Hz, 1H, H-6b Glu), 3.83 – 3.73 (m, 1H, H-2 Gal), 3.61 – 3.47 (m, 2H, H-3 Gal, H-5 Gal), 3.41 (s, 3H, OCH$_3$), 2.94 (d, *J* = 6.1 Hz, 1H, OH-3 Gal), 2.77 – 2.65 (m, 1H, CH$_2$), 2.52 – 2.39 (m, 1H, CH$_2$), 2.09 – 1.95 (m, 3H, CH$_2$, OH-2), 1.52 (s, 3H (C(CH$_3$)$_2$), 1.41 (s, 3H, C(CH$_3$)$_2$), 1.34 (s, 3H, C(CH$_3$)$_2$), 1.30 (s, 3H, C(CH$_3$)$_2$), 1.10 – 0.86 (m, 14H, SiCH(CH$_3$)$_2$), 0.07 (s, 3H, SiCH$_3$).

^{13}C{^{1}H} NMR (126 MHz, $CDCl_3$) δ 172.6 (C=O), 112.3 (C(CH$_3$)$_2$), 109.6 (C(CH$_3$)$_2$), 105.1 (C-1 Glu), 99.3 (C-1 Gal), 83.4 (C-2 Glu), 79.7 (C-4 Glu), 75.7 (C-3 Glu), 74.4 (C-4 Gal), 73.2 (C-3 Gal or C-5 Gal), 72.3 (C-5 Glu), 71.0 (C-3 Gal or C-5 Gal), 70.8 (C-2 Gal), 67.2 (C-6 Glu), 63.7 (C-6 Gal), 55.4 (OCH$_3$), 30.7 (CH$_2$), 29.3 (CH$_2$), 26.9 (C(CH$_3$)$_2$), 26.7 (C(CH$_3$)$_2$), 26.2 (C(CH$_3$)$_2$), 25.1 (C(CH$_3$)$_2$), 17.28, 17.26, 17.25, 17.23 (CH(CH$_3$)$_2$), 12.81, 12.76 (CH(CH$_3$)$_2$), −8.7 (SiCH$_3$).

***R*$_f$** = 0.33 (CH_2Cl_2/MeOH 10:1, brown-orange color upon treatment with 5% methanolic H_3PO_4 and heating).

HRMS (ESI, m/z): calcd. for [$C_{29}H_{52}O_{13}SiNa$]$^+$, 659.3069; found, 659.3072

Compound **3v** (2,3:4,5-di-*O*-isopropylidene-3-(3-methoxy-3-oxopropyl)-1-*O*-methyldiisopropylsilyl-β-D-fructopyranose)

Synthesized according to **General procedure D** from silylated β-D-fructose **2l** and methyl acrylate. The crude product was purified by column chromatography with hexane/EtOAc 10:1 as the eluent, resulting in the desired product **3v** as a yellow oil (6.7 mg, 22% yield).

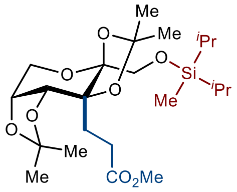

^{1}H NMR (500 MHz, $CDCl_3$) δ 4.31 (d, *J* = 7.6 Hz, 1H, H-4), 4.19 (ddd, *J* = 7.6, 2.8, 1.2 Hz, 1H, H-5), 3.95 (ABq(dd), *J* = 13.0, 2.7 Hz, 1H, H-6a), 3.85 – 3.74 (m, 2H, 2H-1), 3.69 (dd, *J* = 13.0, 1.2 Hz, 1H, H-6b), 3.67 (s, 3H, CO$_2$CH$_3$), 2.68 (ddd, *J* = 14.4, 11.6, 4.4 Hz, 1H, CH$_2$), 2.59 – 2.43 (m, 2H, CH$_2$), 2.30 – 2.19 (m, 1H, CH$_2$), 1.53 (s, 3H),

1.50 (s, 3H), 1.43 (s, 3H), 1.31 (s, 3H, $C(CH_3)_2$), 1.05 – 0.96 (m, 14H, $SiCH(CH_3)_2$), 0.05 (s, 3H, $SiCH_3$).

$^{13}C\{^1H\}$ NMR (126 MHz, $CDCl_3$) δ 174.1 (C=O), 108.7 ($\underline{C}(CH_3)_2$), 108.4 ($\underline{C}(CH_3)_2$), 105.2 (C-2), 80.4 (C-3), 71.7 (C-4), 70.8 (C-5), 65.1 (C-1), 61.5 (C-6), 51.6 ($CO_2\underline{C}H_3$), 29.2 ($C(\underline{C}H_3)_2$), 28.2 (CH_2), 28.0 (CH_2), 27.9 ($C(\underline{C}H_3)_2$), 26.3 ($C(\underline{C}H_3)_2$), 24.4 ($C(\underline{C}H_3)_2$), 17.44, 17.37, 17.35 ($CH(\underline{C}H_3)_2$), 12.95, 12.88 ($\underline{C}H(CH_3)_2$), −8.6 ($SiCH_3$).

***R*$_f$** = 0.28 (hexane/EtOAc 10:1, green color upon treatment with 5% methanolic H_3PO_4 and heating).

HRMS (ESI, m/z): calcd. for $[C_{23}H_{42}O_8SiNa]^+$, 497.2541; found, 497.2541

Compounds **3w/3w'** (1,2-*O*-isopropylidene-3-(3-methoxy-3-oxopropyl)-5-*O*-methyldiisopropylsilyl-α-D-xylo/ribofuranose)

Synthesized according to **General procedure D** from silylated D-xylofuranose **2m** and methyl acrylate. The crude product was purified by column chromatography with hexane/EtOAc 3:1 as the eluent, resulting in the desired product **3w** (Xyl) (13.3 mg, 33% yield) as yellow oil. The remaining chromatographic fractions were concentrated and purified by reversed-phase (C18) column chromatography with MeCN/water 1:1 as the eluent, resulting in the desired product **3w'** (Rib) (7.8 mg, 23% yield) as a yellow oil.

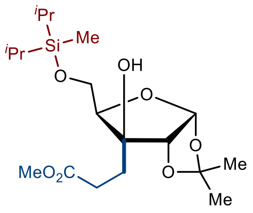

Analytical data for compound **3w**:

^1H NMR (500 MHz, $CDCl_3$) δ 5.93 (d, *J* = 4.1 Hz, 1H, H-1), 4.56 (d, *J* = 4.1, Hz, 1H, H-2), 4.14 (br.d, *J* = 5.3 Hz, 1H, H-4), 3.75 (d, *J* = 10.4 Hz, 1H, H-5a), 3.69 (d, *J* = 10.4 Hz, 1H, H-5b), 3.67 (s, 3H, CO_2CH_3), 3.64 (d, *J* = 6.1 Hz, 1H, OH), 2.56 (ddd, *J* = 16.2, 11.4, 5.0 Hz, 1H, CH_2), 2.34 (ddd, *J* = 16.2, 11.5, 5.2 Hz, 1H, CH_2), 2.16 (ddd, *J* = 14.3, 11.5, 5.0 Hz, 1H, CH_2), 1.92 (ddd, *J* = 14.3, 11.4, 5.2 Hz, 1H, CH_2), 1.53 (s, 3H, $C(CH_3)_2$), 1.31 (s, 3H, $C(CH_3)_2$), 1.01, 1.00, 0.99, 0.98 (all s, 14H, $CH(CH_3)_2$), 0.07 (s, 3H, $SiCH_3$).

$^{13}C\{^1H\}$ NMR (126 MHz, $CDCl_3$) δ 173.8 (CO_2CH_3), 112.5 ($\underline{C}(CH_3)_2$), 105.3 (C-1), 88.6 (C-3), 88.1 (C-2), 81.3 (C-4), 65.7 (C-5), 51.6 ($CO_2\underline{C}H_3$), 29.7 (CH_2), 28.5 (CH_2), 26.8 ($C(\underline{C}H_3)_2$), 26.3 ($C(\underline{C}H_3)_2$), 17.31, 17.29, 17.25, 17.23 ($CH(\underline{C}H_3)_2$), 12.8, 12.7 ($\underline{C}H(CH_3)_2$), −8.7 ($SiCH_3$).

***R*$_f$** = 0.32 (hexane/EtOAc 3:1, green color upon treatment with 5% methanolic H_3PO_4 and heating).

HRMS (ESI, m/z): calcd. for $[C_{19}H_{36}O_7SiNa]^+$, 427.2123; found, 427.2126

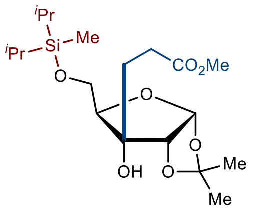

Analytical data for compound **3w'**:

^1H NMR (500 MHz, $CDCl_3$) δ 5.83 (d, *J* = 4.3 Hz, 1H, H-1), 4.61 (dd, *J* = 4.3, 1.1 Hz, 1H, H-2), 4.32 (br.s, 1H, H-4), 3.79 (d, *J* = 9.9 Hz, 1H, H-5a), 3.67 (s, 3H, CO_2CH_3), 3.64 (d, *J* = 9.9 Hz, 1H, H-5b), 2.67 (d, *J* = 4.1 Hz, 1H, OH-3), 2.54 – 2.48 (m, 2H, CH_2), 2.10 – 1.94 (m, 2H, CH_2), 1.53 (s, 3H, $C(CH_3)_2$), 1.30 (s, 3H, $C(CH_3)_2$), 1.03 – 0.97 (m, 14H, $SiCH(CH_3)_2$), 0.03 (s, 3H, $SiCH_3$).

$^{13}C\{^1H\}$ NMR (126 MHz, $CDCl_3$) δ 175.1 ($\underline{C}O_2CH_3$), 112.0 ($\underline{C}(CH_3)_2$), 104.5 (C-1), 89.5 (C-3), 87.8 (C-2), 77.6 (C-4), 65.3 (C-5), 51.7 ($CO_2\underline{C}H_3$), 28.5 (CH_2), 26.7 ($C(\underline{C}H_3)_2$), 26.1 ($C(\underline{C}H_3)_2$), 25.9 (CH_2), 17.39, 17.38, 17.34, 17.31 ($CH(\underline{C}H_3)_2$), 12.87, 12.86 ($\underline{C}H(CH_3)_2$), −8.6 ($SiCH_3$).

***R*$_f$** = 0.25 (hexane/EtOAc 3:1, green color upon treatment with 5% methanolic H_3PO_4 and heating).

HRMS (ESI, m/z): calcd. for $[C_{19}H_{36}O_7SiNa]^+$, 427.2123; found, 427.2125

Compound **6a** ((5*R*,6*R*,8*S*,9*R*,10*R*)-6-(((diisopropyl(methyl)silyl)oxy)methyl)-9,10-dihydroxy-8-methoxy-1,7-dioxaspiro[4.5]decan-2-one)

Synthesized according to the procedure described in **Section 4.3.1**.

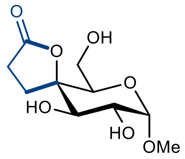

^{1}H NMR (500 MHz, $CDCl_3$) δ 4.76 (d, *J* = 3.1 Hz, 1H, H-1), 3.79 – 3.72 (m, 2H), 3.73 – 3.64 (m, 2H), 3.63 (dd, *J* = 11.3, 6.3 Hz, 1H, H-6b), 3.43 (s, 3H, OCH_3), 2.75 – 2.61 (m, 1H, CH_2), 2.60 – 2.49 (m, 1H, CH_2), 2.28 (t, *J* = 8.8 Hz, 2H, CH_2).

^{13}C{^{1}H} NMR (126 MHz, $CDCl_3$) δ 179.8 (C=O), 101.2 (C-1), 88.9 (C-4), 74.3, 74.2, 71.0, 61.4 (C-6), 55.8 (OCH_3), 29.3 (CH_2), 26.2 (CH_2).

***R*$_f$** = 0.44 (CH_2Cl_2/MeOH 5:1, brown color upon treatment with 5% methanolic H_3PO_4 and heating).

HRMS (ESI, m/z): calcd. for $[C_{10}H_{16}O_7Na]^+$, 271.0788; found, 271.0790

Compound **7a** (methyl 6-*O*-methyldiisopropylsilyl-4-(2-carboxyethyl)-α-D-galactopyranoside)

Synthesized according to the procedure described in **Section 4.3.2**.

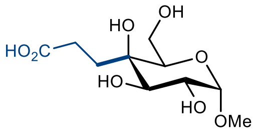

^{1}H NMR (500 MHz, $CDCl_3$) δ 4.71 (d, *J* = 3.9 Hz, 1H, H-1), 3.87 (dd, *J* = 12.0, 2.7 Hz, 1H, H-6a), 3.81 – 3.73 (m, 2H, H-2, H-6b), 3.71 – 3.65 (m, 1H, H-5), 3.65 (d, *J* = 9.9 Hz, 1H, H-3), 3.41 (s, 3H, OCH_3), 2.27 – 2.10 (m, 3H, CH_2), 1.83 – 1.71 (m, 1H, CH_2), 1.43 – 1.29 (m, 3H, OH).

^{13}C{^{1}H} NMR (126 MHz, $CDCl_3$) δ 182.1 (C=O), 101.1 (C-1), 76.4 (C-4), 73.1 (C-5), 71.2 (C-3), 70.9 (C-2, 61.9 (C-6), 55.5 (OCH_3), 33.6 (CH_2), 32.1 (CH_2).

***R*$_f$** = 0.83 (CH_2Cl_2/MeOH 2:1, brown color upon treatment with 5% methanolic H_3PO_4 and heating).

HRMS (ESI, m/z): calcd. for $[C_{10}H_{18}O_8Na]^+$, 289.0894; found, 289.0895

5. Mechanistic studies

5.1 Fluorescence quenching studies

Fluorescence quenching measurements were performed on FS5 steady-state spectrofluorometer (Edinburgh Instruments) under Ar at room temperature (ca. 20 °C), using 10 x 10 mm quartz cuvettes. Fluorescence spectra were recorded on series of solutions in MeCN/^{t}BuOH 9:1, containing the photocatalyst (**PC1**, [Ir(dF(CF_3)ppy)$_2$(dtbbpy)](PF_6), 15 μM) and one of the quenchers (0–10 mM): ^{i}Pr$_2$NEt, substrate **2a**, and methyl acrylate (Figure S5). The Stern-Volmer quenching constants (K_{SV}) were calculated as the slope of linear regression in the plot of normalized emission intensity (I_0/I) at 475 nm over concentration of the quencher (Figure 5, *top right*).

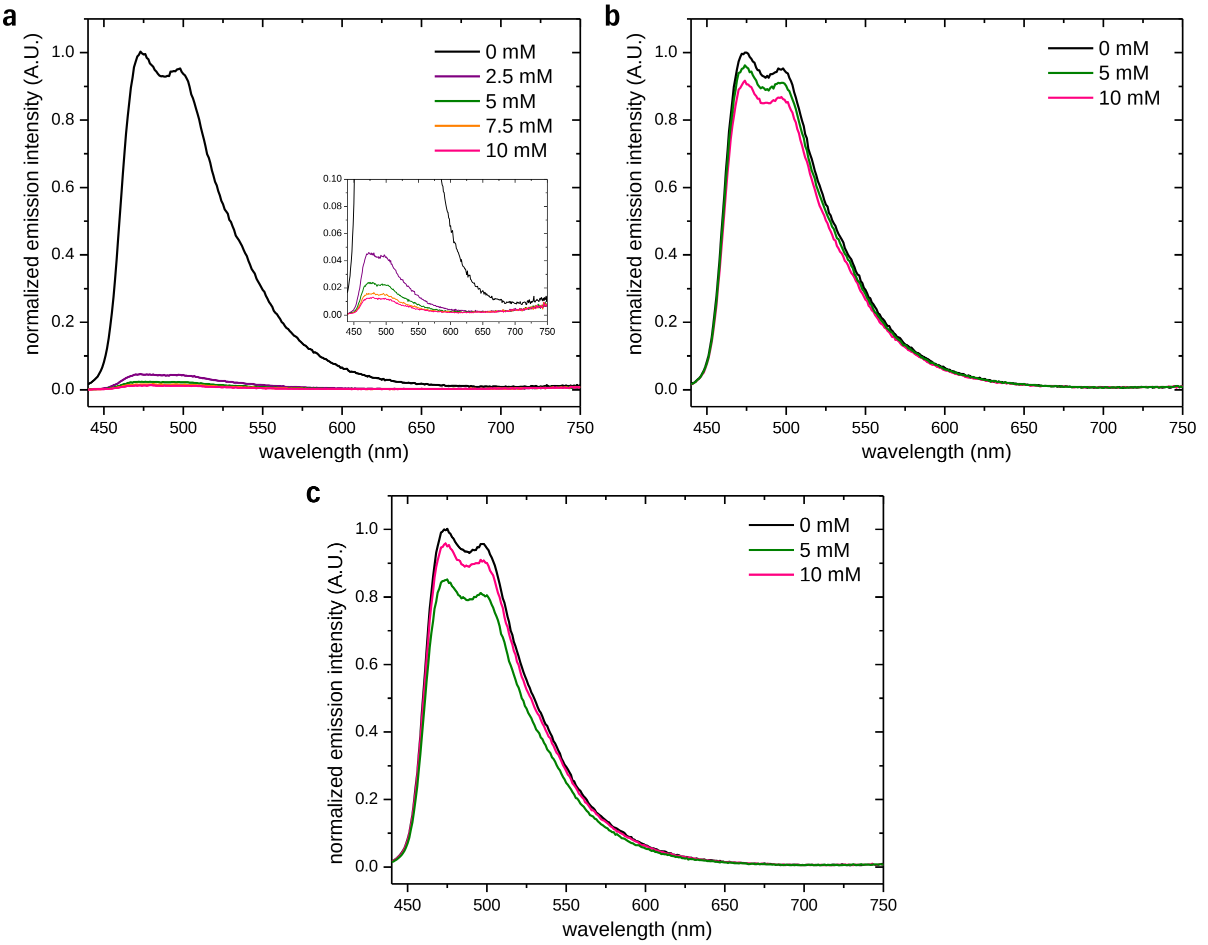

Figure S5. Fluorescence spectra of photocatalyst **PC1** (15 μM) with ^{i}Pr$_2$NEt (**a**), substrate **2a** (**b**), and methyl acrylate (**c**) as the quencher.

5.2 Intramolecular vs. intermolecular hydrogen atom transfer control experiment

Control experiment demonstrating the absence of intermolecular HAT during formation of the C-functionalized carbohydrate products was performed with silylated diacetone α-D-galactose substrate **2e** and deactivated silylated α-D-glucoside substrate **4a**, subjected to the standard photochemical reaction conditions (**General procedure D**). The reaction mixture was terminated after 1 h of irradiation and analyzed by ^{1}H NMR after filtering through a silica plug and concentration on a rotary evaporator (Figure S6). ^{1}H NMR of the reaction mixture demonstrated formation of the expected C-functionalized product **3e** from the intramolecular HAT, while formation of the potential C-functionalized product **3a** from the intermolecular HAT was not observed.

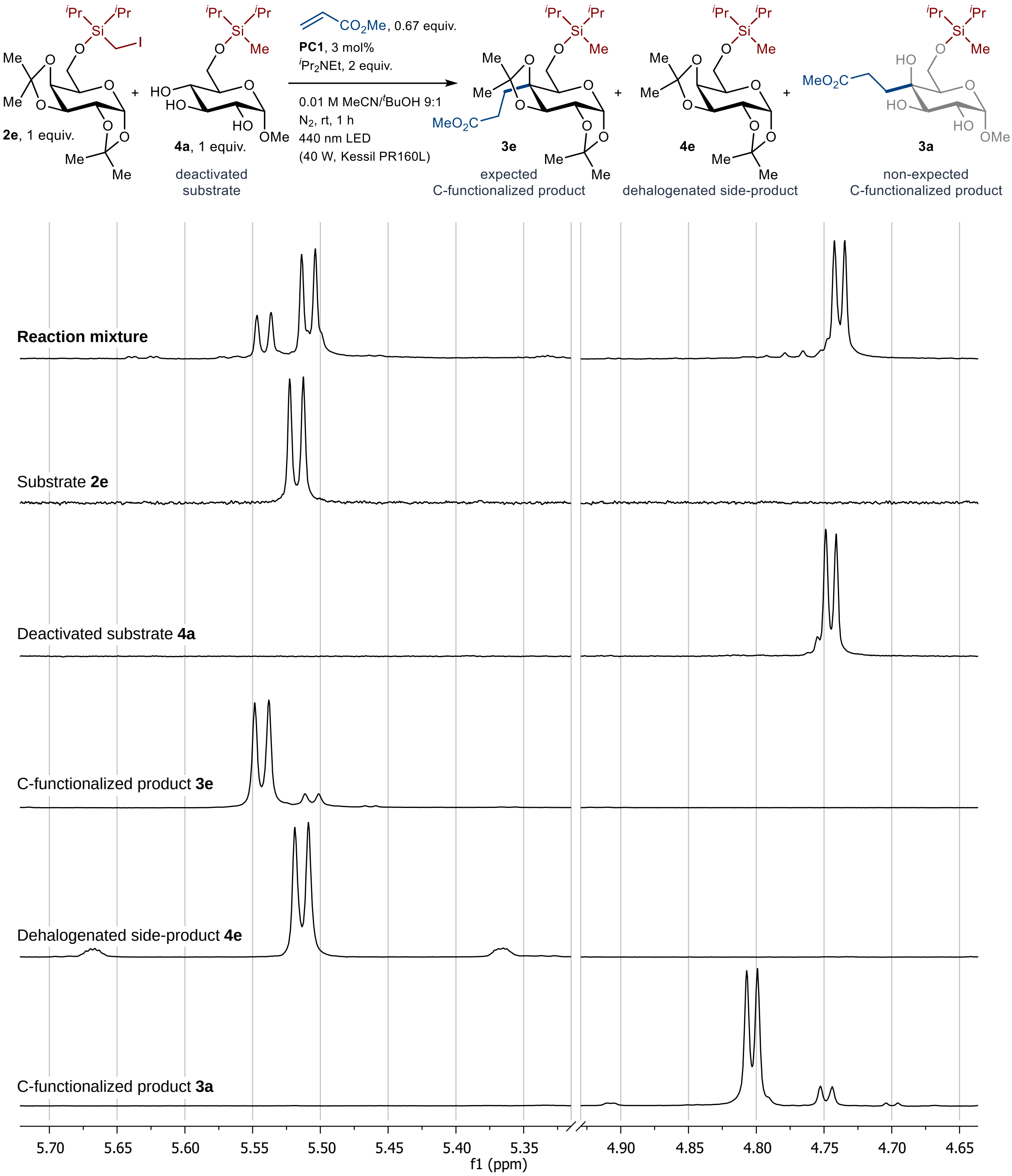

Figure S6. Reaction scheme and ^{1}H NMR spectra for the intra- vs. intermolecular hydrogen atom transfer control experiment with substrate **2e** and deactivated substrate **4a**.

5.3 Computational studies

5.3.1 Computational methods and energies of the calculated species and transition states

All stationary points were optimized at the B3LYP/6-311+G(d,p) level of theory, as implemented in Gaussian 16 Rev D.01 software. During the optimization, the Grimme correction for dispersion (D3) was used in combination with the Conductor-like Polarizable Continuum Model (CPCM), using the parameters for acetonitrile and the default Unified Force Field radii (UFF). All geometries were characterized as minima or saddle points on the potential-energy surface (PES) by using the sign of the eigenvalues of the force constant matrix obtained from the frequency calculation. Transition states with one imaginary frequency were confirmed to describe the correct movement on the PES by the mode analysis and by the intrinsic reaction coordinate (IRC) calculations connecting the correct reactants and products. The evaluated reactions and energy diagrams for α- and β-glycoside substrates are outlined in Figures S7 and S8, respectively. The corresponding calculated energies, enthalpies, and Gibbs free energies are summarized in Table S3.

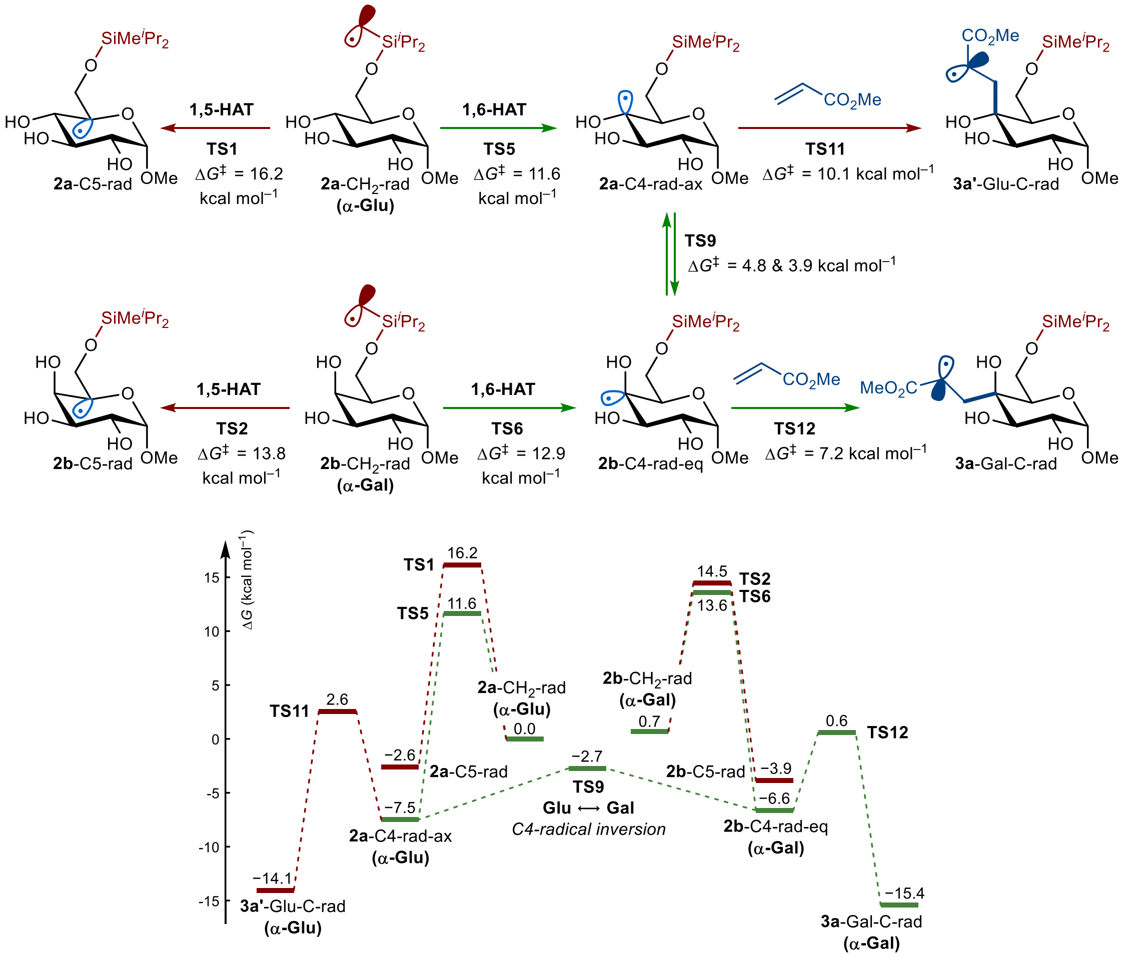

Figure S7. Reaction scheme and calculated energy diagram for the key mechanistic steps in C-functionalization of α-glycoside substrates **2a** and **2b**.

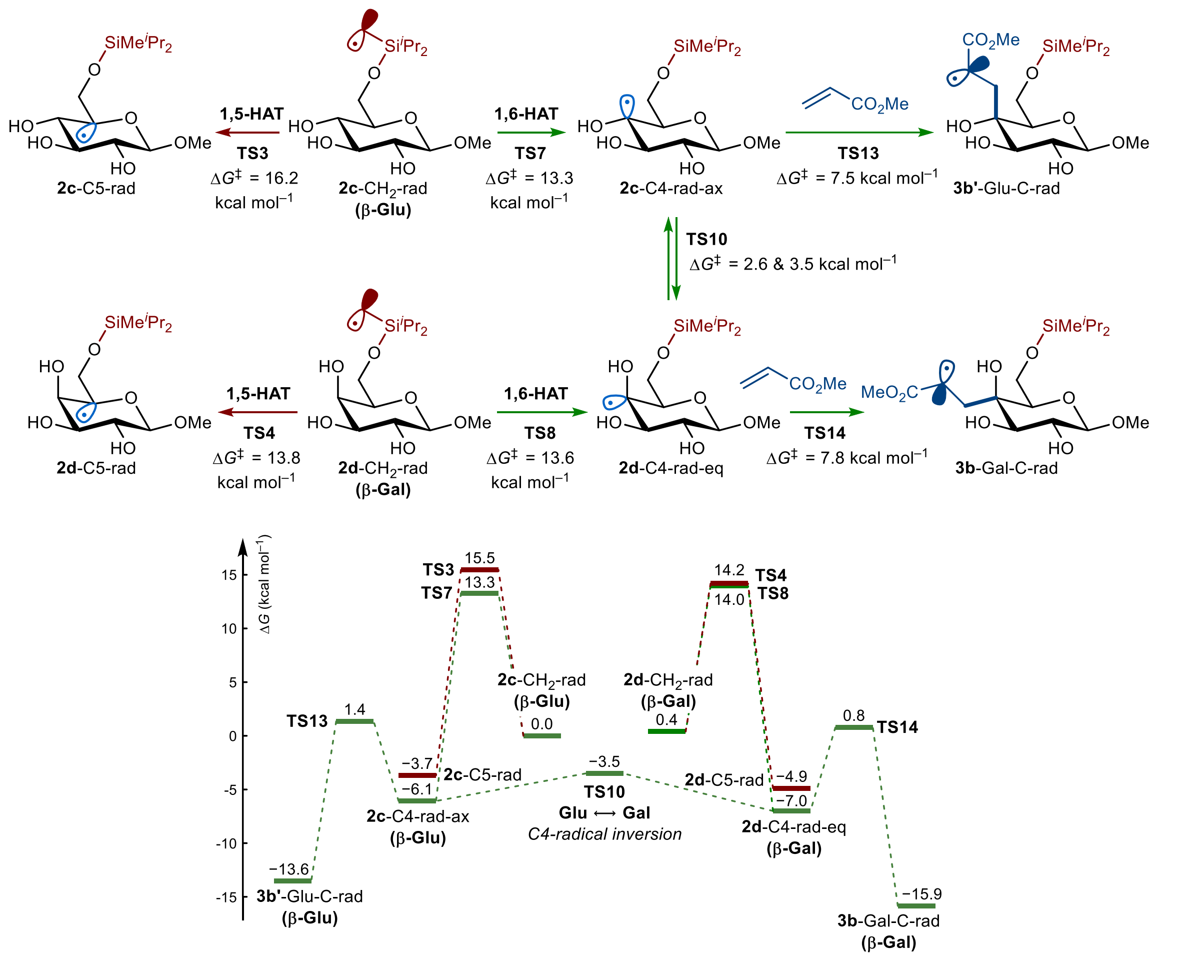

Figure S8. Reaction scheme and calculated energy diagram for the key mechanistic steps in C-functionalization of β-glycoside substrates **2c** and **2d**.

Table S3. Calculated energies, enthalpies, and Gibbs free energies for the intermediates and transition states from Figures S7 and S8.

species / transition states	energy	enthalpy	Gibbs free energy	ΔE	ΔH	ΔG
		(Hartree)			(kcal mol⁻¹)	
**2a**-CH$_2$-rad	-1292.166839	-1291.713187	-1291.800977	0	0	0
**TS1**	-1292.141991	-1291.693798	-1291.775213	15.59	12.17	16.17
**2a**-C5-rad	-1292.173691	-1291.7201	-1291.805133	-4.30	-4.34	-2.61
**2b**-CH$_2$-rad	-1292.169196	-1291.715346	-1291.799899	-1.48	-1.35	0.68
**TS2**	-1292.144181	-1291.695805	-1291.777926	14.22	10.91	14.46
**2b**-C5-rad	-1292.176347	-1291.722326	-1291.807141	-5.97	-5.73	-3.87
**2c**-CH$_2$-rad	-1292.16546	-1291.711967	-1291.799332	0	0	0
**TS3**	-1292.140058	-1291.692162	-1291.774677	15.94	12.43	15.47
**2c**-C5-rad	-1292.172052	-1291.718869	-1291.805231	-4.14	-4.33	-3.70
**2d**-CH$_2$-rad	-1292.167949	-1291.714331	-1291.798702	-1.56	-1.48	0.40
**TS4**	-1292.141171	-1291.693173	-1291.776714	15.24	11.79	14.19

species / transition states	energy	enthalpy	Gibbs free energy	ΔE	ΔH	ΔG
		(Hartree)			(kcal mol⁻¹)	
**2d**-C5-rad	-1292.176347	-1291.722326	-1291.807141	-6.83	-6.50	-4.90
**TS5**	-1292.14825	-1291.700115	-1291.782445	11.66	8.20	11.63
**2a**-C4-rad-ax	-1292.178147	-1291.724493	-1291.812906	-7.10	-7.09	-7.49
**TS6**	-1292.146453	-1291.697908	-1291.779286	12.79	9.59	13.61
**2b**-C4-rad-eq	-1292.18116	-1291.727118	-1291.811547	-8.99	-8.74	-6.63
**TS7**	-1292.142853	-1291.695197	-1291.77813	14.19	10.52	13.30
**2c**-C4-rad-ax	-1292.175678	-1291.722038	-1291.809025	-6.41	-6.32	-6.08
**TS8**	-1292.143025	-1291.694862	-1291.777075	14.08	10.73	13.97
**2d**-C4-rad-eq	-1292.179329	-1291.725462	-1291.810505	-8.70	-8.47	-7.01
**TS9**	-1292.171924	-1291.71953	-1291.805324	-3.19	-3.98	-2.73
**TS10**	-1292.173392	-1291.72112	-1291.804914	-4.98	-5.74	-3.50
**TS11**	-1598.765496	-1598.207986	-1598.308287	-14.51	-13.53	2.56
**3a'**-Glu-C-rad	-1598.795271	-1598.23502	-1598.334778	-33.19	-30.50	-14.06
**TS12**	-1598.767694	-1598.210142	-1598.31144	-15.89	-14.88	0.58
**3a**-Gal-C-rad	-1598.797401	-1598.237237	-1598.336974	-34.53	-31.89	-15.44
**TS13**	-1598.764196	-1598.207066	-1598.308572	-14.56	-13.72	1.35
**3b'**-Glu-C-rad	-1598.791838	-1598.232077	-1598.332321	-31.90	-29.41	-13.55
**TS14**	-1598.765911	-1598.208584	-1598.309485	-15.64	-14.67	0.78
**3b**-Gal-C-rad	-1598.793163	-1598.233679	-1598.335991	-32.74	-30.42	-15.86

5.3.2 Cartesian coordinates

2a-CH_2-rad

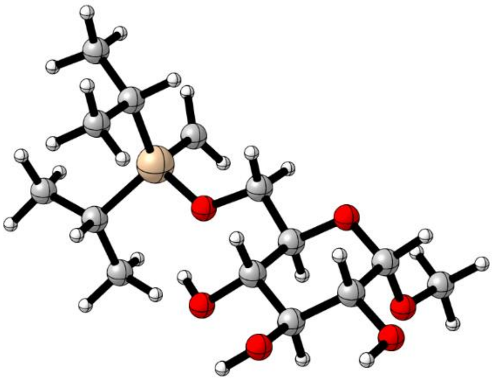

C	0.951785	-0.867602	1.405453
C	-0.416434	-1.293687	1.944840
C	-1.323771	-1.711449	0.793949
C	-1.417889	-0.596996	-0.242395
C	-0.011533	-0.144155	-0.671680
O	0.777605	0.198390	0.478347
O	-0.245724	-2.334721	2.898372
O	-2.601452	-2.014261	1.363112
O	-2.170540	-1.108078	-1.341898
C	-0.034853	1.116983	-1.528255
O	-0.846803	0.868975	-2.675297
H	-1.127223	-2.638801	3.147971

H	-3.170267	-2.346344	0.658753	H	-1.038104	5.011070	-4.527259
Si	-1.122065	1.988157	-3.923393	H	-2.701636	4.559219	-4.910175
H	-0.852739	-0.409274	2.426771	C	-2.740717	1.807699	-6.294294
H	-0.906800	-2.604664	0.315144	H	-3.394979	1.194026	-6.923427
H	-1.939584	0.258169	0.211490	H	-3.300251	2.704946	-6.019012
H	0.463799	-0.958645	-1.229869	H	-1.891547	2.119181	-6.911637
H	-2.036778	-0.483559	-2.074716	C	-1.635900	-0.328646	-5.487076
H	-0.444448	1.941700	-0.932862	H	-2.343008	-0.922493	-6.076976
H	0.988502	1.375924	-1.820413	H	-0.749821	-0.161464	-6.108629
C	-1.900548	3.531142	-3.145291	H	-1.331081	-0.927660	-4.625297
H	-1.206894	3.806858	-2.338405	C	0.498211	2.392698	-4.723258
C	-2.275822	1.007716	-5.063894	H	0.761782	3.384602	-5.078838
H	-3.158538	0.782434	-4.449914	H	1.257223	1.635570	-4.904481
C	-3.261781	3.204393	-2.501470	O	1.581134	-1.975189	0.826000
H	-3.647900	4.069804	-1.951838	C	2.936036	-1.731258	0.435110
H	-4.005710	2.940587	-3.260277	H	3.326423	-2.675494	0.058243
H	-3.192720	2.368289	-1.799133	H	2.989772	-0.972655	-0.350924
C	-2.003591	4.744099	-4.088949	H	3.534516	-1.404816	1.293643
H	-2.370925	5.620559	-3.543612	H	1.568125	-0.443889	2.205950

TS1

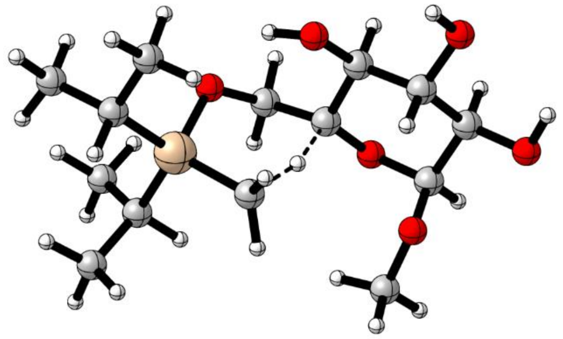

C	-3.920415	0.180997	-0.913224	H	-3.597235	-1.912288	-1.088017
C	-3.926247	-1.221403	-0.300801	H	-3.235706	-0.606269	1.643342
C	-2.927573	-1.294633	0.849189	H	-1.206538	-1.650855	-0.373911
C	-1.544079	-0.891211	0.351502	H	-1.802587	1.392101	0.599974
C	-1.604161	0.453507	-0.366224	H	0.128770	-0.385756	1.233941
O	-2.593486	0.501159	-1.357887	H	0.190957	0.152634	-1.543108
O	-5.246965	-1.543585	0.110628	H	-0.491827	1.785086	-1.618385
O	-2.948337	-2.641367	1.327921	C	0.302233	4.215820	-0.056787
O	-0.668418	-0.879451	1.476234	H	-0.628513	4.174059	-0.640316
C	-0.286243	0.940832	-0.950032	C	1.455641	2.646285	2.484689
O	0.621402	1.306773	0.096051	H	1.117889	3.449823	3.154050
H	-5.201368	-2.387512	0.577207	C	1.474228	4.225035	-1.054745
H	-2.381535	-2.692680	2.106345	H	1.394726	5.083358	-1.731445
Si	0.224921	2.676751	1.049591	H	2.438229	4.301052	-0.544629

H	1.497676	3.319101	-1.666182
C	0.262008	5.512189	0.776557
H	0.191550	6.389645	0.124291
H	-0.594473	5.538456	1.457580
H	1.168723	5.625720	1.379419
C	1.388490	1.321410	3.269152
H	2.006722	1.378823	4.171645
H	0.371171	1.068489	3.578917
H	1.773156	0.491485	2.667778
C	2.906400	2.954127	2.066954
H	3.571010	2.913277	2.937364
H	3.274143	2.224124	1.339026
H	3.007487	3.947820	1.624772
C	-1.545465	2.371647	1.544777
H	-1.750427	1.845605	2.475830
H	-2.286659	3.141530	1.335660
O	-4.382041	1.096779	0.029377
C	-4.584166	2.415501	-0.489893
H	-4.987350	3.012134	0.326759
H	-3.641896	2.852187	-0.830862
H	-5.299530	2.398520	-1.320065
H	-4.525071	0.211100	-1.825399

2a-C5-rad

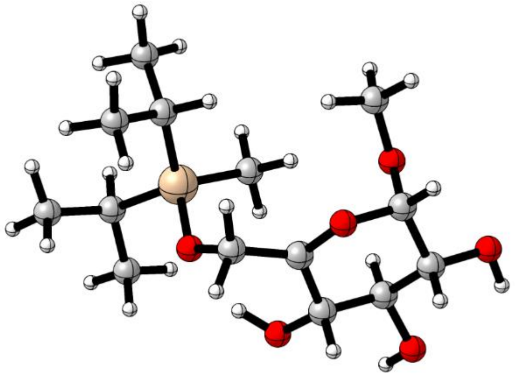

C	-2.521470	-0.935954	-1.540397
C	-2.719496	-2.285932	-0.849070
C	-1.727659	-2.435418	0.299279
C	-0.301687	-2.303196	-0.242742
C	-0.178576	-1.061128	-1.076975
O	-1.159731	-0.828992	-2.011239
O	-4.068810	-2.387582	-0.418466
O	-1.956691	-3.720343	0.879649
O	0.625288	-2.351929	0.844860
C	1.142931	-0.444912	-1.383965
O	1.809015	-0.021318	-0.175771
H	-4.135565	-3.188876	0.116114
H	-1.412265	-3.793075	1.672261
Si	1.423844	1.393268	0.695638
H	-2.502863	-3.060804	-1.596659
H	-1.900709	-1.647877	1.039502
H	-0.112317	-3.186893	-0.879639
H	-1.033759	0.994729	0.864281
H	1.221212	-1.587969	0.773229
H	1.827254	-1.158826	-1.862374
H	0.991072	0.386915	-2.077935
C	1.292839	2.843522	-0.521659
H	0.361892	2.650010	-1.073849
C	2.811330	1.516877	1.978914
H	2.490748	2.310383	2.668631
C	2.433251	2.938888	-1.552794
H	2.203899	3.701684	-2.305268
H	3.376468	3.222806	-1.080206
H	2.604029	1.996096	-2.078589
C	1.112506	4.182984	0.221599
H	0.947271	4.999639	-0.489801
H	0.259601	4.166011	0.906175
H	2.003756	4.435215	0.805717
C	2.926230	0.209017	2.789383
H	3.672790	0.314030	3.584406
H	1.981738	-0.074613	3.261921
H	3.243230	-0.620630	2.149570
C	4.182339	1.914367	1.402266
H	4.942514	1.923235	2.191820
H	4.513848	1.206037	0.636364
H	4.166701	2.909990	0.953271
C	-0.223284	1.170865	1.574517

H	-0.191038	0.317146	2.256562	H	-3.159454	2.085475	-0.467022
H	-0.465806	2.061582	2.162791	H	-1.894159	1.673147	-1.652160
O	-2.826236	0.090616	-0.653587	H	-3.616025	1.418761	-2.058505
C	-2.872438	1.390703	-1.254455	H	-3.123094	-0.868972	-2.451786

2b-CH_2-rad

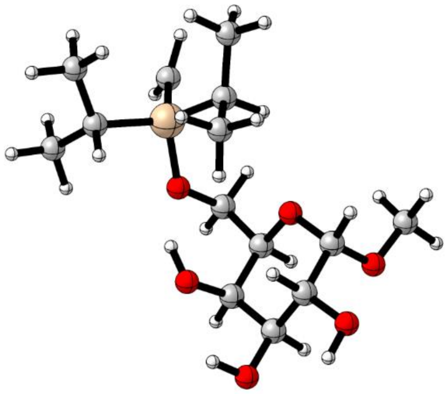

C	0.736150	0.142334	1.255051	H	-1.729718	2.353010	0.819145
C	-0.394853	-0.610026	1.957938	C	-0.429964	5.172267	-0.803362
C	-0.942205	-1.718581	1.053973	H	0.184987	5.499268	0.042978
C	-1.383690	-1.125983	-0.287822	H	0.140810	5.363976	-1.716074
C	-0.236245	-0.298033	-0.890689	H	-1.315292	5.814515	-0.824897
O	0.254749	0.683863	0.034994	C	-3.955463	4.833112	-2.014490
O	0.074808	-1.122585	3.199475	H	-5.038763	4.992983	-1.973923
O	-2.016510	-2.399865	1.707513	H	-3.517869	5.340633	-1.151298
C	-0.605552	0.434003	-2.173658	H	-3.585771	5.330218	-2.917604
O	-1.755991	1.267575	-2.000540	C	-4.394540	2.613757	-3.164792
H	-0.628870	-1.680337	3.554876	H	-5.475035	2.763132	-3.058509
H	-2.792892	-1.825933	1.644974	H	-4.103328	3.009646	-4.143567
Si	-1.774813	2.963395	-2.105468	H	-4.201863	1.537966	-3.167717
H	-1.194219	0.120977	2.126182	C	-1.016516	3.444920	-3.725195
H	-0.162014	-2.463989	0.890592	H	-0.498355	4.385476	-3.888078
H	0.571160	-0.998898	-1.139545	H	-1.095559	2.818246	-4.610181
H	0.259299	1.017473	-2.505749	O	1.813698	-0.735087	1.055840
H	-0.833155	-0.305994	-2.946695	C	2.985281	-0.097847	0.539480
C	-0.799155	3.684879	-0.650293	H	3.758428	-0.862913	0.482130
H	0.130807	3.103831	-0.642073	H	2.803889	0.315303	-0.457040
C	-3.635018	3.327507	-2.031325	H	3.316083	0.706259	1.207521
H	-3.967448	2.898192	-1.076067	H	1.050381	1.011466	1.844209
C	-1.513092	3.415730	0.688088	O	-2.567645	-0.366372	-0.027571
H	-0.883708	3.730016	1.528587	H	-2.608727	0.337694	-0.699252
H	-2.454626	3.970345	0.759447	H	-1.613509	-1.942909	-0.984982

2c-CH_2-rad

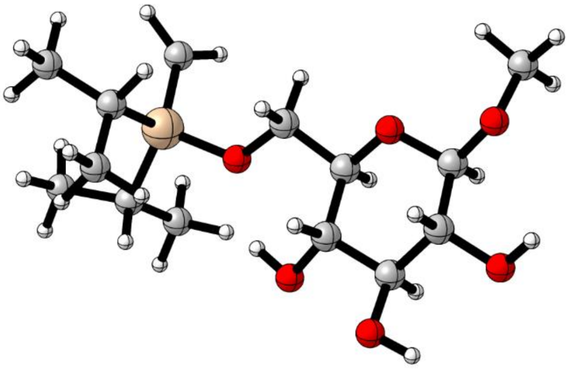

C	1.334407	-1.314531	0.786838
C	0.592003	-0.715035	1.977618
C	-0.863756	-0.439265	1.612726
C	-0.966402	0.391444	0.338884
C	-0.083196	-0.213272	-0.775262
O	1.254654	-0.412762	-0.314969
O	0.577840	-1.603308	3.092430
O	-1.523389	0.259410	2.668271
O	-2.334317	0.421604	-0.059389
C	0.045715	0.707026	-1.986402
O	-1.257794	0.999944	-2.484645
H	1.488187	-1.731477	3.384655
H	-1.396457	-0.254516	3.475404
Si	-1.585650	1.999584	-3.819223
H	1.086727	0.228591	2.239144
H	-1.364101	-1.401517	1.434161
H	-0.605753	1.407079	0.557126
H	-0.527014	-1.169256	-1.087910
H	-2.337671	0.756320	-0.972294
H	0.559667	1.624024	-1.674347
H	0.654596	0.211947	-2.750160
O	2.666193	-1.469538	1.145662
H	0.890848	-2.282501	0.499087
C	3.434575	-2.271857	0.238184
H	4.436002	-2.343683	0.659512
H	3.001283	-3.274272	0.150086
H	3.483072	-1.805050	-0.748574
C	-0.860528	3.710705	-3.446055
H	0.187127	3.511327	-3.180179
C	-3.479399	1.941409	-3.876877
H	-3.805195	2.339982	-2.906382
C	-1.541912	4.348462	-2.219894
H	-1.039085	5.280215	-1.938653
H	-2.588289	4.592285	-2.430605
H	-1.523990	3.685504	-1.349705
C	-0.847723	4.676957	-4.645515
H	-0.335101	5.607815	-4.378683
H	-0.331392	4.254347	-5.511641
H	-1.860187	4.944744	-4.960197
C	-4.082216	2.825571	-4.983575
H	-5.175042	2.747056	-4.981531
H	-3.830634	3.881095	-4.854795
H	-3.734056	2.516890	-5.974901
C	-3.988804	0.491840	-3.992229
H	-5.082973	0.464769	-3.940436
H	-3.694638	0.046166	-4.948373
H	-3.598780	-0.143573	-3.193149
C	-0.801057	1.255283	-5.322041
H	-0.786372	0.181659	-5.493239
H	-0.351223	1.841460	-6.118156

TS3

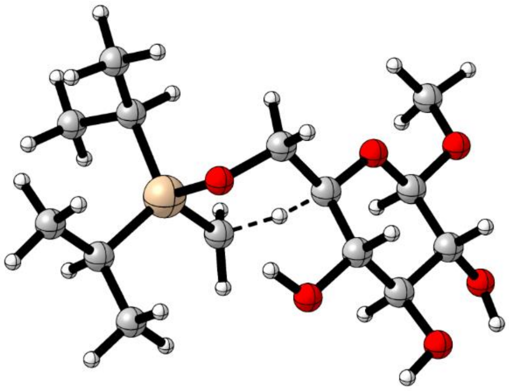

C	-3.955088	0.186444	-0.816216	C	1.443204	4.250724	-1.054617
C	-3.968557	-1.210400	-0.199344	H	1.363298	5.120364	-1.716584
C	-2.878609	-1.315741	0.863941	H	2.409983	4.315036	-0.548163
C	-1.519407	-0.926149	0.297586	H	1.460225	3.354834	-1.680849
C	-1.615602	0.435291	-0.383323	C	0.244096	5.510914	0.804819
O	-2.639338	0.485010	-1.328179	H	0.175293	6.399891	0.168252
O	-5.253622	-1.431773	0.369353	H	-0.610016	5.528990	1.489075
O	-2.900594	-2.662155	1.339702	H	1.153255	5.609850	1.406534
O	-0.585949	-0.946256	1.374306	C	1.369896	1.285348	3.228816
C	-0.320380	0.957355	-0.987579	H	1.987969	1.333391	4.131923
O	0.597814	1.319113	0.051591	H	0.353753	1.023949	3.535299
H	-5.194488	-2.235516	0.901370	H	1.757685	0.464963	2.616666
H	-2.297746	-2.726677	2.089750	C	2.881877	2.938110	2.046688
Si	0.201003	2.672432	1.029333	H	3.547610	2.886879	2.915609
H	-3.767592	-1.935865	-0.998453	H	3.250441	2.219041	1.308426
H	-3.119392	-0.628054	1.685170	H	2.979906	3.938044	1.618133
H	-1.229659	-1.669679	-0.463424	C	-1.569286	2.358075	1.530619
H	-1.806038	1.354054	0.600545	H	-1.770888	1.857072	2.476342
H	0.177783	-0.404933	1.124374	H	-2.318050	3.114555	1.296596
H	0.160651	0.189857	-1.603837	H	-4.204811	0.942097	-0.055408
H	-0.554043	1.810628	-1.634730	O	-4.839326	0.227964	-1.875847
C	0.275678	4.229187	-0.051470	C	-5.121587	1.550776	-2.355293
H	-0.657626	4.200804	-0.631557	H	-4.221169	2.021403	-2.757395
C	1.432424	2.621032	2.462543	H	-5.862379	1.440727	-3.145480
H	1.094182	3.414602	3.143459	H	-5.532980	2.171127	-1.551448

2c-C5-rad

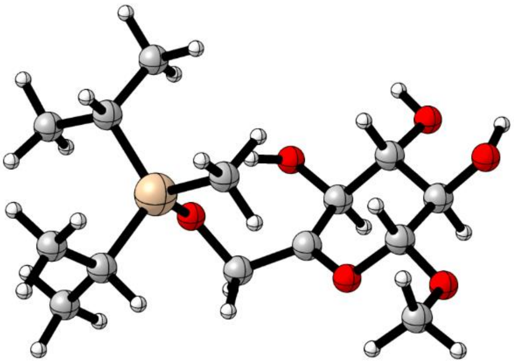

C	-2.527716	-0.952468	-0.902419	C	3.017690	2.983543	-1.402304
C	-2.498214	-2.401796	-0.425685	H	3.126885	3.822080	-2.099545
C	-1.214874	-2.659208	0.356201	H	3.920048	2.953751	-0.785426
C	0.010954	-2.356046	-0.509882	H	2.992389	2.062187	-1.989979
C	-0.120731	-0.995007	-1.119511	C	1.784060	4.479668	0.243477
O	-1.323171	-0.627959	-1.653029	H	1.903607	5.332073	-0.434486
O	-3.646516	-2.610256	0.388188	H	0.867229	4.643844	0.818006
O	-1.249543	-4.022405	0.773781	H	2.623670	4.501519	0.945713
O	1.210950	-2.493471	0.255681	C	2.058384	0.355338	3.007143
C	1.061343	-0.163408	-1.460739	H	2.592536	0.379471	3.963485
O	1.745347	0.248754	-0.246699	H	0.990168	0.278802	3.227590
H	-3.518371	-3.449608	0.848491	H	2.359647	-0.562306	2.491856
H	-0.481100	-4.181163	1.335239	C	3.917186	1.686627	1.918053
Si	1.361832	1.679335	0.584672	H	4.464806	1.627359	2.865568
H	-2.525543	-3.055966	-1.307451	H	4.263184	0.862177	1.286725
H	-1.192706	-2.001168	1.234888	H	4.203862	2.622244	1.432317
H	0.059561	-3.130806	-1.292810	C	-0.473769	1.709642	0.995525
H	-1.073868	1.701459	0.081362	H	-0.765360	0.849871	1.603556
H	1.514453	-1.602565	0.497057	H	-0.725839	2.618066	1.551803
H	1.803460	-0.725065	-2.038455	H	-2.568472	-0.270466	-0.041886
H	0.743627	0.696094	-2.058555	O	-3.601745	-0.766976	-1.745672
C	1.749358	3.155608	-0.546497	C	-3.909465	0.609064	-2.016798
H	0.892975	3.208441	-1.233879	H	-3.088826	1.094834	-2.550088
C	2.399513	1.601045	2.165143	H	-4.802305	0.607795	-2.639580
H	2.099835	2.486947	2.742678	H	-4.112941	1.147611	-1.084954

2d-CH_2-rad

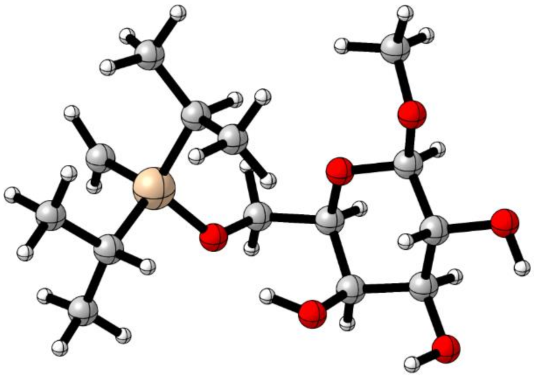

C	0.436195	-0.051478	1.579194	H	-1.719463	2.419134	0.938027
C	-0.720460	-0.895251	2.110538	C	-0.203197	5.090557	-0.740635
C	-1.159687	-1.920822	1.056762	H	0.396243	5.412567	0.118598
C	-1.464747	-1.244896	-0.279526	H	0.413658	5.210951	-1.635201
C	-0.260788	-0.378176	-0.687026	H	-1.046226	5.783369	-0.822966
O	0.065300	0.554321	0.342792	C	-3.773366	4.839279	-1.991741
O	-0.297781	-1.556280	3.300234	H	-4.851122	5.031355	-1.945673
O	-2.273924	-2.670961	1.544737	H	-3.311205	5.366107	-1.152967
C	-0.473189	0.407661	-1.974508	H	-3.400117	5.291334	-2.916770
O	-1.643166	1.227695	-1.900525	C	-4.270340	2.594279	-3.067456
H	-0.980538	-2.204102	3.517345	H	-5.346333	2.779322	-2.971972
H	-3.052876	-2.102506	1.468528	H	-3.963604	2.945686	-4.058390
Si	-1.642873	2.922850	-2.011668	H	-4.110206	1.513751	-3.031235
H	-1.554505	-0.214713	2.318719	C	-0.862003	3.385189	-3.626365
H	-0.352079	-2.645361	0.918943	H	-0.382150	4.342645	-3.807536
H	0.588580	-1.057353	-0.861253	H	-0.890483	2.727688	-4.491822
H	0.421059	1.008278	-2.170348	O	-2.671312	-0.500409	-0.102287
H	-0.596197	-0.298109	-2.801517	H	-2.641198	0.244282	-0.729000
C	-0.670108	3.635700	-0.548573	H	-1.609494	-2.016088	-1.047917
H	0.221768	2.999750	-0.490776	O	0.710429	0.959331	2.490717
C	-3.494813	3.325400	-1.956393	H	1.328032	-0.683600	1.418437
H	-3.839569	2.938127	-0.987828	C	1.868159	1.739502	2.173368
C	-1.436999	3.461162	0.775422	H	2.026100	2.414739	3.013225
H	-0.810964	3.764770	1.621872	H	2.748238	1.096935	2.051673
H	-2.342247	4.076701	0.797806	H	1.711145	2.319292	1.260825

TS4

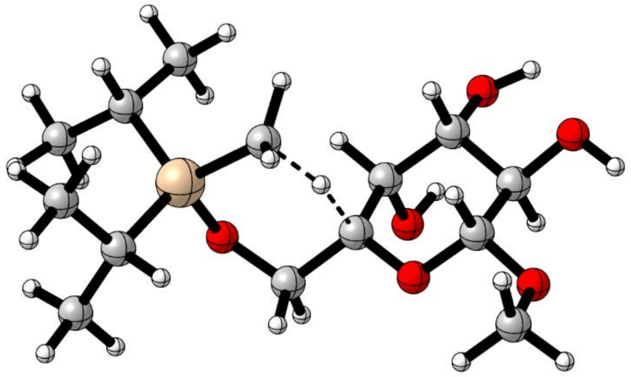

C	-3.901968	0.105289	-0.960709
C	-3.848553	-1.242573	-0.242456
C	-2.800002	-1.194953	0.862086
C	-1.442539	-0.781315	0.290802
C	-1.593131	0.486505	-0.528765
O	-2.608496	0.440009	-1.487903
O	-5.095480	-1.549688	0.374779
O	-2.636145	-2.469011	1.484065
C	-0.315485	1.030933	-1.148667
O	0.590655	1.457291	-0.136998
H	-5.754143	-1.672980	-0.319078
H	-3.514052	-2.824498	1.670724
Si	0.181011	2.737300	0.908710
H	-3.574393	-2.009047	-0.975502
H	-3.109740	-0.450863	1.607352
H	-1.834407	1.453191	0.386184
H	0.180199	0.255585	-1.740009
H	-0.585489	1.852617	-1.824170
C	0.422747	4.379781	-0.010895
H	-0.468591	4.464506	-0.648952
C	1.285872	2.488275	2.423445
H	0.977713	3.263146	3.138833
C	1.654476	4.410437	-0.933669
H	1.677290	5.340283	-1.513561
H	2.587195	4.359465	-0.364718
H	1.652953	3.574982	-1.638638
C	0.414103	5.578532	0.957832
H	0.443228	6.524739	0.406024
H	-0.480233	5.592605	1.588930
H	1.286267	5.559526	1.619508
C	1.034322	1.113648	3.073821
H	1.624714	1.007269	3.990758
H	-0.016078	0.965098	3.340169
H	1.325190	0.303574	2.398086
C	2.785139	2.682485	2.126819
H	3.380476	2.511554	3.031032
H	3.133859	1.977022	1.366093
H	3.008107	3.692233	1.773905
C	-1.633847	2.494644	1.283444
H	-1.928665	2.055486	2.235826
H	-2.339664	3.254175	0.947649
O	-0.903035	-1.813789	-0.546110
H	-1.013374	-2.647403	-0.069225
H	-0.753455	-0.577632	1.117765
O	-4.793809	-0.004831	-2.014292
H	-4.207606	0.901284	-0.263377
C	-5.180103	1.247452	-2.599839
H	-5.908673	1.014670	-3.374580
H	-5.639468	1.897582	-1.847399
H	-4.318167	1.750928	-3.044078

2d-C5-rad

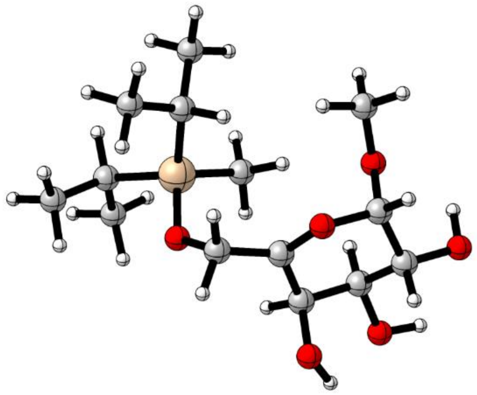

C	-2.368775	-0.695804	-1.524632	H	2.776153	2.289227	-1.767636
C	-2.692263	-2.027480	-0.840968	C	0.495282	3.890587	0.356471
C	-1.732694	-2.259691	0.325891	H	0.216495	4.675288	-0.356074
C	-0.290166	-2.233992	-0.185444	H	-0.405526	3.614222	0.911095
C	-0.047314	-1.021921	-1.016987	H	1.202162	4.333408	1.066644
O	-0.995375	-0.635325	-1.936589	C	2.963213	0.696468	3.215117
O	-4.050784	-2.072517	-0.416522	H	3.801335	0.939600	3.878291
O	-1.958685	-3.530695	0.935160	H	2.041114	0.815136	3.789910
C	1.334003	-0.532068	-1.298668	H	3.055595	-0.362079	2.948746
O	2.001734	-0.080857	-0.114370	C	4.353047	1.477514	1.252608
H	-4.229224	-1.258263	0.074501	H	5.166158	1.725005	1.944895
H	-2.912234	-3.640494	1.042333	H	4.518890	0.457260	0.895014
Si	1.518221	1.255226	0.807300	H	4.436973	2.144795	0.392169
H	-2.568932	-2.830985	-1.568170	C	-0.025485	0.856795	1.807644
H	-1.859640	-1.459731	1.062862	H	0.084133	-0.097088	2.330731
H	-0.906831	0.797746	1.164339	H	-0.202563	1.631065	2.561171
H	1.959856	-1.335086	-1.707750	O	-2.671939	0.319198	-0.610871
H	1.278644	0.261195	-2.052300	C	-2.742569	1.633682	-1.178597
C	1.117731	2.686626	-0.376041	H	-2.955296	2.315733	-0.357407
H	0.346343	2.276382	-1.042329	H	-1.792276	1.907269	-1.642612
C	2.988621	1.591397	1.959437	H	-3.544488	1.687698	-1.922293
H	2.861487	2.632437	2.289802	H	-2.938019	-0.575592	-2.450608
C	2.300355	3.130526	-1.255329	O	-0.024544	-3.411476	-0.990012
H	1.968559	3.841941	-2.020224	H	-0.377385	-4.166459	-0.497942
H	3.068063	3.632189	-0.658442	H	0.399536	-2.217678	0.665322

TS5

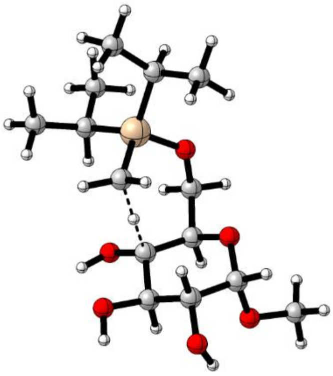

C	-3.032913	0.866815	-0.104072	C	4.058665	-0.174528	1.572023
C	-2.902321	-0.335926	-1.047016	H	4.552508	-0.936508	2.185836
C	-2.246397	-1.507894	-0.314587	H	4.839958	0.346897	1.008968
C	-0.964660	-1.063111	0.351446	H	3.599675	0.550972	2.250453
C	-1.141514	0.176951	1.213198	C	3.679522	-1.876990	-0.276224
O	-1.781492	1.215586	0.455422	H	4.188589	-2.635312	0.330052
O	-4.158995	-0.712837	-1.600300	H	2.946078	-2.394536	-0.900244
O	-1.921708	-2.579254	-1.203029	H	4.429833	-1.438692	-0.941017
O	-0.327637	-2.071400	1.078308	C	4.080227	1.410177	-2.055215
C	0.152621	0.750892	1.791511	H	4.644389	2.254157	-2.468368
O	1.045431	1.300988	0.838341	H	4.802541	0.719418	-1.612605
H	-4.793754	-0.751237	-0.871457	H	3.599793	0.896856	-2.895349
H	-2.746018	-2.925752	-1.564990	C	2.144949	2.996308	-1.641574
Si	2.009540	0.495483	-0.295260	H	2.749049	3.840217	-1.994380
H	-2.268720	-0.047270	-1.887180	H	1.586798	2.613187	-2.502763
H	-2.936695	-1.870631	0.462349	H	1.421075	3.378830	-0.917499
H	-0.134287	-0.706126	-0.635377	C	0.856623	-0.307688	-1.516131
H	-1.773729	-0.093520	2.073109	H	0.314886	0.349673	-2.196900
H	-0.363710	-2.882797	0.553971	H	1.142212	-1.241805	-1.997270
H	-0.124364	1.559130	2.473637	O	-3.986103	0.512401	0.869402
H	0.631248	-0.038340	2.382859	C	-4.408063	1.598181	1.702236
C	3.024264	-0.816421	0.628329	H	-5.168591	1.200922	2.372631
H	2.279867	-1.337484	1.245413	H	-3.570661	1.988574	2.286789
C	3.041729	1.903072	-1.030675	H	-4.836870	2.404443	1.096651
H	3.582027	2.343314	-0.181802	H	-3.356314	1.758319	-0.652133

2a-C4-rad-ax

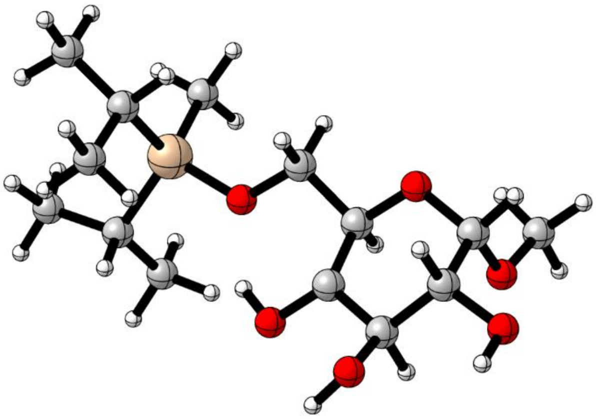

C	3.618974	1.115450	0.032142
C	4.094893	-0.252063	0.522114
C	3.208944	-1.358108	-0.042634
C	1.767775	-1.015813	0.155130
C	1.324622	0.412311	-0.037971
O	2.287474	1.337837	0.482917
O	5.460594	-0.428311	0.169267
O	3.595407	-2.571906	0.624948
O	0.907756	-1.999881	-0.248865
C	0.011821	0.730995	0.680262
O	-0.985434	-0.159136	0.176618
H	5.715764	-1.316363	0.448196
H	3.106960	-3.302445	0.228582
Si	-2.641578	-0.095775	0.573205
H	3.981348	-0.250638	1.613034
H	3.425984	-1.461570	-1.118500
H	-3.110713	1.757861	-1.015863
H	1.176426	0.604376	-1.114249
H	0.002152	-1.629795	-0.192304
H	0.148418	0.593230	1.758309
H	-0.256815	1.774801	0.486894
C	-2.771925	-0.318219	2.450660
H	-2.059540	0.414144	2.857710
C	-3.343165	-1.530394	-0.439043
H	-2.812596	-2.425598	-0.087822
C	-2.307517	-1.718628	2.892840
H	-2.279470	-1.792591	3.985636
H	-2.989899	-2.494431	2.530296
H	-1.306906	-1.955456	2.518575
C	-4.155838	0.025981	3.033455
H	-4.135638	-0.032090	4.127427
H	-4.476034	1.035816	2.762150
H	-4.925244	-0.670171	2.687131
C	-4.849878	-1.750116	-0.203012
H	-5.215280	-2.583366	-0.813378
H	-5.075730	-1.985497	0.840020
H	-5.432722	-0.865200	-0.479754
C	-3.041877	-1.359511	-1.940862
H	-3.381744	-2.235959	-2.503792
H	-3.560623	-0.486917	-2.351734
H	-1.972989	-1.231967	-2.131136
C	-3.328311	1.566667	0.038464
H	-4.413987	1.594553	0.170287
H	-2.902343	2.382457	0.629871
O	3.721774	1.161467	-1.363079
C	3.476025	2.456940	-1.920079
H	3.631151	2.368735	-2.994376
H	2.450727	2.783174	-1.723750
H	4.175220	3.194279	-1.509028
H	4.207306	1.915354	0.495187

TS6

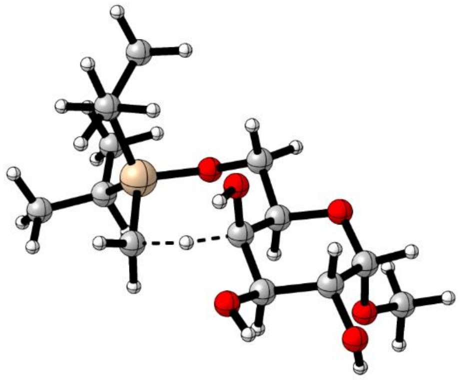

C	-3.248989	0.996906	-0.654707	C	4.278930	-2.146189	0.988675
C	-3.787171	-0.270981	0.026804	H	5.197640	-1.744196	1.430658
C	-2.648940	-1.030594	0.730394	H	3.633270	-2.477024	1.807615
C	-1.538308	-1.272145	-0.270500	H	4.560087	-3.031882	0.408369
C	-1.069008	0.027979	-0.888477	C	0.959577	-4.125745	-1.837949
O	-2.177312	0.703356	-1.531495	H	1.159273	-4.929768	-2.556146
O	-4.849620	0.021901	0.927911	H	0.793135	-4.590478	-0.862520
O	-3.100953	-2.295881	1.212144	H	0.025570	-3.638551	-2.130671
C	0.032744	-0.136098	-1.937557	C	2.434367	-2.613370	-3.233586
O	1.301465	-0.327260	-1.327452	H	2.664040	-3.451448	-3.901543
H	-4.560217	0.756757	1.486569	H	1.573042	-2.087153	-3.655543
H	-3.953213	-2.160314	1.645606	H	3.282685	-1.925721	-3.261208
Si	1.926416	-1.701951	-0.561726	C	0.778902	-2.152681	0.840005
H	-4.207028	-0.919251	-0.743945	H	0.596717	-3.202573	1.065723
H	-2.264146	-0.419161	1.555005	H	0.874582	-1.556897	1.749583
H	-0.217570	-0.955039	-2.618864	O	-2.877901	1.877240	0.380008
H	0.080433	0.787535	-2.519083	C	-2.557931	3.201673	-0.059654
C	3.594040	-1.090138	0.100628	H	-2.337475	3.783158	0.834188
H	3.331299	-0.233411	0.737086	H	-1.684461	3.193059	-0.717184
C	2.132096	-3.126128	-1.810339	H	-3.407369	3.648670	-0.588436
H	3.021490	-3.669566	-1.460155	H	-4.019580	1.451071	-1.287109
C	4.552586	-0.590765	-0.995190	H	-0.679683	0.665464	-0.087655
H	5.444834	-0.134625	-0.550650	O	-1.877538	-2.213684	-1.246668
H	4.893920	-1.417248	-1.626889	H	-2.357362	-2.928283	-0.804785
H	4.081684	0.153948	-1.642182	H	-0.458653	-1.728961	0.360196

2b-C4-rad-eq

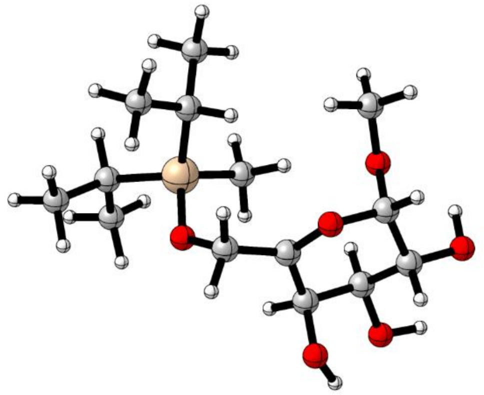

C	-2.368775	-0.695804	-1.524632
C	-2.692263	-2.027480	-0.840968
C	-1.732694	-2.259691	0.325891
C	-0.290166	-2.233992	-0.185444
C	-0.047314	-1.021921	-1.016987
O	-0.995375	-0.635325	-1.936589
O	-4.050784	-2.072517	-0.416522
O	-1.958685	-3.530695	0.935160
C	1.334003	-0.532068	-1.298668
O	2.001734	-0.080857	-0.114370
H	-4.229224	-1.258263	0.074501
H	-2.912234	-3.640494	1.042333
Si	1.518221	1.255226	0.807300
H	-2.568932	-2.830985	-1.568170
H	-1.859640	-1.459731	1.062862
H	-0.906831	0.797746	1.164339
H	1.959856	-1.335086	-1.707750
H	1.278644	0.261195	-2.052300
C	1.117731	2.686626	-0.376041
H	0.346343	2.276382	-1.042329
C	2.988621	1.591397	1.959437
H	2.861487	2.632437	2.289802
C	2.300355	3.130526	-1.255329
H	1.968559	3.841941	-2.020224
H	3.068063	3.632189	-0.658442

H	2.776153	2.289227	-1.767636
C	0.495282	3.890587	0.356471
H	0.216495	4.675288	-0.356074
H	-0.405526	3.614222	0.911095
H	1.202162	4.333408	1.066644
C	2.963213	0.696468	3.215117
H	3.801335	0.939600	3.878291
H	2.041114	0.815136	3.789910
H	3.055595	-0.362079	2.948746
C	4.353047	1.477514	1.252608
H	5.166158	1.725005	1.944895
H	4.518890	0.457260	0.895014
H	4.436973	2.144795	0.392169
C	-0.025485	0.856795	1.807644
H	0.084133	-0.097088	2.330731
H	-0.202563	1.631065	2.561171
O	-2.671939	0.319198	-0.610871
C	-2.742569	1.633682	-1.178597
H	-2.955296	2.315733	-0.357407
H	-1.792276	1.907269	-1.642612
H	-3.544488	1.687698	-1.922293
H	-2.938019	-0.575592	-2.450608
O	-0.024544	-3.411476	-0.990012
H	-0.377385	-4.166459	-0.497942
H	0.399536	-2.217678	0.665322

TS7

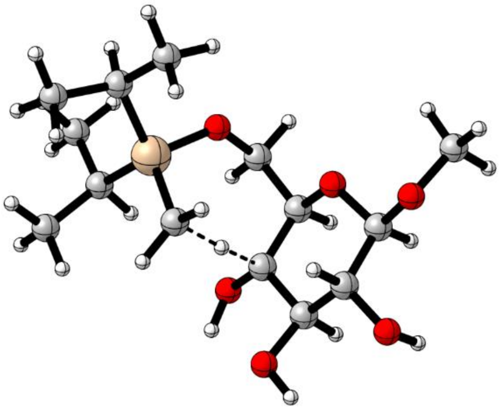

C	-3.088173	0.803972	-0.107871
C	-2.935648	-0.376854	-1.073682
C	-2.247017	-1.539869	-0.353338
C	-0.976458	-1.089392	0.330409
C	-1.177152	0.150024	1.189117
O	-1.807139	1.166788	0.408461
O	-4.170098	-0.802659	-1.643318
O	-1.903545	-2.596058	-1.249322
O	-0.341550	-2.096239	1.059291
C	0.104929	0.731519	1.790006
O	1.004410	1.293590	0.850825
H	-4.792816	-1.011810	-0.935288
H	-2.718010	-2.930394	-1.644161
Si	1.983364	0.503356	-0.281042
H	-2.315613	-0.051136	-1.911020
H	-2.933938	-1.927722	0.419150
H	-0.141900	-0.717845	-0.646119
H	-1.822317	-0.122573	2.043241
H	-0.352809	-2.903013	0.526828
H	-0.188009	1.533974	2.472148
H	0.582452	-0.056818	2.383461
C	2.998669	-0.809967	0.640159
H	2.254873	-1.333865	1.255688
C	3.013930	1.920109	-1.000438
H	3.551902	2.355120	-0.147467
C	4.032048	-0.169476	1.586041
H	4.526636	-0.932678	2.197693
H	4.812878	0.354855	1.025043
H	3.571908	0.553351	2.266633
C	3.654675	-1.866930	-0.267935
H	4.166835	-2.625430	0.335481
H	2.920746	-2.384527	-0.891306
H	4.402296	-1.425377	-0.933587
C	4.055239	1.435472	-2.026272
H	4.620364	2.282713	-2.431294
H	4.776550	0.741097	-1.587571
H	3.576703	0.928873	-2.871546
C	2.116555	3.015807	-1.605719
H	2.719935	3.862648	-1.952602
H	1.559860	2.637115	-2.469767
H	1.391387	3.392756	-0.880097
C	0.844534	-0.295804	-1.517565
H	0.294192	0.365349	-2.187552
H	1.142253	-1.218505	-2.013079
H	-3.748574	0.529676	0.735513
O	-3.601657	1.890063	-0.797719
C	-4.018616	2.974600	0.042526
H	-4.444009	3.730128	-0.616237
H	-4.781751	2.638952	0.754051
H	-3.170822	3.396413	0.587675

2c-C4-rad-ax

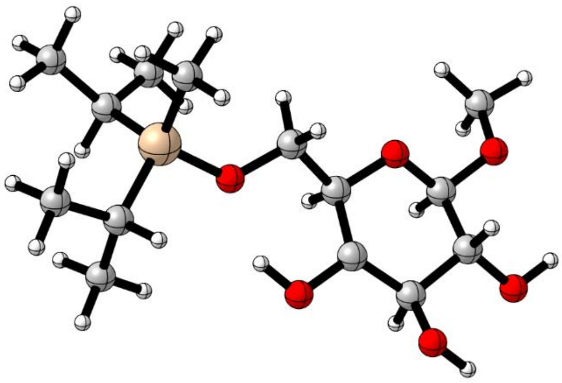

C	1.624070	-3.072941	1.231047	H	3.588233	-4.291473	-0.073484
C	0.569291	-3.317589	2.307245	H	4.106581	-2.624522	0.315447
C	-0.797487	-2.812212	1.846827	C	-2.371838	2.875342	-0.777131
C	-0.686682	-1.454858	1.231652	H	-2.573296	2.671549	0.284171
C	0.529448	-1.140890	0.390366	C	0.079241	2.591357	-2.760928
O	1.715717	-1.671648	0.979289	H	-0.738731	2.171480	-3.361050
O	0.422755	-4.703709	2.600884	C	-3.235688	1.914957	-1.616907
O	-1.724251	-2.775375	2.939138	H	-4.298918	2.058357	-1.395111
O	-1.873696	-0.921058	0.820117	H	-3.099254	2.096009	-2.687932
C	0.773184	0.361082	0.226399	H	-2.997961	0.864360	-1.427493
O	-0.379846	0.926738	-0.401449	C	-2.759210	4.340536	-1.054302
H	1.267118	-5.026357	2.938338	H	-3.815904	4.507727	-0.818344
H	-1.825650	-3.681750	3.254906	H	-2.172637	5.046058	-0.459029
Si	-0.519697	2.532545	-0.960966	H	-2.620836	4.596482	-2.109224
H	0.872426	-2.759109	3.200797	C	0.329954	4.034492	-3.243504
H	-1.176165	-3.522589	1.088519	H	0.606651	4.041683	-4.303643
H	0.202570	3.542479	1.192229	H	-0.548757	4.674799	-3.129919
H	0.396782	-1.570559	-0.621184	H	1.151707	4.498758	-2.688674
H	-1.658848	-0.115982	0.303927	C	1.326198	1.717983	-2.999635
H	0.934925	0.812062	1.210879	H	1.619602	1.748903	-4.055075
H	1.671715	0.505454	-0.379995	H	2.183144	2.072974	-2.416784
O	2.843836	-3.520607	1.718930	H	1.148951	0.672089	-2.736245
H	1.359646	-3.594542	0.295355	C	0.516391	3.636133	0.148572
C	3.881207	-3.609170	0.732051	H	1.578618	3.382156	0.087330
H	4.759973	-4.002191	1.240764	H	0.409173	4.684097	-0.144012

TS8

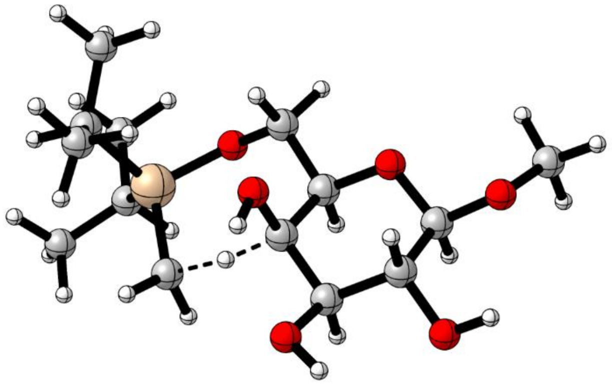

C	-3.223756	1.022978	-0.577508	C	4.257497	-2.150566	1.005915
C	-3.789174	-0.253254	0.055210	H	5.170445	-1.748869	1.459905
C	-2.668974	-1.056746	0.721384	H	3.608070	-2.496170	1.815757
C	-1.552676	-1.277965	-0.280284	H	4.548337	-3.026766	0.416102
C	-1.084706	0.033485	-0.883195	C	0.966334	-4.115361	-1.858068
O	-2.192519	0.705860	-1.511413	H	1.173975	-4.910700	-2.583616
O	-4.753023	0.042344	1.061542	H	0.798384	-4.591168	-0.888256
O	-3.133046	-2.333983	1.157018	H	0.030745	-3.630685	-2.149915
C	0.024503	-0.120447	-1.927438	C	2.441417	-2.582308	-3.231373
O	1.289022	-0.317738	-1.311525	H	2.680484	-3.413200	-3.904963
H	-5.503889	0.478010	0.640239	H	1.579850	-2.057628	-3.654714
H	-3.977741	-2.208070	1.607042	H	3.285932	-1.889574	-3.247337
Si	1.912988	-1.697931	-0.554624	C	0.756898	-2.164735	0.835462
H	-4.232384	-0.856228	-0.745721	H	0.573768	-3.217126	1.048378
H	-2.281361	-0.481210	1.572079	H	0.848329	-1.579465	1.752229
H	-0.222479	-0.932702	-2.617675	H	-0.691658	0.661348	-0.071606
H	0.075150	0.809038	-2.499193	O	-1.880402	-2.208129	-1.268787
C	3.572750	-1.086738	0.127075	H	-2.378252	-2.920193	-0.842713
H	3.301005	-0.239927	0.772940	H	-0.474094	-1.738007	0.353722
C	2.132978	-3.109381	-1.814696	O	-4.264840	1.649618	-1.250338
H	3.023263	-3.651041	-1.464015	H	-2.811654	1.683601	0.204644
C	4.535676	-0.567459	-0.955534	C	-3.983992	2.997532	-1.652495
H	5.424252	-0.116117	-0.498917	H	-4.891079	3.376538	-2.120621
H	4.882566	-1.382678	-1.598695	H	-3.735508	3.614785	-0.782058
H	4.065923	0.186467	-1.592640	H	-3.159982	3.026126	-2.369449

2d-C4-rad-eq

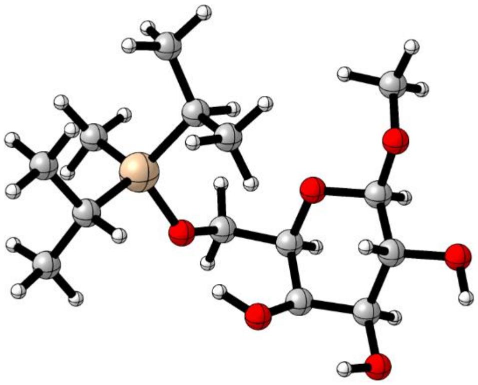

C	-3.338299	-0.729031	0.286507
C	-3.624426	0.750720	0.014113
C	-2.642798	1.291997	-1.055369
C	-1.239622	0.980413	-0.625234
C	-1.023768	-0.477415	-0.331825
O	-1.978484	-0.909456	0.676561
O	-4.970241	0.884497	-0.423282
O	-2.891857	2.677908	-1.281558
C	0.366486	-0.841004	0.171830
O	0.773939	0.011679	1.247905
H	-5.075832	1.793696	-0.732063
H	-2.458739	3.173264	-0.573298
Si	1.100556	-0.492374	2.844517
H	-3.459390	1.299612	0.950989
H	-2.866486	0.783055	-1.997885
H	3.231585	-1.598780	2.194770
H	-1.205367	-1.050148	-1.252235
H	0.363874	-1.892309	0.474211
H	1.079587	-0.721513	-0.648464
C	-0.526410	-1.065464	3.625454
H	-0.987807	-1.686637	2.847408
C	1.811432	1.078954	3.619878
H	1.023803	1.837436	3.515566
C	-1.481334	0.113538	3.887606
H	-2.455619	-0.249081	4.234251

H	-1.088136	0.785081	4.657930
H	-1.655151	0.697564	2.980424
C	-0.347691	-1.937337	4.882763
H	-1.319556	-2.300370	5.236372
H	0.280407	-2.811774	4.690369
H	0.106865	-1.378516	5.706529
C	2.112763	0.914111	5.121827
H	2.514578	1.845112	5.536910
H	1.219881	0.657539	5.697551
H	2.859026	0.131943	5.297585
C	3.054986	1.578516	2.860356
H	3.404049	2.531057	3.274773
H	3.882134	0.865309	2.942461
H	2.848456	1.729649	1.797647
C	2.353638	-1.889034	2.778168
H	2.687758	-2.150417	3.786543
H	1.926245	-2.790255	2.328949
O	-0.720992	1.918369	0.226726
H	-0.024555	1.478830	0.764771
H	-3.535798	-1.323469	-0.623569
O	-4.147055	-1.154780	1.333272
C	-4.097284	-2.565460	1.575947
H	-3.105654	-2.868879	1.920648
H	-4.833963	-2.772126	2.351073
H	-4.355876	-3.124498	0.668913

TS9

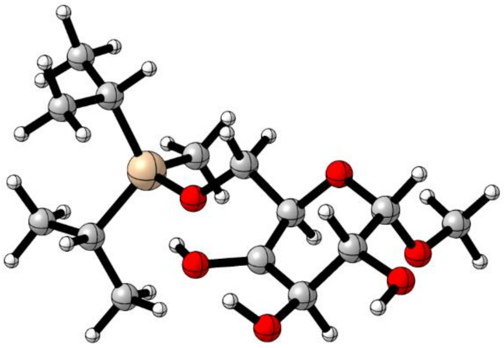

C	3.634459	1.048056	0.049715
C	4.137552	-0.392722	0.171141
C	3.202725	-1.357543	-0.582608
C	1.789255	-1.086729	-0.166885
C	1.331444	0.332552	-0.014342
O	2.344945	1.154782	0.632167
O	5.473774	-0.464438	-0.306106
O	3.621461	-2.706989	-0.373723
O	1.043127	-2.148817	0.263805
C	0.091129	0.485463	0.865902
O	-0.926766	-0.378572	0.360680
H	5.735628	-1.393260	-0.274100
H	3.211650	-3.033655	0.437132
Si	-2.599614	-0.171485	0.609410
H	4.097565	-0.650110	1.237912
H	3.345780	-1.172919	-1.655732
H	-2.818644	1.487880	-1.227569
H	1.127150	0.779064	-0.997451
H	0.118743	-1.834927	0.352690
H	0.344290	0.209269	1.895338
H	-0.224018	1.532931	0.855459
C	-2.885294	-0.145697	2.482872
H	-2.134978	0.565491	2.858503
C	-3.324455	-1.676803	-0.273934
H	-2.883798	-2.549505	0.226349
C	-2.595051	-1.514923	3.125892
H	-2.653300	-1.451987	4.218171
H	-3.322981	-2.266185	2.802596
H	-1.598506	-1.886428	2.868288
C	-4.268066	0.389584	2.901461
H	-4.337818	0.459009	3.992757
H	-4.463146	1.385407	2.493972
H	-5.074925	-0.269288	2.567252
C	-4.855752	-1.772491	-0.134163
H	-5.233934	-2.654710	-0.662404
H	-5.171883	-1.855610	0.908822
H	-5.353903	-0.897680	-0.565389
C	-2.902059	-1.717269	-1.755420
H	-3.262625	-2.634816	-2.233729
H	-3.323963	-0.872955	-2.310890
H	-1.815472	-1.683207	-1.869721
C	-3.123779	1.449159	-0.178376
H	-4.211043	1.562945	-0.137493
H	-2.682606	2.306302	0.338651
O	3.640080	1.412528	-1.305033
C	3.352934	2.794845	-1.534497
H	3.444353	2.958928	-2.607359
H	2.339088	3.047164	-1.210642
H	4.069897	3.433360	-1.005123
H	4.266187	1.725743	0.636055

TS10

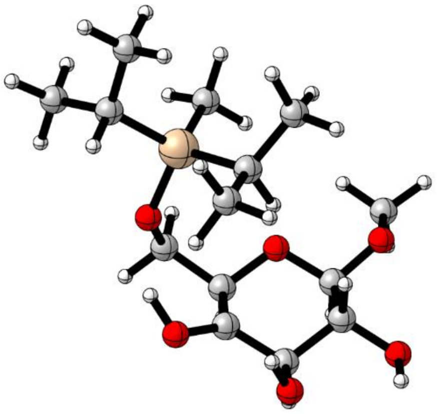

C	-2.955158	-1.437854	0.250899	H	-0.336904	2.251886	4.420065
C	-3.678339	-0.118411	0.535601	H	-0.780608	2.451341	2.721470
C	-3.064149	1.043653	-0.281777	C	-1.231385	-0.417252	4.686476
C	-1.579438	0.991283	-0.161136	H	-2.253890	-0.164458	4.989858
C	-0.918791	-0.333472	-0.366516	H	-1.193636	-1.498334	4.526348
O	-1.555501	-1.306982	0.496959	H	-0.576507	-0.184270	5.531571
O	-5.054989	-0.281558	0.218214	C	2.361601	0.593498	5.046168
O	-3.642811	2.281773	0.125958	H	3.205215	1.144578	5.476858
C	0.577202	-0.327719	-0.070114	H	1.455689	0.932716	5.554710
O	0.906182	0.431291	1.094160	H	2.505729	-0.463974	5.292716
H	-5.474011	0.582711	0.317802	C	3.636776	0.459303	2.856753
H	-3.153197	2.607148	0.892278	H	4.448740	1.065630	3.274126
Si	0.854264	-0.110147	2.711974	H	3.896289	-0.591578	3.024271
H	-3.550657	0.109130	1.601613	H	3.611622	0.628884	1.776967
H	-3.390501	0.907069	-1.325196	C	1.095064	-1.970992	2.735452
H	2.003428	-2.264928	2.202155	H	1.181203	-2.327544	3.765848
H	-1.044005	-0.687851	-1.406250	H	0.242024	-2.473196	2.271728
H	0.919623	-1.362368	0.015814	O	-0.883162	2.153889	-0.009131
H	1.098870	0.133406	-0.912369	H	-0.045253	1.925495	0.441115
C	-0.838729	0.366712	3.419913	H	-3.109877	-1.730600	-0.802854
H	-1.525811	0.062597	2.622344	O	-3.450503	-2.411342	1.109120
C	2.293685	0.812705	3.522649	C	-3.016441	-3.741135	0.798176
H	2.096503	1.878331	3.342624	H	-1.934456	-3.838441	0.916659
C	-0.999192	1.883262	3.630137	H	-3.523321	-4.403571	1.498418
H	-2.026590	2.124770	3.926385	H	-3.297469	-4.009847	-0.226750

TS11

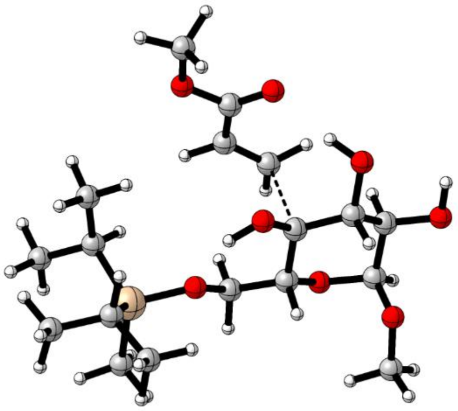

C	3.584371	-1.626025	-0.698797
C	3.949661	-0.201351	-0.274778
C	2.972113	0.323555	0.786902
C	1.547412	0.101072	0.353801
C	1.265258	-1.270998	-0.229806
O	2.246441	-1.633228	-1.202106
O	5.286759	-0.178217	0.206517
O	3.295504	1.667897	1.130275
O	0.636301	0.587748	1.223845
C	-0.099541	-1.414337	-0.902569
O	-1.094815	-1.033344	0.047915
H	5.465087	0.725873	0.495506
H	2.620733	2.292470	0.797938
Si	-2.777711	-1.219045	-0.171922
H	3.869638	0.417595	-1.173595
H	3.128791	-0.277659	1.695392
H	-2.606802	-3.692059	-0.379851
H	1.294804	-1.984707	0.609140
H	-0.230730	0.186472	0.993112
H	-0.151288	-0.783241	-1.793733
H	-0.225411	-2.457040	-1.208433
C	-3.355551	0.195236	-1.295389
H	-2.569668	0.268942	-2.061540
C	-3.447695	-1.106391	1.590726
H	-3.164556	-0.108878	1.952379
C	-3.415671	1.541668	-0.548644
H	-3.615738	2.363003	-1.245568
H	-4.218069	1.542911	0.195730
H	-2.483114	1.776803	-0.029323
C	-4.681679	-0.092369	-2.025685
H	-4.930029	0.728945	-2.707106
H	-4.633914	-1.010640	-2.617183
H	-5.515058	-0.193230	-1.323341
C	-4.984607	-1.210395	1.639627
H	-5.342349	-1.119272	2.671048
H	-5.473805	-0.427972	1.053358
H	-5.331555	-2.177074	1.259712
C	-2.793744	-2.150310	2.515640
H	-3.142237	-2.024147	3.546808
H	-3.050510	-3.169514	2.207527
H	-1.703993	-2.065737	2.515324
C	-3.087525	-2.895644	-0.954679
H	-4.160713	-3.104305	-0.988276
H	-2.710791	-2.937183	-1.980628
C	1.374360	1.521916	-1.539103
H	2.357847	1.907157	-1.308743
H	1.331511	0.735804	-2.280115
C	0.278994	2.276922	-1.238357
C	0.394853	3.355276	-0.273001
O	-0.760360	4.029888	-0.105620
O	1.416252	3.641660	0.352625
C	-0.753216	5.094239	0.867635
H	-0.036637	5.866302	0.583853
H	-1.764186	5.494643	0.866785
H	-0.497372	4.704851	1.854115
H	-0.708900	2.035323	-1.606845
O	3.742155	-2.486164	0.390903
H	4.198535	-1.948150	-1.546660
C	3.594514	-3.872574	0.063595
H	4.309827	-4.164710	-0.713662
H	3.799610	-4.430846	0.975702
H	2.579262	-4.090948	-0.279433

3a'-Glu-C-rad

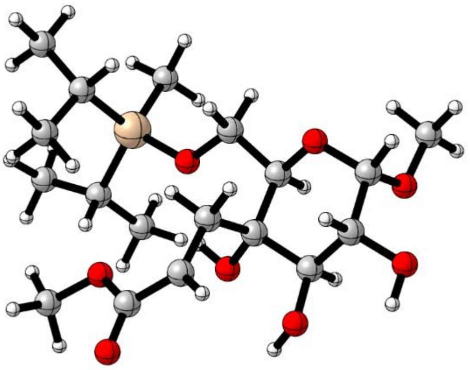

C	3.695271	-1.467843	-0.664228
C	3.999744	0.008836	-0.388817
C	3.012868	0.560870	0.635495
C	1.549606	0.354821	0.199398
C	1.384410	-1.161252	-0.095199
O	2.337539	-1.596739	-1.075488
O	5.344408	0.137451	0.053402
O	3.352013	1.931115	0.843972
O	0.772970	0.721599	1.336593
C	0.022090	-1.568709	-0.651728
O	-0.995704	-0.986328	0.156104
H	5.466838	1.056383	0.323134
H	2.821855	2.264983	1.577309
Si	-2.656072	-1.327471	0.037327
H	3.869202	0.543679	-1.337060
H	3.143612	0.006783	1.571782
H	-2.435000	-3.508222	1.210315
H	1.553199	-1.685344	0.851610
H	-0.132479	0.419240	1.156234
H	-0.063661	-1.230894	-1.690267
H	-0.042971	-2.661927	-0.648200
C	-3.236702	-0.786367	-1.685381
H	-2.463978	-1.175574	-2.364413
C	-3.372543	-0.299051	1.452627
H	-3.052227	0.731134	1.250065
C	-3.263287	0.746186	-1.838285
H	-3.474929	1.028715	-2.875687
H	-4.046849	1.190563	-1.215815
H	-2.315651	1.209324	-1.552916
C	-4.576861	-1.414000	-2.115399
H	-4.824310	-1.122885	-3.142453
H	-4.549521	-2.506462	-2.079065
H	-5.401739	-1.081896	-1.477634
C	-4.912078	-0.320013	1.491458
H	-5.283535	0.296128	2.317781
H	-5.355703	0.066108	0.570239
H	-5.294494	-1.334602	1.645730
C	-2.783403	-0.716529	2.813849
H	-3.141262	-0.052886	3.608892
H	-3.084526	-1.735442	3.079006
H	-1.690628	-0.682327	2.816853
C	-2.912726	-3.170638	0.286467
H	-3.979004	-3.406433	0.350911
H	-2.496363	-3.747203	-0.544835
O	3.973050	-2.213698	0.485842
C	3.875355	-3.628869	0.295457
H	4.171316	-4.090429	1.236358
H	2.851759	-3.924271	0.048412
H	4.549869	-3.960248	-0.502594
H	4.280027	-1.832417	-1.515760
C	1.127030	1.198584	-1.048519
H	1.728257	0.872435	-1.900367
H	0.087102	0.941434	-1.253489
C	1.233665	2.667384	-0.878236
C	0.184775	3.504191	-0.346729
O	-0.982809	2.846324	-0.142000
O	0.316979	4.701794	-0.106086
C	-2.071578	3.622605	0.389181
H	-1.829841	3.981790	1.390985
H	-2.290309	4.472484	-0.259168
H	-2.922259	2.946548	0.424141
H	2.151816	3.182945	-1.119791

TS12

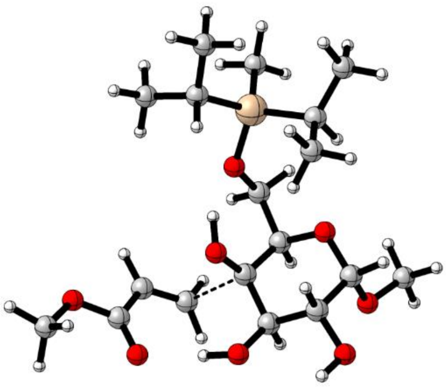

C	-0.304678	3.124592	-0.325091	C	4.164174	-2.796032	-1.330330
C	-1.372158	2.483791	-1.220605	H	4.083941	-3.708591	-1.931341
C	-2.246838	1.510714	-0.404613	H	4.527464	-2.002093	-1.987907
C	-1.308693	0.527280	0.246598	H	4.930431	-2.975333	-0.568620
C	-0.262094	1.190262	1.108969	C	2.254125	-3.643383	0.111580
O	0.496608	2.119422	0.280803	H	2.149658	-4.525213	-0.530503
O	-2.138000	3.506641	-1.839301	H	2.928829	-3.914934	0.930451
O	-3.176583	0.936209	-1.306048	H	1.274577	-3.424494	0.544416
C	0.736554	0.251171	1.771238	C	3.940890	-1.070767	1.883889
O	1.246819	-0.716677	0.848722	H	4.975193	-1.290532	1.603475
H	-2.811466	3.071148	-2.376483	H	3.949330	-0.156871	2.485159
H	-3.583682	0.143193	-0.904479	O	-0.938969	3.936764	0.626332
Si	2.872124	-0.875547	0.353480	C	-0.034321	4.720668	1.409887
H	-0.842295	1.892133	-1.976986	H	-0.645024	5.342625	2.062870
H	-2.758619	2.089361	0.374590	H	0.617997	4.084140	2.014634
H	3.578304	-1.889866	2.510798	H	0.581619	5.360372	0.766932
H	-0.764206	1.760990	1.896898	H	0.395425	3.711333	-0.930795
H	1.538906	0.854574	2.205552	O	-0.940787	-0.494696	-0.553537
H	0.237135	-0.284860	2.581843	H	-0.106734	-0.869140	-0.186857
C	3.336111	0.681601	-0.618175	C	-2.748163	-0.516556	1.909115
H	2.957248	1.502779	0.003872	H	-3.503593	0.178282	1.566192
C	2.805471	-2.449882	-0.691745	H	-2.166213	-0.215488	2.770343
H	2.097165	-2.232577	-1.502738	C	-2.771132	-1.813279	1.506721
C	2.596491	0.756138	-1.966845	C	-3.585270	-2.218684	0.372495
H	2.787114	1.716600	-2.459138	O	-3.537002	-3.547797	0.151742
H	2.928541	-0.033176	-2.649412	O	-4.249407	-1.464717	-0.338610
H	1.515429	0.662980	-1.838241	C	-4.287656	-4.043243	-0.976668
C	4.853074	0.883921	-0.794287	H	-4.111145	-5.115988	-0.993923
H	5.055860	1.839626	-1.290940	H	-5.350358	-3.832938	-0.848920
H	5.380429	0.892095	0.163791	H	-3.933410	-3.582268	-1.899752
H	5.300237	0.098714	-1.411405	H	-2.120578	-2.559996	1.944271

3a-Gal-C-rad

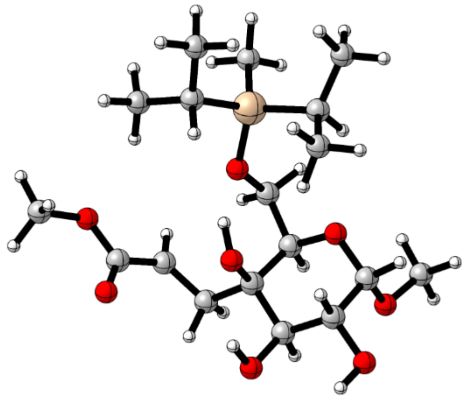

C	3.193678	-1.096689	-0.138487	C	-4.108300	-1.997943	-1.538019
C	3.244983	0.046701	-1.146976	H	-4.789116	-1.514568	-2.247594
C	2.792328	1.361740	-0.504268	H	-3.560663	-2.770471	-2.083796
C	1.391910	1.205029	0.129239	H	-4.726614	-2.496937	-0.784119
C	1.417377	-0.030923	1.069238	C	-3.971388	0.196379	-0.267533
O	1.873299	-1.200316	0.370161	H	-4.598087	0.687013	-1.021019
O	4.558268	0.154240	-1.683468	H	-4.638304	-0.170676	0.520093
O	2.810660	2.398017	-1.488092	H	-3.320857	0.955255	0.172050
C	0.080922	-0.406355	1.699777	C	-2.819015	-2.340379	1.892054
O	-0.982544	-0.391204	0.745412	H	-3.546635	-3.119764	1.647052
H	4.565955	0.938037	-2.247525	H	-2.102790	-2.767454	2.600323
H	2.036693	2.265540	-2.053898	O	4.136541	-0.861727	0.873921
Si	-1.961547	-1.724018	0.339609	C	4.272004	-1.946536	1.796400
H	2.529515	-0.206838	-1.937160	H	5.064781	-1.670059	2.490166
H	3.514160	1.654757	0.260042	H	3.343105	-2.113966	2.349477
H	-3.350224	-1.525765	2.391762	H	4.549931	-2.868749	1.272597
H	2.113619	0.190172	1.887067	H	3.384006	-2.058816	-0.627551
H	0.200113	-1.393933	2.156753	O	0.509739	1.017065	-0.973839
H	-0.158509	0.296983	2.499771	H	-0.287218	0.584276	-0.619280
C	-0.835967	-3.057003	-0.399823	C	1.044454	2.526451	0.904880
H	0.013894	-3.101707	0.294235	C	-0.388847	2.711341	1.228665
C	-3.169291	-0.958095	-0.897891	C	-1.286385	3.398982	0.326637
H	-2.540564	-0.536554	-1.694198	O	-2.570481	3.341150	0.764434
C	-0.275913	-2.637497	-1.772026	O	-0.963185	3.974635	-0.703713
H	0.448918	-3.375966	-2.133962	C	-3.558700	3.972801	-0.071828
H	-1.069359	-2.563970	-2.523243	H	-4.510178	3.814239	0.430122
H	0.236538	-1.674303	-1.716783	H	-3.351081	5.039596	-0.170050
C	-1.473313	-4.458463	-0.452070	H	-3.570294	3.511645	-1.060719
H	-0.747649	-5.195625	-0.814427	H	-0.816353	2.321347	2.142280
H	-1.817110	-4.790351	0.531724	H	1.643810	2.547514	1.820008
H	-2.331550	-4.490581	-1.130092	H	1.368183	3.344779	0.259034

TS13

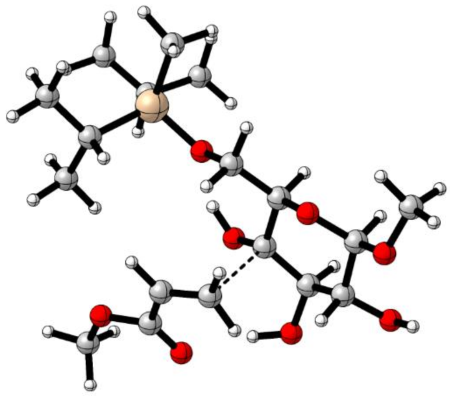

C	3.606615	-1.524628	-0.052988	H	-4.484741	-1.700160	-2.594345
C	3.933996	-0.064920	0.277322	H	-5.477057	-0.714278	-1.516485
C	2.829423	0.559382	1.137780	C	-5.083637	-1.163279	1.588726
C	1.461296	0.209057	0.604716	H	-5.509048	-0.921158	2.568777
C	1.271074	-1.226593	0.149519	H	-5.566028	-0.514165	0.853208
O	2.337622	-1.621859	-0.703264	H	-5.366327	-2.195785	1.357960
O	5.150375	0.009514	1.013386	C	-2.917020	-1.829493	2.733409
O	3.037655	1.953955	1.319332	H	-3.334212	-1.547940	3.706690
O	0.460228	0.758080	1.324697	H	-3.114120	-2.897421	2.591238
C	-0.033291	-1.491447	-0.604847	H	-1.833519	-1.692880	2.779241
O	-1.112680	-1.040114	0.212295	C	-2.935264	-3.166405	-0.560367
H	5.842881	-0.383905	0.468242	H	-3.991229	-3.448219	-0.604972
H	2.324824	2.473818	0.900855	H	-2.500731	-3.358006	-1.545574
Si	-2.765869	-1.363769	-0.068700	C	1.438650	1.381586	-1.455273
H	4.021454	0.480798	-0.667985	H	2.362603	1.857342	-1.156612
H	2.907454	0.094485	2.135350	H	1.534235	0.512782	-2.091642
H	-2.442371	-3.819106	0.165433	C	0.270953	2.082293	-1.378547
H	1.261650	-1.859543	1.055828	C	0.194780	3.267100	-0.541834
H	-0.370940	0.300639	1.065954	O	-1.013405	3.863982	-0.597462
H	-0.024379	-0.965309	-1.562989	O	1.106048	3.698846	0.164068
H	-0.106857	-2.565255	-0.799661	C	-1.201844	5.022266	0.240631
C	-3.342950	-0.205589	-1.454241	H	-0.502326	5.812224	-0.036245
H	-2.519220	-0.223673	-2.182777	H	-2.226238	5.342759	0.066496
C	-3.552380	-0.990923	1.608209	H	-1.057315	4.758948	1.289486
H	-3.333819	0.066468	1.808853	H	-0.646116	1.727477	-1.830247
C	-3.509960	1.246521	-0.967078	H	3.582040	-2.126109	0.871581
H	-3.716298	1.916127	-1.809417	O	4.586787	-1.988285	-0.917818
H	-4.348360	1.333749	-0.268765	C	4.603233	-3.413076	-1.088691
H	-2.618198	1.626257	-0.462220	H	3.670774	-3.760704	-1.539641
C	-4.609565	-0.694692	-2.183492	H	5.437269	-3.635281	-1.752258
H	-4.855567	-0.024662	-3.014806	H	4.756297	-3.913458	-0.126357

3b'-Glu-C-rad

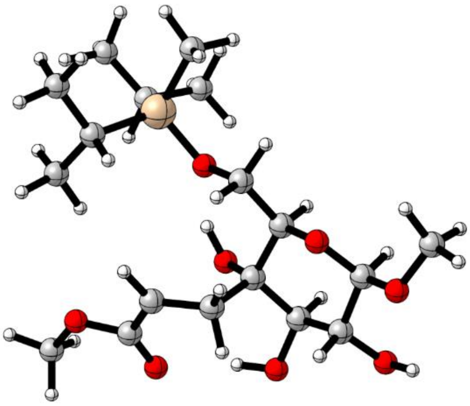

C	3.659301	-1.456955	-0.044256	H	-4.363106	-2.010596	-2.556315
C	3.980412	0.023304	0.181062	H	-5.431272	-1.045217	-1.534036
C	2.856301	0.703784	0.959196	C	-5.017975	-1.209833	1.627441
C	1.479155	0.424026	0.321374	H	-5.454312	-0.906103	2.585365
C	1.328704	-1.105868	0.129824	H	-5.537289	-0.655749	0.840973
O	2.389070	-1.606818	-0.679060	H	-5.243988	-2.272500	1.489909
O	5.185514	0.153071	0.929693	C	-2.814297	-1.666555	2.802620
O	3.137254	2.095512	1.064974	H	-3.243212	-1.333887	3.754560
O	0.526082	0.872259	1.283861	H	-2.952001	-2.751663	2.745078
C	0.029283	-1.539064	-0.545195	H	-1.739922	-1.467410	2.828944
O	-1.064368	-1.019822	0.206834	C	-2.821855	-3.258263	-0.385186
H	5.884307	-0.285446	0.428779	H	-3.869926	-3.569149	-0.421291
H	2.314440	2.606658	0.970100	H	-2.368017	-3.513550	-1.347044
Si	-2.702310	-1.418853	-0.029829	C	1.353206	1.182997	-1.064105
H	4.085648	0.497439	-0.802269	H	2.206768	1.859872	-1.154838
H	2.826476	0.266728	1.966309	H	1.432136	0.458428	-1.873583
H	-2.323743	-3.841433	0.394437	C	0.115418	1.984036	-1.194217
H	1.363461	-1.560216	1.130302	C	-0.010016	3.230625	-0.472262
H	-0.309766	0.415918	1.079303	O	-1.215528	3.809665	-0.649288
H	0.001540	-1.175235	-1.578297	O	0.869735	3.729490	0.226212
H	0.006251	-2.632924	-0.573472	C	-1.440942	5.052645	0.047493
C	-3.339540	-0.383979	-1.487388	H	-0.727626	5.808742	-0.283864
H	-2.506863	-0.387356	-2.205938	H	-2.455900	5.347763	-0.206918
C	-3.498229	-0.954614	1.620402	H	-1.342954	4.906260	1.124174
H	-3.335369	0.125393	1.734955	H	-0.722431	1.649977	-1.791169
C	-3.611207	1.078937	-1.086590	H	3.642243	-1.990871	0.920673
H	-3.834458	1.688206	-1.969542	O	4.640929	-1.976980	-0.876591
H	-4.473599	1.148823	-0.416250	C	4.665618	-3.409893	-0.941907
H	-2.762017	1.541526	-0.577508	H	3.734735	-3.795920	-1.364136
C	-4.561993	-0.997172	-2.197585	H	5.500037	-3.676173	-1.588674
H	-4.848413	-0.388918	-3.062902	H	4.822920	-3.837436	0.054381

TS14

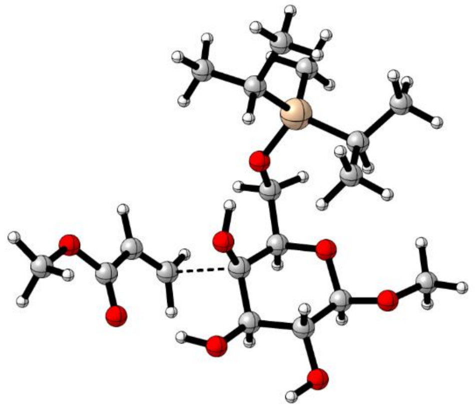

C	0.174834	3.150733	0.205511
C	-1.004898	2.879222	-0.732549
C	-2.025622	1.945563	-0.048616
C	-1.297563	0.724565	0.445120
C	-0.154967	1.071027	1.372377
O	0.759789	1.922789	0.645500
O	-1.606540	4.122811	-1.068150
O	-3.044843	1.692347	-0.998037
C	0.639768	-0.114010	1.906517
O	0.974422	-1.035760	0.865179
H	-2.407252	3.925399	-1.570247
H	-3.600871	0.944843	-0.700463
Si	2.551934	-1.427504	0.343102
H	-0.619856	2.373914	-1.626609
H	-2.429739	2.486356	0.820031
H	3.035156	-2.774647	2.377118
H	-0.551896	1.624447	2.234280
H	1.533479	0.270533	2.406471
H	0.036433	-0.643596	2.647607
C	3.301308	0.115817	-0.457120
H	3.057452	0.922794	0.245602
C	2.218615	-2.841170	-0.867306
H	1.564768	-2.415354	-1.640428
C	2.617965	0.457812	-1.793920
H	2.994302	1.409248	-2.186337
H	2.812103	-0.309065	-2.551065
H	1.536126	0.558591	-1.677451
C	4.833952	0.063977	-0.603675
H	5.211885	1.014704	-0.996659
H	5.332241	-0.118887	0.352555
H	5.151017	-0.720783	-1.297299
C	3.500376	-3.345810	-1.557468
H	3.266108	-4.158441	-2.254008
H	4.000718	-2.559523	-2.128610
H	4.218858	-3.738681	-0.830191
C	1.461754	-4.001531	-0.193439
H	1.210094	-4.775662	-0.927076
H	2.072445	-4.474802	0.582814
H	0.531976	-3.664284	0.271900
C	3.549129	-1.975820	1.835746
H	4.527626	-2.352591	1.523741
H	3.720992	-1.147166	2.528903
O	-1.109579	-0.228257	-0.489865
H	-0.367244	-0.798001	-0.182542
C	-2.922813	-0.241687	1.963865
H	-3.536903	0.619430	1.734352
H	-2.306262	-0.175112	2.850615
C	-3.183418	-1.445090	1.389912
C	-4.048910	-1.533188	0.225043
O	-4.247707	-2.804715	-0.175025
O	-4.553903	-0.578231	-0.365266
C	-5.067822	-2.993480	-1.347399
H	-5.091019	-4.067515	-1.515016
H	-6.074832	-2.612326	-1.172677
H	-4.627896	-2.481687	-2.204456
H	-2.686810	-2.352044	1.710864
H	-0.167732	3.720613	1.086971
O	1.142707	3.858639	-0.492558
C	2.225178	4.329733	0.319442
H	2.854722	4.938366	-0.328043
H	1.850377	4.943973	1.146456
H	2.806367	3.495060	0.718990

3b-Gal-C-rad

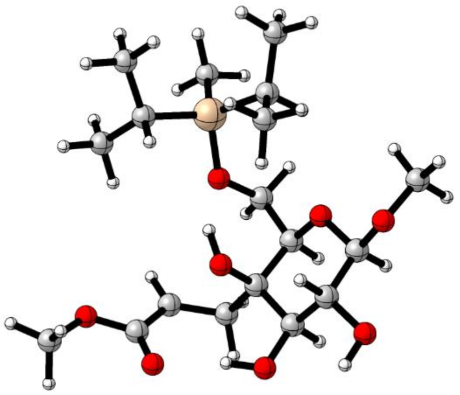

C	-0.506275	3.184701	0.095803	C	3.678433	-2.834383	-1.512369
C	-1.722070	2.684941	-0.679947	H	3.505900	-3.623364	-2.252779
C	-2.520017	1.694589	0.174655	H	4.184499	-2.010805	-2.023258
C	-1.624113	0.551172	0.668003	H	4.369264	-3.236523	-0.763731
C	-0.421971	1.187338	1.415599	C	1.601337	-3.597692	-0.264881
O	0.273691	2.078683	0.548711	H	1.420456	-4.363248	-1.027950
O	-2.533682	3.807886	-1.020728	H	2.185303	-4.066487	0.534497
O	-3.649286	1.250385	-0.576905	H	0.635124	-3.305743	0.154314
C	0.597861	0.182847	1.943154	C	3.633757	-1.531017	1.853555
O	0.990166	-0.744373	0.929979	H	4.645871	-1.790404	1.529732
H	-3.342752	3.457512	-1.414407	H	3.718169	-0.727578	2.591472
H	-3.615932	0.284994	-0.700616	O	-1.183832	-0.139722	-0.498751
Si	2.582395	-1.003831	0.389922	H	-0.352429	-0.584264	-0.253018
H	-1.365091	2.173378	-1.578907	C	-2.448135	-0.389420	1.643328
H	-2.900676	2.234751	1.051229	H	-3.497966	-0.087602	1.593797
H	3.201061	-2.404997	2.348225	H	-2.117599	-0.236142	2.671023
H	-0.815006	1.738887	2.283270	C	-2.348551	-1.821962	1.277568
H	1.452838	0.747066	2.328990	C	-3.069848	-2.316625	0.126173
H	0.166230	-0.377958	2.776271	O	-2.817996	-3.618180	-0.120539
C	3.209905	0.618122	-0.364145	O	-3.835382	-1.646334	-0.563699
H	2.883936	1.388151	0.347952	C	-3.481561	-4.195709	-1.264673
C	2.348797	-2.394376	-0.870612	H	-3.150482	-5.230560	-1.300261
H	1.716851	-1.969536	-1.662058	H	-4.564276	-4.143544	-1.141743
C	2.524148	0.926123	-1.708070	H	-3.191977	-3.669057	-2.175171
H	2.820241	1.916322	-2.072769	H	-1.691423	-2.502923	1.802430
H	2.804159	0.196922	-2.475586	H	-0.830933	3.776230	0.970703
H	1.436300	0.926436	-1.609940	O	0.286810	3.952113	-0.746694
C	4.743297	0.704664	-0.481094	C	1.384428	4.597467	-0.091126
H	5.043510	1.691027	-0.852873	H	1.861982	5.230612	-0.837674
H	5.238977	0.549585	0.481332	H	1.029481	5.218251	0.740089
H	5.139706	-0.037872	-1.180801	H	2.102729	3.863766	0.282793

4. NMR spectra

^{1}H NMR (500 MHz, $CDCl_3$) of compound **SI-1**

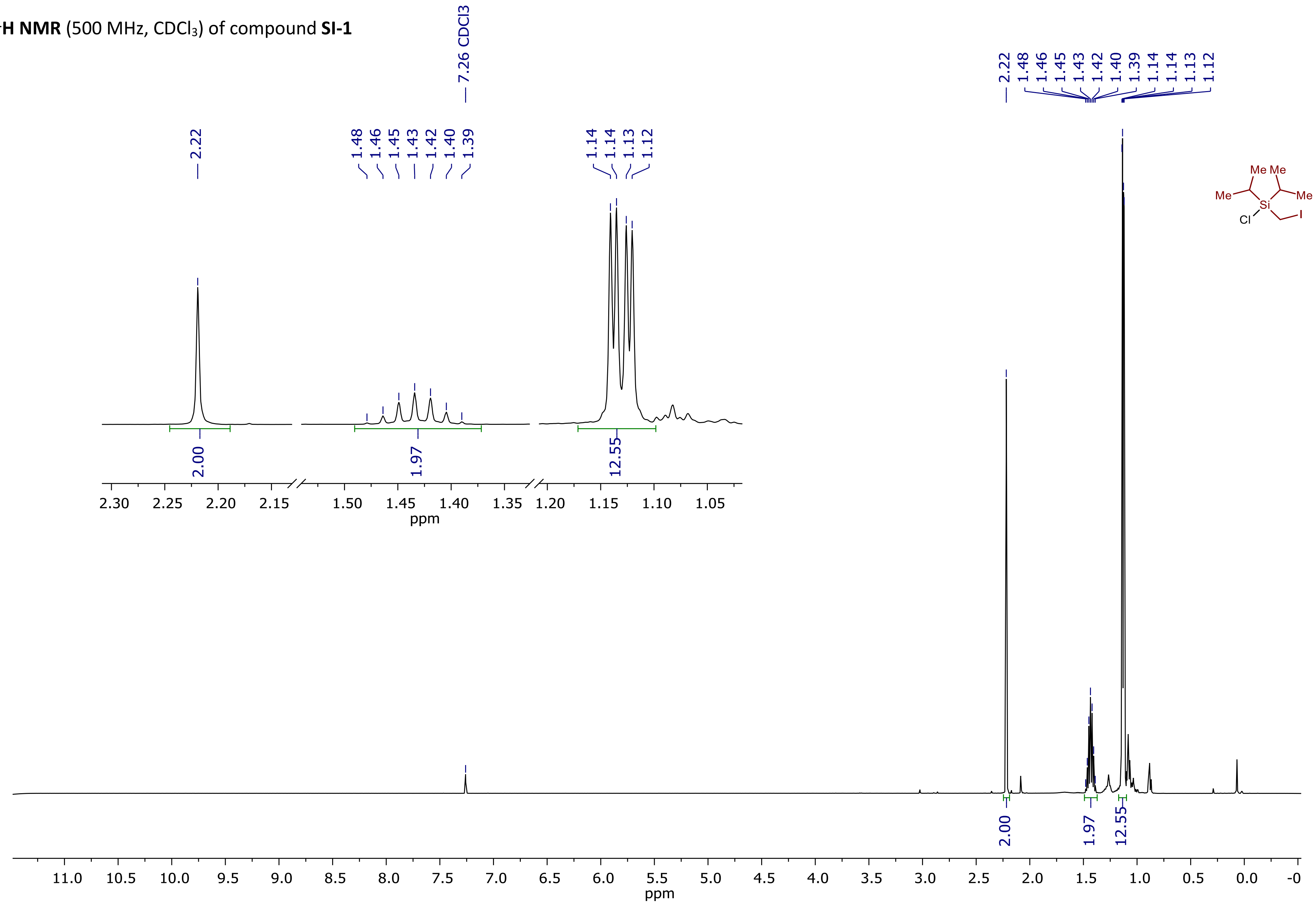

$^{13}C\{^{1}H\}$ **NMR** (126 MHz, $CDCl_3$) of compound **SI-1**

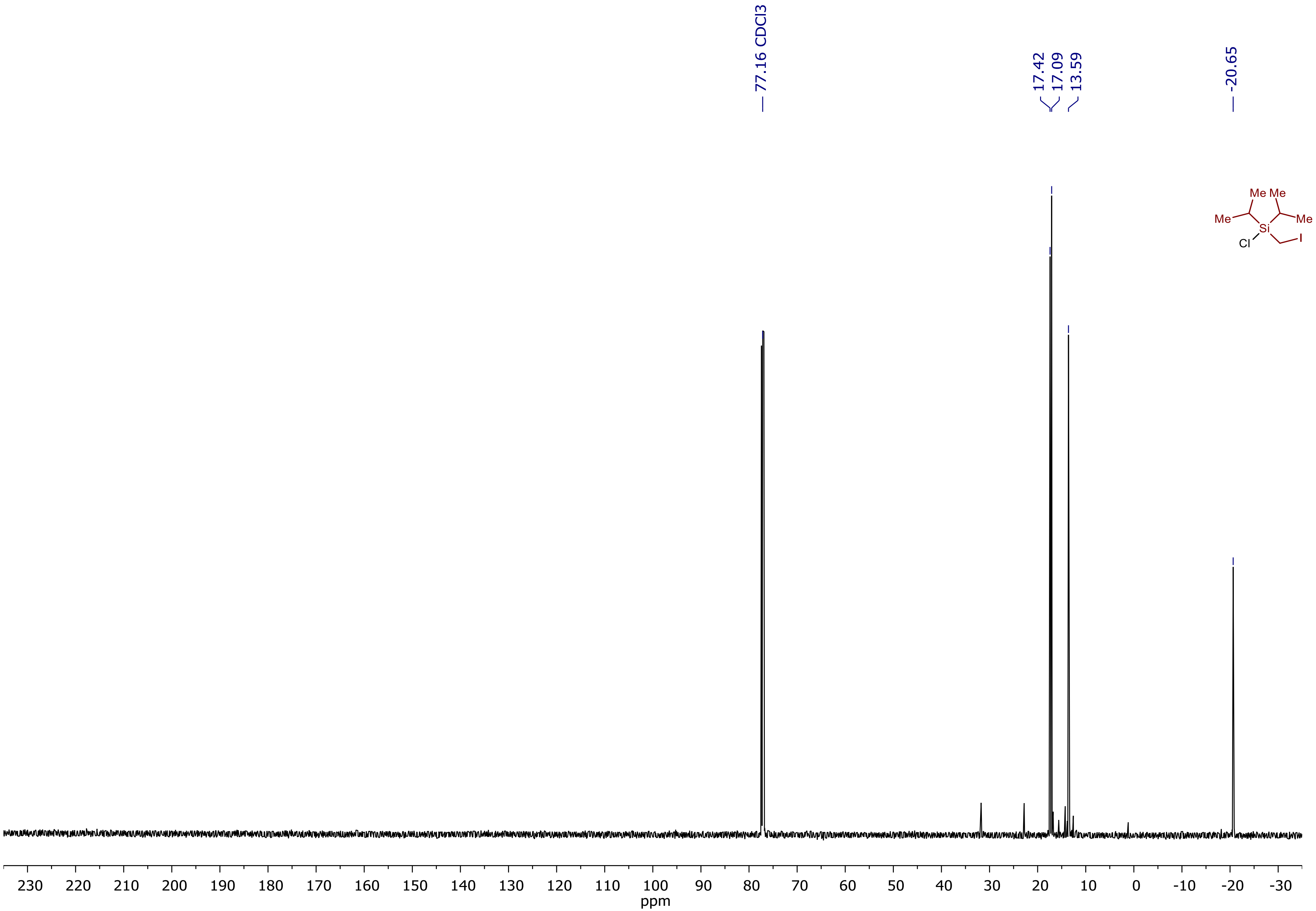

HSQC of compound **SI-1**

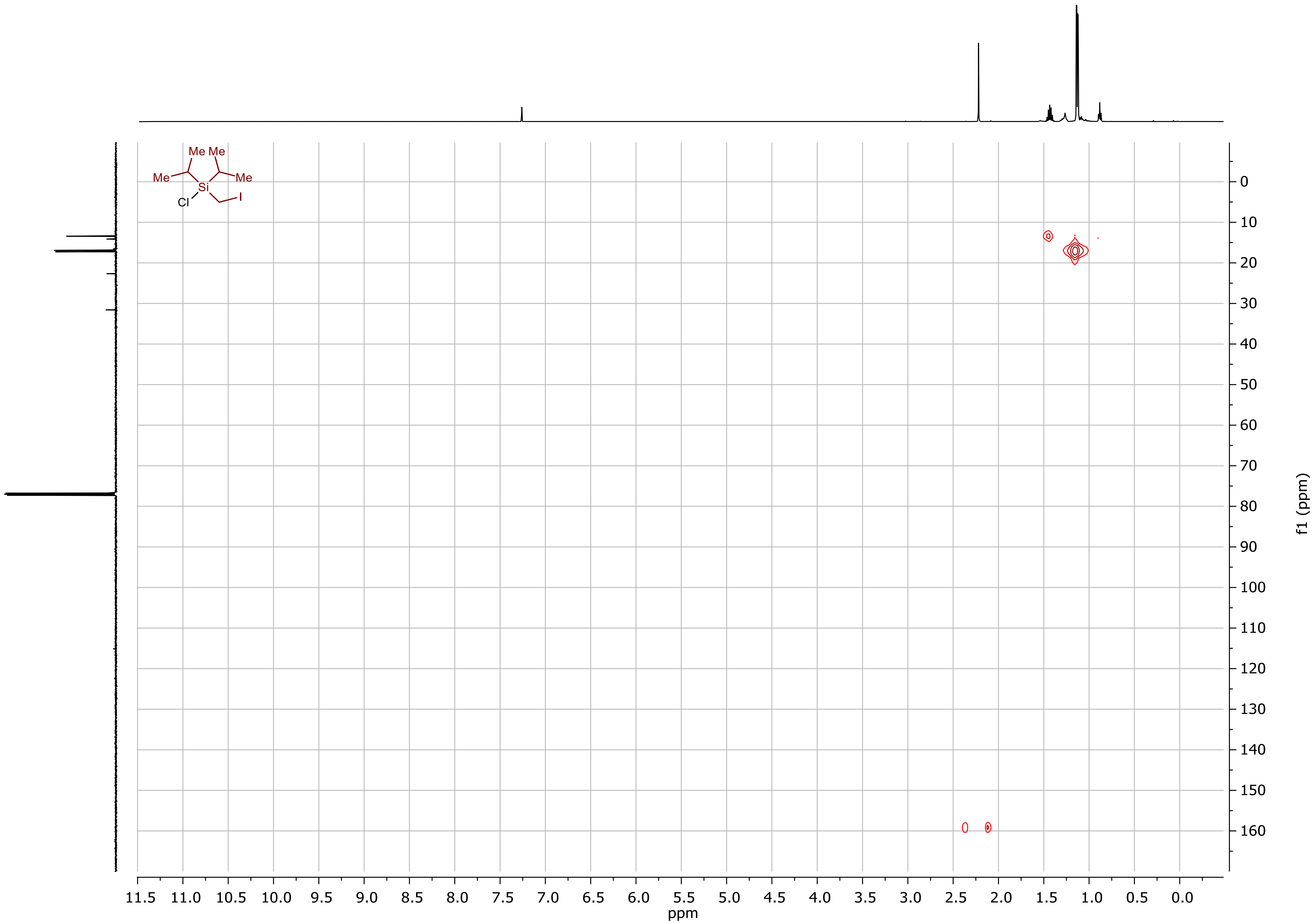

^{1}H NMR (500 MHz, $CDCl_3$) of compound **2a**

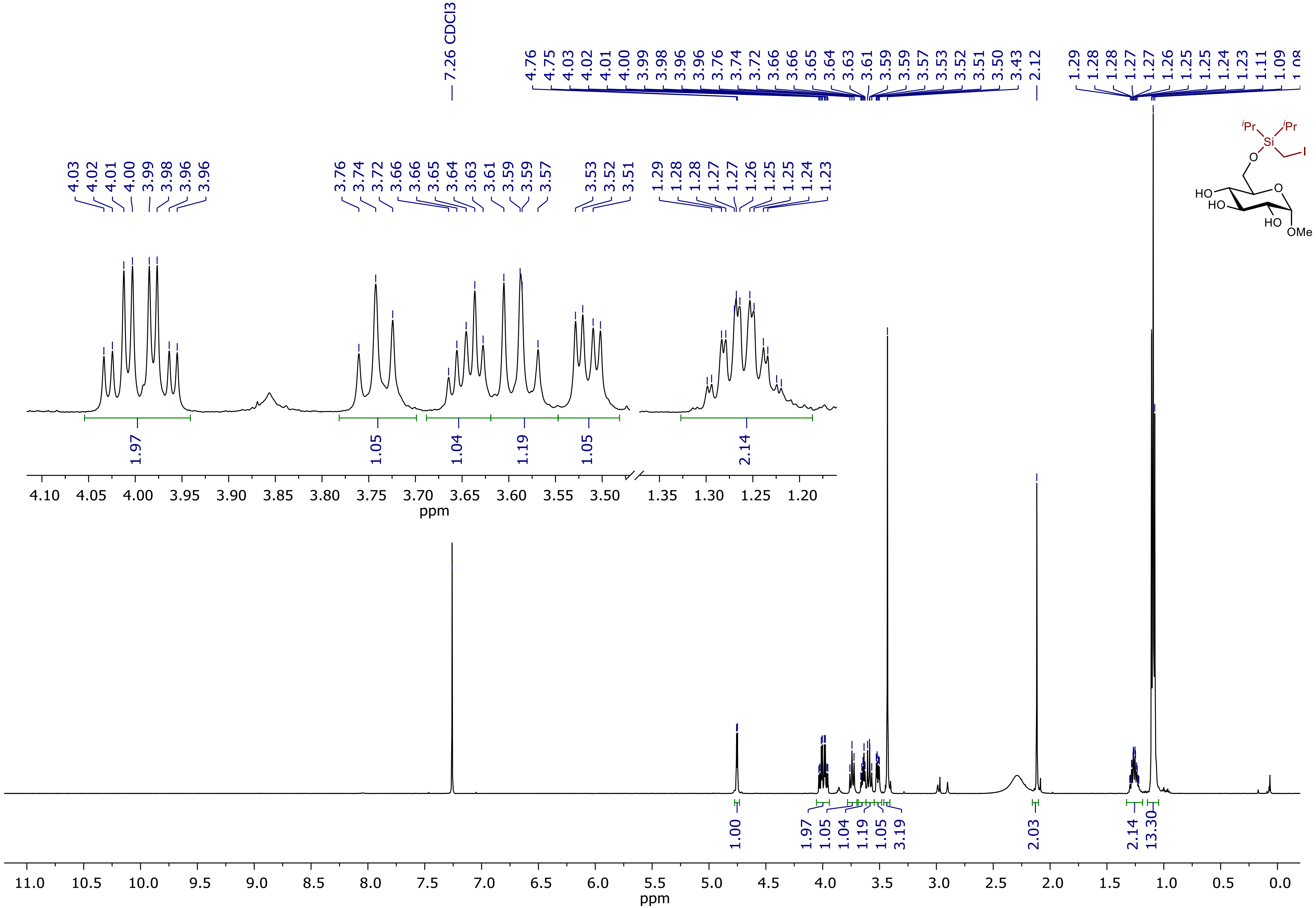

$^{13}C\{^1H\}$ NMR (126 MHz, $CDCl_3$) of compound **2a**

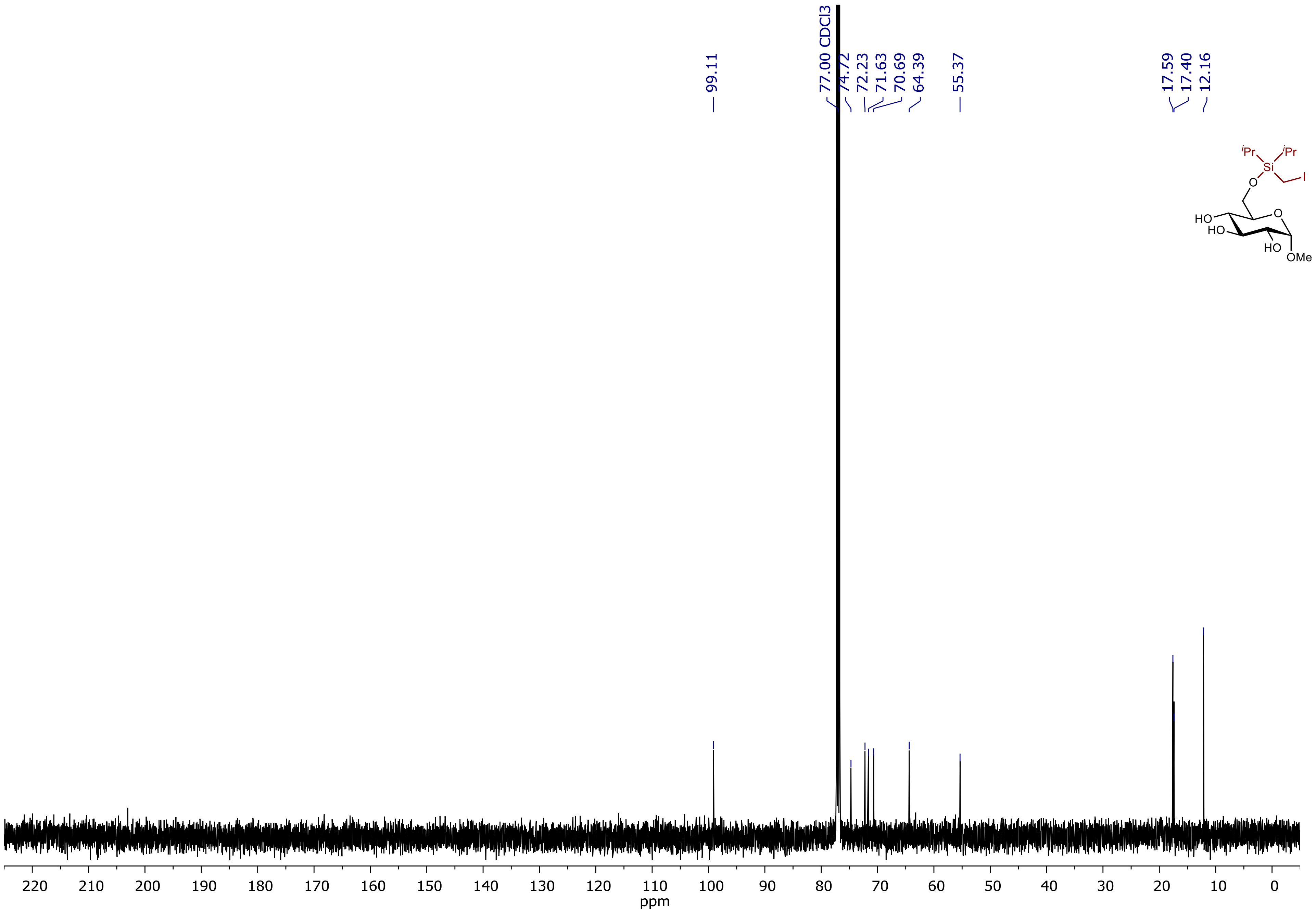

COSY of compound **2a**

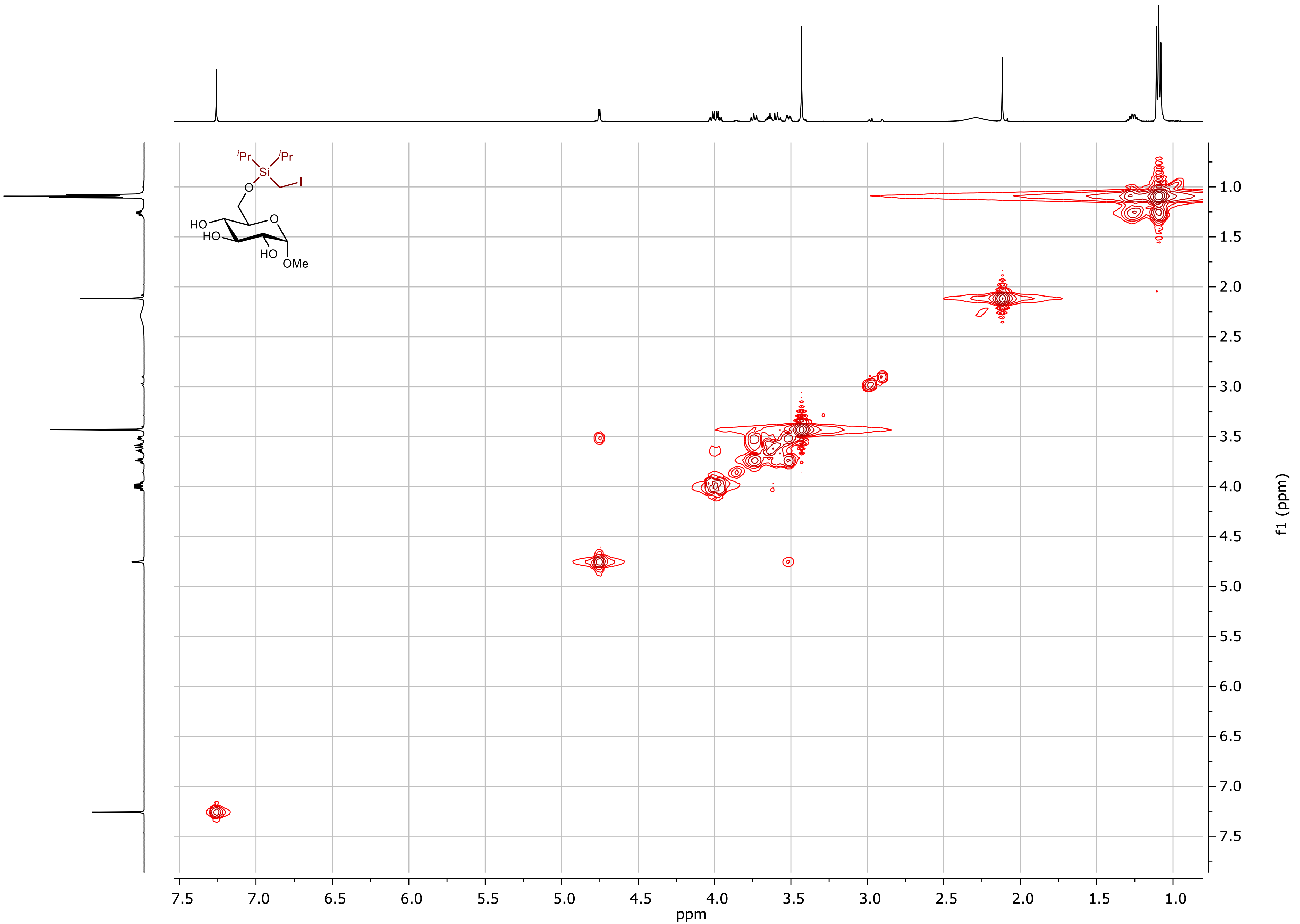

HSQC of compound **2a**

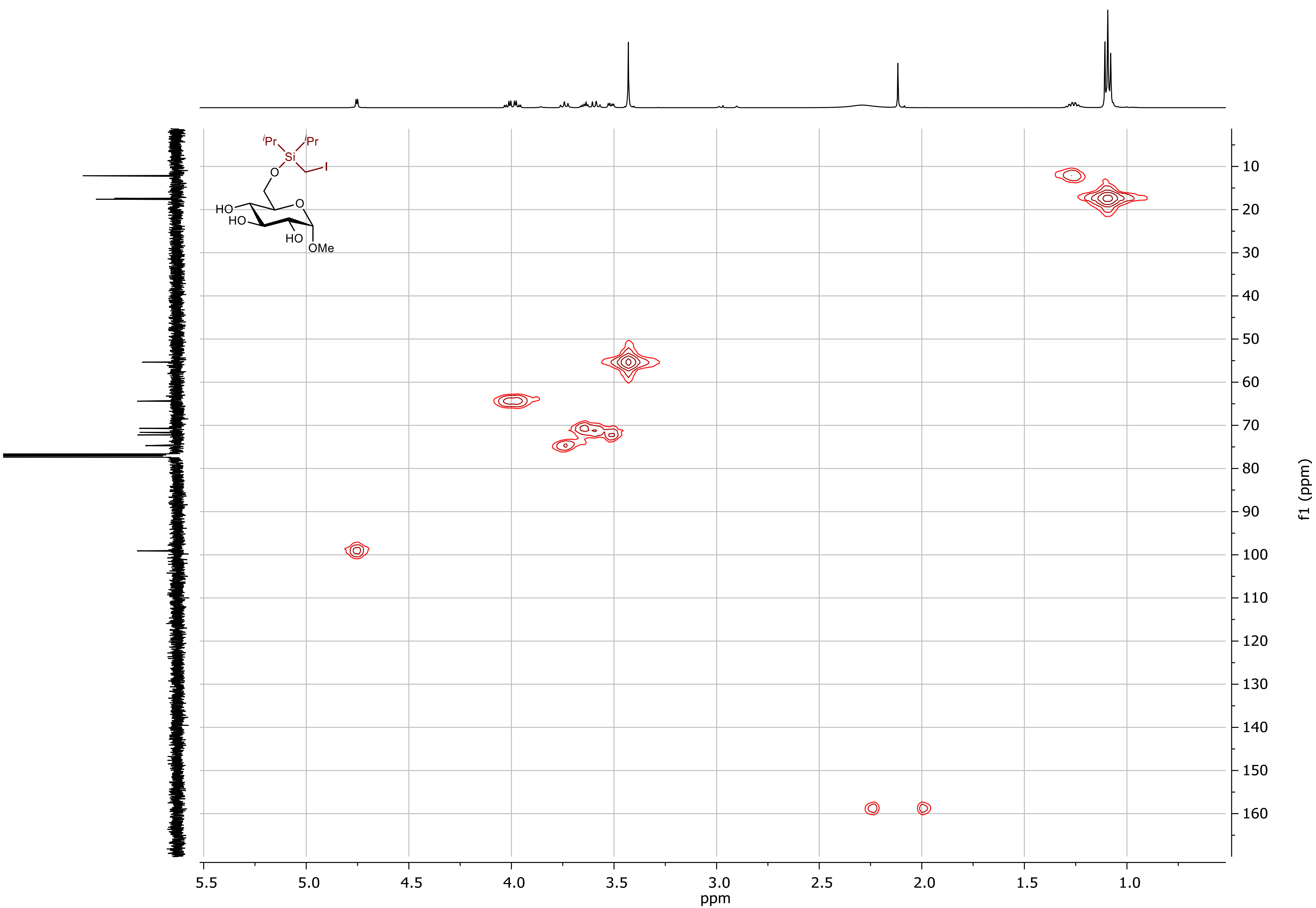

^{1}H NMR (500 MHz, $CDCl_3$) of compound **2b**

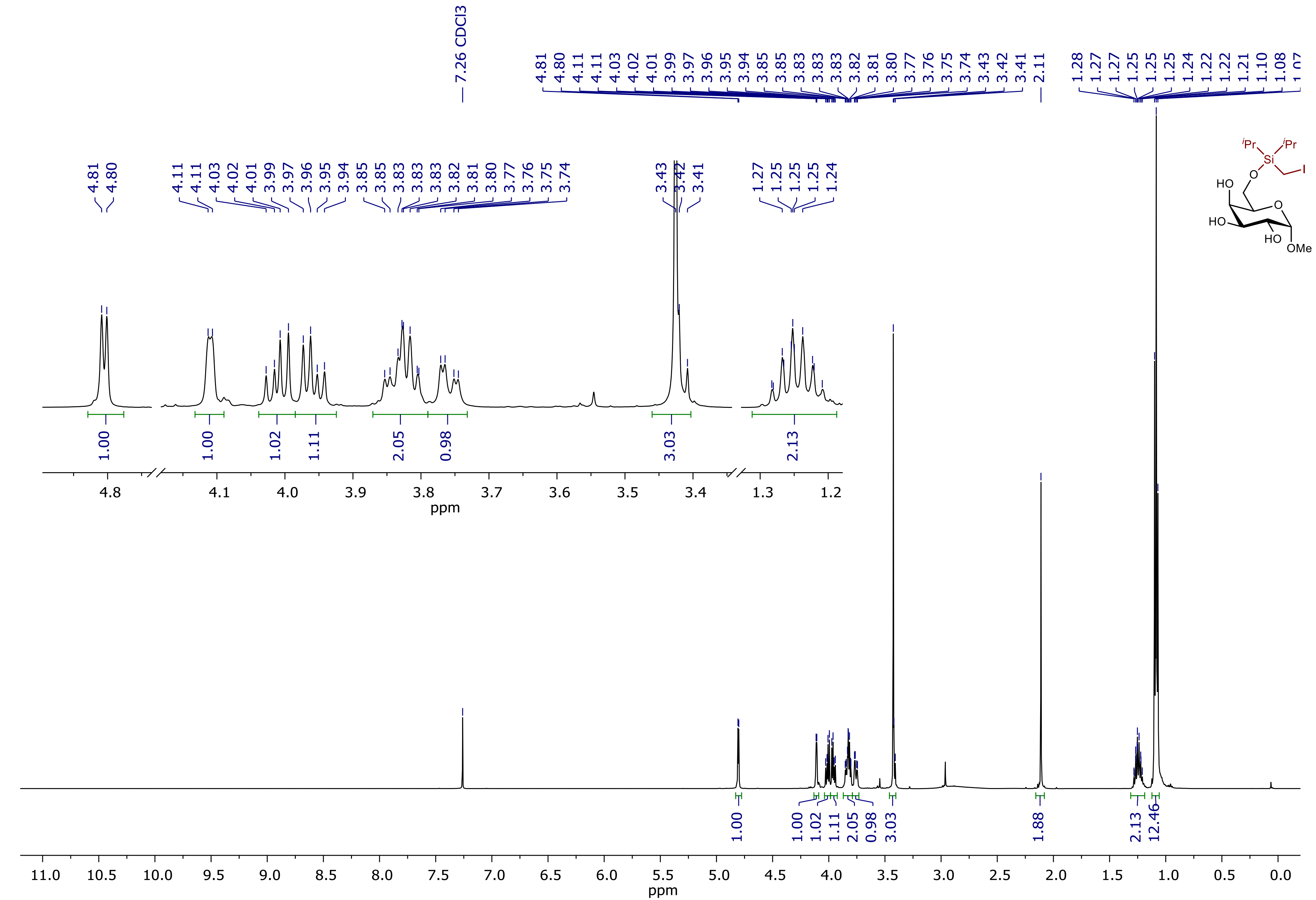

^{13}C{^{1}H} NMR (126 MHz, $CDCl_3$) of compound **2b**

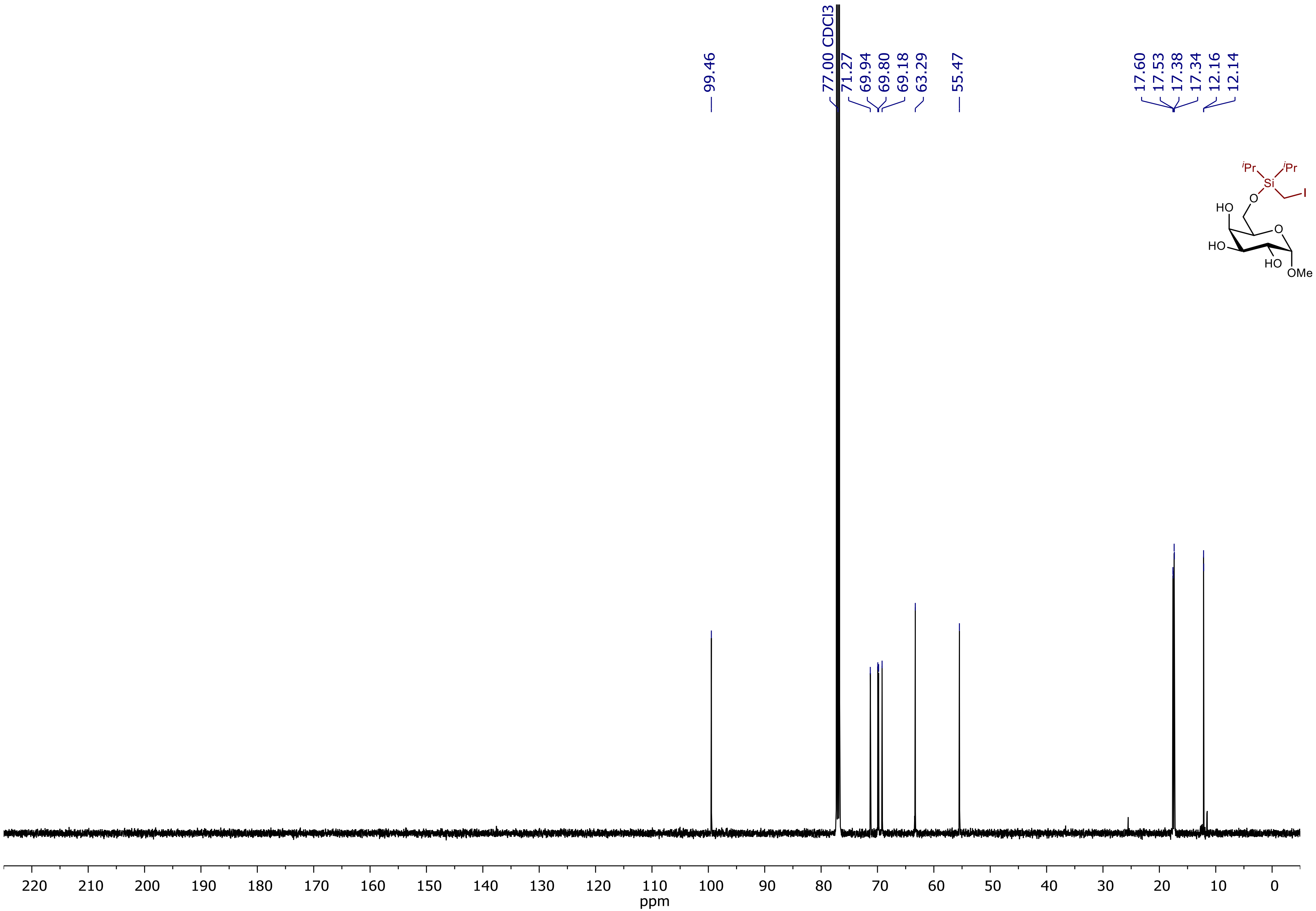

COSY of compound **2b**

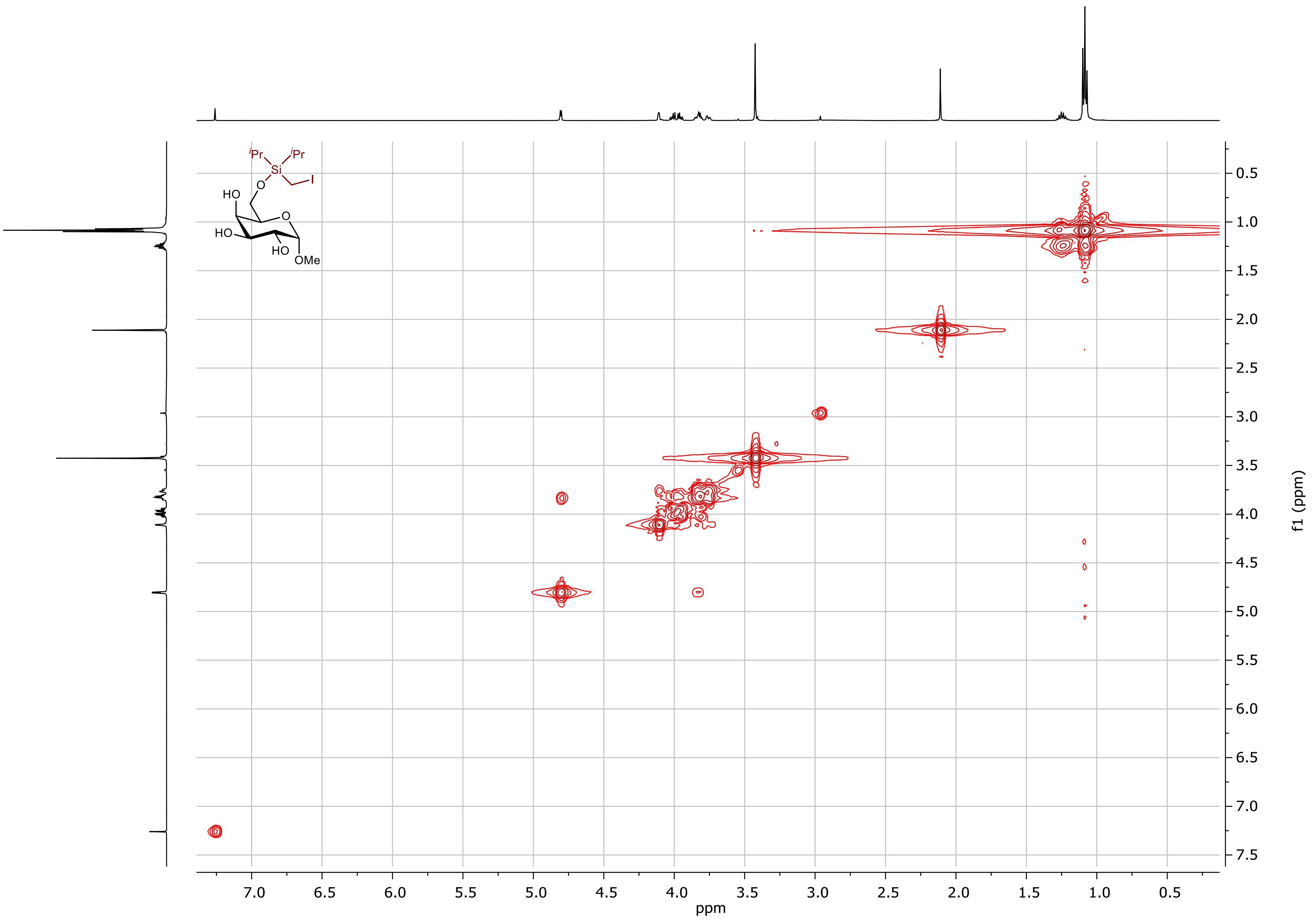

HSQC of compound **2b**

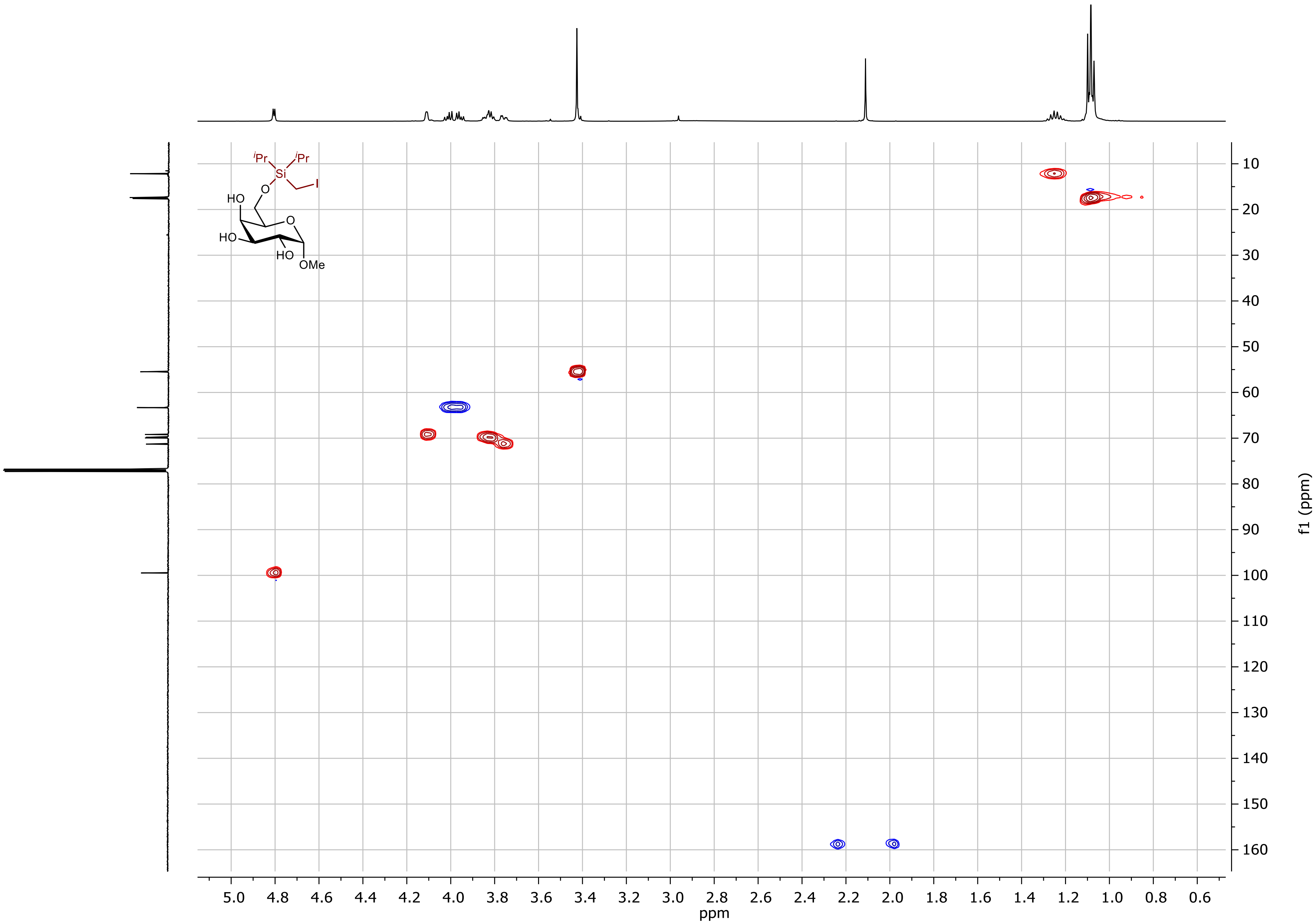

^{1}**H NMR** (500 MHz, $CDCl_3$) of compound **2c**

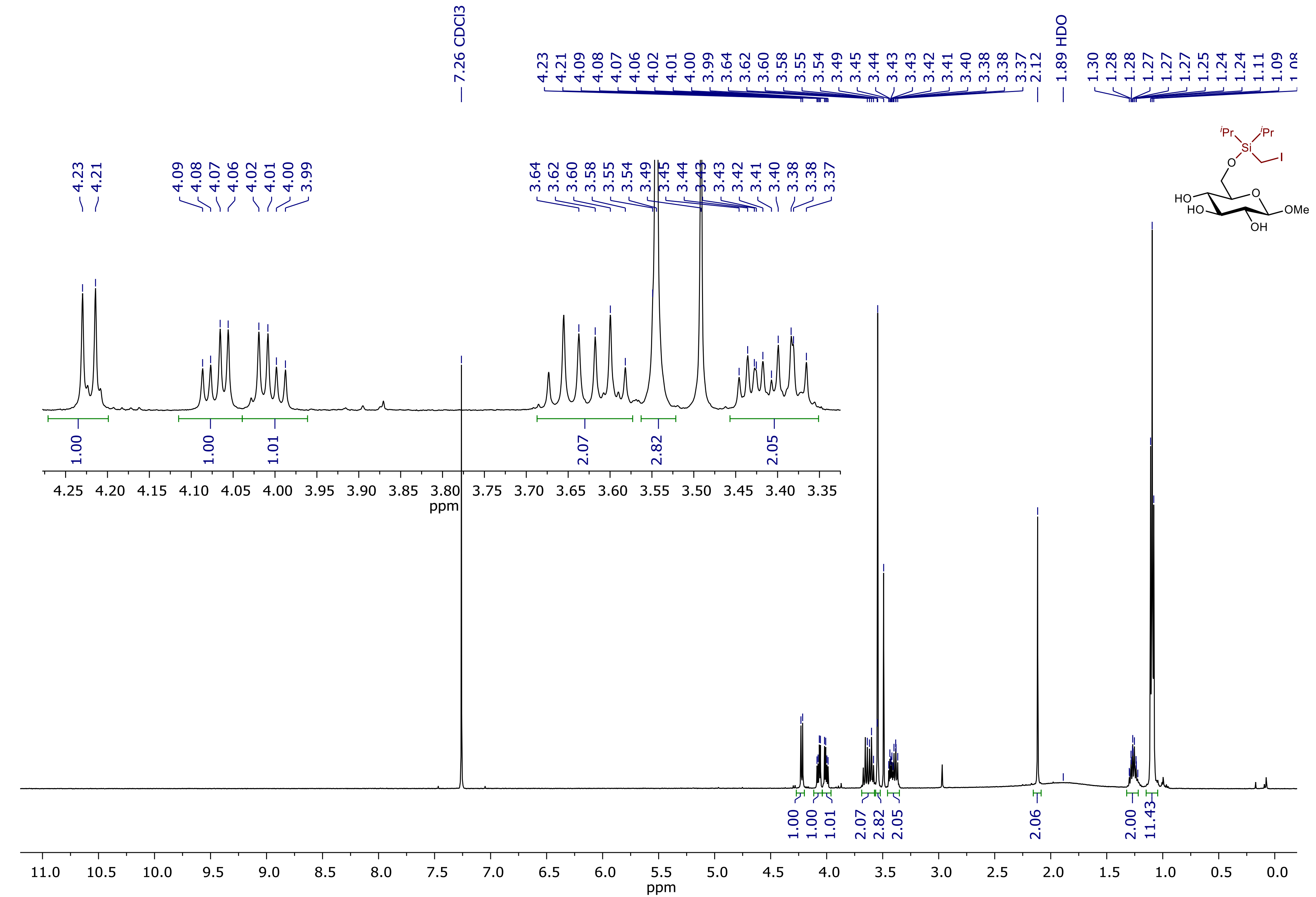

^{13}C{^{1}H} NMR (126 MHz, $CDCl_3$) of compound **2c**

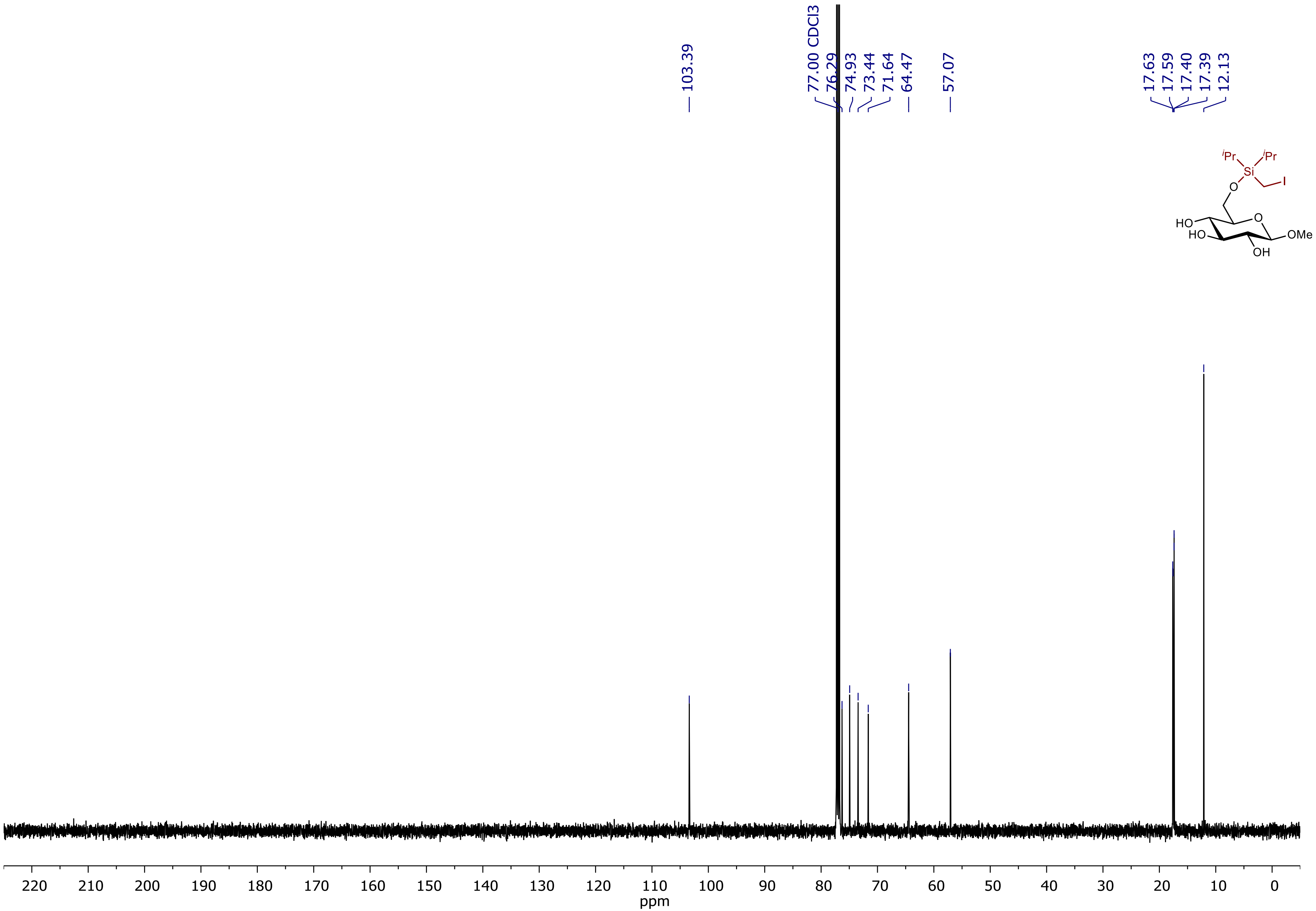

COSY of compound **2c**

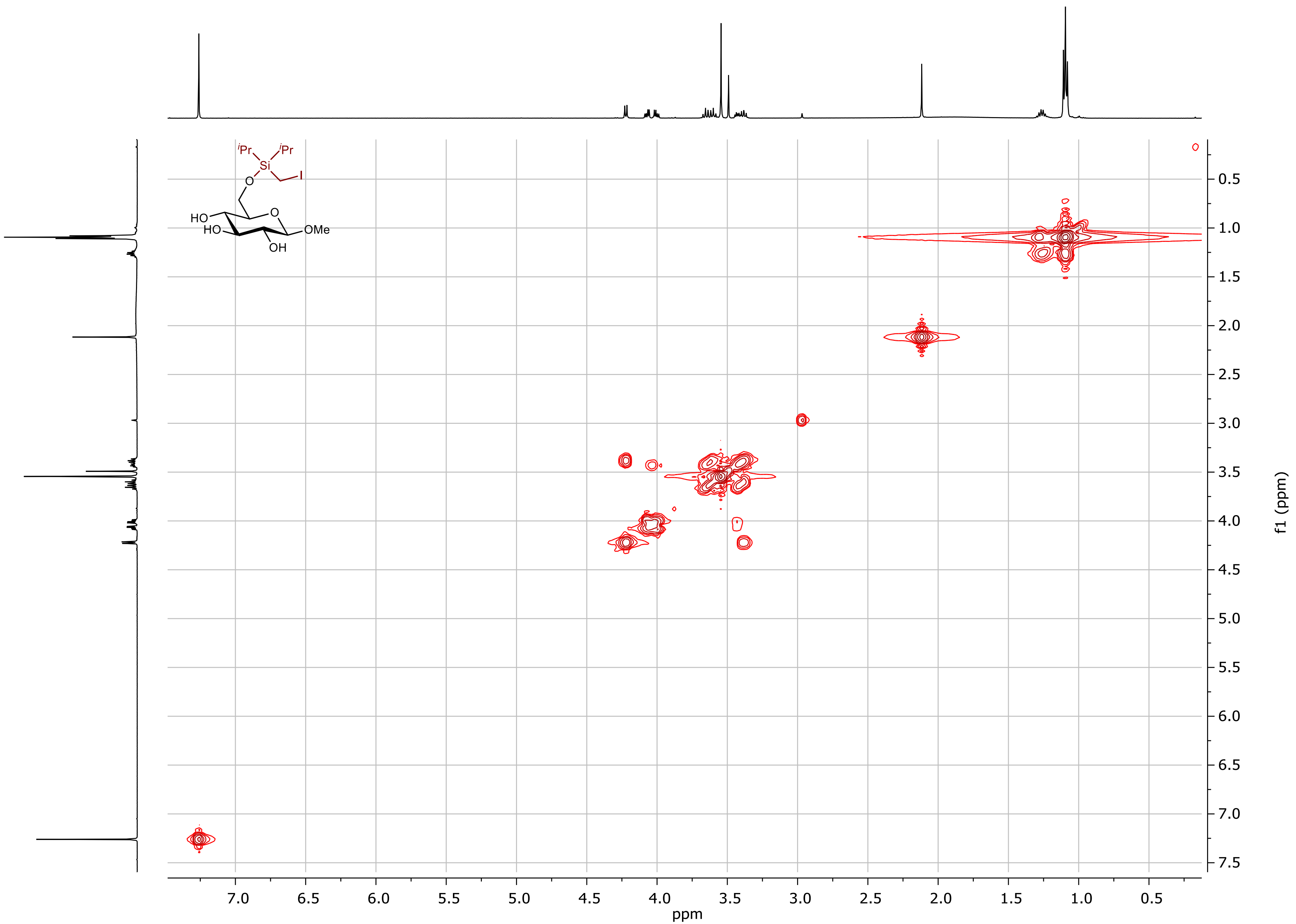

HSQC of compound **2c**

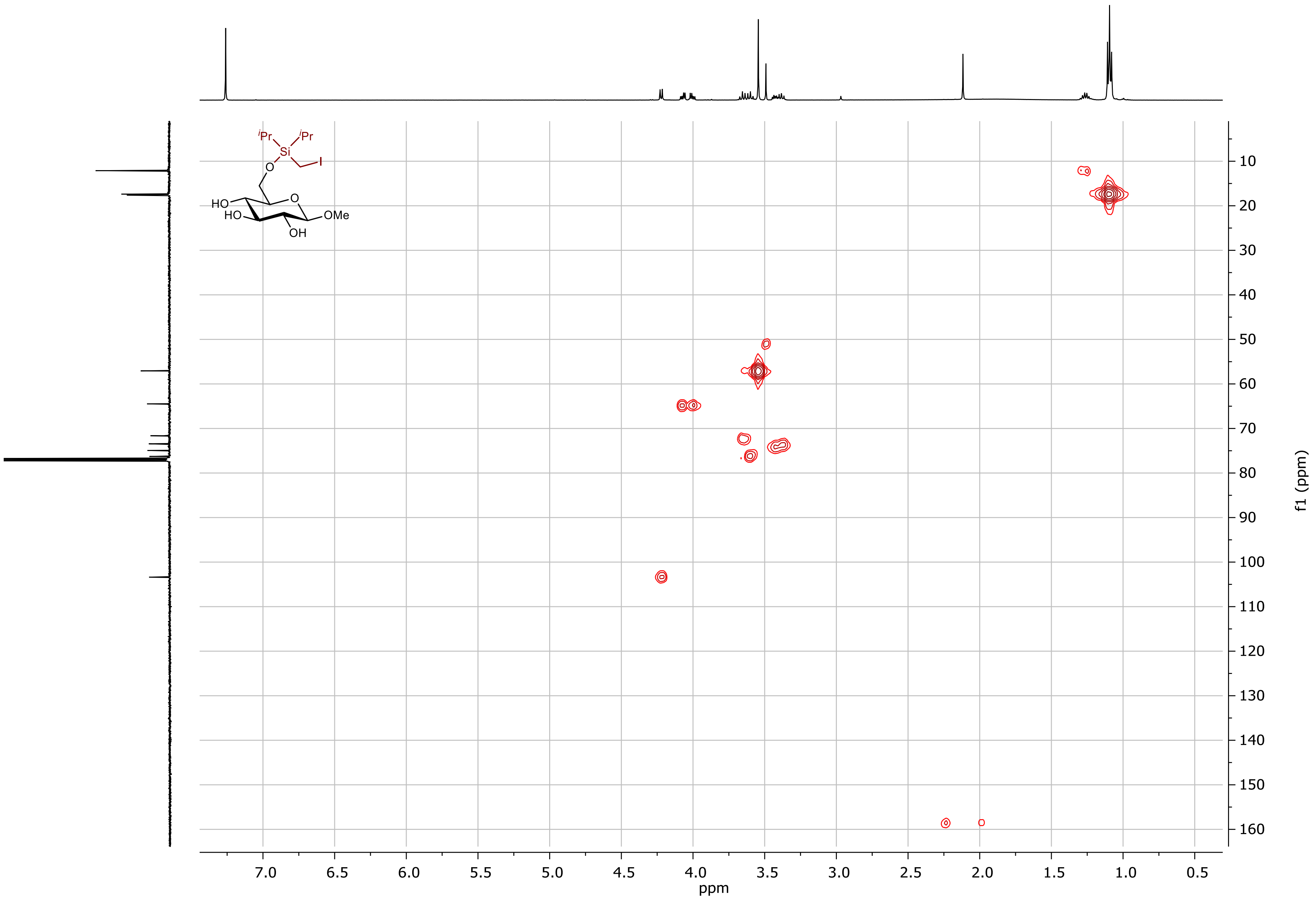

^{1}H NMR (500 MHz, $CDCl_3$) of compound **2d**

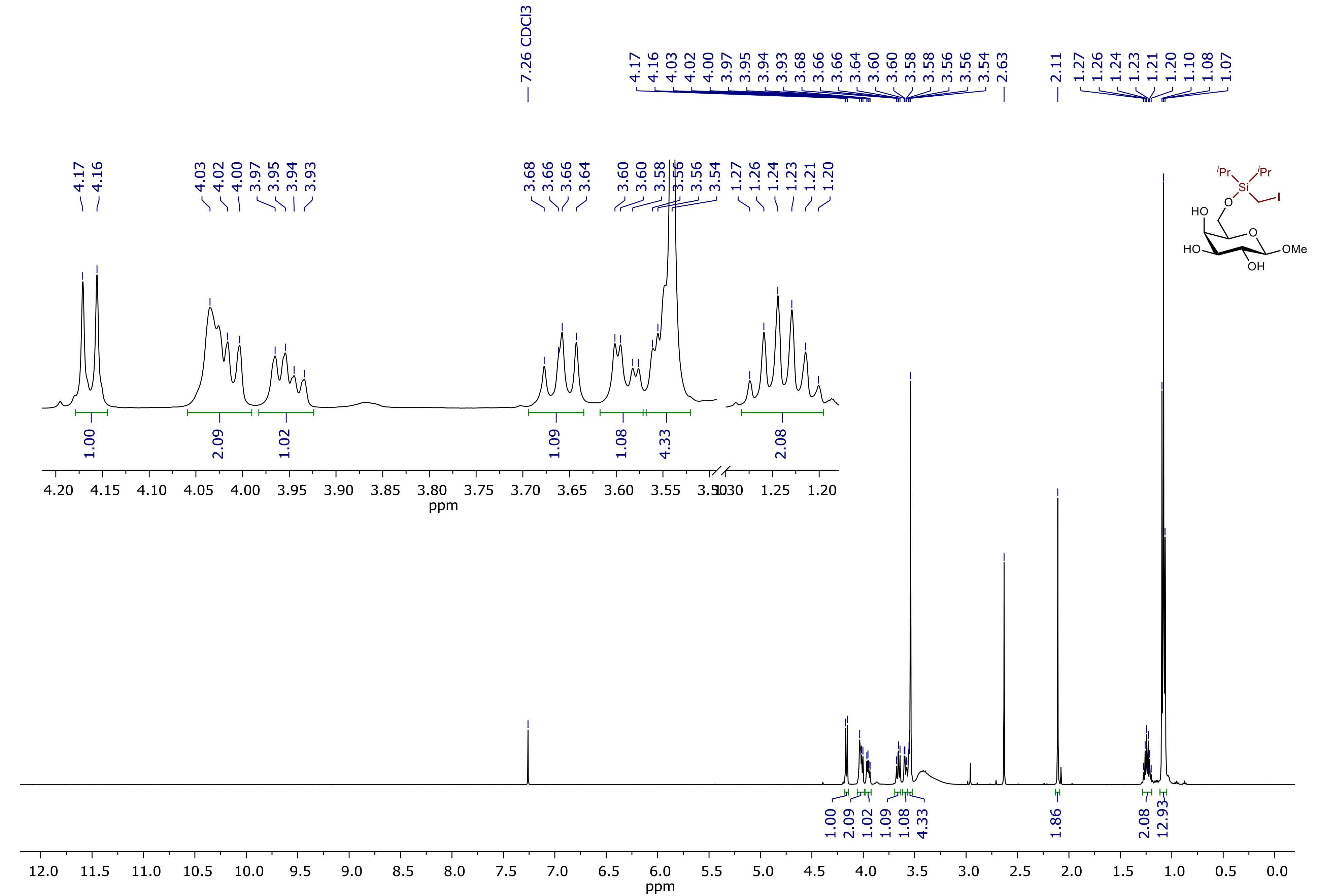

$^{13}C\{^1H\}$ NMR (126 MHz, $CDCl_3$) of compound **2d**

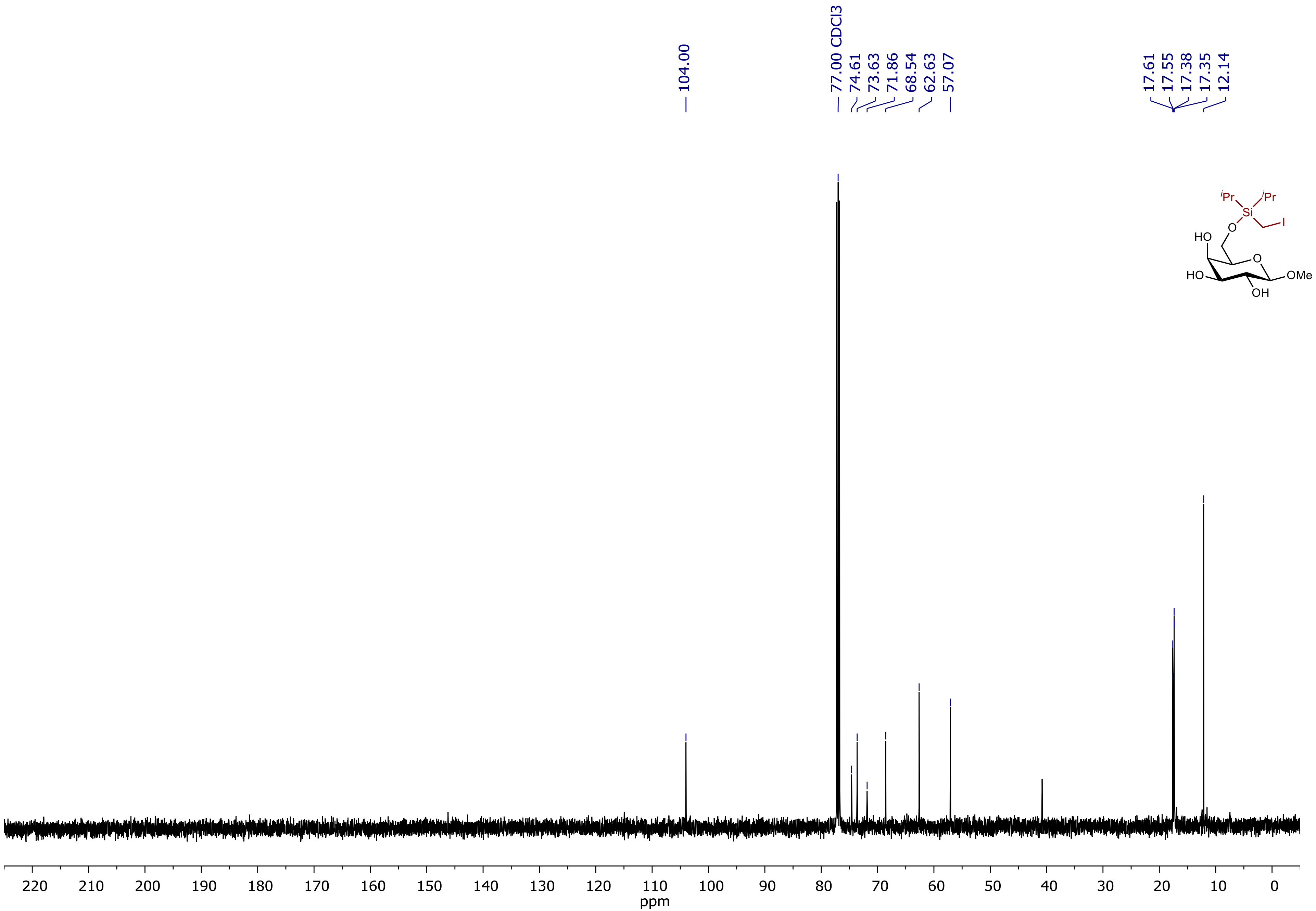

COSY of compound **2d**

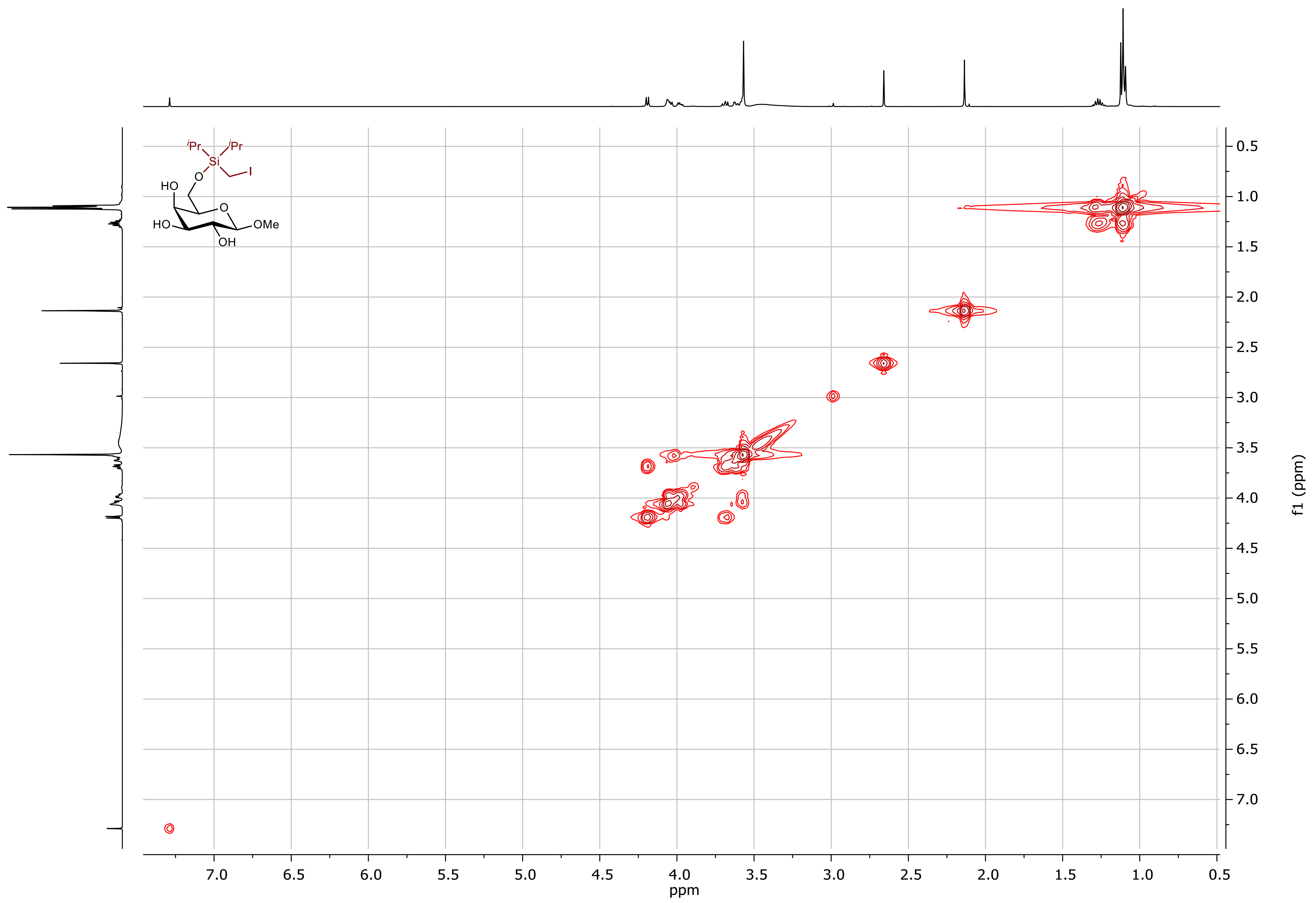

HSQC of compound **2d**

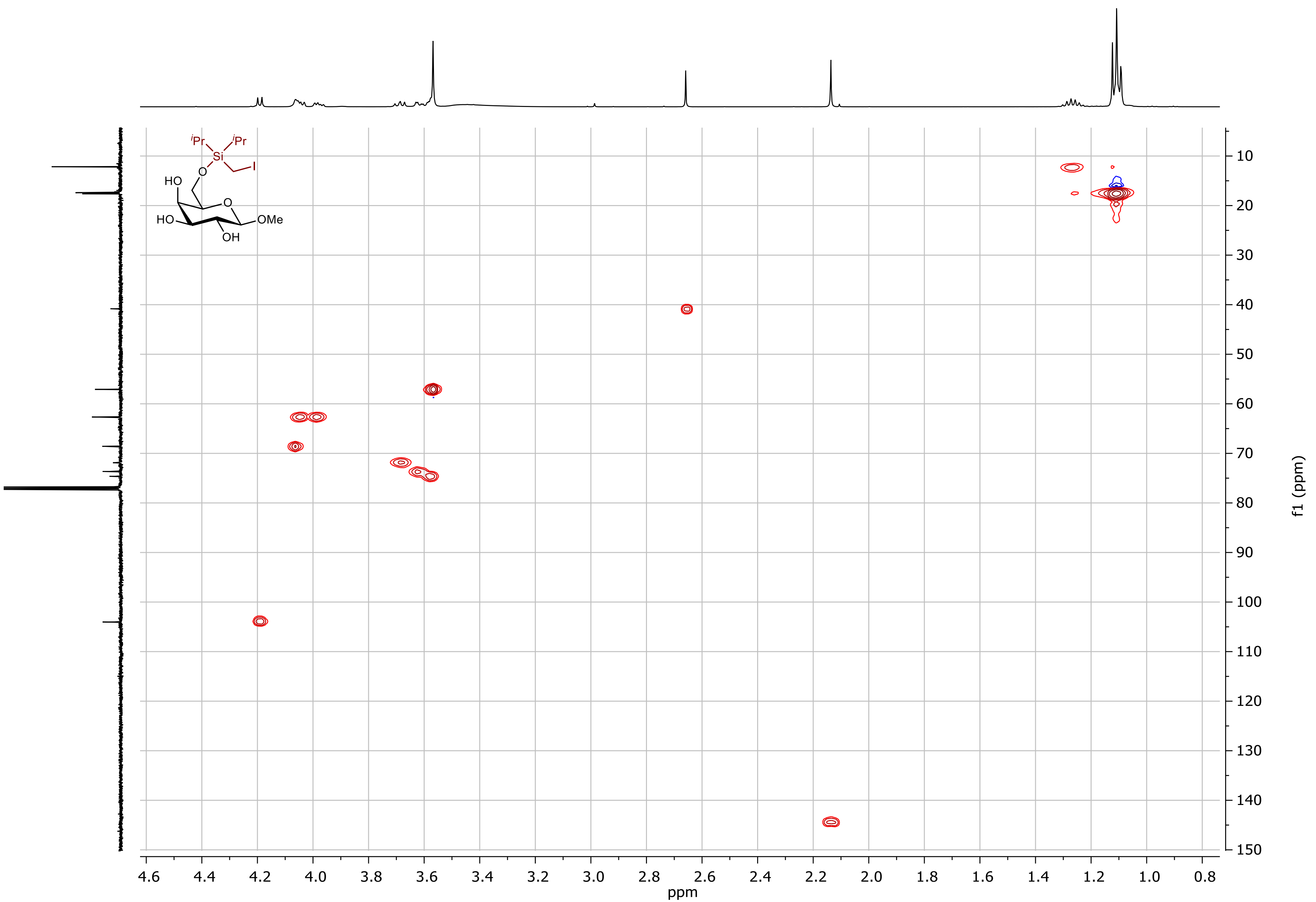

^{1}H NMR (500 MHz, $CDCl_3$) of compound **2e**

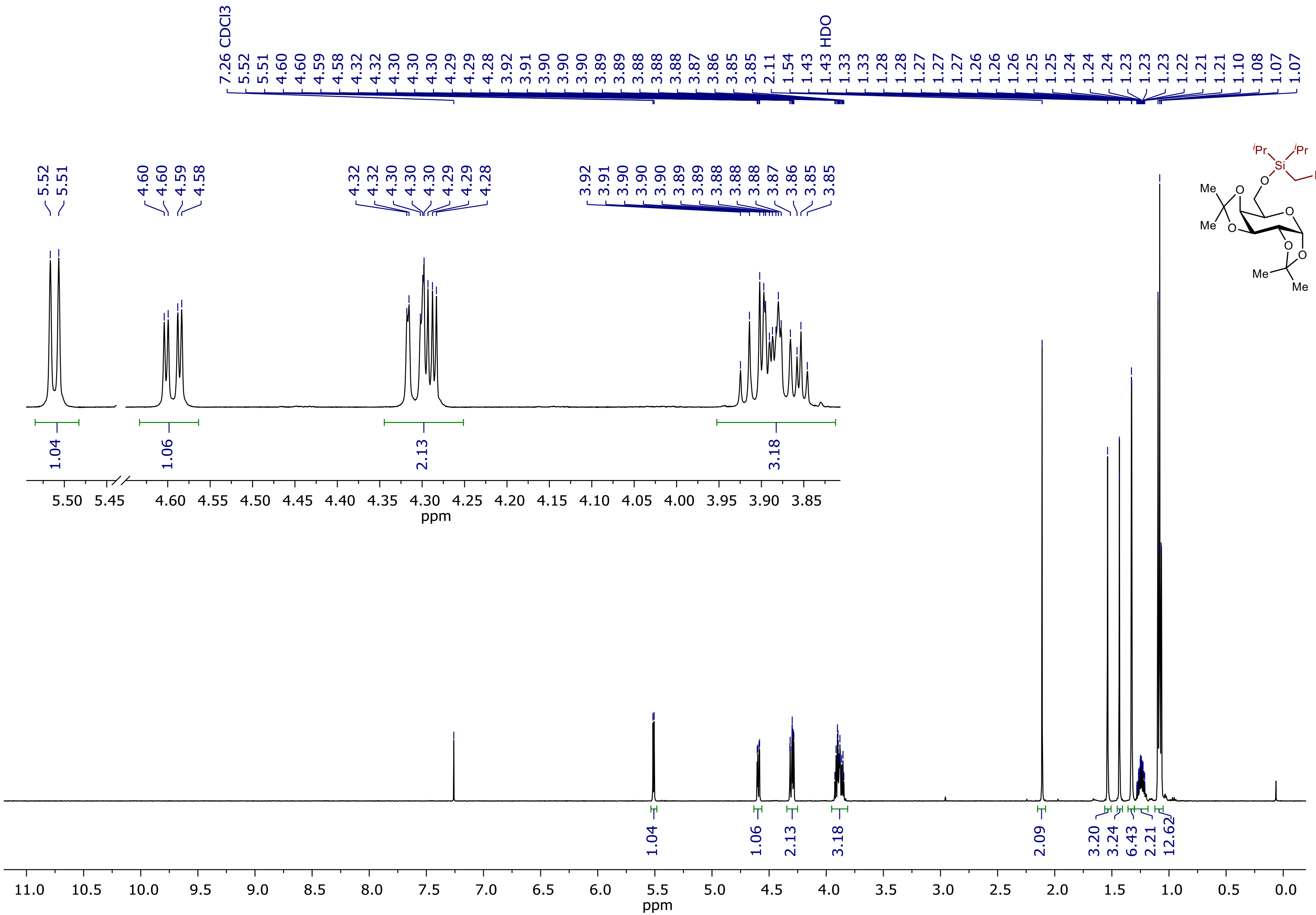

$^{13}C\{^{1}H\}$ **NMR** (126 MHz, $CDCl_3$) of compound **2e**

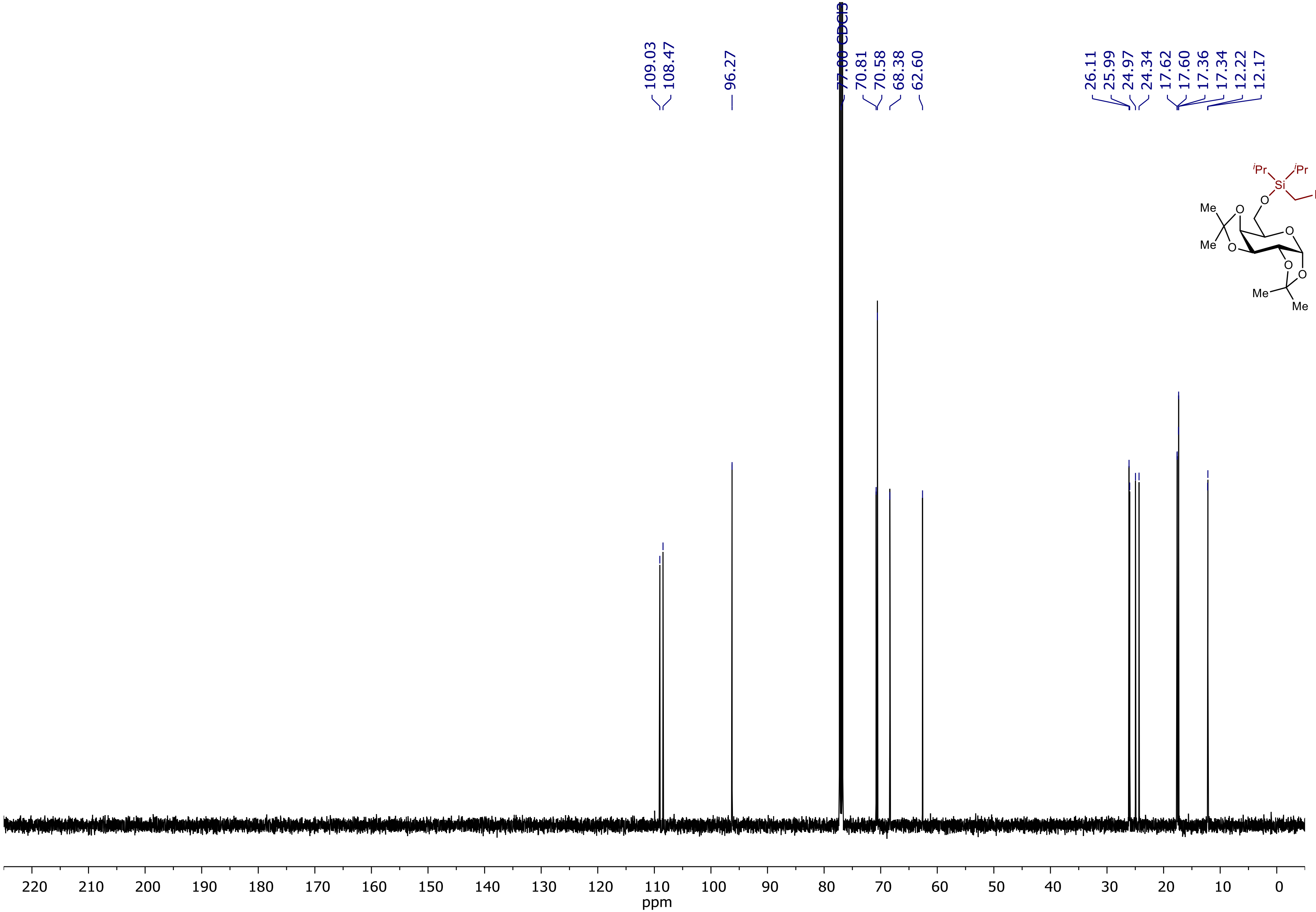

COSY of compound **2e**

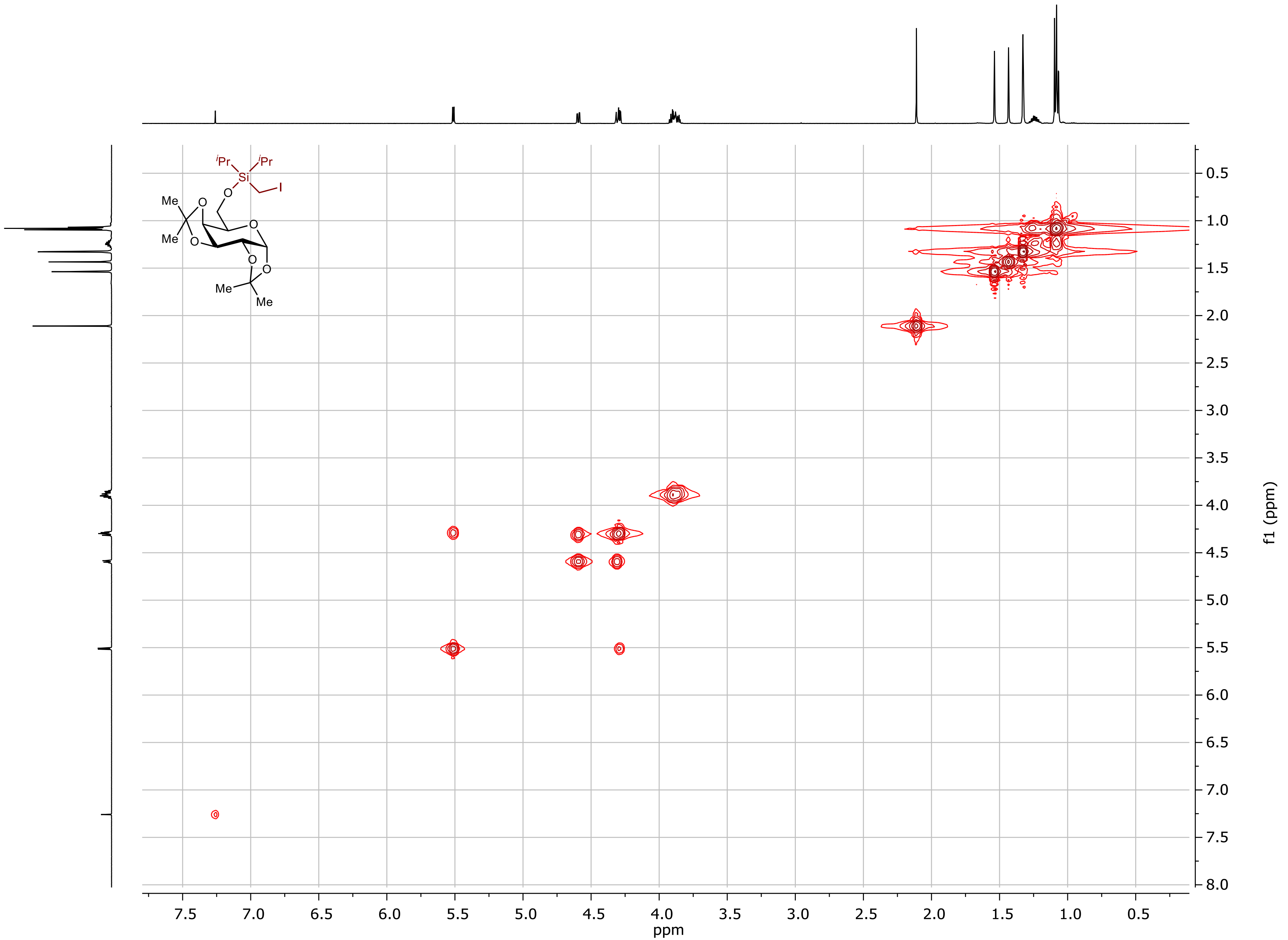

HSQC of compound **2e**

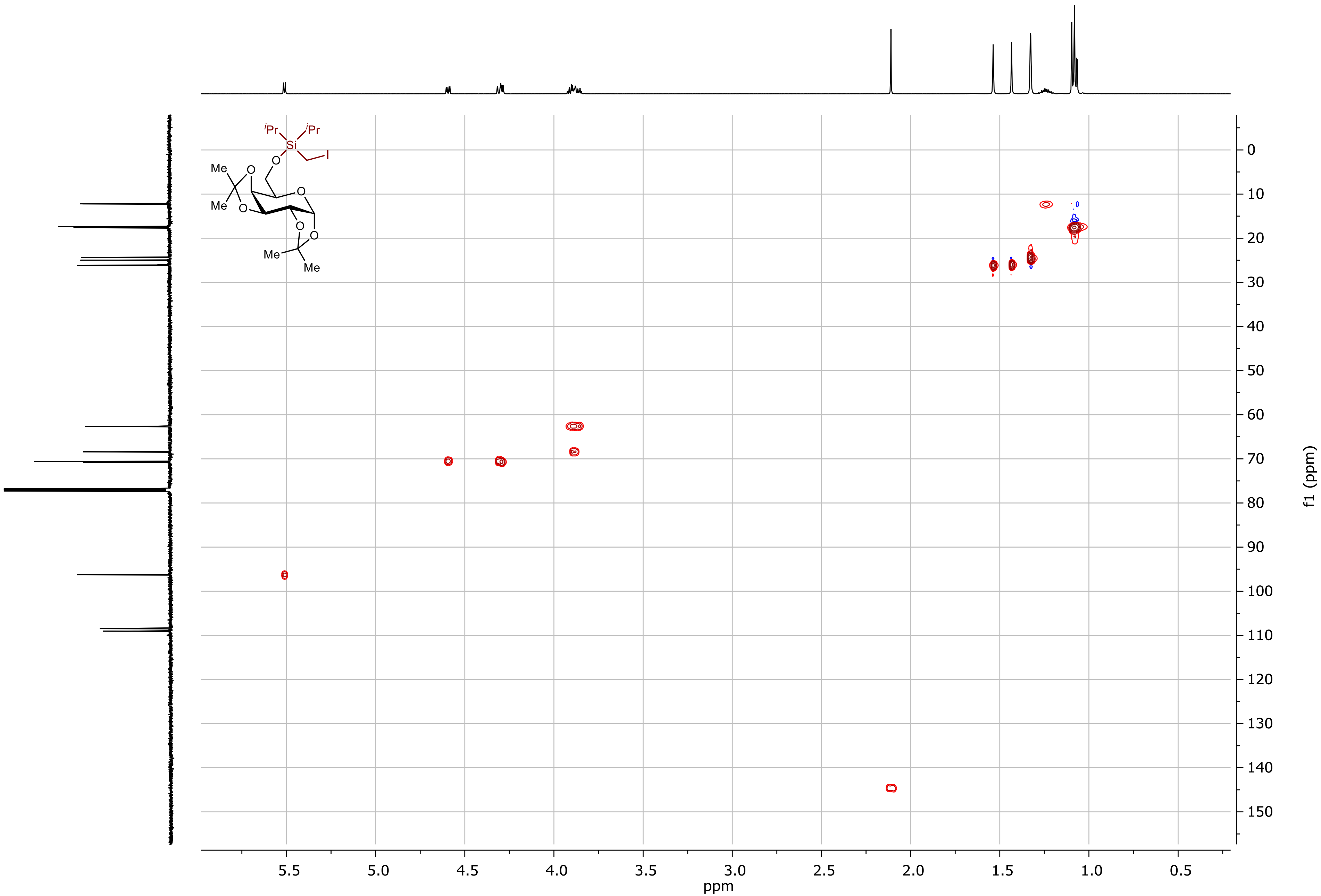

^{1}H NMR (500 MHz, $CDCl_3$) of compound **2f**

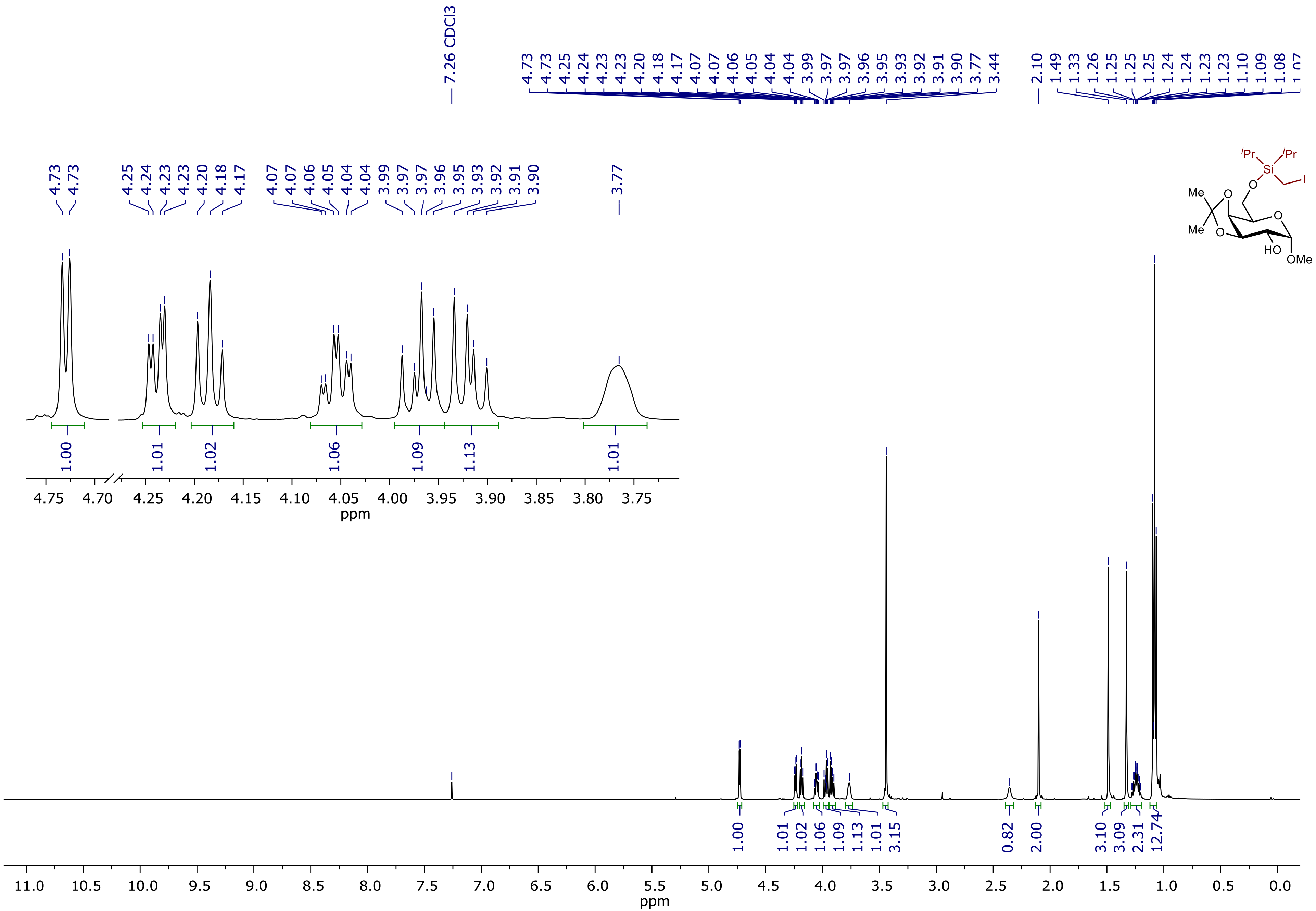

$^{13}C\{^1H\}$ **NMR** (126 MHz, $CDCl_3$) of compound **2f**

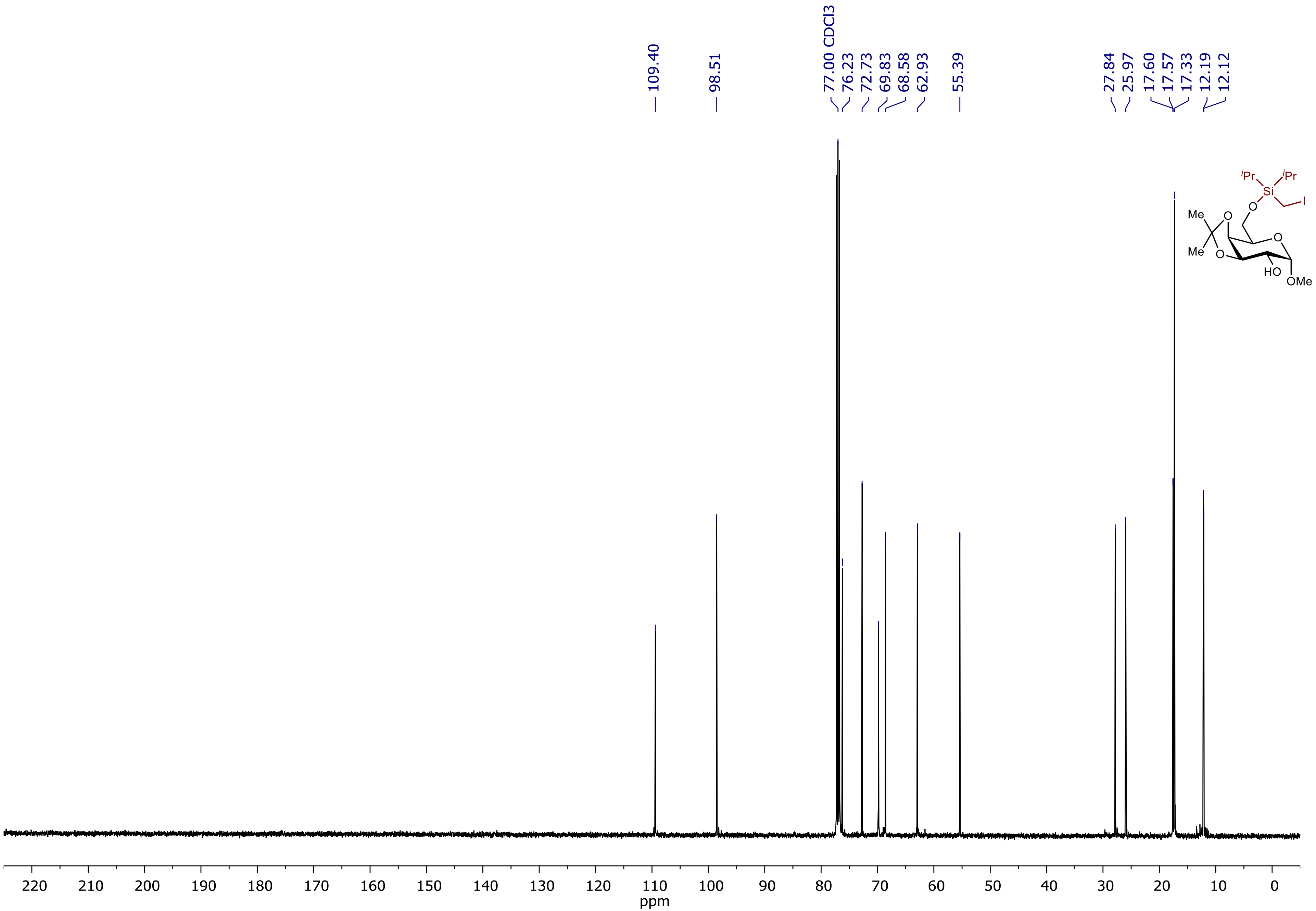

COSY of compound **2f**

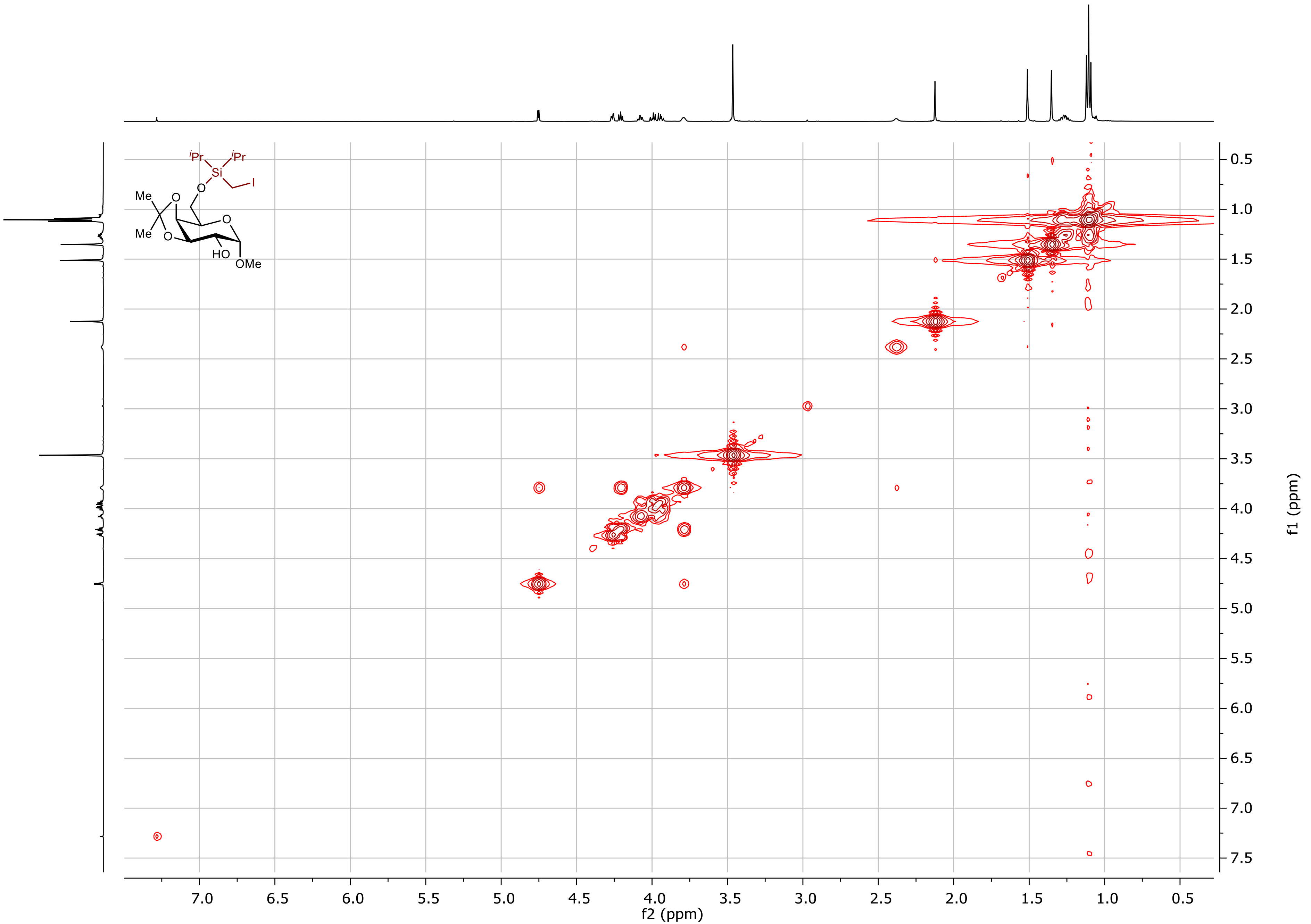

HSQC of compound **2f**

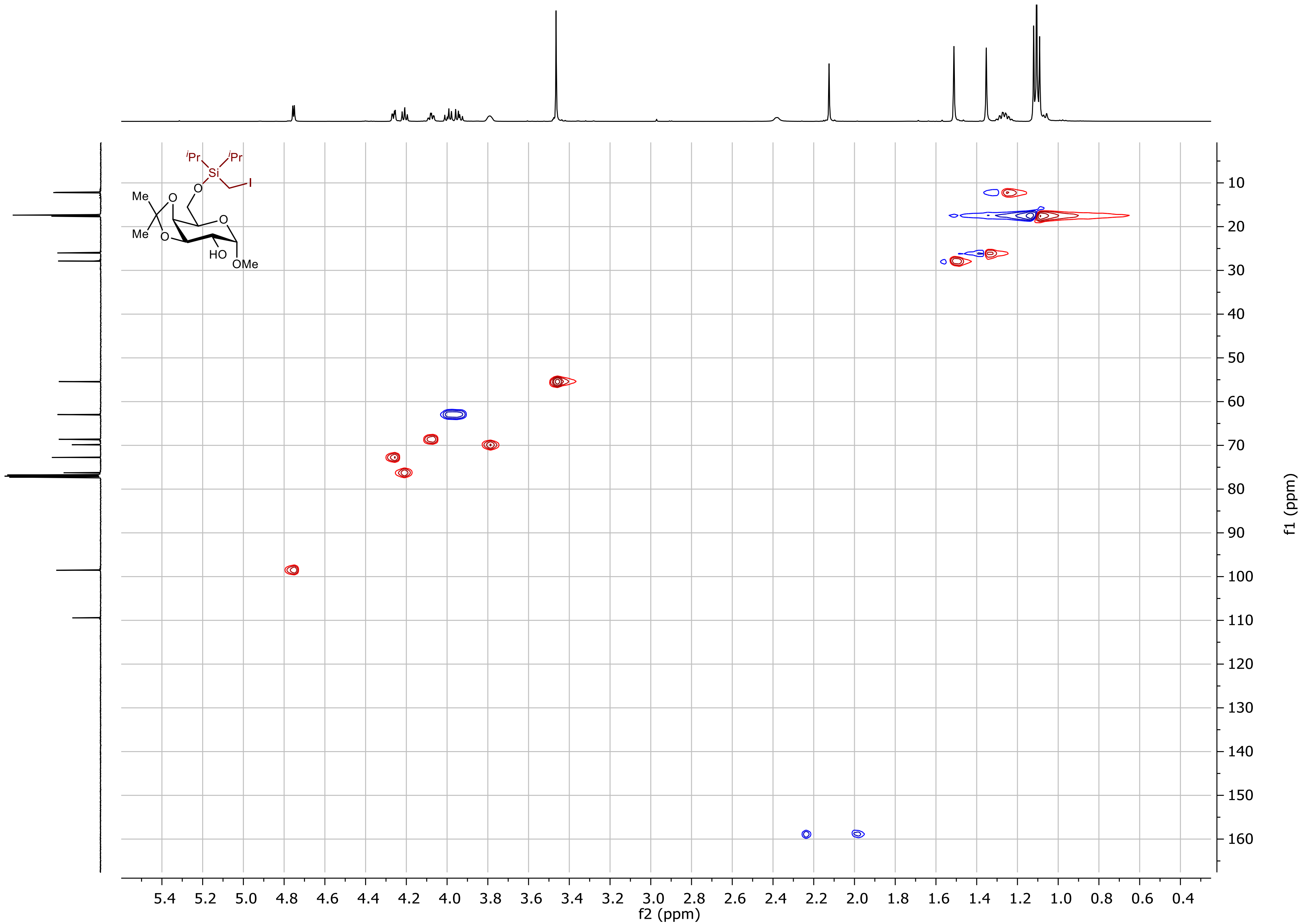

^{1}H NMR (500 MHz, $CDCl_3$) of compound **2g**

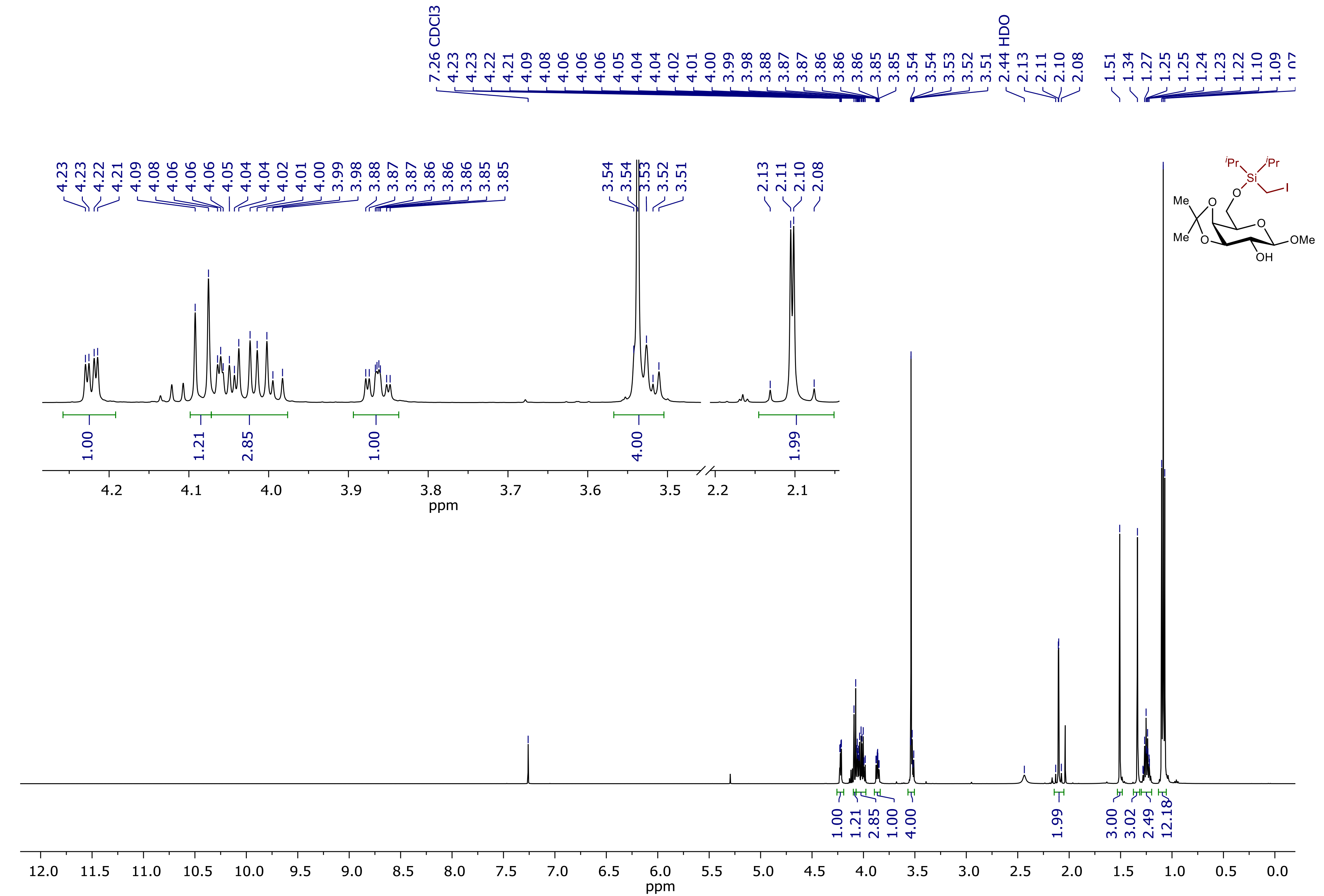

$^{13}C\{^{1}H\}$ NMR (126 MHz, $CDCl_3$) of compound **2g**

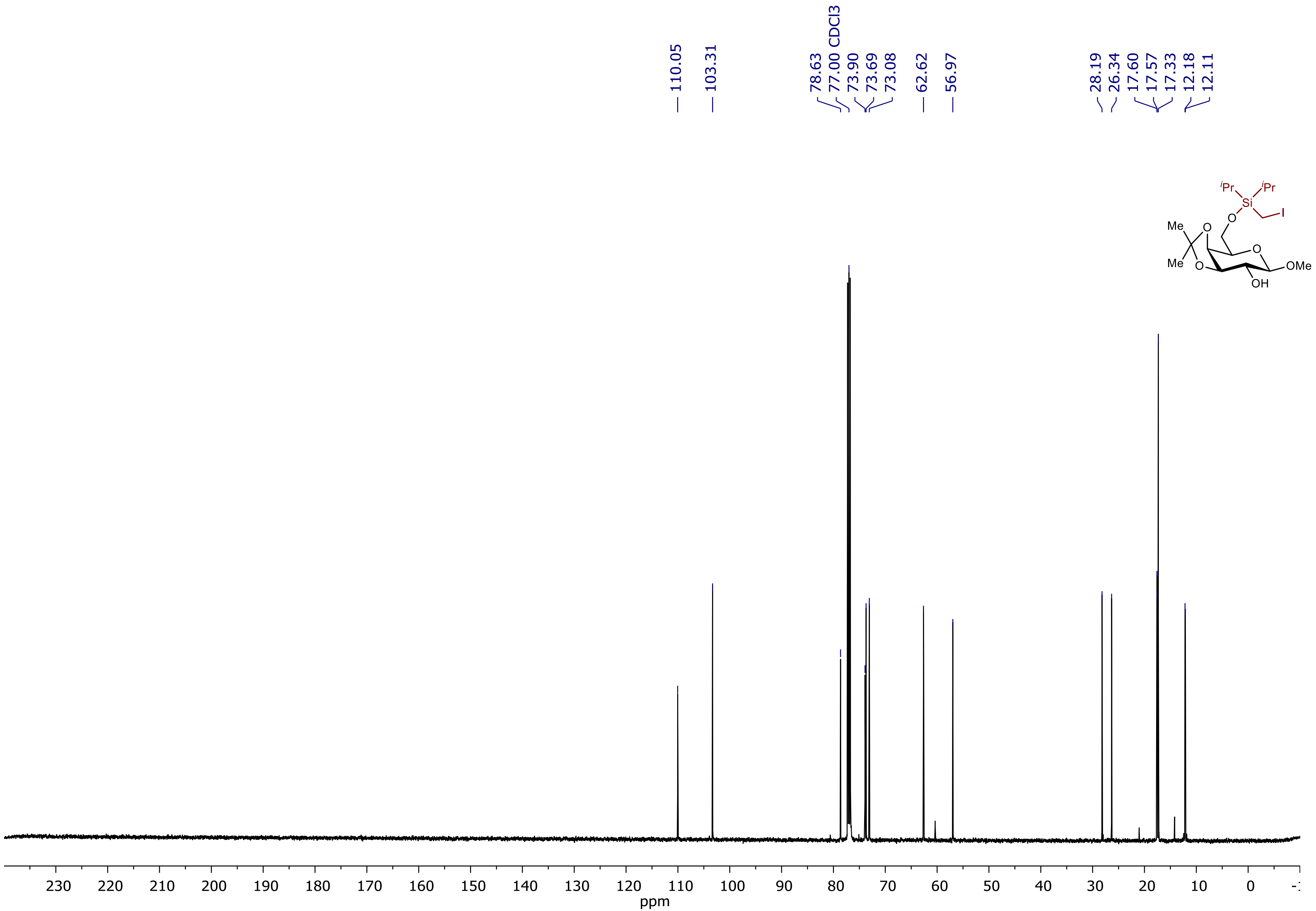

COSY of compound **2g**

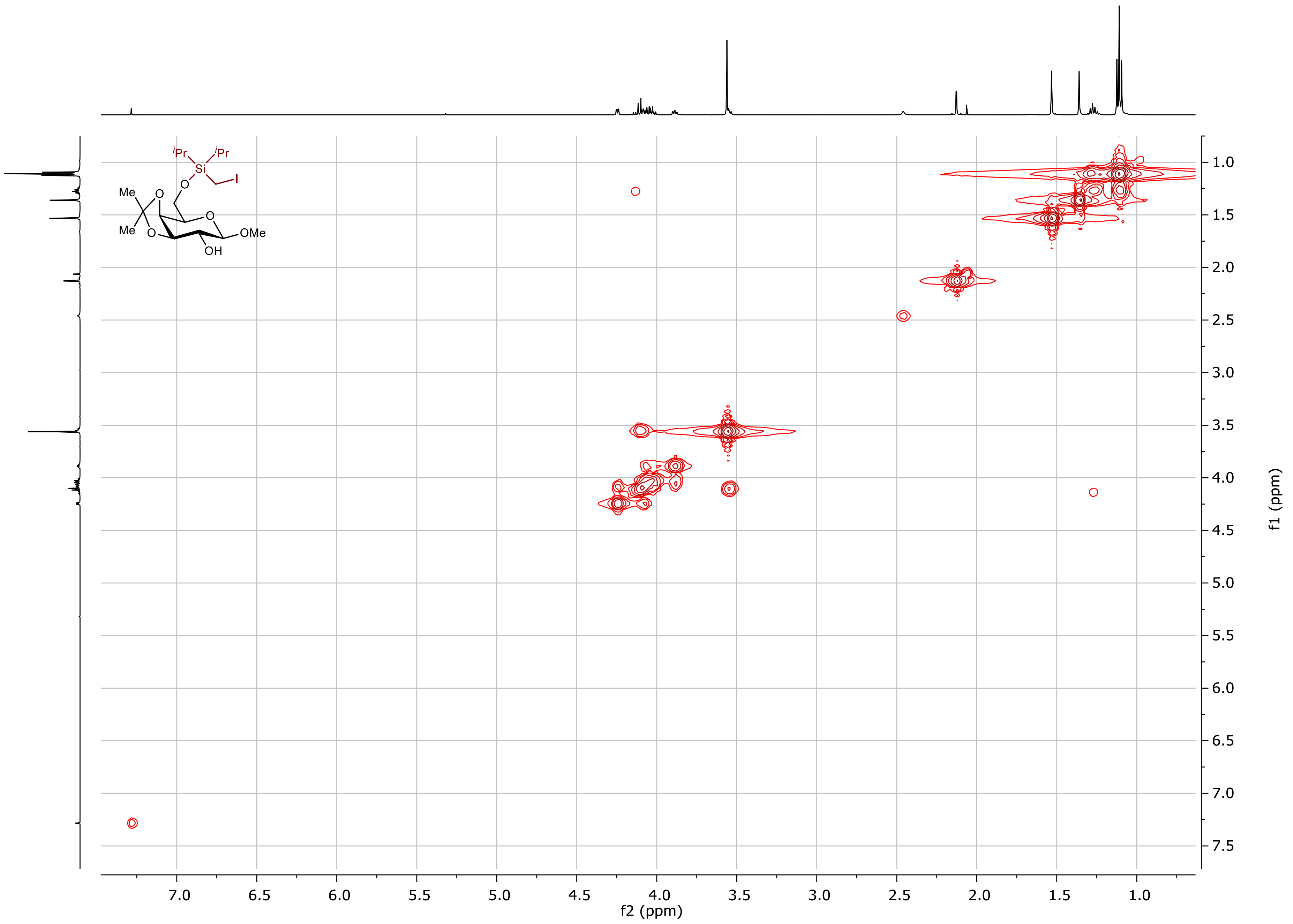

HSQC of compound **2g**

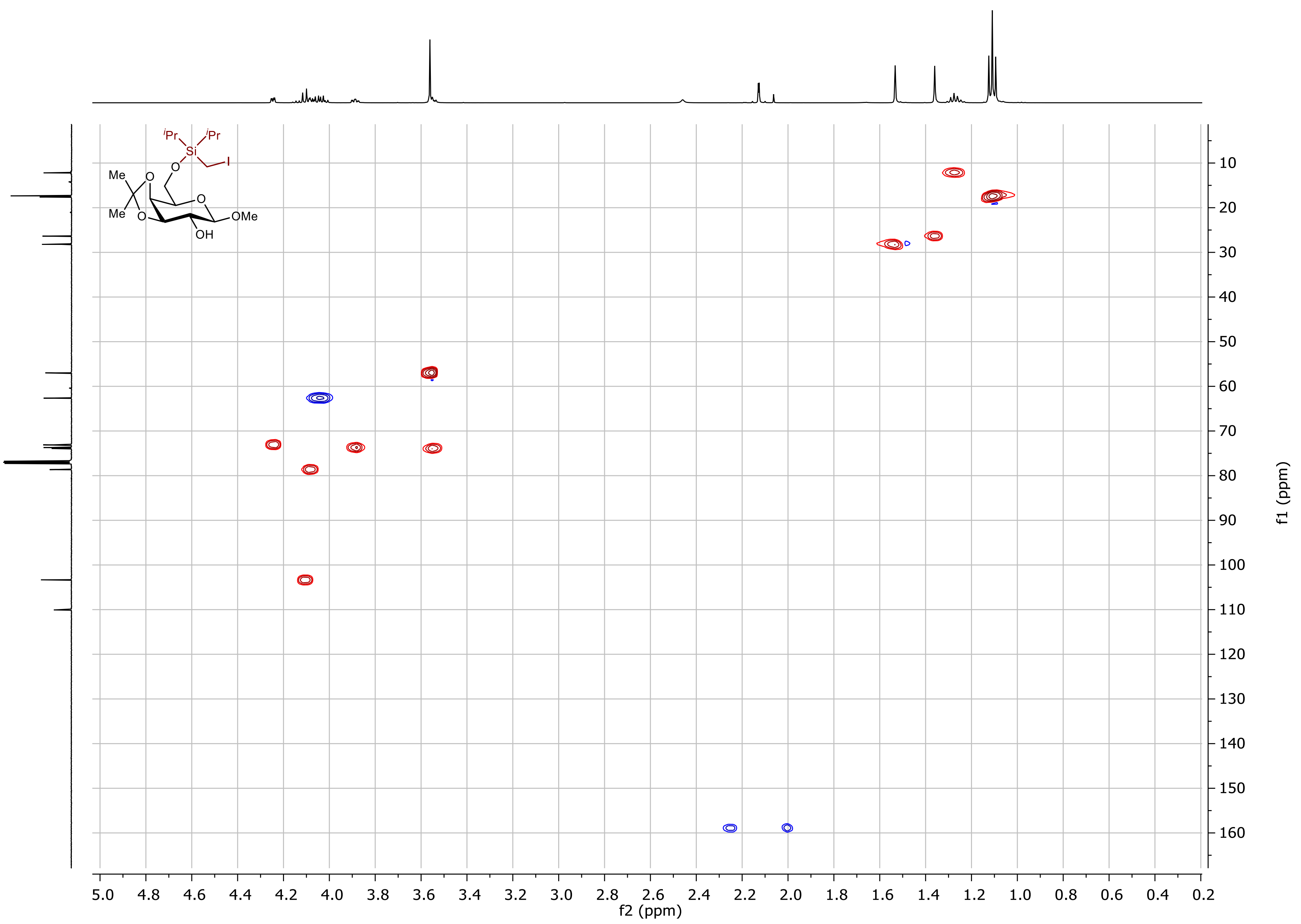

^{1}H NMR (500 MHz, $CDCl_3$) of compound **2h**

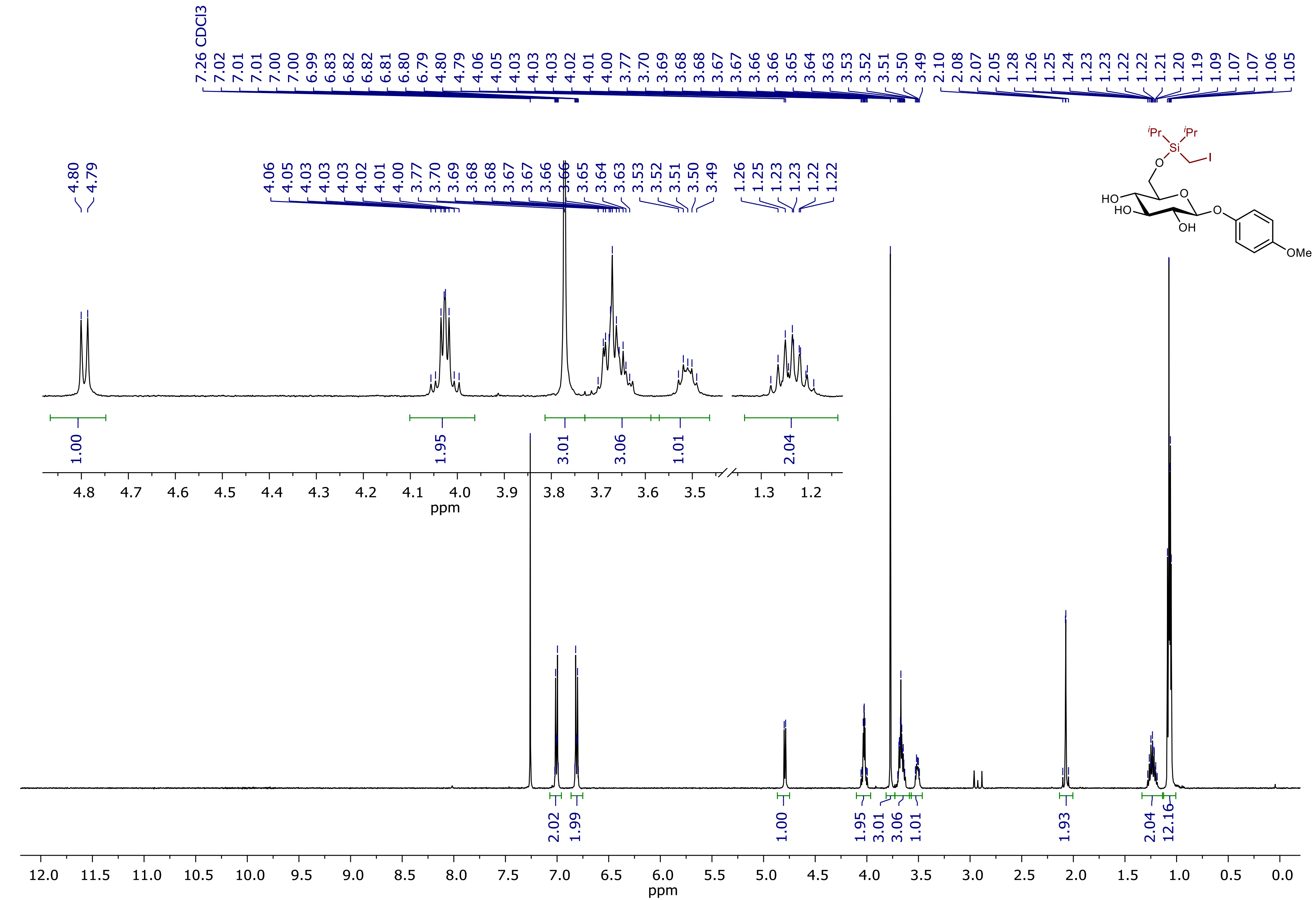

$^{13}C\{^{1}H\}$ NMR (126 MHz, $CDCl_3$) of compound **2h**

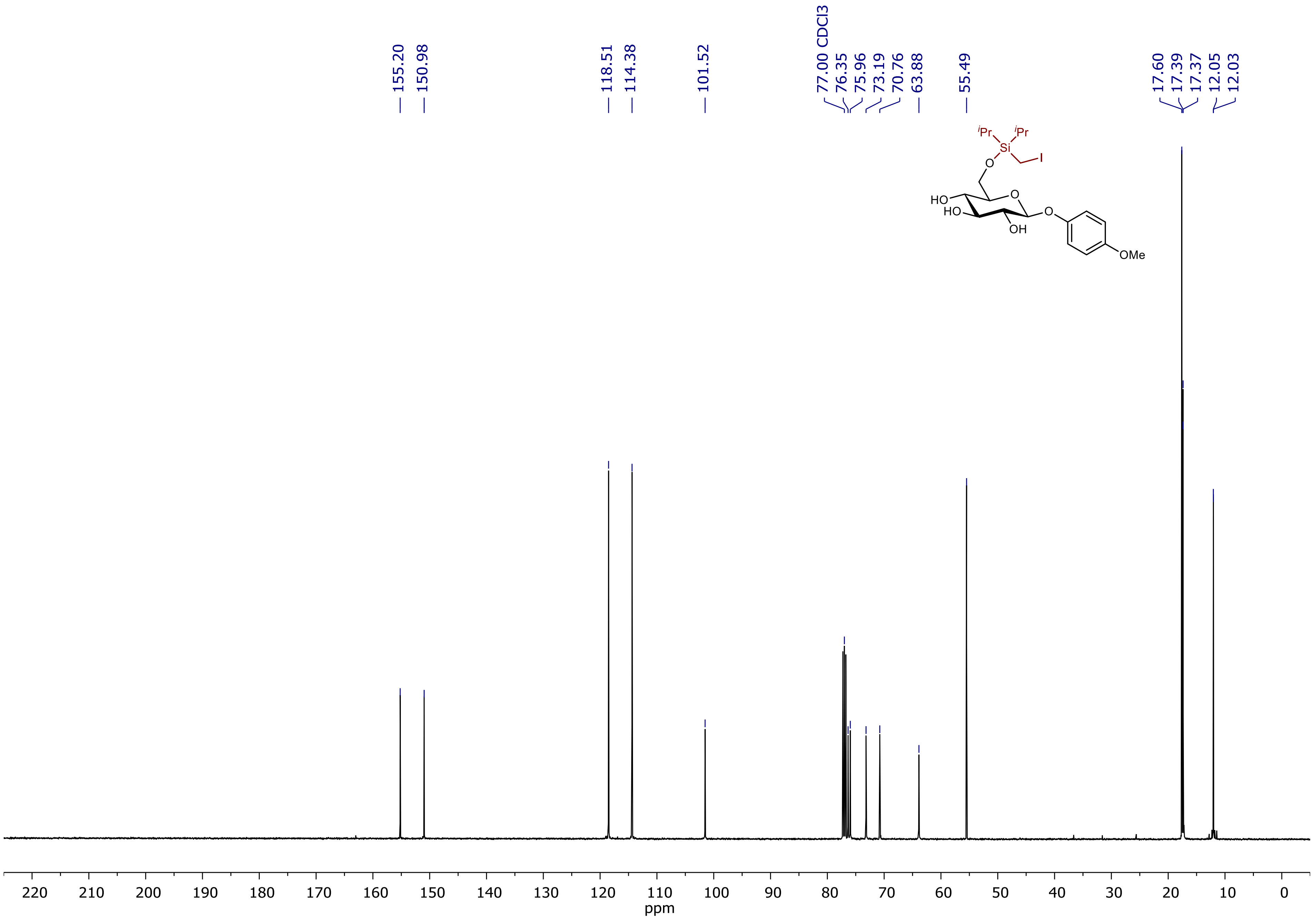

COSY of compound **2h**

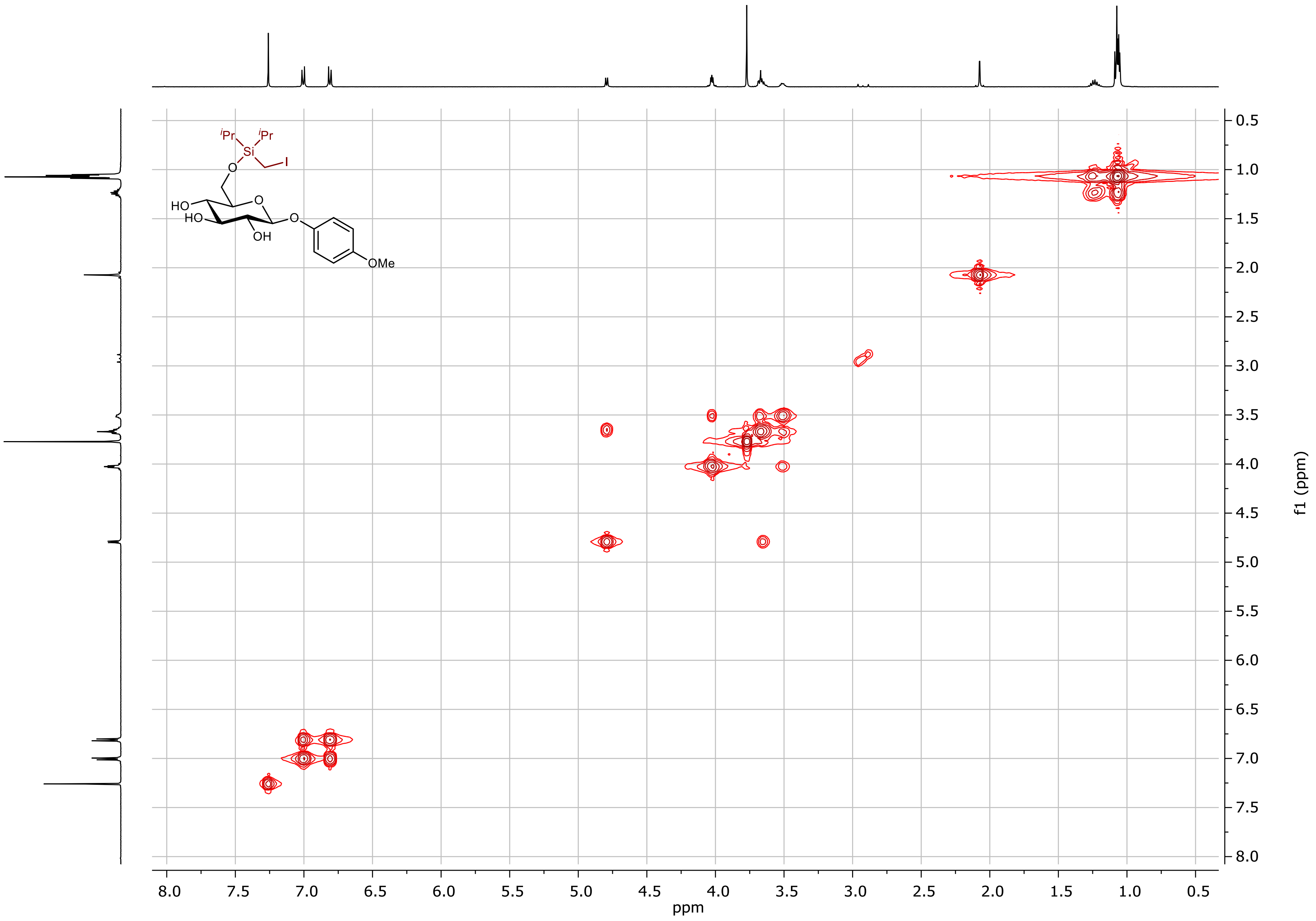

HSQC of compound **2h**

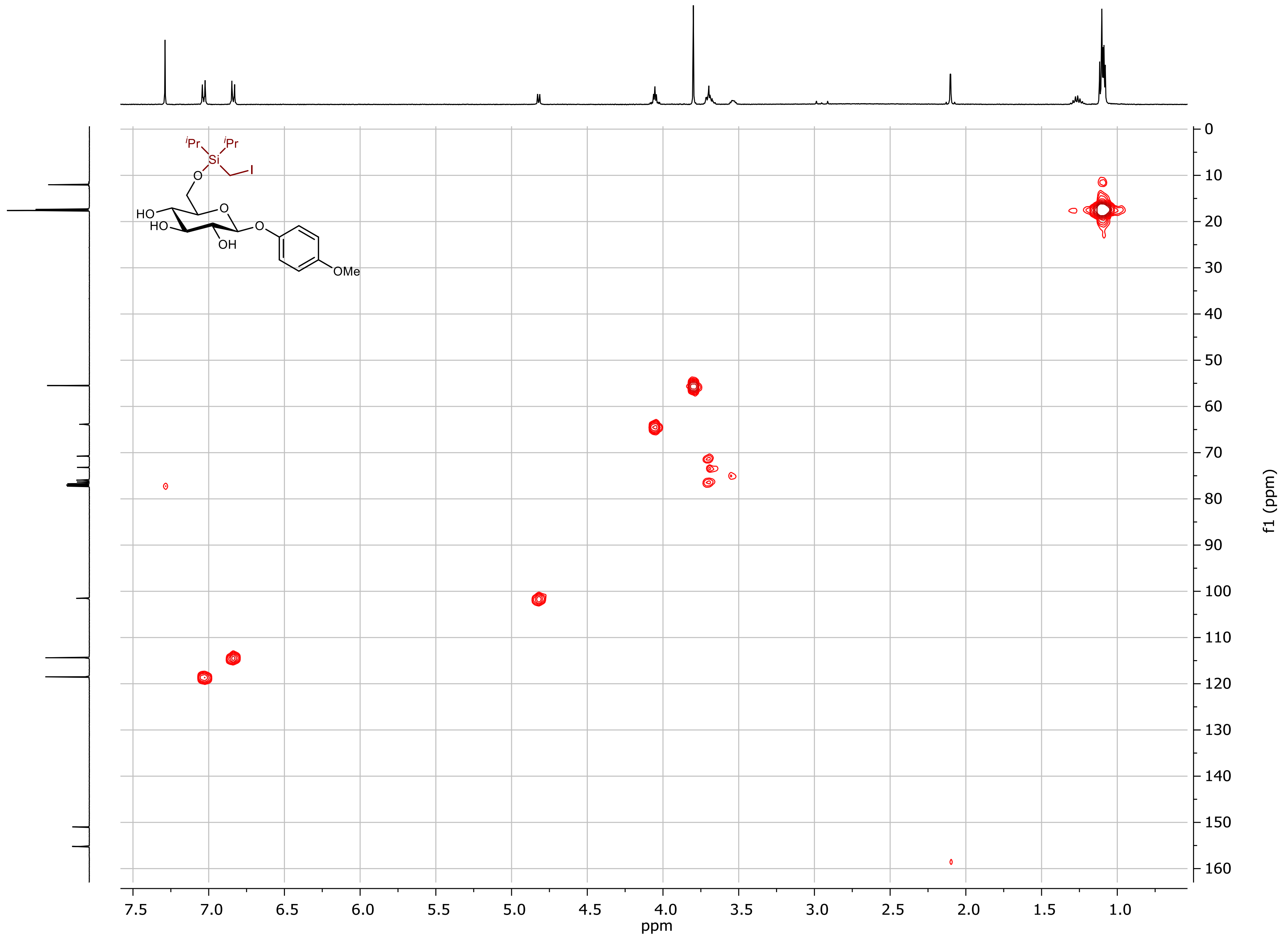

^{1}H NMR (500 MHz, $CDCl_3$) of compound **2i**

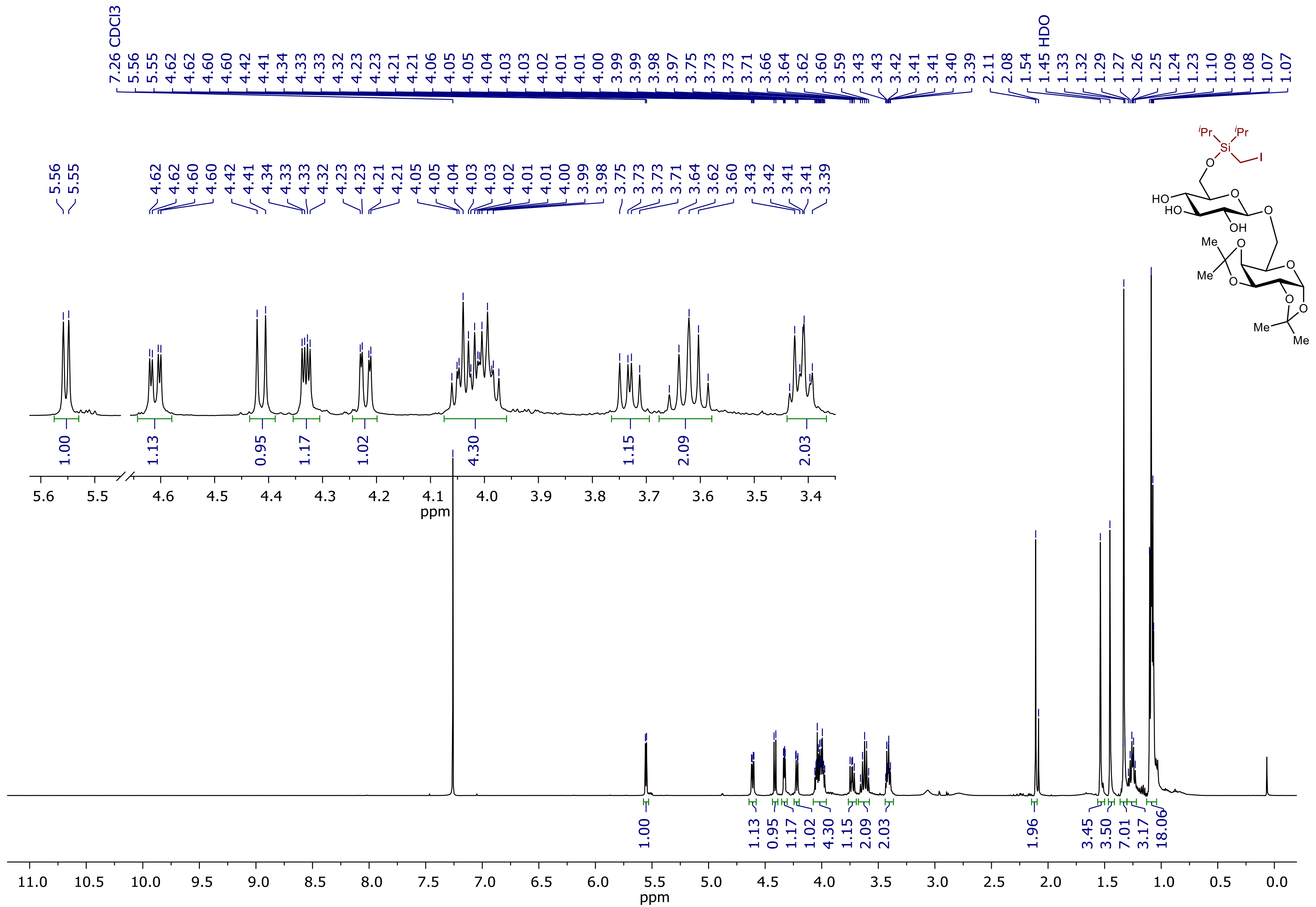

$^{13}C\{^{1}H\}$ NMR (126 MHz, $CDCl_3$) of compound **2i**

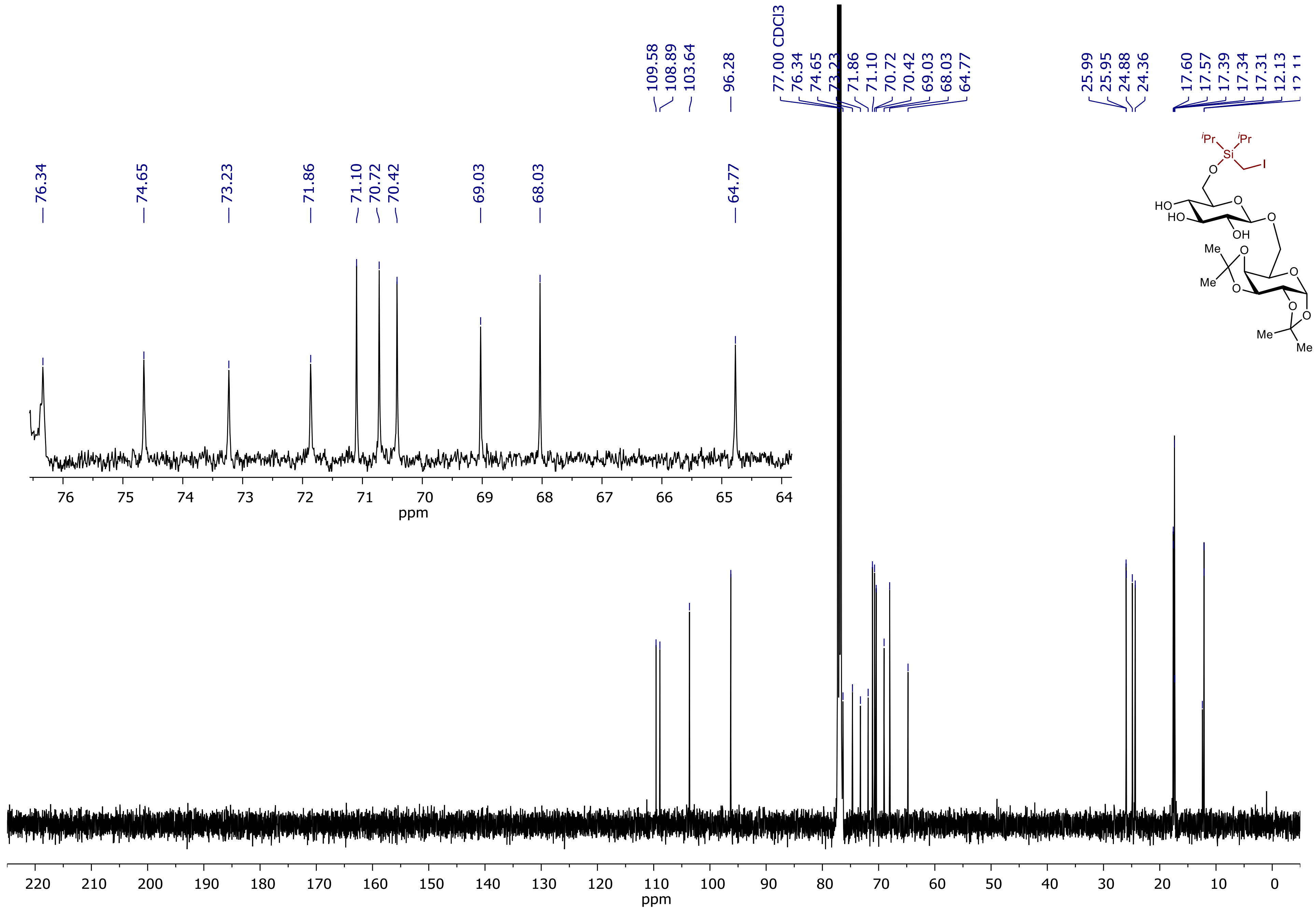

COSY of compound **2i**

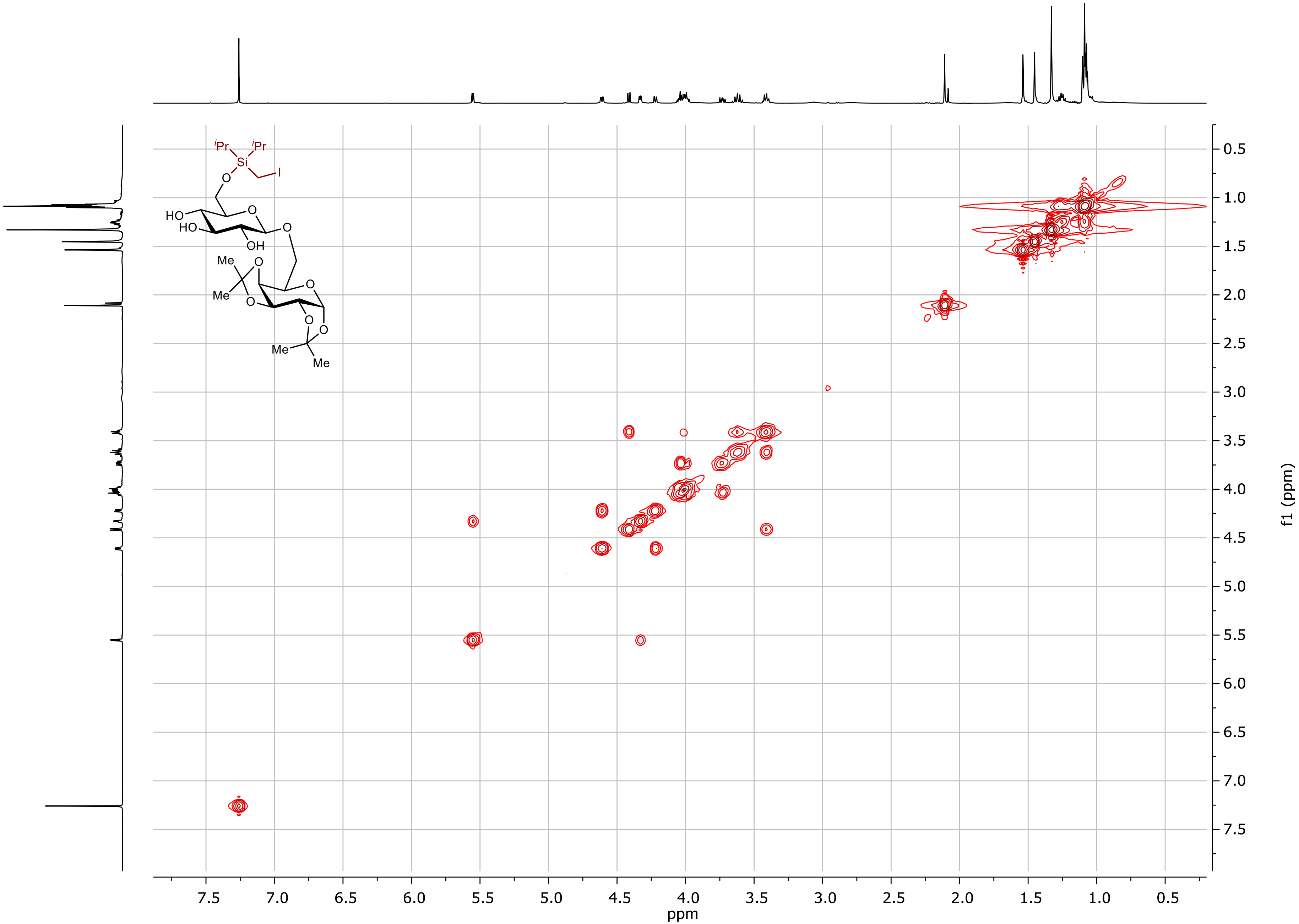

HSQC of compound **2i**

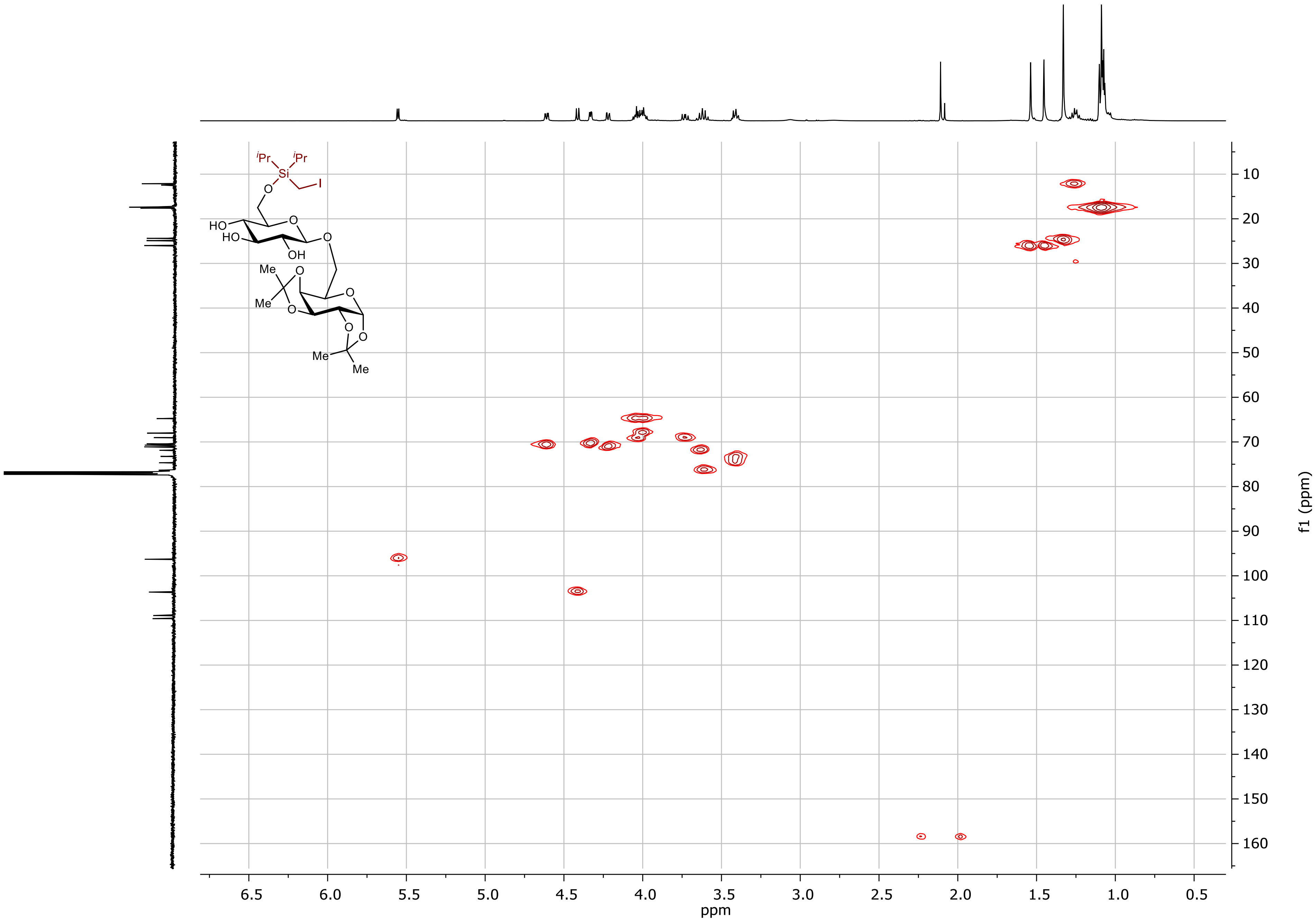

^{1}H NMR (500 MHz, MeOD) of compound **2j**

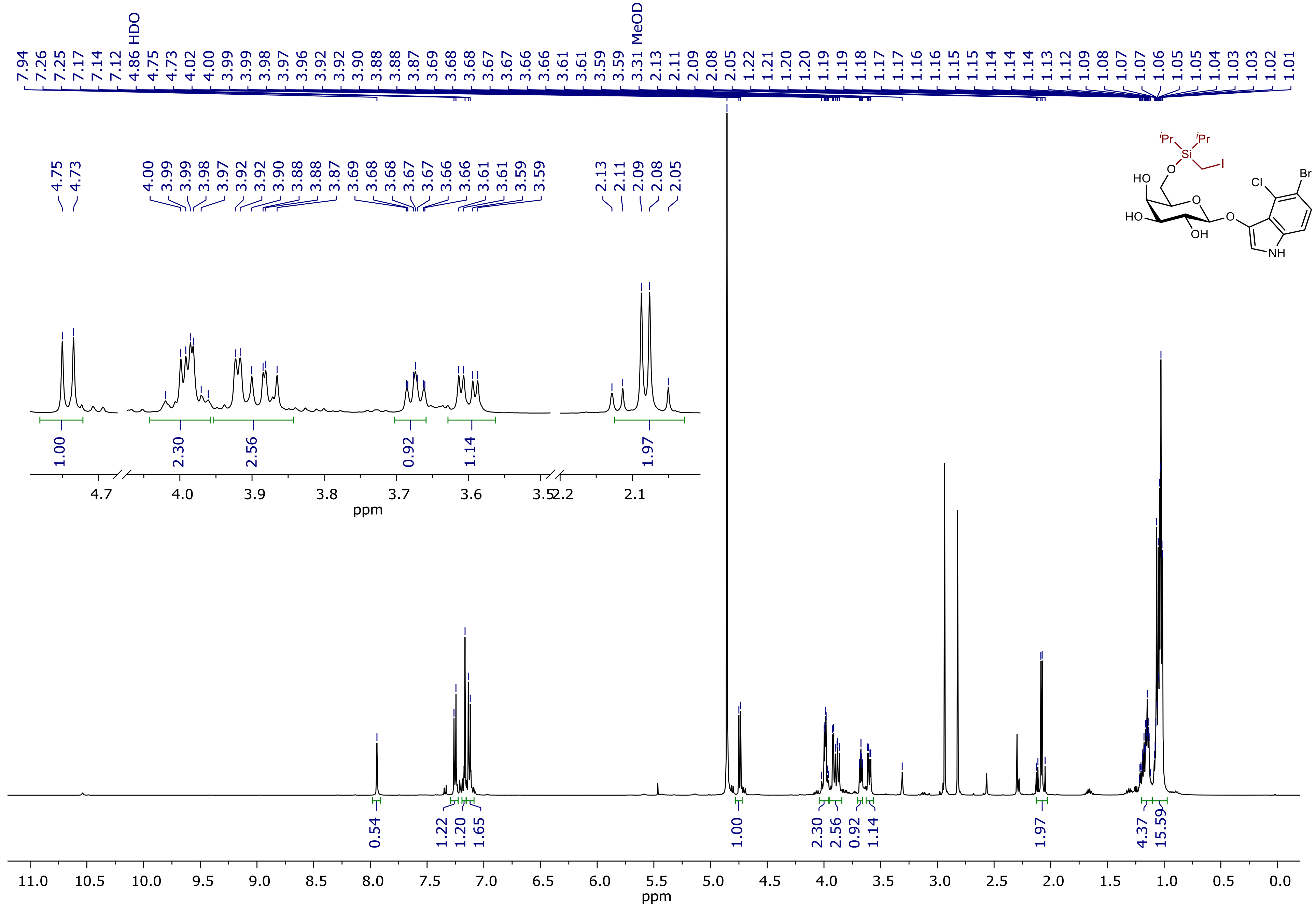

$^{13}C\{^1H\}$ NMR (126 MHz, MeOD) of compound **2j**

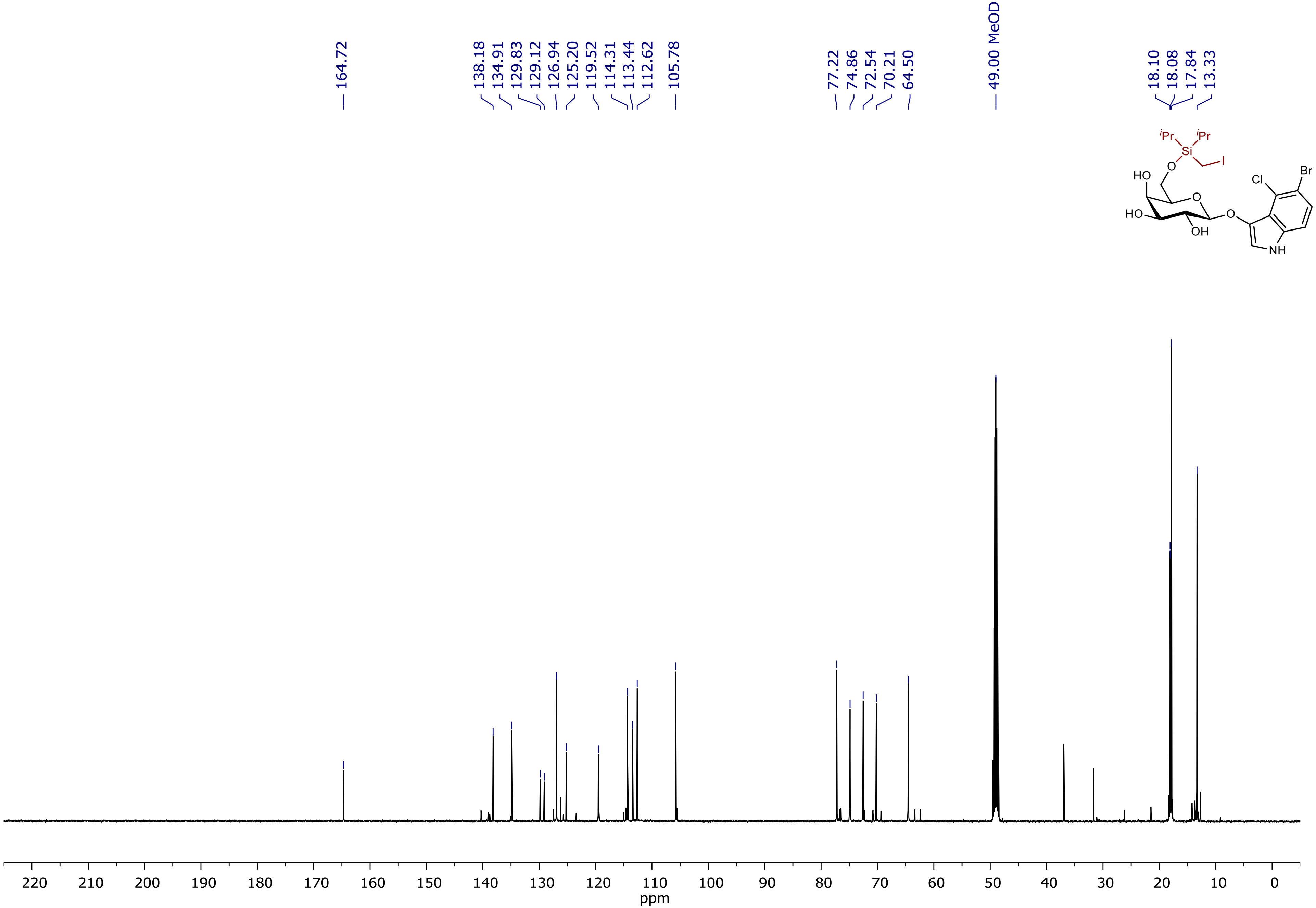

COSY of compound **2j**

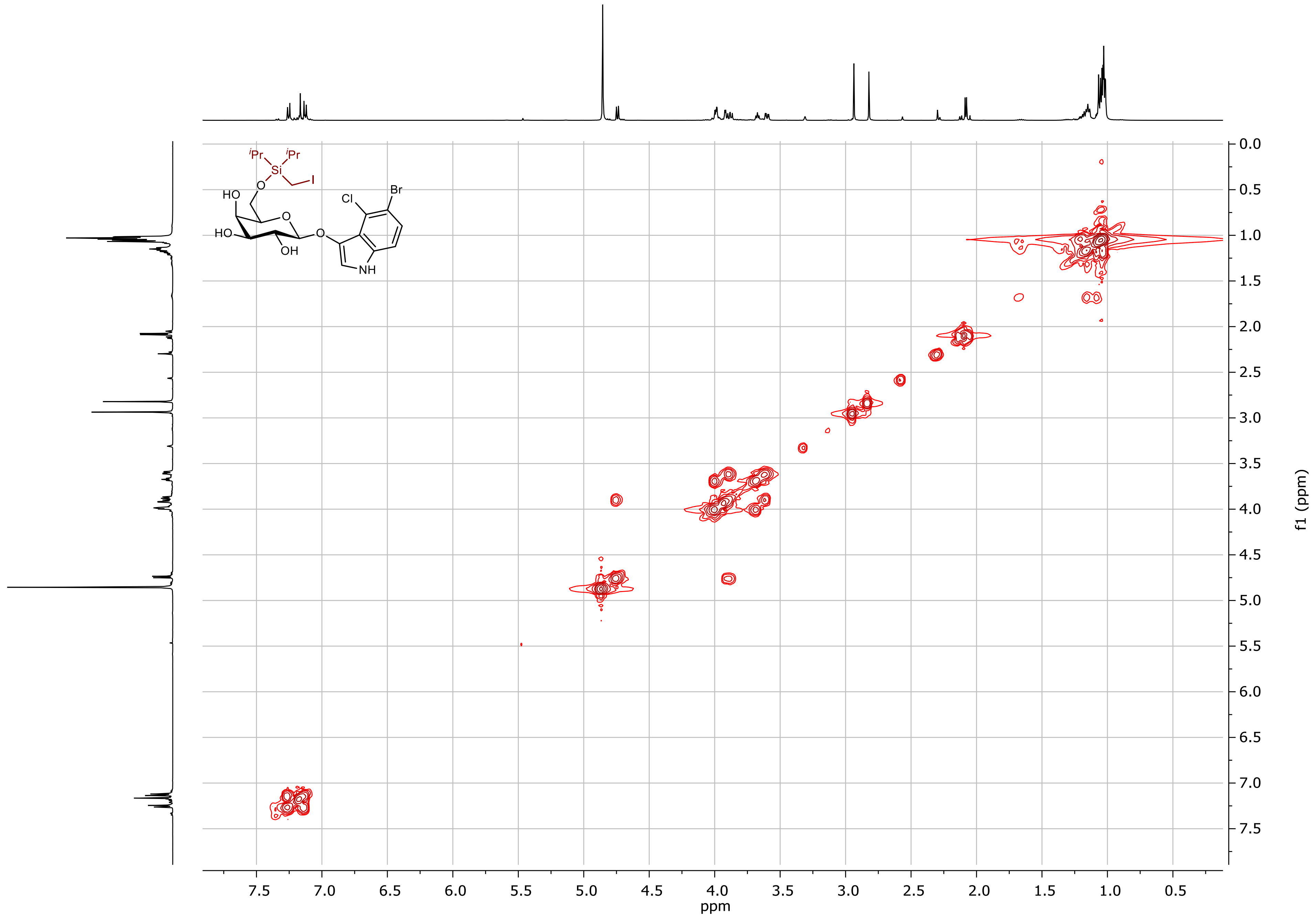

HSQC of compound **2j**

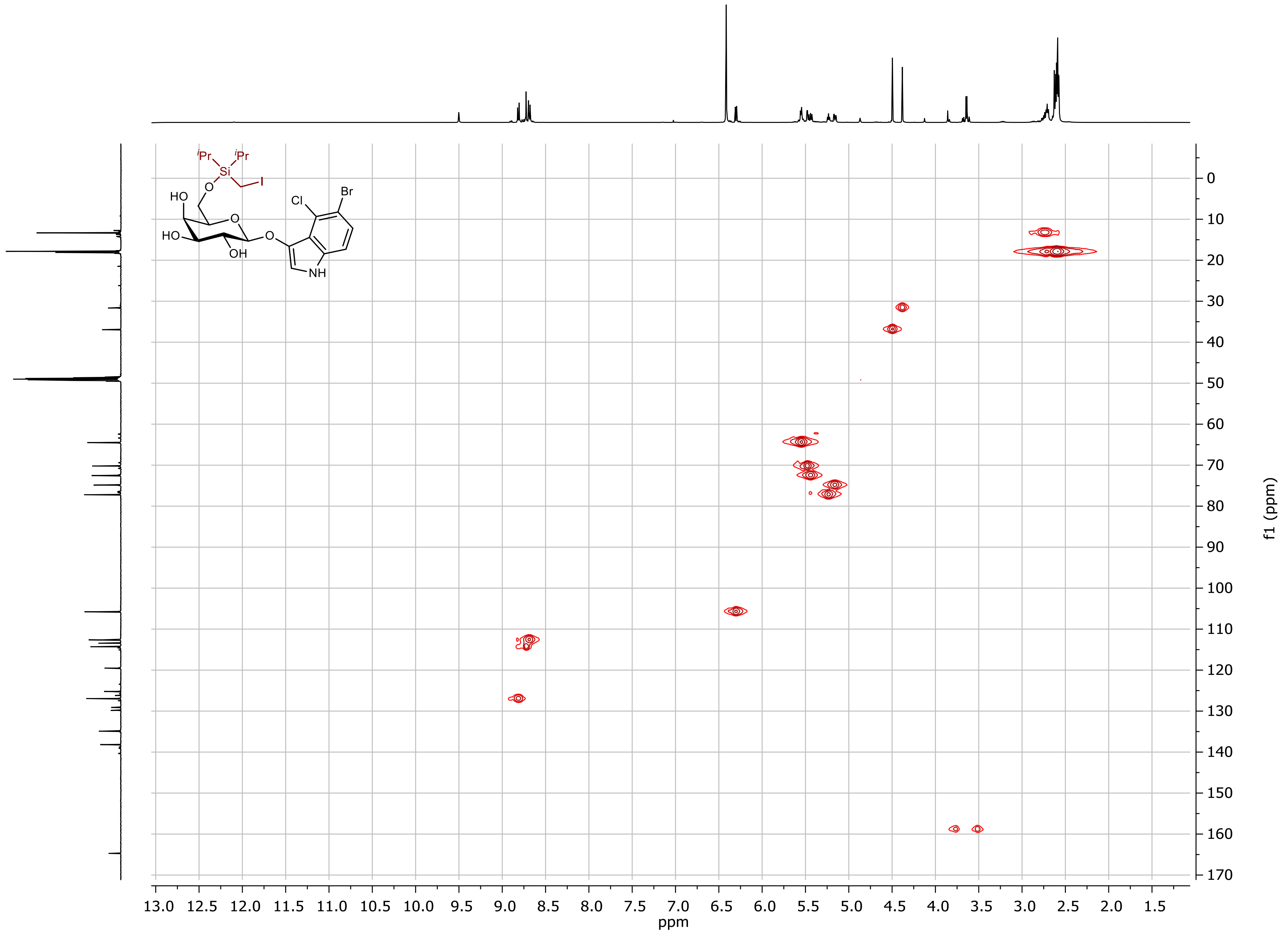

^{1}H NMR (500 MHz, MeOD) of compound **2k**

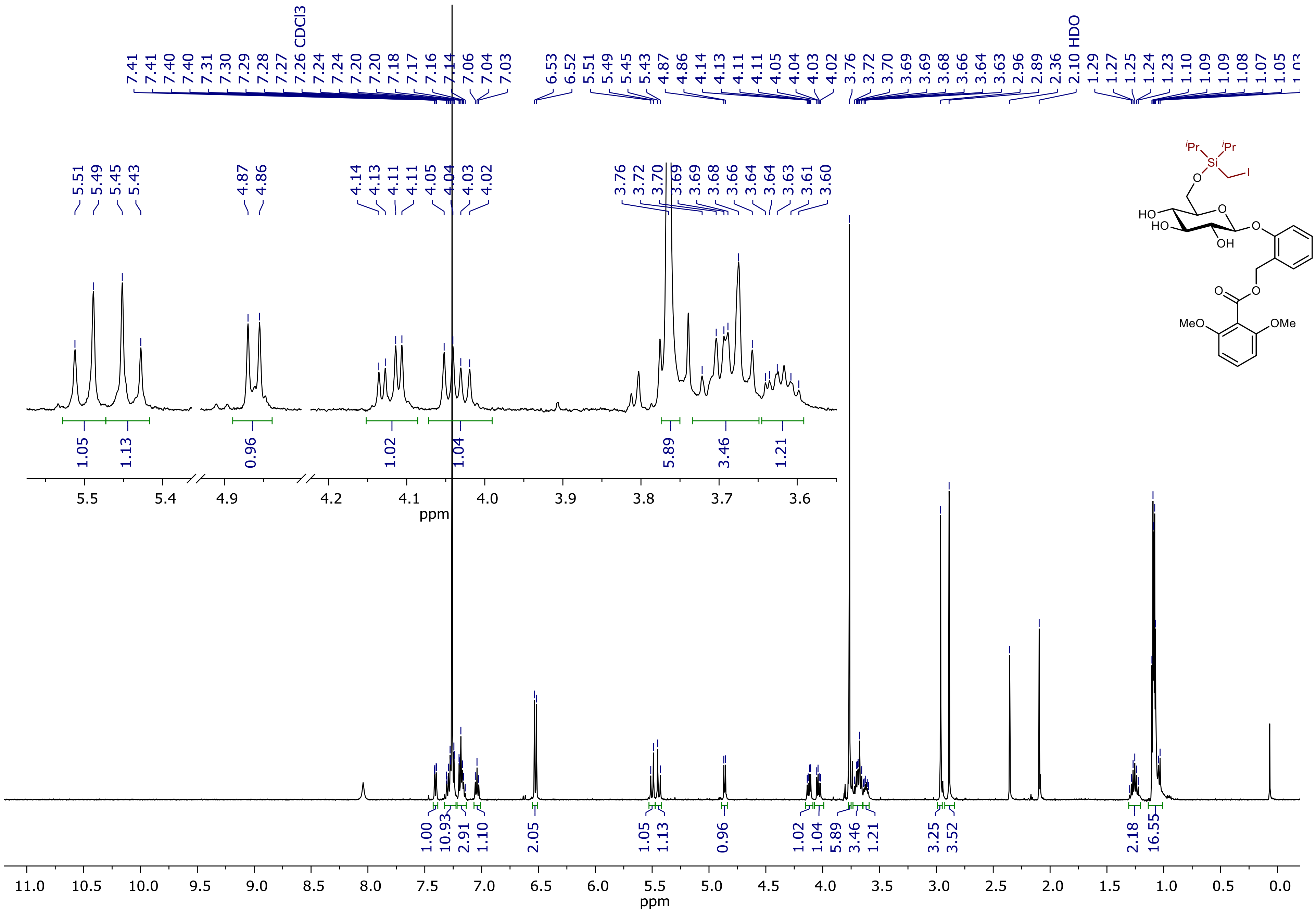

^{1}H NMR (500 MHz, $CDCl_3$) of compound **2l**

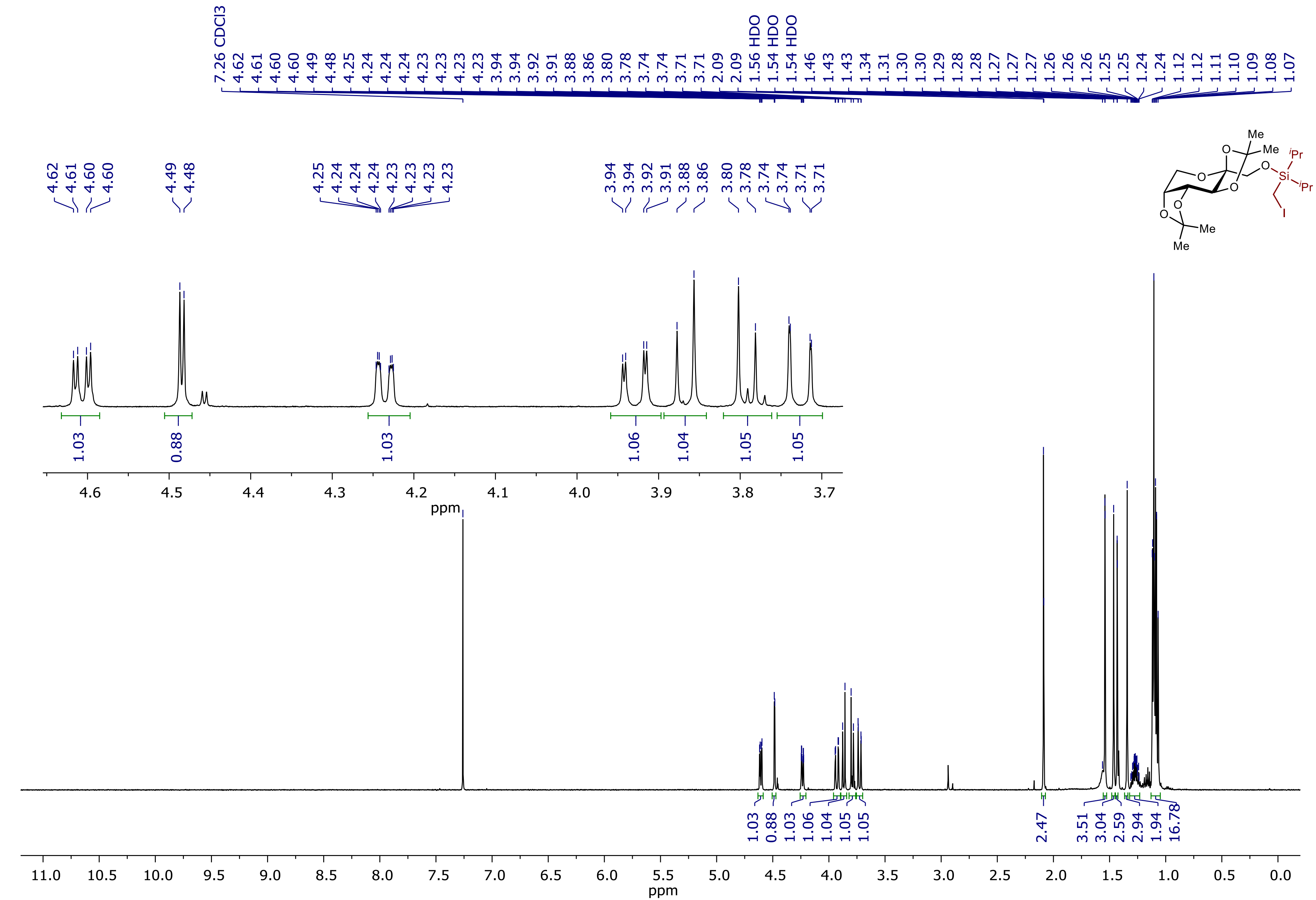

$^{13}C\{^1H\}$ **NMR** (126 MHz, $CDCl_3$) of compound **2l**

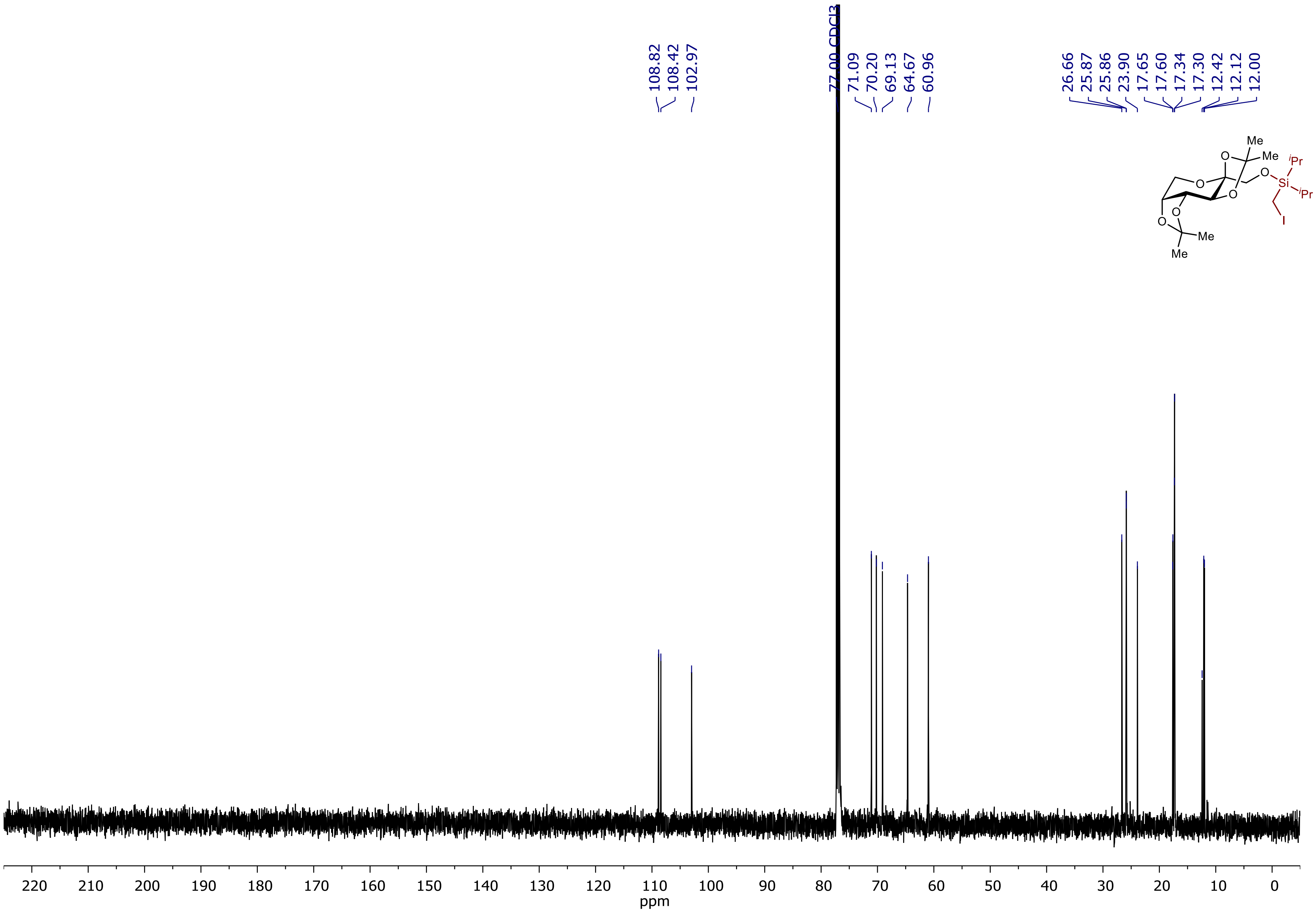

COSY of compound **2l**

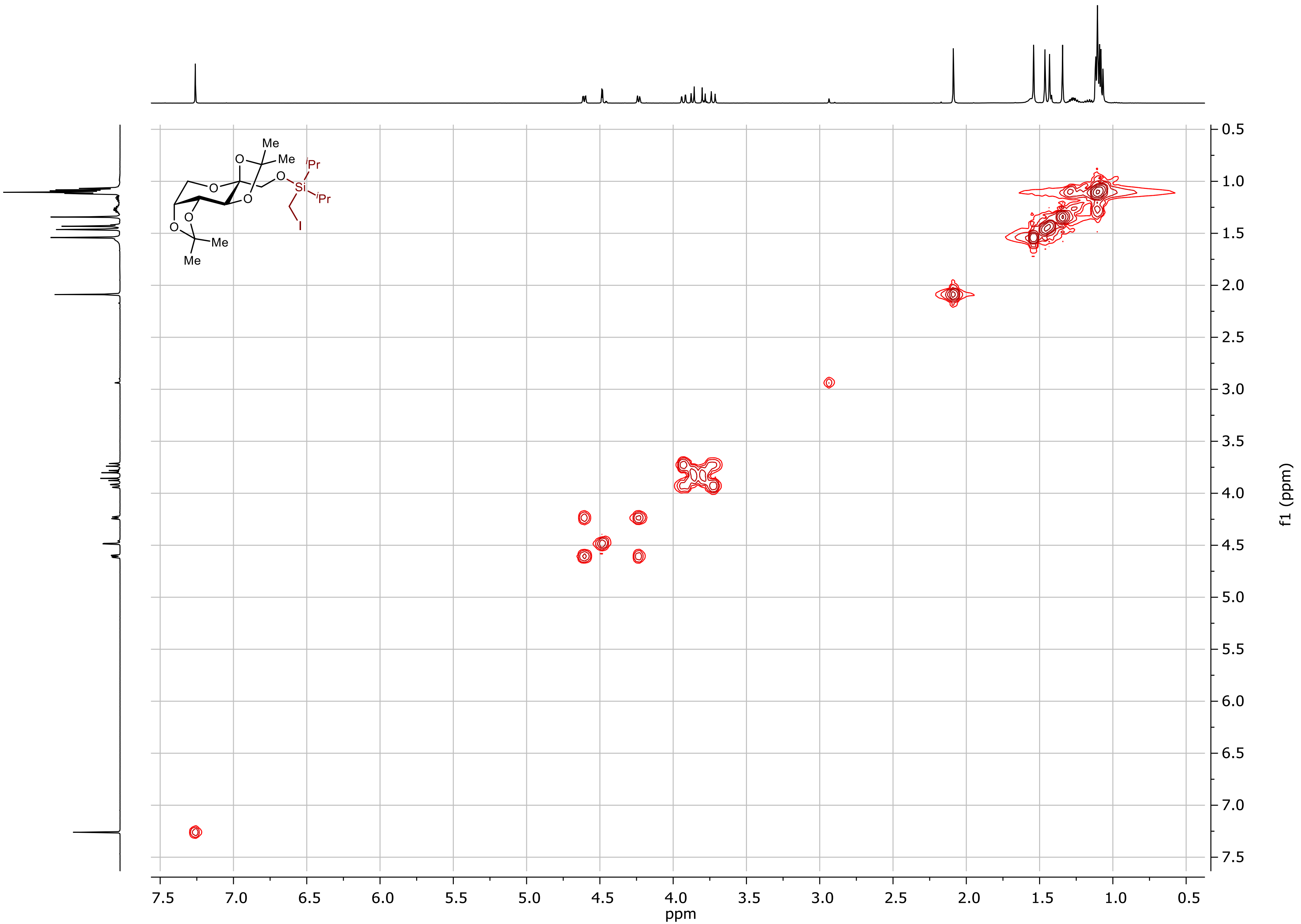

HSQC of compound **2l**

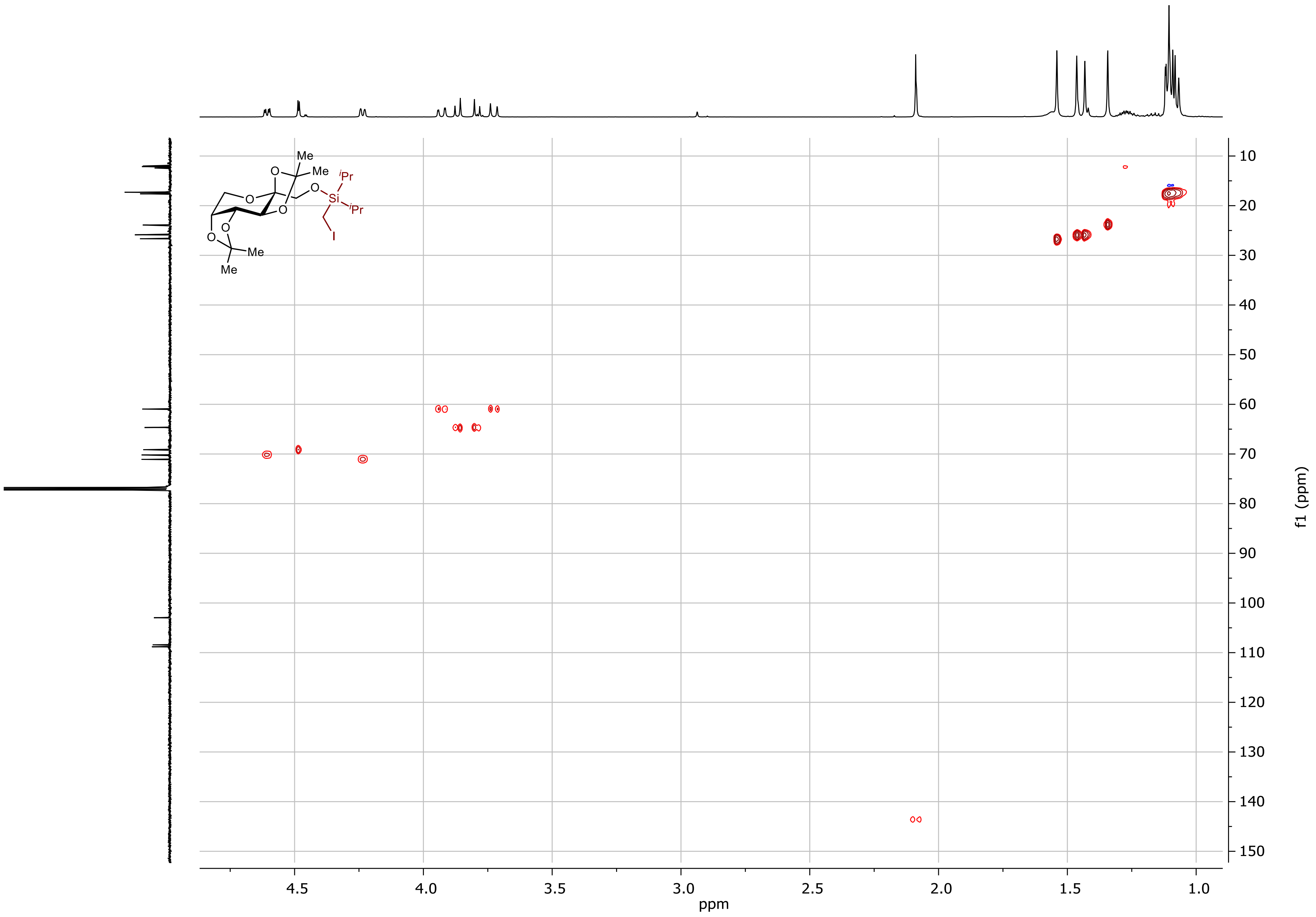

^{1}H NMR (500 MHz, $CDCl_3$) of compound **2m**

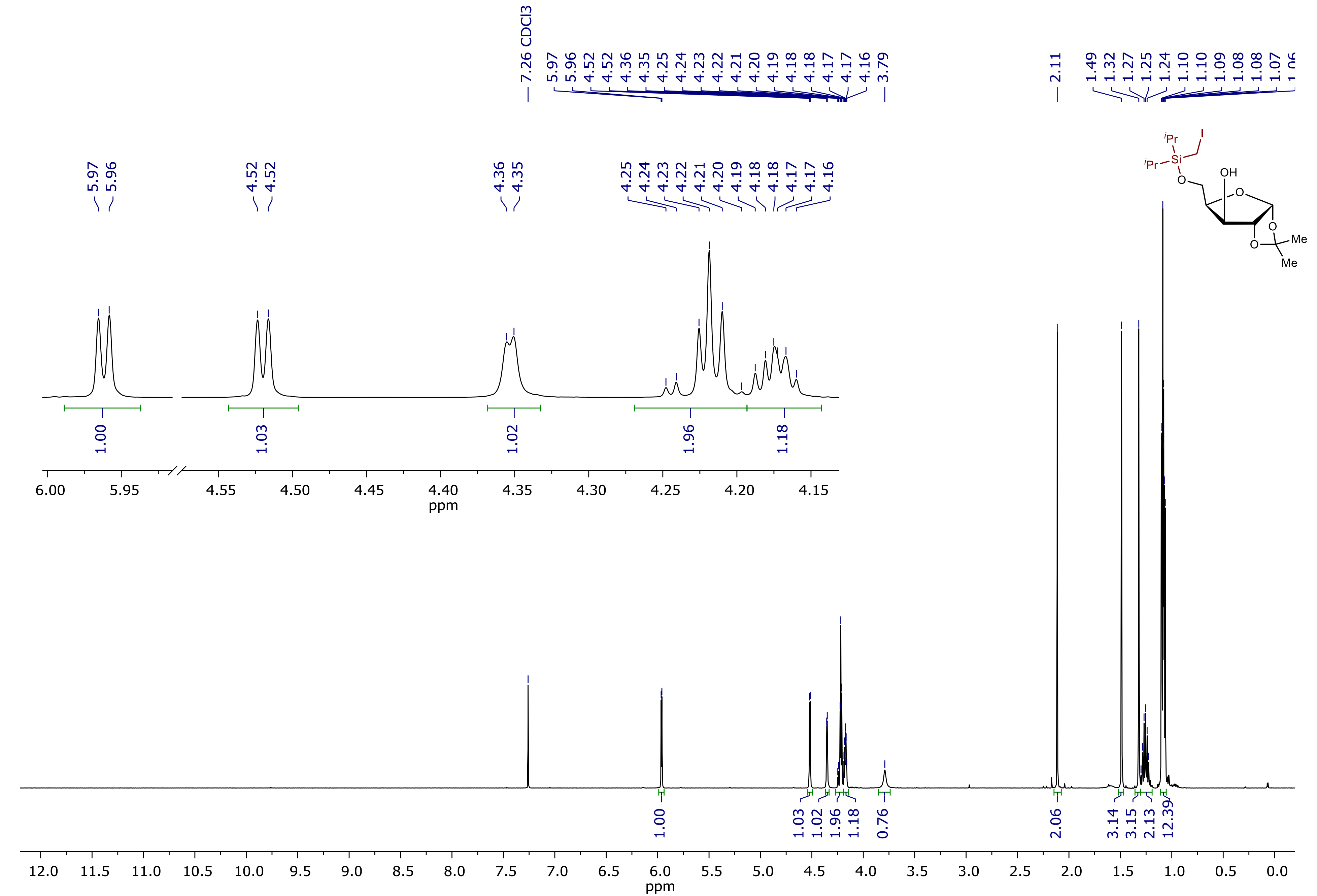

$^{13}C\{^1H\}$ **NMR** (126 MHz, $CDCl_3$) of compound **2m**

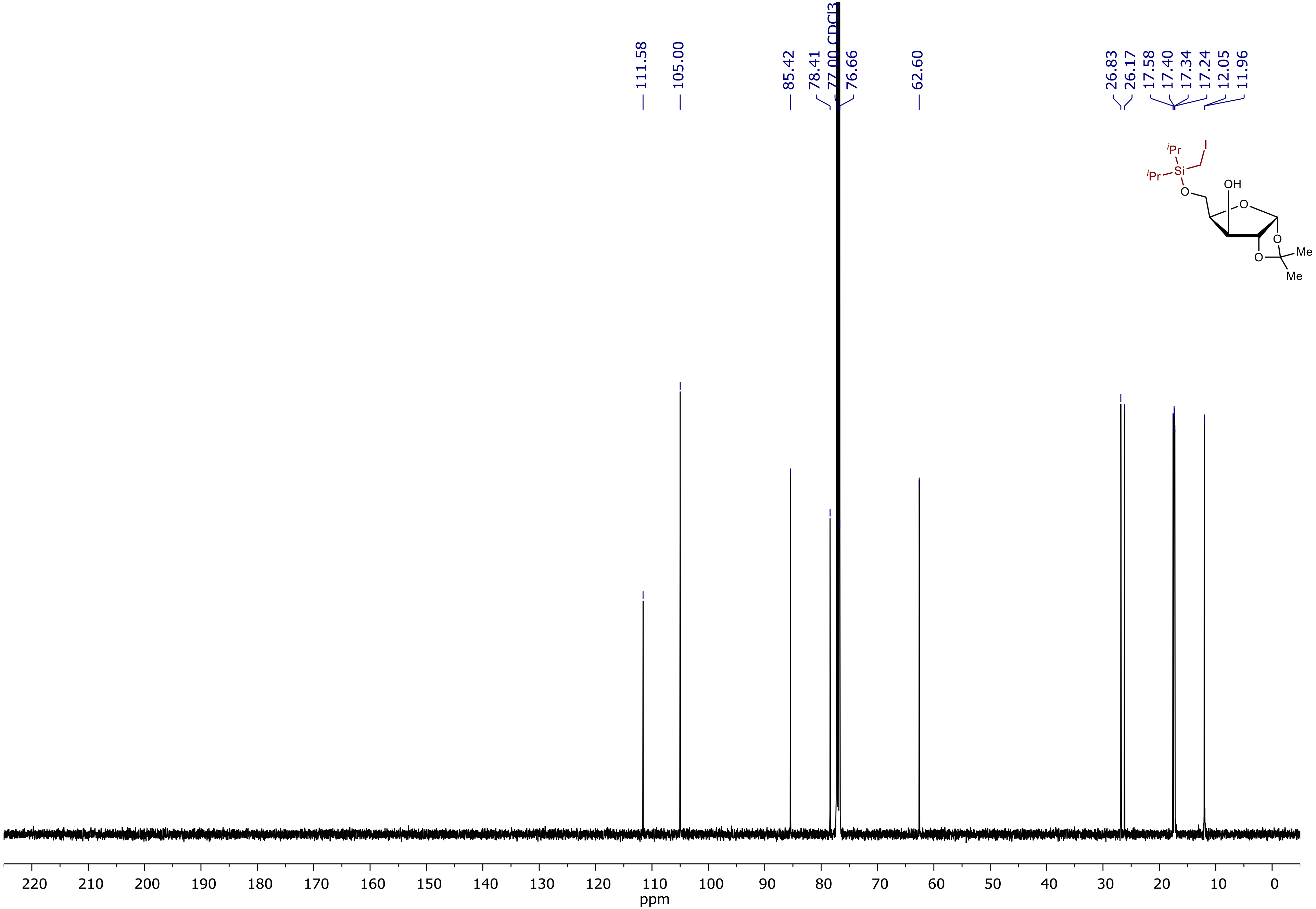

COSY of compound **2m**

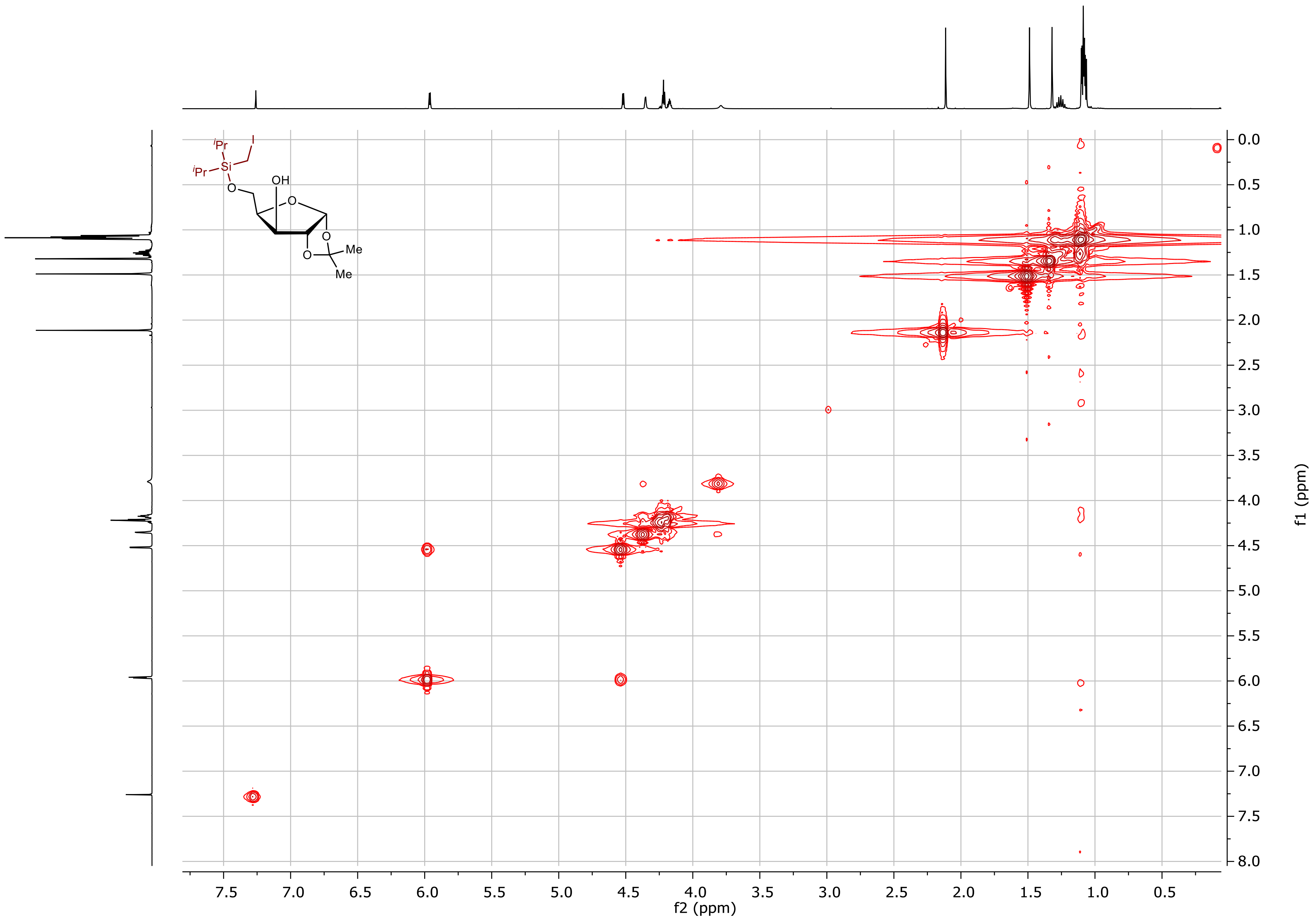

HSQC of compound **2m**

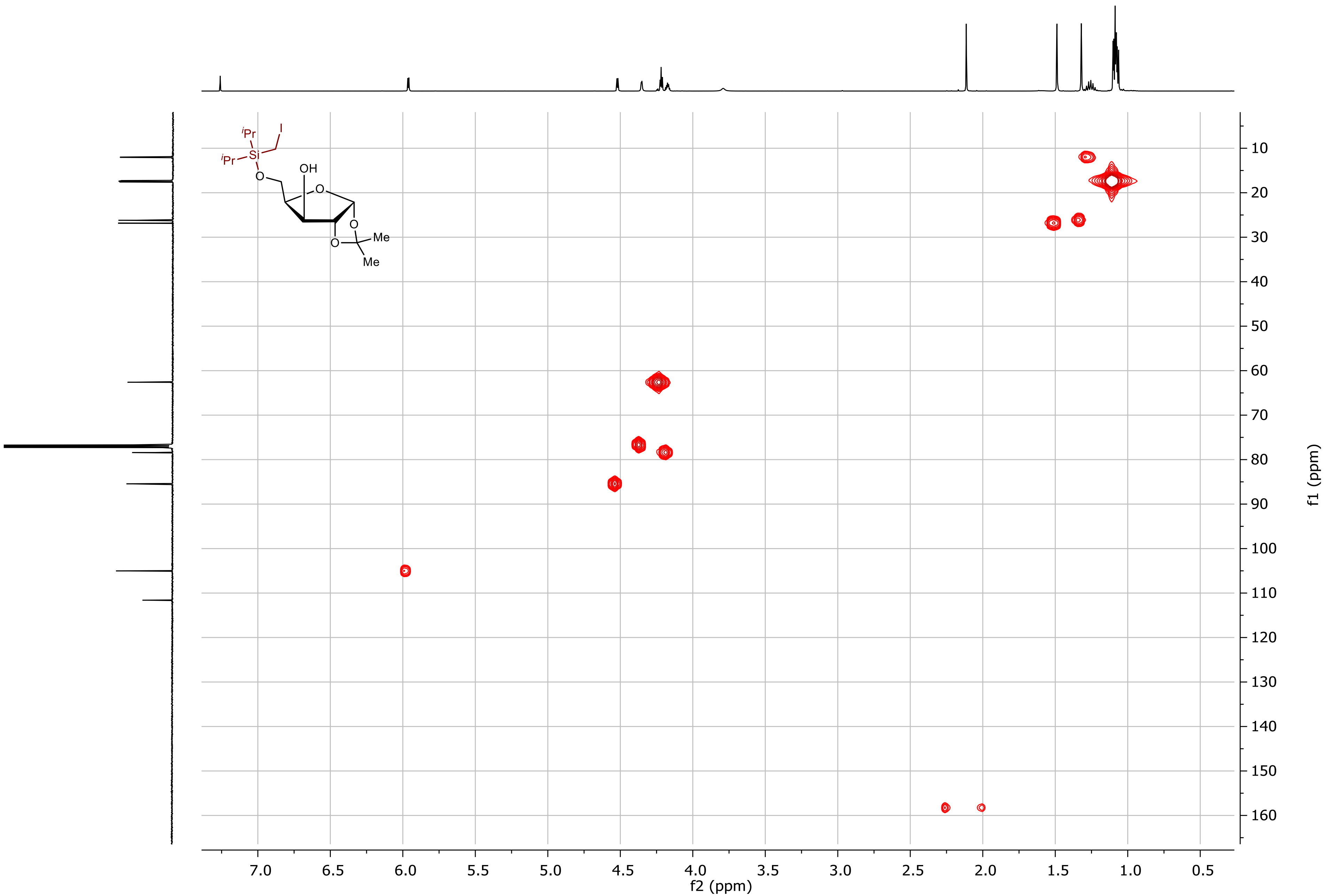

^{1}**H NMR** (500 MHz, $CDCl_3$) of compound **SI-2**

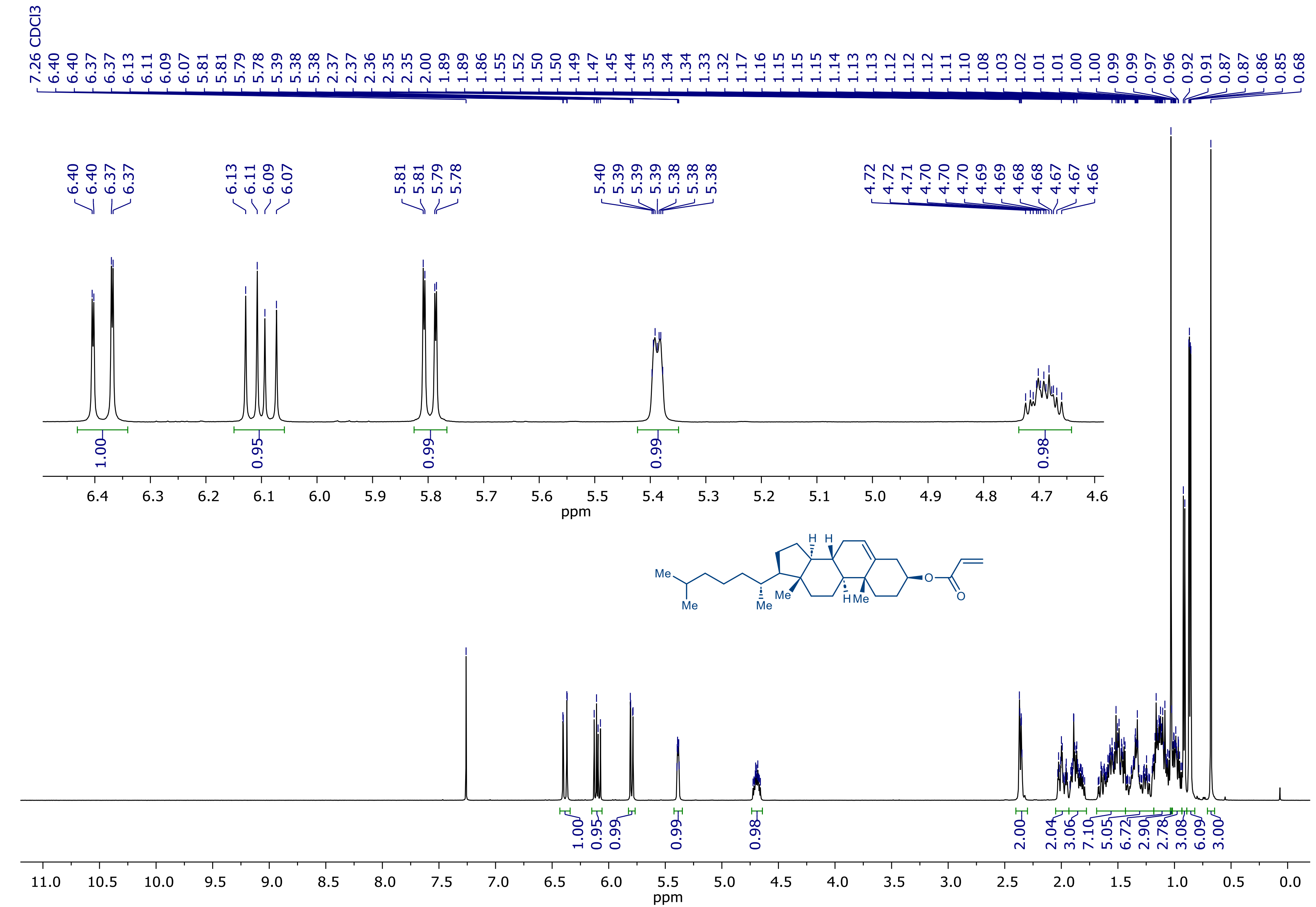

$^{13}C\{^1H\}$ **NMR** (126 MHz, $CDCl_3$) of compound **SI-2**

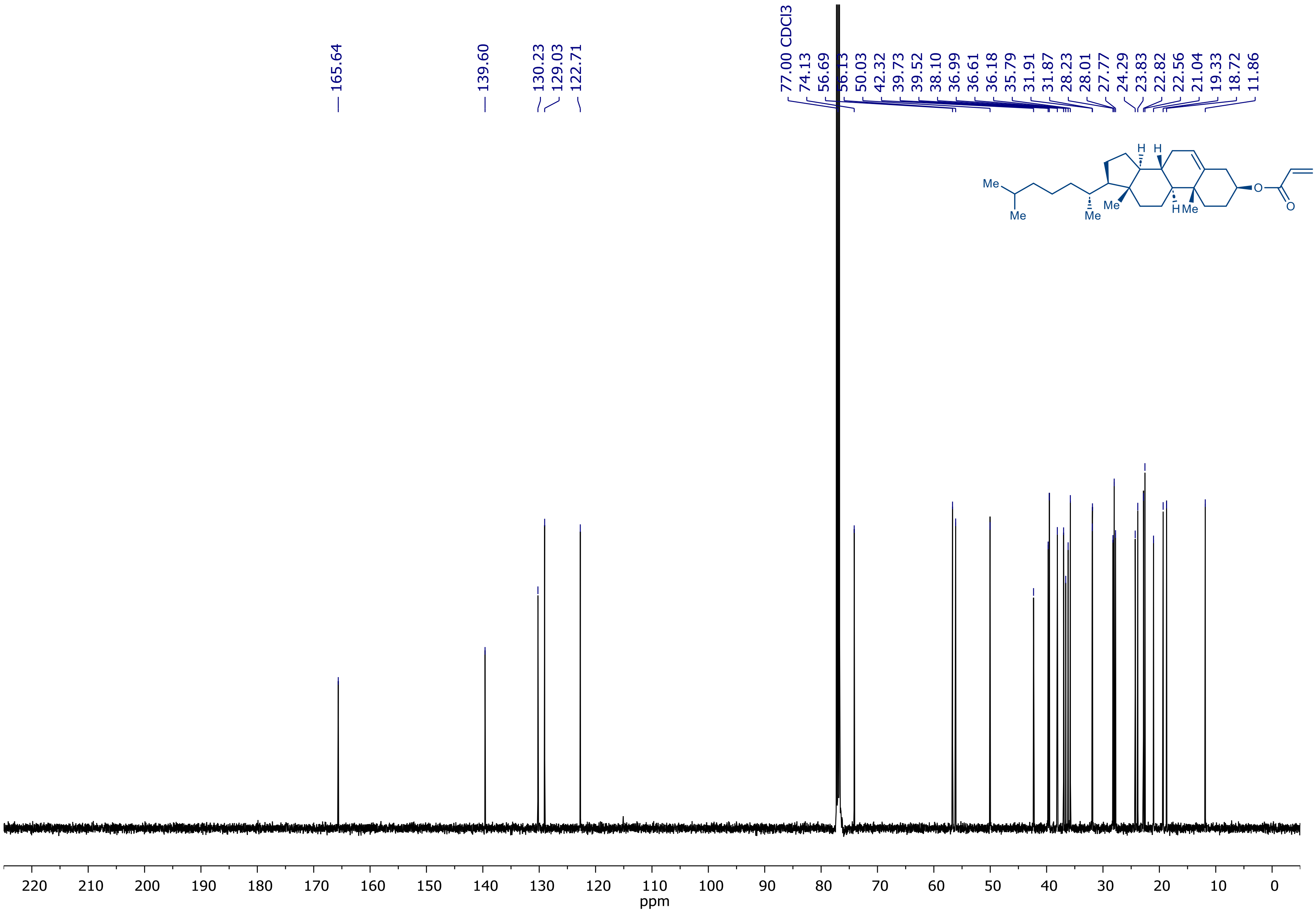

COSY of compound **SI-2**

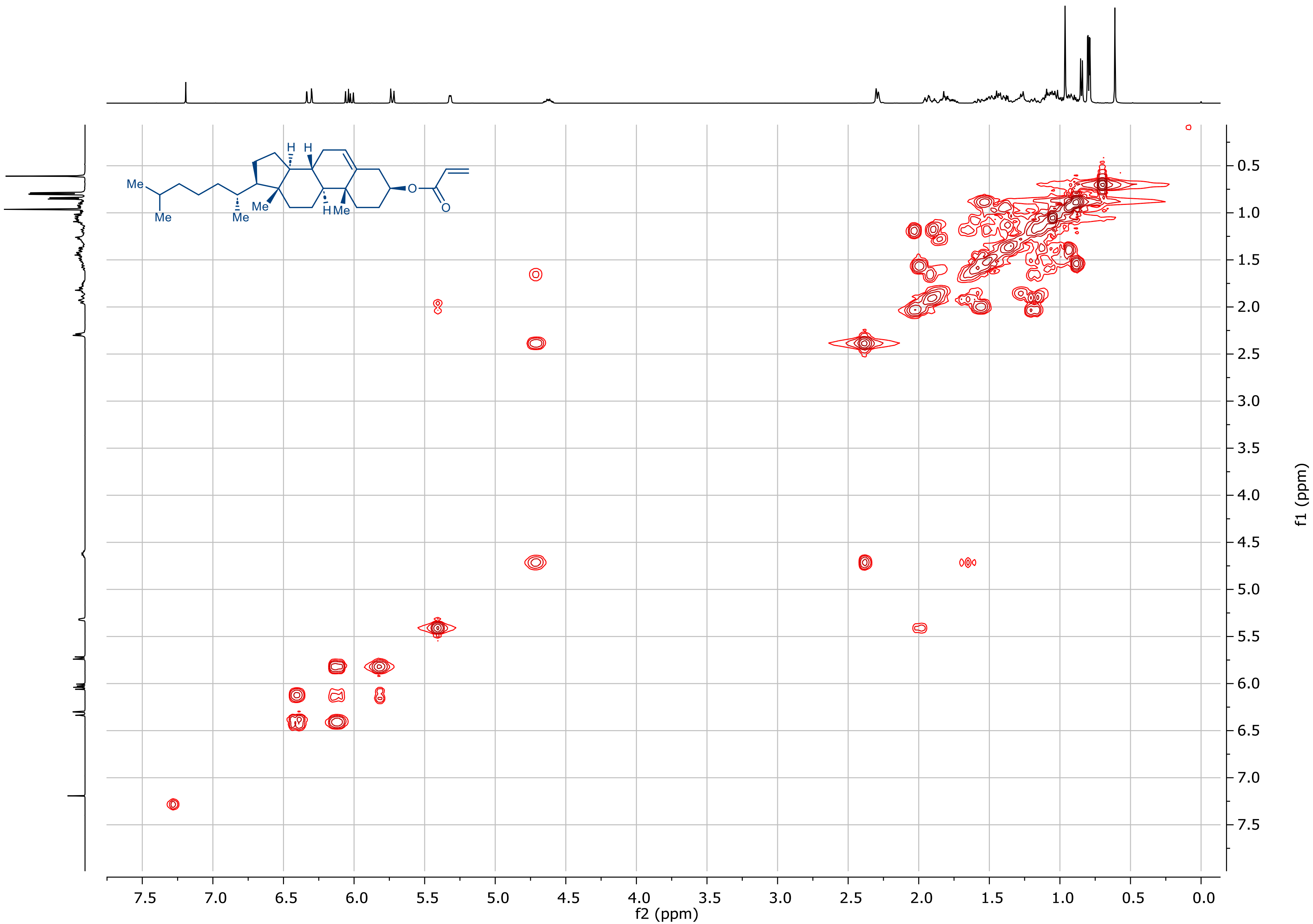

HSQC of compound **SI-2**

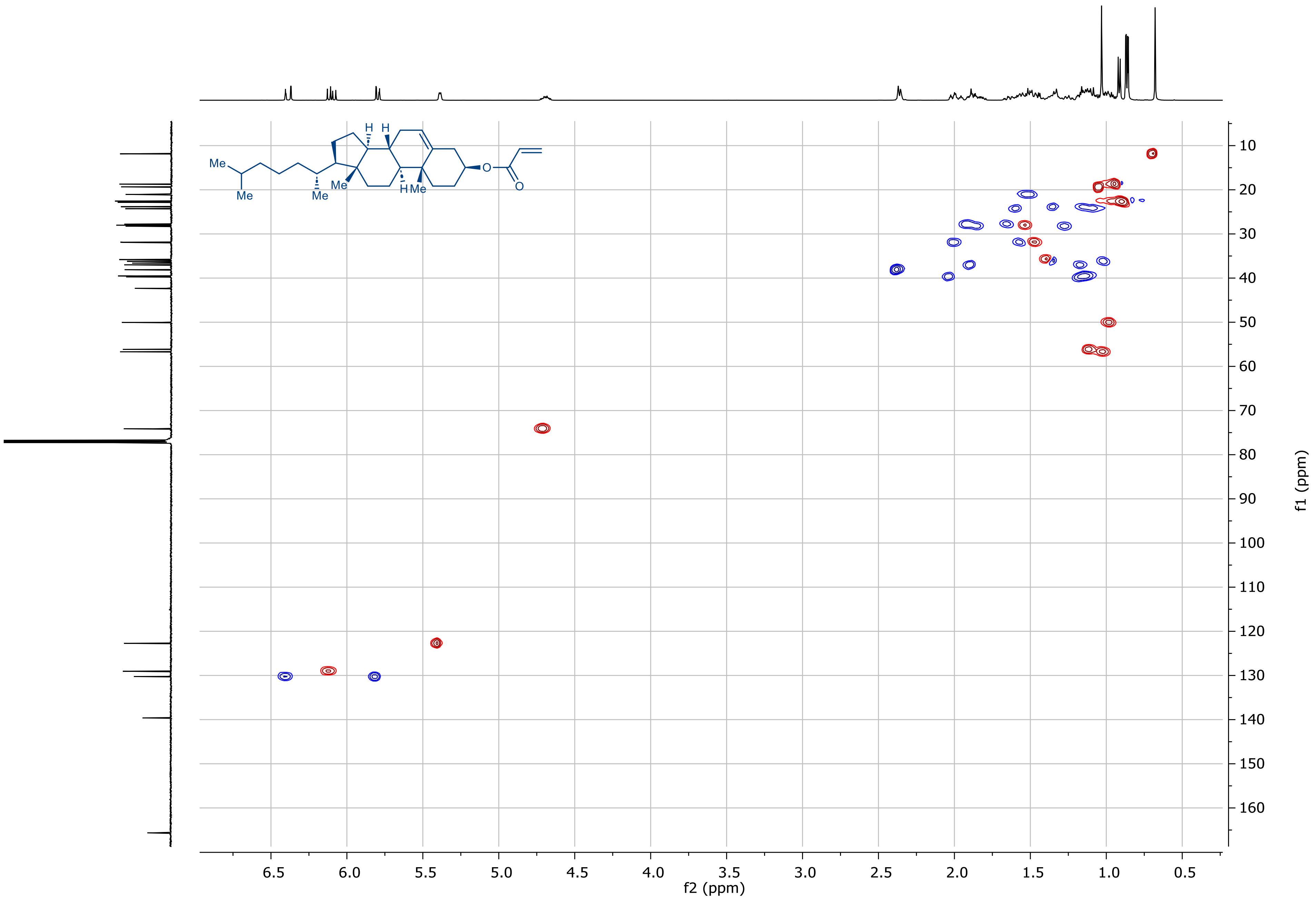

^{1}H NMR (500 MHz, $CDCl_3$) of compound **SI-3**

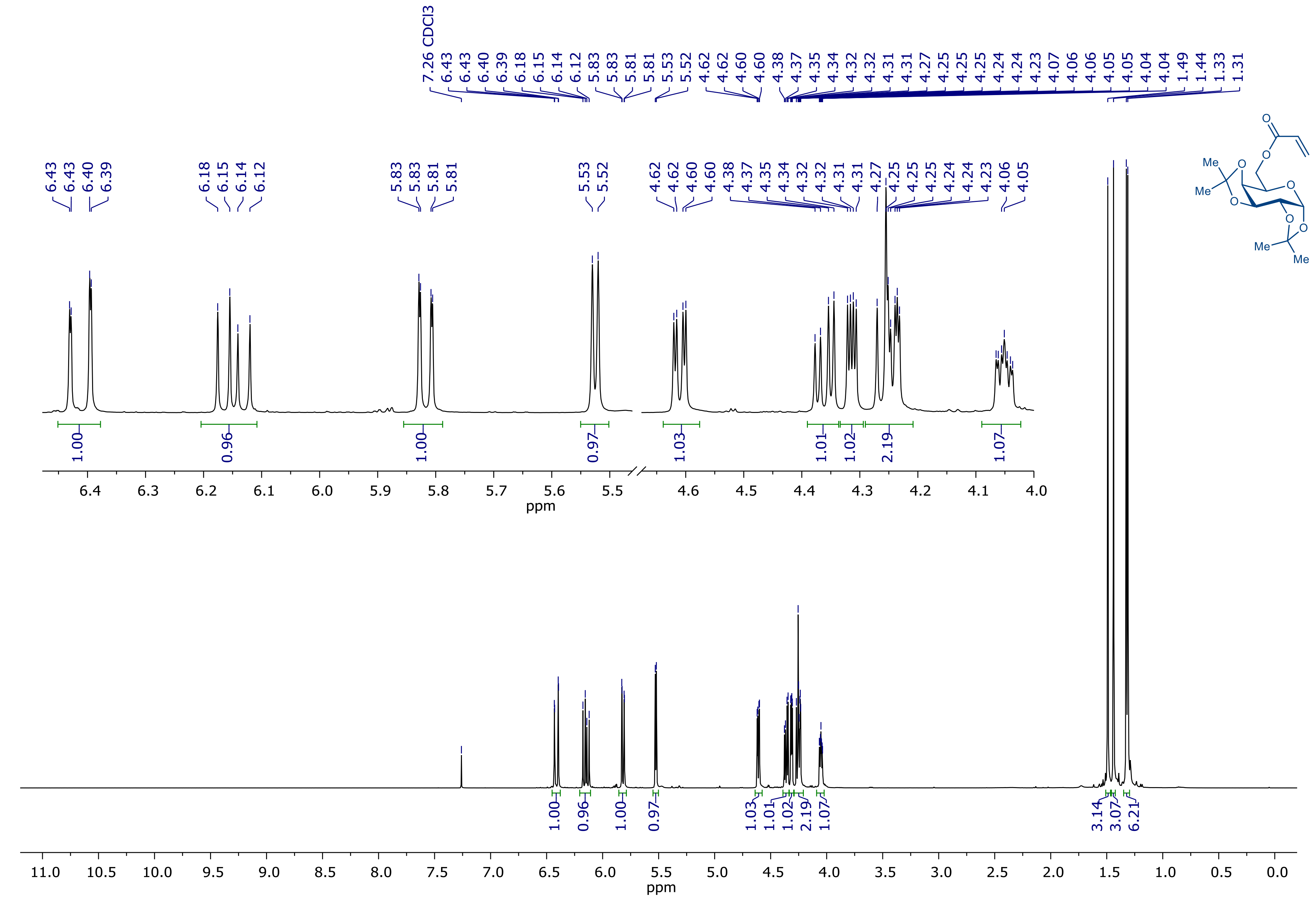

$^{13}C\{^1H\}$ NMR (126 MHz, $CDCl_3$) of compound **SI-3**

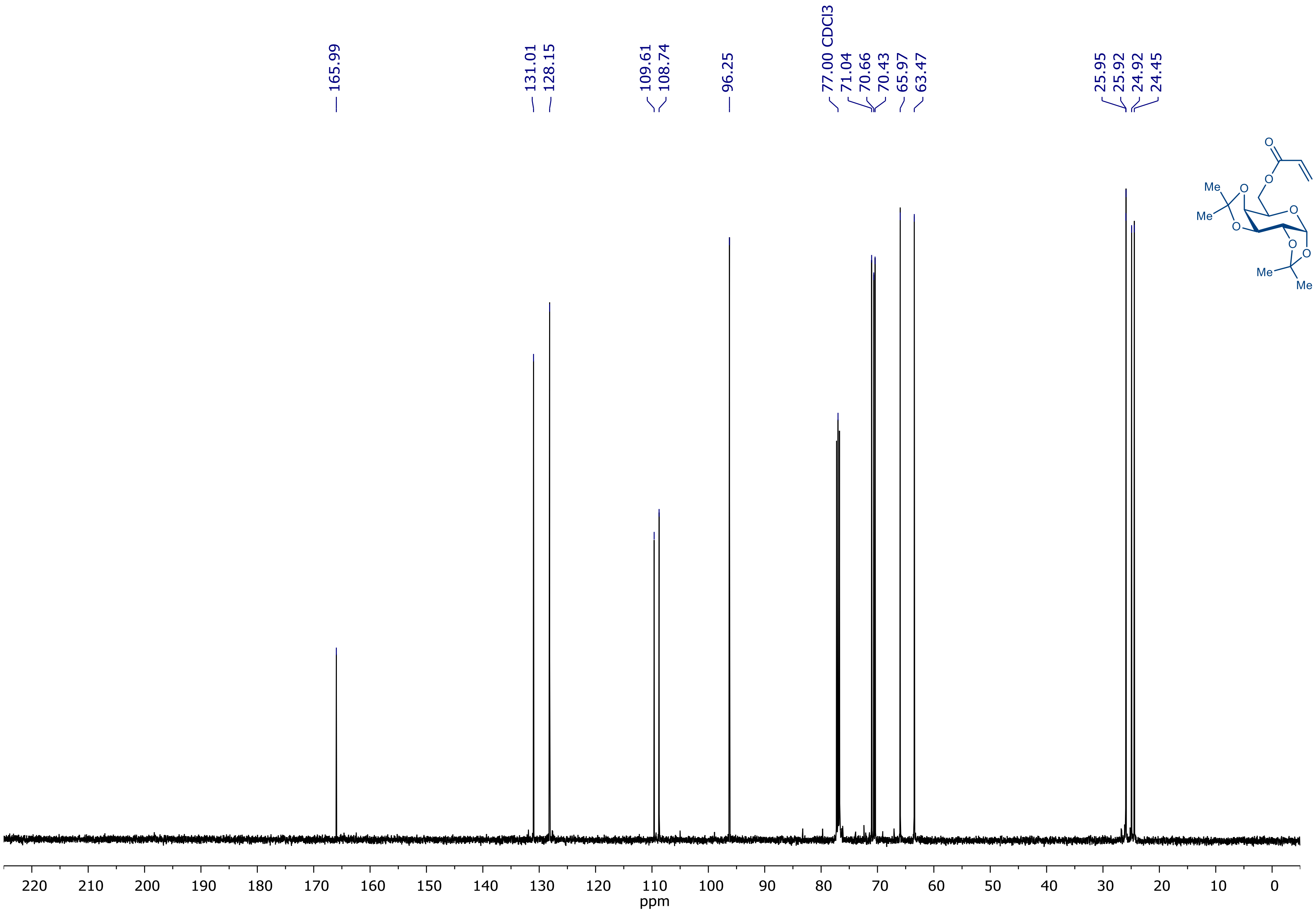

COSY of compound **SI-3**

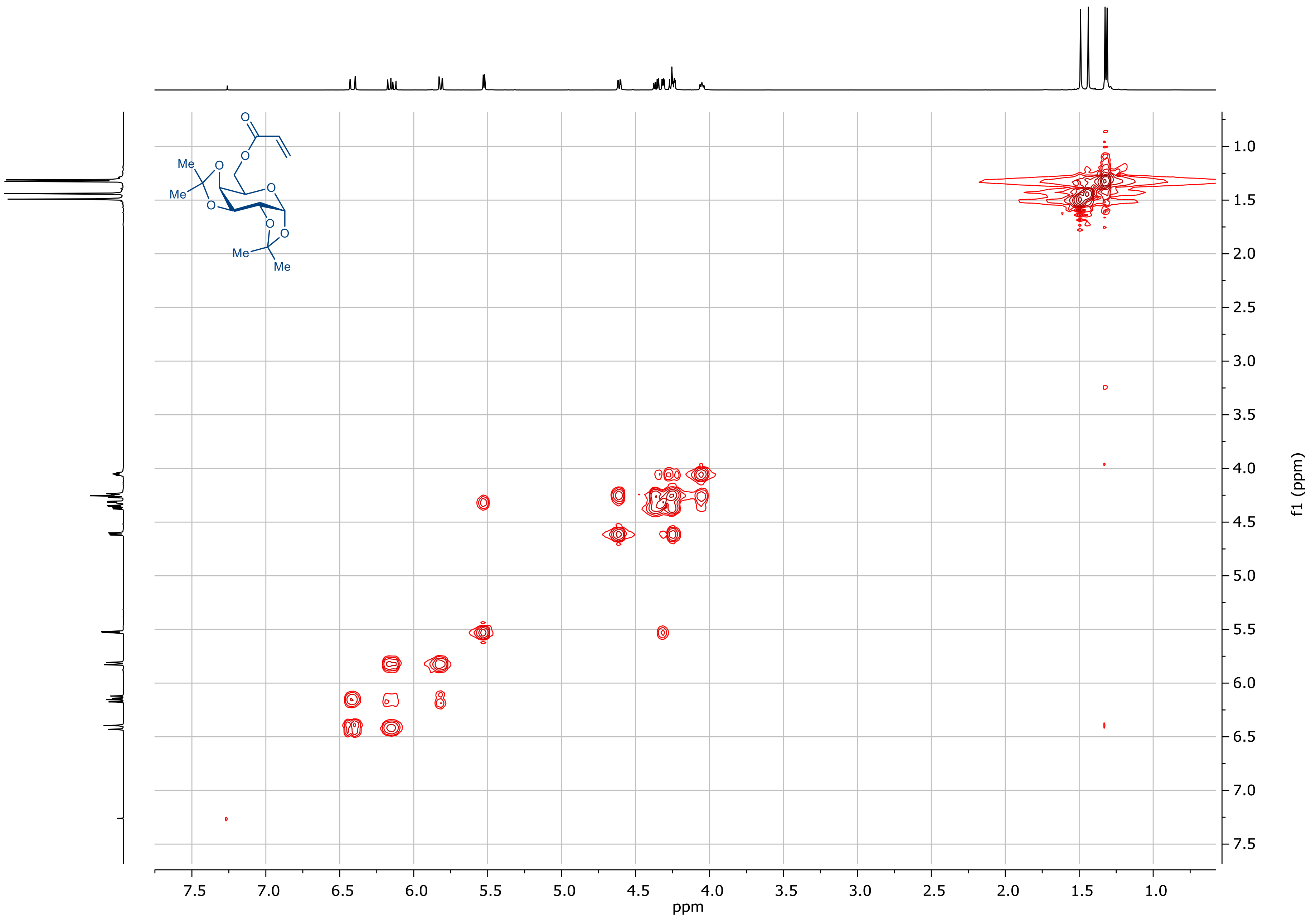

HSQC of compound **SI-3**

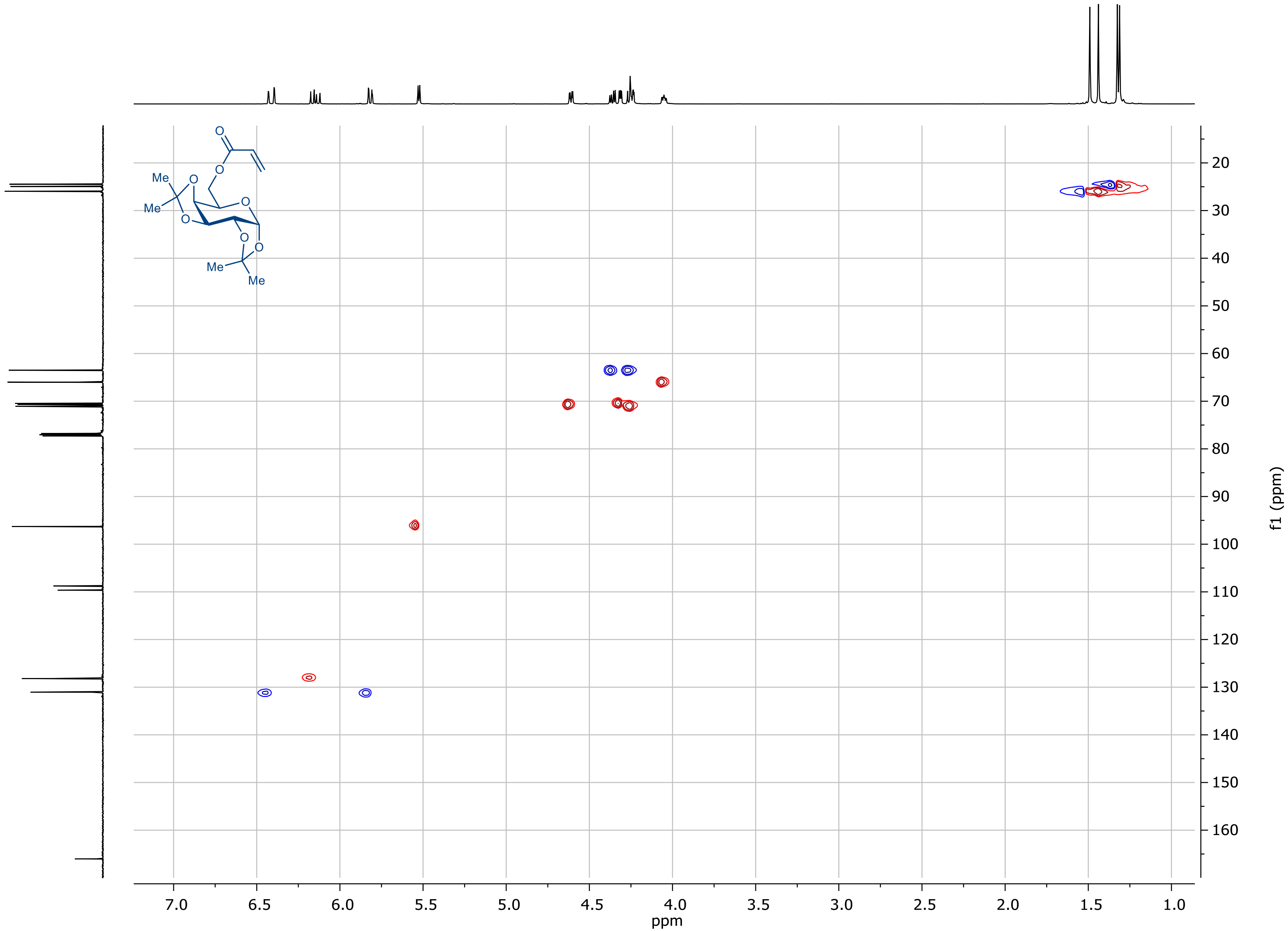

^{1}H NMR (500 MHz, CDCl$_3$) of compound **SI-4**

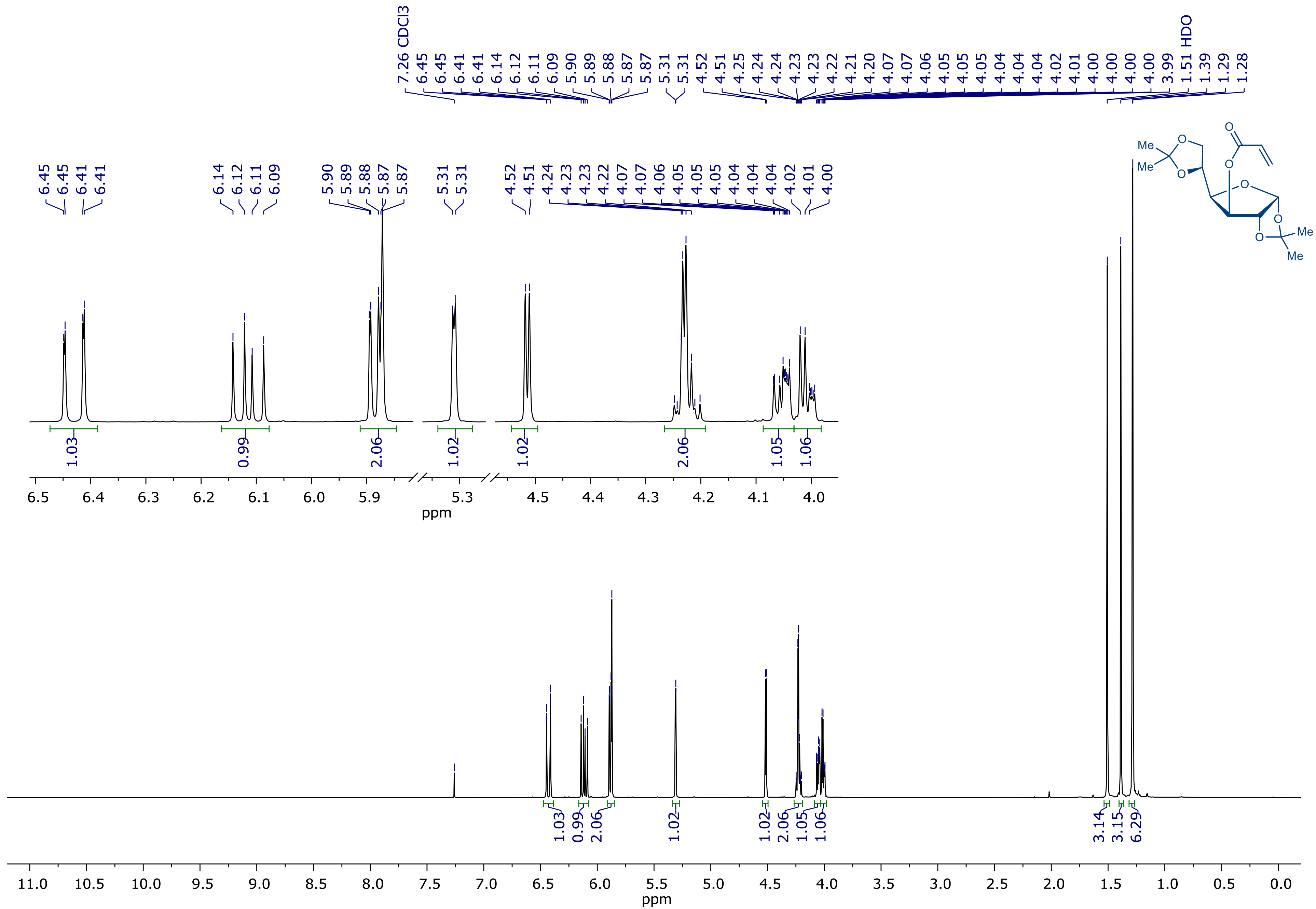

$^{13}C\{^{1}H\}$ **NMR** (126 MHz, $CDCl_3$) of compound **SI-4**

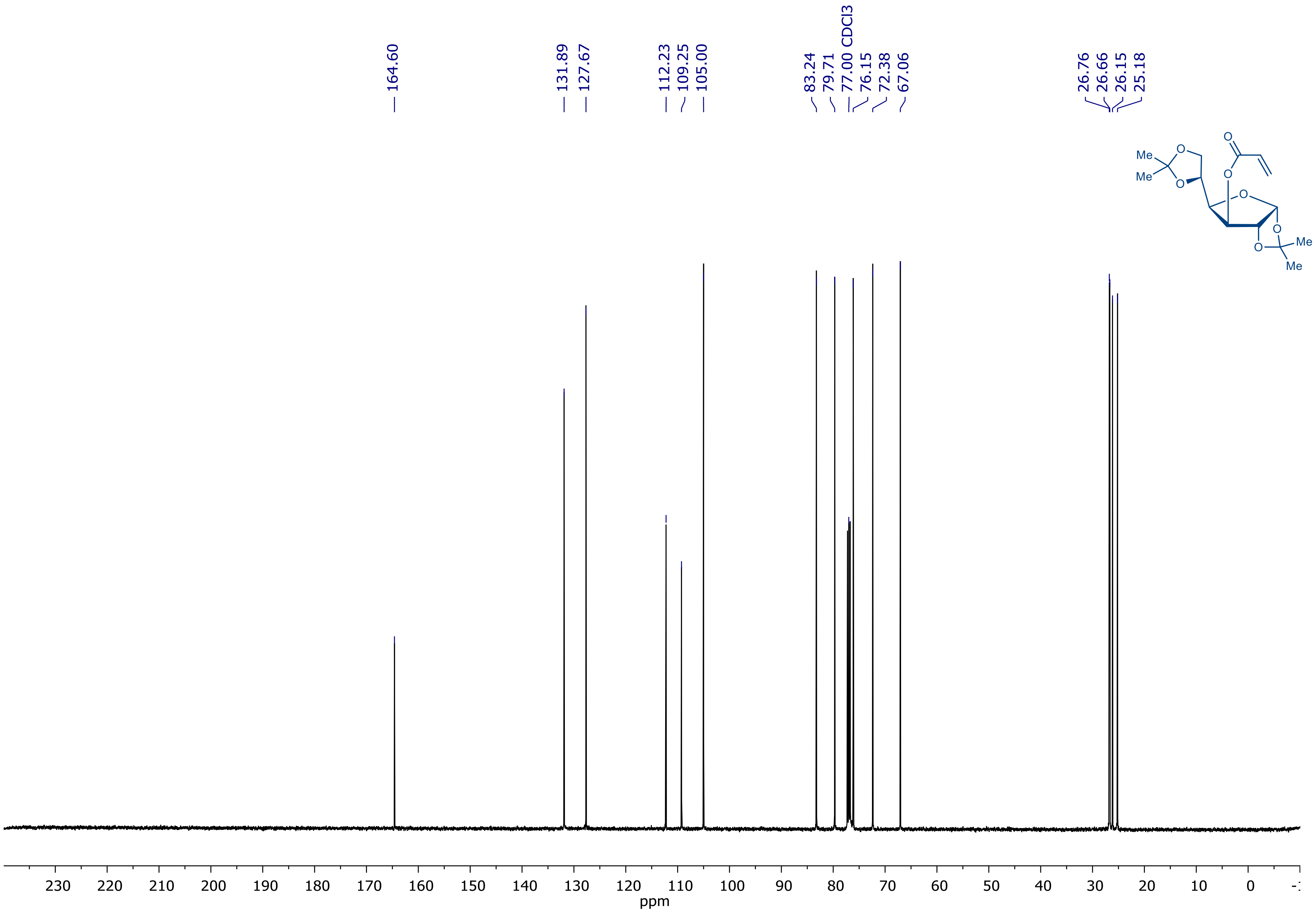

COSY of compound **SI-4**

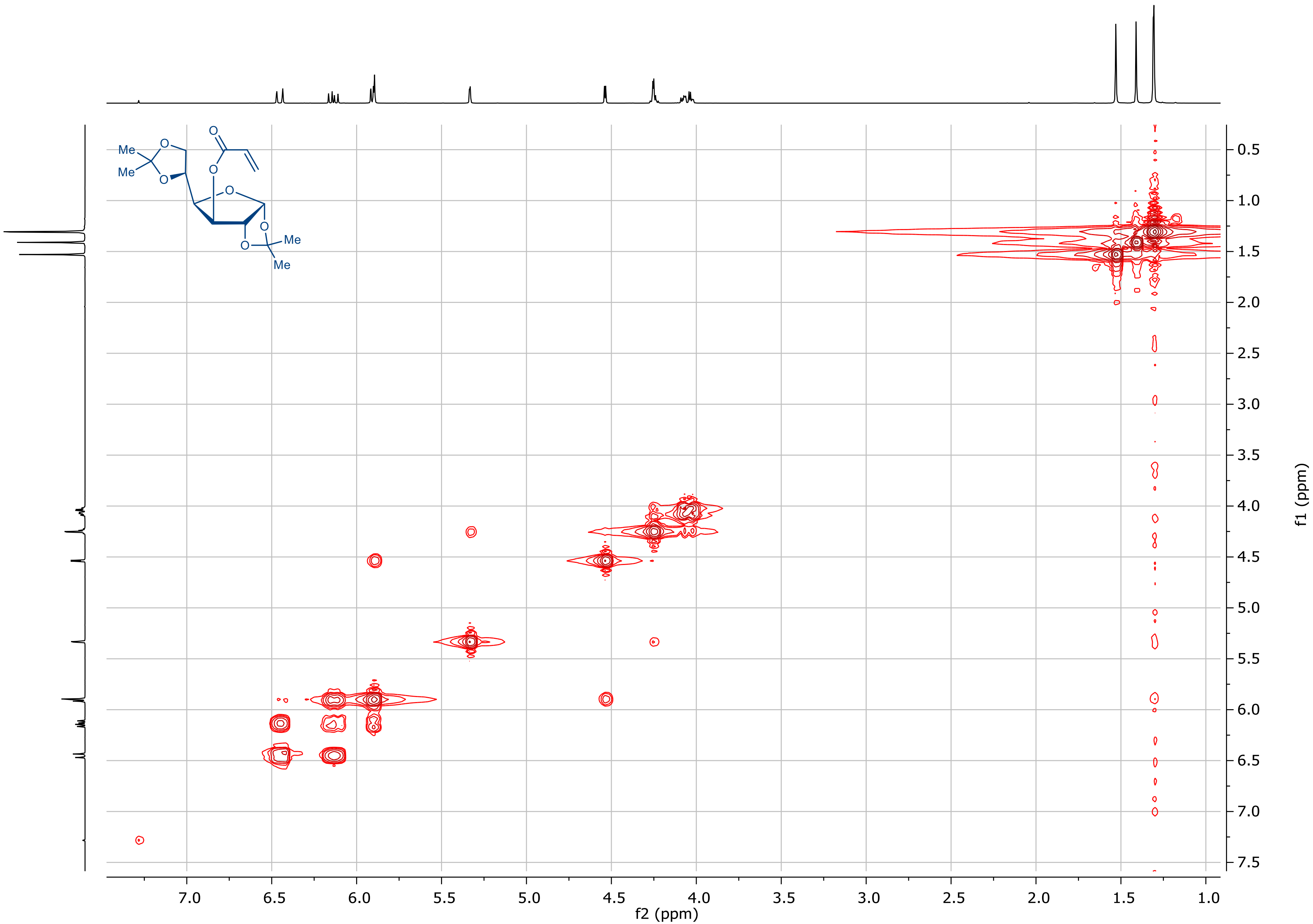

HSQC of compound **SI-4**

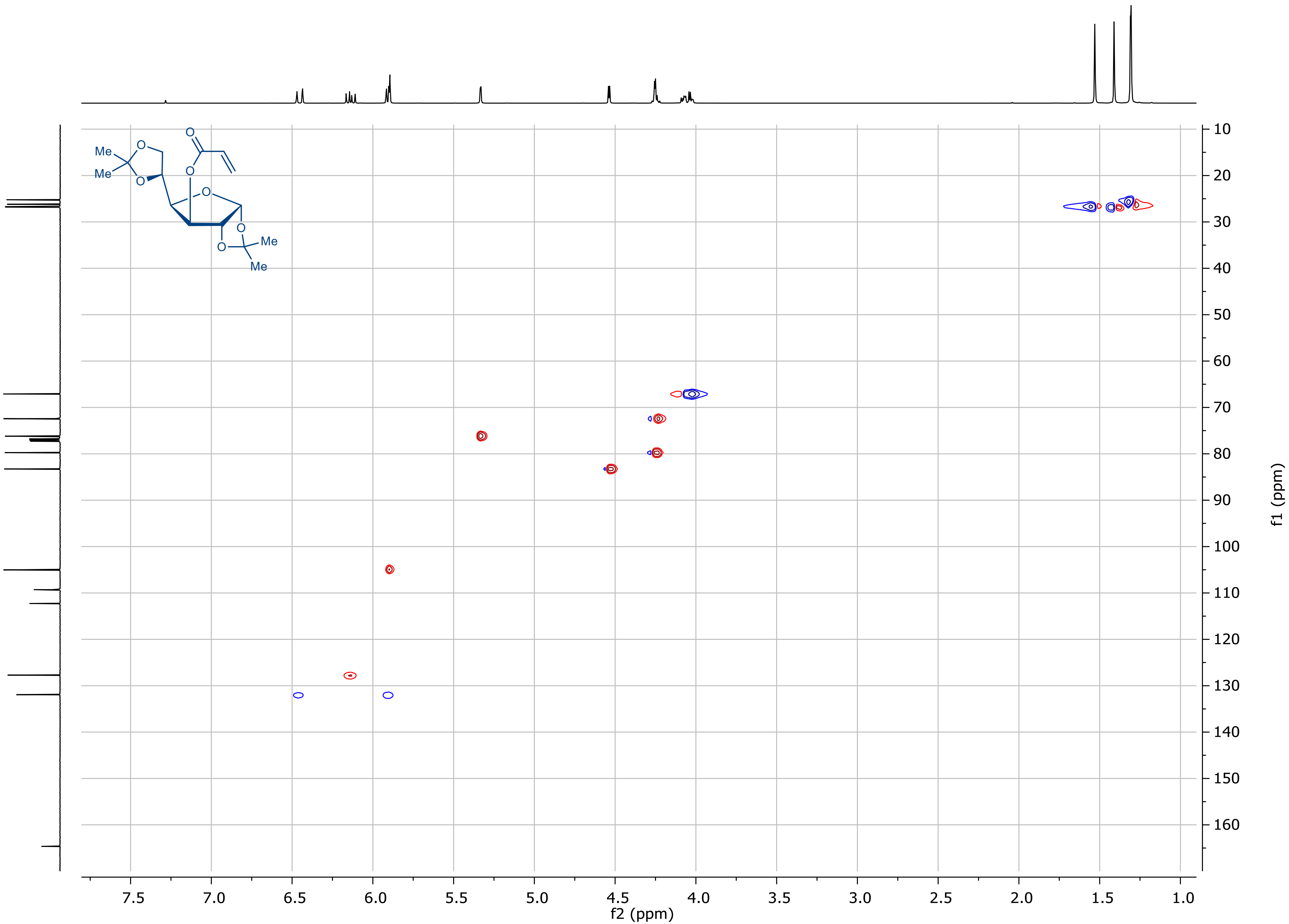

^{1}**H NMR** (500 MHz, $CDCl_3$) of compound **3a**

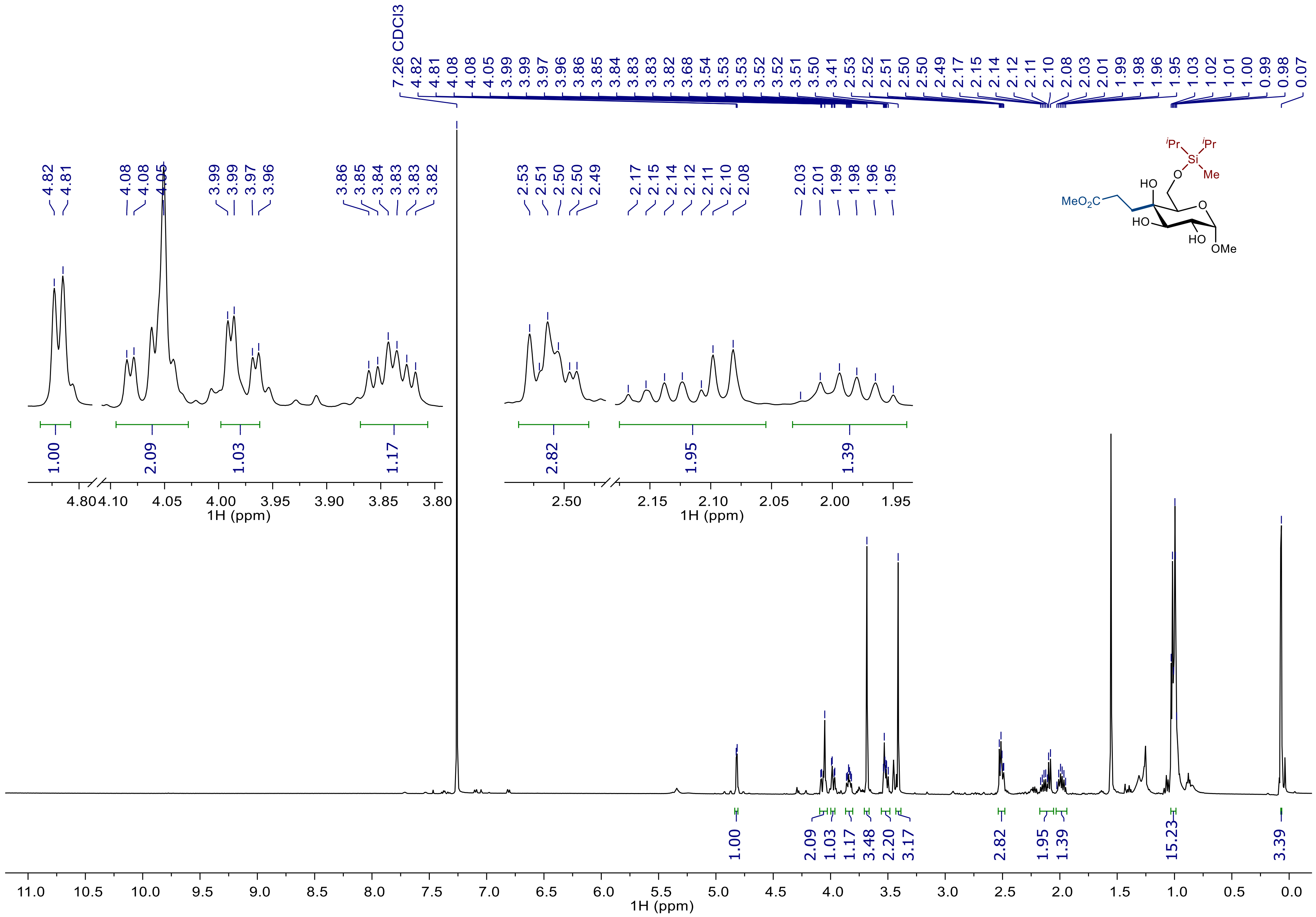

$^{13}C\{^1H\}$ NMR (126 MHz, $CDCl_3$) of compound **3a**

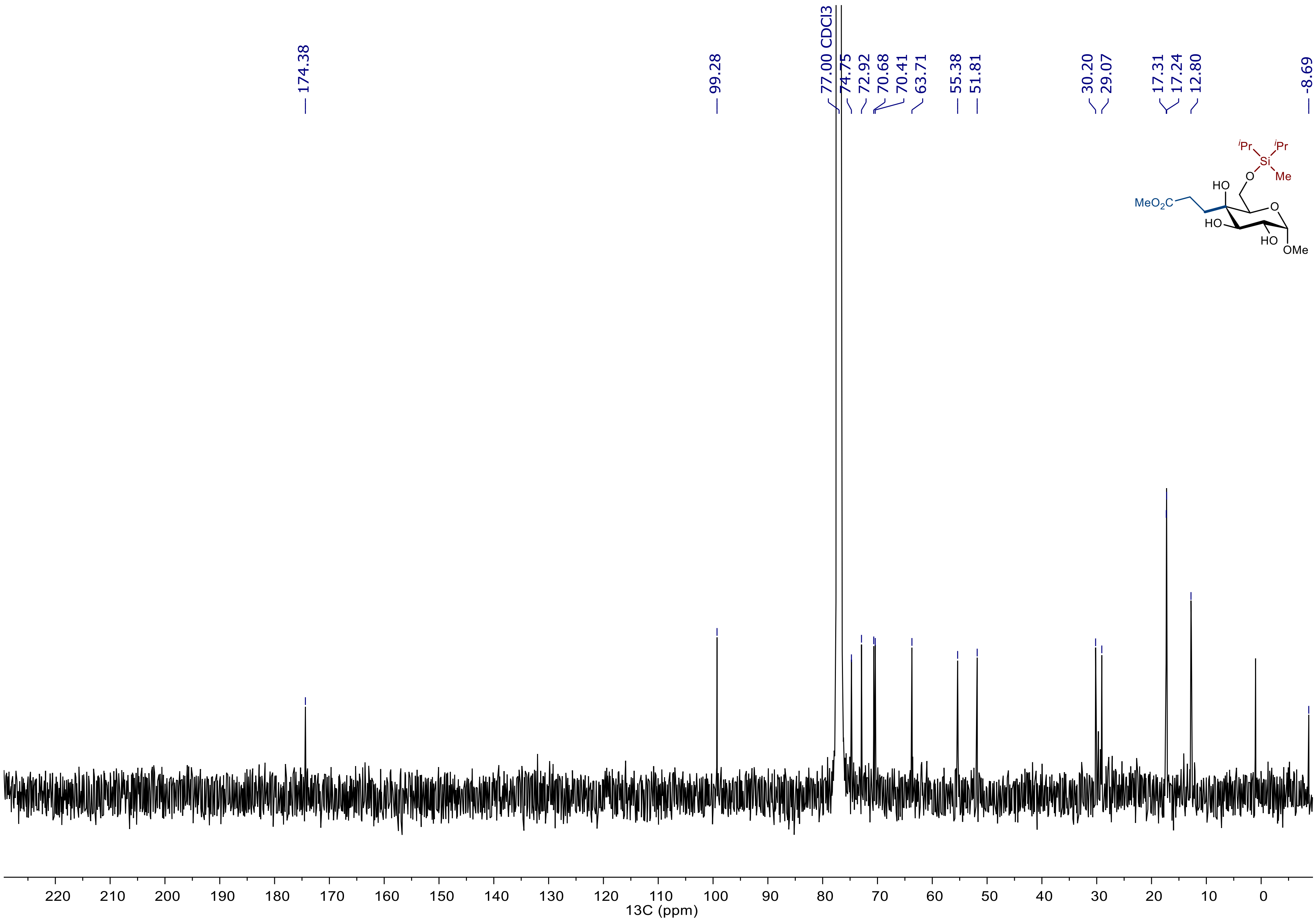

COSY of compound **3a**

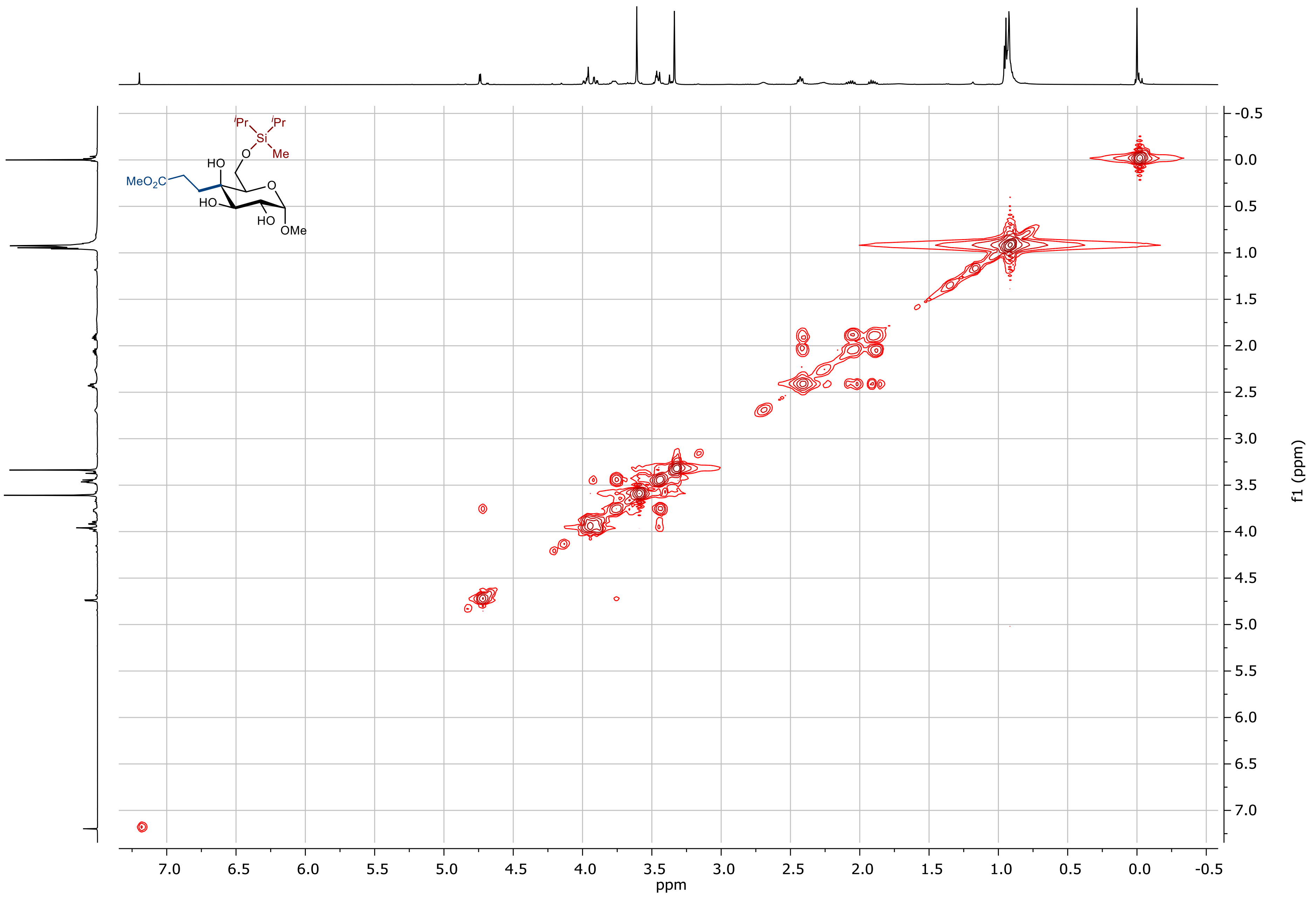

HSQC of compound **3a**

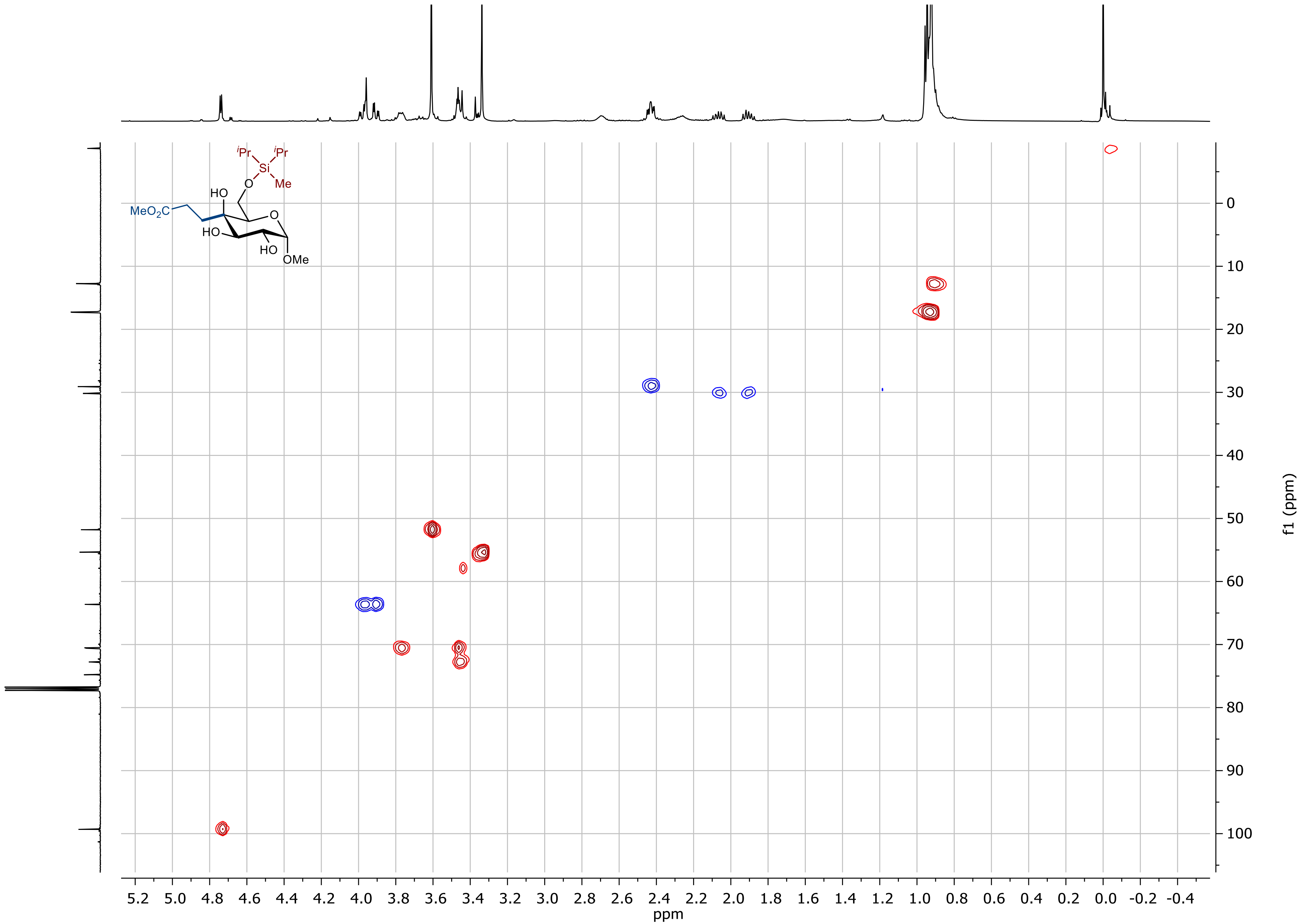

HMBC of compound **3a**

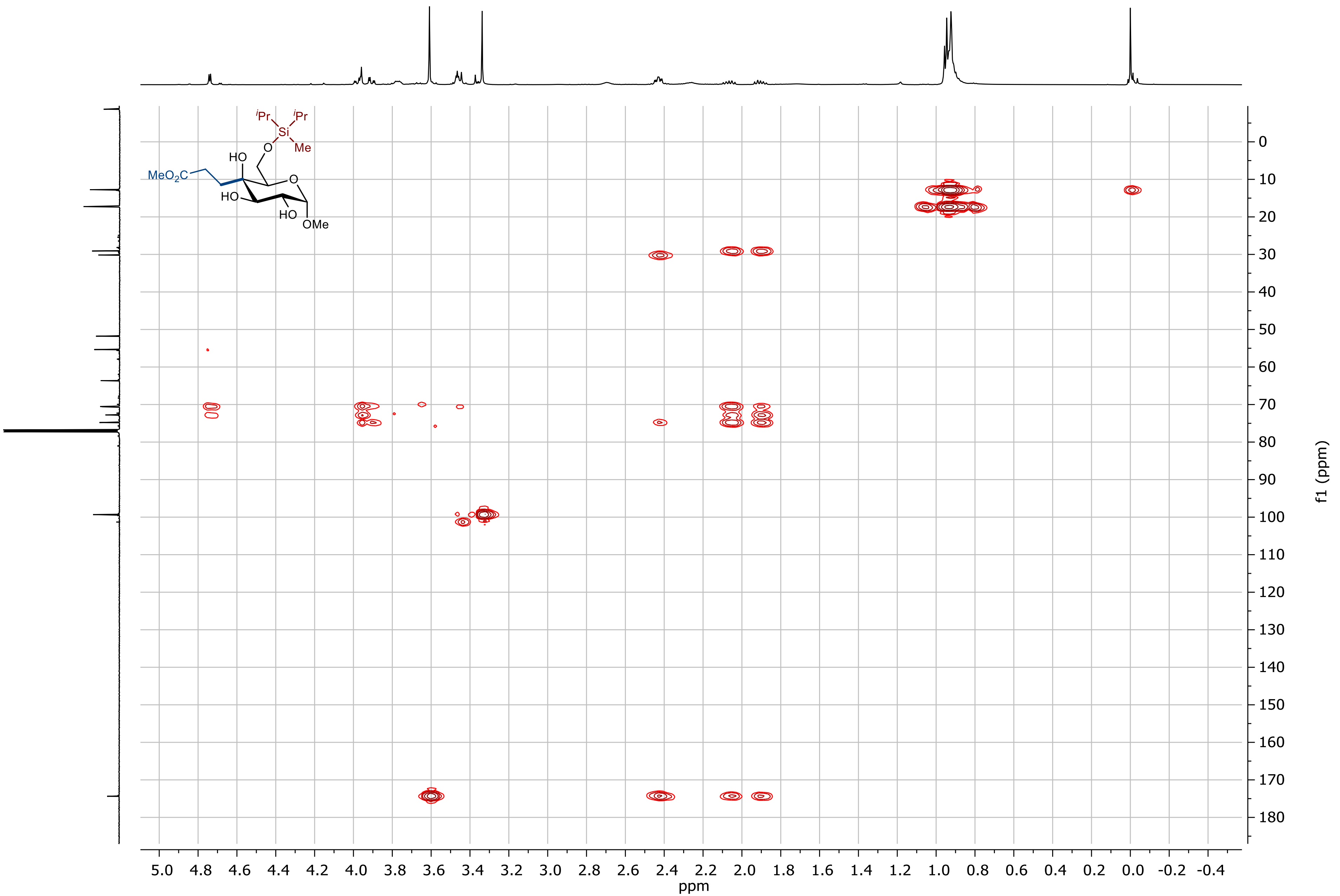

¹H NMR (500 MHz, $CDCl_3$) of compound **4a**

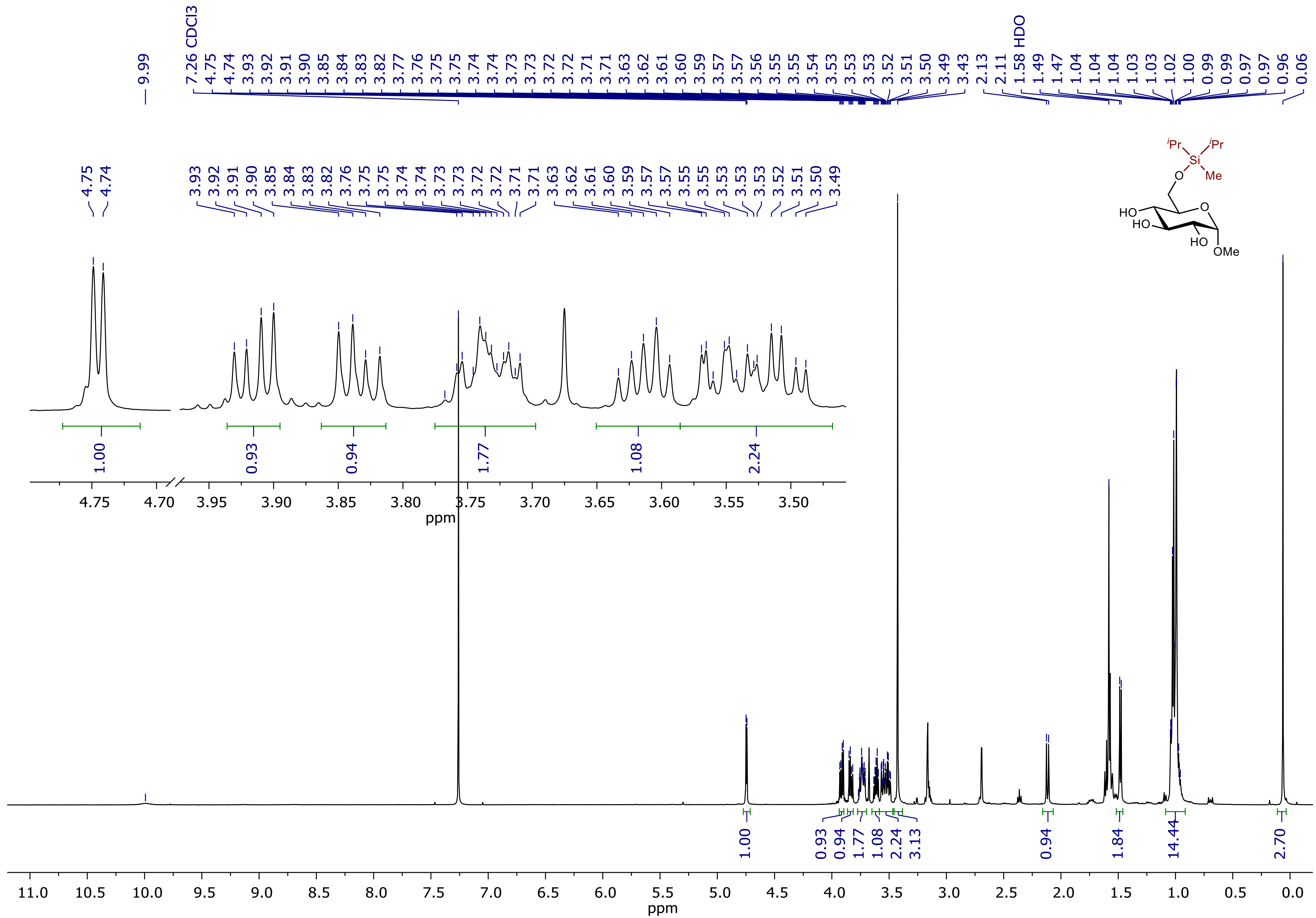

$^{13}C\{^{1}H\}$ NMR (126 MHz, $CDCl_3$) of compound **4a**

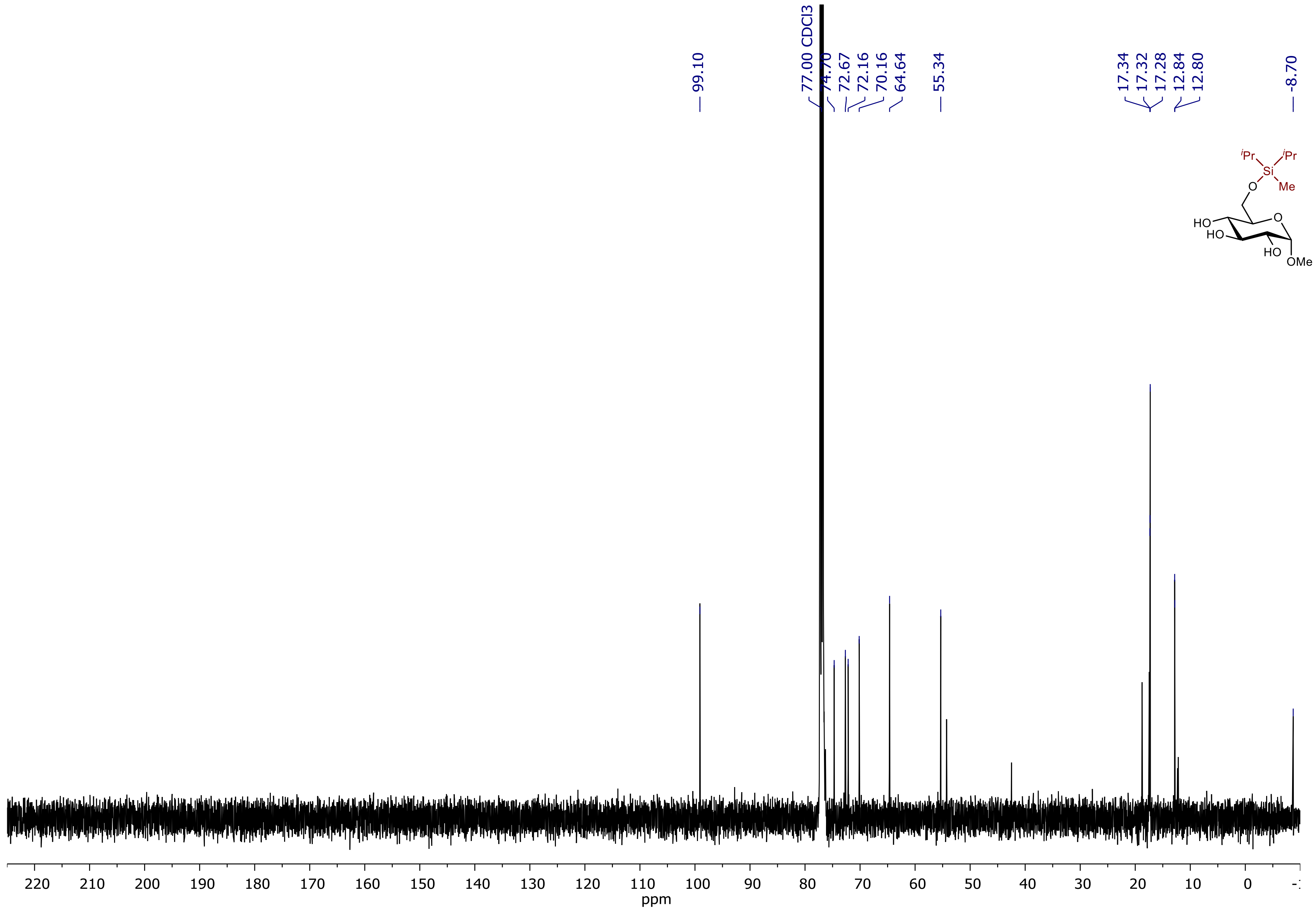

COSY of compound **4a**

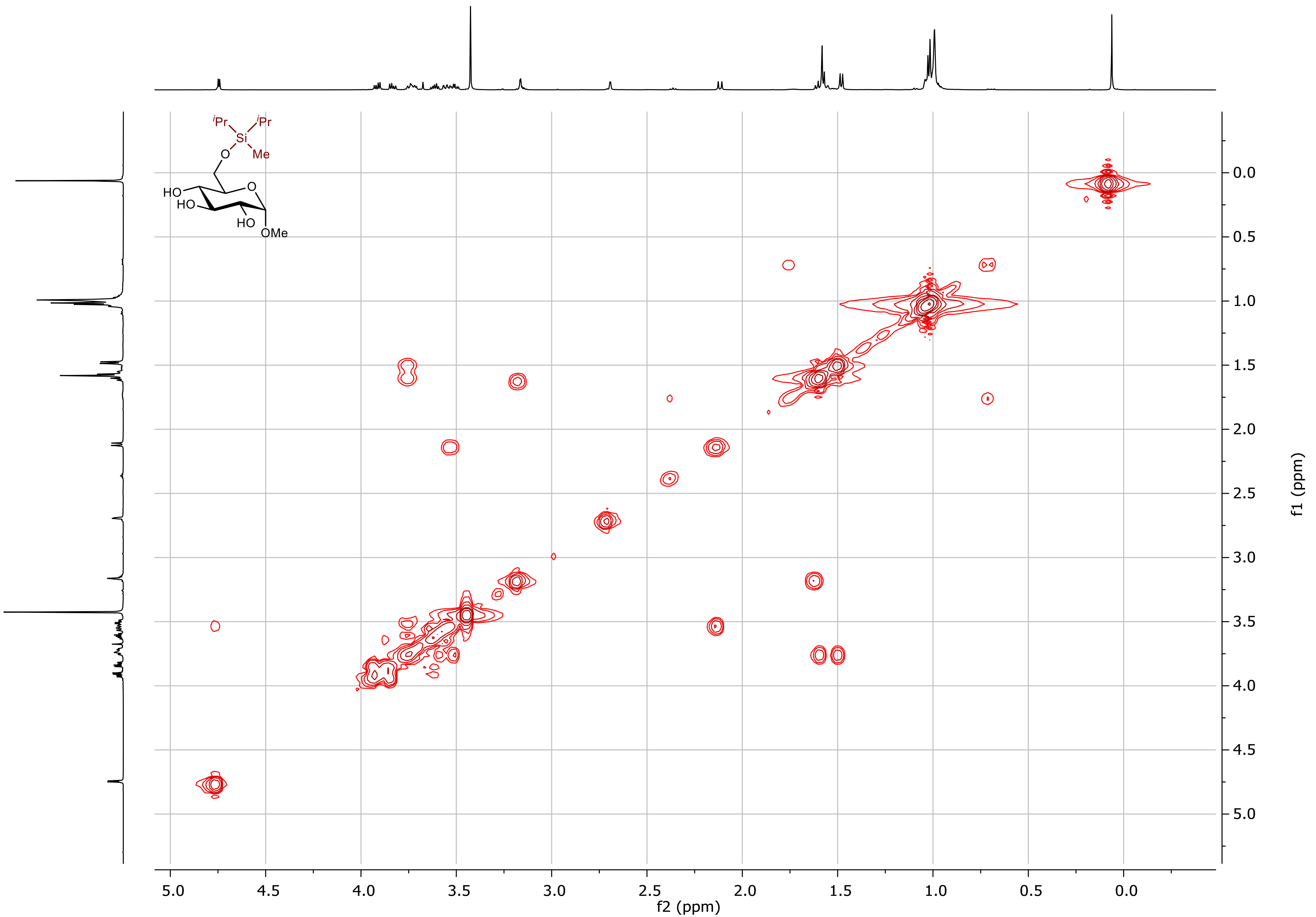

HSQC of compound **4a**

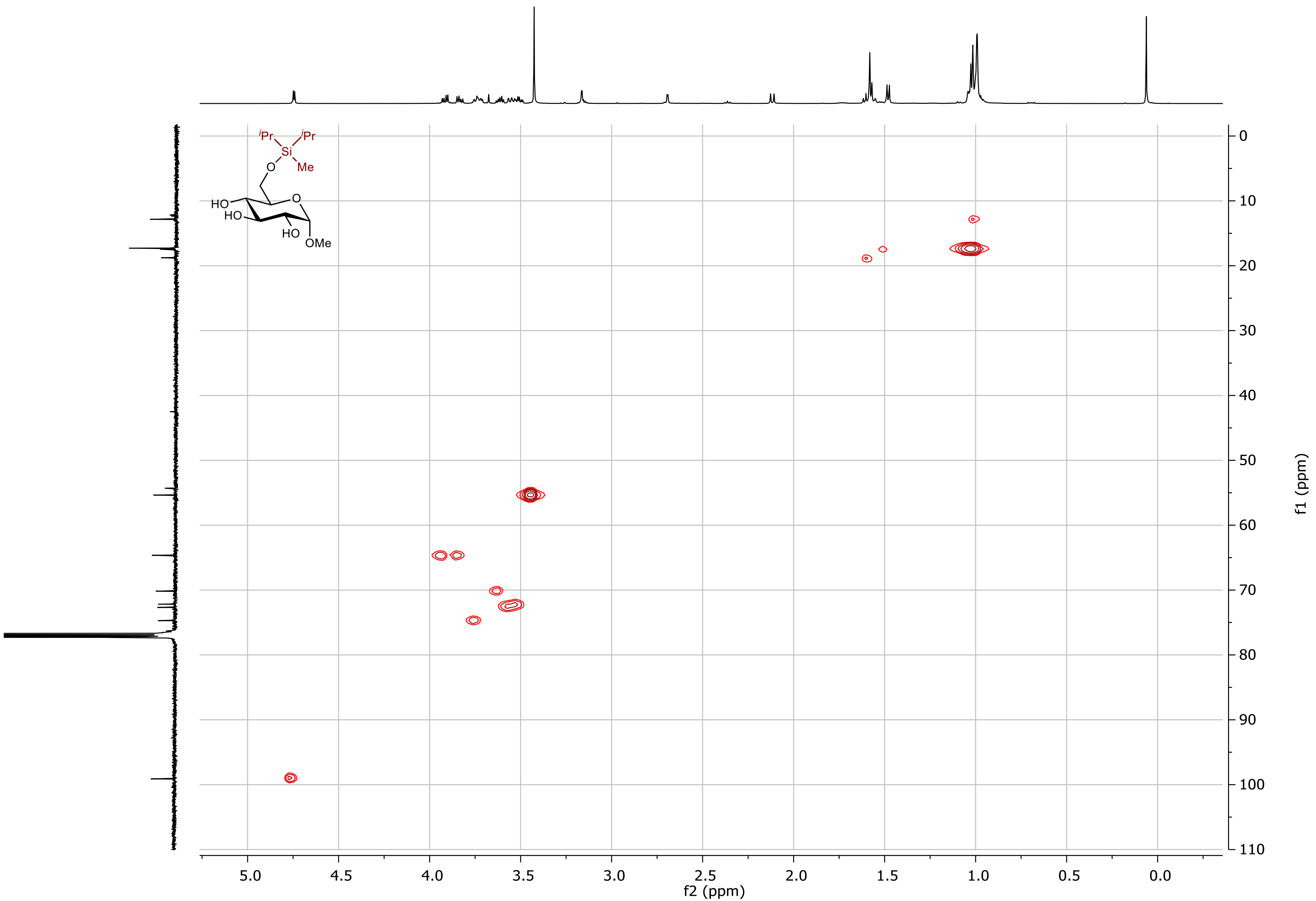

^{1}H NMR (500 MHz, $CDCl_3$) of compound **3b/3b'** (from **2c**)

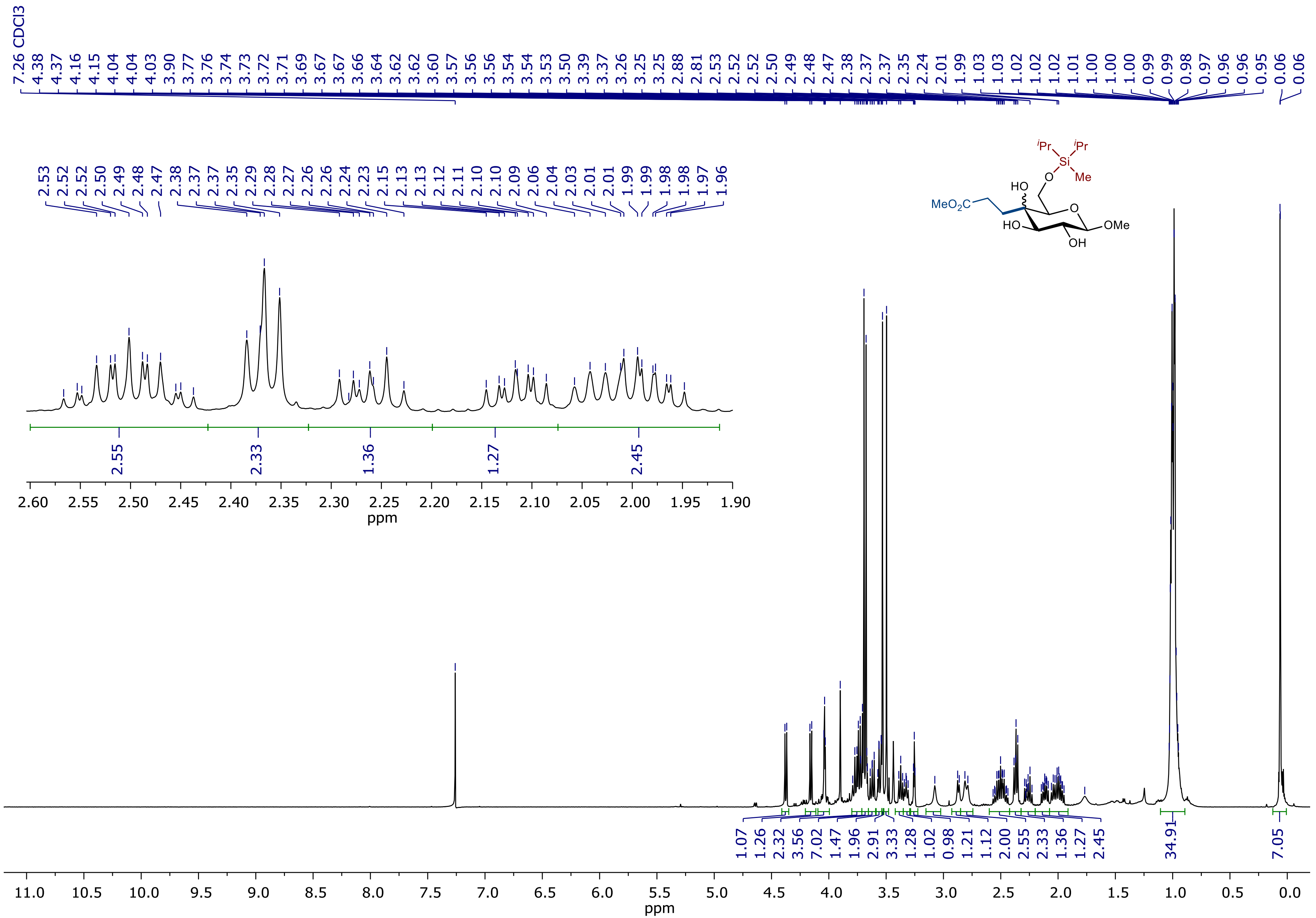

$^{13}C\{^1H\}$ NMR (126 MHz, $CDCl_3$) of compound **3b/3b'** (from **2c**)

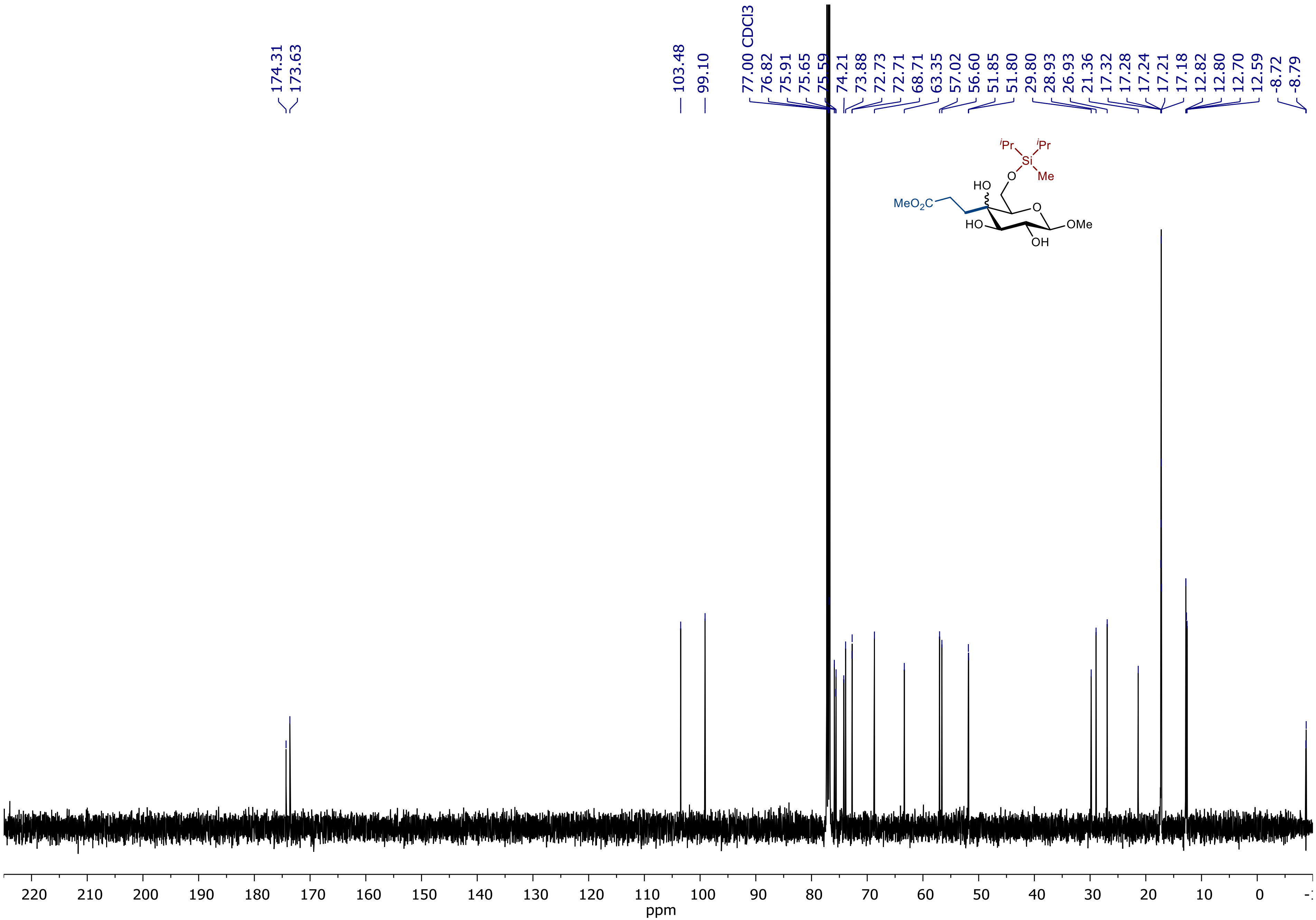

COSY of compound **3b/3b'** (from **2c**)

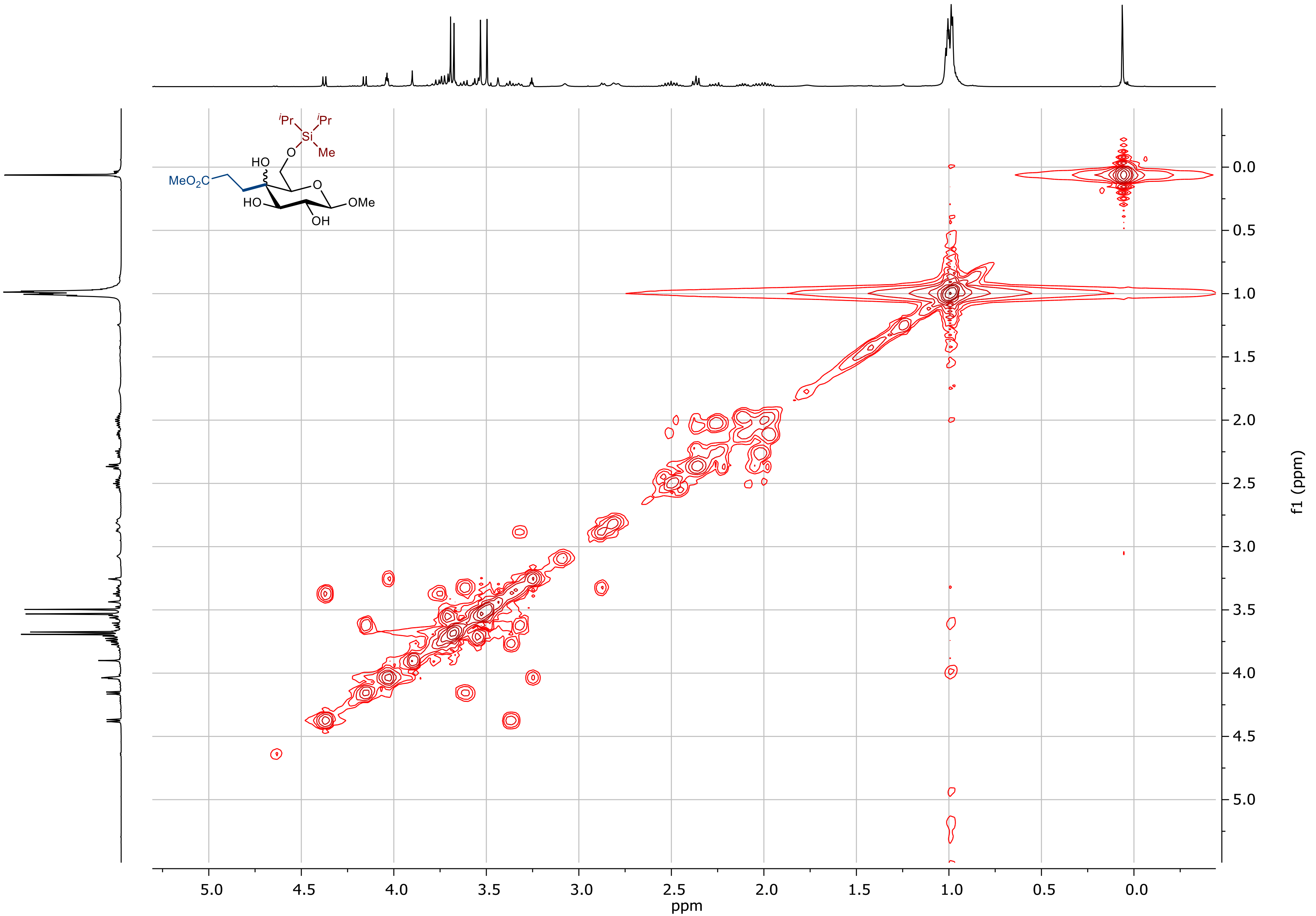

HSQC of compound **3b/3b'** (from **2c**)

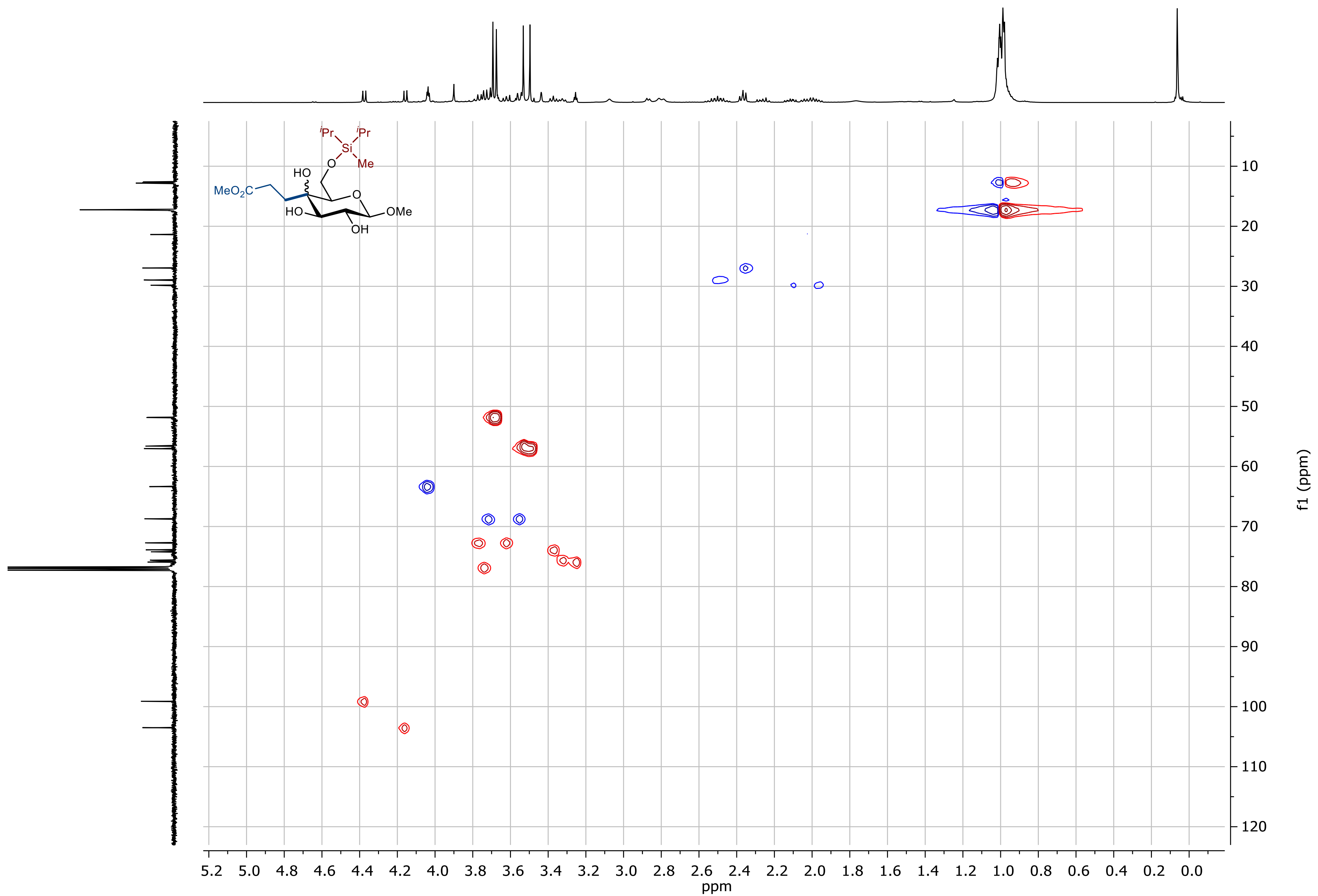

^{1}H NMR (500 MHz, $CDCl_3$) of compound **3b/3b'** (from **2d**)

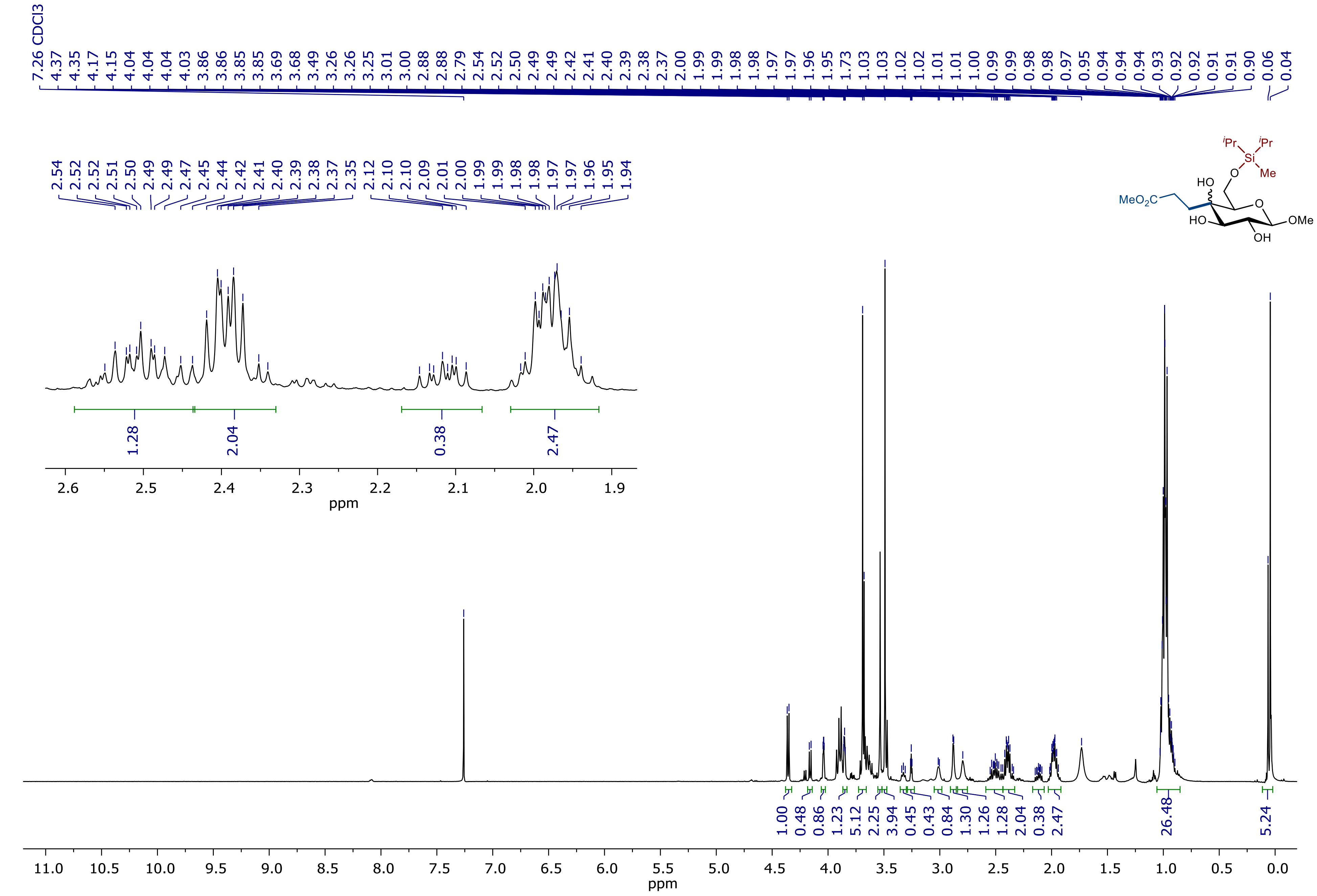

^{1}**H NMR** (500 MHz, $CDCl_3$) of compound **3c**

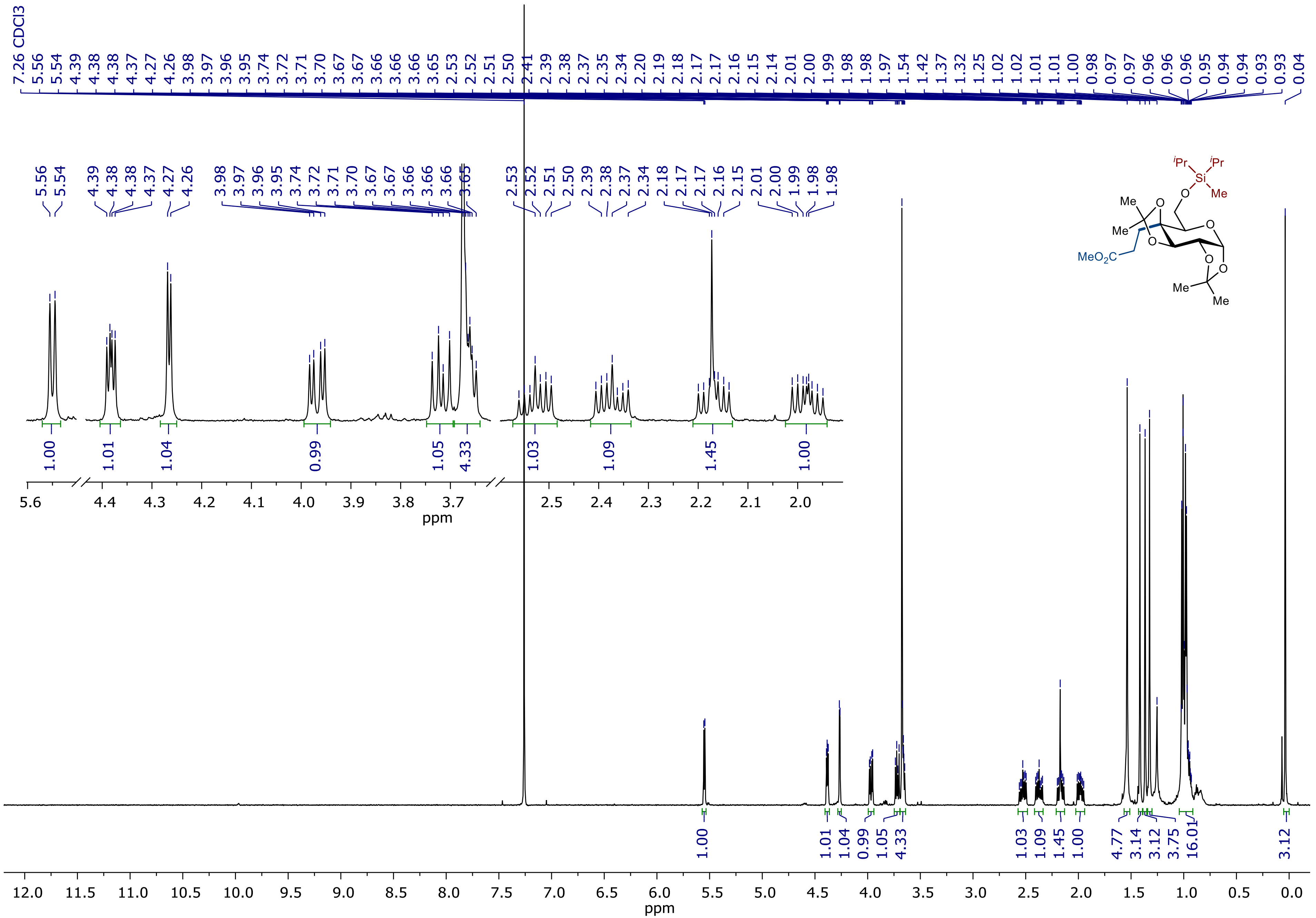

$^{13}C\{^{1}H\}$ NMR (126 MHz, $CDCl_3$) of compound **3c**

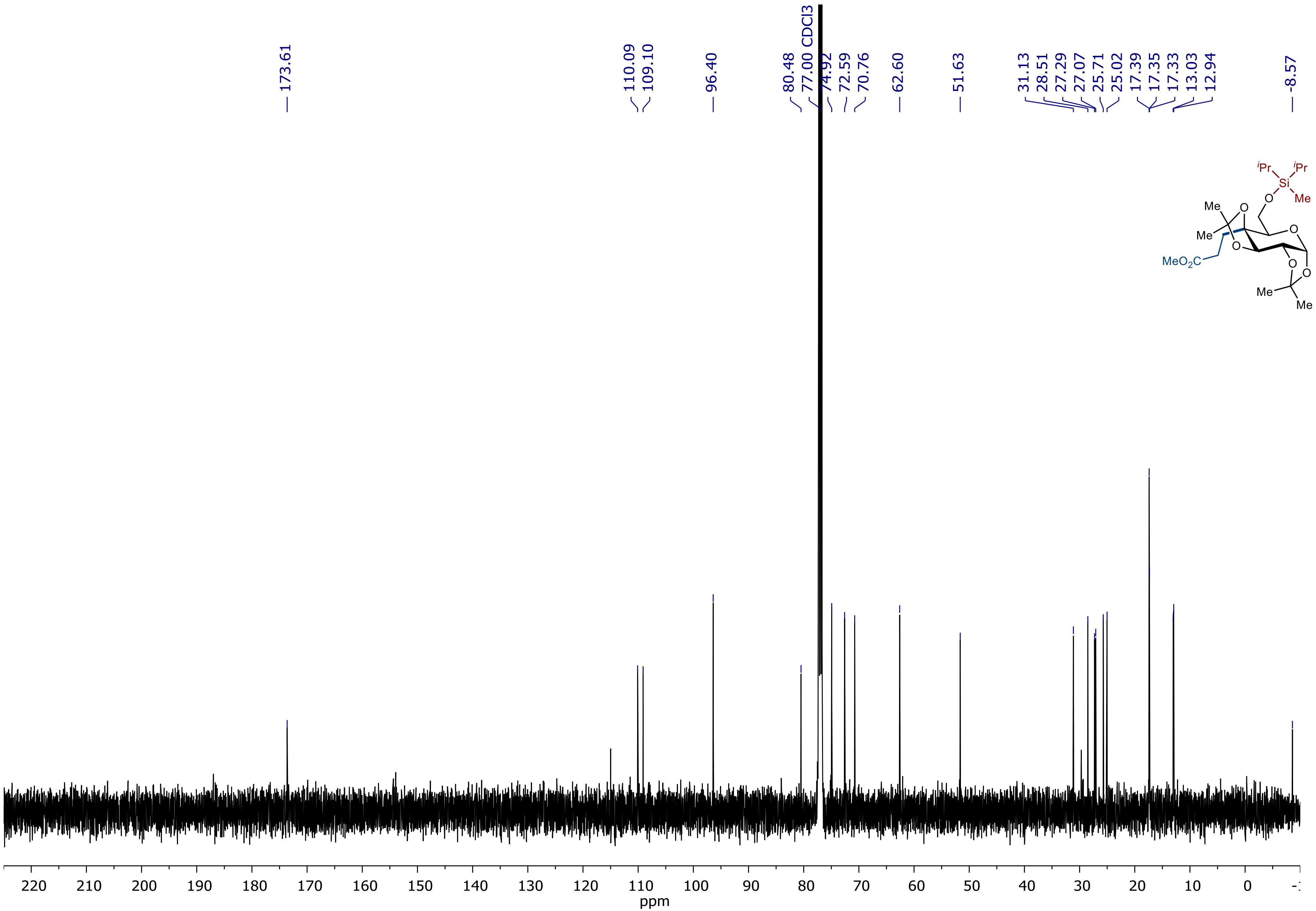

COSY of compound **3c**

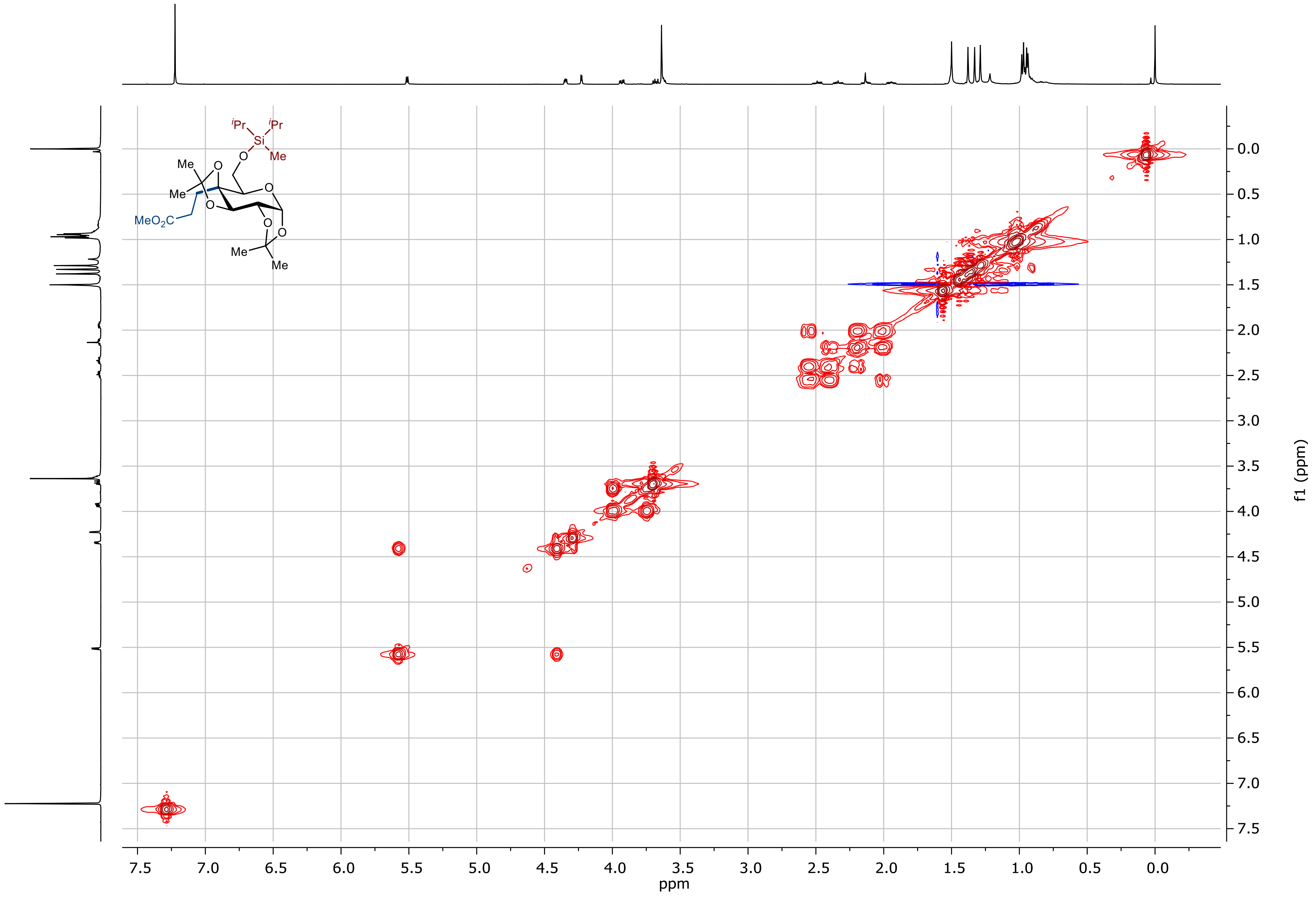

HSQC of compound **3c**

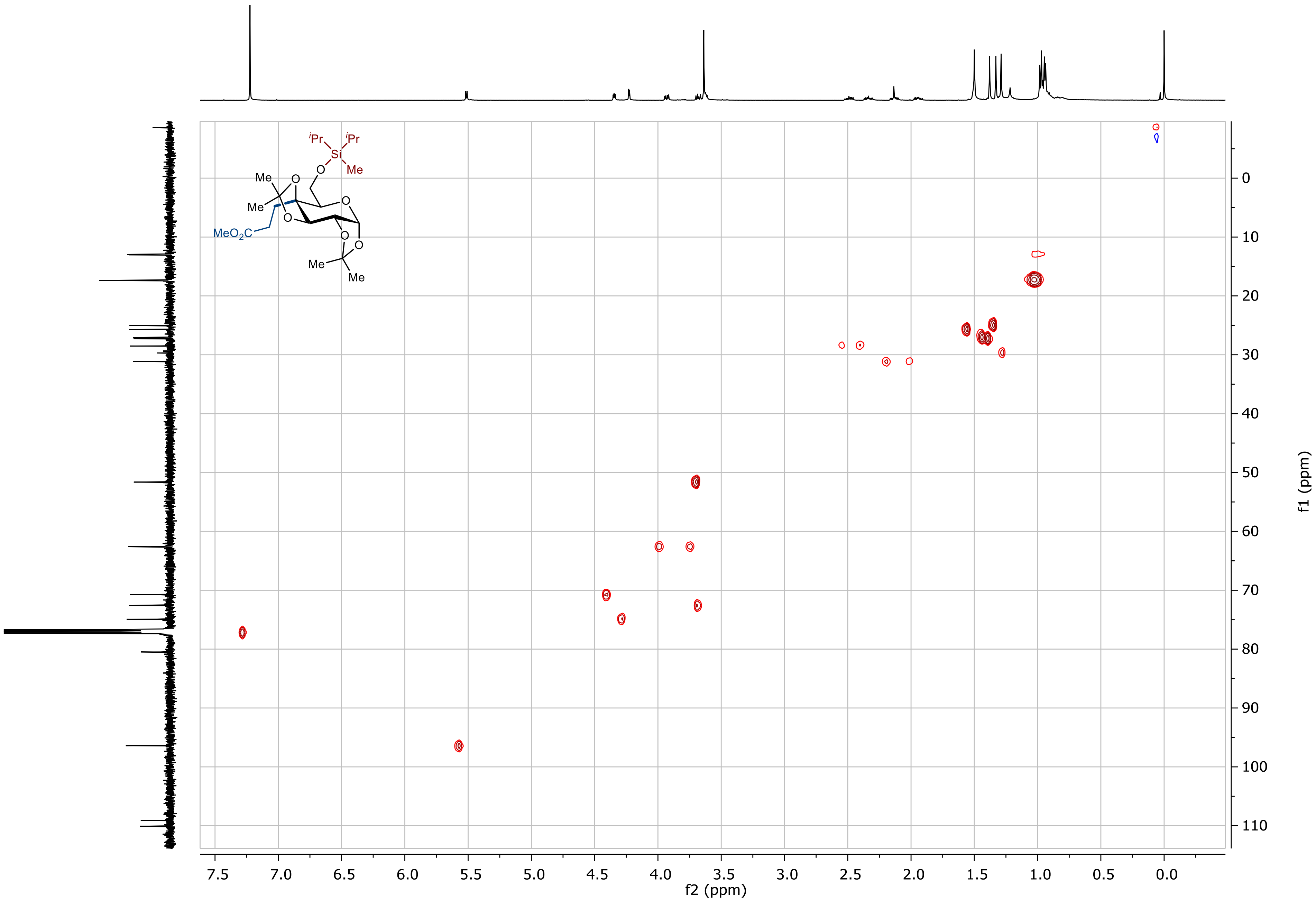

^{1}**H NMR** (500 MHz, $CDCl_3$) of compound **3d**

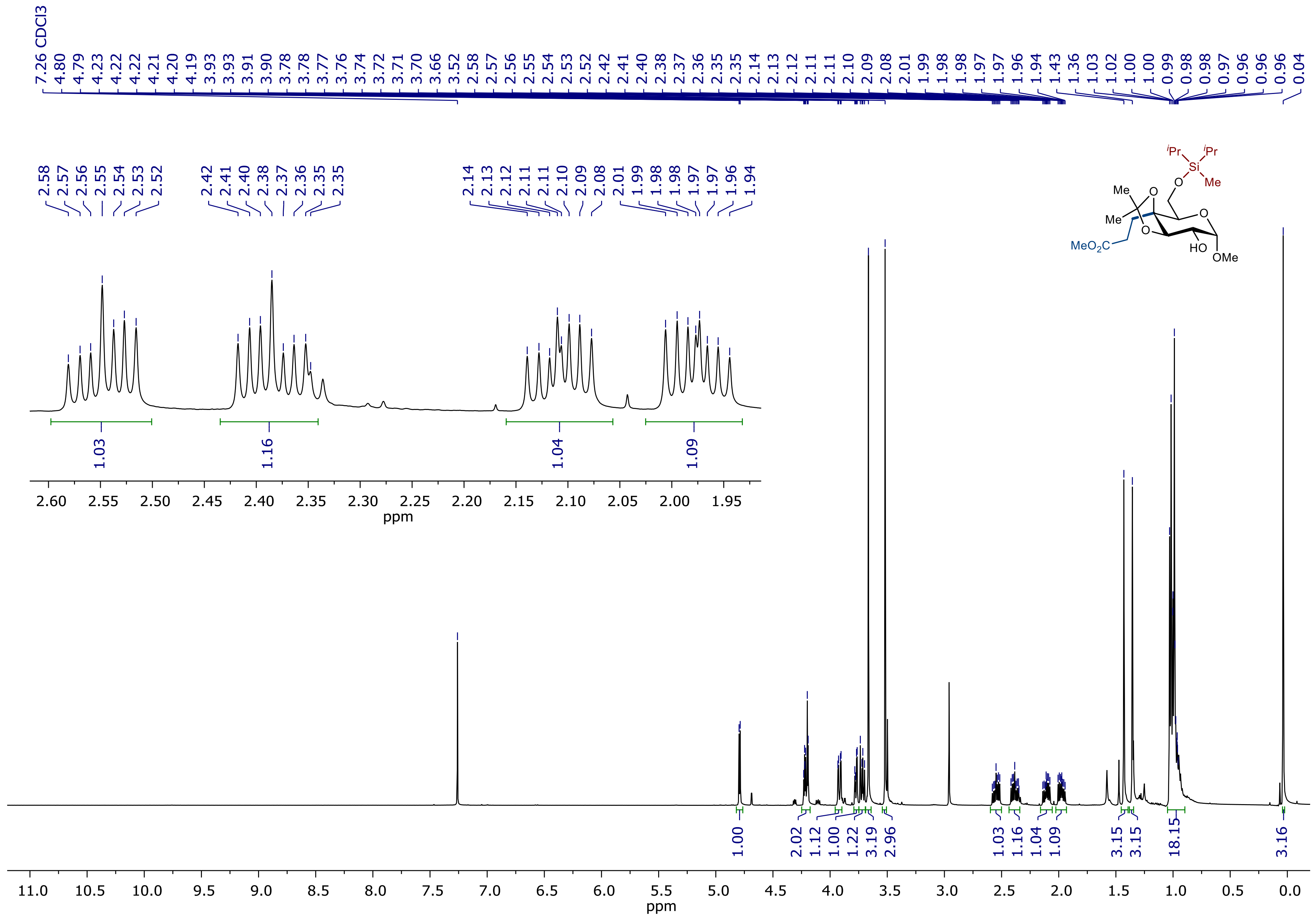

^{13}**C{^{1}H} NMR** (126 MHz, $CDCl_3$) of compound **3d**

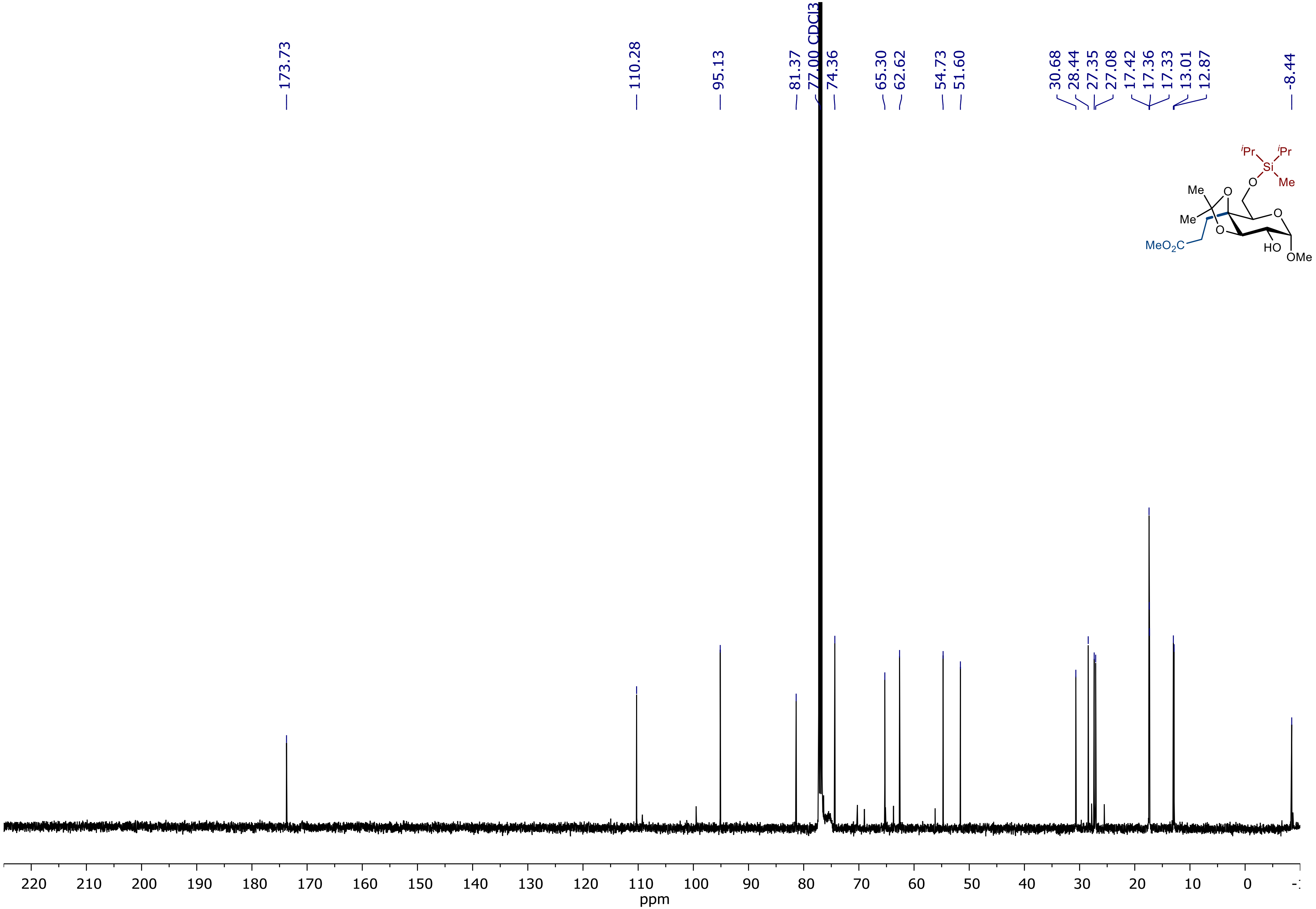

COSY of compound **3d**

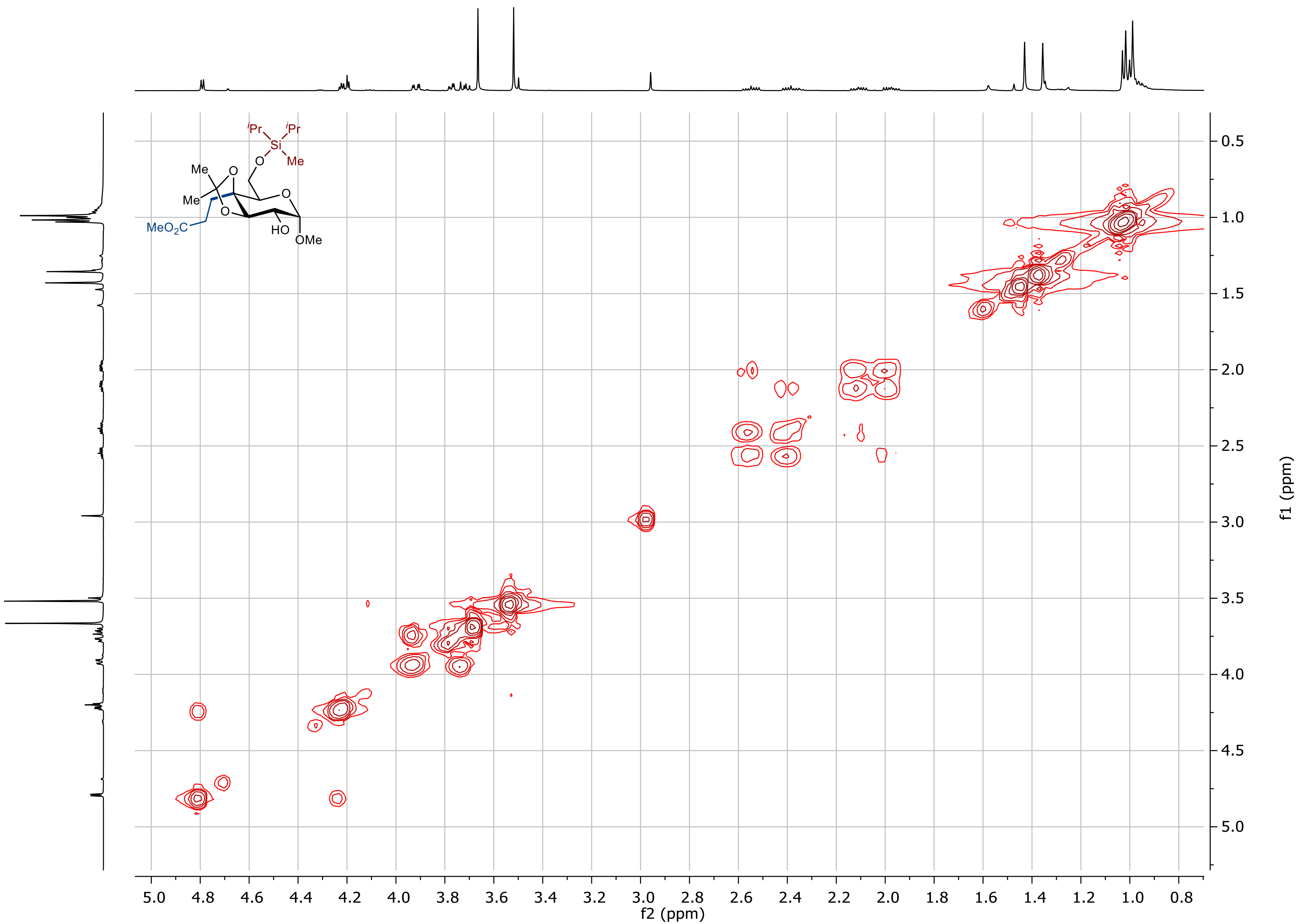

HSQC of compound **3d**

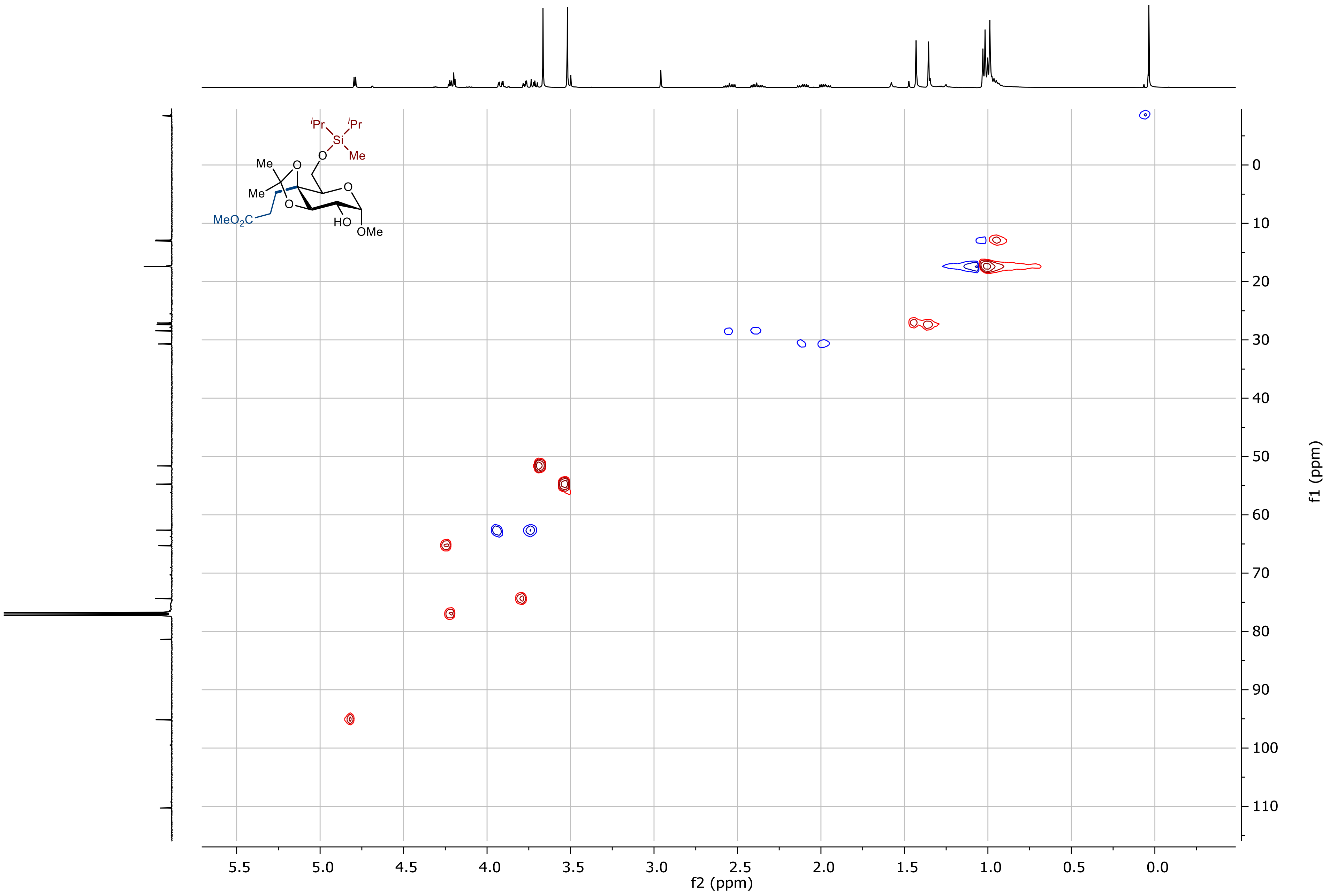

HMBC of compound **3d**

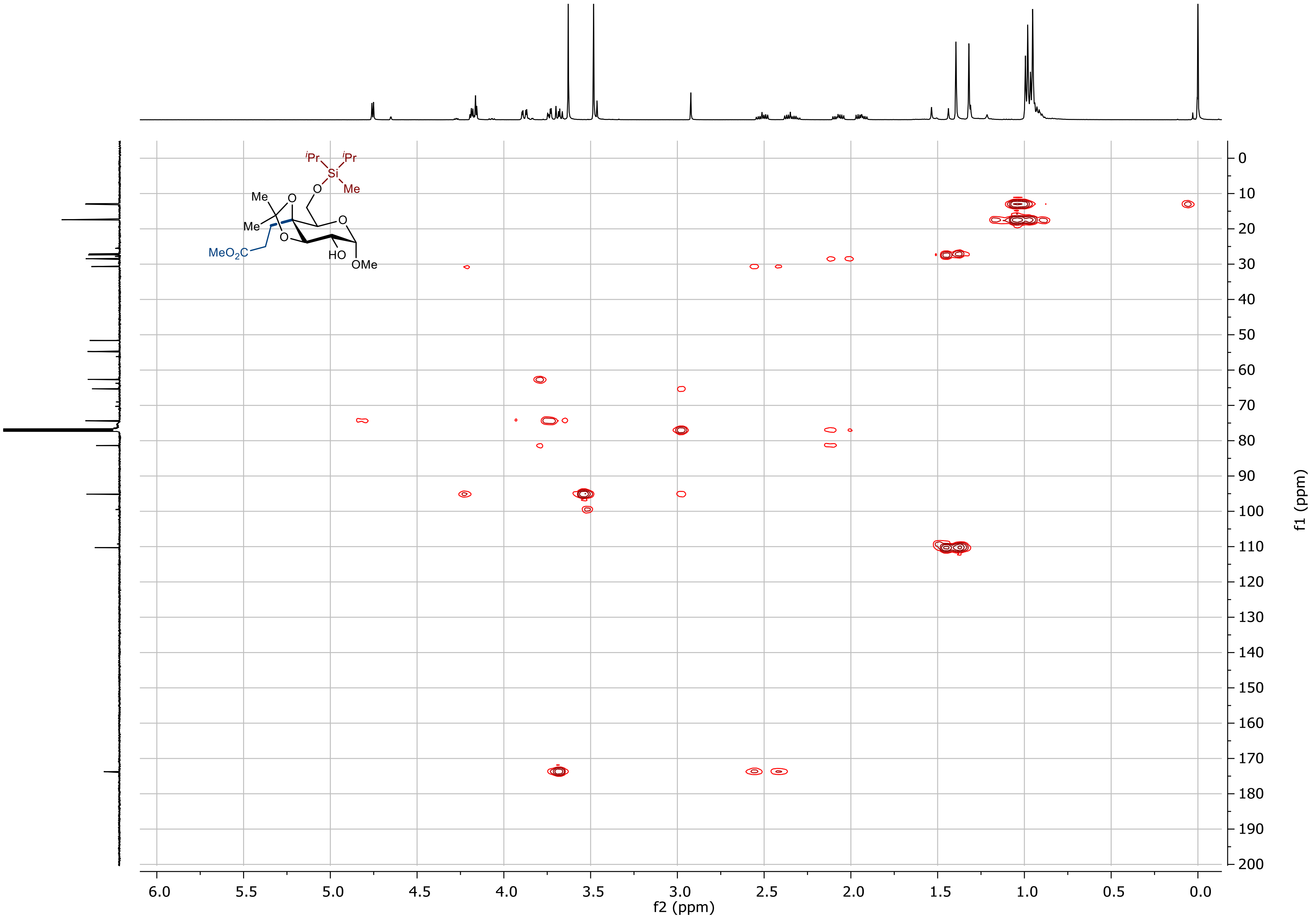

¹H NMR (500 MHz, $CDCl_3$) of compound **3e**

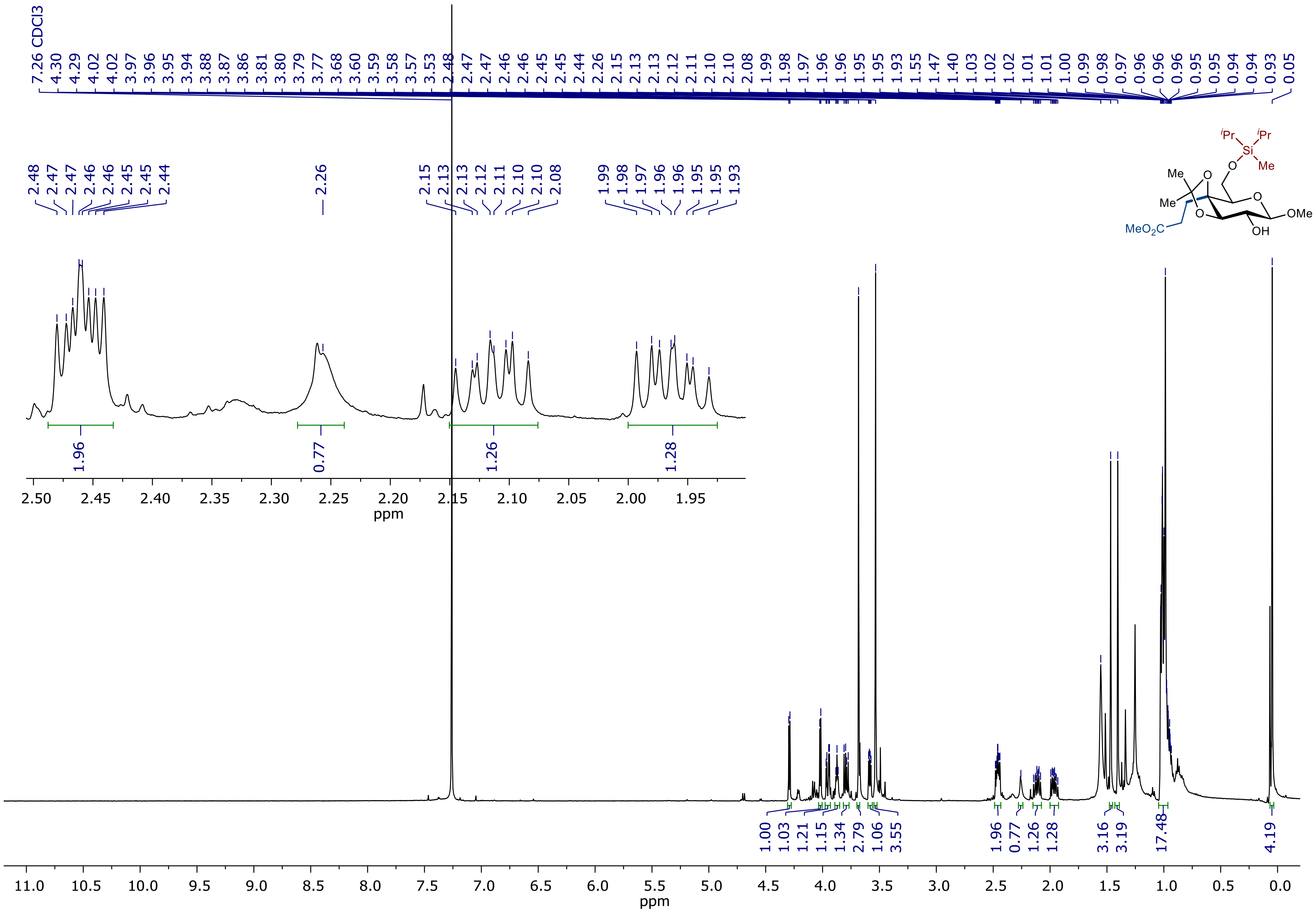

$^{13}C\{^1H\}$ NMR (126 MHz, $CDCl_3$) of compound **3e**

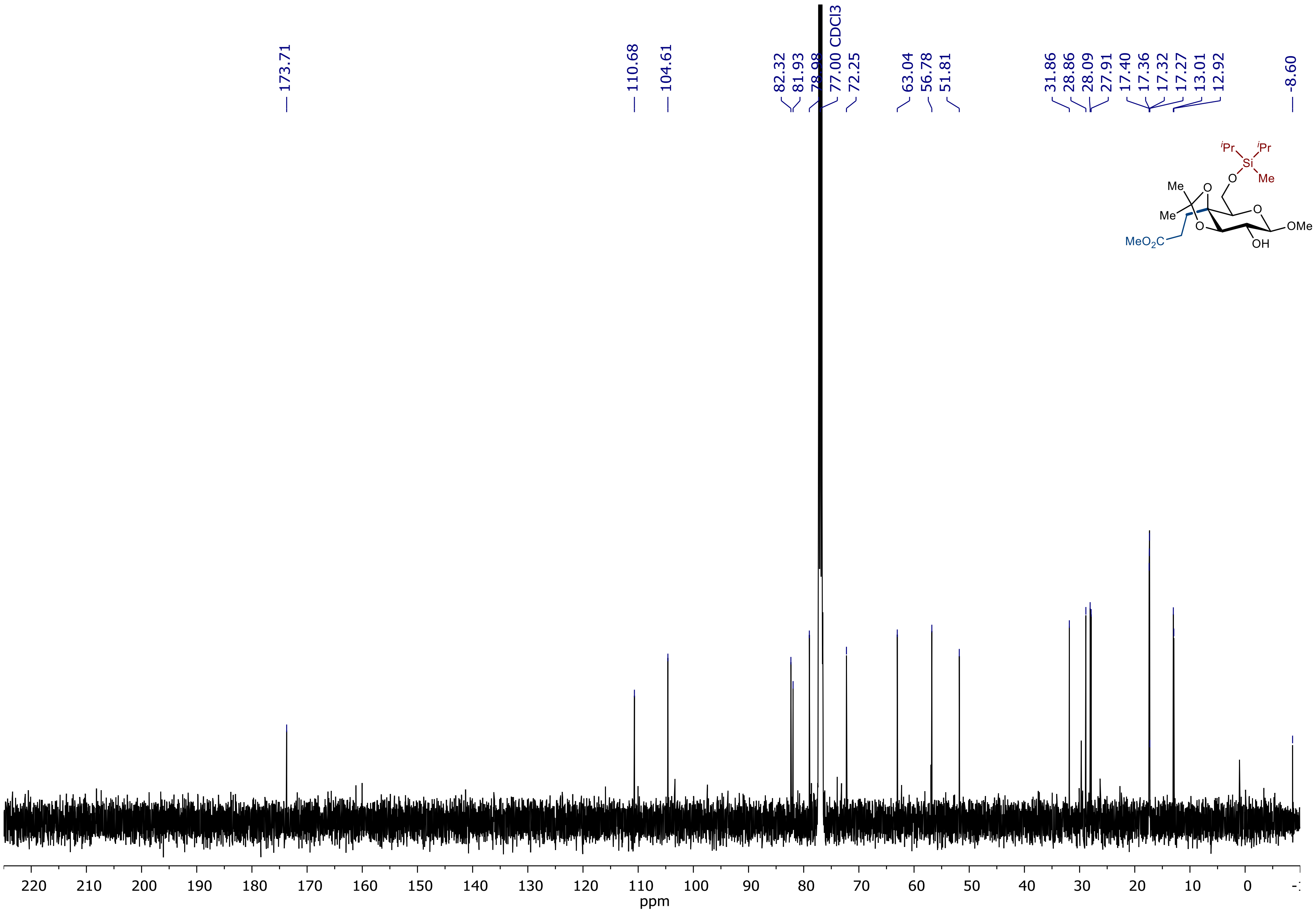

COSY of compound **3e**

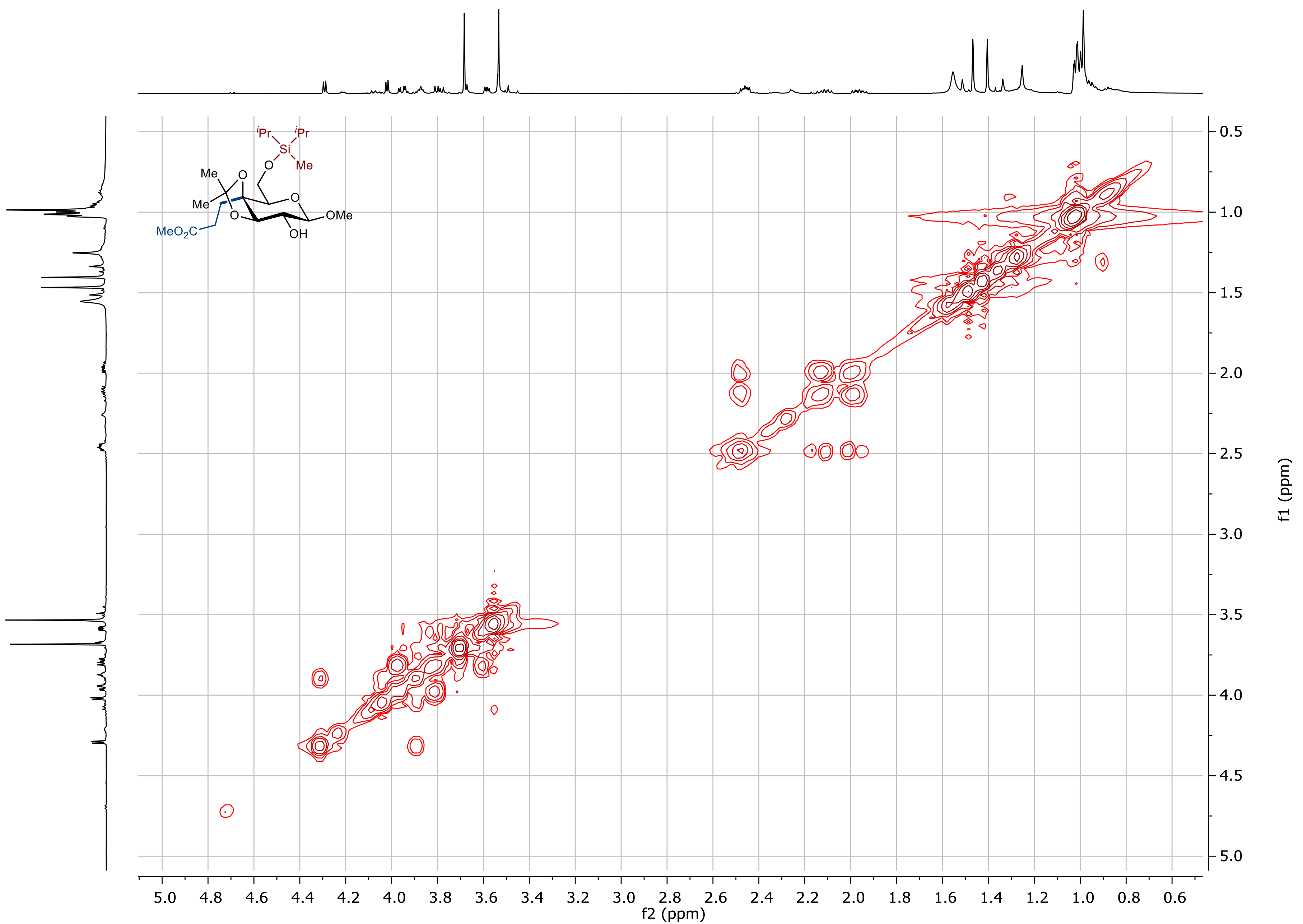

HSQC of compound **3e**

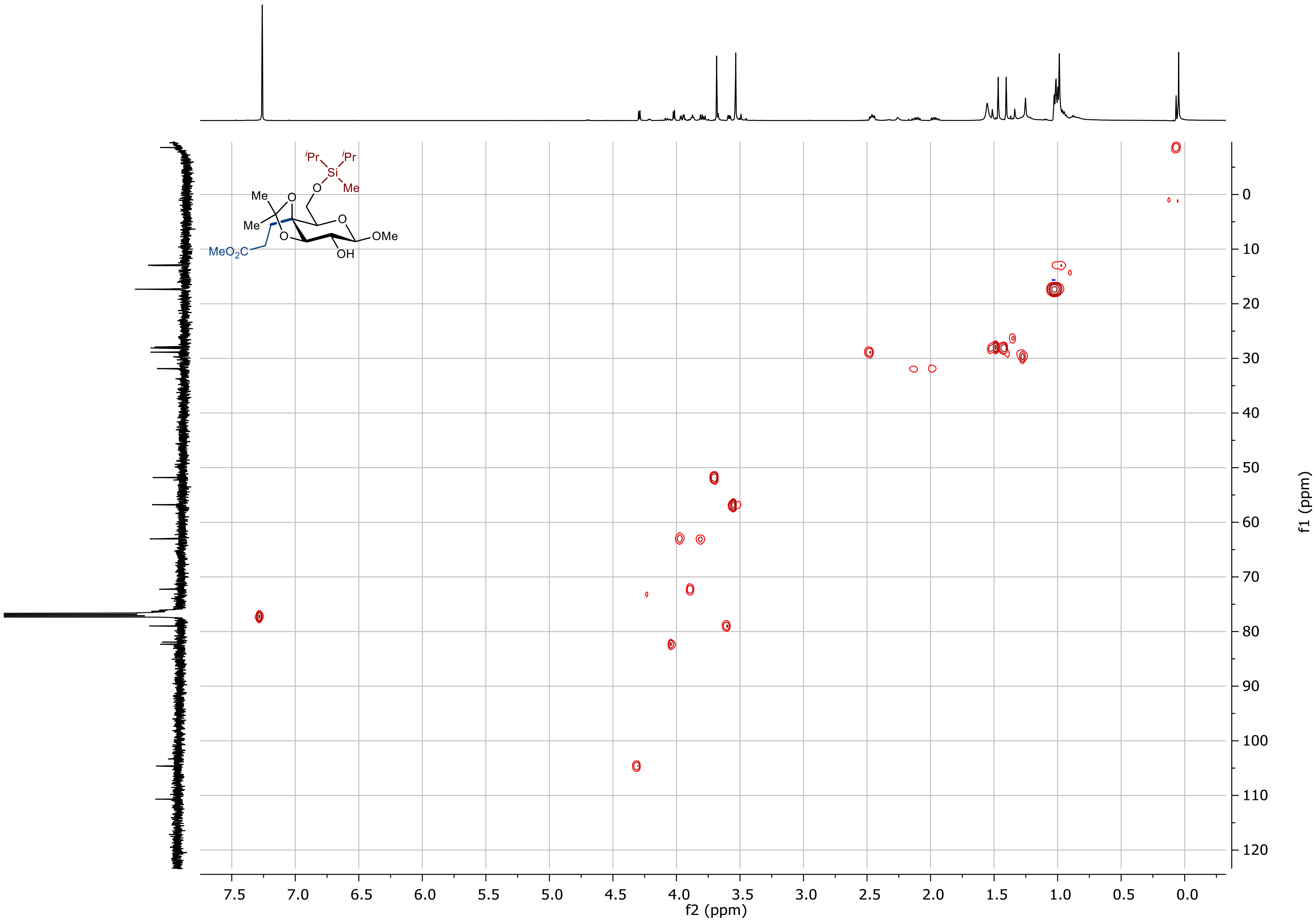

NOESY of compound **3e**

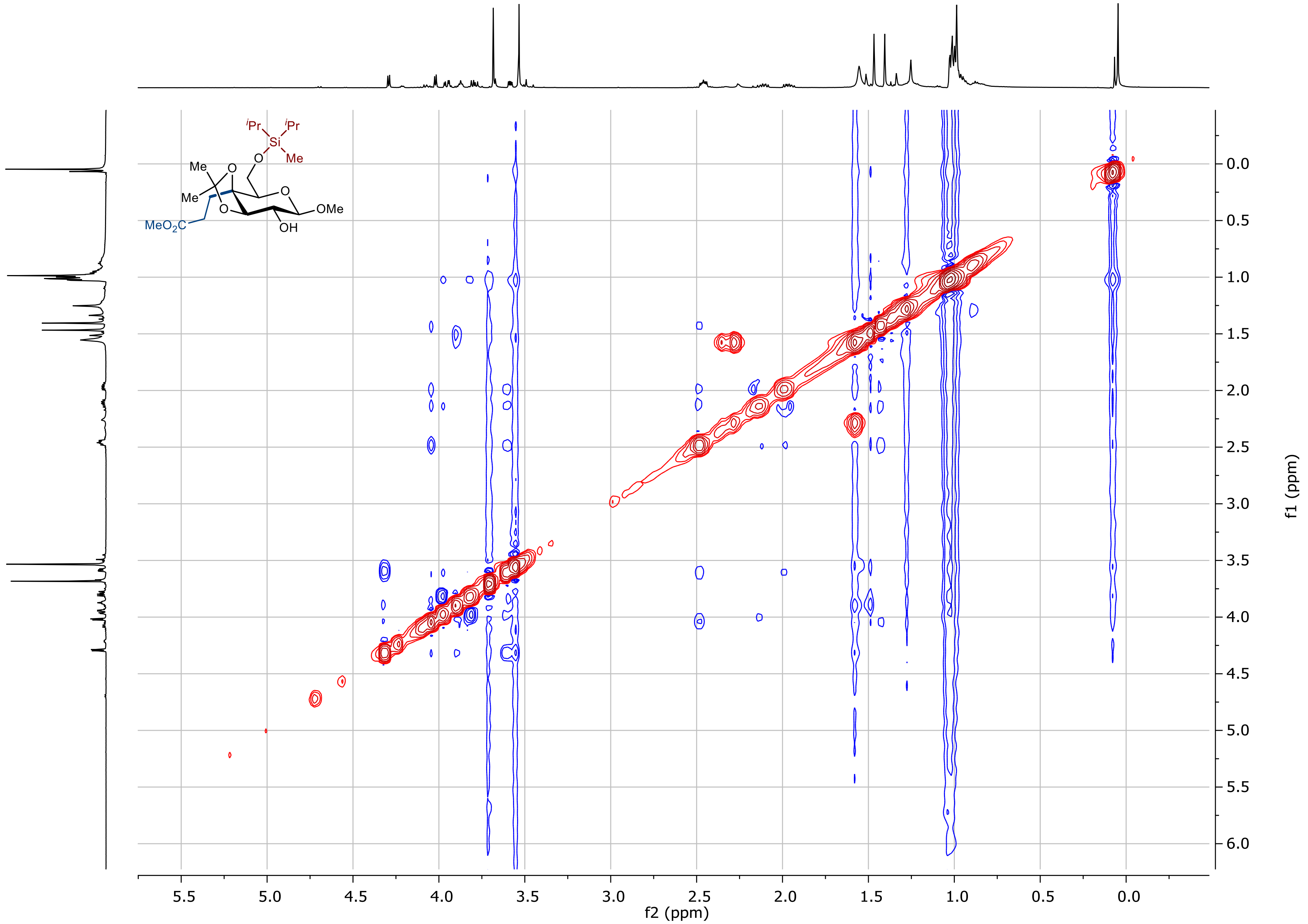

^{1}H NMR (500 MHz, $CDCl_3$) of compound **3e'**

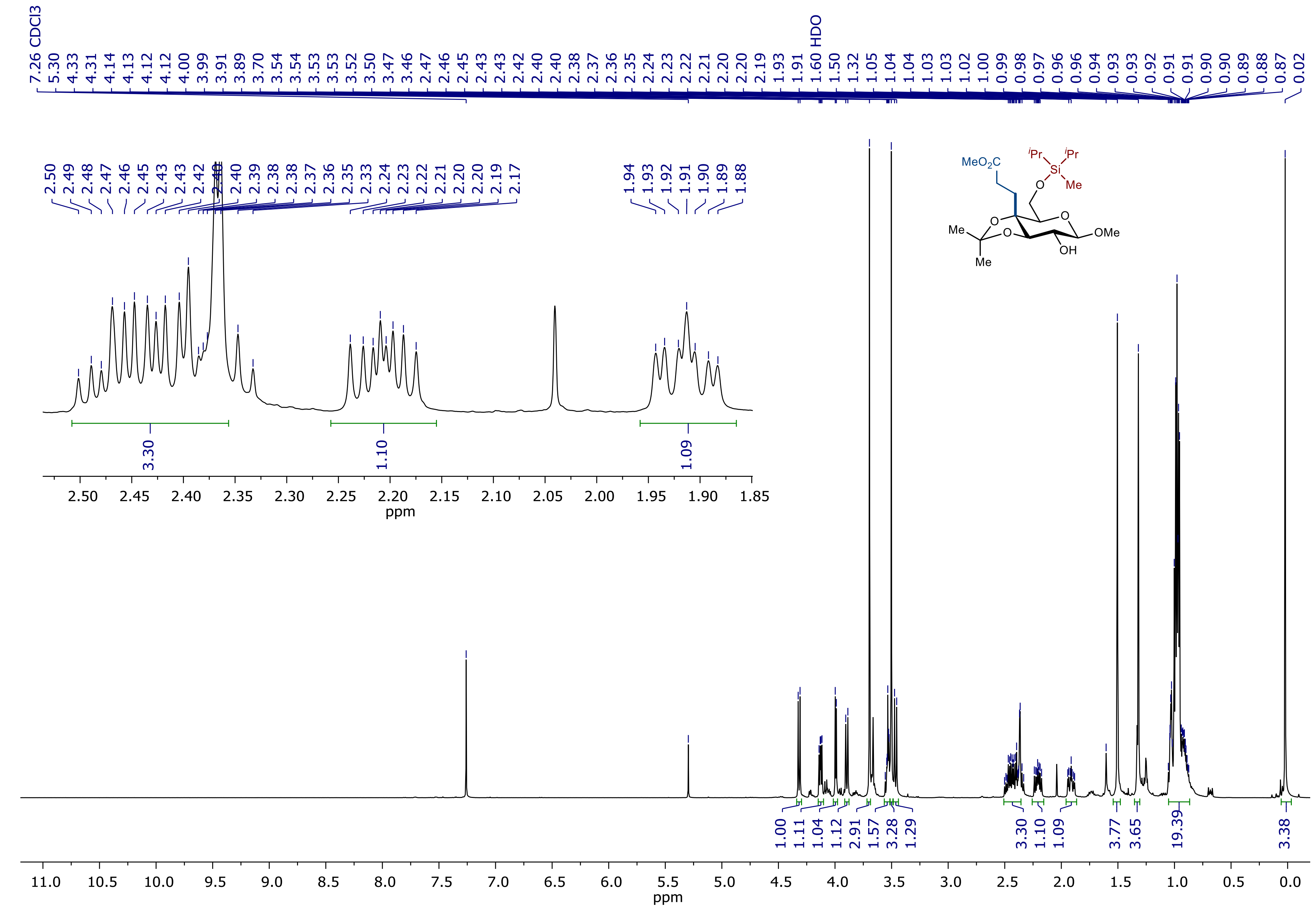

$^{13}C\{^{1}H\}$ NMR (126 MHz, $CDCl_3$) of compound **3e'**

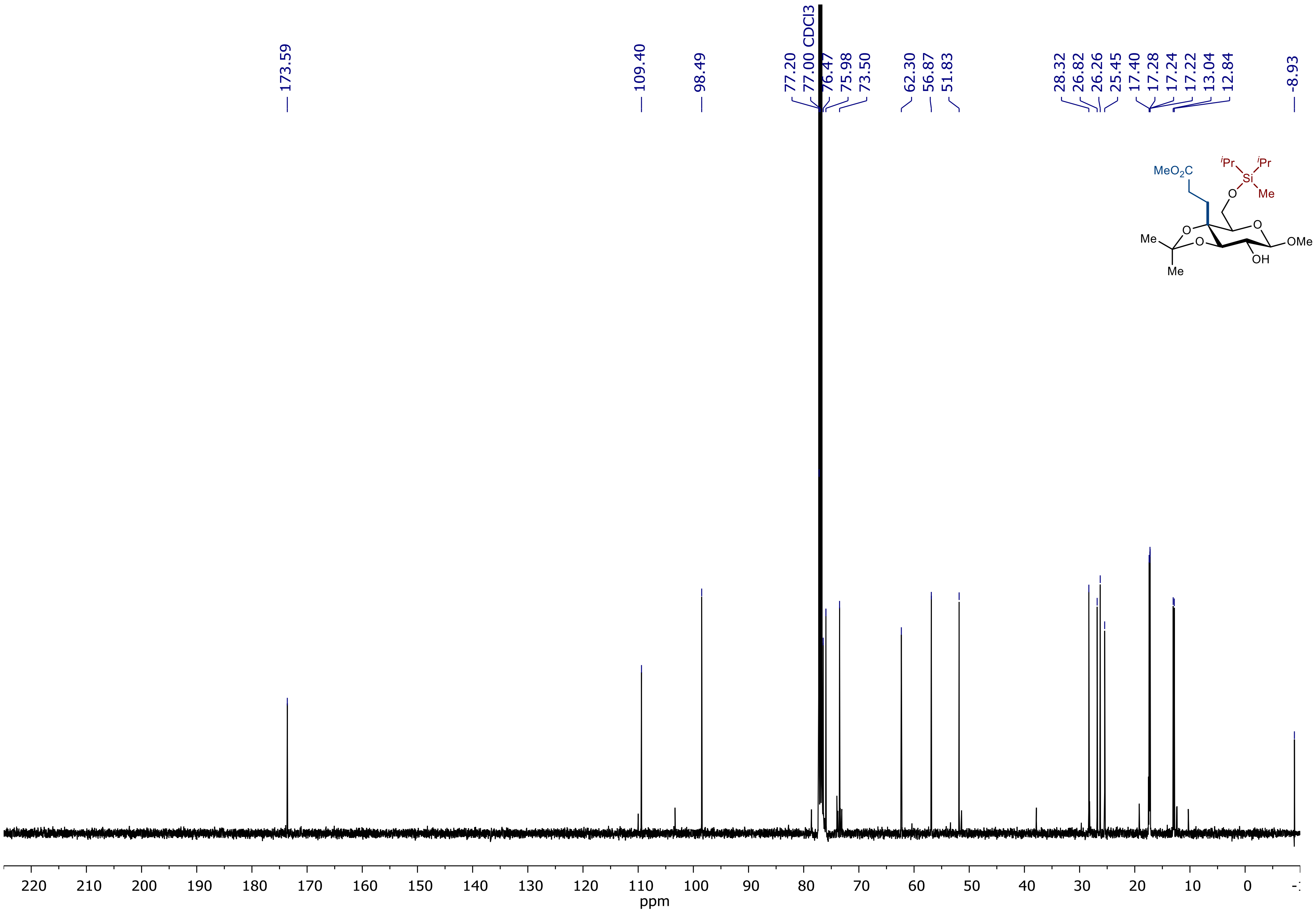

COSY of compound **3e’**

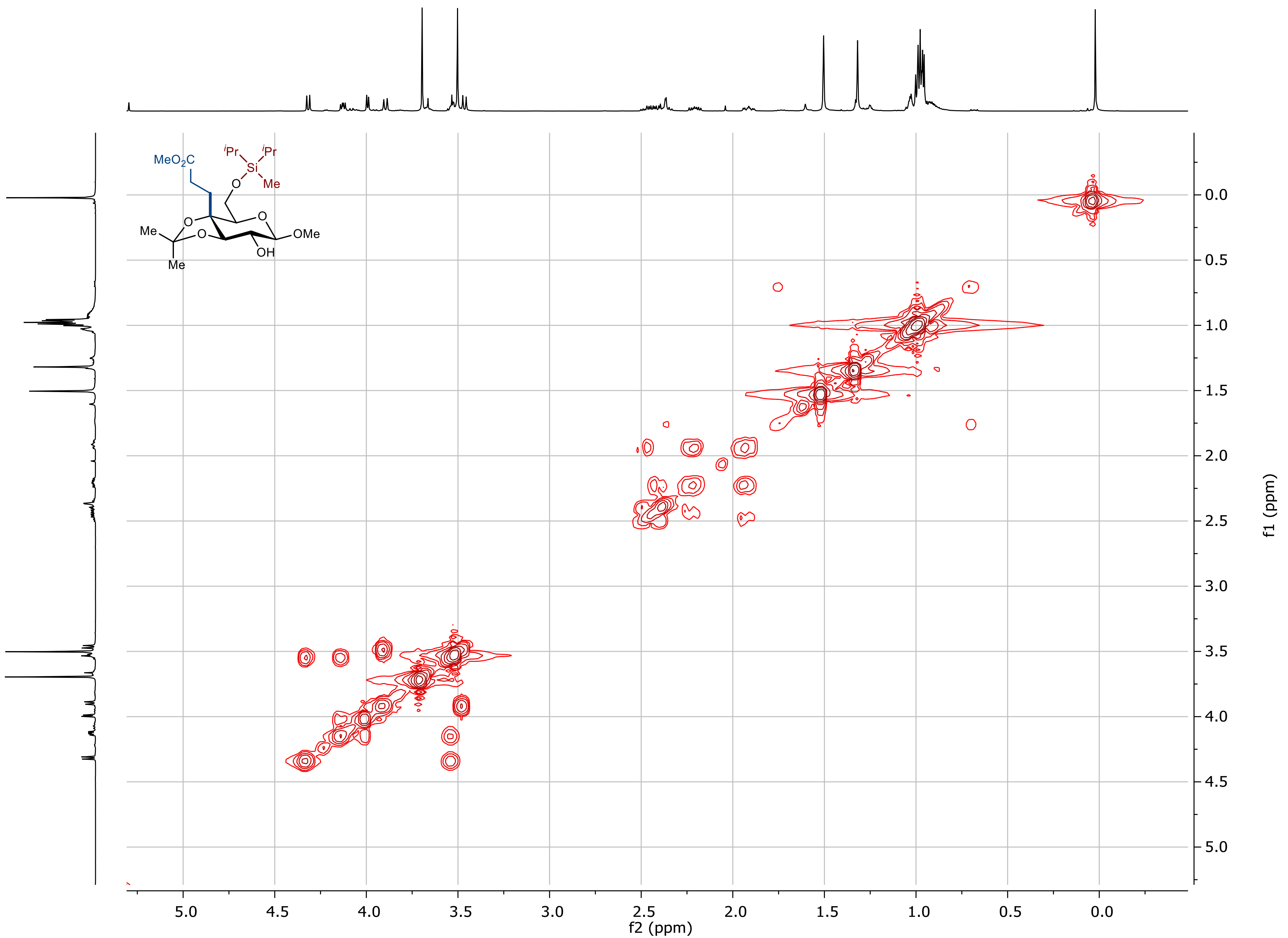

HSQC of compound **3e'**

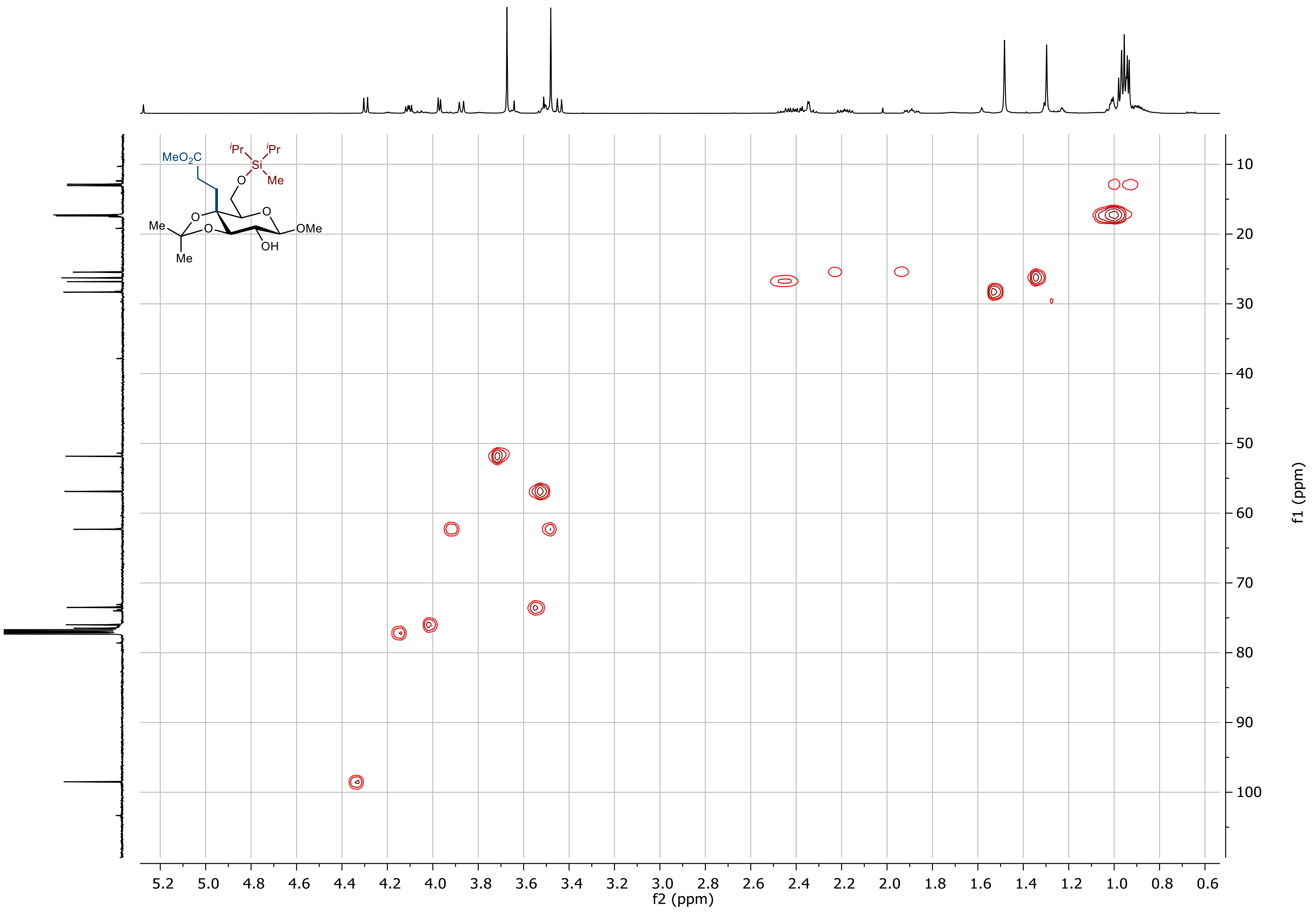

HMBC of compound **3e'**

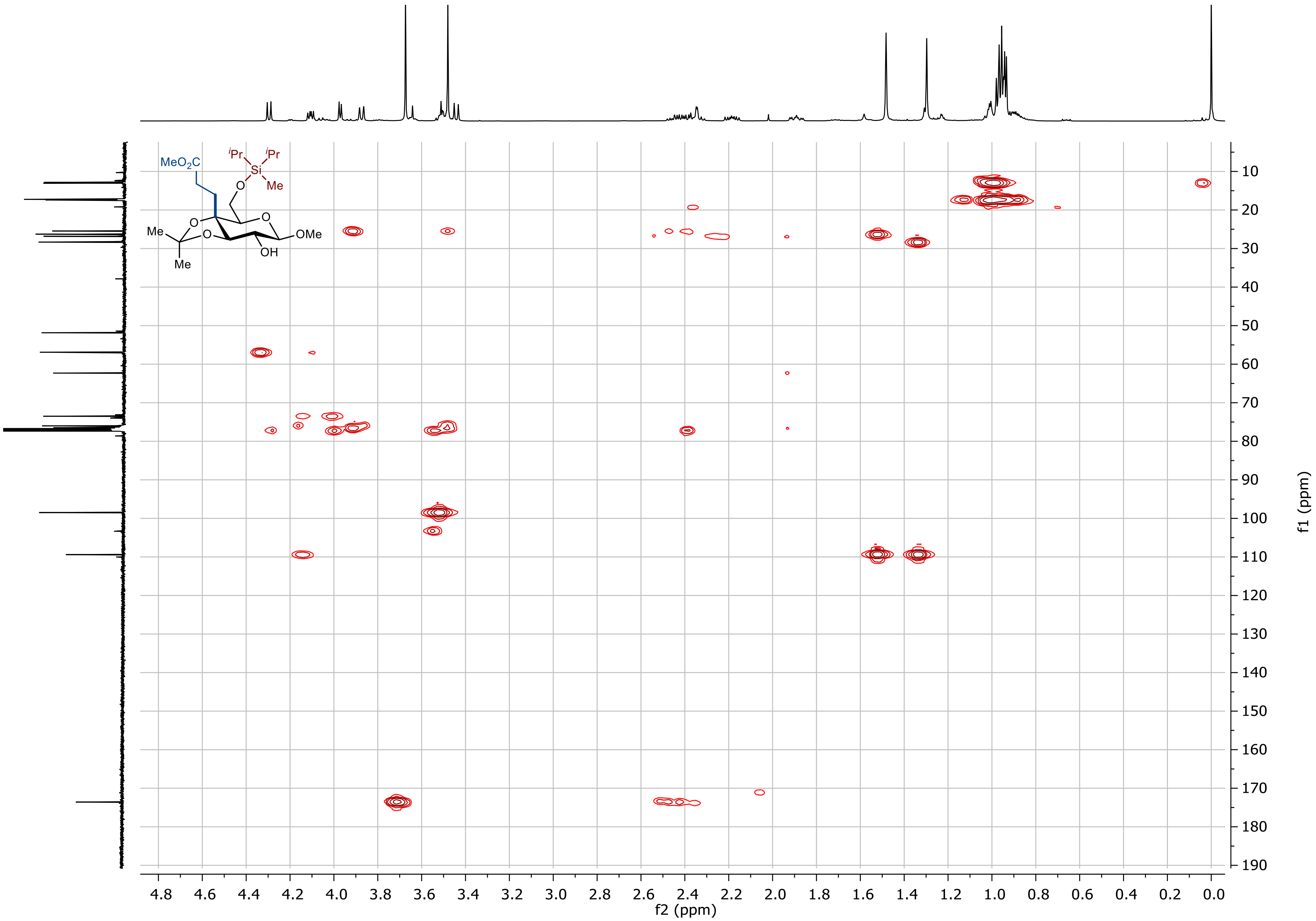

^{1}H NMR (500 MHz, $CDCl_3$) of compound **3f**

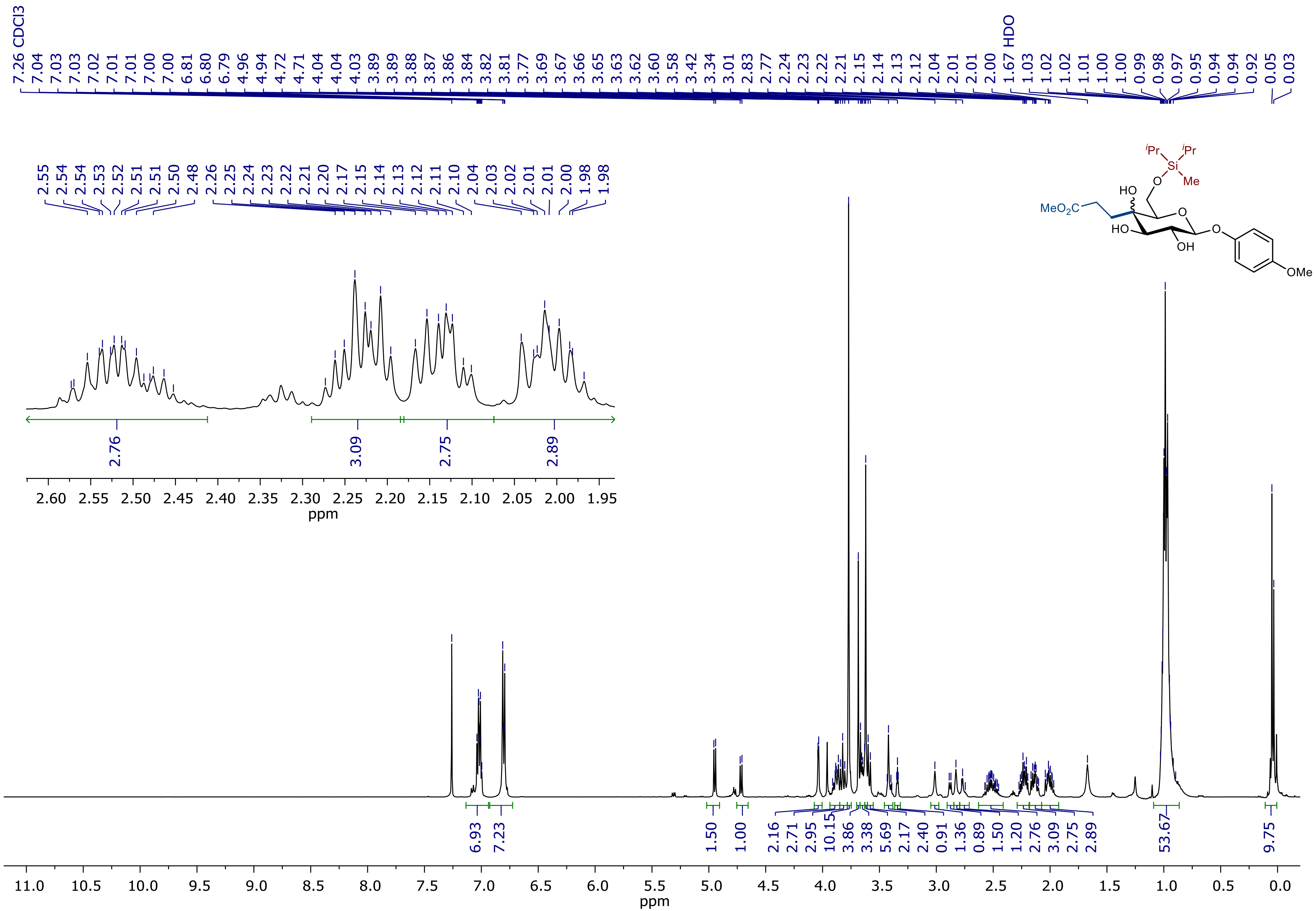

$^{13}C\{^1H\}$ NMR (126 MHz, $CDCl_3$) of compound **3f**

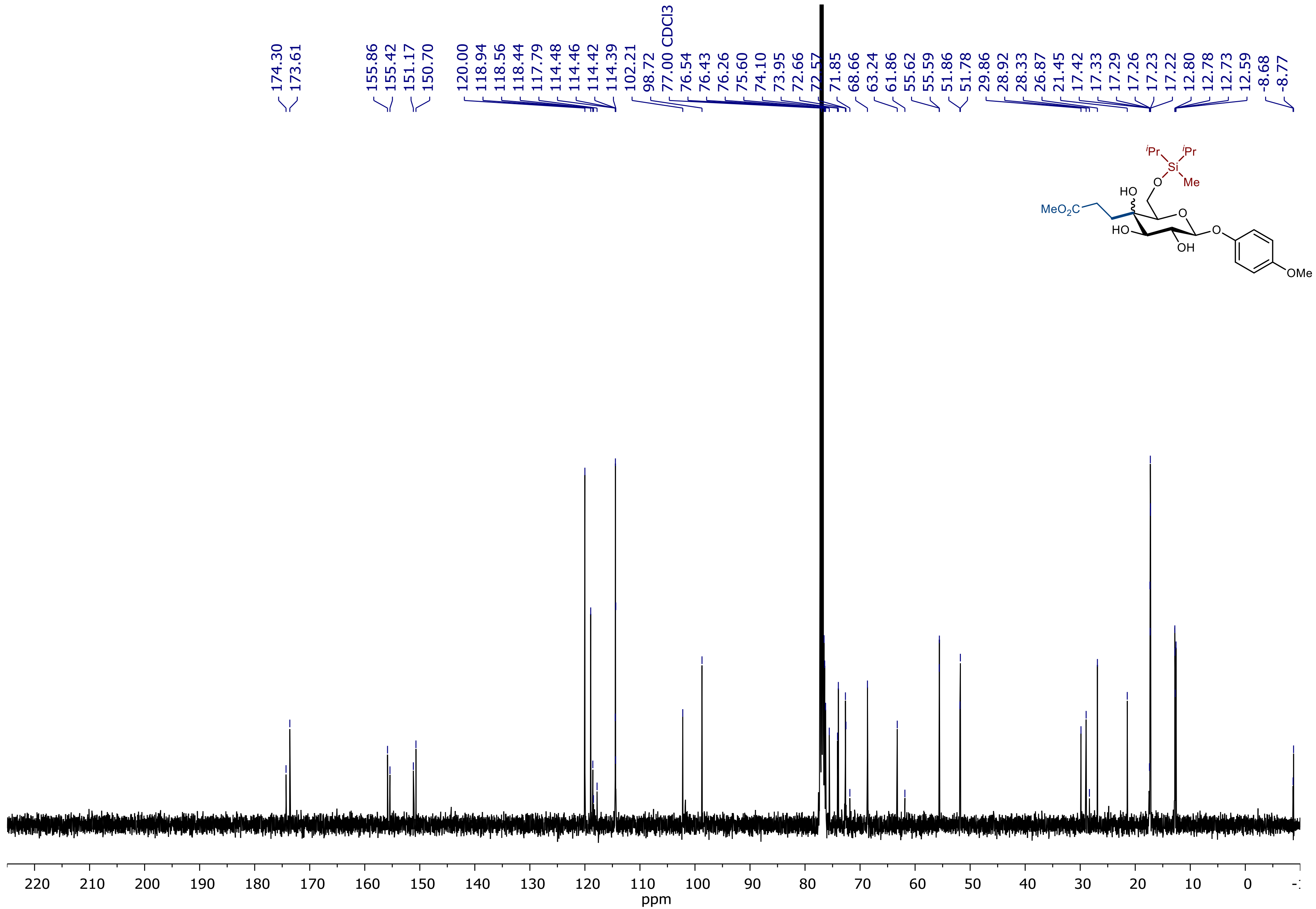

COSY of compound **3f**

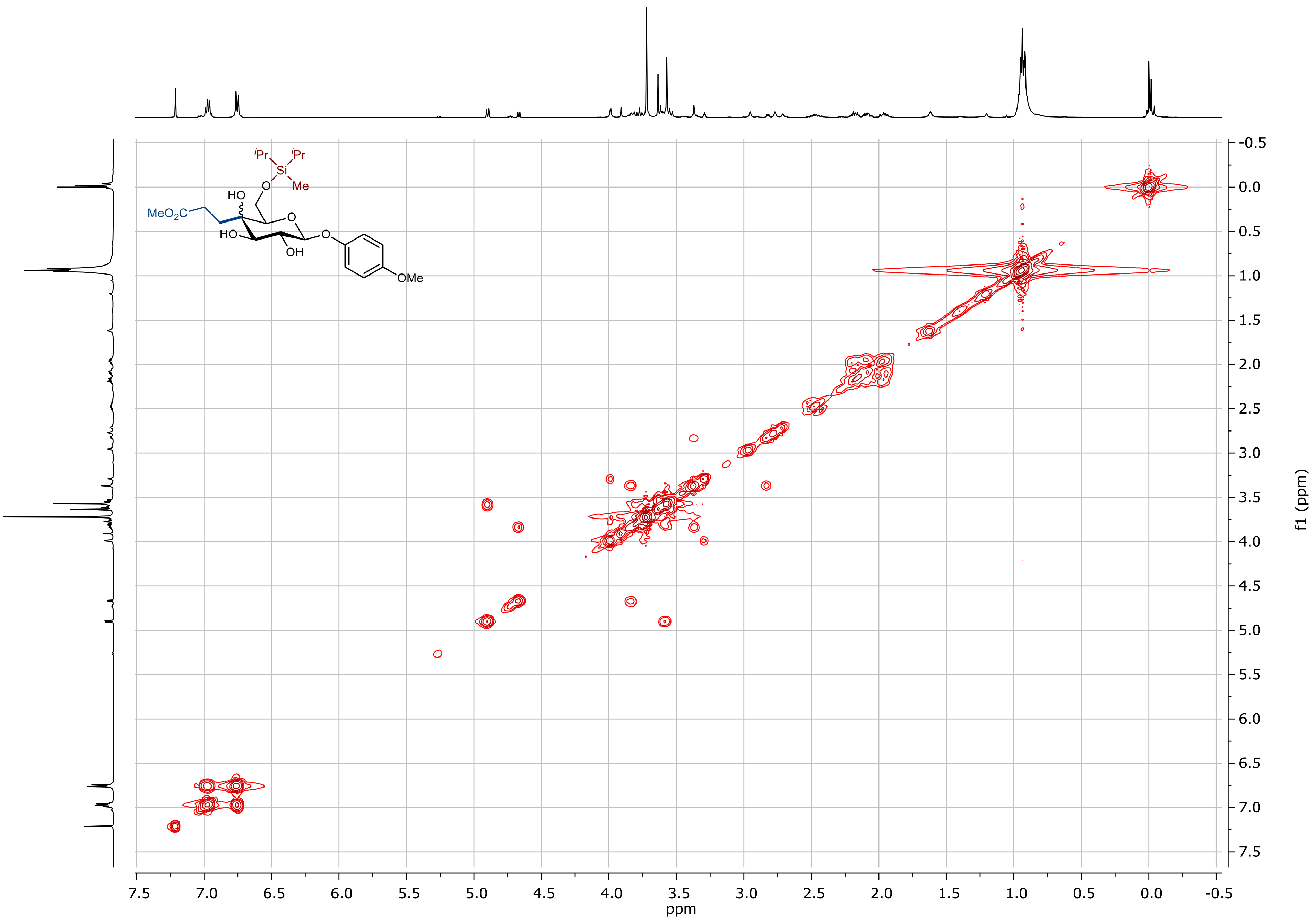

HSQC of compound **3f**

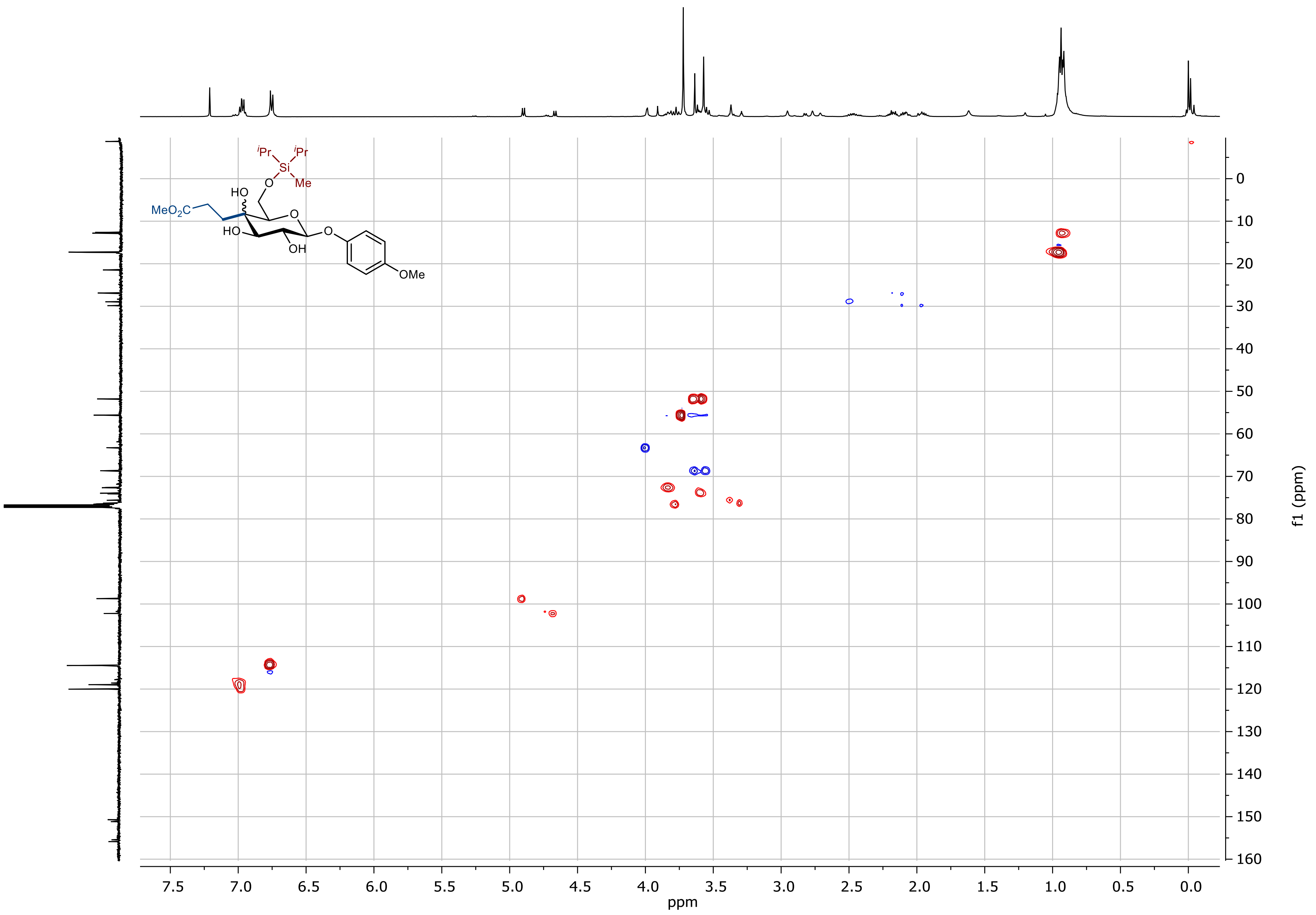

^{1}H NMR (500 MHz, $CDCl_3$) of compound **3g**

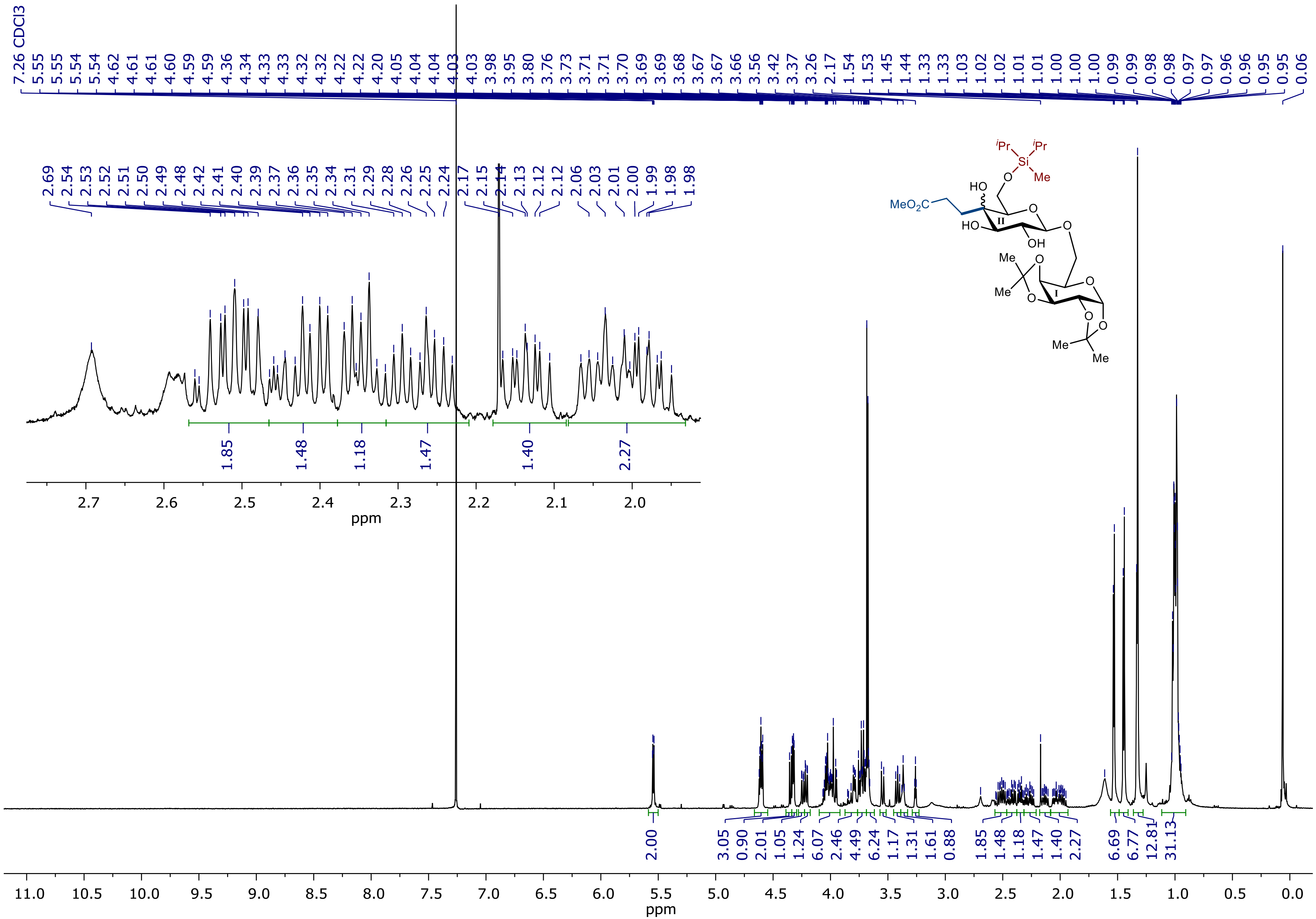

$^{13}C\{^{1}H\}$ **NMR** (126 MHz, $CDCl_3$) of compound **3g**

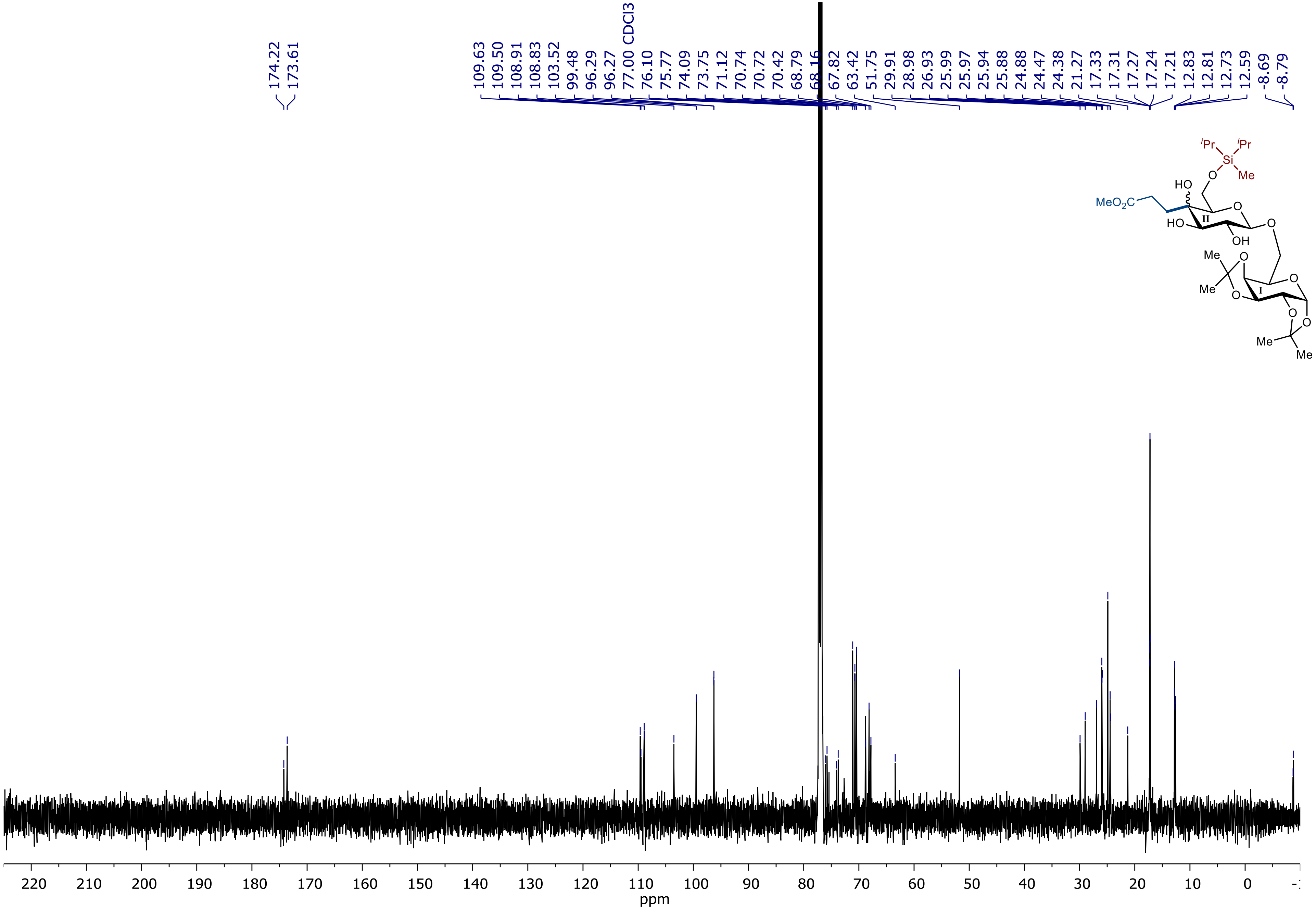

COSY of compound **3g**

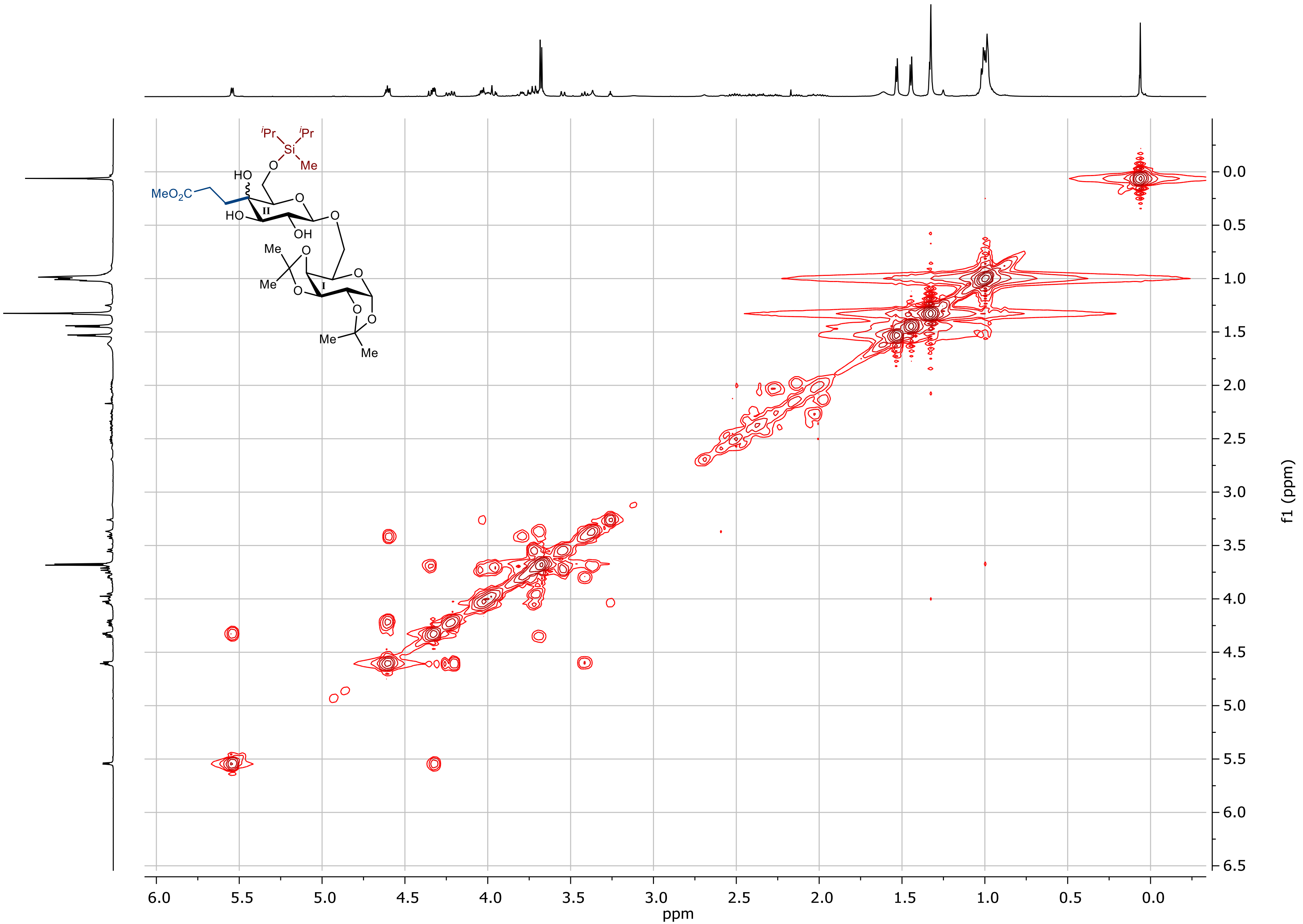

HSQC of compound **3g**

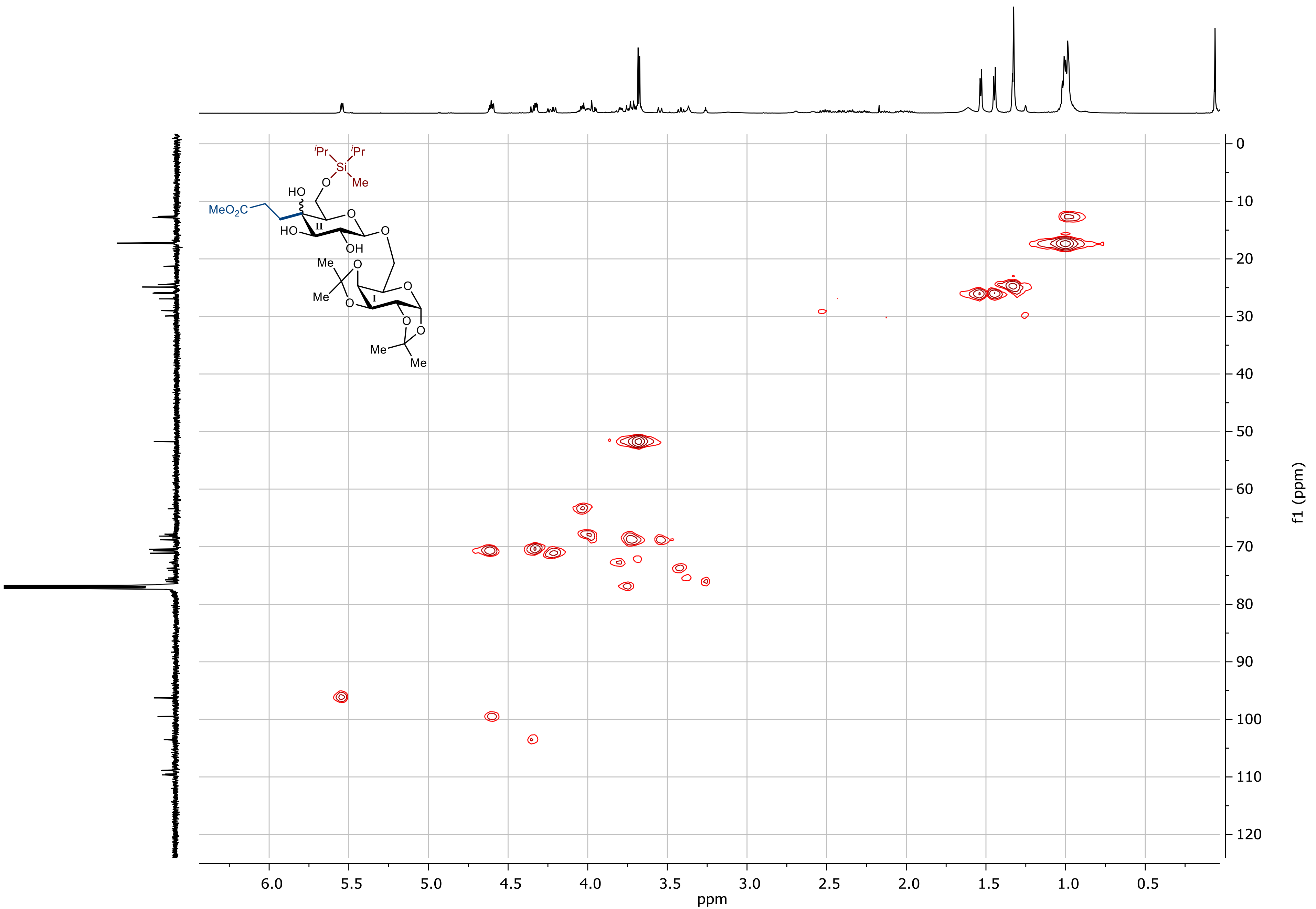

^{1}H NMR (500 MHz, acetone-d_6) of compound 3h

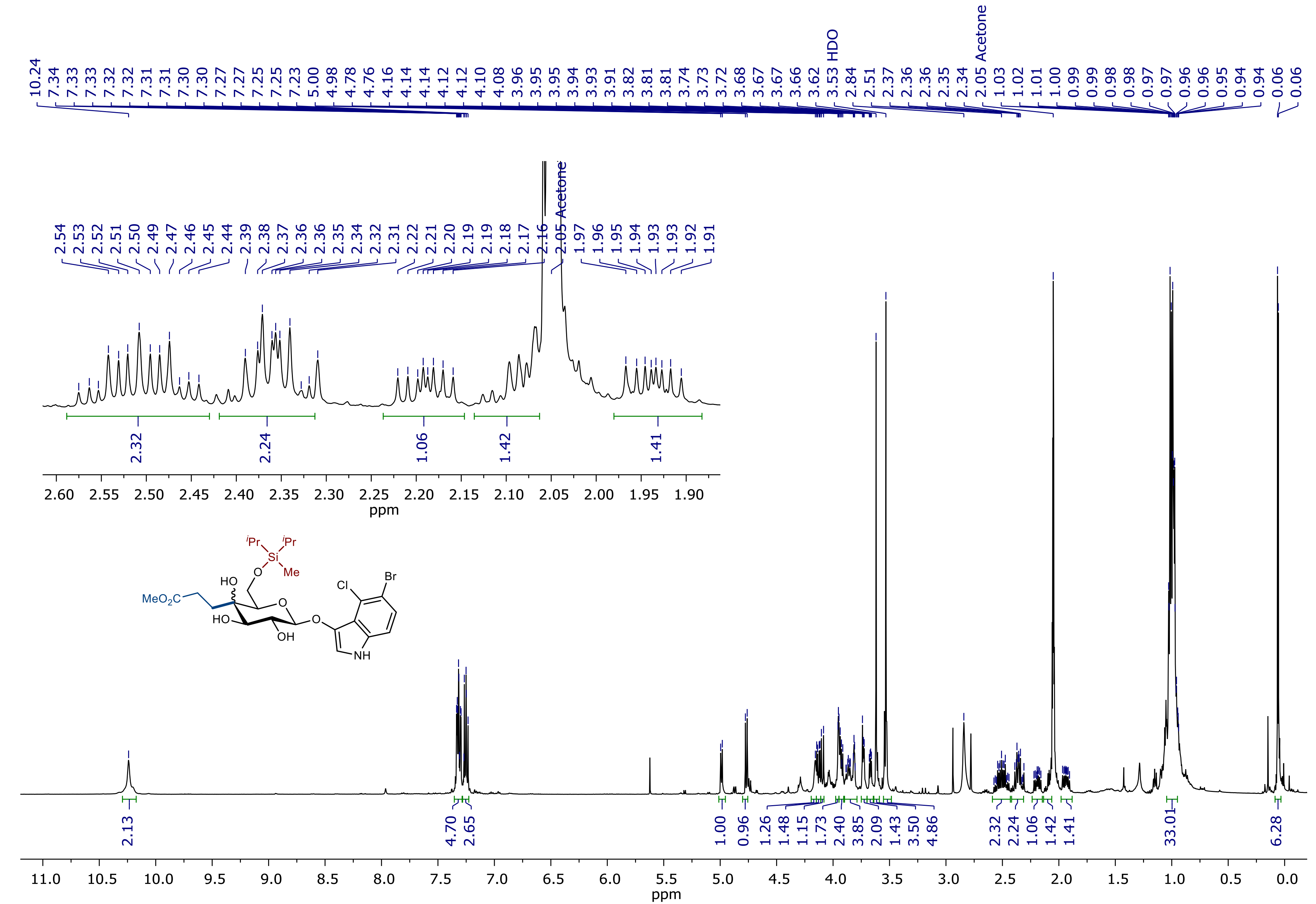

$^{13}C\{^1H\}$ NMR (126 MHz, acetone-d_6) of compound **3h**

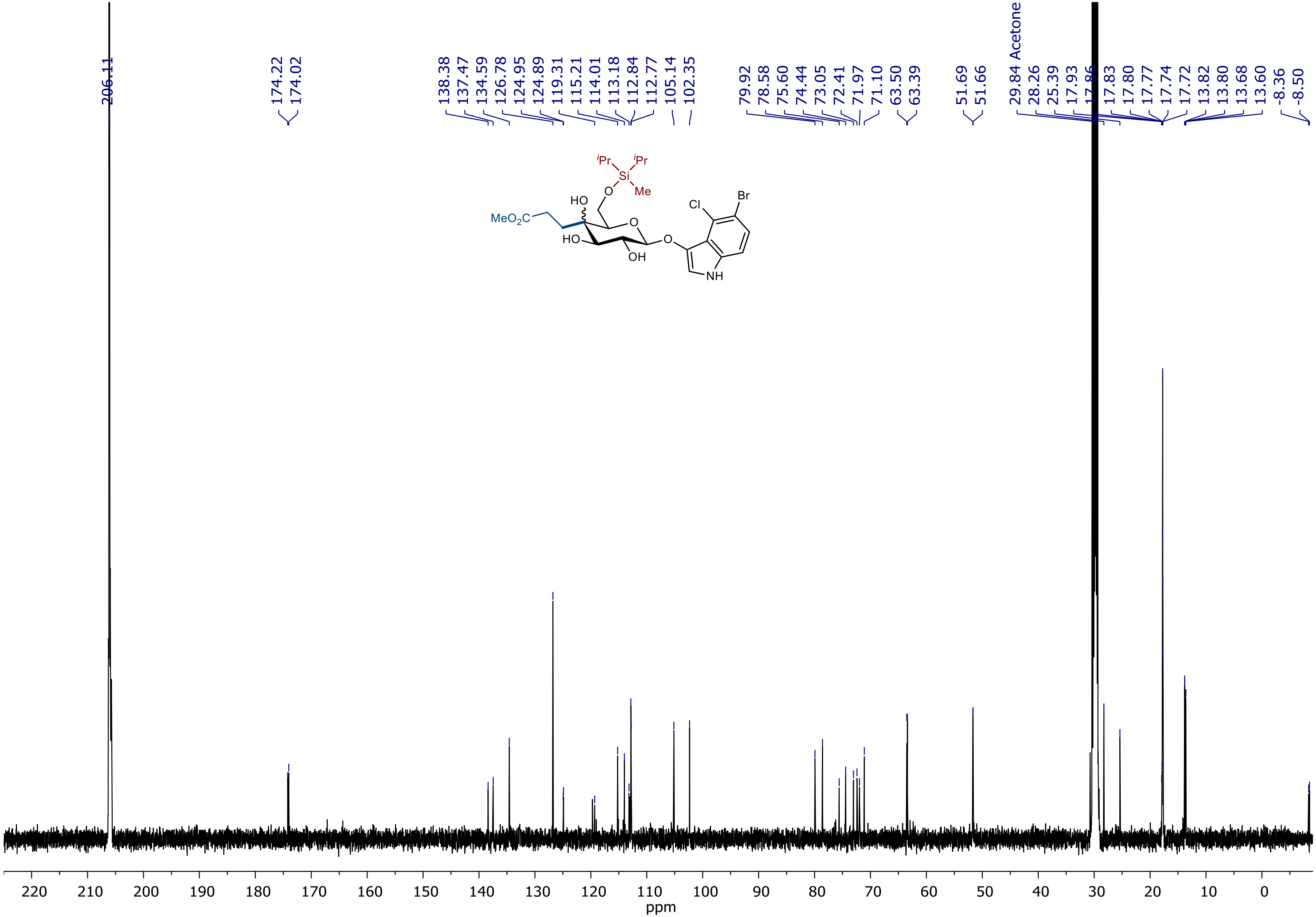

COSY of compound **3h**

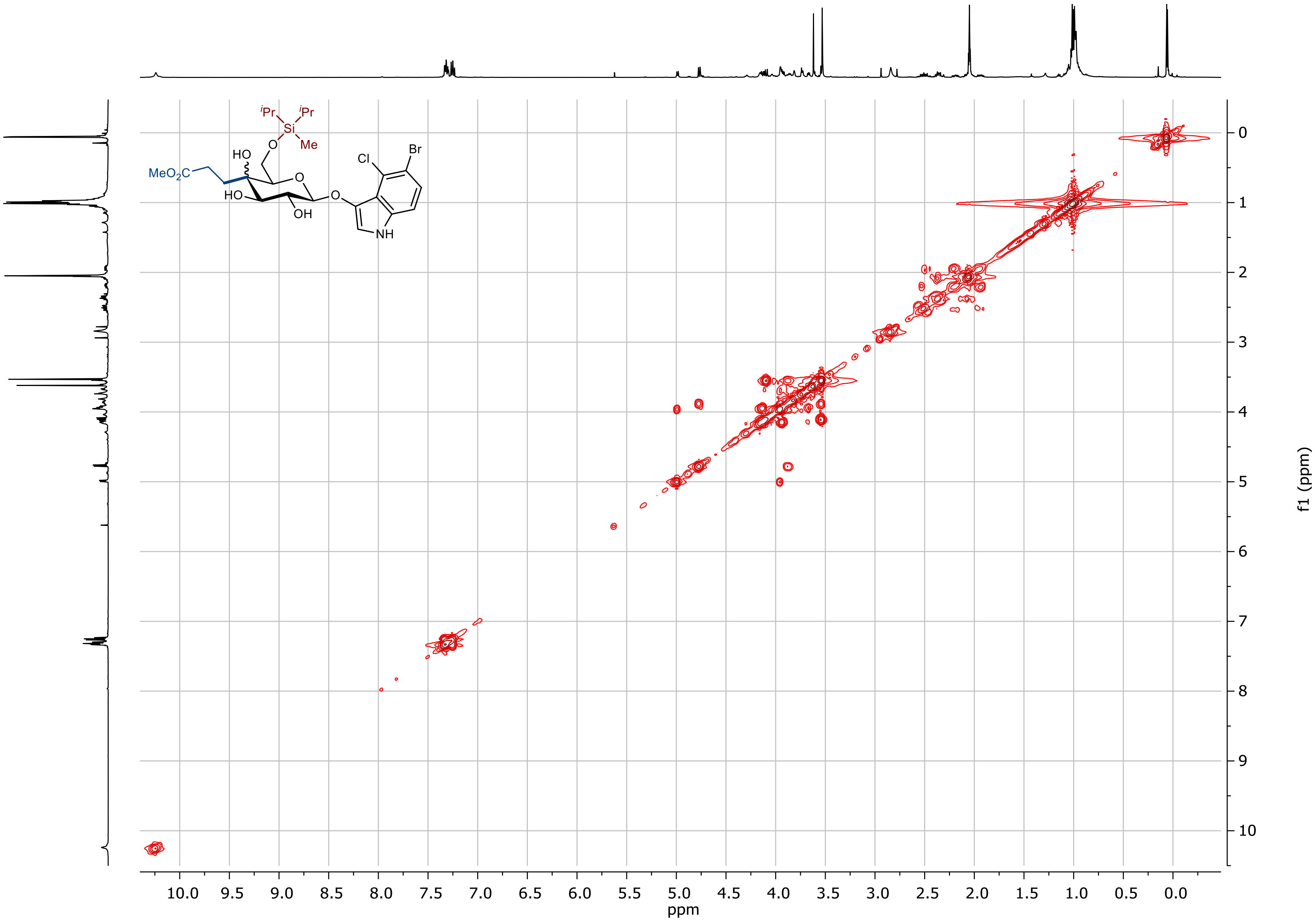

HSQC of compound **3h**

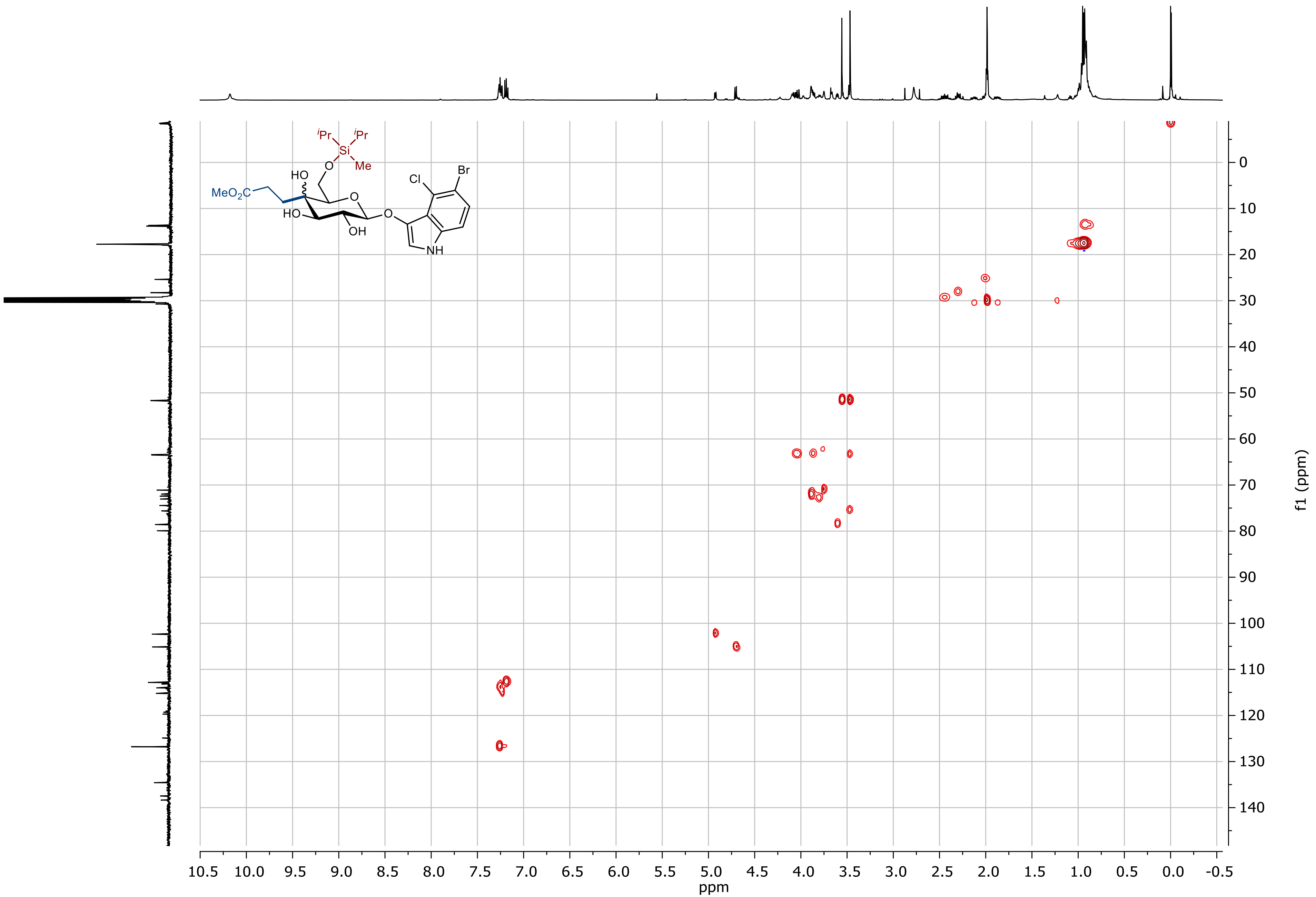

^{1}H NMR (500 MHz, $CDCl_3$) of compound **3i**

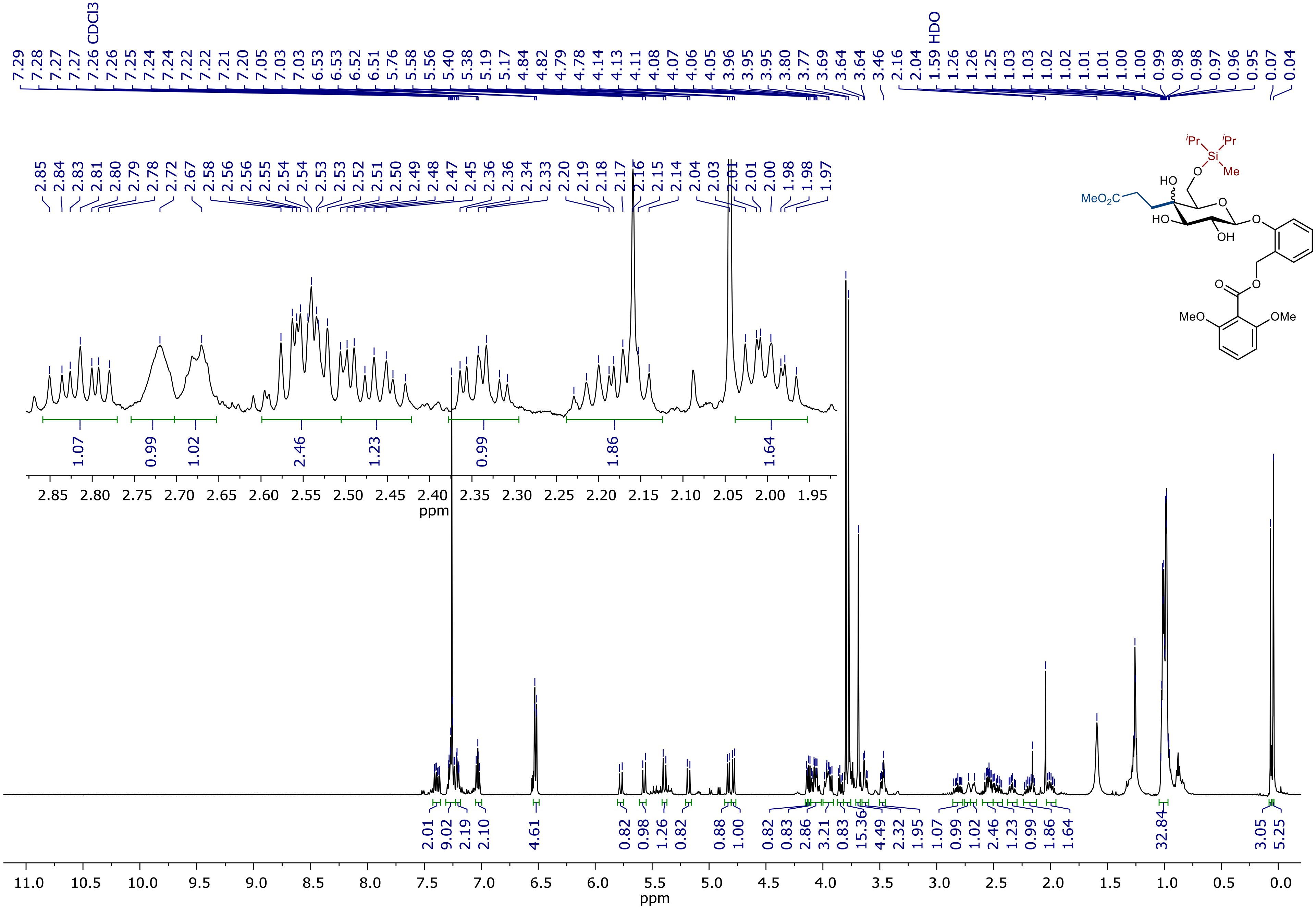

$^{13}C\{^1H\}$ **NMR** (126 MHz, $CDCl_3$) of compound **3i**

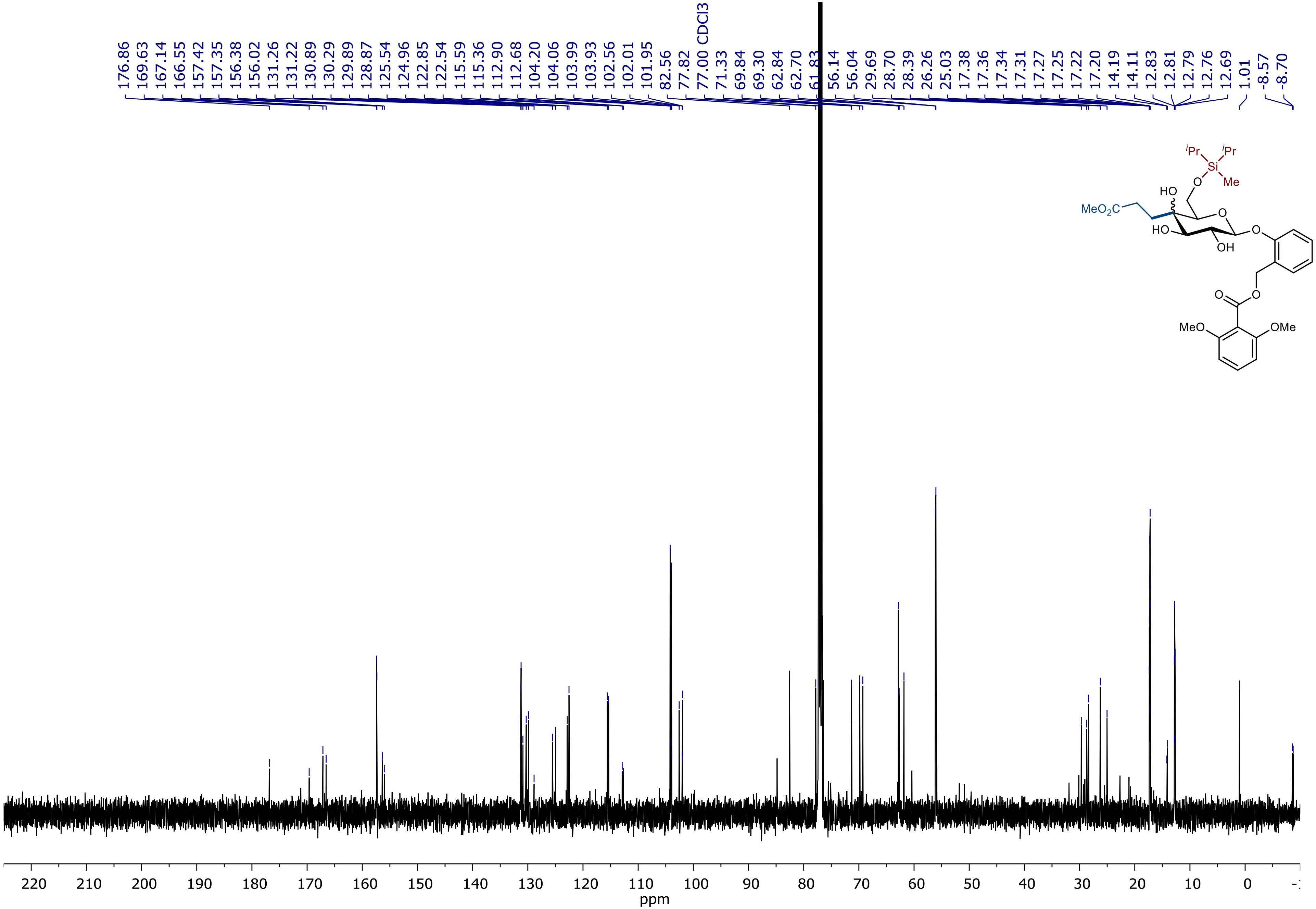

COSY of compound **3i**

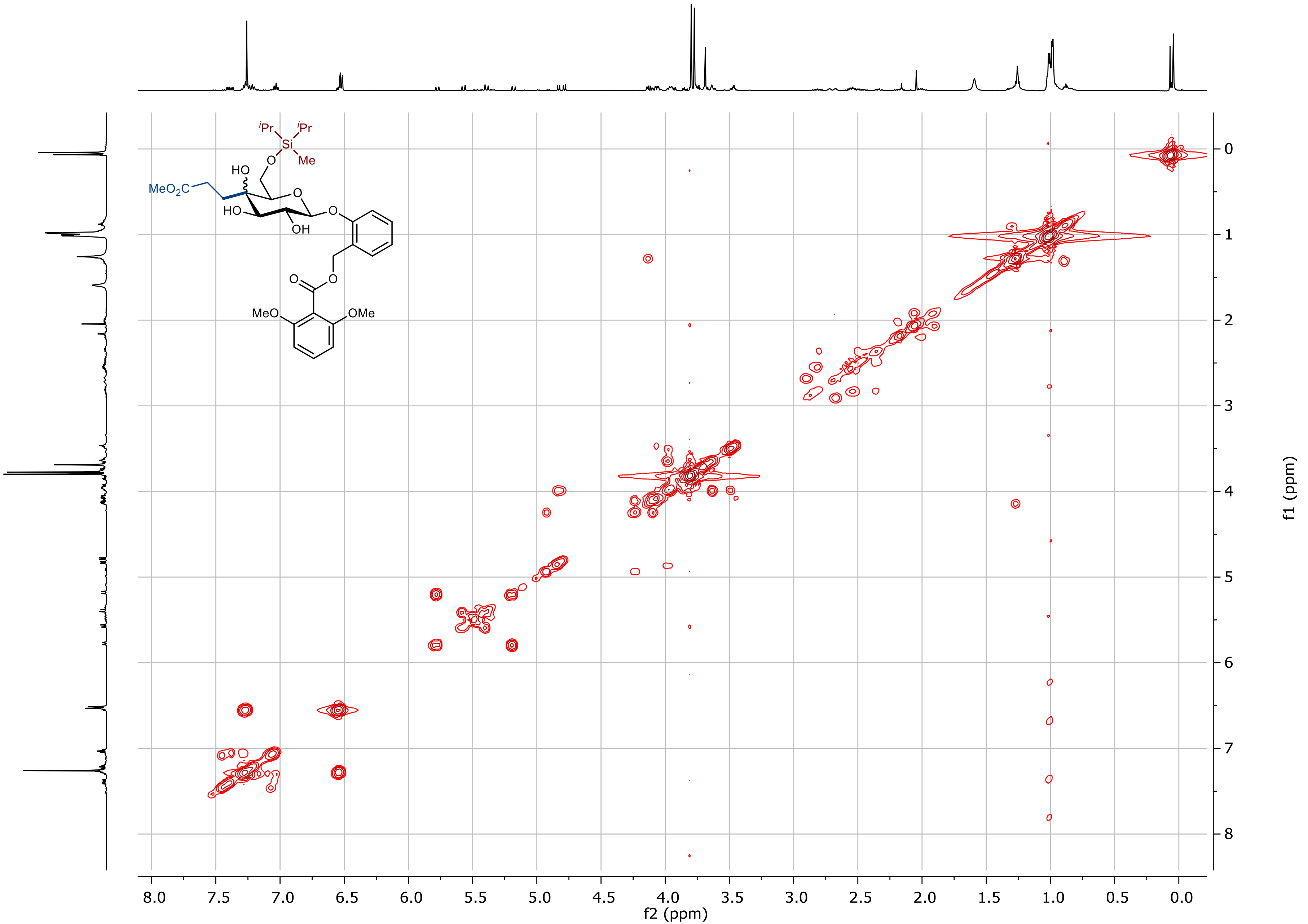

¹H NMR (500 MHz, $CDCl_3$) of compound **3j**

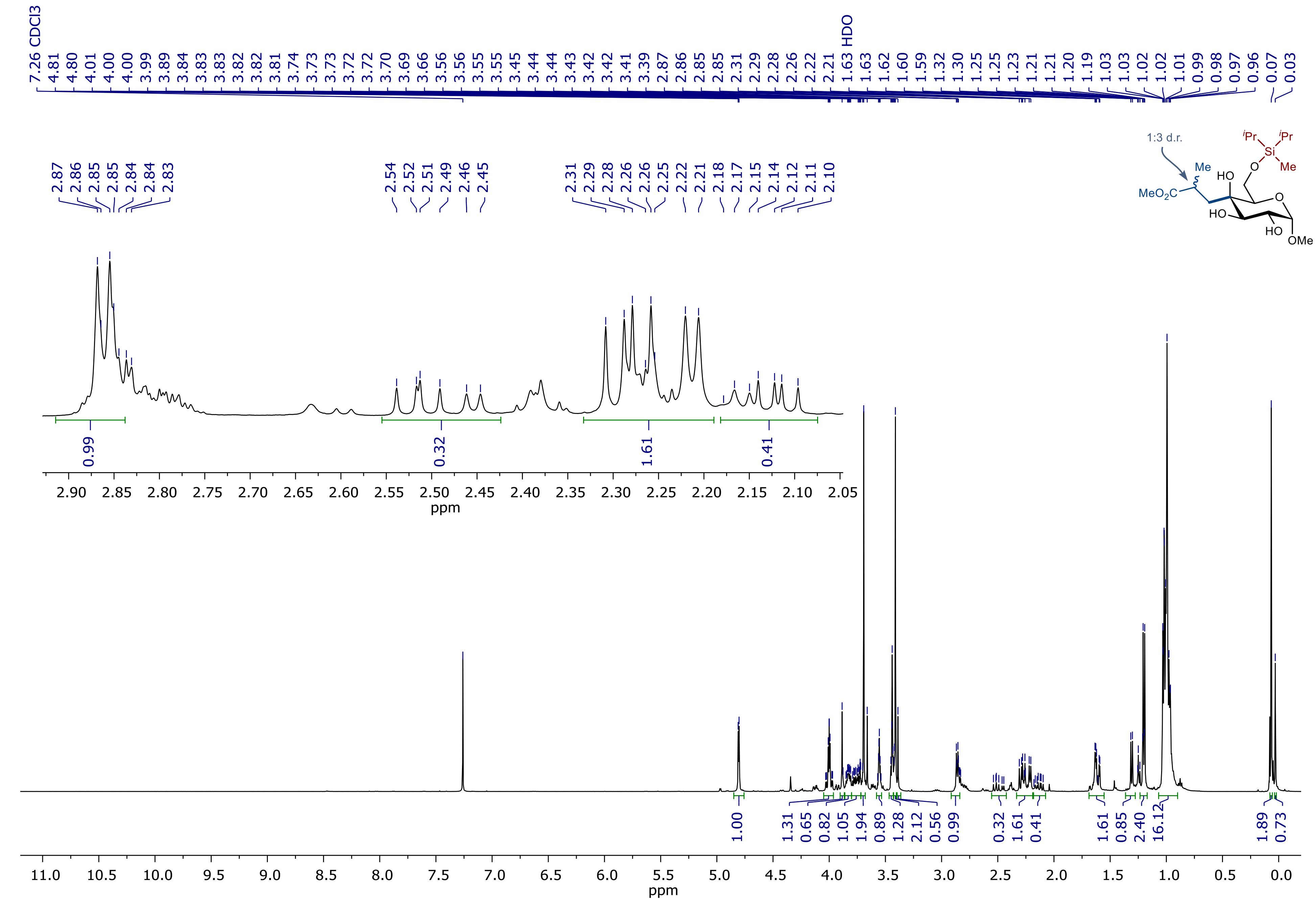

$^{13}C\{^{1}H\}$ NMR (126 MHz, $CDCl_3$) of compound **3j**

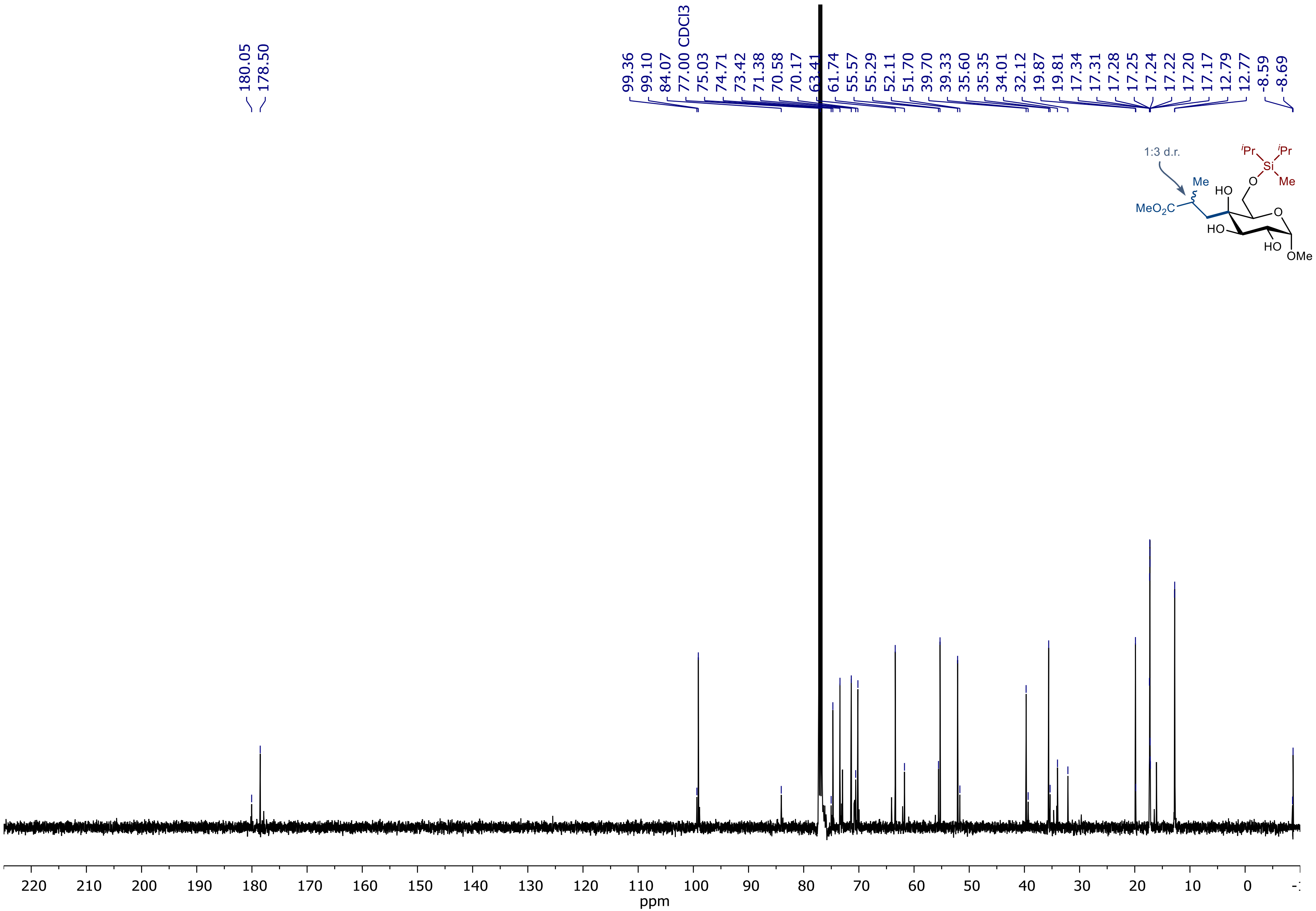

COSY of compound **3j**

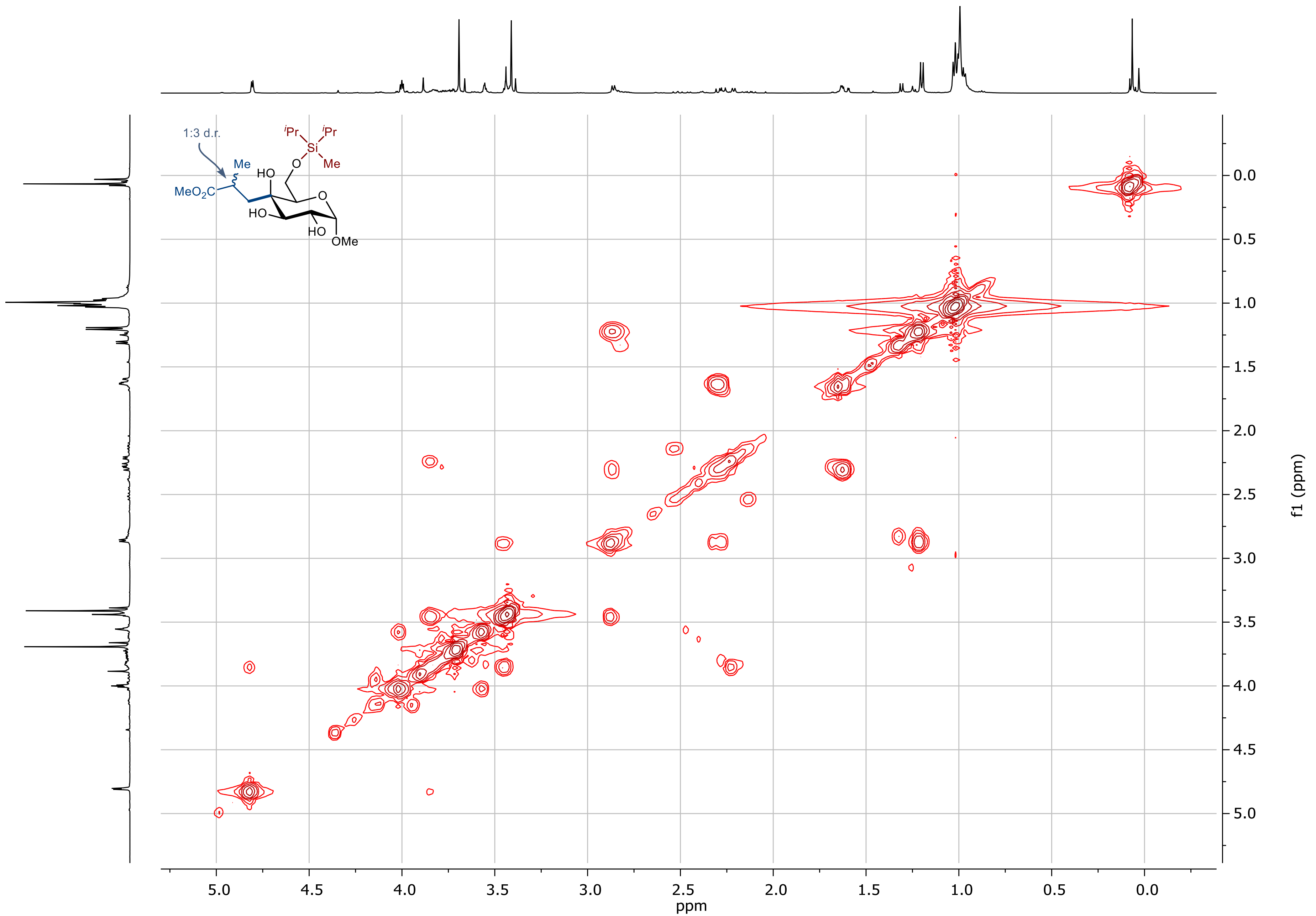

HSQC of compound **3j**

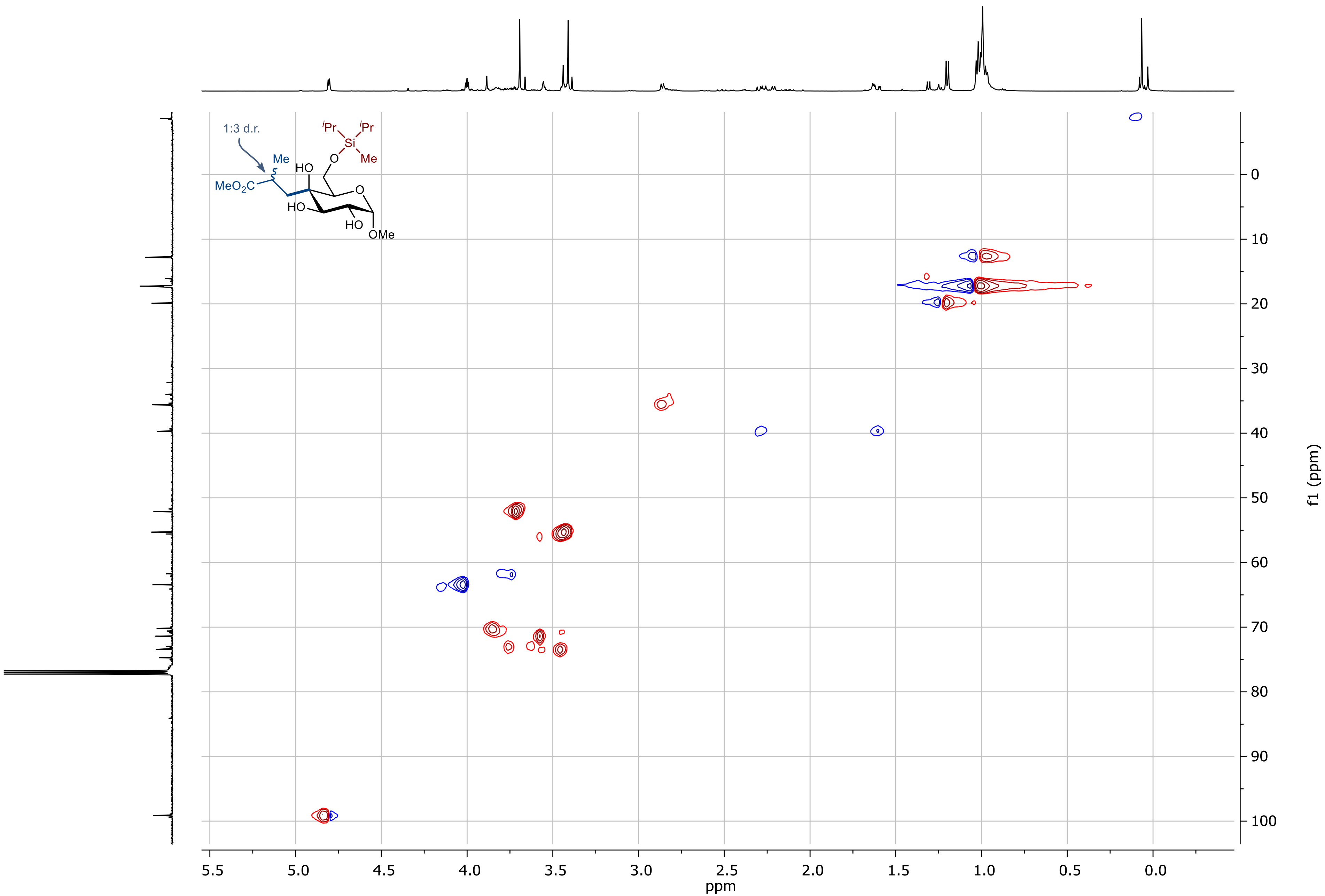

^{1}H NMR (500 MHz, $CDCl_3$) of compound **3k**

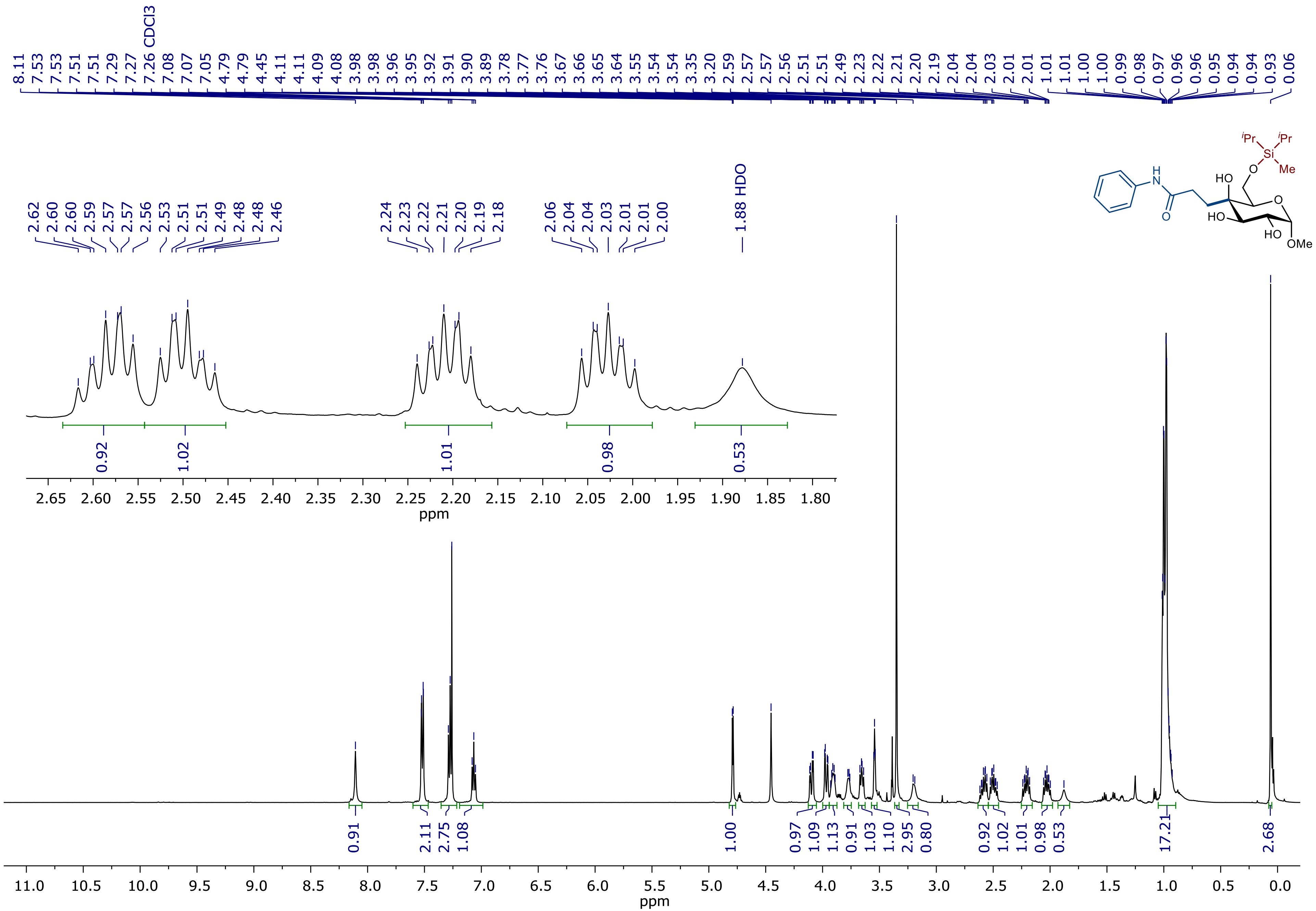

$^{13}C\{^{1}H\}$ **NMR** (126 MHz, $CDCl_3$) of compound **3k**

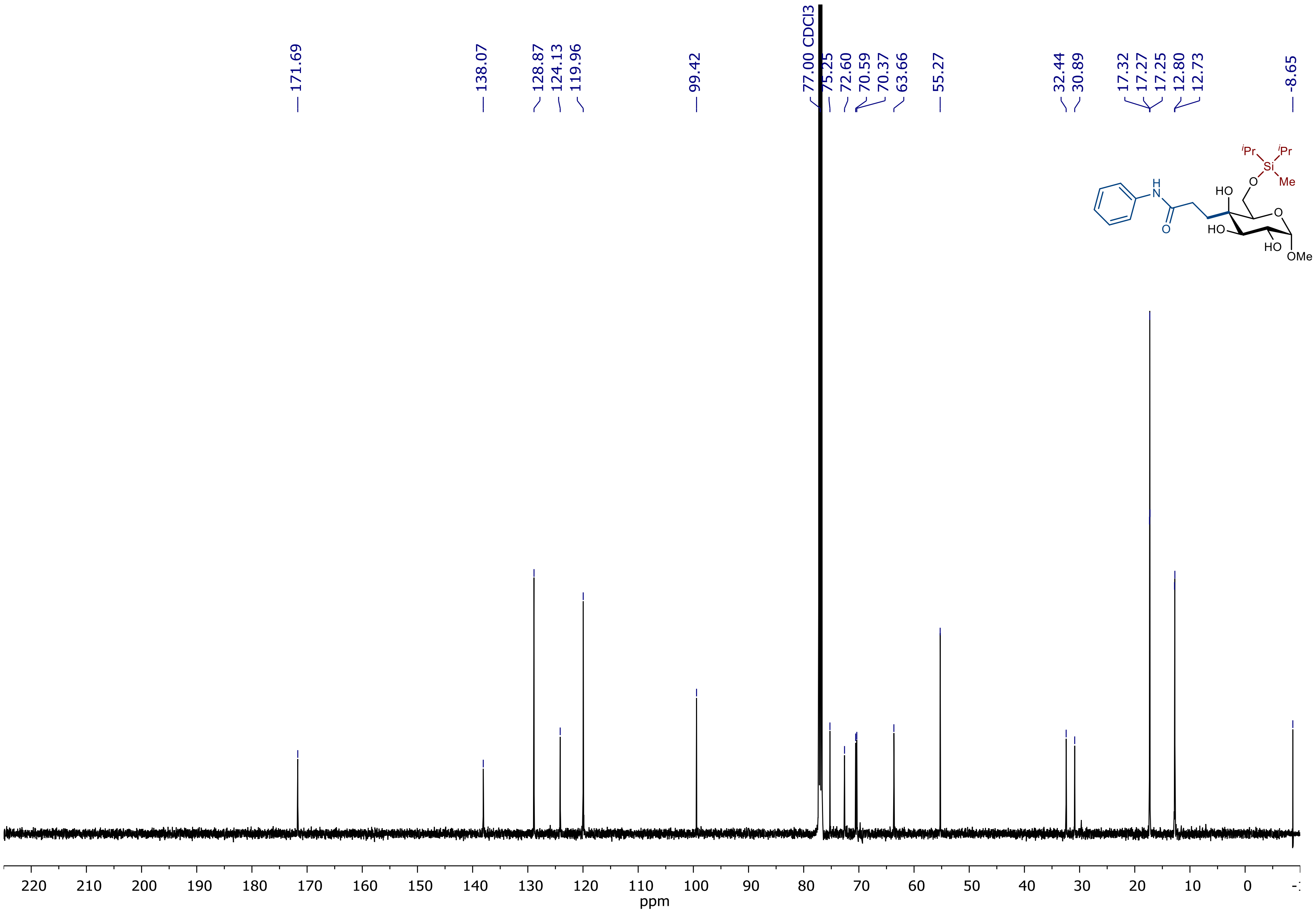

COSY of compound **3k**

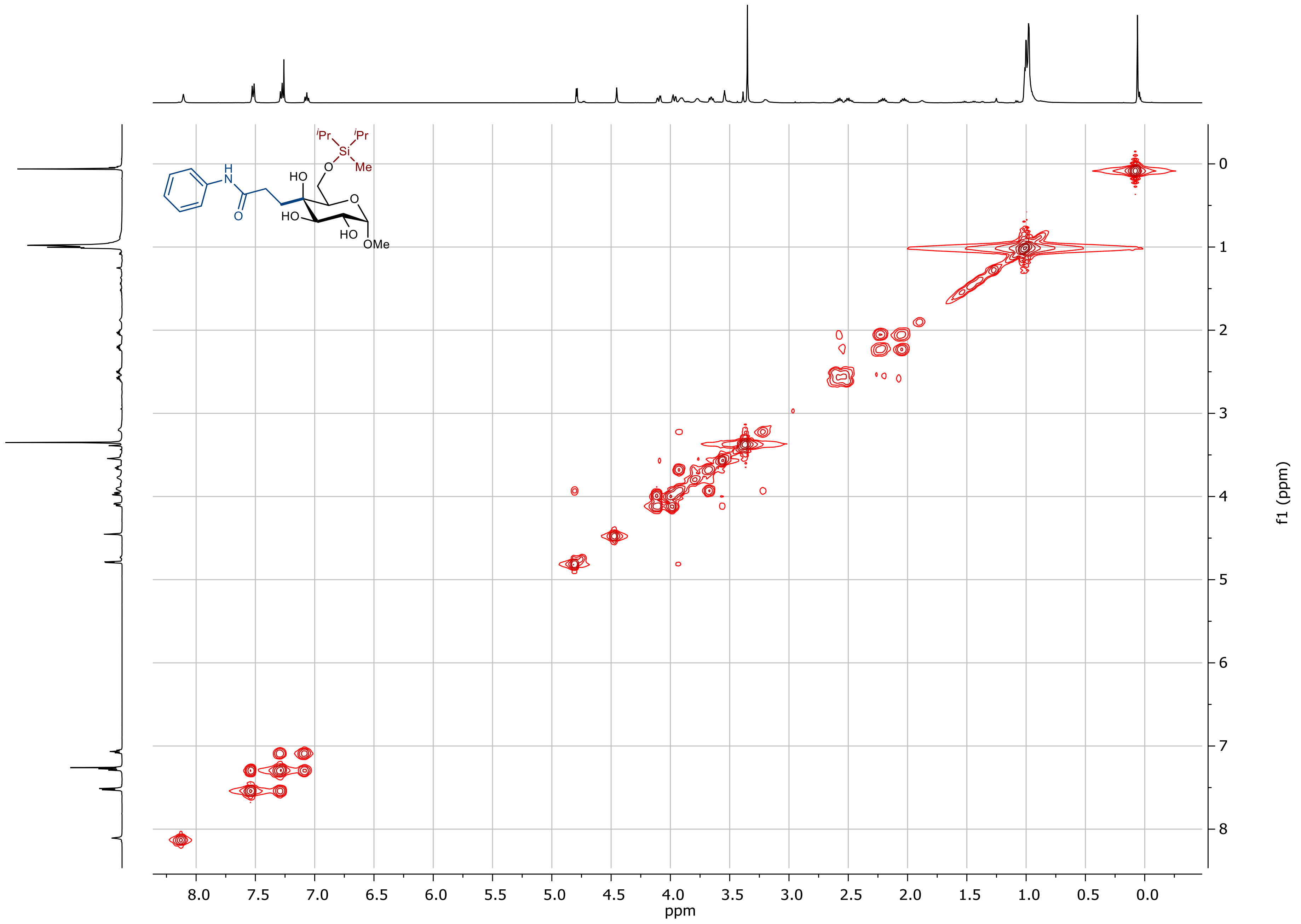

HSQC of compound **3k**

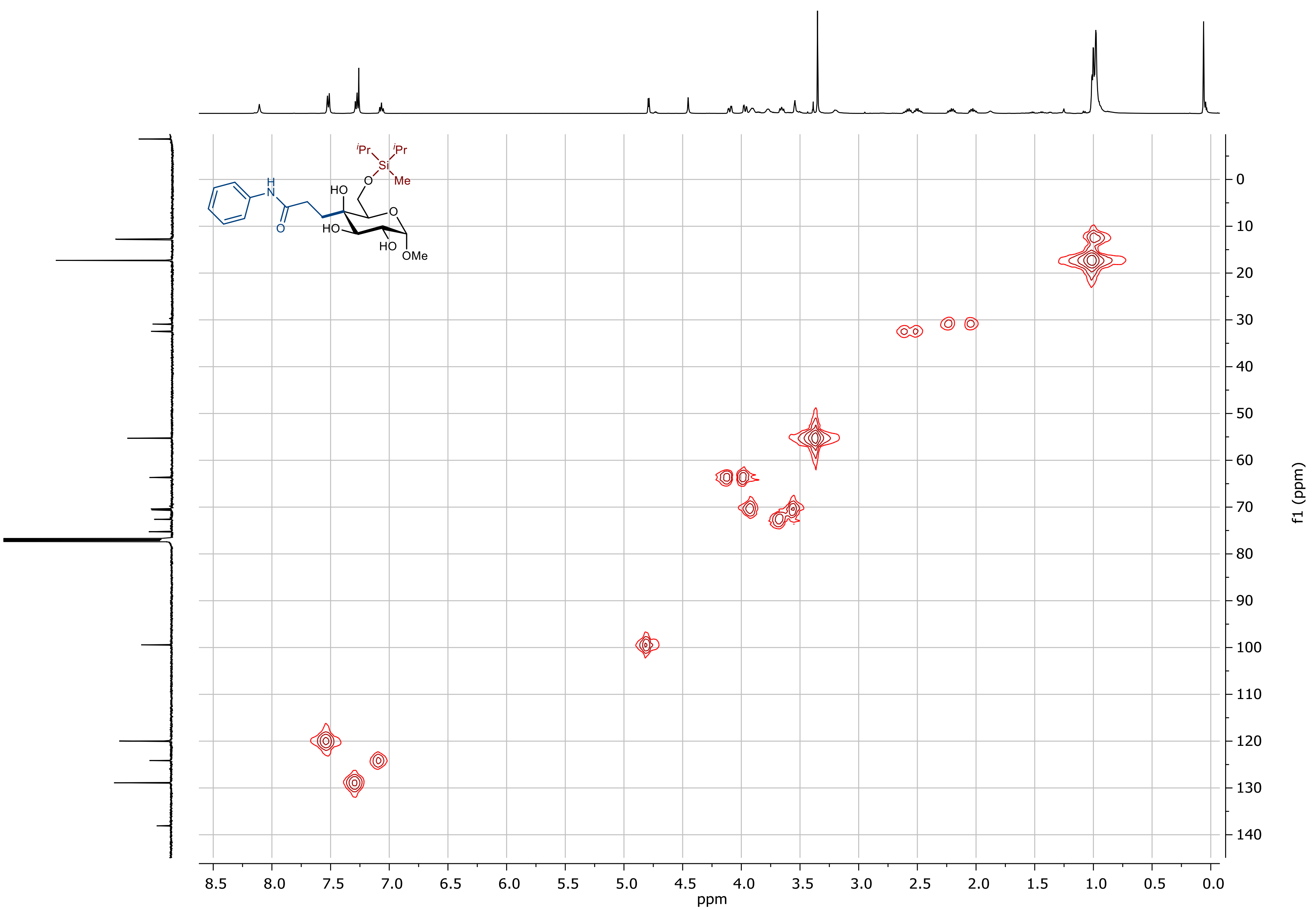

¹H NMR (500 MHz, $CDCl_3$) of compound **3l**

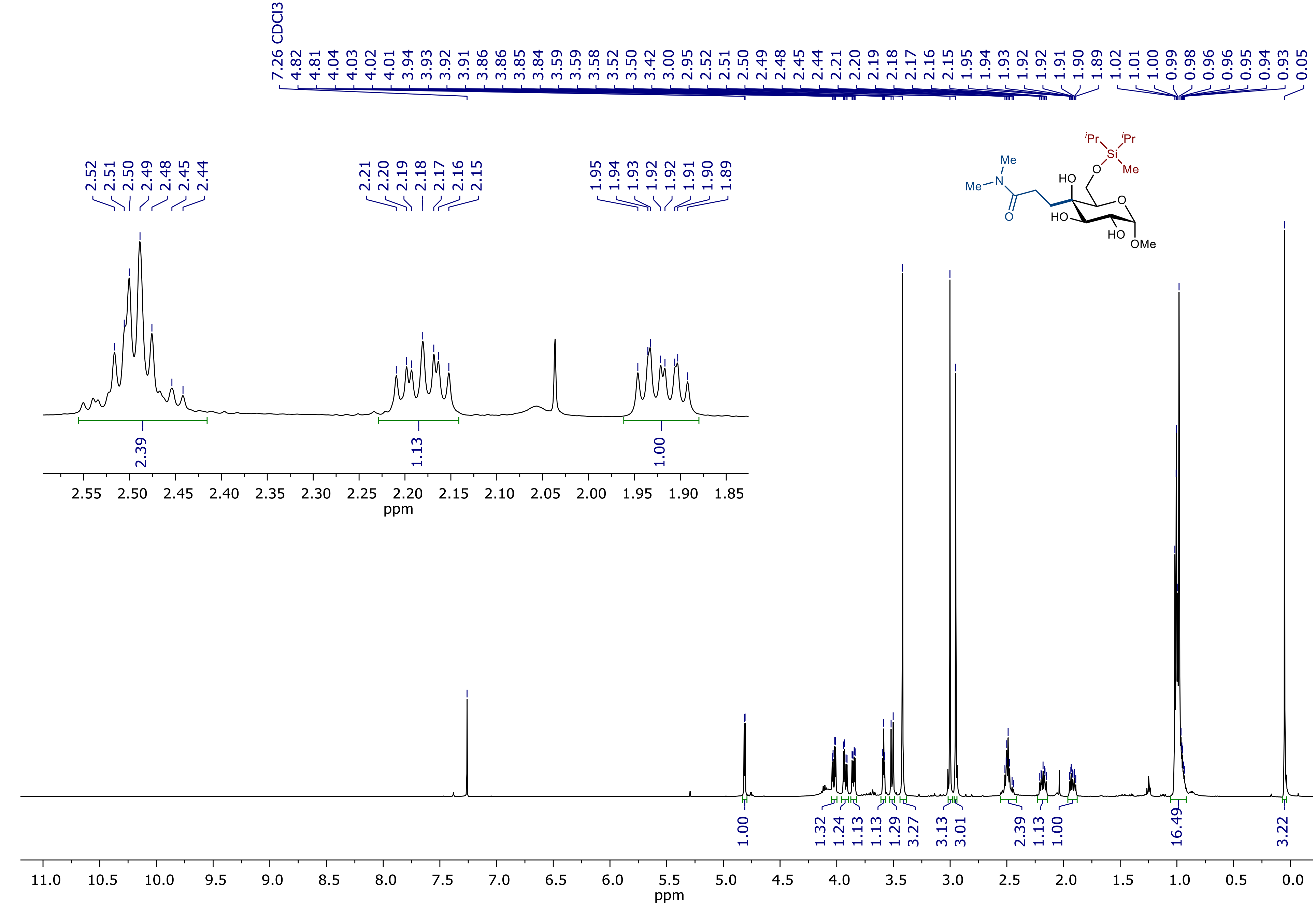

$^{13}C\{^1H\}$ **NMR** (126 MHz, $CDCl_3$) of compound **3l**

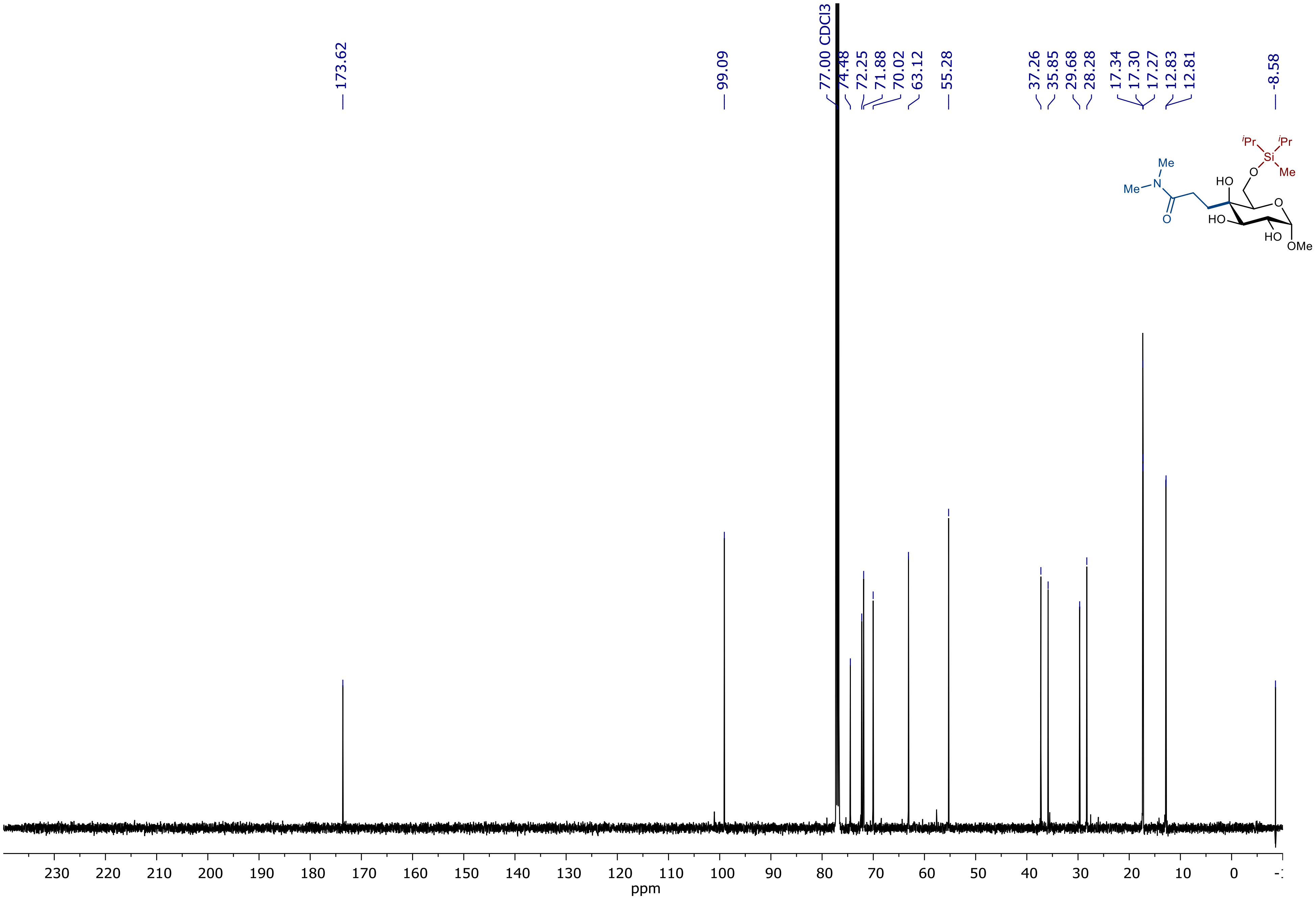

COSY of compound **3l**

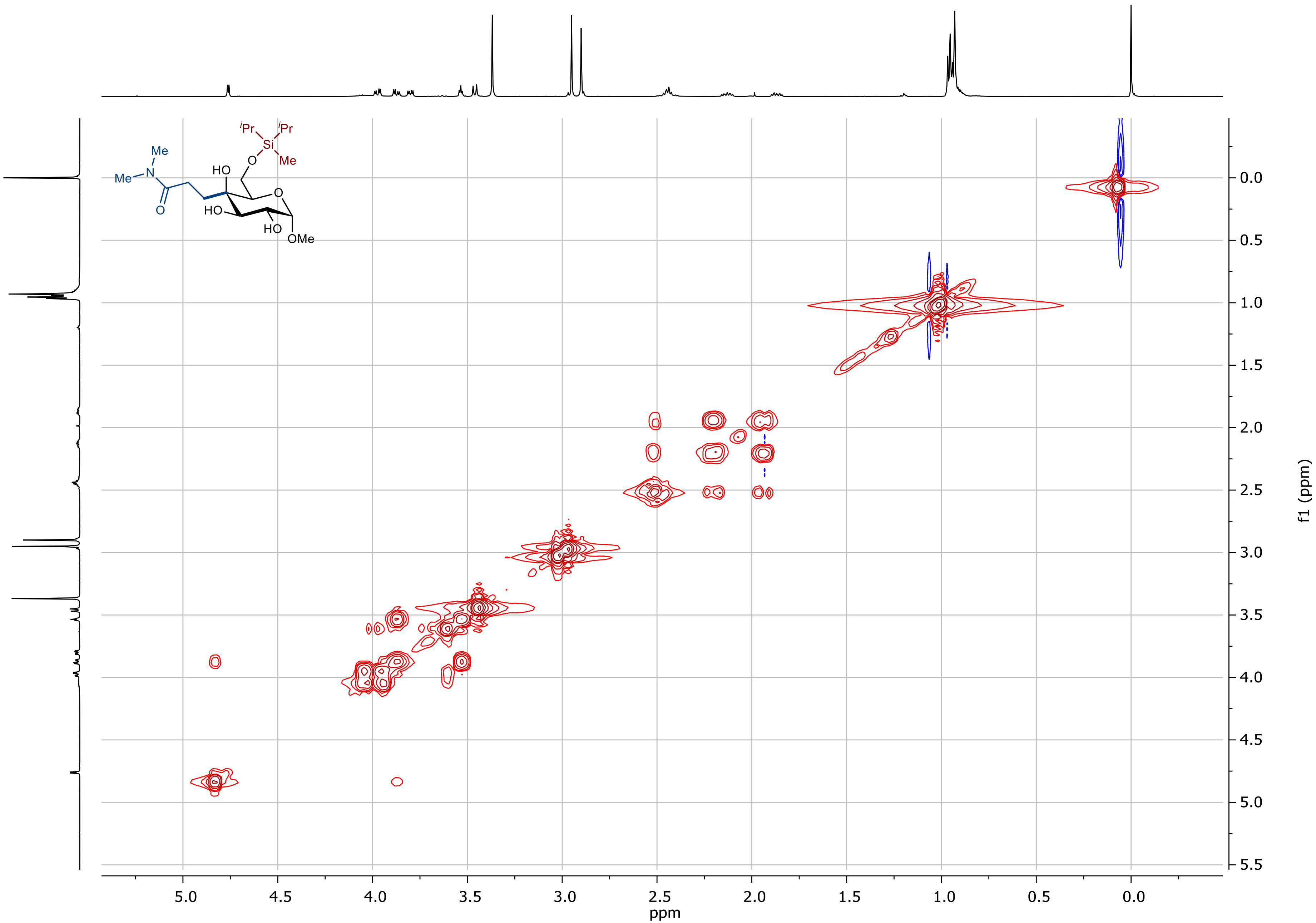

HSQC of compound **3l**

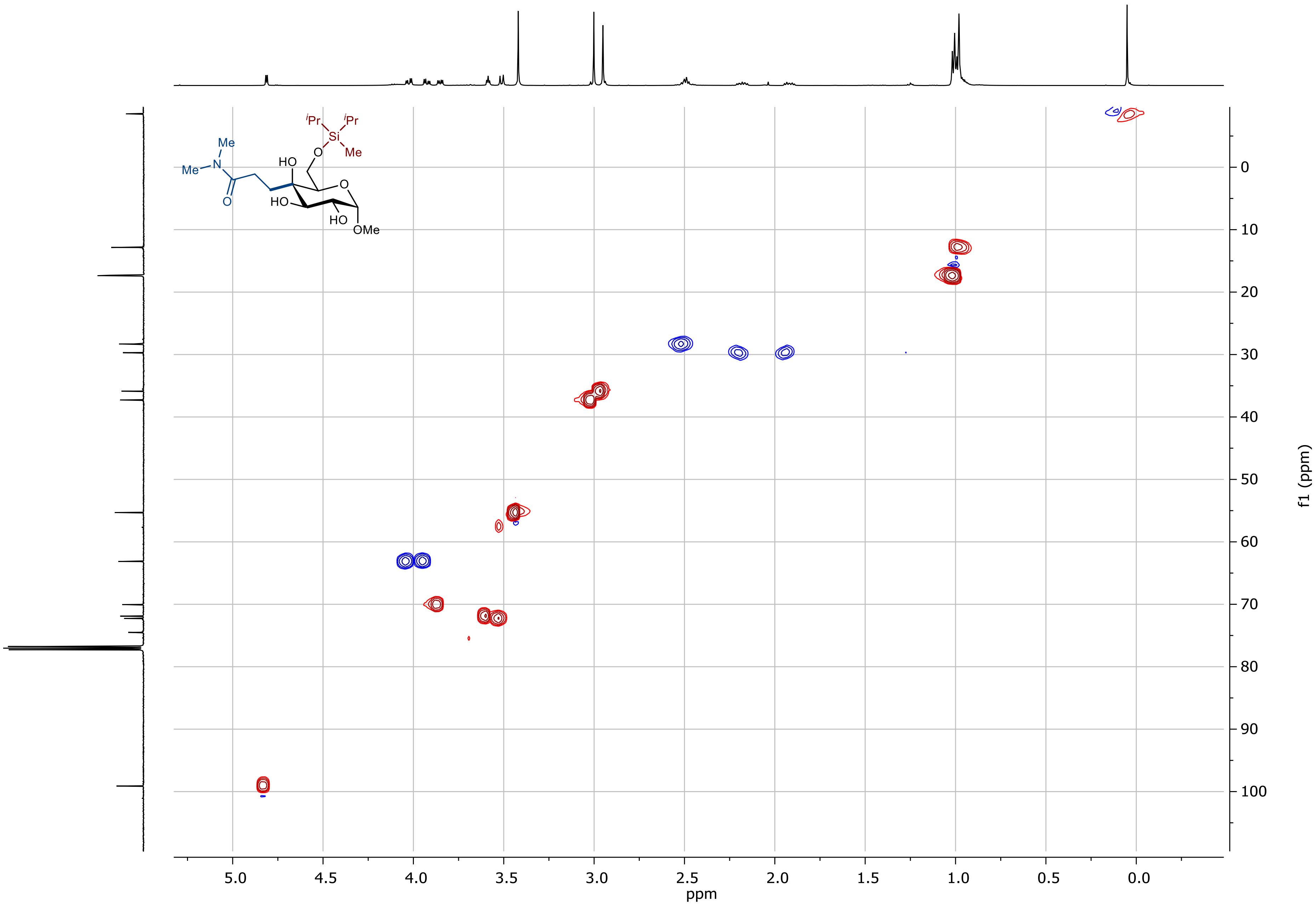

^{1}**H NMR** (500 MHz, $CDCl_3$) of compound **3m**

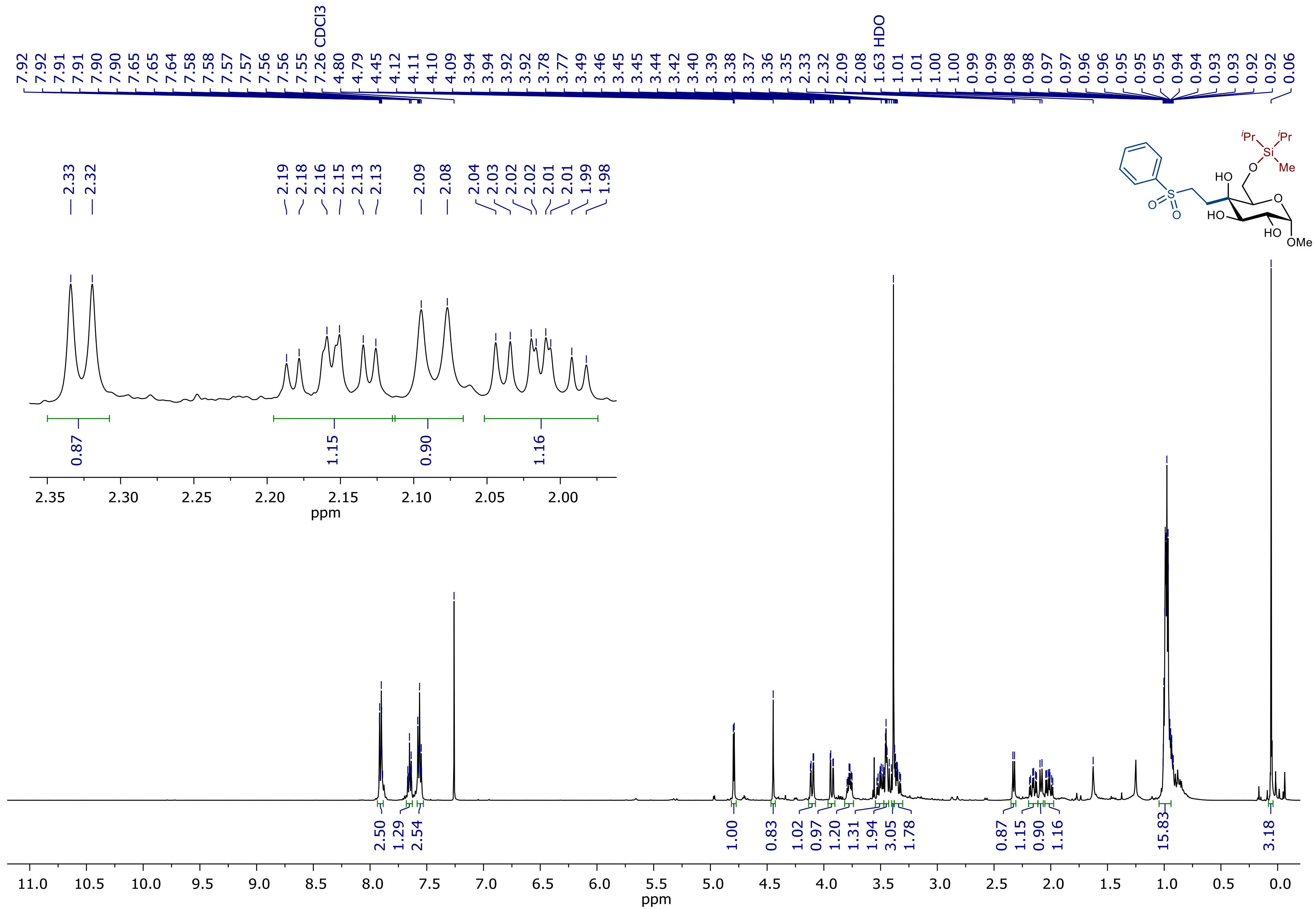

$^{13}C\{^{1}H\}$ NMR (126 MHz, $CDCl_3$) of compound **3m**

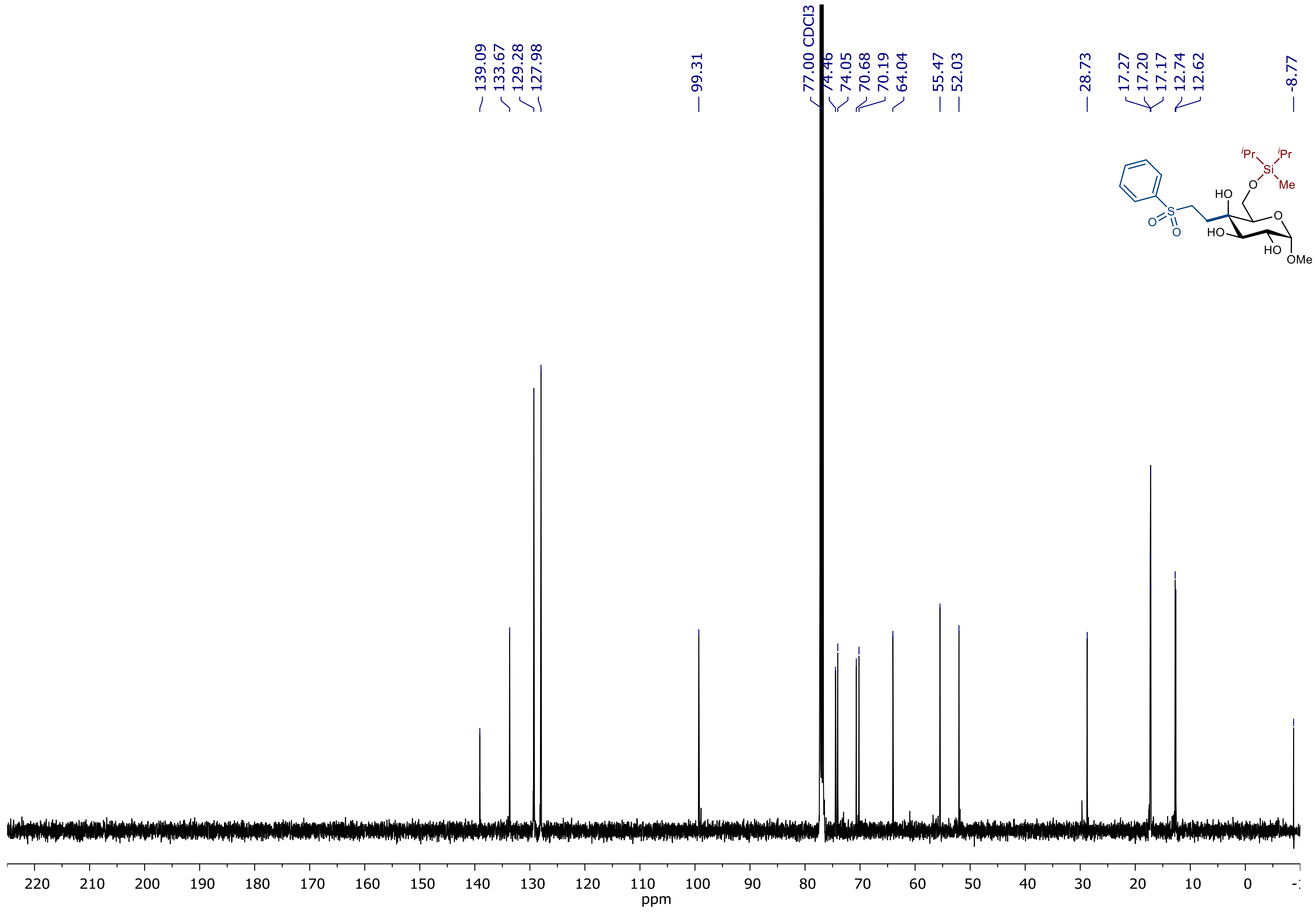

COSY of compound **3m**

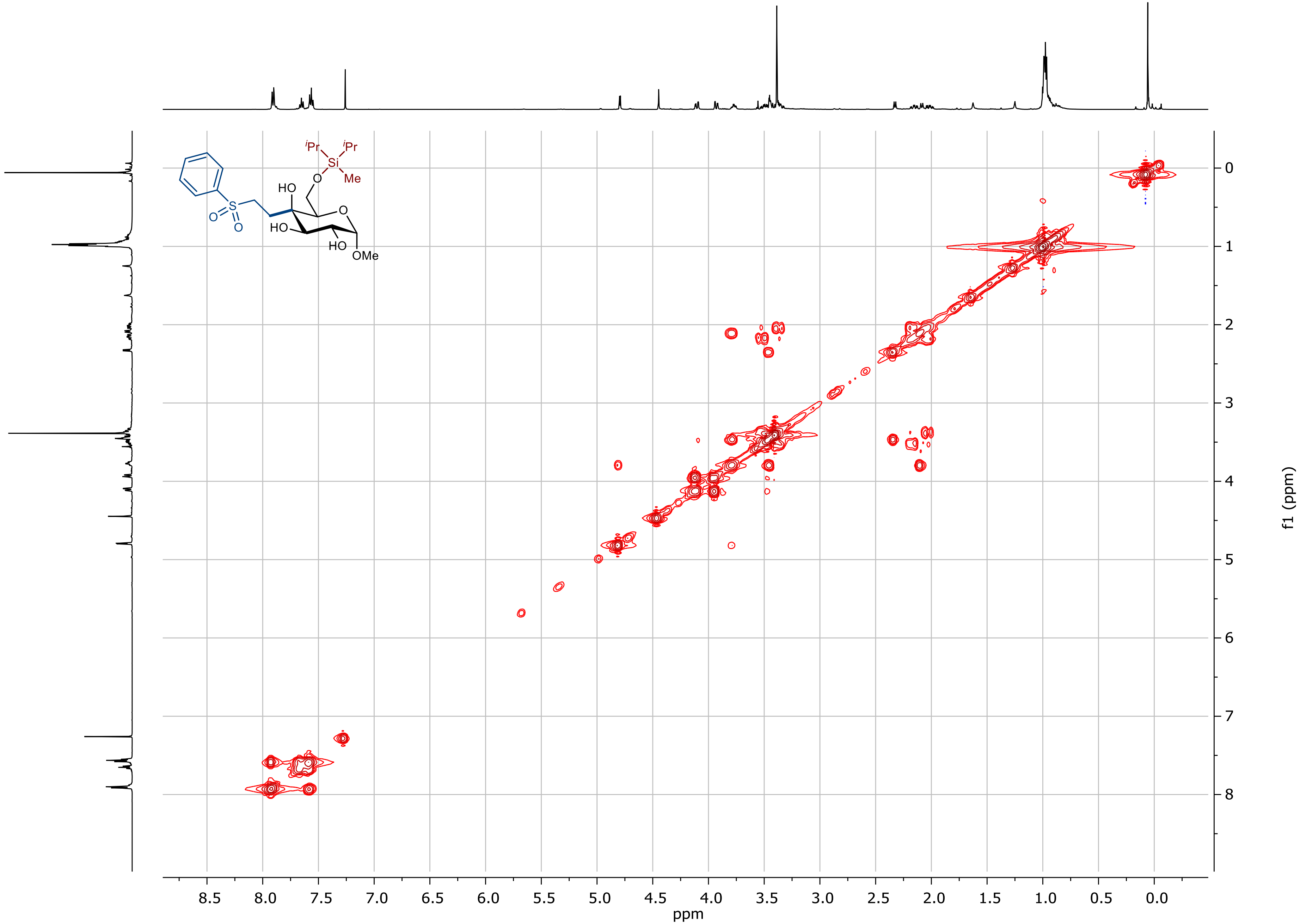

HSQC of compound **3m**

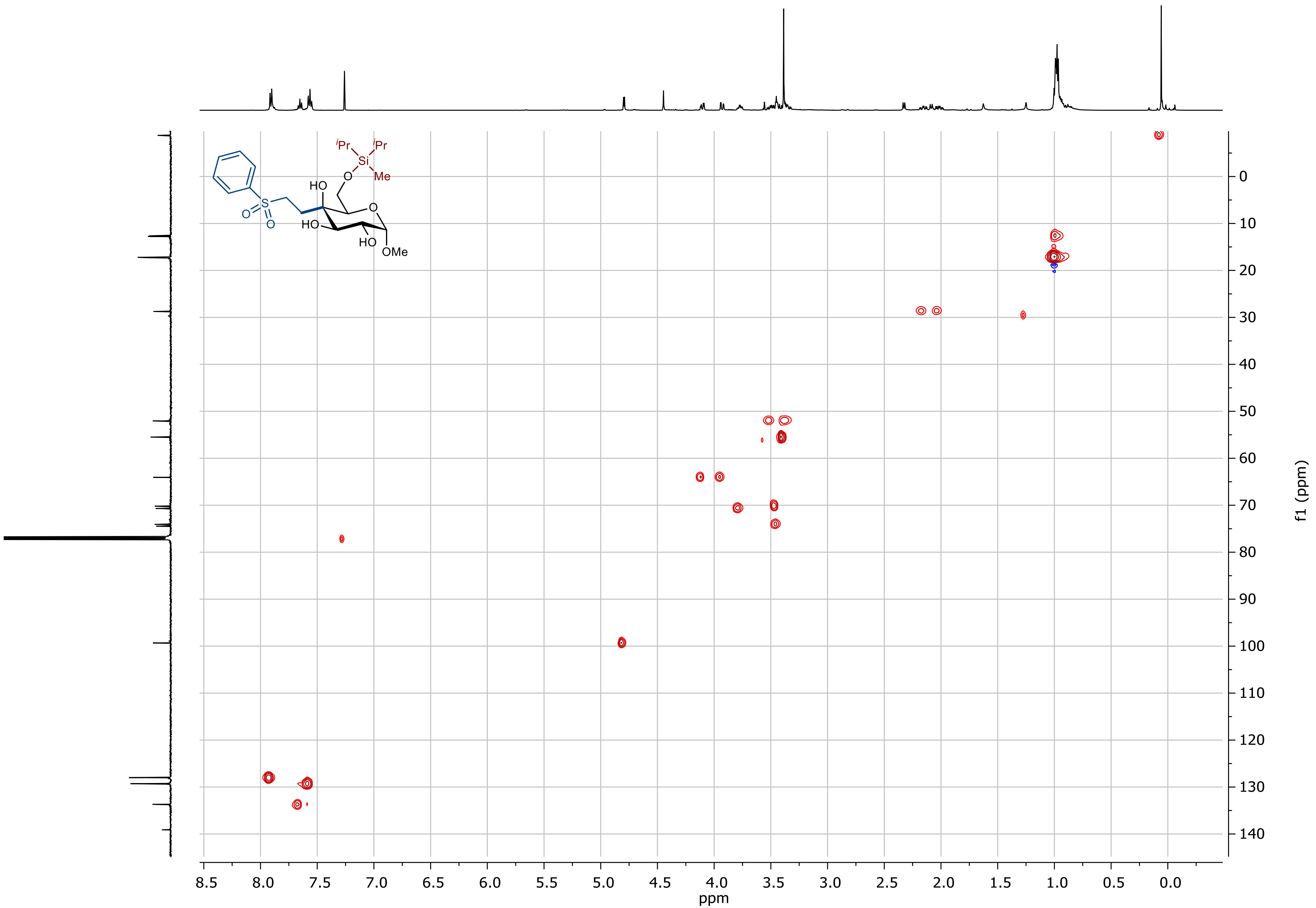

^{1}H NMR (500 MHz, $CDCl_3$) of compound **3n**

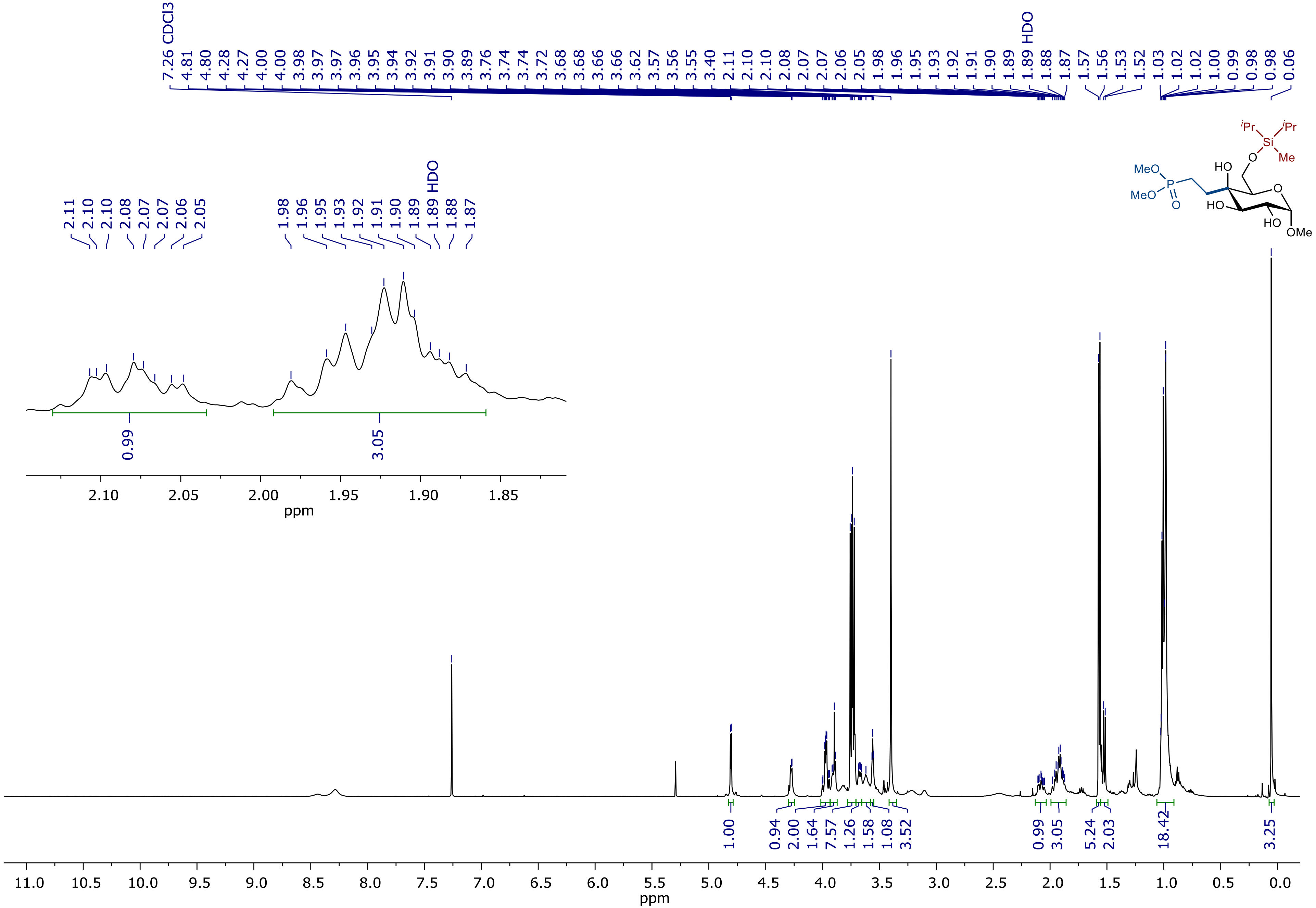

$^{13}C\{^{1}H\}$ **NMR** (126 MHz, $CDCl_3$) of compound **3n**

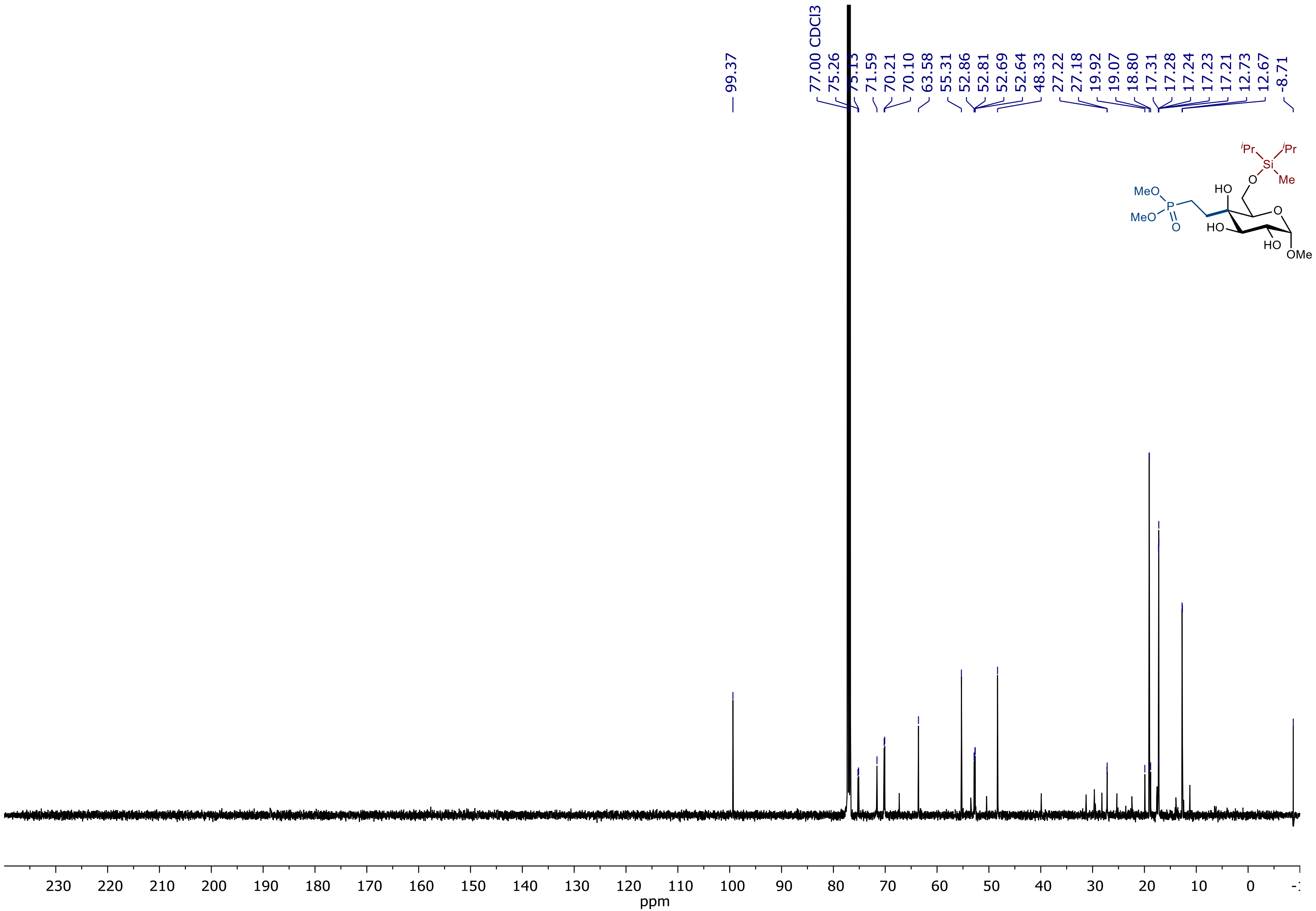

COSY of compound **3n**

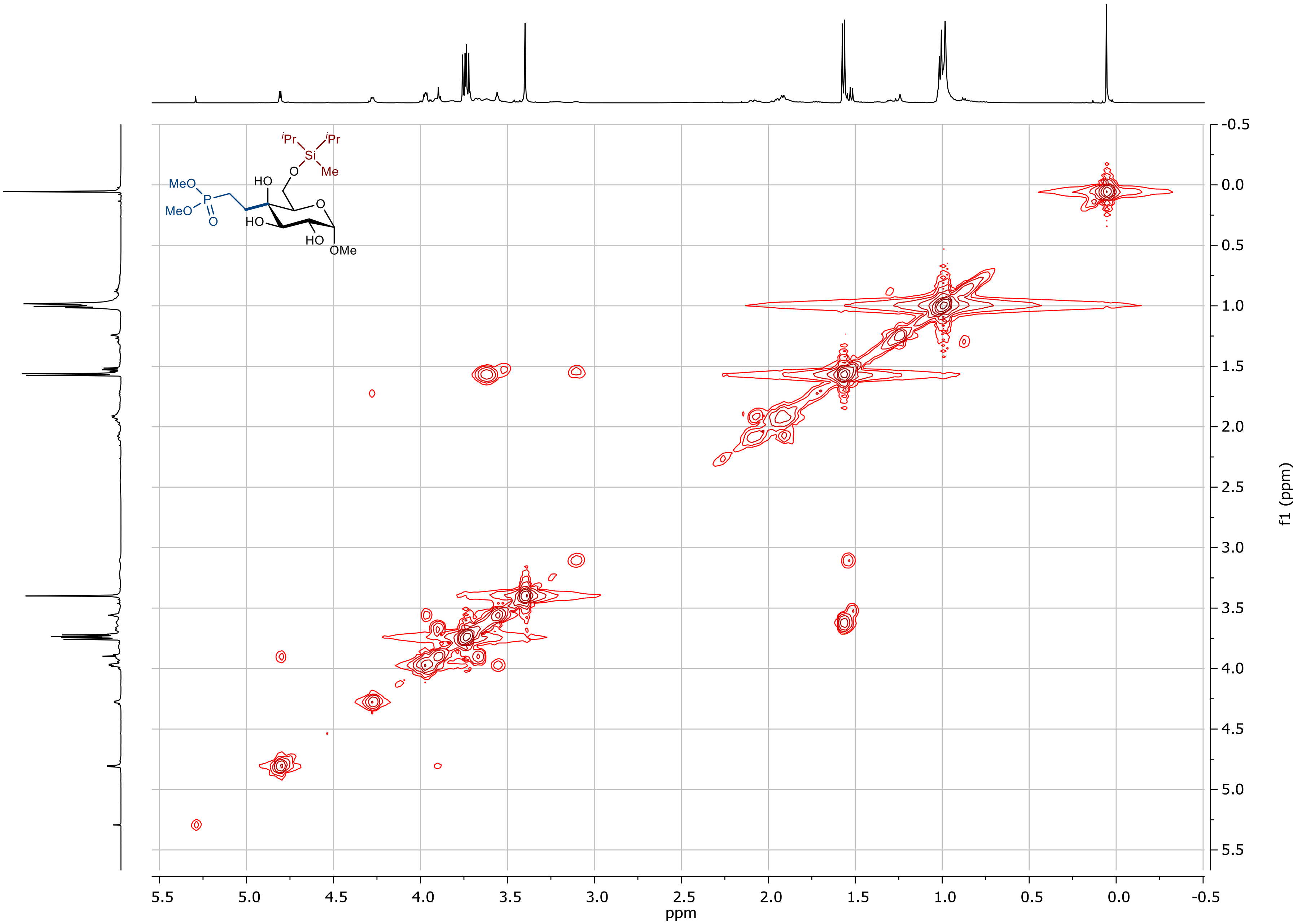

HSQC of compound **3n**

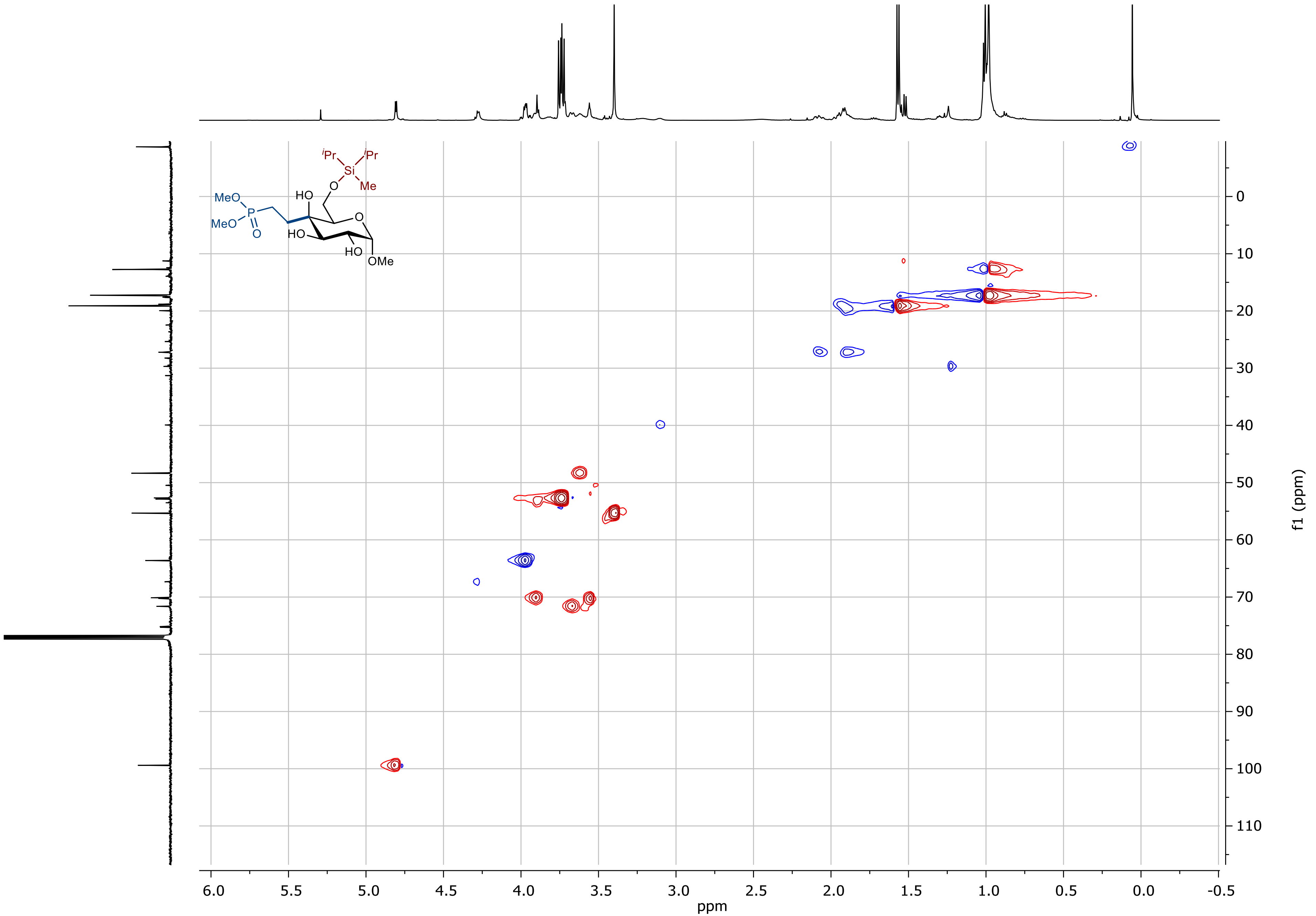

¹H NMR (500 MHz, $CDCl_3$) of compound **3o**

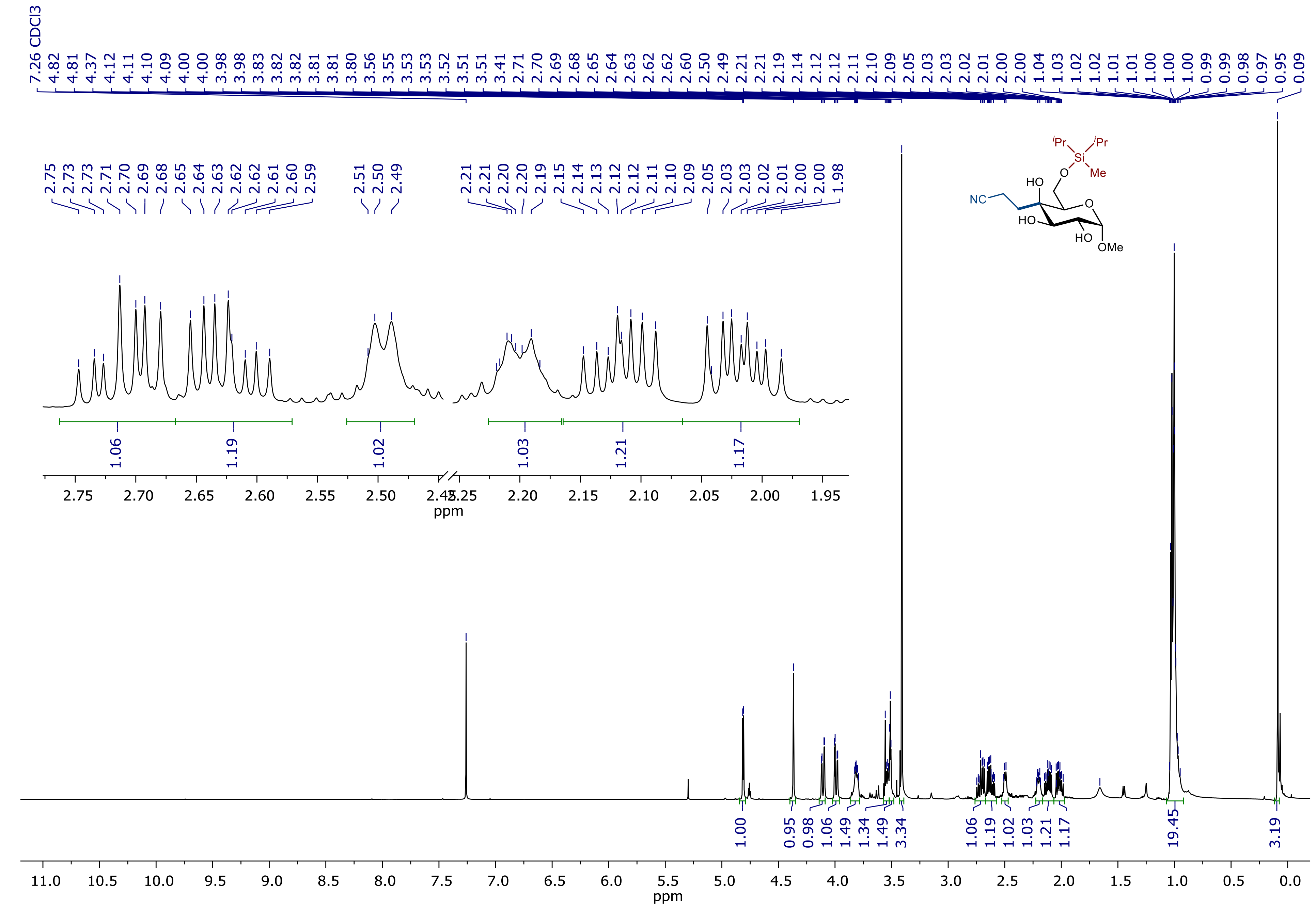

$^{13}C\{^{1}H\}$ NMR (126 MHz, $CDCl_3$) of compound **3o**

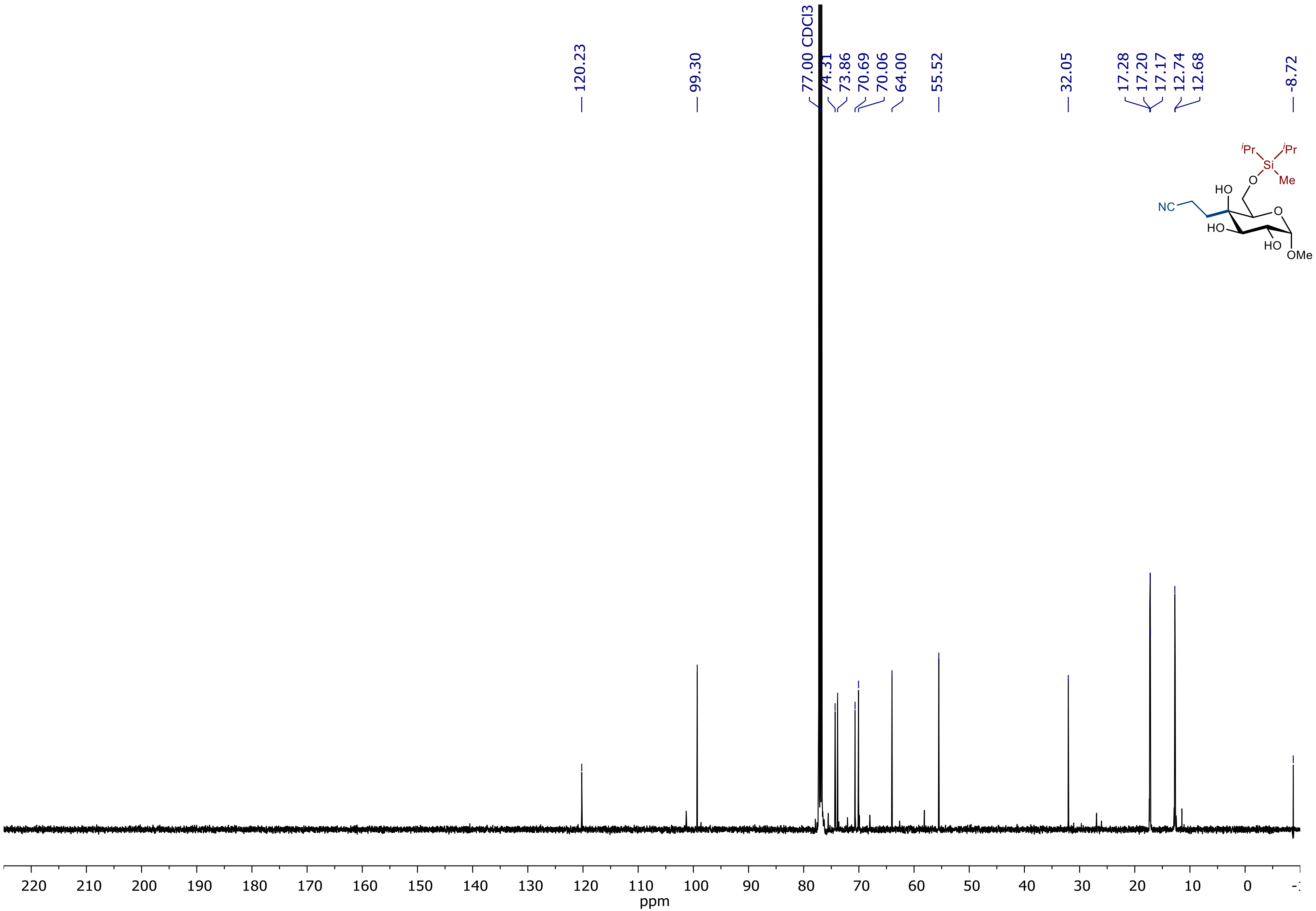

COSY of compound **3o**

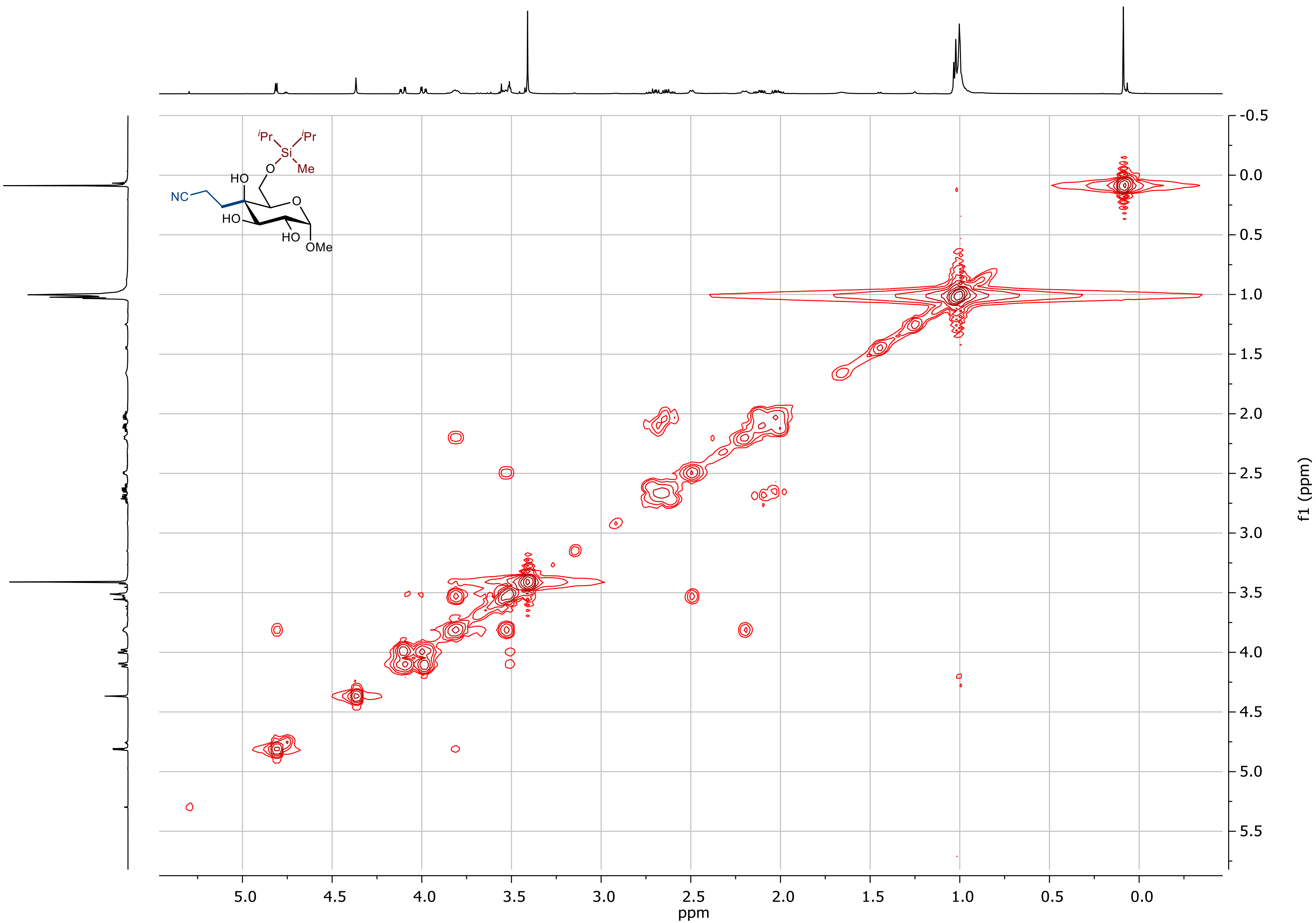

HSQC of compound **3o**

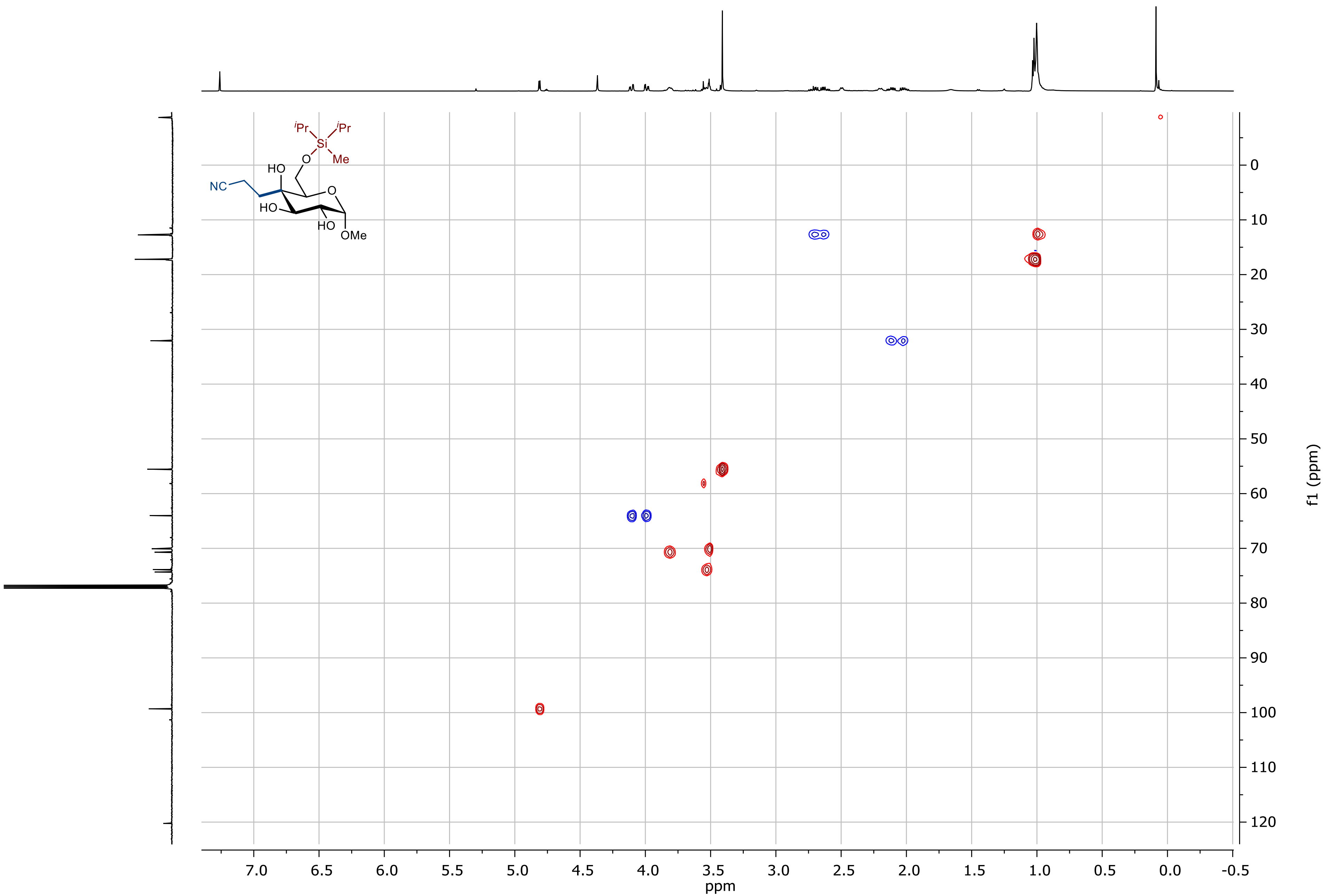

^{1}H NMR (500 MHz, $CDCl_3$) of compound **3p**

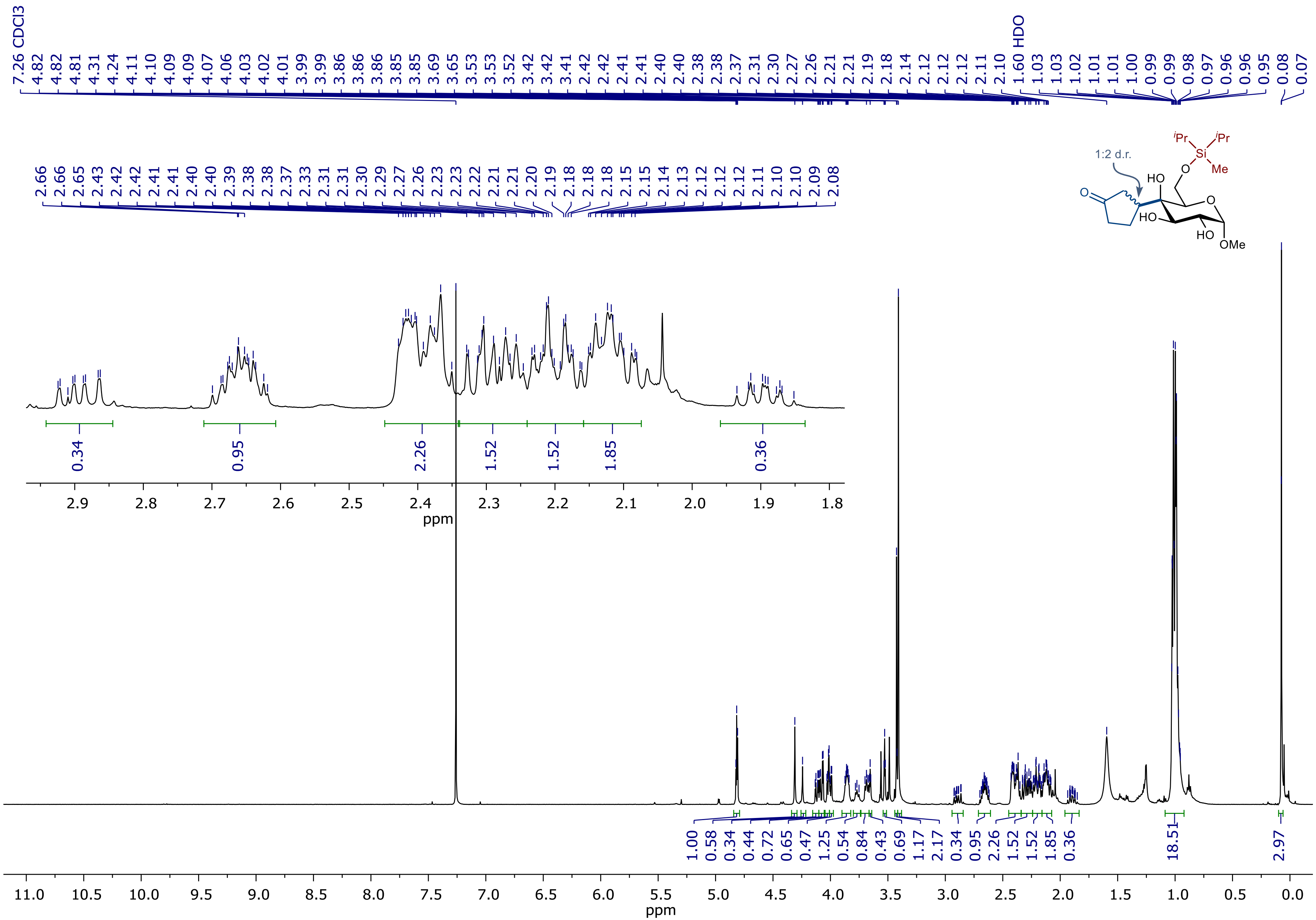

$^{13}C\{^1H\}$ NMR (126 MHz, $CDCl_3$) of compound **3p**

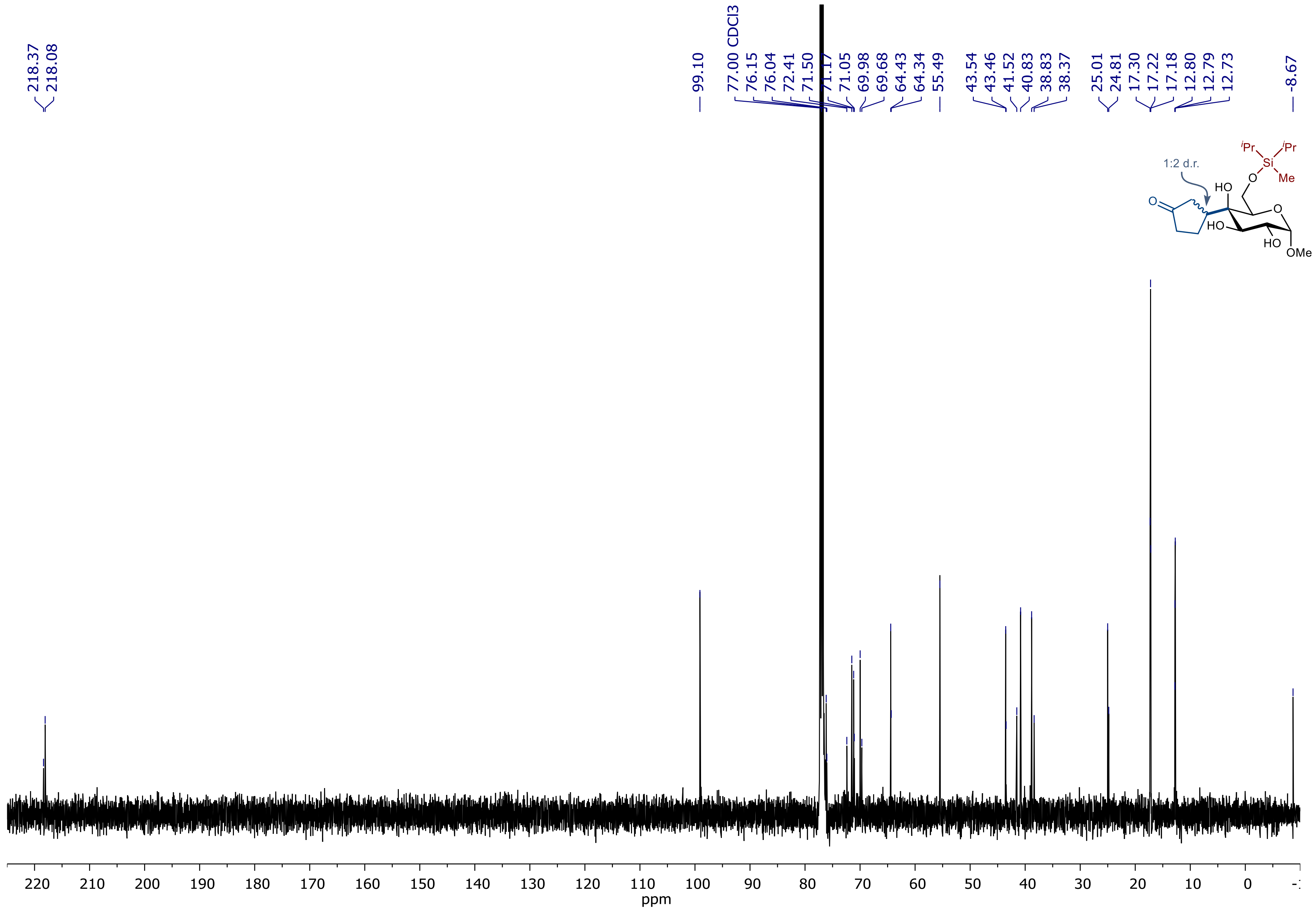

COSY of compound **3p**

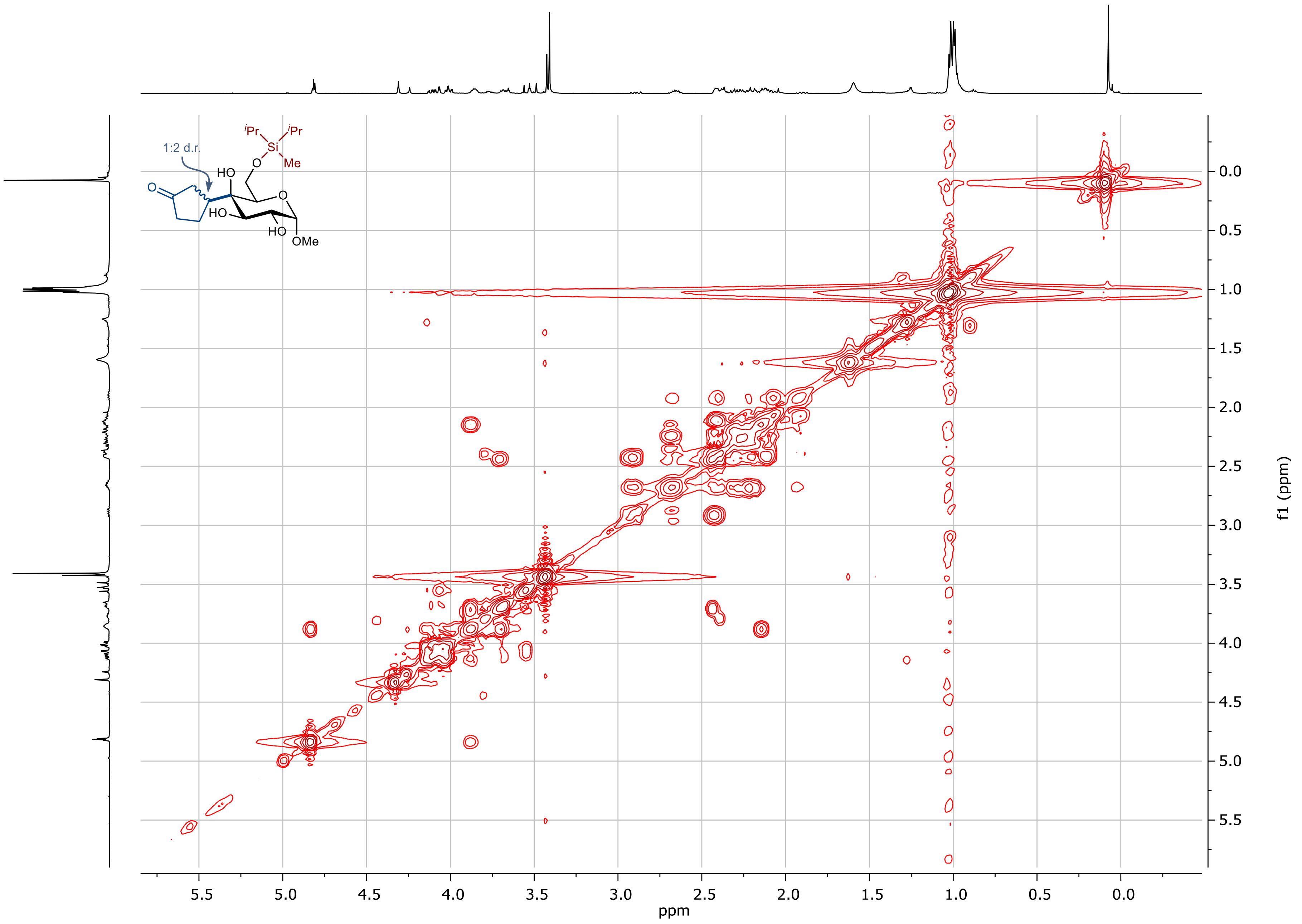

HSQC of compound **3p**

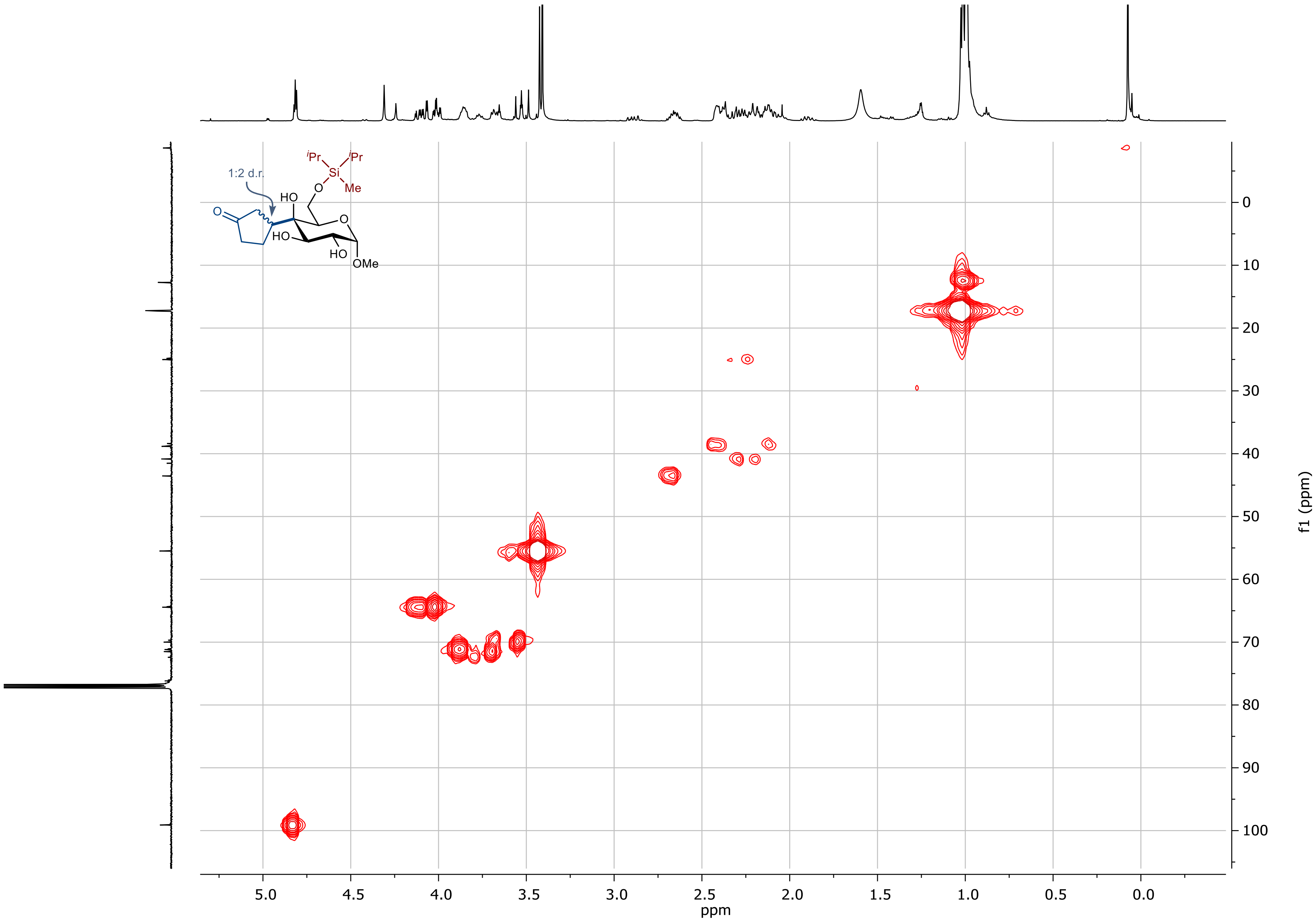

^{1}H NMR (500 MHz, $CDCl_3$) of compound **3q**

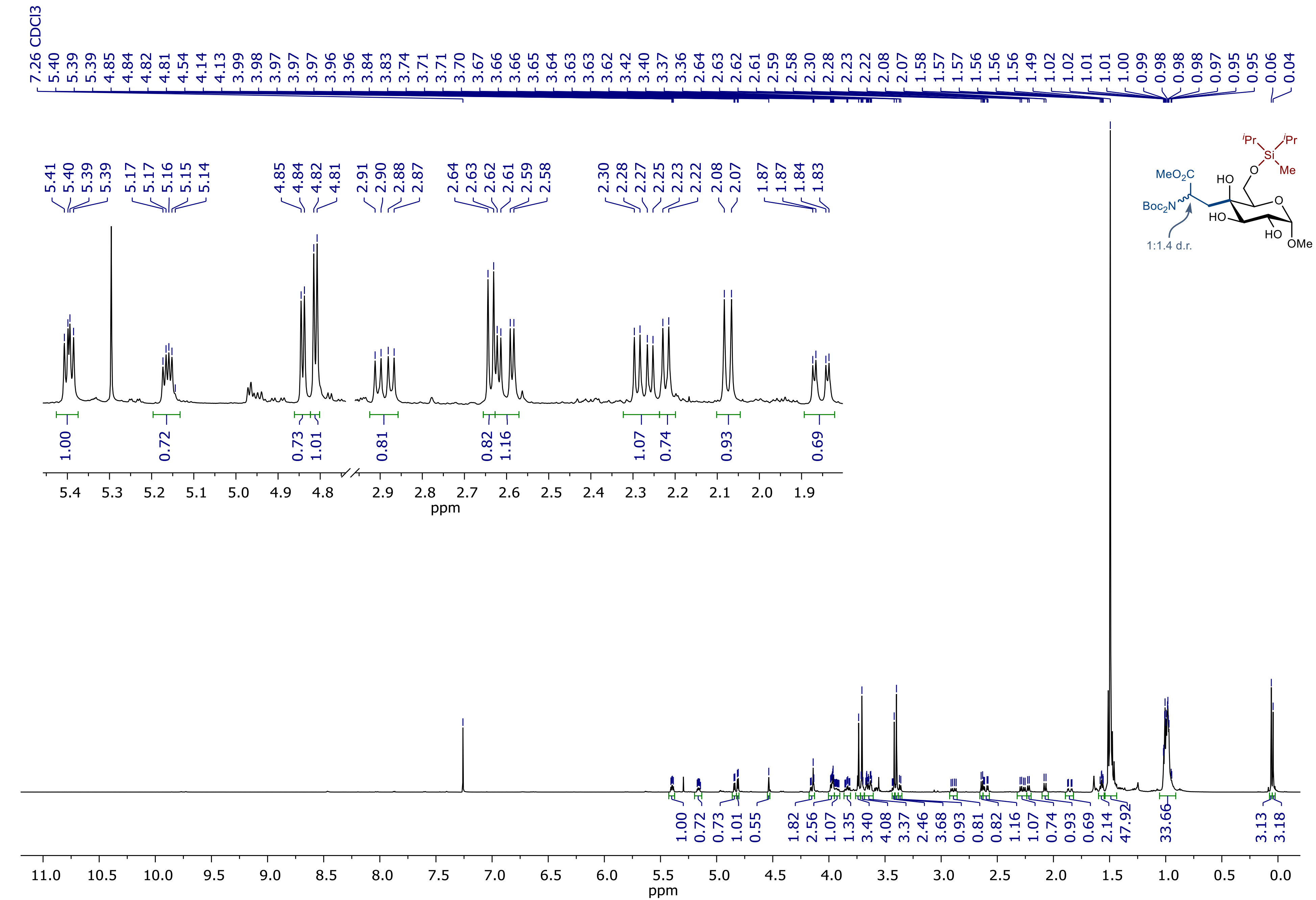

$^{13}C\{^{1}H\}$ NMR (126 MHz, $CDCl_3$) of compound **3q**

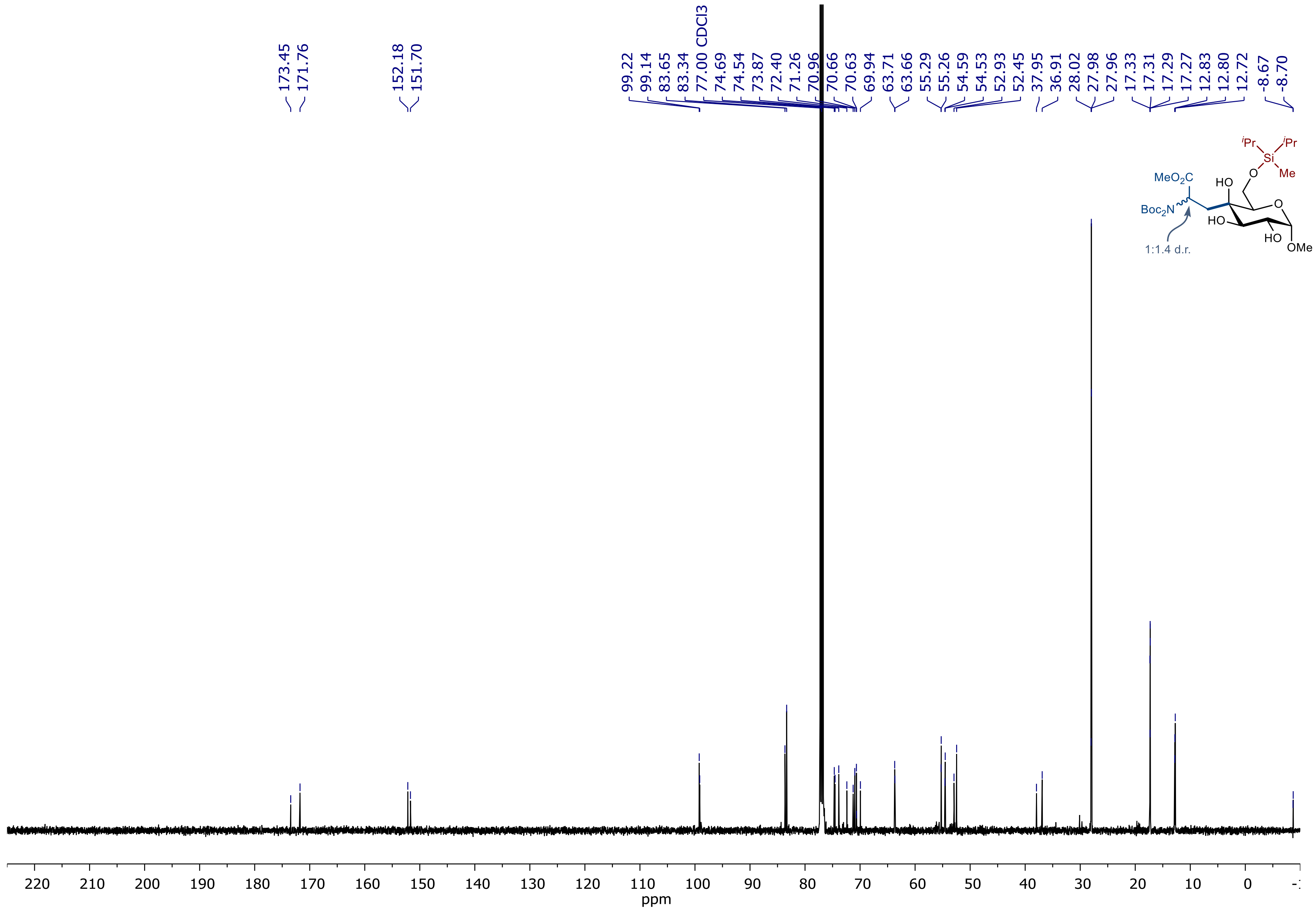

COSY of compound **3q**

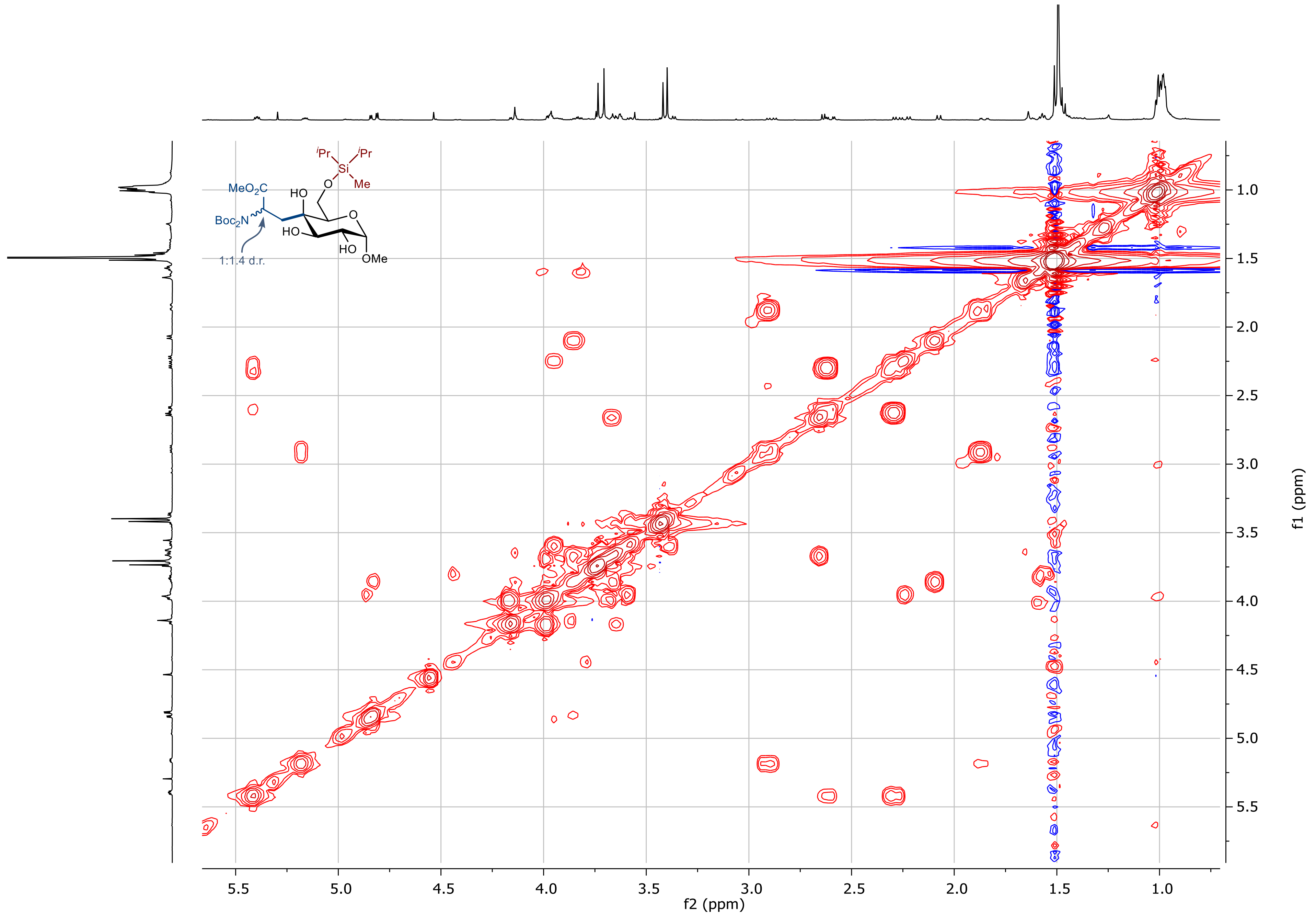

HSQC of compound **3q**

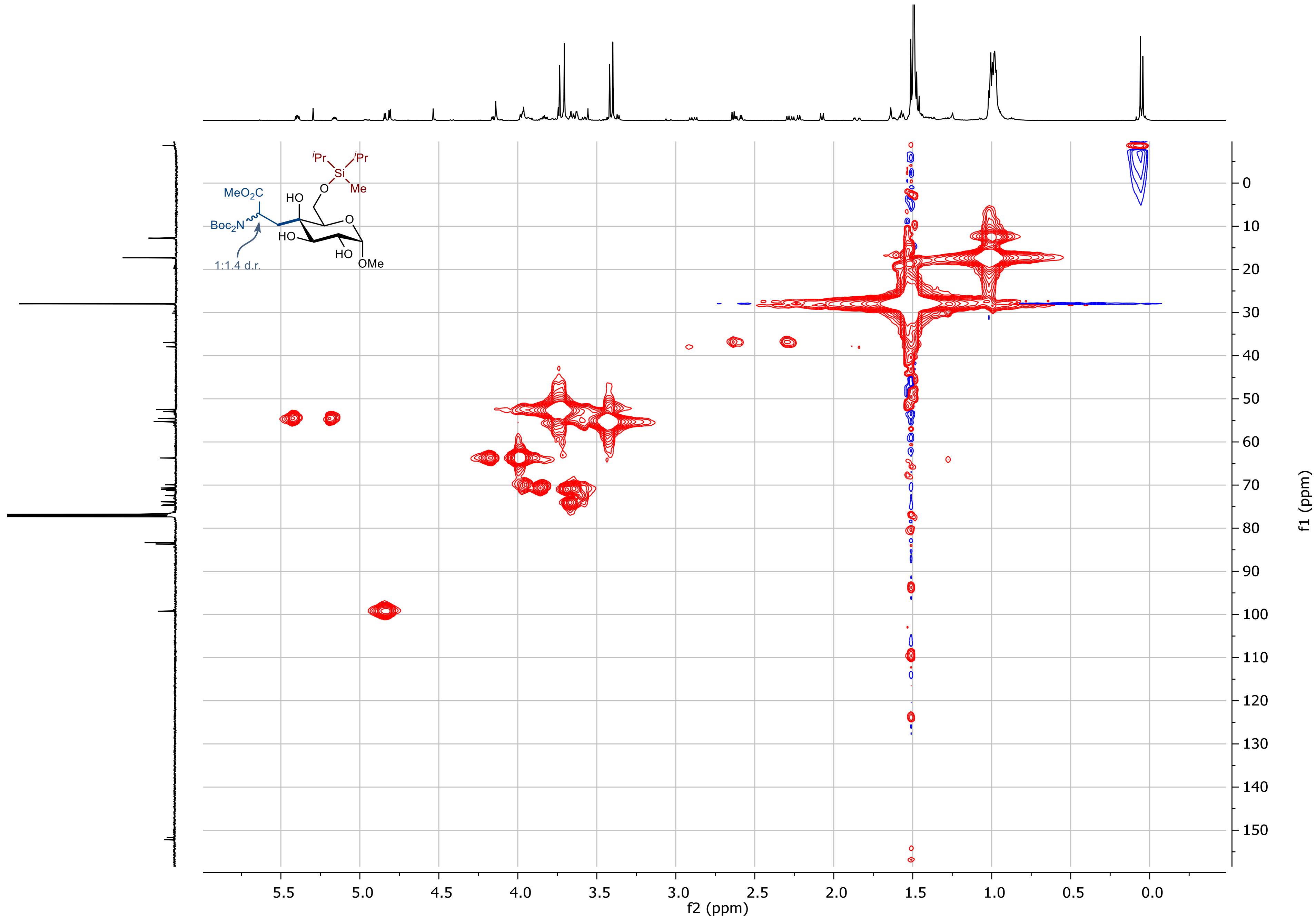

^{1}H NMR (500 MHz, $CDCl_3$) of compound **3r**

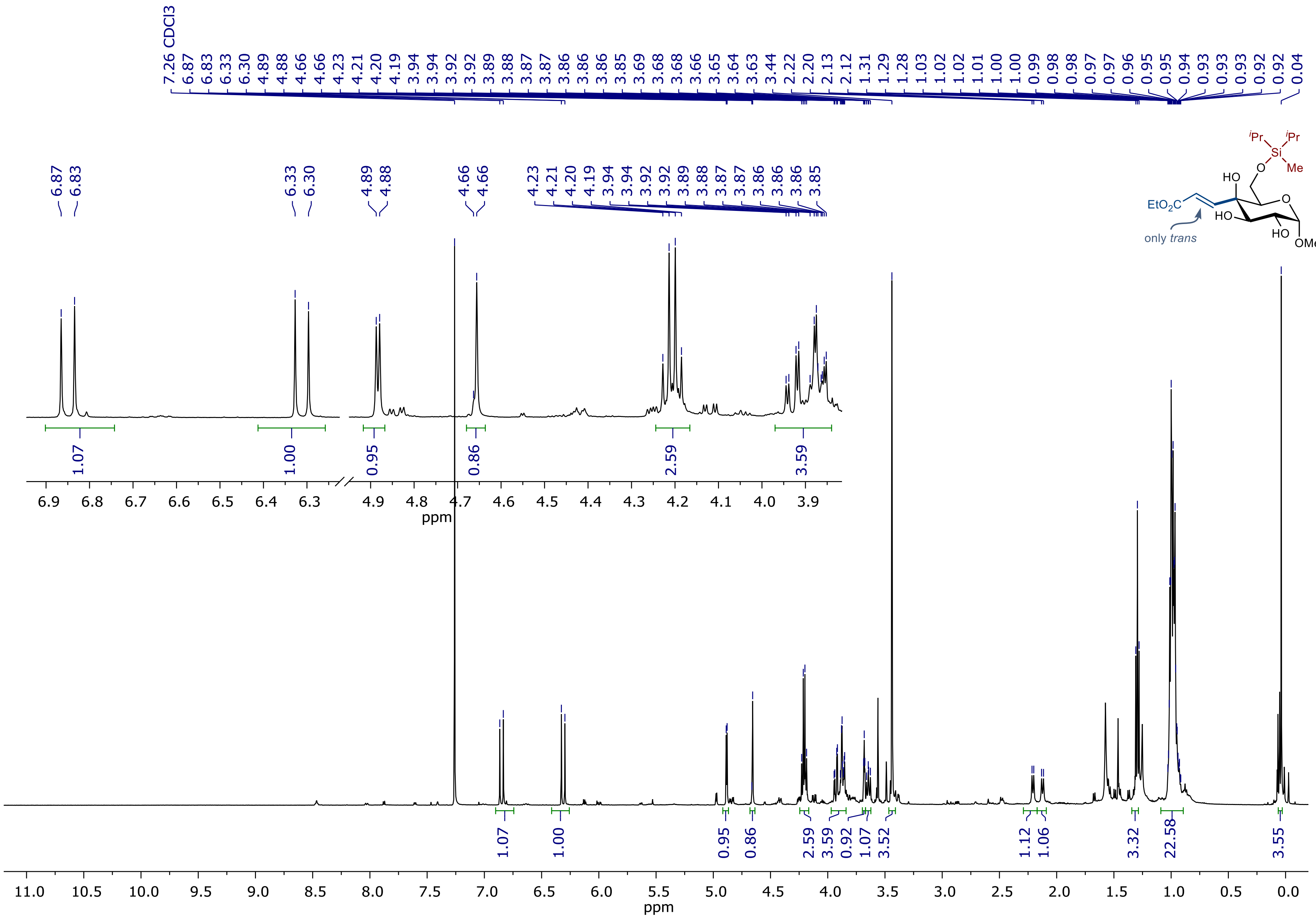

^{13}C{^{1}H} NMR (126 MHz, $CDCl_3$) of compound **3r**

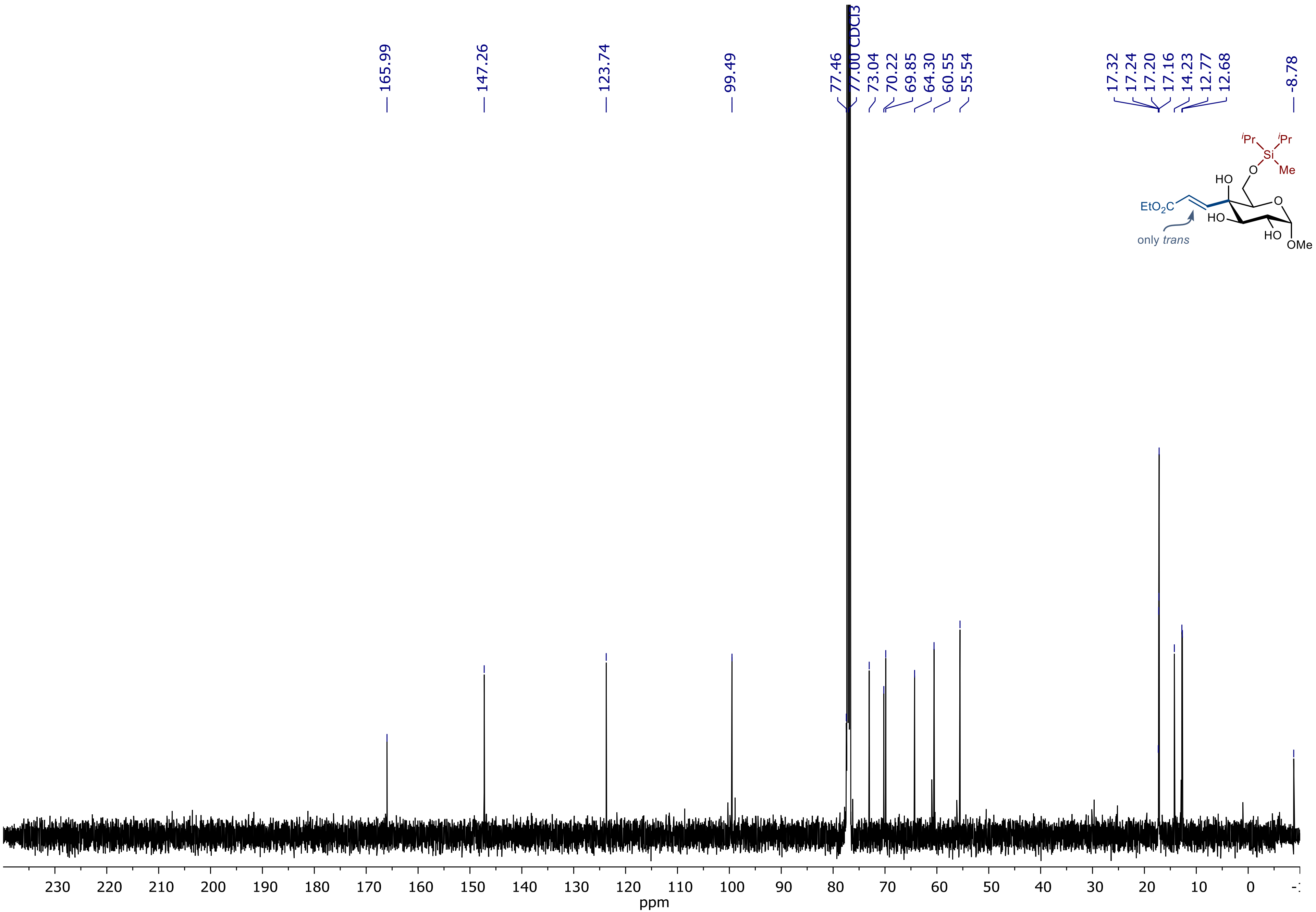

COSY of compound **3r**

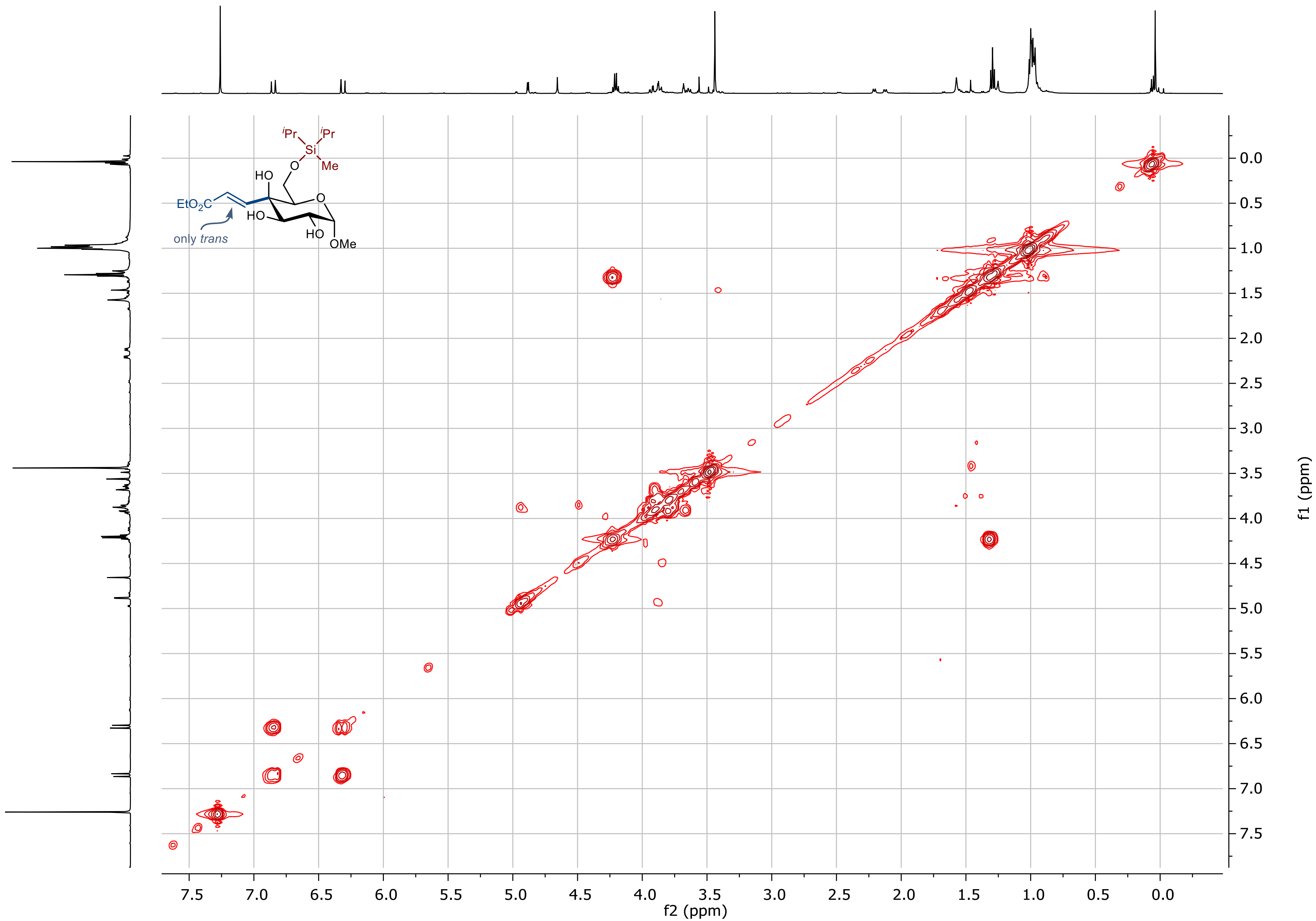

^{1}H NMR (500 MHz, $CDCl_3$) of compound **3s**

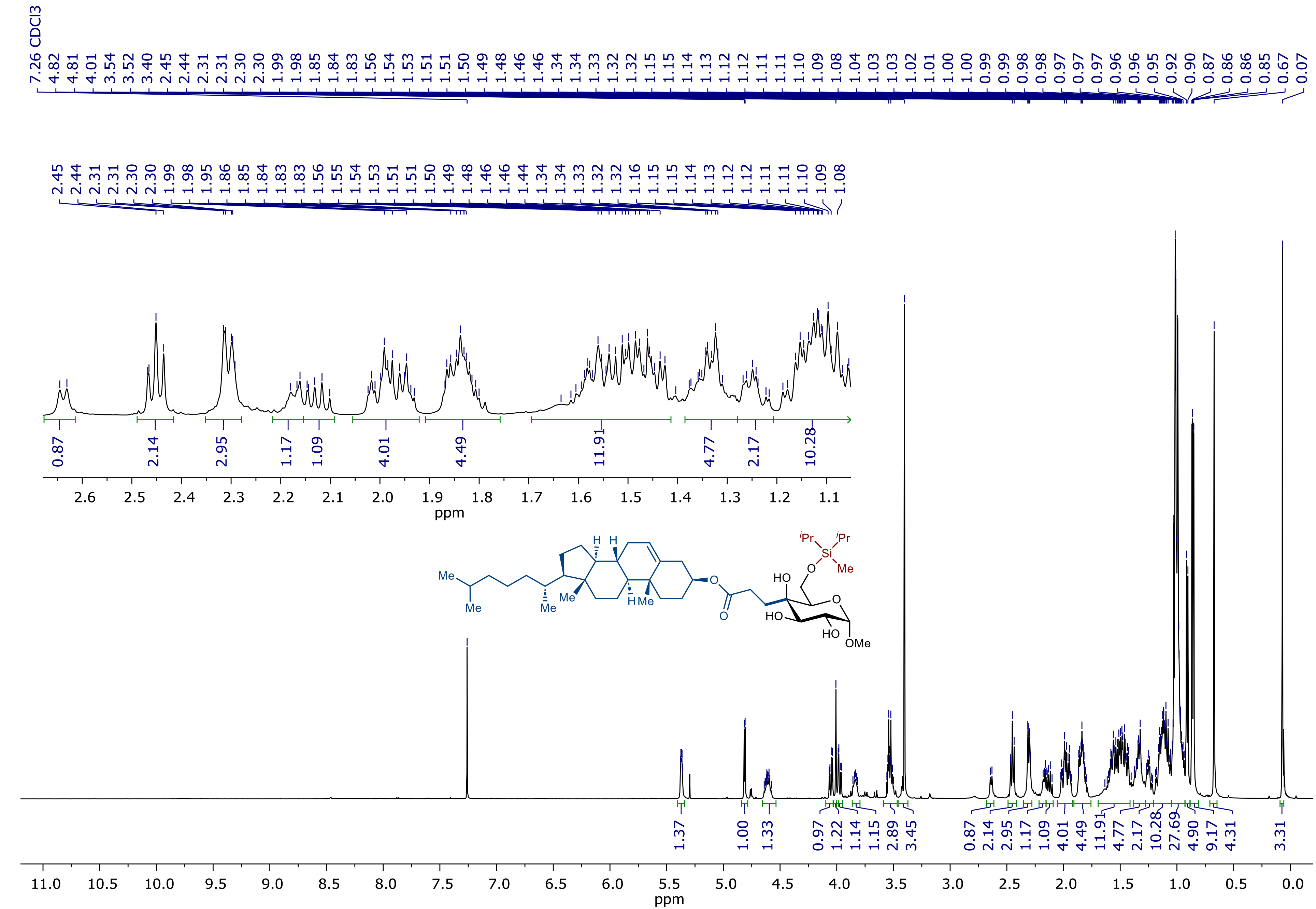

$^{13}C\{^{1}H\}$ **NMR** (126 MHz, $CDCl_3$) of compound **3s**

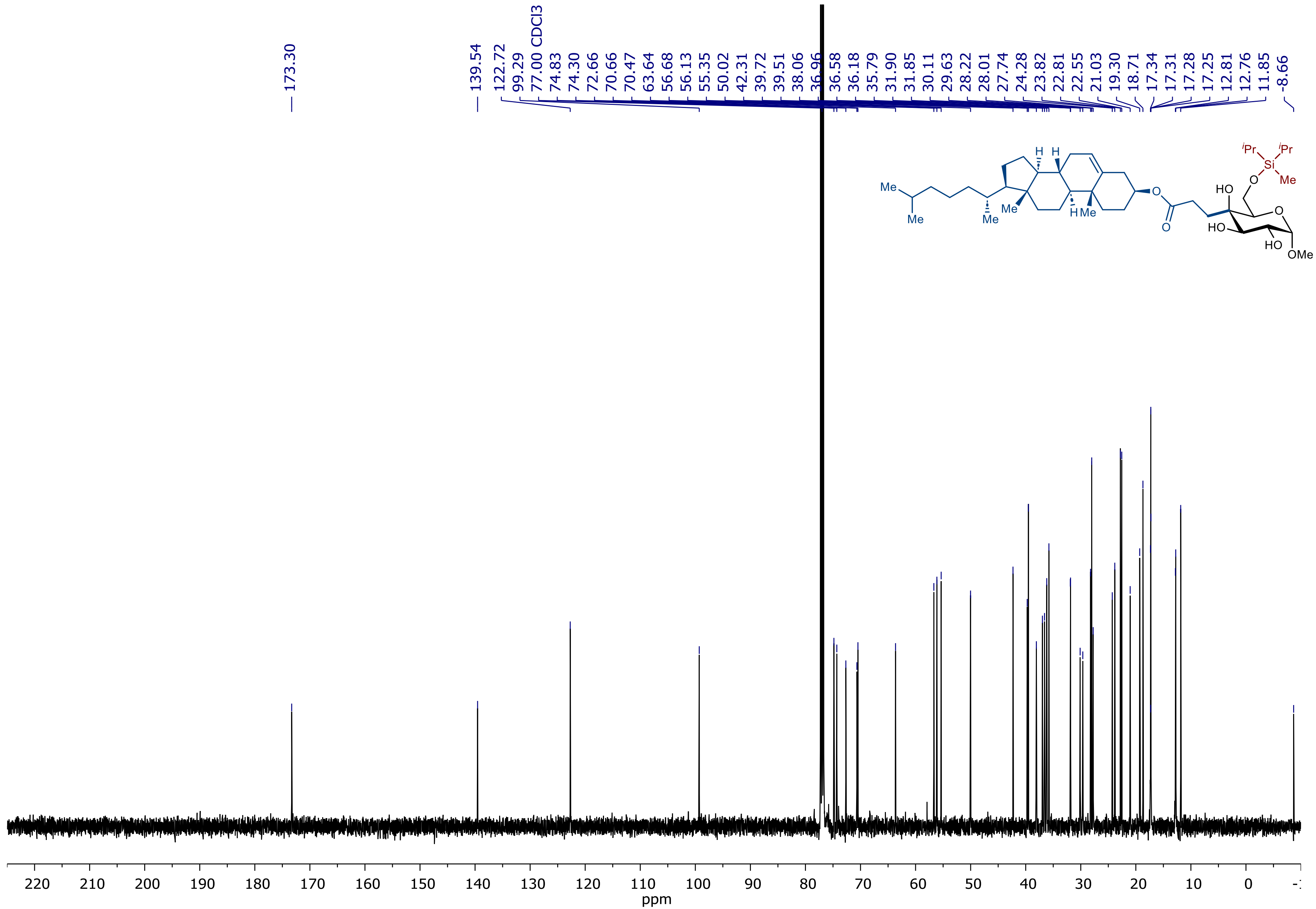

COSY of compound **3s**

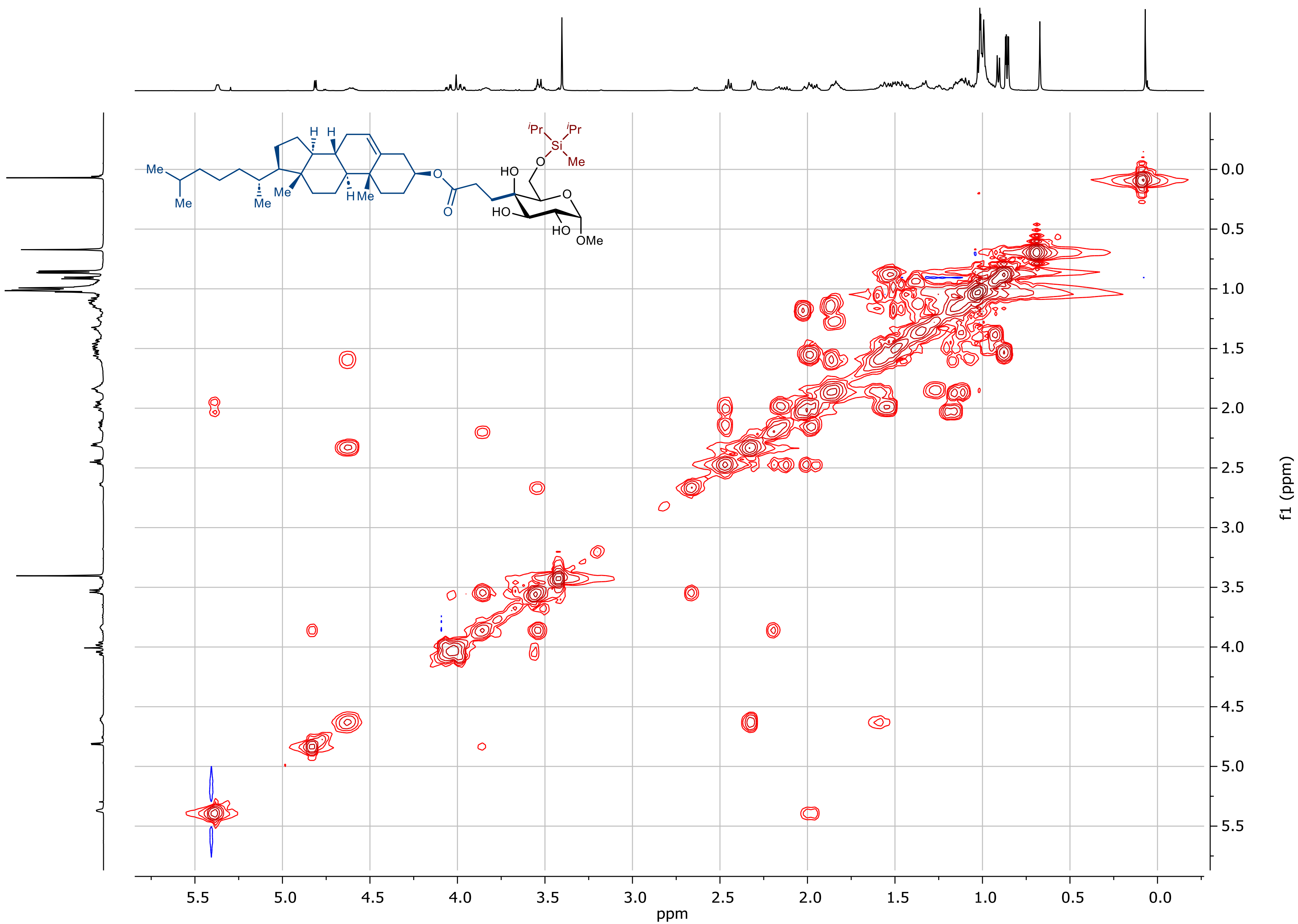

HSQC of compound **3s**

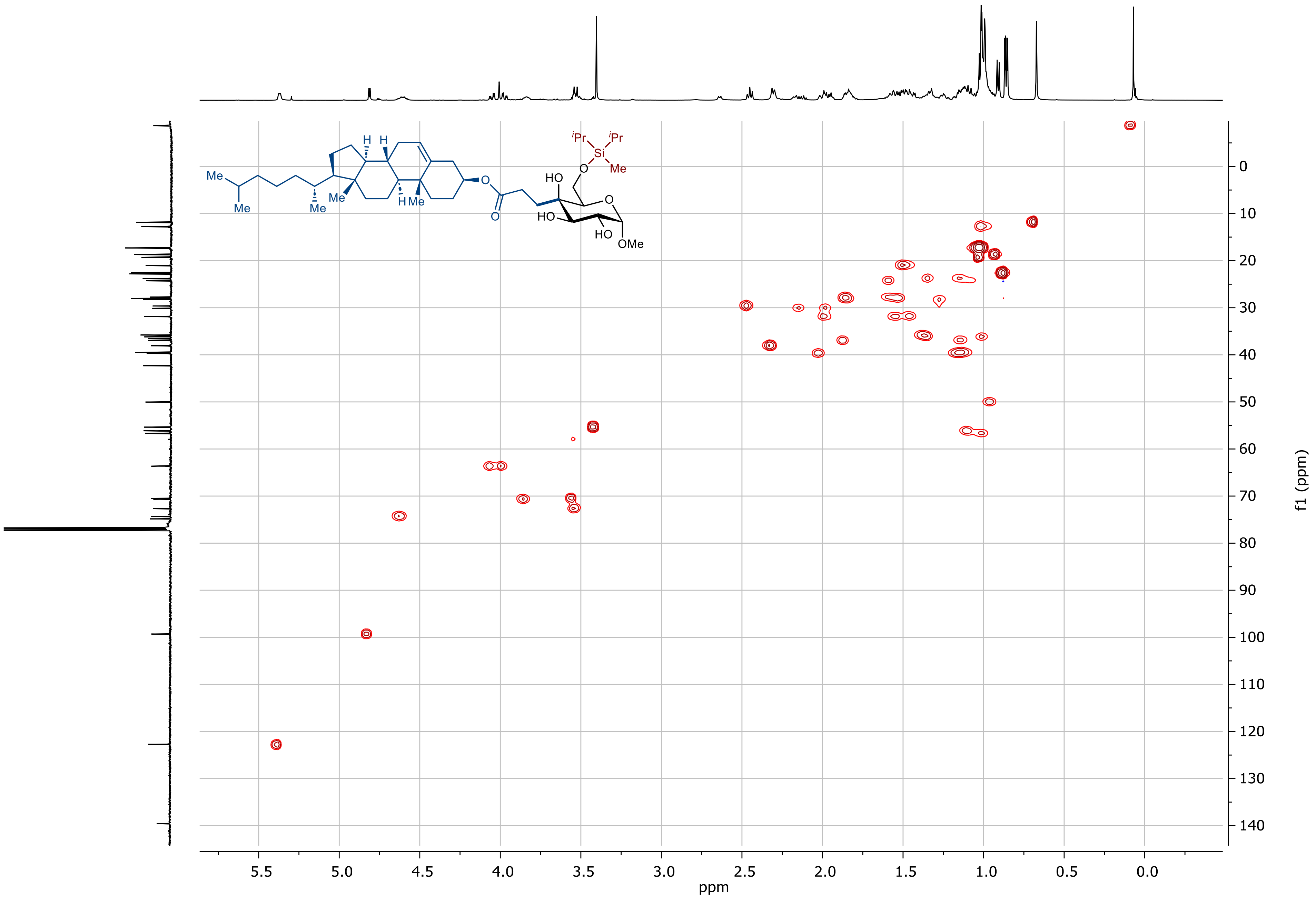

^{1}H NMR (500 MHz, $CDCl_3$) of compound **3t**

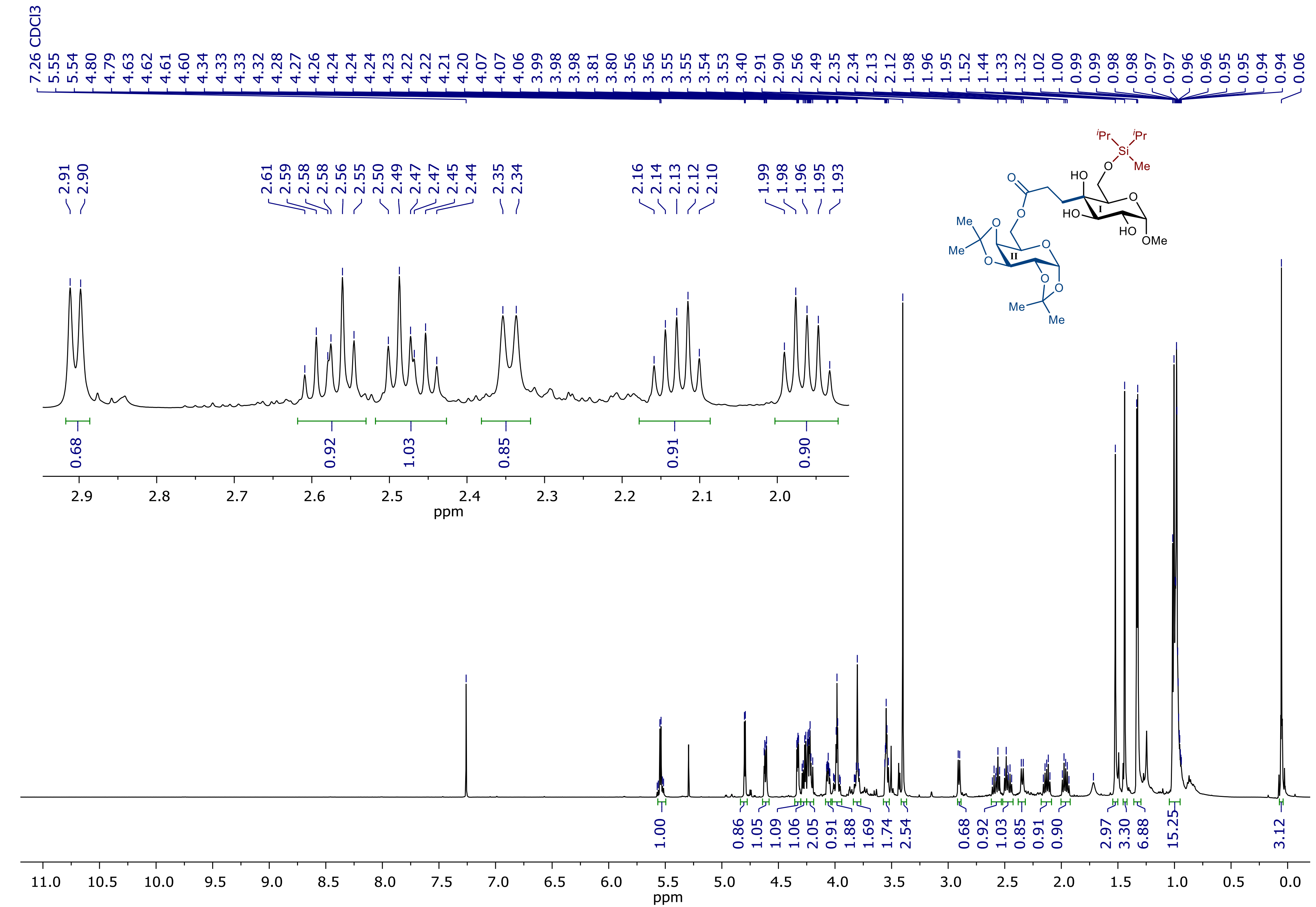

$^{13}C\{^1H\}$ NMR (126 MHz, $CDCl_3$) of compound **3t**

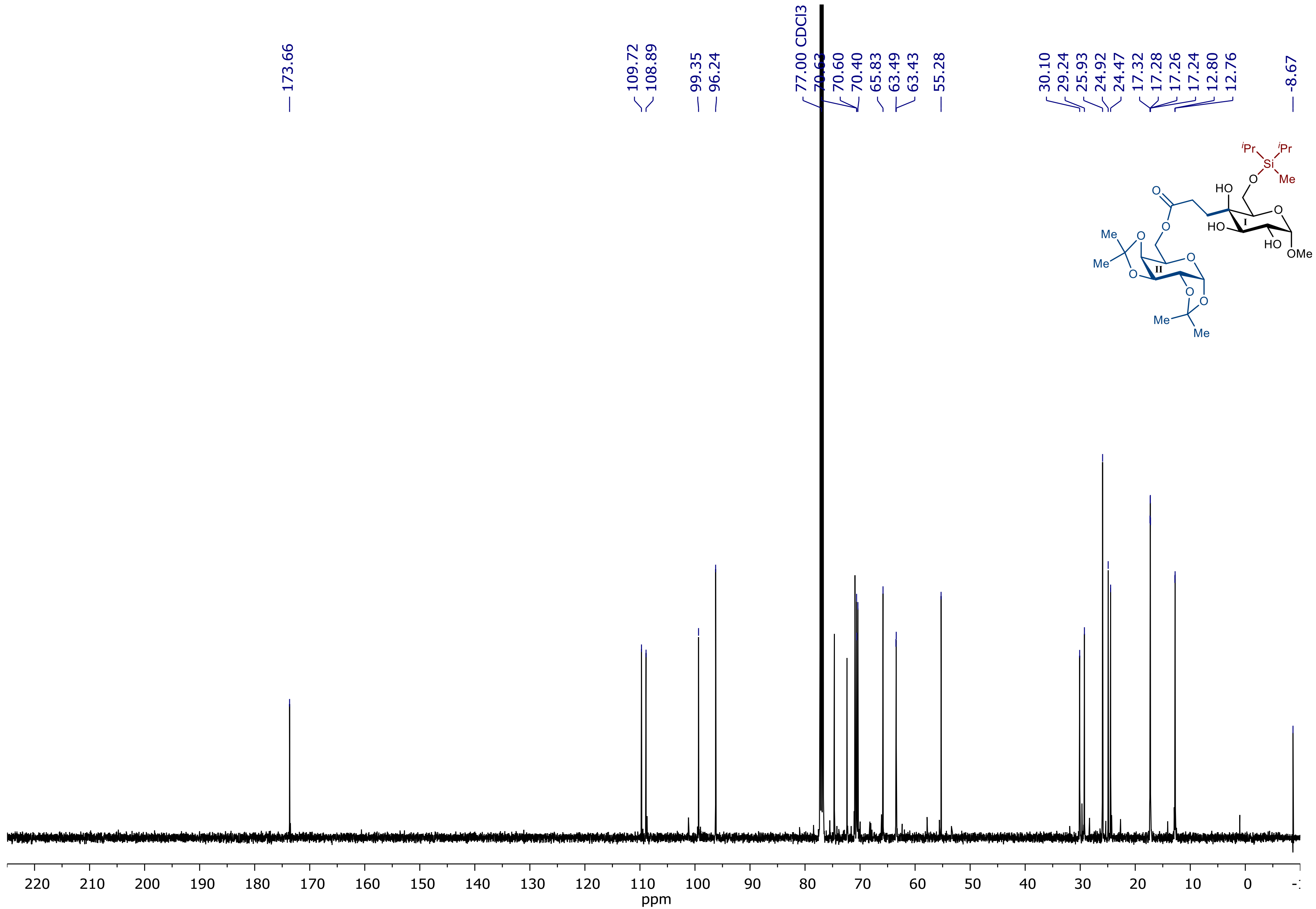

COSY of compound **3t**

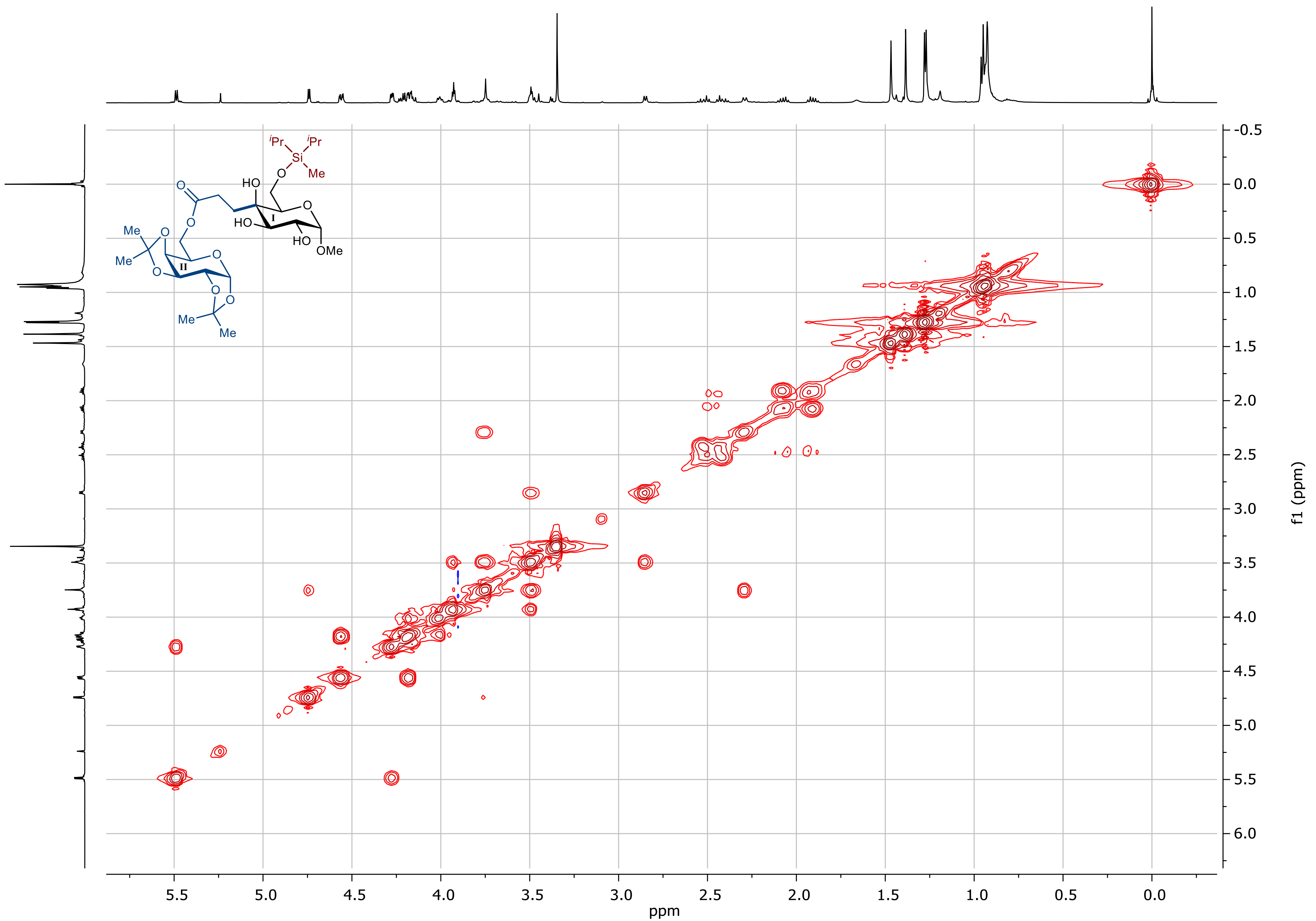

HSQC of compound **3t**

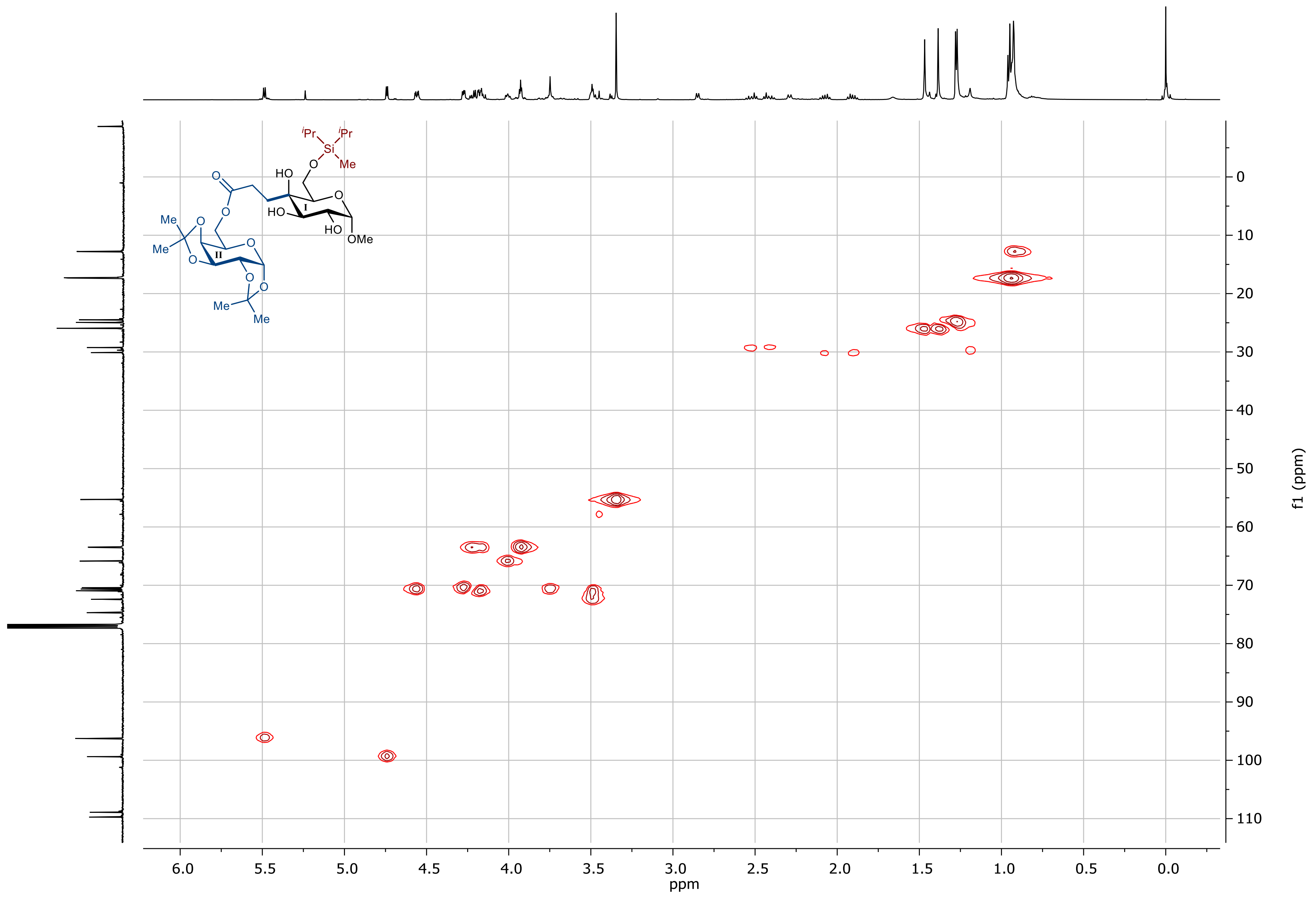

¹H NMR (500 MHz, $CDCl_3$) of compound **3u**

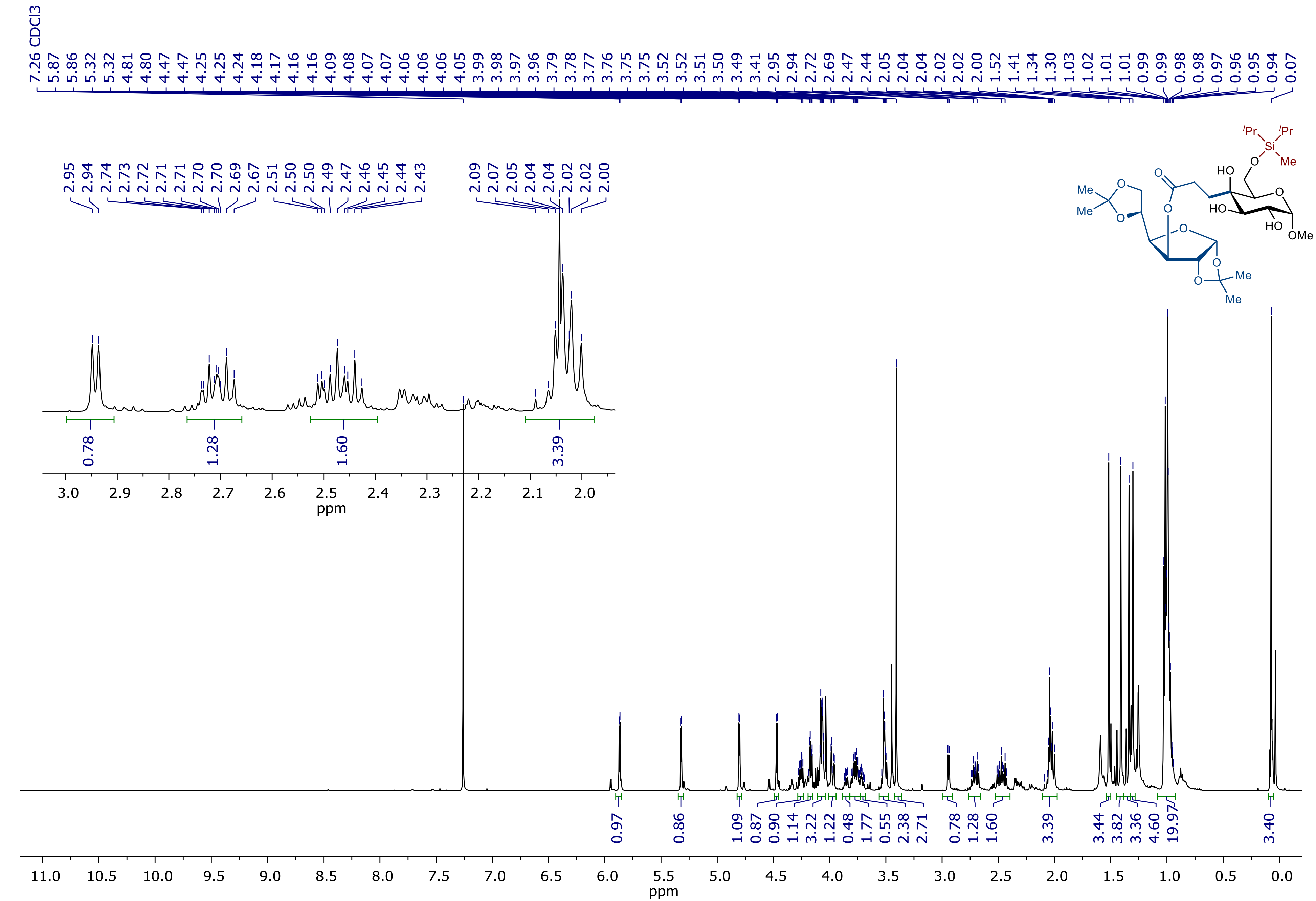

$^{13}C\{^1H\}$ NMR (126 MHz, $CDCl_3$) of compound **3u**

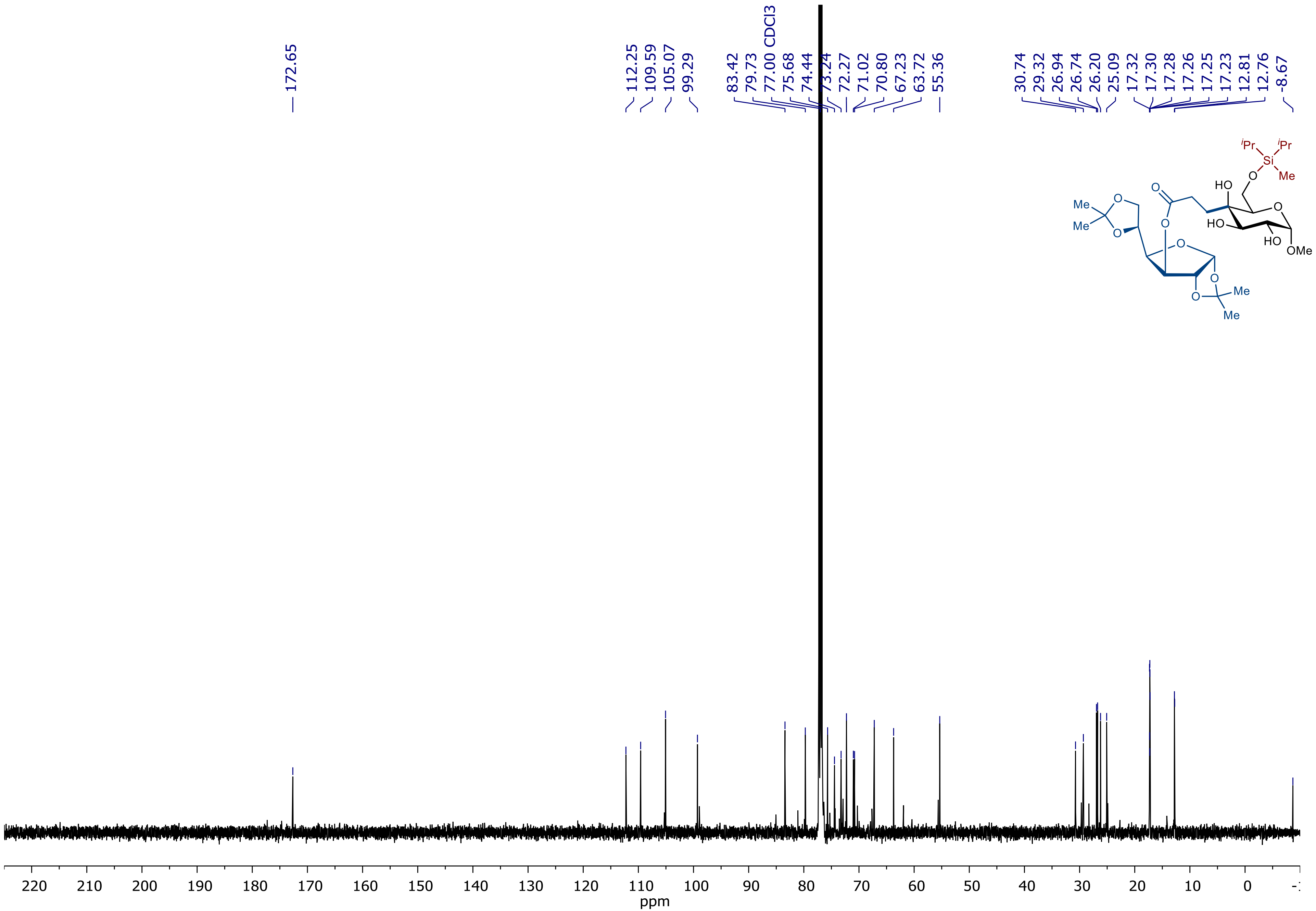

COSY of compound **3u**

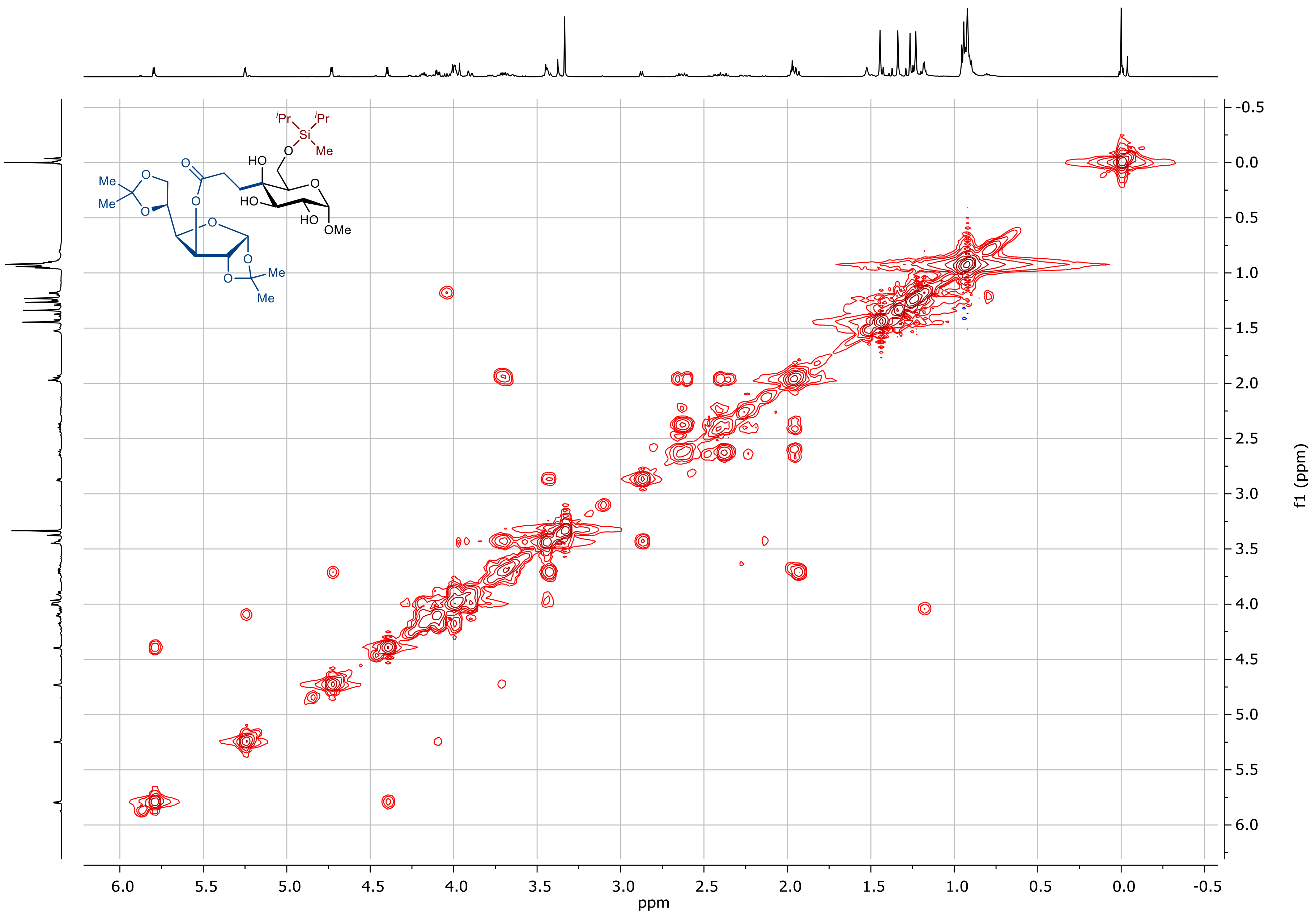

HSQC of compound **3u**

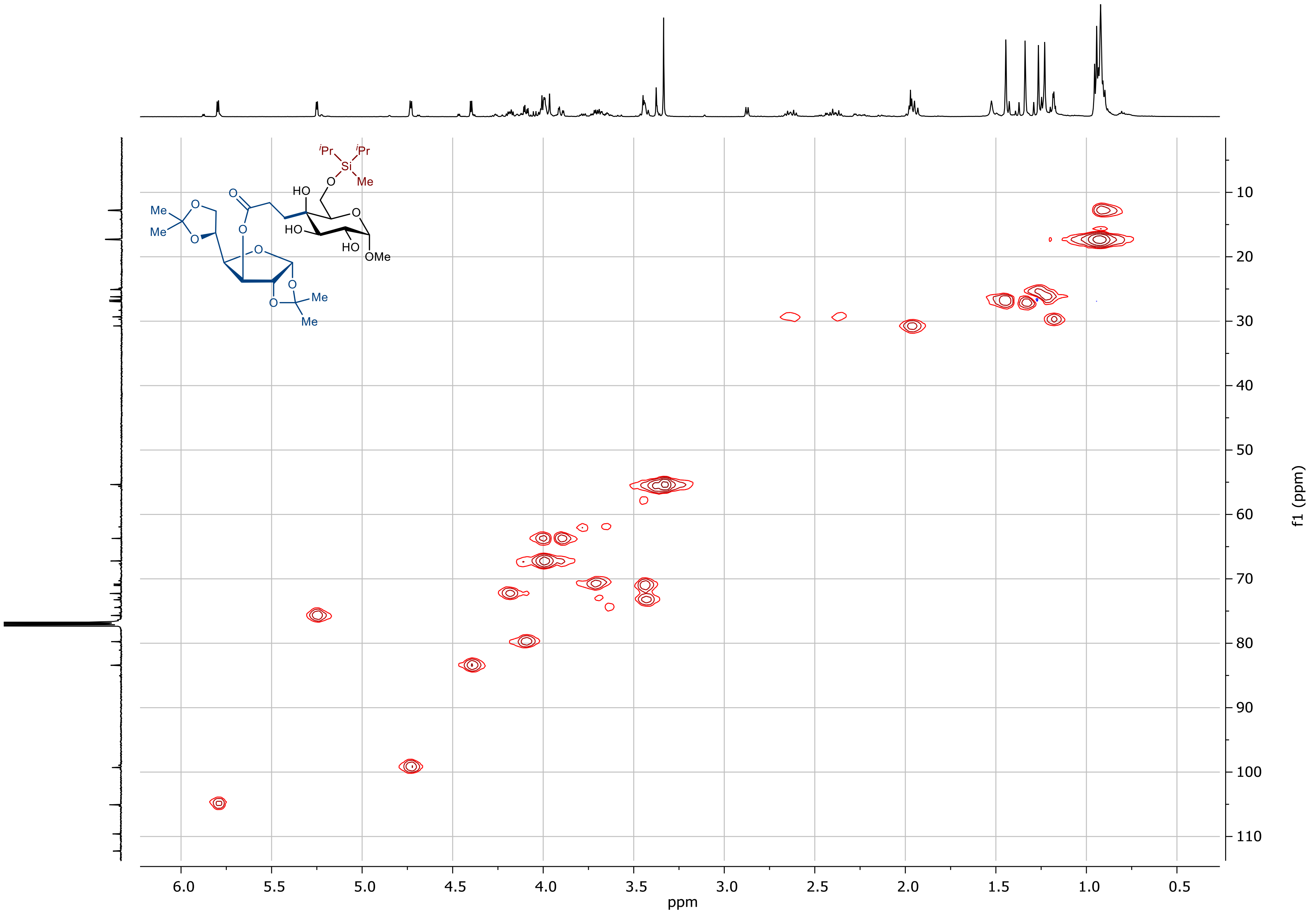

^{1}H NMR (500 MHz, $CDCl_3$) of compound **3v**

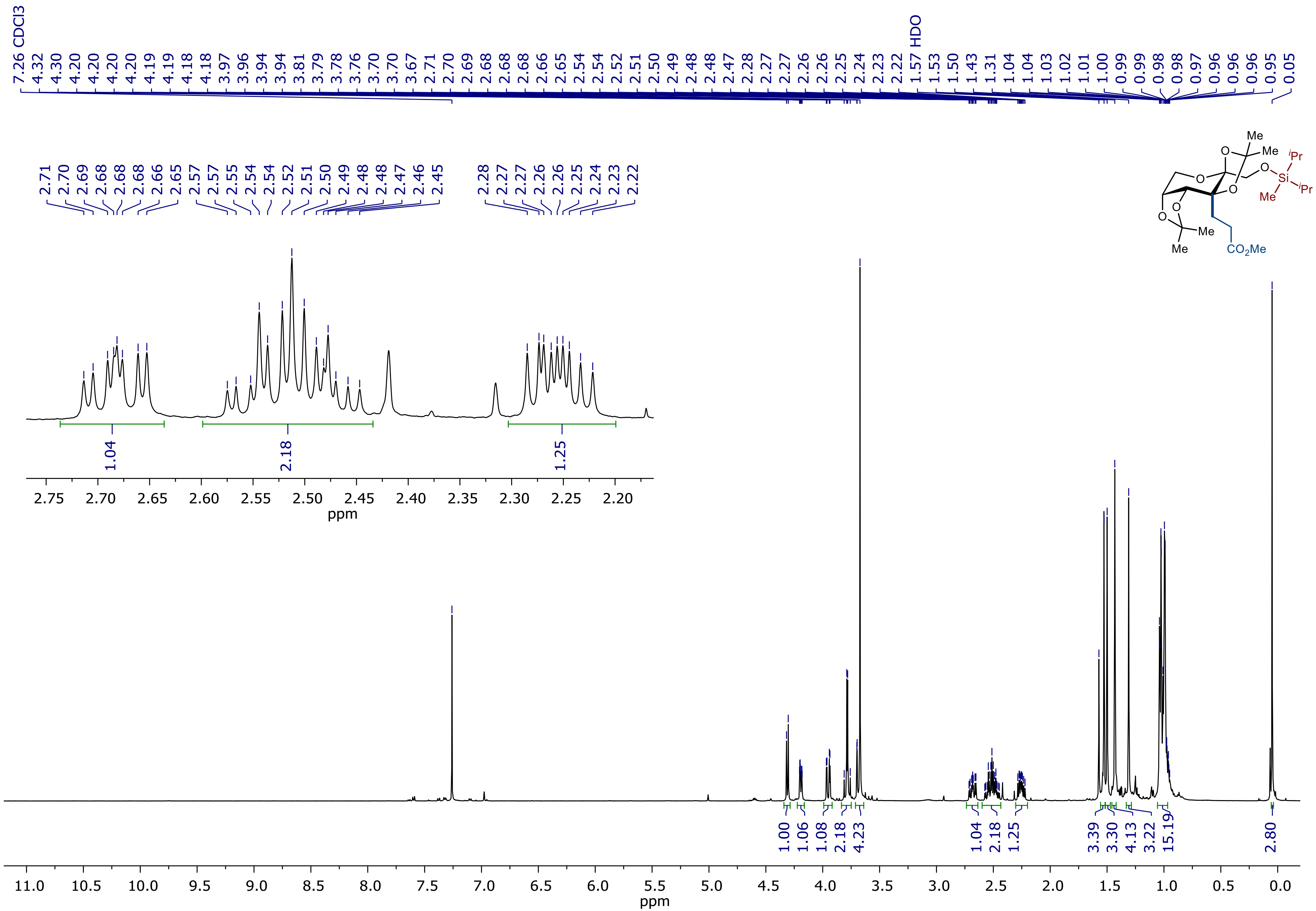

^{13}C{^{1}H} NMR (126 MHz, $CDCl_3$) of compound **3v**

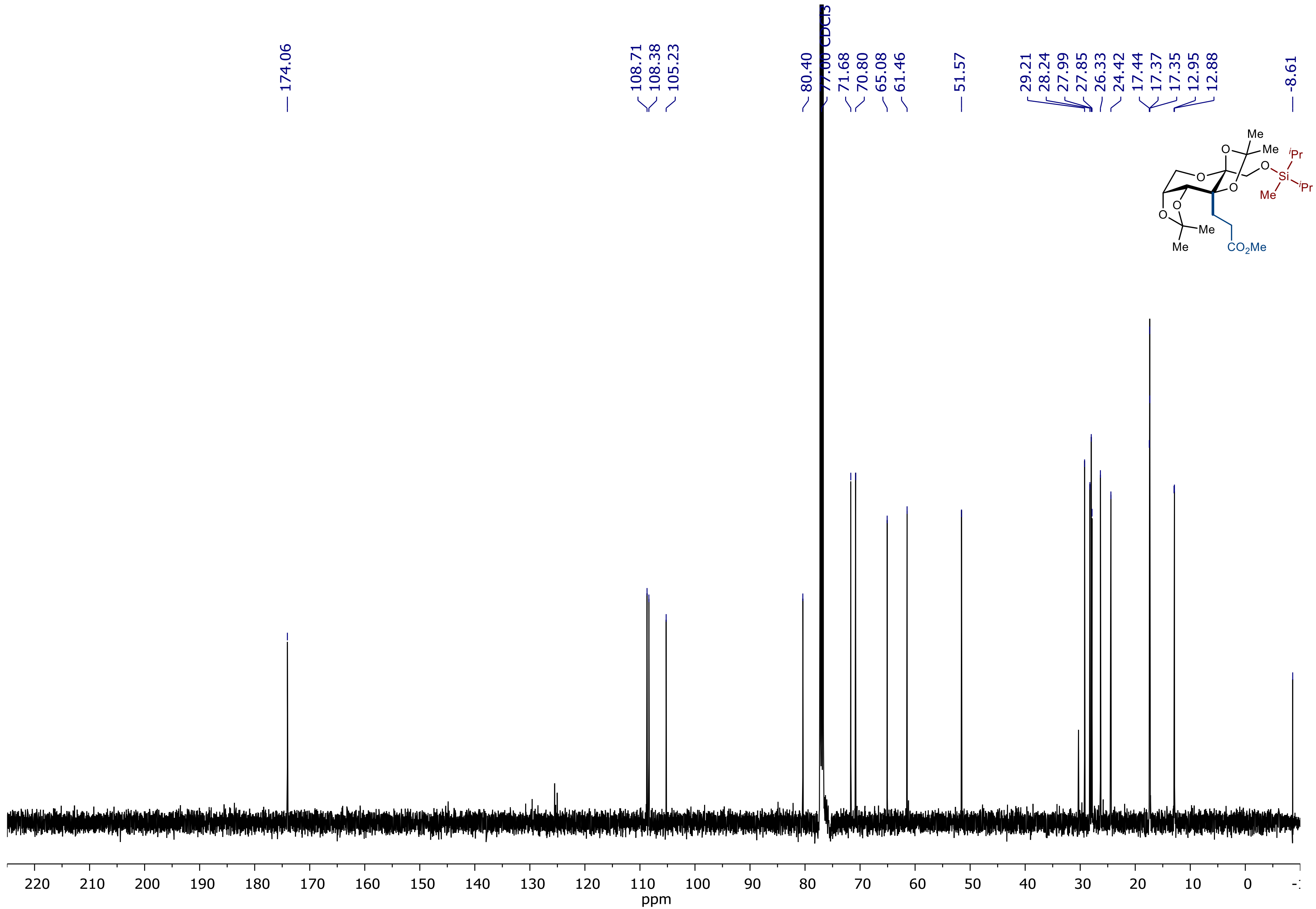

COSY of compound **3v**

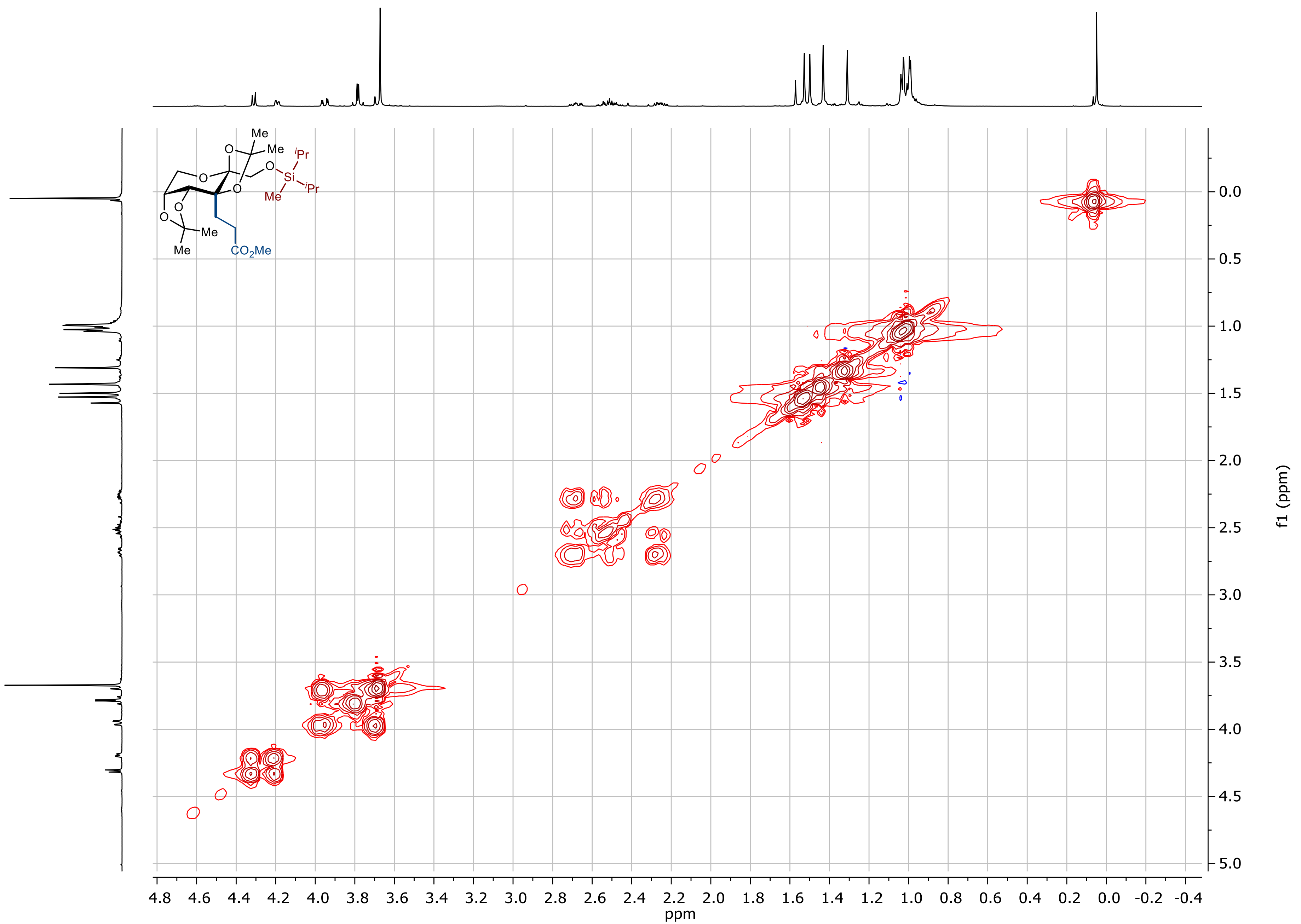

HSQC of compound **3v**

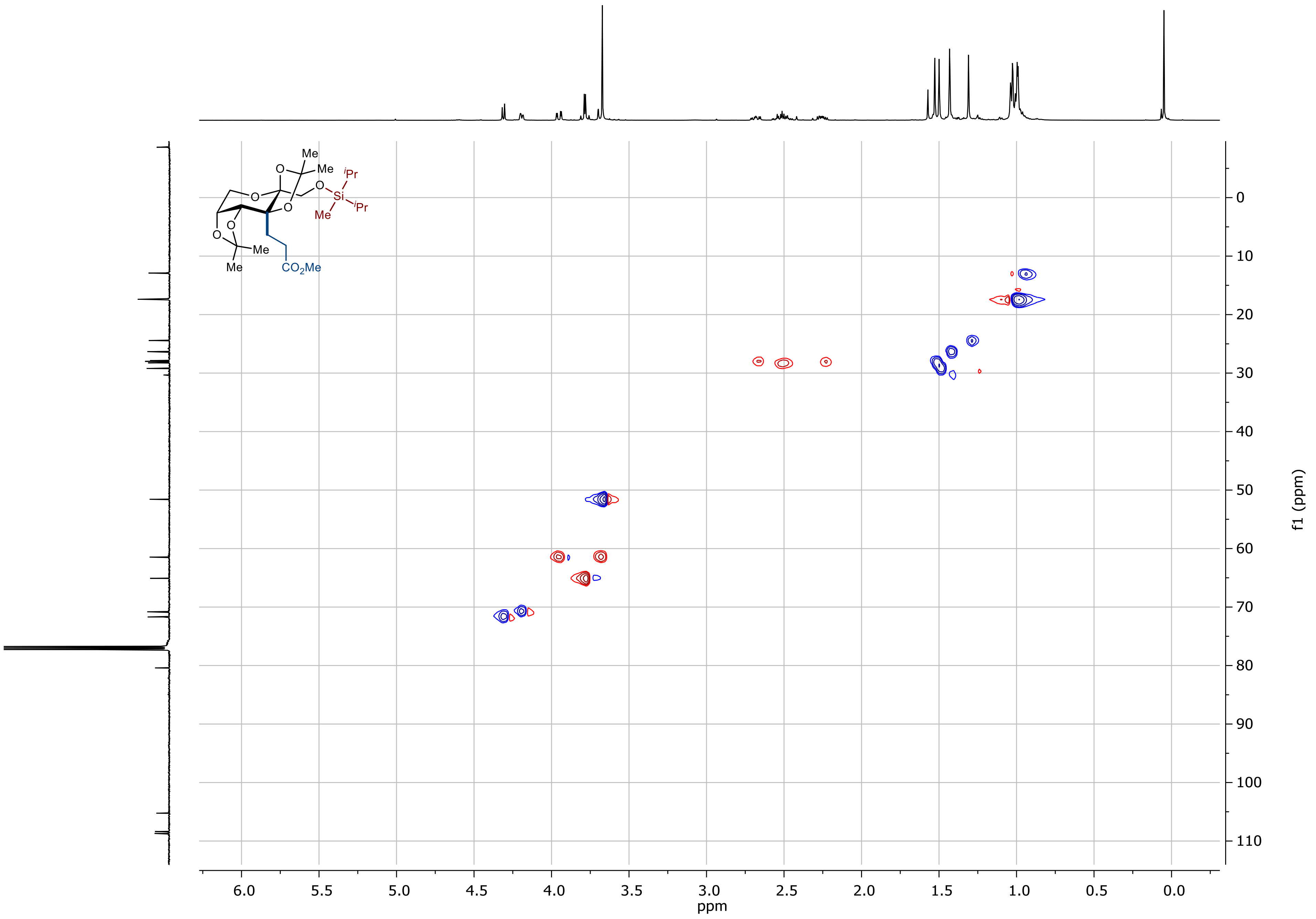

^{1}H NMR (500 MHz, $CDCl_3$) of compound **3w**

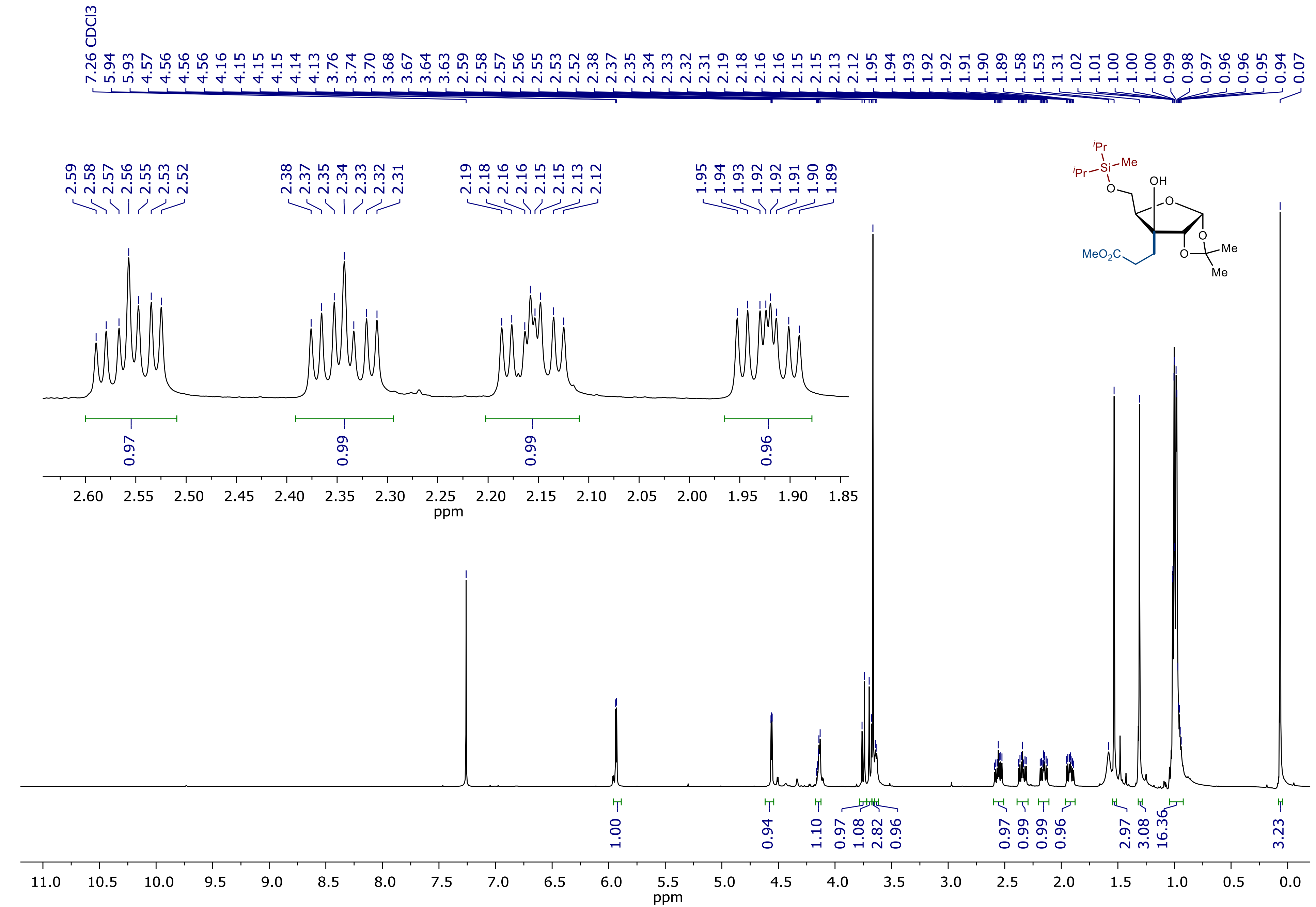

$^{13}C\{^1H\}$ NMR (126 MHz, $CDCl_3$) of compound **3w**

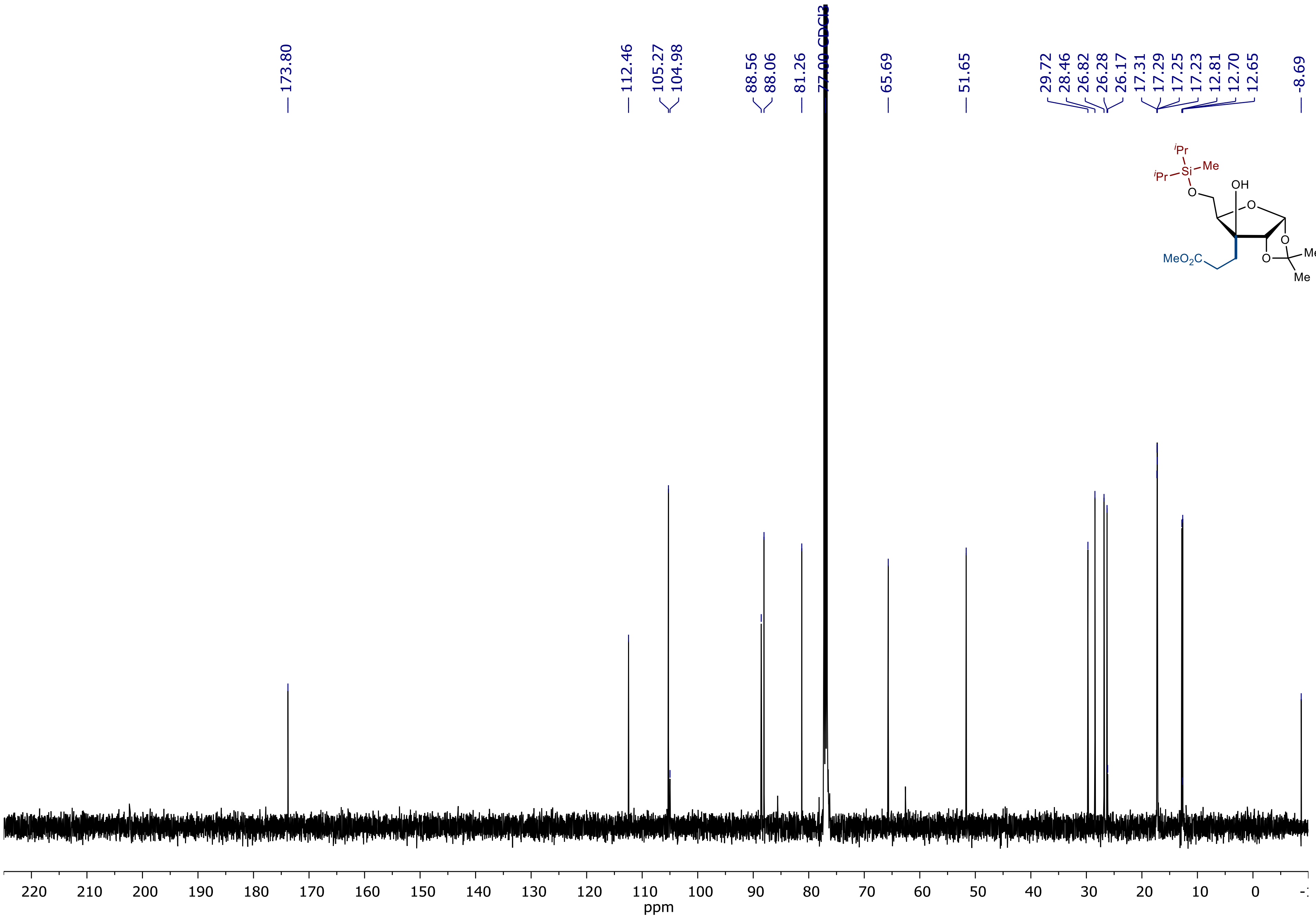

COSY of compound **3w**

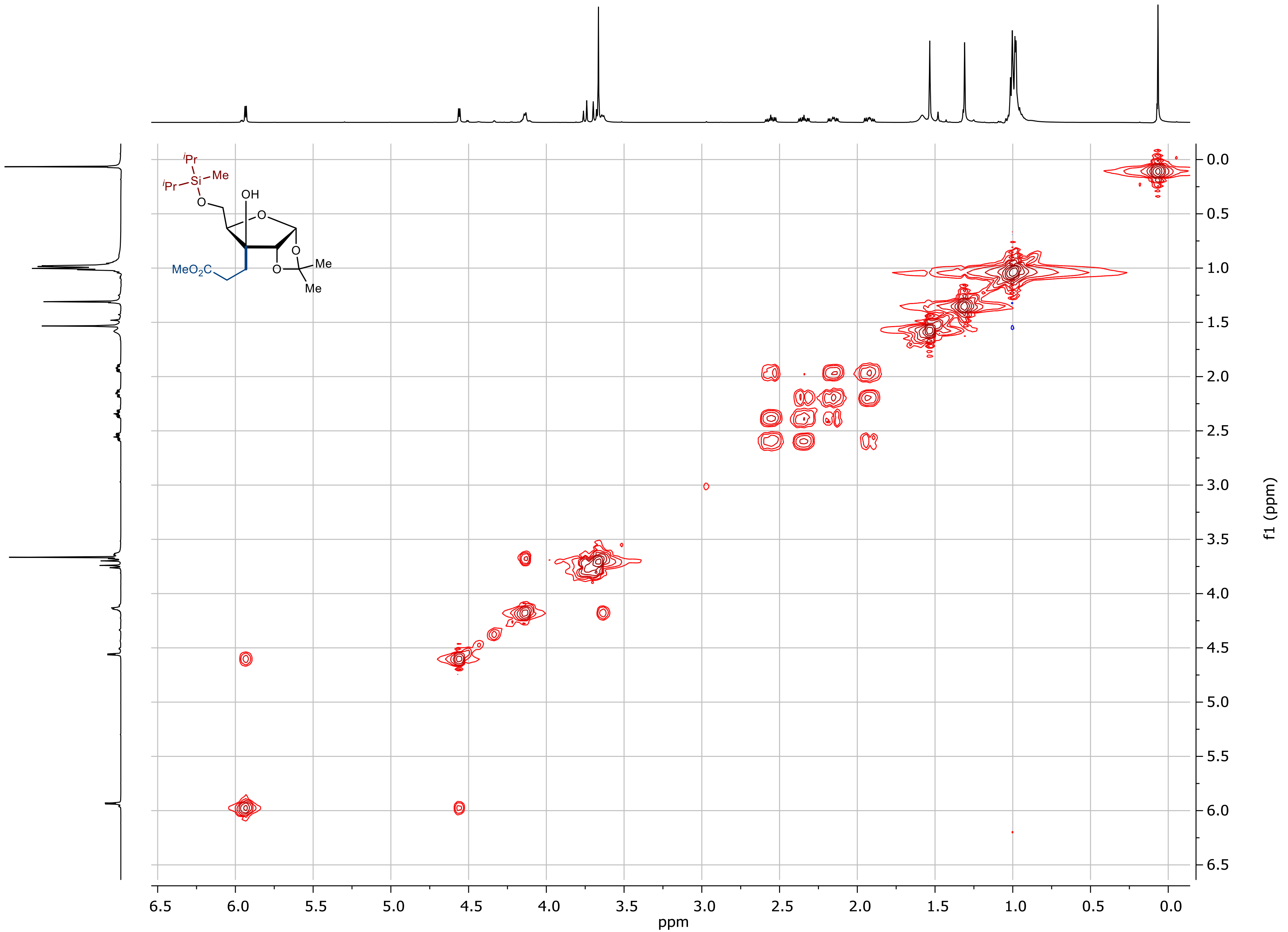

HSQC of compound **3w**

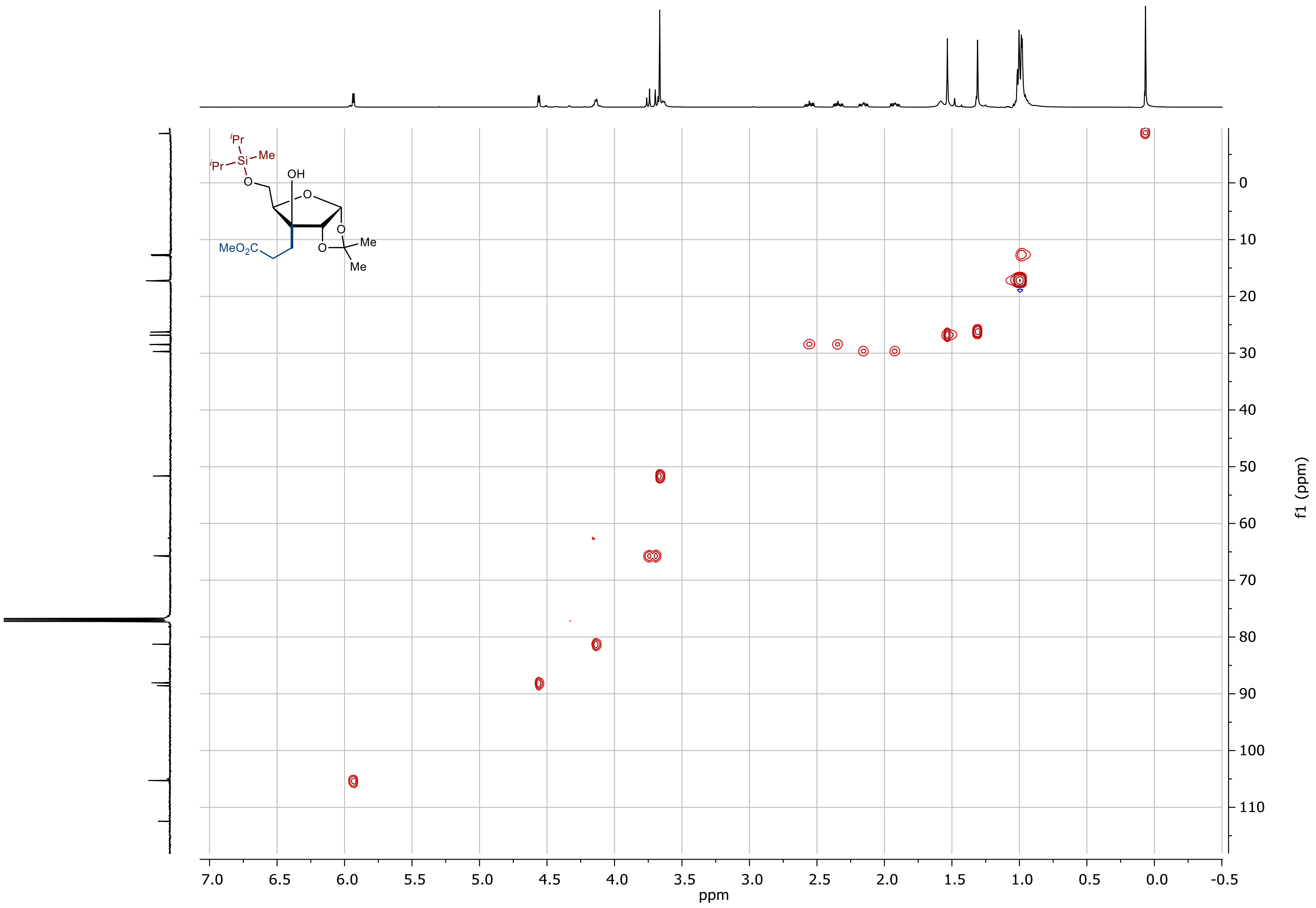

NOESY of compound **3w**

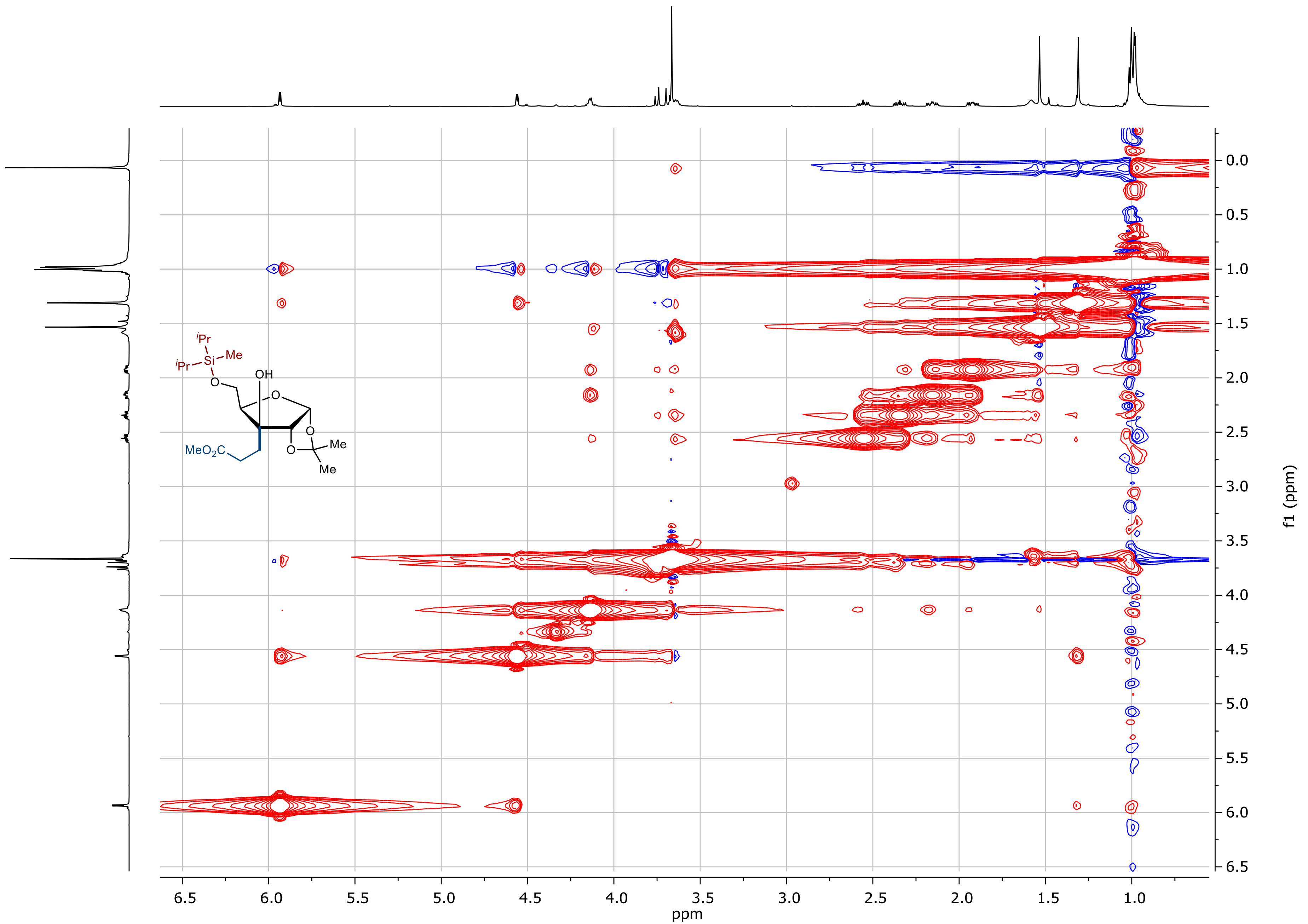

^{1}H NMR (500 MHz, $CDCl_3$) of compound **3w’**

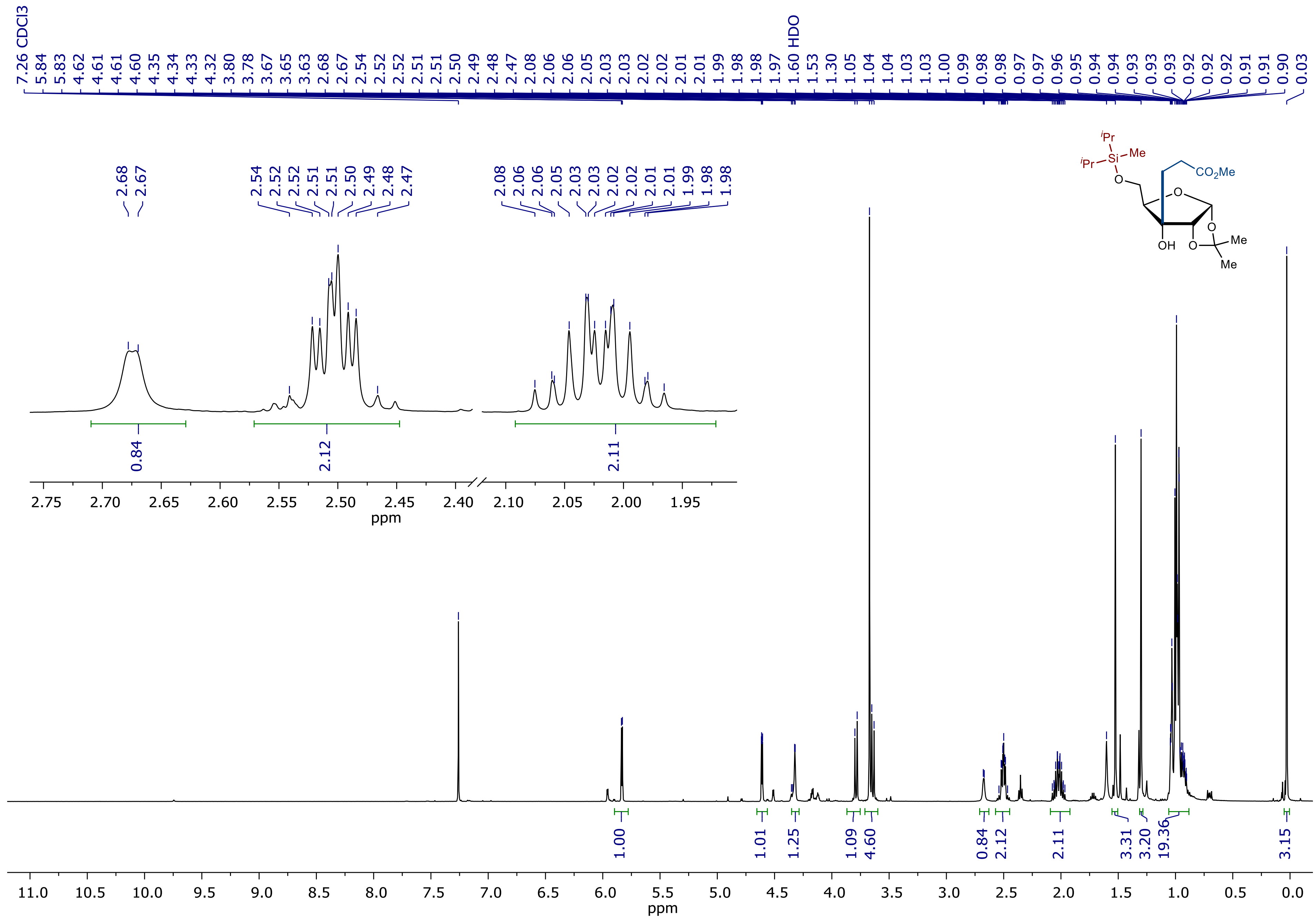

$^{13}C\{^{1}H\}$ NMR (126 MHz, $CDCl_3$) of compound **3w'**

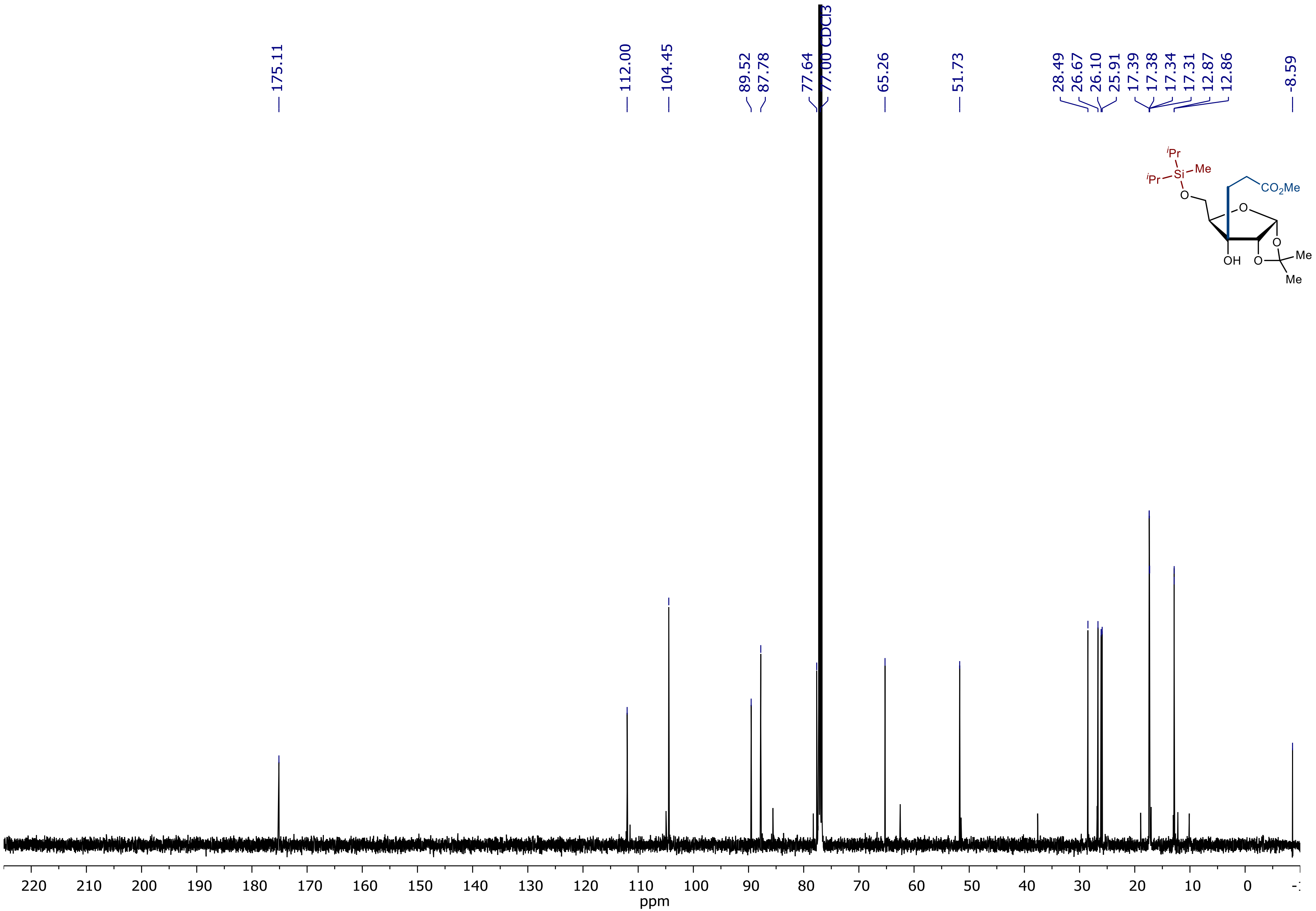

COSY of compound **3w'**

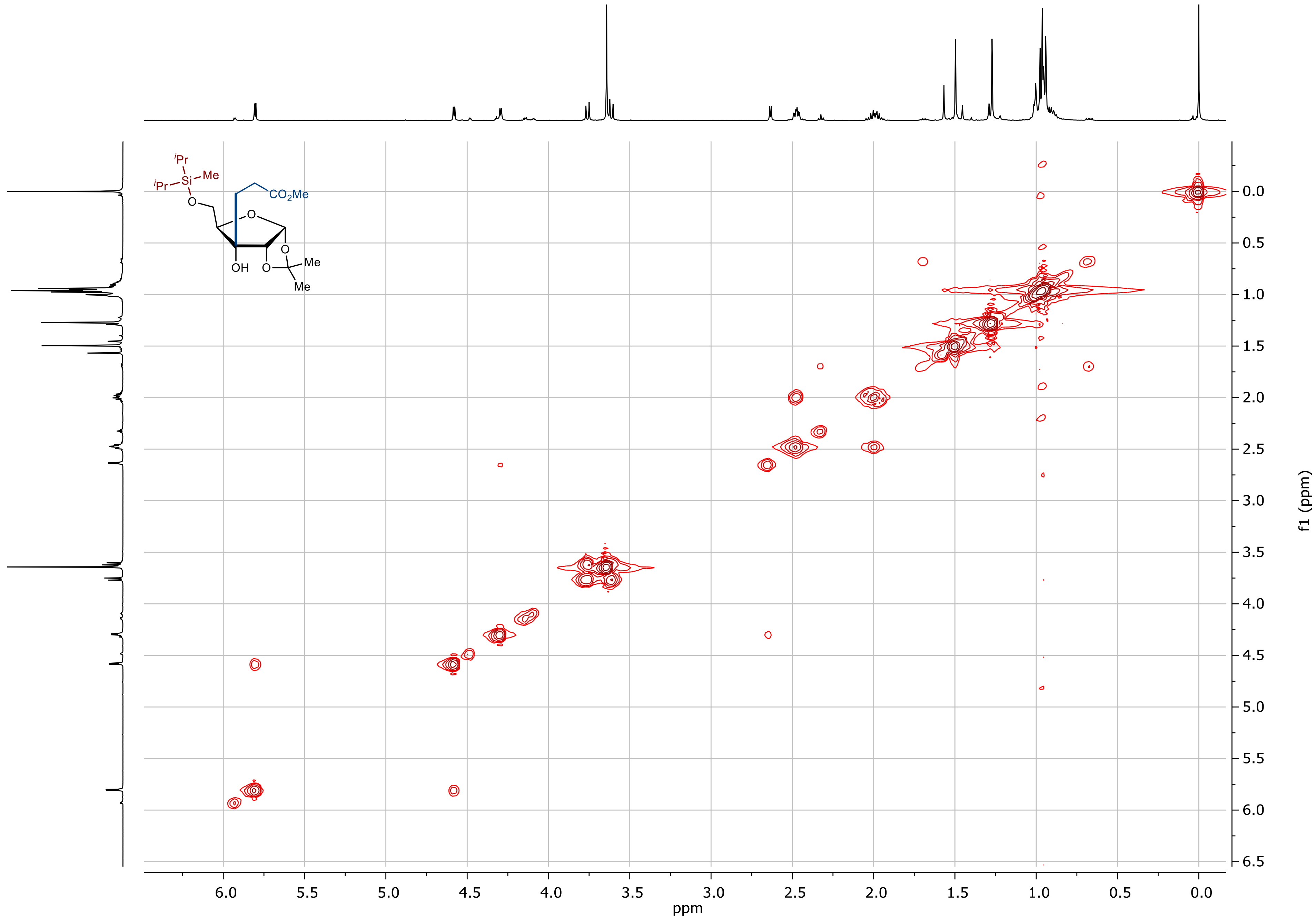

HSQC of compound **3w'**

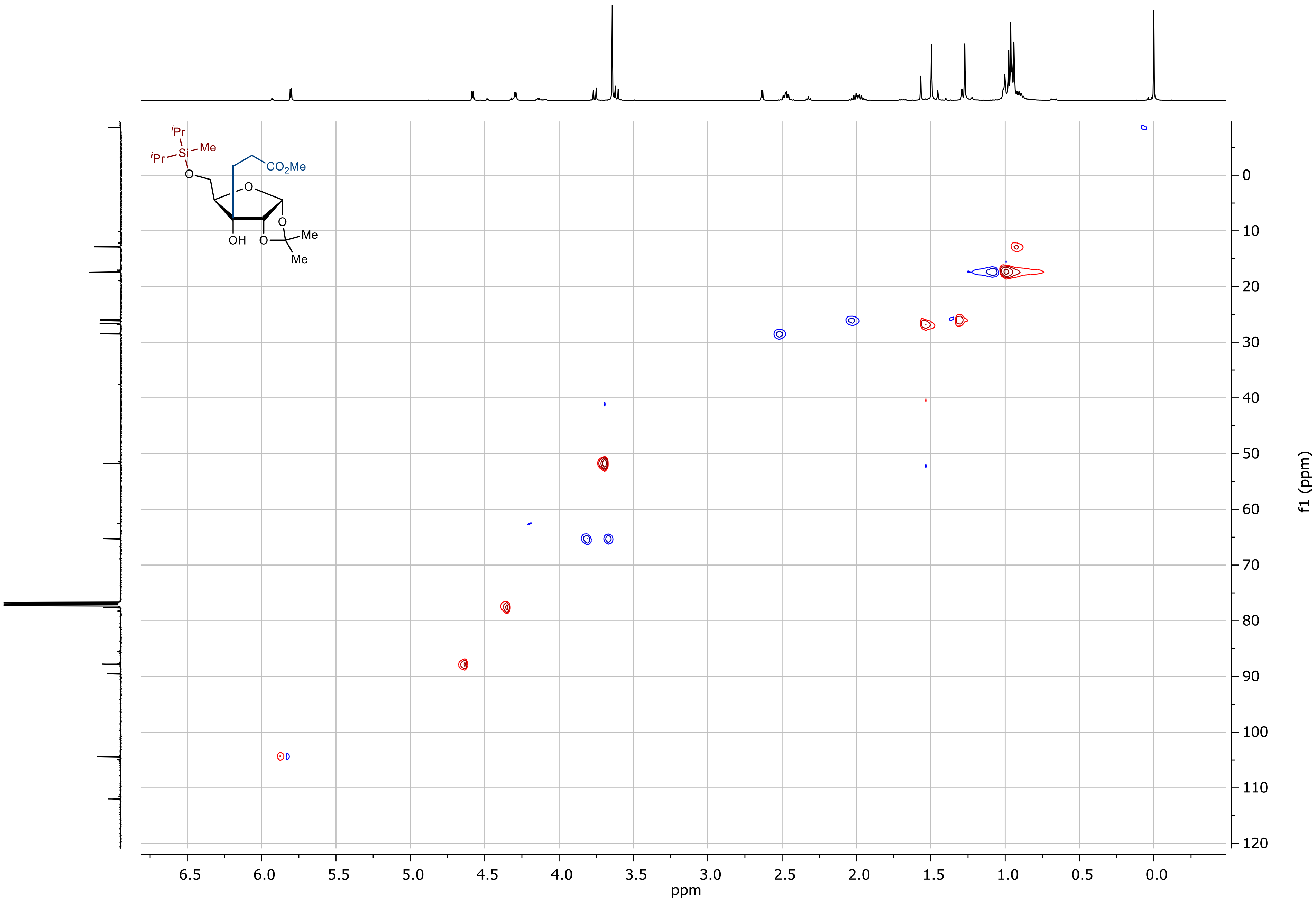

^{1}H NMR (500 MHz, $CDCl_3$) of compound **6a**

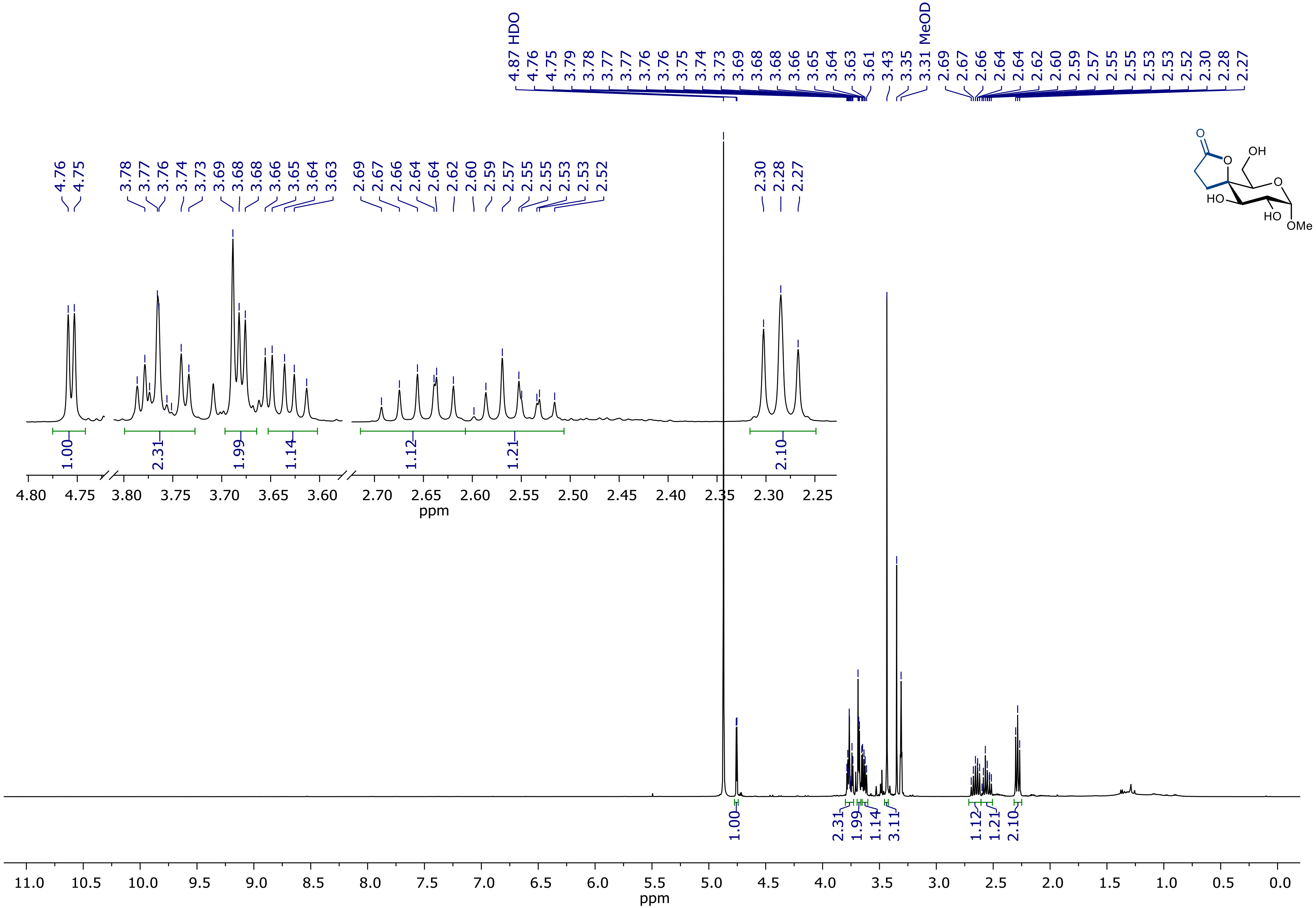

$^{13}C\{^{1}H\}$ NMR (126 MHz, $CDCl_3$) of compound **6a**

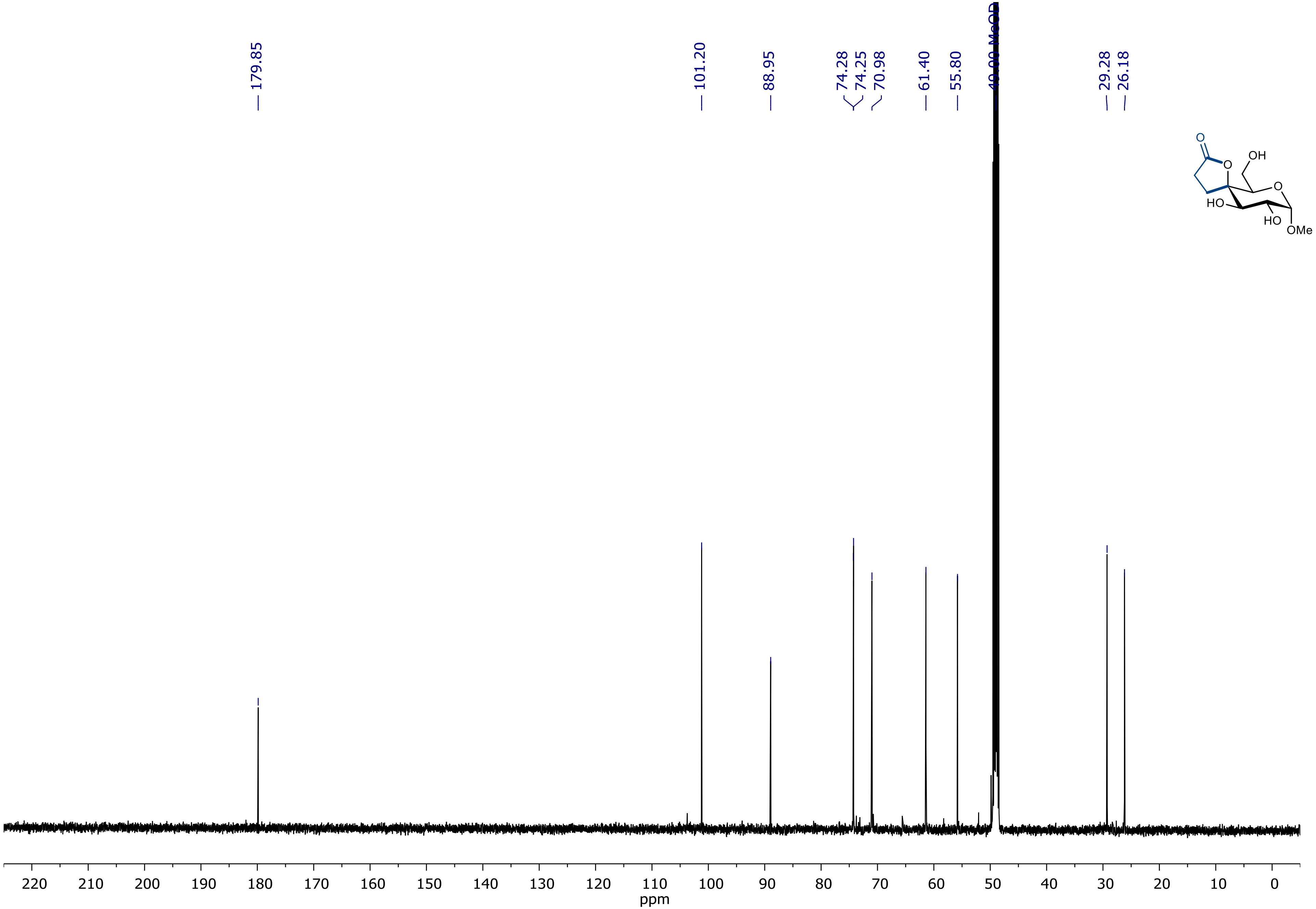

COSY of compound **6a**

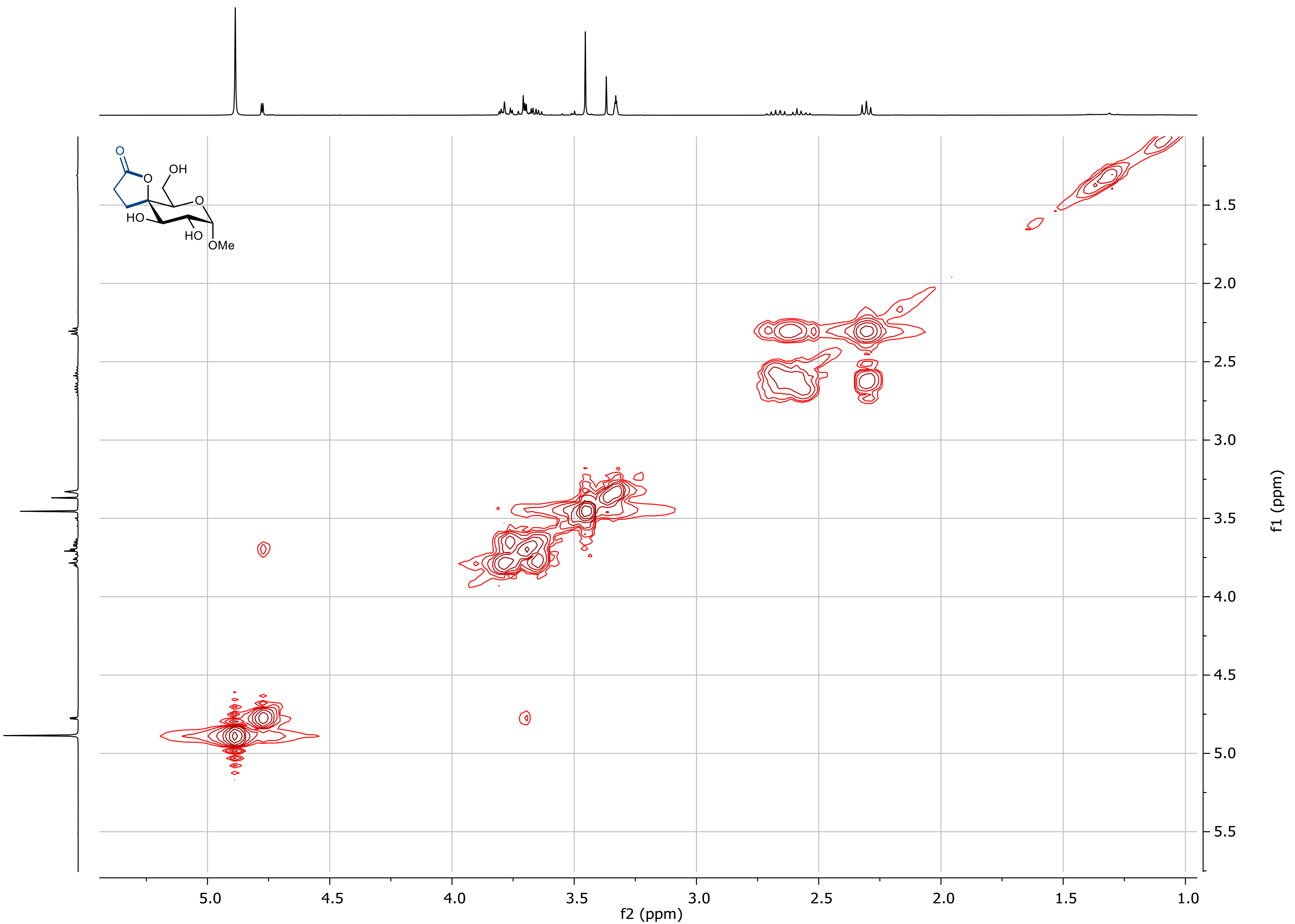

HSQC of compound **6a**

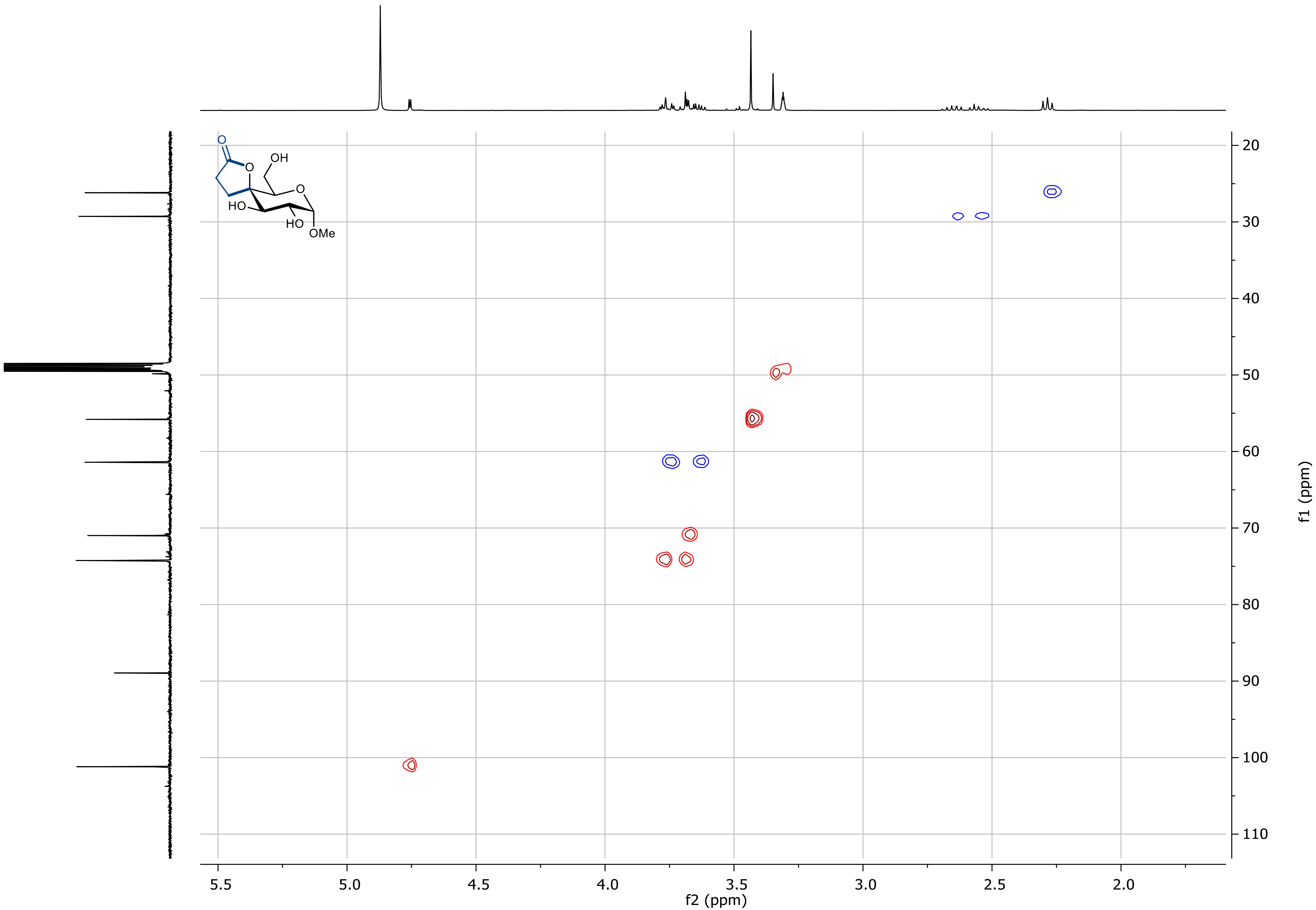

HMBC of compound **6a**

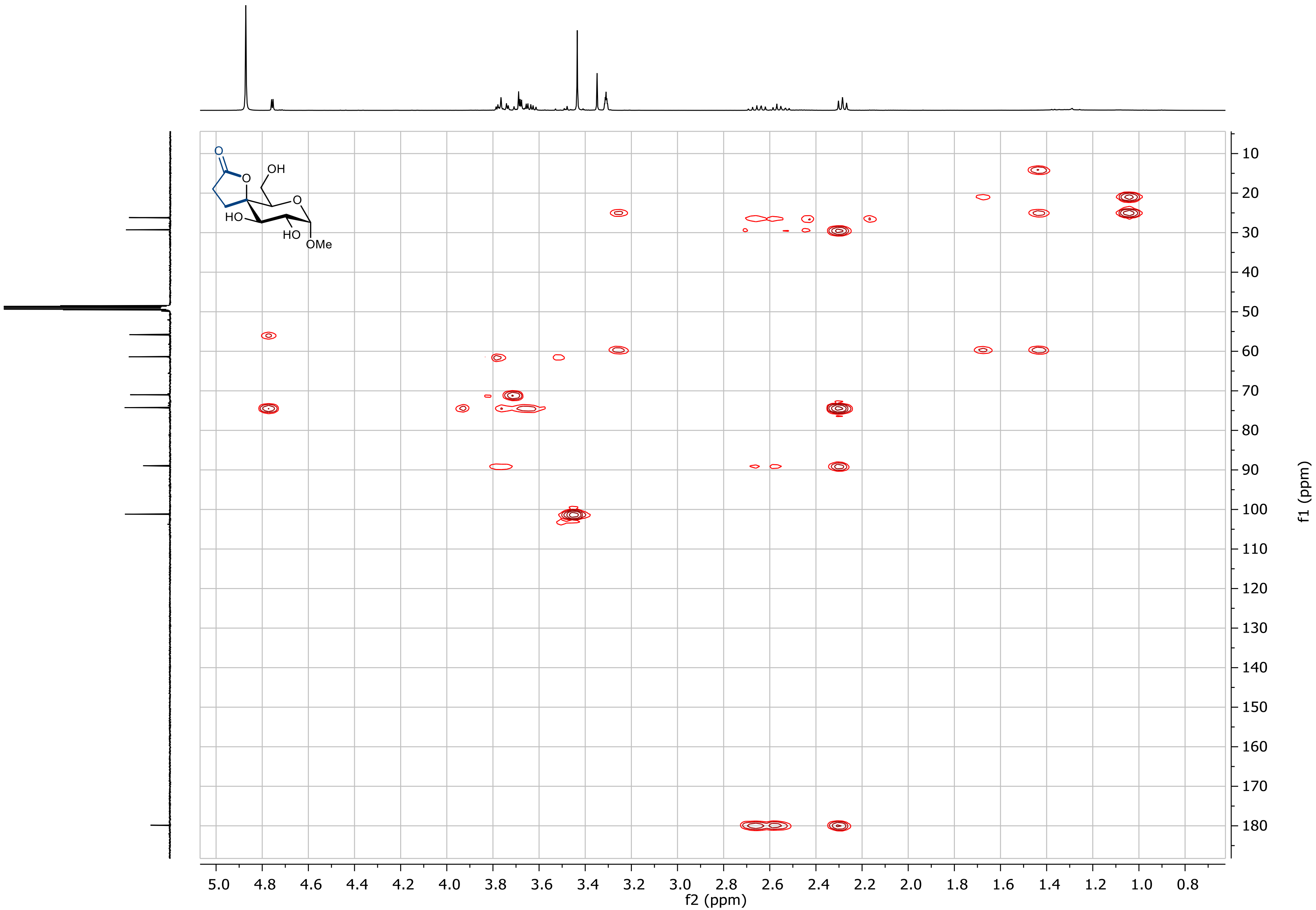

^{1}H NMR (500 MHz, $CDCl_3$) of compound **7a**

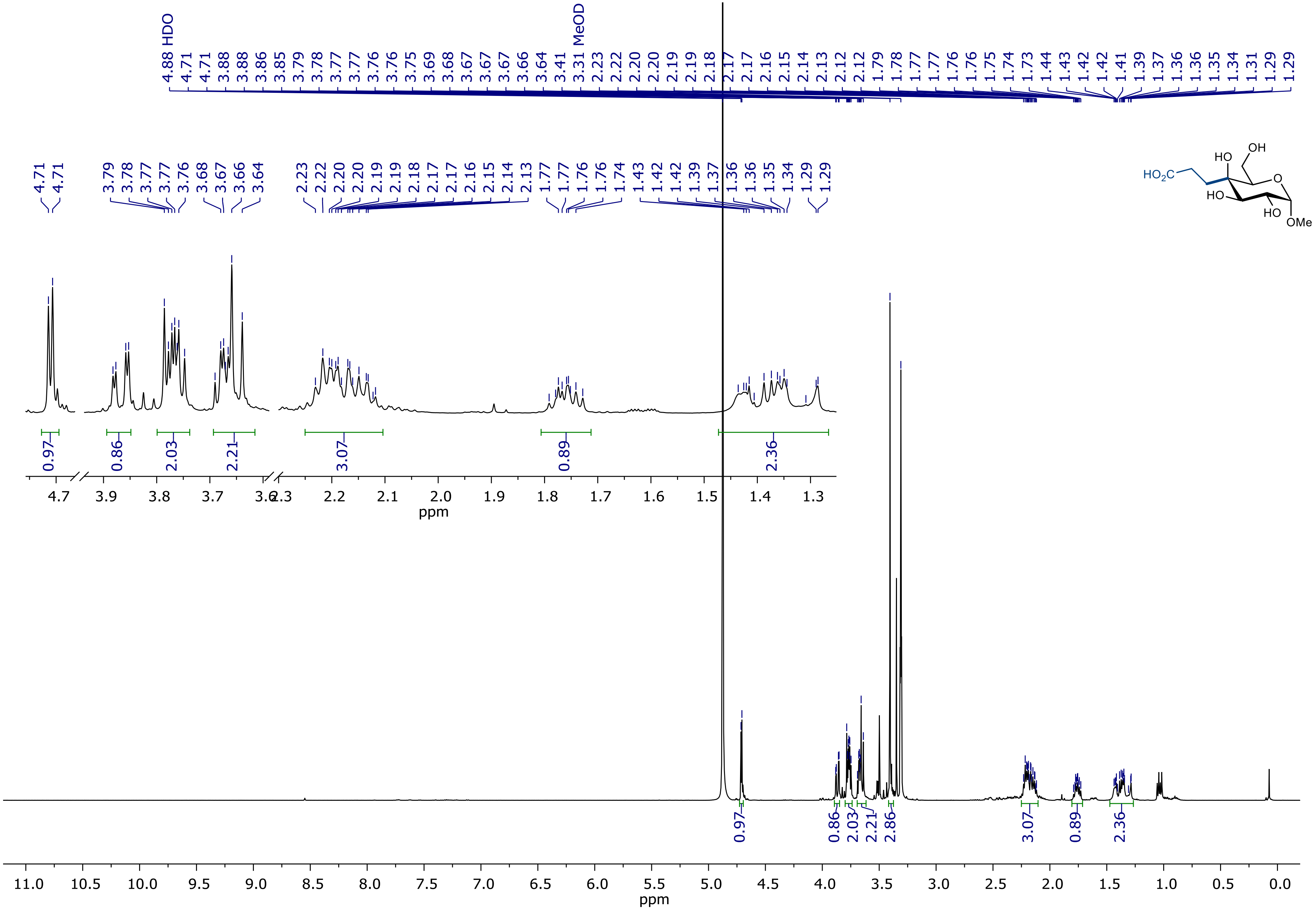

$^{13}C\{^{1}H\}$ NMR (126 MHz, $CDCl_3$) of compound **7a**

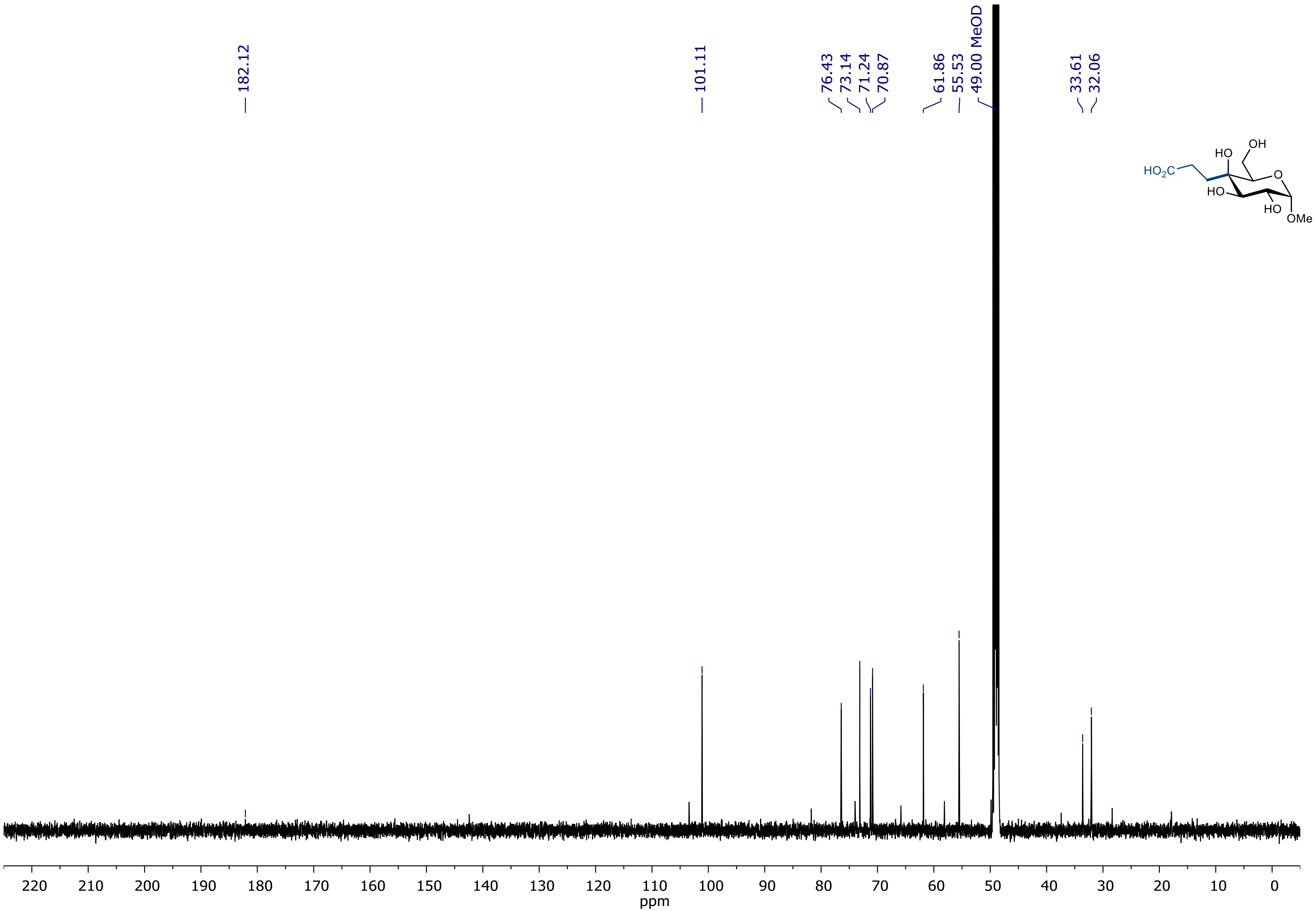

COSY of compound **7a**

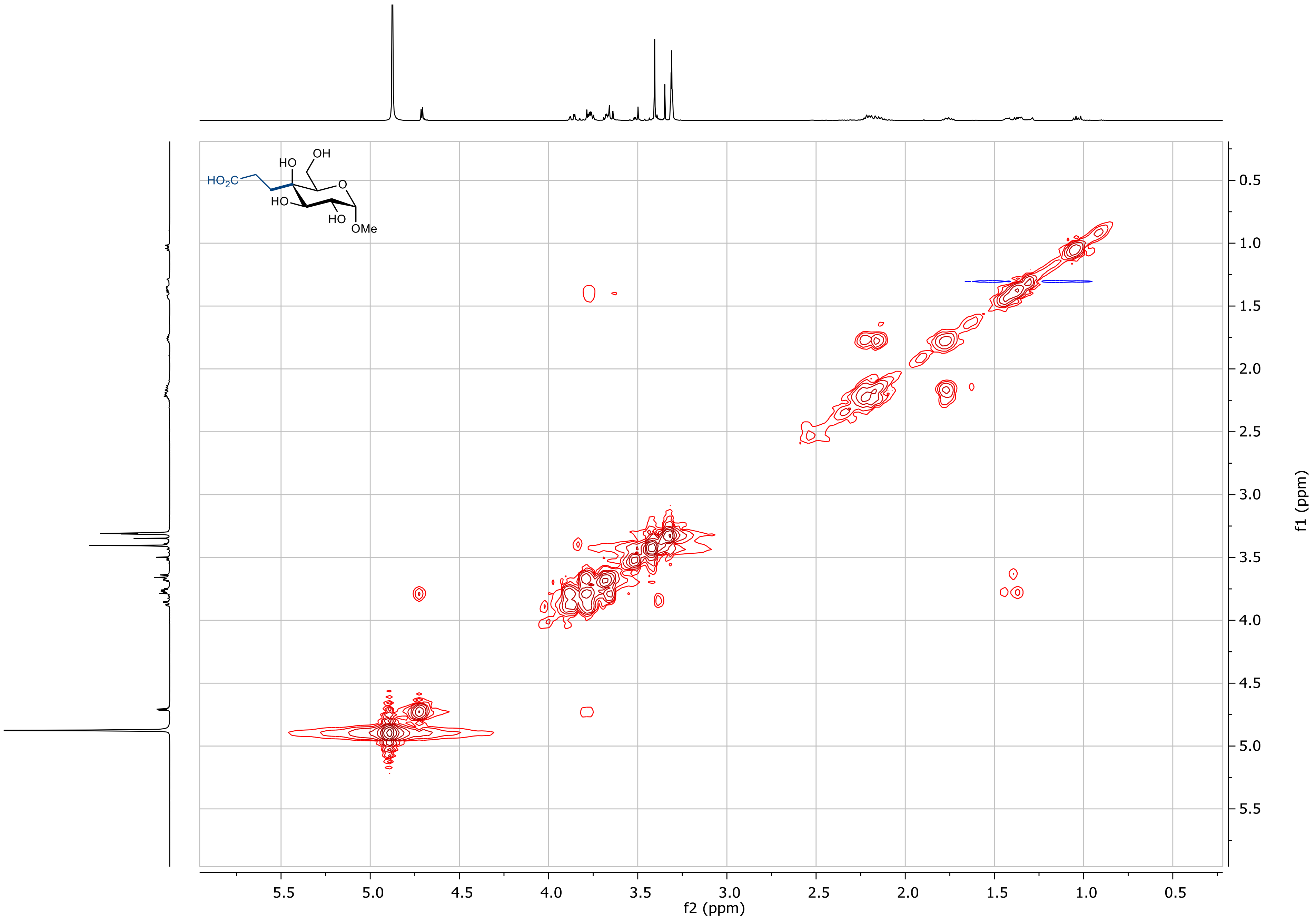

HSQC of compound **7a**

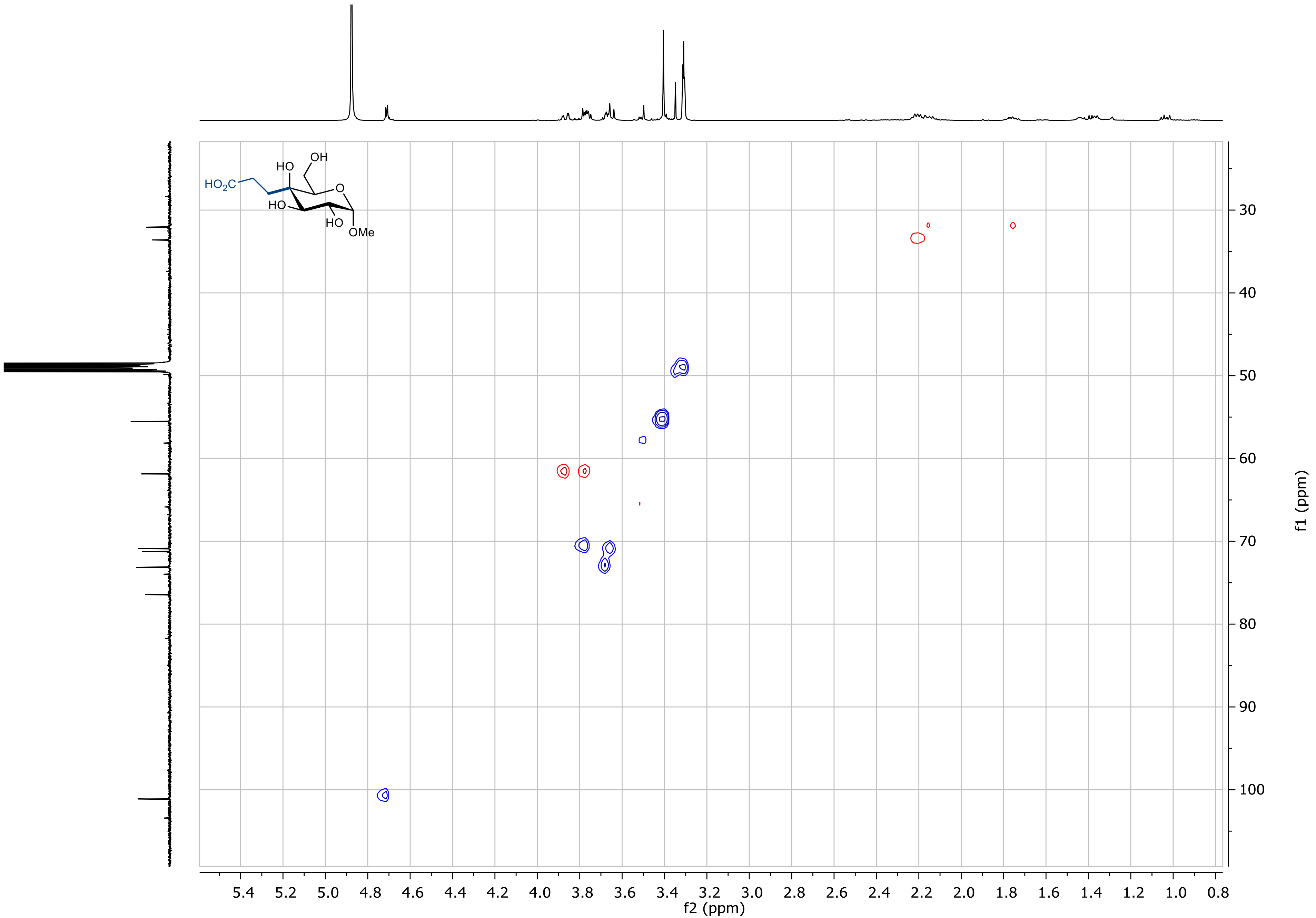

HMBC of compound **7a**

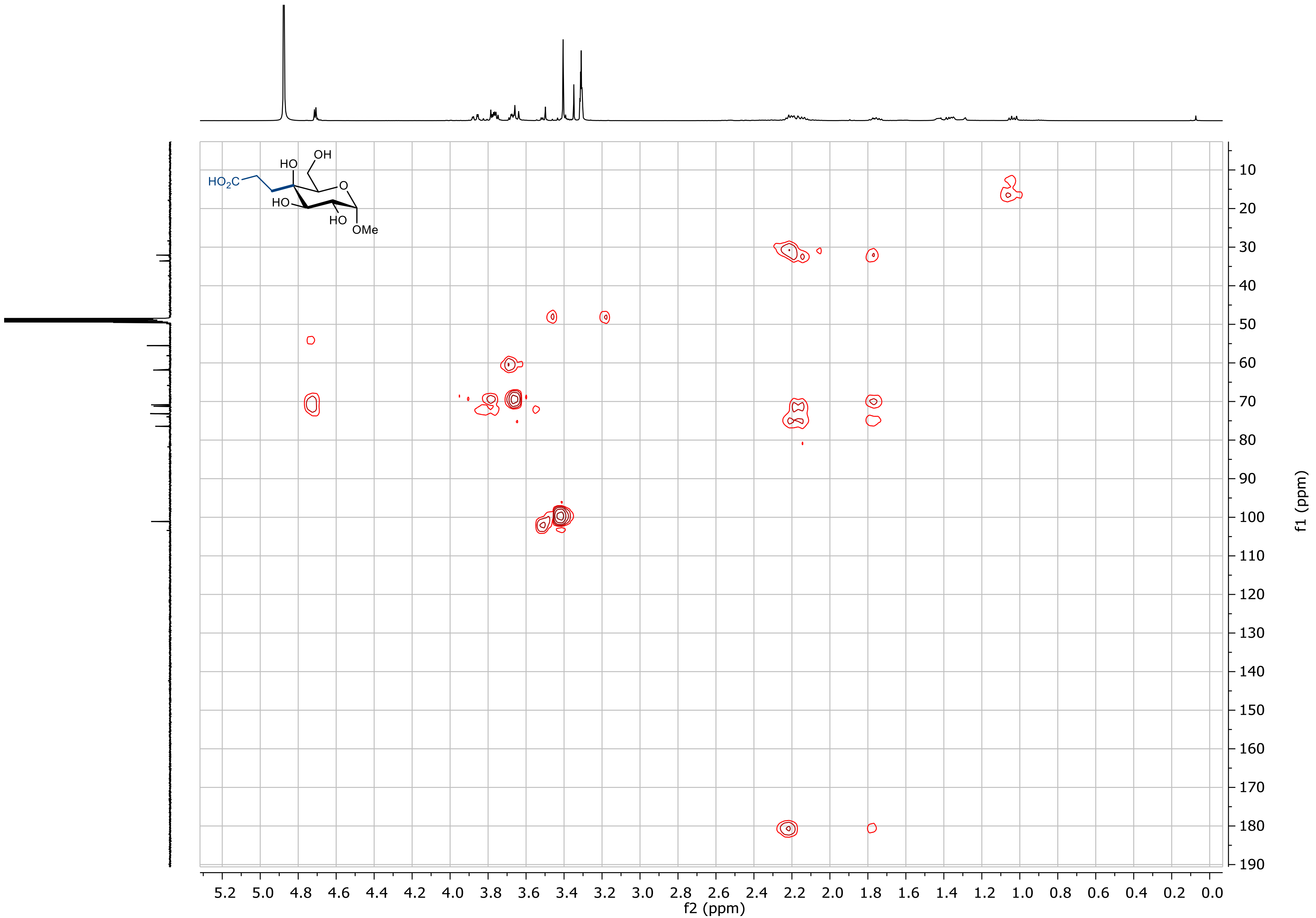

References

(1) Parasram, M.; Iaroshenko, V. O.; Gevorgyan, V. Endo-selective Pd-catalyzed silyl methyl Heck reaction. *J. Am. Chem. Soc.* **2014**, *136*, 17926–17929. https://doi.org/10.1021/ja5104525

(2) Braga, F. G.; Soares Coimbra, E.; de Oliveira Matos, M.; Lino Carmo, A. M.; Damato Cancio, M.; da Silva, A. D. Synthesis and biological evaluation of some 6-substituted purines. *Eur. J. Med. Chem.* **2007**, *42*, 530–537. https://doi.org/10.1016/j.ejmech.2006.10.014

(3) Rashid, A.; Mackie, W.; Colquhoun, I. J.; Lamba, D. Novel synthesis of monosulphated methyl α- D-galactopyranosides. *Can. J. Chem.* **1990**, *68*, 1122–1127. https://doi.org/10.1139/v90-173

(4) Betaneli, V. I.; Ott, A. Y.; Brukhanova, O. V.; Kochetkov, N. K. Synthesis of 1,2-O-cyanoethylidene derivatives of alkyl glycopyranuronates by oxidation of the 6-trityl ethers of their hexose analogues. *Carbohydr. Res.* **1988**, *179*, 37–50. https://doi.org/10.1016/0008-6215(88)84108-9

(5) Pongener, I.; Miller, G. J. D-glucuronate and D-glucuronate glycal acceptors for the scalable synthesis of D-GlcN-α-1,4-D-GlcA disaccharides and modular assembly of heparan sulfate. *J. Org. Chem.* **2023**, *88*, 11130–11139. https://doi.org/10.1021/acs.joc.3c01108

(6) Parlato, M. C.; Kamat, M. N.; Wang, H.; Stine, K. J.; Demchenko, A. V. Application of glycosyl thioimidates in solid-phase oligosaccharide synthesis. *J. Org. Chem.* **2008**, *73*, 1716–1725. https://doi.org/10.1021/jo701902f

(7) Zuo, A.-X.; Shen, Y.; Jiang, Z.-Y.; Zhang, X.-M.; Zhou, J.; Lü, J.; Chen, J.-J. Three new phenolic glycosides from *Curculigo orchioides* G. *Fitoterapia* **2010**, *81*, 910–913. https://doi.org/10.1016/j.fitote.2010.06.003

(8) Banks, M. R.; Cadogan, J. I. G.; Gosney, I.; Gould, R. O.; Hodgson, P. K. G.; McDougall, D. Preparation of enantiomerically pure fructose-derived 1,3-oxazin-2-one by INIR methodology and its application as a chiral auxiliary in some model asymmetric reactions. *Tetrahedron* **1998**, *54*, 9765–9784. https://doi.org/10.1016/S0040-4020(98)00531-6

(9) Hanaya, T.; Baba, H.; Toyota, H.; Yamamoto, H. Efficient total syntheses of natural pterin glycosides: limipterin and tepidopterin. *Tetrahedron* **2008**, *64*, 2090–2100. https://doi.org/10.1016/j.tet.2007.12.042

(10) Dahiya, A.; Schoetz, M. D.; Schoenebeck, F. Orthogonal olefination with organogermanes. *Angew. Chem. Int. Ed.* **2023**, *62*, e202310380. https://doi.org/10.1002/anie.202310380

(11) Sevrin, M. J.; Furst, L.; Nguyen, J. D.; Collins, J. L.; Stephenson, C. R. J. Lithium bis-catechol borate as an effective reductive quencher in photoredox catalysis. *Tetrahedron* **2018**, *74*, 3246–3252. https://doi.org/10.1016/j.tet.2018.04.053

(12) (a) Hendy, C. M.; Smith, G. C.; Xu, Z.; Lian, T.; Jui, N. T. Radical chain reduction via carbon dioxide radical anion ($CO_2^{•-}$). *J. Am. Chem. Soc.* **2021**, *143*, 8987–8992. https://doi.org/10.1021/jacs.1c04427 (b) Chmiel, A. F.; Williams, O. P.; Chernowsky, C. P.; Yeung, C. S.; Wickens, Z. K. Non-innocent radical ion intermediates in photoredox catalysis: Parallel reduction modes enable coupling of diverse aryl chlorides. *J. Am. Chem. Soc.* **2021**, *143*, 10882–10889. https://doi.org/10.1021/jacs.1c05988

(13) Wang, Y.; Carder, H. M.; Wendlandt, A. E. Synthesis of rare sugar isomers through site-selective epimerization. *Nature* **2020**, *578*, 403–408. https://doi.org/10.1038/s41586-020-1937-1

(14) Dimakos, V.; Su, H. Y.; Garrett, G. E.; Taylor, M. S. Site-selective and stereoselective C–H alkylations of carbohydrates via combined diarylborinic acid and photoredox catalysis. *J. Am. Chem. Soc.* **2019**, *141*, 5149–5153. https://doi.org/10.1021/jacs.9b01531

(15) (a) Lucarini, M.; Marchesi, E.; Pedulli, G. F.; Chatgilialoglu, C. Homolytic reactivity of group 14 organometallic hydrides toward nitroxides. *J. Org. Chem.* **1998**, *63*, 1687–1693. https://doi.org/10.1021/jo972178i (a) Le, C.; Chen, T. Q.; Liang, T.; Zhang, P.; MacMillan, D. W. C. A radical approach to the copper oxidative addition problem: Trifluoromethylation of bromoarenes. *Science* **2018**, *360*, 1010–1014. https://doi.org/10.1126/science.aat4133

(16) Adams, L. A.; Aggarwal, V. K.; Bonnert, R. V.; Bressel, B.; Cox, R. J.; Shepherd, J.; de Vicente, J.; Walter, M.; Whittingham, W. G.; Winn, C. L. Diastereoselective synthesis of cyclopropane amino acids using diazo compounds generated in situ. *J. Org. Chem.* **2003**, *68*, 9433–9440. https://doi.org/10.1021/jo035060c
